# A genome-wide analysis of the RNA-guided silencing pathway in coffee reveals insights into its regulatory mechanisms

**DOI:** 10.1371/journal.pone.0176333

**Published:** 2017-04-27

**Authors:** Christiane Noronha Fernandes-Brum, Pâmela Marinho Rezende, Thales Henrique Cherubino Ribeiro, Raphael Ricon de Oliveira, Thaís Cunha de Sousa Cardoso, Laurence Rodrigues do Amaral, Matheus de Souza Gomes, Antonio Chalfun-Junior

**Affiliations:** 1 Department of Biology, Section of Plant Physiology, Laboratory of Plant Molecular Physiology (LFMP), Federal University of Lavras (UFLA), Lavras, Minas Gerais, Brazil; 2 Departamento de Genética, Universidad de Córdoba (UCO), Córdoba, Spain; 3 Institute of Genetics and Biochemistry (INGEB),Laboratory of Bioinformatics and Molecular Analysis (LBAM), Federal University of Uberlândia (UFU)- Campus Patos de Minas, Patos de Minas, Minas Gerais, Brasil; Louisiana State University, UNITED STATES

## Abstract

microRNAs (miRNAs) are derived from self-complementary hairpin structures, while small-interfering RNAs (siRNAs) are derived from double-stranded RNA (dsRNA) or hairpin precursors. The core mechanism of sRNA production involves DICER-like (DCL) in processing the smallRNAs (sRNAs) and ARGONAUTE (AGO) as effectors of silencing, and siRNA biogenesis also involves action of RNA-Dependent RNA Polymerase (RDR), Pol IV and Pol V in biogenesis. Several other proteins interact with the core proteins to guide sRNA biogenesis, action, and turnover. We aimed to unravel the components and functions of the RNA-guided silencing pathway in a non-model plant species of worldwide economic relevance. The sRNA-guided silencing complex members have been identified in the *Coffea canephora* genome, and they have been characterized at the structural, functional, and evolutionary levels by computational analyses. Eleven AGO proteins, nine DCL proteins (which include a DCL1-like protein that was not previously annotated), and eight RDR proteins were identified. Another 48 proteins implicated in smallRNA (sRNA) pathways were also identified. Furthermore, we identified 235 miRNA precursors and 317 mature miRNAs from 113 MIR families, and we characterized *ccp-MIR156*, *ccp-MIR172*, and ccp-*MIR390*. Target prediction and gene ontology analyses of 2239 putative targets showed that significant pathways in coffee are targeted by miRNAs. We provide evidence of the expansion of the loci related to sRNA pathways, insights into the activities of these proteins by domain and catalytic site analyses, and gene expression analysis. The number of MIR loci and their targeted pathways highlight the importance of miRNAs in coffee. We identified several roles of sRNAs in *C*. *canephora*, which offers substantial insight into better understanding the transcriptional and post-transcriptional regulation of this major crop.

## Introduction

Small RNA (sRNA) silencing pathways have attracted increasing interest in the fields of genetics and molecular biology, and our current knowledge regarding the mechanisms and components involved in these pathways has rapidly evolved. Such RNA-based processes consist of sequence-specific inhibition of gene expression at the transcriptional or translational level by the action of small (20–26 nt) homologous RNA sequences [1].

Plant sRNAs are produced by processing of double-stranded duplexes from the helical regions of larger RNA precursors and are classified according to the intra- or intermolecular hybridization of the duplex [2]. microRNAs (miRNAs) are derived from self-complementary hairpin structures, while small-interfering RNAs (siRNAs) are derived from double-stranded RNA (dsRNA) or hairpin precursors [3, 4].

MIR genes are transcribed by RNA polymerase II (Pol II) [5] and undergo several modifications from transcription to maturity. Primary transcripts (pri-miRNAs) are similar to protein-coding RNA precursors (pre-mRNA) in size [6] but possess a hairpin structure that is stabilized by the RNA-binding protein DAWDLE (DDL) [7]. These molecules are processed by the endonuclease activity of DICER-LIKE 1 (DCL1) [8] into precursors (pre-miRNAs) assisted by additional enzymes, including HYPONASTIC LEAVES 1 (HYL1) [8], SERRATE (SE) [9, 10], and TOUGH (TGH) [11]. The pre-miRNAs are then processed by the DCL complex to form a duplex structure containing two 3’ nucleotide overhangs at each end. miRNAs are generally 21 nt long (DCL1 and DCL4), but their size varies depending on the DCL that induces cleavage, being 22 nt for DCL2 and 24 nt for DCL3 [12]. miRNAs negatively regulate their target genes through sequence-specific degradation or translational repression [13]. However, some miRNAs are also involved in DNA methylation [14].

The duplex is 3’ methylated by the methyltransferase HUA ENHANCER 1 (HEN1), which protects it from further modification and degradation [15]. The exportin HASTY (HST) is responsible for binding the duplex and transporting it from the nucleus to the cytoplasm [16]. Exportation in the absence of this protein is also possible but occurs via an unknown mechanism [17]. In the cytoplasm, one strand of the duplex is loaded onto an ARGONAUTE (AGO) family protein containing the PAZ and PIWI domains to form the RISC (RNA-Induced Silencing Complex). The PIWI domain possesses endonuclease activity and cleaves the target mRNA, which is also recognized by nearly perfect complementarity with the miRNA [12, 18].

The other major class of sRNAs, siRNAs, can act either at the transcriptional level by guiding DNA methylation or at the post-transcriptional level by guiding the cleavage and degradation of homologous cellular transcripts [1, 19]. RNA-dependent RNA Polymerases (RDRs) play an important role in siRNA production, synthesizing a second-strand RNA from the RNA template and thus producing a double-stranded RNA (dsRNA) molecule [20] with initial priming-dependent or priming-independent characteristics [21]. The biogenesis of siRNA shares a core mechanism with miRNAs. siRNAs are processed by a DCL protein (DCL2, DCL3, and DCL4), methylated by HEN1, and loaded onto a protein of the AGO family [2].

Additionally, two plant-specific DNA-Dependent RNA Polymerases, Pol IV and Pol V, are involved in the biogenesis of 24-nt siRNAs, which mediate RNA-Dependent DNA Methylation (RdDM). RdDM occurs through cytosine methylation (CG, CHG, and CHH, where H = A, C, or T) by the *de novo* methyltransferase DOMAINS REARRANGED METHYLTRANSFERASE 2 (DRM2) at the target DNA locus [22, 23]. Pol IV transcribes heterochromatic regions, which code for siRNAs [24], followed by dsRNA synthesis by RDR2, processing by DCL3, and the assembly of the resulting siRNA duplexes in the AGO4 clade of AGOs [23]. Pol V produces transcripts from Intergenic Non-coding (IGN) regions at loci that will be further methylated and is required for the recruitment of RdDM machinery, including DRM2 and siRNA-loaded AGO [25, 26]. This recruitment occurs by the interaction between protein-protein (Pol V-AGO) and nucleic acids, however, it remains unclear whether siRNA:IGN or siRNA:DNA. [27, 28].

Along with the core mechanism of sRNA production described above, using DCL in processing and AGOs as effectors, and additional participation of the RDR, Pol IV and Pol V in siRNA biogenesis, several other proteins interact with these core proteins to guide sRNA biogenesis, action, and turnover. These proteins have been recently reviewed [17, 19]. For instance, RECEPTOR FOR ACTIVATED C KINASE 1 (RACK1) and C-TERMINAL DOMAIN PHOSPHATASE-LIKE 1 (CPL1) interact with SE and have been implicated in pri-miRNA processing [29, 30]. Due to their recent emergence, the sRNA silencing pathways have not been fully elucidated, and knowledge of these pathways is constantly evolving. More recently, the protein REGULATOR OF CBF GENE EXPRESSION 3 (RCF3) has been described as a cofactor affecting miRNA biogenesis in specific plant tissues by interacting with CPL1 and CPL2 [31].

Aiming to expand the knowledge from model plants, the silencing complex has been identified in native and cultivated species, including rice (*Oryza sativa*) [32], common bean (*Phaseolus vulgaris*) [33], sorghum (*Sorghum bicolor*), and soybean (*Glycine max*) [34]. In *Coffea arabica* and *Coffea canephora*, the main economically important species of coffee, one of the most important crops in the world and the second most traded global commodity, MIR families have been identified based on Expressed Sequence Tags (EST), Genome Survey Sequences (GSS), and other transcript-based analyses [35–38].

With the release of the *C*. *canephora* genome, miRNAs were also identified [39]. However, the number of miRNAs was significantly underestimated. Moreover, the genes implicated in the generation and function of the miRNAs and siRNAs have not been described in coffee plants.

In this work, we present a thorough analysis of the identification and characterization of the small RNA-guided silencing complex in the *C*. *canephora* genome. Eleven AGO proteins; nine DCL-like proteins, including a previously unannotated DCL1; eight RDR proteins; and 48 other proteins implicated in the sRNA pathways, including HYL1, HST, HEN1, SE, and TGH, were identified. Furthermore, we conducted a conserved domain, catalytic site, and phylogenetic analysis to characterize the main proteins of the silencing pathway and validated their expression using RNA-seq libraries. We also identified 235 miRNA precursors producing 317 mature miRNAs belonging to 113 MIR families. We structurally and evolutionarily characterized and identified the putative targets of the MIR families *MIR156*, *MIR172*, and *MIR390*. A total of 2239 putative *C*. *canephora* miRNA targets were identified, and gene ontology analyses showed that significant pathways were targeted by miRNAs, demonstrating the importance of miRNAs in *C*. *canephora*.

The identification and analysis of the sRNA silencing pathways in *C*. *canephora* not only provide insights into the species but also provide a basis for further study of *C*. *canephora* and *C*. *arabica* regarding sRNA biogenesis and activity. The comprehension of these pathways in such an important crop provides insights into the species for further use of genetic engineering technologies available for crop breeding.

## Materials and methods

### miRNA and protein prediction datasets

The *C*. *canephora* genome data and genome features were accessed and downloaded from The Coffee Genome Hub [39]. Mature plant miRNA sequences and precursor miRNA sequences were downloaded from miRBase version 21. For protein prediction, Arabidopsis (*Arabidopsis thaliana*) ortholog sequences were retrieved from the nucleotide and protein databases at the NCBI (National Center for Biotechnology Information).

### Prediction of genes and proteins involved in the sRNA pathway in *C*. *canephora*

Putative proteins involved in the sRNA pathways were identified and selected by mining *C*. *canephora* sequences in the Coffee Genome Hub, an integrated web-based database, using the Basic Local Alignment Search Tool (BLAST) algorithm BLASTp with protein sequences from Arabidopsis as queries to search previously annotated protein-coding genes. The resulting protein sequences were retrieved for further analysis.

### Prediction of mature miRNAs and their precursors (pre-miRNAs)

To search for putative conserved miRNAs and their precursors, we applied an adapted algorithm previously described by de Souza Gomes *et al*. (2011) to the genome and transcriptome databases of *C*. *canephora* [40]. First, the genome and transcriptome sequences of *C*. *canephora* were searched using BLASTN to identify putative hairpin-like structures. The retrieved sequences were E-inverted (EMBOSS tool) using the maximum repeat parameters of 336 nucleotides and a threshold value of 25. Then, several filters were applied based on the thermodynamics and structural characteristics of known miRNAs. These filters included a GC content (guanine and cytosine) between 20% and 65%, Minimum Free Energy (MFE), homology with known mature miRNAs, homology to repetitive regions in RepeatMasker 4.0.2 [41], and homology to non-coding RNAs, such as rRNA, snRNA, SL RNA, SRP, tRNA, and RNase P, deposited in the Rfam microRNA Registry version 11.0 [42].

The sequences of pre-miRNAs identified in *C*. *canephora* were characterized according to their structures and thermodynamic parameters. The assessed parameters included the MFE, Adjusted Minimum Free Energy (AMFE), Minimum Free Energy Index (MFEI), size, A content, U content, C content, G content, GC and AU contents, GC ratio, AU ratio, Minimum Free Energy of the thermodynamic ensemble (MFEE), Ensemble Diversity (Diversity), and frequency of the MFE structure in the ensemble (Frequency). The adjusted MFE (AMFE) was determined to be a sequence of 100 nt, and the MFEI was determined using the equation MFEI = [(AMFE) X 100]/(G% + C%)] [43, 44]. The secondary structures of pre-miRNA, diversity, MFE, frequency ensemble, and MFE were predicted using RNA-fold software (http://rna.tbi.univie.ac.at/cgi-bin/RNAfold.cgi). The GC content and other structural properties were defined using Perl scripts.

### Analyses of the sRNA pathway proteins and miRNA precursors

The protein families, domains, and active sites were analyzed using PFAM (version 27.0, available at http://pfam.sanger.ac.uk) and the Conserved Domains Database (CDD; http://www.ncbi.nlm.nih.gov/cdd/). The protein sequences from *C*. *canephora* and their orthologs from different species were used to perform multiple sequence alignments using ClustalX 2.0 based on the default settings (available at http://www.clustal.org/clustal2/; [45]). The homologs and the *C*. *canephora* pre-miRNAs were aligned using ClustalX 2.0 based on the following alignment parameters: a gap opening of 22.50 and a gap extension of 0.83. They were also aligned in RNAalifold (http://rna.tbi.univie.ac.at/cgi-bin/RNAalifold.cgi). Phylogenetic trees were inferred using the neighbor-joining method, and sequence divergence was estimated using the Jones–Taylor–Thornton model for proteins [46] and Kimura’s (1980) two-parameter model for pre-miRNAs [47]. Statistical reliabilities of the internal branches were assessed using 2000 bootstrap replicates for proteins and 5000 bootstrap replicates for pre-miRNAs with values greater than 30 above the branches. Molecular phylogenetic analyses were conducted using MEGA 5 software [48]. The catalytic domains of ARGONAUTE and DICER-like proteins were aligned using Clustal Omega. Pictures highlighting the catalytic residues were generated from the alignment. Multiple Em for Motif Elicitation (MEME) (Version 4.11.2) [49] was then used to find RDR-like catalytic motifs.

### RNA-seq analysis

RNA-seq libraries were downloaded from the SRA (https://www.ncbi.nlm.nih.gov/sra/?term=ERP003741) for the three leaf stages (young, expanded, and old) and stems of the *C*. *canephora* samples.

For *CcDCL1* prediction, the RNA-seq libraries were assembled using Trinity [50]. BLASTN was run against the assembled data using AtDCL1 as a query. The six retrieved sequences were re-assembled using CAP3 [51], and two novel contigs were formed. The protein sequence of the largest contig was predicted using GenScan (http://genes.mit.edu/GENSCAN.html).

For expression validation, the transcriptome in different tissues was assembled using the alignment of the RNA-seq reads against the *C*. *canephora* genome with the software TopHat2. The subsequent identification of new genes and alternative splicing analysis were completed with the Cufflinks package. After alignment, possible coding sequences were extracted and identified with the Trans Decoder algorithm and subjected to homology analysis with BLAST. After selecting the proteins involved in the sRNA pathways, differential expression analysis was conducted with the CuffDiff software. The results were visualized and plotted using several packages of the statistical environment R, including the cummeRbund package.

### Prediction of *C*. *canephora* miRNA target genes

To search for putative target genes of the predicted miRNAs in *C*. *canephora*, transcript (CDS+UTR) sequences were retrieved from the Coffee Genome Hub (http://coffee-genome.org/download) and from RNA-seq libraries (transcript-predicted) of two tissue types: leaves and stem. *C*. *canephora* miRNA target genes were predicted using the webtool psRNATarget [52]. To avoid false-positive predictions for the miRNA target genes, we used a stringent cutoff threshold for a maximum expectation of 2.0. The other parameters were based on default settings, which included a length for complementarity scoring (hspsize) of 20 bp, top number of target genes for each small RNA of 200, target accessibility, maximum energy to unpair the target site (UPE) of 25, flanking length around the target site for target accessibility analysis of 17 bp upstream/13 bp downstream, and a range of the central mismatch leading to translational inhibition of 9–11 nt.

Using the RNA-seq sequences, BLAST2GO was run with the resulting predicted targets for each of the miRNAs *MIR156*, *MIR172*, and *MIR390*. BLAST2GO began with a BLASTP search against SwissProt, followed by mapping and annotation.

GO classes of the miRNA targets were classified and grouped using the web tool SEA (Singular Enrichment Analysis) from agriGO (http://bioinfo.cau.edu.cn/agriGO/index.php) [53]. The input was the target genomic IDs, which were compared against all of the IDs of the Coffee Genome Hub.

## Results

### sRNAs pathways proteins prediction and validation

The proteins involved in the miRNA pathways were identified by BLASTP in the Coffee Genome using Arabidopsis orthologs as queries. The components of the miRNA pathway, HYL1, SE, DDL and TGH [7, 9–11], were identified, and one copy of each of these proteins was identified in the *C*. *canephora* genome (Table 1). Two core proteins of the sRNA pathways, HEN1 and HST, were also identified. One putative CcHEN1 and one CcHST protein were identified (Table 1). In addition, we also identified at least 48 proteins in the *C*. *canephora* genome associated with the sRNA pathways described in the literature (S1 Table).

**Table 1:** HYL1, SE, DDL, TGH, HEN1, and HST orthologs of *C*. *canephora*.

**Protein Name**	**ID Arabidopsis**	**Size (aa)**	***C*. *canephora*** **Locus name**	**Locus Position**	**Size (aa)**
DDL	NP_188691.1	314	Cc05_g13470	chr5:27034635..27039361	402
TGH	NP_001031926.1	900	Cc04_g07720	chr4:6122482..6132431	852
HYL1	NP_563850.1	419	Cc10_g15960	chr10:26908423..26911736	321
HEN1	NP_001190782.1	942	Cc09_g07800	chr9:10021237..10030396	951
SE	NP_565635.1	720	Cc01_g07580	chr1:25540845..25550602	761
HST	NP_187155.2	1202	Cc02_g32190	chr2:43066609..43081800	1199

Protein name, ID, and size in Arabidopsis, *C*. *canephora* locus name, position, and protein size

The core proteins of the sRNA pathways- DCL-like, AGO-like, and RDR-like—were identified and characterized as described below. The *C*. *canephora* protein name, locus position, length, and identity with their respective orthologs from Arabidopsis are presented in Table 2.

**Table 2:** The *Coffea canephora* DCL-like, AGO-like and RDR-like protein orthologs.

**Protein Name**	**ID Arabidopsis**	**Protein length (aa)**	**BLASTP** **(e-value) vs** ***A*. *thaliana***	**Identity**	***C*. *Canephora*** **ortholog**	**Locus**	**Location coordinates**	**Protein length (aa)**
AGO1	NP_171612.1	1060	0.0	84%	CcAGO1	Cc04_g08880	chr4:7327522..7334534	1070
AGO2	NP_174413.2	1014	0.0	48%	CcAGO2.2	Cc09_g06780	chr9:7781473..7787026	1103
	NP_174413.2	1014	0.0	46%	CcAGO2.1	Cc09_g06770	chr9:7773251..7777143	1072
AGO4	NP_001189613.1	924	4e^-81^	43%	CcAGO4.1	Cc04 g10830 Cc04 g10840	chr4:10274296..10280759	
AGO4	NP_001189613.1	924	0.0	74%	CcAGO4.2	Cc01_g06780	chr1:24122477..24129690	869
AGO4	NP_001189613.1	924	0.0	69%	CcAGO4.3	Cc00_g14230	chr0:103099681..103105365	867
AGO5					CcAGO5	Cc01_g10060	chr1:28754803..28760661	960
AGO7	NP_177103.1		0.0	69%	CcAGO7	Cc11_g12560	chr11:29570089..29573706	1014
AGO10	NP_001190464.1	988	0.0	81%	CcAGO10.1	Cc03_g04370	chr3:3329168..3336865	992
	NP_001190464.1	988	0.0	73%	CcAGO10.2	Cc06_g09120	chr6:7288302..7294655	932
AGO16					CcAGO16	Cc05_g02730	chr5:12039961..12045923	909
DCL1	NP_171612.1	1909	0.0	76%	CcDCL1	-	chr0:59461839..59481838	1747
DCL2	NP_566199.4	1388	0.0	55%	CcDCL2.1	Cc09_g03980	chr9:3364371..3376041	1352
	NP_566199.4	1388	0.0	47%	CcDCL2.2	Cc02_g14900 Cc02_g14910	chr2:13049228..13060040	778
	NP_566199.4	1388	3e^-112^	51%	CcDCL2.5	Cc06_g19770	chr6:21807446..21809500	354
	NP_566199.4	1388	0.0	48%	CcDCL2.6	Cc06_g19980	chr6:22425311..22432933	762
	NP_566199.4	1388	0.0	50%	CcDCL2.4	Cc02_g14930	chr2:13070716..13077527	802
	NP_566199.4	1388	0.0	48%	CcDCL2.3	Cc02_g14920	chr2:13060040..13066011	727
DCL3	NP_001154662.2	1580	0.0	48%	CcDCL3	Cc08_g06780	chr8:17408330..17423075	1584
DCL4	NP_001190348.1	1688	0.0	51%	CcDCL4	Cc06_g07320	chr6:5843020..5862408	1656
RDR1	NP_172932.1	1107	0.0	63%	CcRDR1.1	Cc11_g06970	chr11:23552744..23560803	1114
	NP_172932.1	1107	0.0	64%	CcRDR1.2	Cc11_g06940	chr11:23487397..23495045	1113
	NP_172932.1	1107	0.0	60%	CcRDR1.3	Cc11_g06960	chr11:23538795..23545065	1132
	NP_172932.1	1107	0.0	56%	CcRDR1.4	Cc11_g06950	chr11:23504270..23516759	1188
RDR2	NP_192851.1	1133	0.0	57%	CcRDR2	Cc00_g08850	chr0:76051887..76058404	1121
RDR3	NP_179581.2	992	0.0	43%	CcRDR3.1	Cc06_g10360	chr6:8381378..8392034	1020
	NP_179581.2	992	0.0	47%	CcRDR3.2	Cc06_g10350	chr6:8366687..8376181	876
RDR6	NP_190519.1	1196	0.0	67%	CcRDR6	Cc08_g00760	chr8:779886..784083	1050

Protein name, ID, and length in Arabidopsis, BLASTp e-value and Identity of *C*. *canephora* vs. Arabidopsis. *C*. *canephora* ortholog name, locus name, locus position, and protein length.

The number of DCLs may vary among species. For instance, there are five DCLs in poplar, maize (*Zea mays*), and sorghum (*S*. *bicolor*) [34, 54]; seven in tomato (*Solanum lycopersicum*) [55]; eight in rice (*O*. *sativa*) [56]; and six in common bean (*P*. *vulgaris*) [33].

The annotated protein-coding sequences identified from the BLASTP of the DCL-like search in the Coffee Genome Hub were retrieved, and conserved domain analysis revealed that nine of these sequences contained DCL-like conserved domains (Table 3). Two of the sequences (Cc02_14900 and Cc02_14910) that are sequential in chromosome 2 presented complementary domains of a DCL protein. Then, the genomic region comprising both contigs was retrieved, and the resulting protein was predicted using GenScan (http://genes.mit.edu/GENSCAN.html) and used for further analyses.

**Table 3:** Conserved domain analysis of the *C*. *canephora* DCL-like orthologs.

**Locus Name**	**Protein Name**	**DExD**	**Helicase-C**	**DUF283**	**PAZ**	**RIBOC**	**RIBOC**	**DSRM**	**DSRM**
-	CcDCL1	114–266	503–619	693–784	1029–1164	1201–1387	1423–1579	1582–1643	1674–1742
Cc09_g03980	CcDCL2.1	2–137	318–436	507–592	760–887	935–1087	1119–1272	-	-
Cc02_g14900 Cc02_g14910	CcDCL2.2	-	-	-	162–290	338–478	519–705	709–765	-
Cc06_g19770	CcDCL2.5	-	-	-	-	48–85	126–280	284–340	
Cc06_g19980	CcDCL2.6	-	-	-	174–291	339–490	524–679	685–738	-
Cc02_g14930	CcDCL2.4	-	-	-	177–305	353–497	538–692	-	-
Cc02_g14920	CcDCL2.3	-	-	-	153–273	321–465	506–660	664–723	-
Cc08_g06780	CcDCL3	53–215	406–524	603–690	889–1037	1079–1243	1289–1439	-	-
Cc06_g07320	CcDCL4	81–232	412–534	606–683	873–993	1041–1204	1242–1386	1395–1459	1572–1645

Multiple alignments with ortholog DCLs from other angiosperm species and phylogenetic analyses were performed to assign the coffee DCLs and to determine the evolutionary relationship among species. One DCL3, one DCL4, and six DCL2s were assigned. No DCL1 was found using this approach, then we identified one putative CcDCL1 from RNA-seq libraries. Conserved domain analysis (Table 3) of the resulting sequence confirmed a DCL protein, and BLASTP at the NCBI database matched DCL1 proteins with 99% coverage and an E-value of 0. The sequence was then searched by tBLASTN in the Coffee Genome Hub and aligned with a genomic sequence in chromosome 0, an arbitrary pseudochromosome created with all of the unmapped sequences from the 11 chromosomes [39] (S1 Fig). Therefore, although present in the genome assembly, the CcDCL1 was not previously annotated as a protein-coding gene on the Coffee Genome Hub.

**Fig 1:**
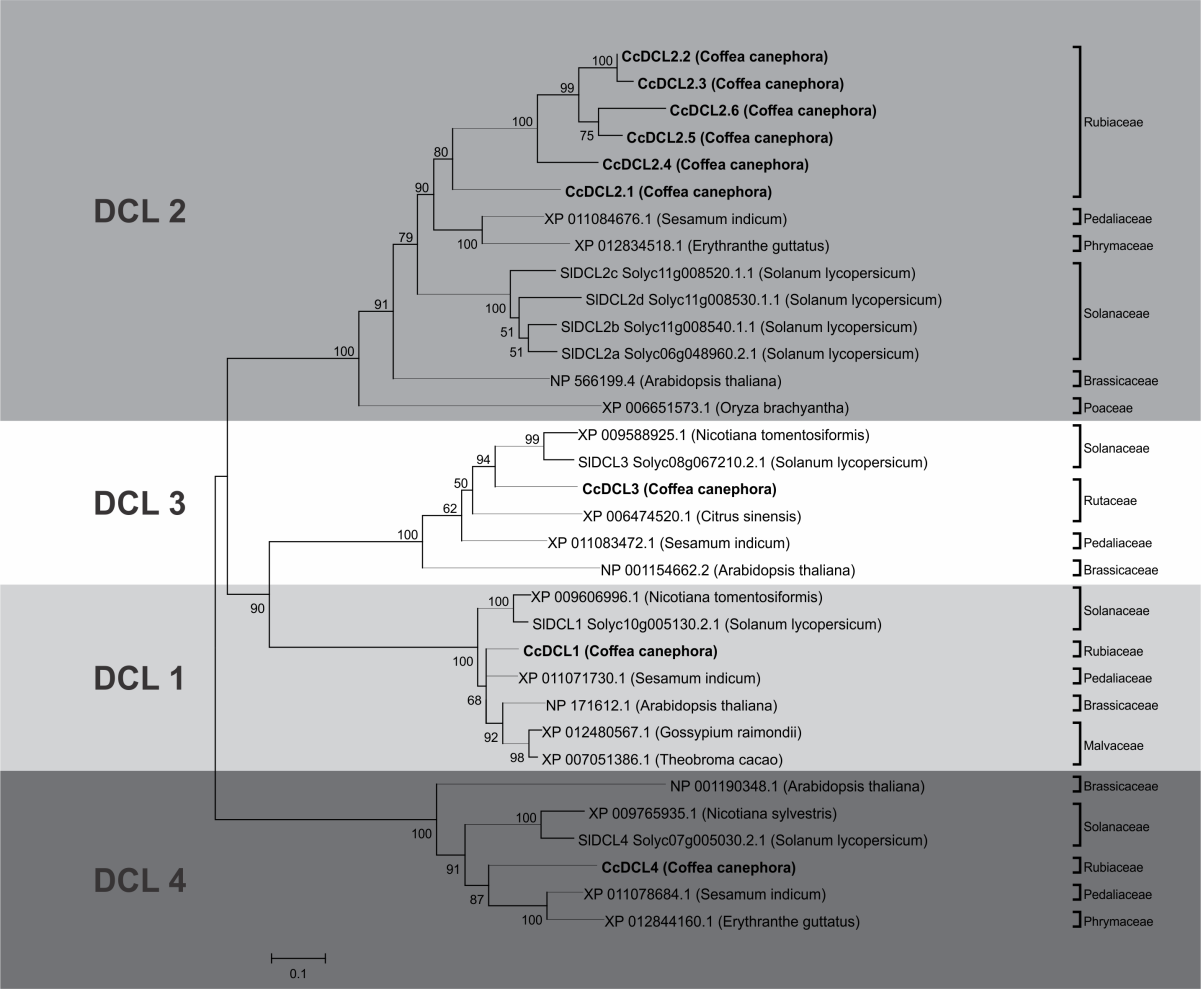
Phylogenetic tree of DCL-like proteins identified in *Coffea canephora*. Phylogenetic tree showing relationships between the paralogous and orthologs proteins of the DCL family. The evolutionary history was inferred using the Neighbor-Joining method [46]. The bootstrap consensus tree inferred from 2000 replicates is taken to represent the evolutionary history of the taxa analyzed. Branches corresponding to partitions reproduced in less than 50% bootstrap replicates are collapsed. The percentage of replicate trees in which the associated taxa clustered together in the bootstrap test (2000 replicates) are shown next to the branches. The tree is drawn to scale, with branch lengths in the same units as those of the evolutionary distances used to infer the phylogenetic tree. The evolutionary distances were computed using the JTT matrix-based method and are in the units of the number of amino acid substitutions per site [48]. The analysis involved 33 amino acid sequences. All positions containing gaps and missing data were eliminated. There were a total of 286 positions in the final dataset.

The new phylogenetic analysis, including the putative CcDCL1, generated a tree in which the CcDCL clustered similarly to their respective orthologs from other species (Fig 1). In total, nine DCL-like proteins were found in the *C*. *canephora* genome (Table 2) and were distributed in four distinct clades in the phylogenetic tree (Fig 1); the clades matched the four paralogous DCL-like proteins described in Arabidopsis [57].

The DCL proteins have six domains types, DExD-helicase (DExDc), Helicase-C (HELICc), Duf283, PAZ, RNAse III (RIBOc), and double-stranded RNA-binding (dsRB), although some of these may not be present [58]. Conserved domain analysis (Table 3) revealed that the CcDCL1-like and CcDCL4-like proteins contain DExD, Helicase-C, Dicer-dimer, PAZ, two RNAse III (RIBOc), and two dsRB (DSRM) domains. The CcDCL3-like, CcDCL2.1-like, and DCL4-like proteins contain no DSRM domains. The CcDCL2 proteins have five more paralogs, which appear to be partial sequences lacking the N-terminal domains (DExD, Helicase-C, and DUF283). These sequences also lack one (CcDCL2.3, CcDCL2.4, and CcDCL 2.6) or two (CcDCL2.5) DSRM domains. The shortest CcDCL2-like protein, CcDCL2.3, also lacks a PAZ domain.

**Fig 2:**
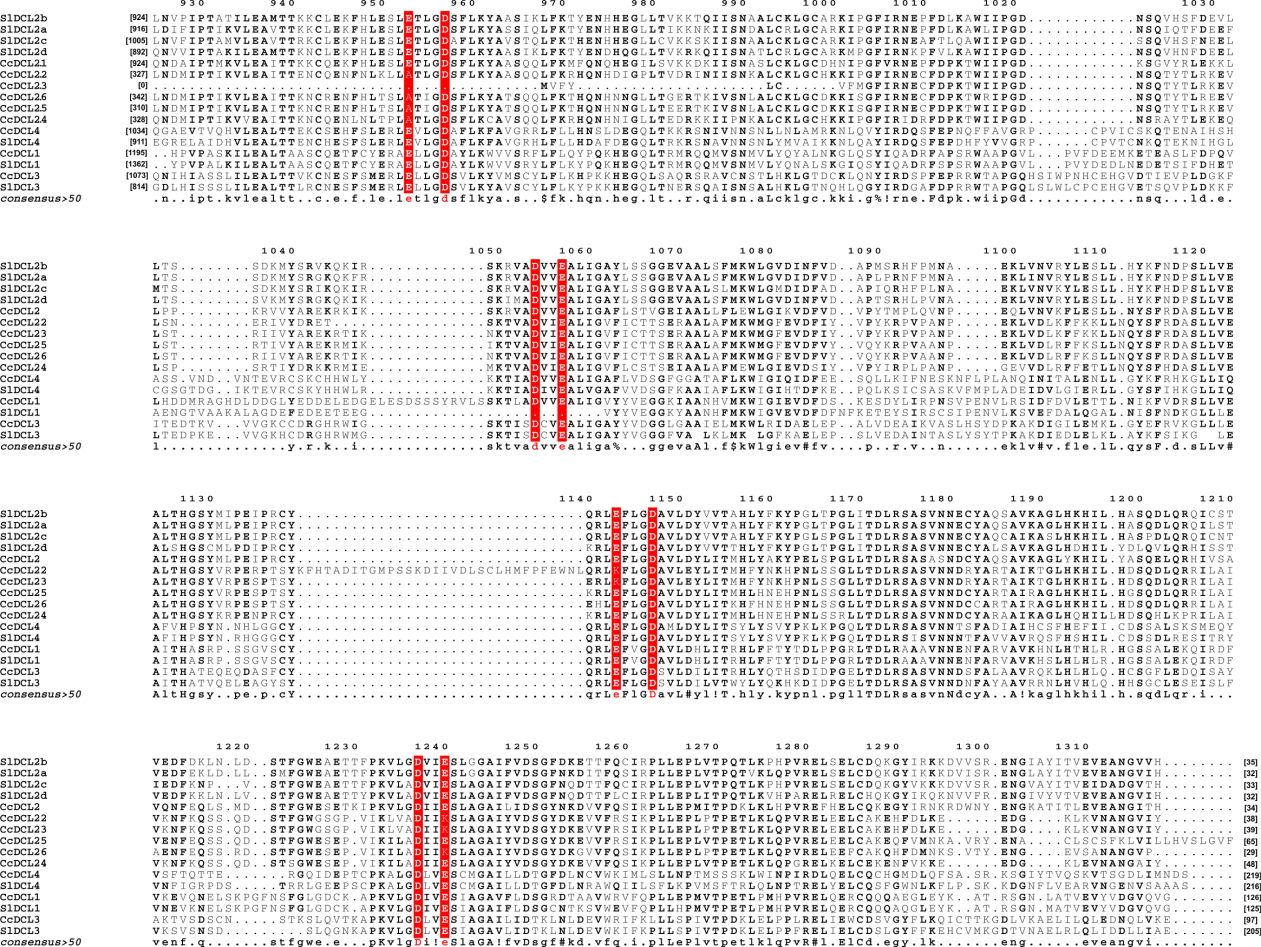
Analysis of the catalytic residues of the CcDCL-like proteins. The two RNase III domains (RIBOc I and II) at the glutamate (E), aspartate (D), glutamate (D), aspartate (E) (EDDE) position. The catalytic sites are highlighted.

We also analyzed the conservation of the RNase III catalytic sites of CcDCL-like proteins in the two RNase III domains (RIBOc I and II): glutamate (E), aspartate (D), glutamate (D), aspartate (E) (EDDE) [59]. CcDCL1, CcDCL2.1, CcDCL3, and CcDCL4 contain these conserved catalytic residues (Fig 2).

ARGONAUTES have been observed in variable numbers in plants. For instance, there are 10 AGOs in Arabidopsis [60], 22 in soybean (*G*. *max*) [34], 17 in common bean (*P*. *vulgaris*) [33], 19 in rice (*O*. *sativa*) [32], and 17 in maize (*Z*. *mays*) [54]. A BLASTP search using AtAGO as a query in the Coffee Genome Hub resulted in 12 *C*. *canephora* protein-coding sequences, which were retrieved and subjected to Conserved Domain analysis to confirm the presence of the conserved domains of ARGONAUTE proteins (N-terminal, PAZ, ArgoMid, and PIWI). Two of the sequences (Cc04_g10830 and Cc04_g10840) that were found sequentially in Chromosome 4 presented as partial sequences, one containing a PIWI domain (Cc04_g10830) and the other containing a PAZ (Cc04_g10840) domain. The genomic sequence comprising both contigs was retrieved, and the protein product was predicted using GenScan (http://genes.mit.edu/GENSCAN.html). BLASTP and Conserved Domain analysis confirmed an AGO protein that was considered for further analyses. Therefore, in total, eleven putative AGO proteins comprising seven homologs were found in *C*. *canephora* (Table 2).

**Fig 3:**
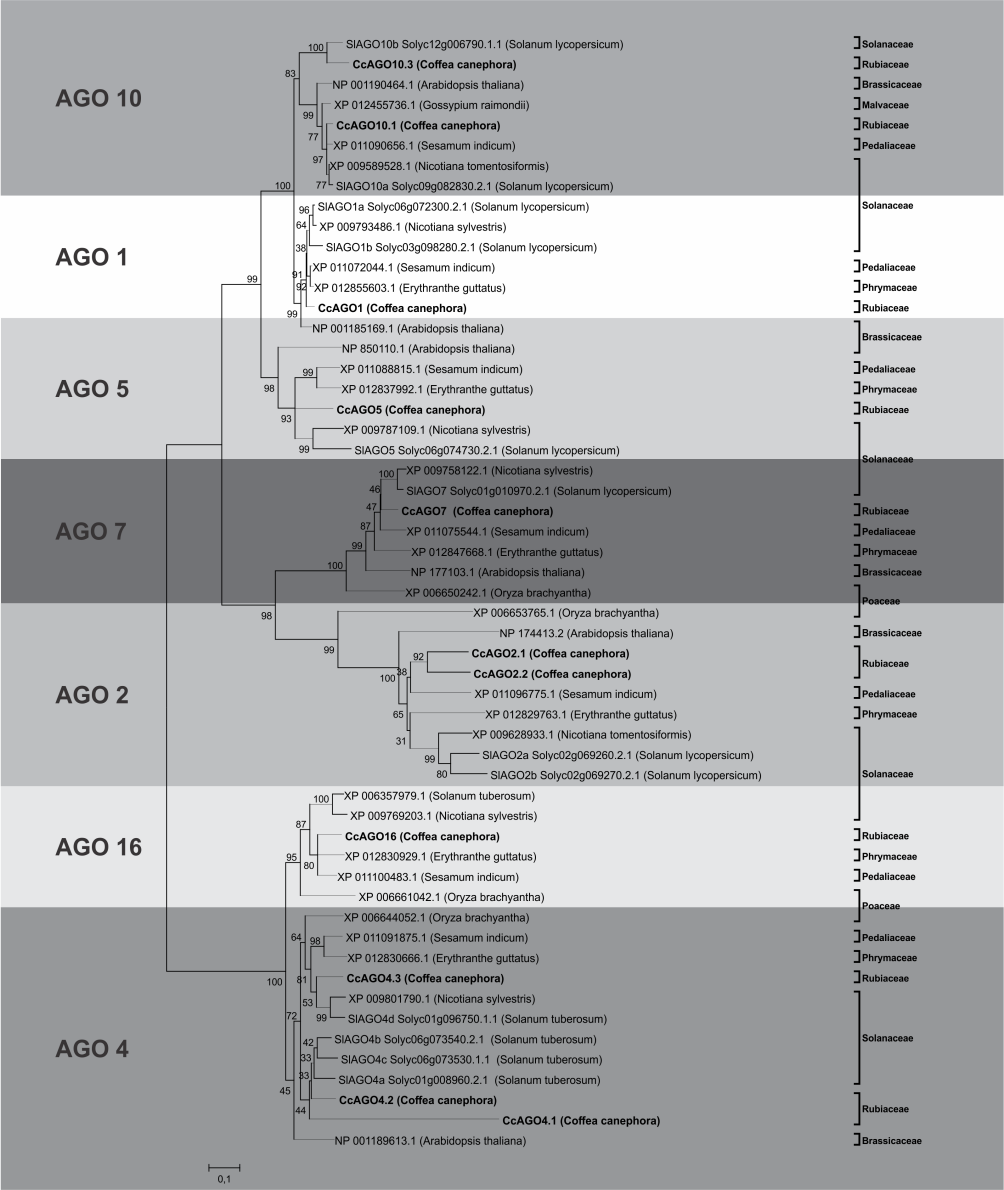
Phylogenetic tree of AGO proteins identified in *Coffea canephora*. Phylogenetic tree showing relationships between the paralogous and orthologs proteins of the AGO family. The evolutionary history was inferred using the Neighbor-Joining method [46]. The bootstrap consensus tree inferred from 2000 replicates is taken to represent the evolutionary history of the taxa analyzed. Branches corresponding to partitions reproduced in less than 50% bootstrap replicates are collapsed. The percentage of replicate trees in which the associated taxa clustered together in the bootstrap test (2000 replicates) are shown next to the branches. The evolutionary distances were computed using the JTT matrix-based method and are in the units of the number of amino acid substitutions per site [48]. The analysis involved 55 amino acid sequences. All positions containing gaps and missing data were eliminated. There were a total of 333 positions in the final dataset.

Conserved domain analysis confirmed the presence of the N-terminal, PAZ, and PIWI domains in all sequences but showed an only variable presence of ArgoMid (Table 4). AGO1 proteins have an additional glycine-rich region at the N-terminus (Gly-rich_Ago1), which was present in one putative AGO sequence. To further determine the evolutionary conservation and assign the AGO-like proteins found in *C*. *canephora*, we compared the sequences to orthologs from other angiosperm species on a phylogenetic tree. The eleven AGO proteins were assigned and found to cluster with their closest orthologs from other species; the *C*. *canephora* AGO proteins also similarly grouped into three major phylogenetic clades [17, 61]: one AGO1, one AGO5, and two AGO10s in Clade I; two AGO2s and one AGO7 in Clade II; and three AGO4s in Clade III (Fig 3). One AGO16 was also identified, which grouped with the AGO4s in Clade III. A similar pattern has been found in rice, maize, Arabidopsis, soybean, sorghum, and other species, indicating the conservation of small RNA functions in higher plants [34].

**Table 4:** Identification of the conserved domains and their start and end positions in the *C*. *canephora* AGO orthologs.

**Locus Name**	**Protein Name**	**Gly-rich_Ago1**	**ArgoN**	**PAZ**	**ArgoMid**	**Piwi**
Cc04 g08880	CcAGO1	76–186	205–341	407–532	600–674	694–1013
Cc09 g06780	CcAGO2.2	-	253–393	458–581	-	758–1052
Cc09 g06770	CcAGO2.1	-	218–362	426–551	-	728–1022
Cc04 g10830/Cc04 g10840	CcAGO4.1	-	62–184	264–355	-	355–465
Cc01 g06780	CcAGO4.2	-	3–172	238–368	432–495	522–828
Cc00 g14230	CcAGO4.3	-	4–172	238–366	-	520–827
Cc01 g10060	CcAGO5	-	117–257	322–441	508–583	601–919
Cc11 g12560	CcAGO7	-	151–308	380–502	-	666–972
Cc03 g04370	CcAGO10.1	-	136–279	350–471	538–615	630–949
Cc06 g09120	CcAGO10.2	-	88–227	305–419	486–563	578–896
Cc05 g02730	CcAGO16	-	38–202	269–399	-	553–868

To investigate whether CcAGOs possess conserved catalytic residues and could potentially act as the slicer component of RISC, we aligned the PIWI domains of all of the CcAGOs and searched for the Asp-Asp-His (DDH) catalytic triad in CcAGOs and for a residue corresponding to the conserved H798 residue of AtAGO1 [62]. Four proteins (CcAGO1, CcAGO5, CcAGO7, and CcAGO10.1) possessed the conserved DDH/H798 residues (Table 5). In four CcAGOs, the DDH catalytic motif was conserved, but the H798 was replaced by a serine (CcAGO16), proline (CcAGO4.2 and CcAGO4.3), or glutamine (CcAGO10.2). Two CcAGO proteins contained an aspartate residue in place of the third histidine of the DDH motif (CcAGO2.1 and Cc AGO2.2). CcAGO4.1 contained neither the catalytic DDH nor the H798 residue. The detailed alignment of the PIWI domain is presented in S2 Fig.

**Table 5:** Analysis of active site amino acids and their respective position in the conserved PIWI domain (PF02171) from the CcAGO proteins.

CcAGO	Motifs*	POSITION
CcAGO1	DDH/H	777-863-1003/815
CcAGO2.1	DDD/H	807-880-1014/845
CcAGO2.2	DDD/H	837-910-1045/875
CcAGO4.1	ENR/R	384-445-489/422
CcAGO4.2	DDH/P	603-686-818/641
CcAGO4.3	DDH/P	601-684-816/639
CcAGO5	DDH/H	683-769-909/721
CcAGO7	DDH/H	750-823-963/788
CcAGO10.1	DDH/H	713-799-939/751
CcAGO10.2	DDH/Q	661-747-887/699
CcAGO16	DDH/S	634-725-857/672

*Motifs show the residues in *C*. *canephora* AGO proteins that correspond to D760, D845, H986/H798 of AtAGO1

**Fig 4:**
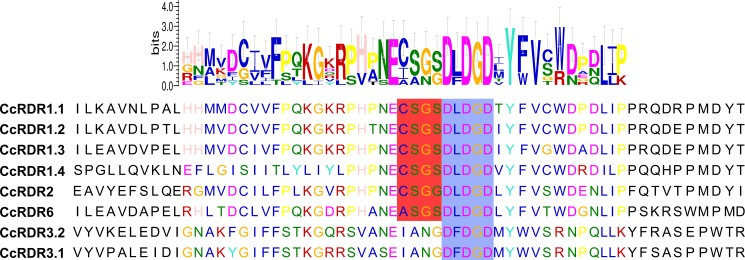
Analysis of the DxDGD catalytic motif of the RNA-dependent RNA polymerase (RdRP) conserved domain. Six coffee RDR possess a DLDGD motif (CcRDR1.1–1.4, CcRDR2 and CcRDR6) and two have the DFDGD motif (CcRDR3.1 and CcRDR3.2), corresponding to the RDRα clade and the RDRγ clade, respectively (Blue box). Additionally, the RDRα displays a conserved subsequences (C/A)SG(S/G) before the DLDGD motif and, all CcRDR1 and the CcRDR2 showed the CSGS sequence, while CcRDR6 showed the ASGS sequence (red box).

**Fig 5:**
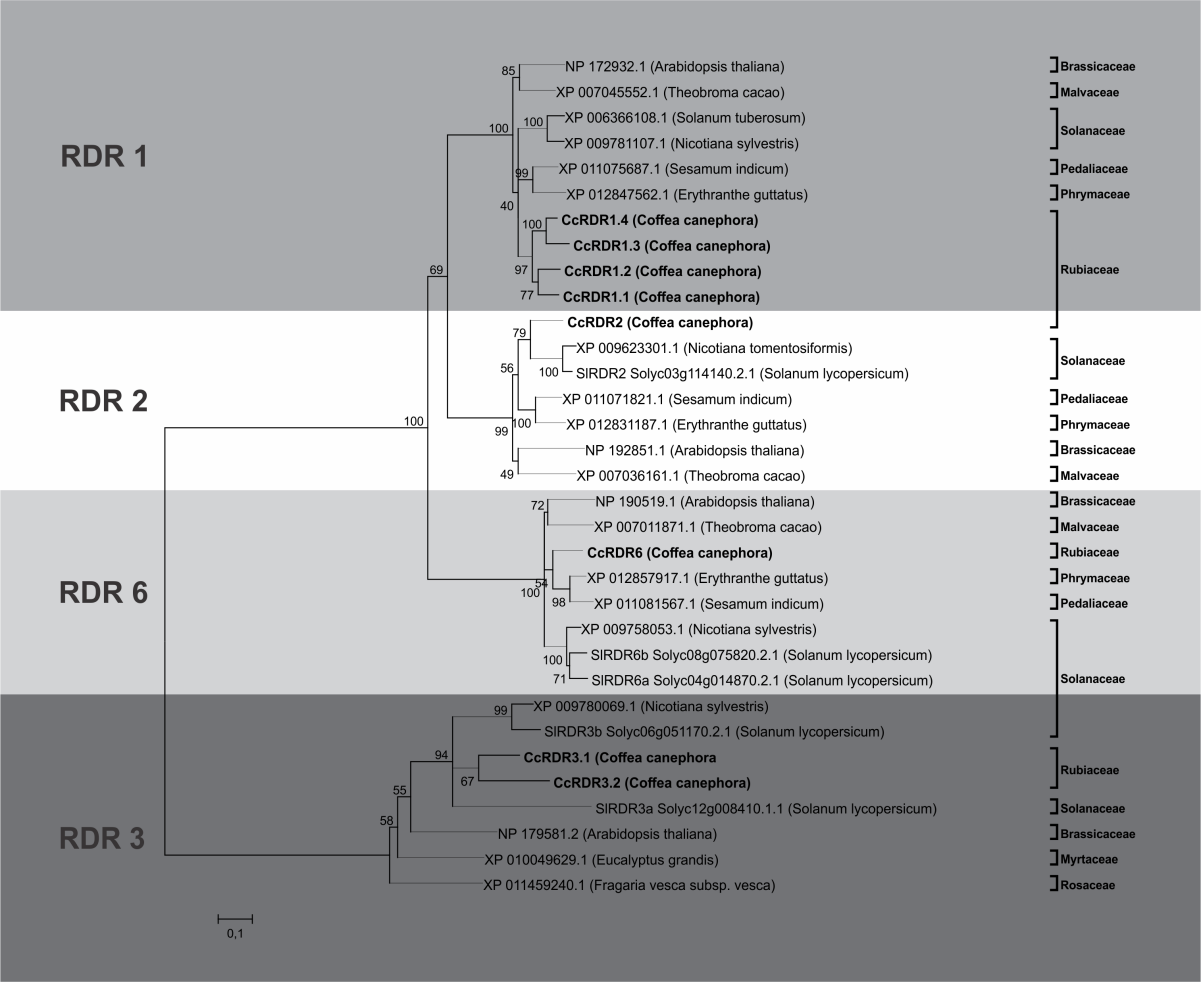
Phylogenetic tree of RDR proteins identified in *C*. *canephora*. Phylogenetic tree showing relationships between the paralogous and orthologs proteins of the RDR family. The evolutionary history was inferred using the Neighbor-Joining method [46]. The bootstrap consensus tree inferred from 2000 replicates is taken to represent the evolutionary history of the taxa analyzed. Branches corresponding to partitions reproduced in less than 50% bootstrap replicates are collapsed. The percentage of replicate trees in which the associated taxa clustered together in the bootstrap test (2000 replicates) are shown next to the branches. The tree is drawn to scale, with branch lengths in the same units as those of the evolutionary distances used to infer the phylogenetic tree. The evolutionary distances were computed using the JTT matrix-based method and are in the units of the number of amino acid substitutions per site [48]. The analysis involved 33 amino acid sequences. All positions containing gaps and missing data were eliminated. There were a total of 312 positions in the final dataset.

In *C*. *canephora*, eight putative RDR proteins were found after BLASTP on the Coffee Genome Hub. Conserved domain analysis confirmed the presence of the RNA-dependent RNA polymerase (RdRP) domain, and Multiple Em for Motif Elicitation (MEME) (Version 4.11.2) [49] analysis revealed that six coffee RDR proteins possess a DLDGD motif and two possess a DFDGD motif (Fig 4). Multiple alignments with orthologs sequences and phylogenetic tree analysis were also performed to assign the coffee RDR proteins and to determine the evolutionary relationship with the other angiosperm species. Four RDRs corresponded to RDR1, one to RDR2, one to RDR6, and two to RDR3 (Fig 5). The name, locus position, length, and identity of the CcRDR proteins with their respective orthologs from Arabidopsis are presented in Table 2.

In Arabidopsis, the six RDR proteins are divided into four families: RDR1, RDR2, RDR3 (RDR3a and RDR3b), and RDR6 [63]. RDR1, RDR2, and RDR6 function in the formation of dsRNA from ssRNA sequences, which are processed into several types of siRNAs targeting specific endogenous loci [64]. Among the six Arabidopsis RDR genes, AtRDR1, AtRDR2, and AtRDR6 are involved in processes such as viral resistance, chromatin silencing, and Post-Translational Gene Silencing (PTGS) [65]. The function of the RDR3 genes remains unknown, but the presence of at least one copy of the RDR3 gene in several plant genomes and other organisms suggests that these proteins may have functional significance [66].

In the phylogenetic tree, two main clades are observed, one consisting of RDR1, RDR2, and RDR6 and the other consisting of RDR3. This observation is consistent with the division of the two clades predicted based on their catalytic motifs (Fig 5). Although we found two RDR3 genes in *C*. *canephora*, similarly to tomato (SlRDR3a and SlRDR3b), the two CcRDR3 genes grouped with SlRDR3a (Fig 5).

**Fig 6:**
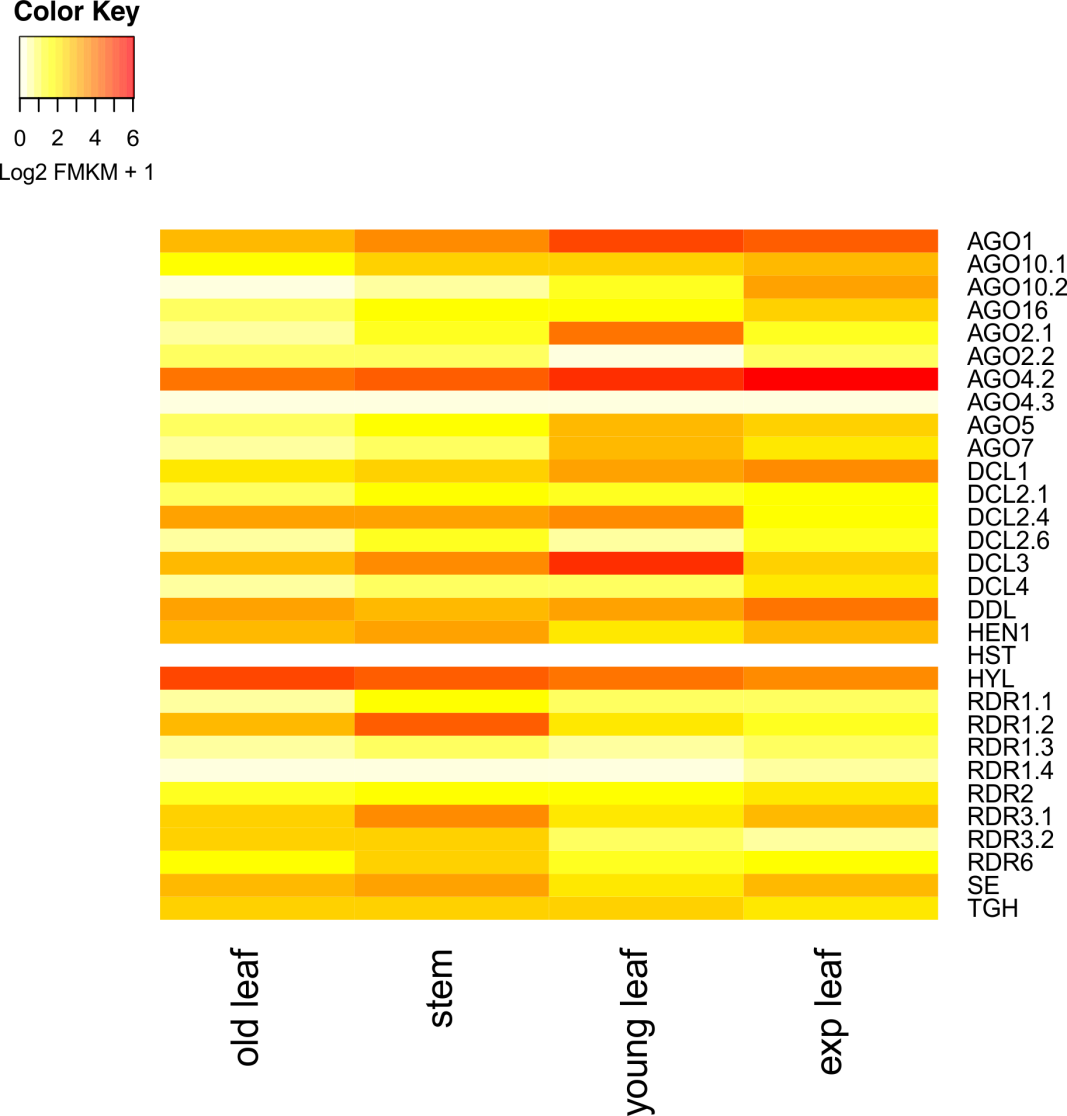
Validation of the main proteins of genes involved in the coffee RNA-guided silencing pathways from RNAseq libraries. Heatmap showing the expression pattern of the *C*. *canephora* RNA-silencing genes in three leaf developmental stages—Young, Expandend (“exp” in the figure), and Old—and Stem. (Transcriptome available at https://www.ncbi.nlm.nih.gov/sra/?term=ERP003741).

To confirm the expression of the main RNA-silencing components, we searched the RNA-seq data of *Coffea canephora* publicly available in the Sequence Read Archive (SRA) of the NCBI (https://www.ncbi.nlm.nih.gov/sra/?term=ERP003741). Sequencing data of leaves collected at different development stages (young, expanded, and old) and stem tissues were analyzed to determine the expression profile of the sRNA silencing components identified in coffee, including *CcAGO*, *CcDCL*, *CcRDR*, *CcHYL1*, *CcSE*, *CcDDL*, *CcTG*, *CcHEN1*, and *CcHST*. The heatmap showed expression in all the tested tissues (Fig 6). However, Cufflinks analysis assigned three loci annotated as DCL2 in the coffee genome (Cc02_g14900, Cc02_g14910, and Cc02_g14920 –herein referred to as DCL2.2 and DCL2.3) as isoforms of the same genetic locus; therefore, these were not included in the heatmap (S3 Fig). Furthermore, CcAGO4.1 was not expressed in any of the tissues.

### miRNAs and miRNA target prediction

Homology-based miRNA search was conducted by comparing plant miRNAs deposited in the miRBase database version 21 against the coffee genome. After applying filters to retrieve miRNA precursors, a total of 235 precursors and 317 mature miRNAs were identified and characterized, belonging to 113 MIR families (S2 Table). The mature miRNAs were found in both the 3' and 5’ arms of the precursor, with sizes ranging from 19 to 25 nt, most of which were 21 nt (S2 Table). The preferred first 5’ nucleotide was Uracil (U). The location of the pre-miRNAs in the genome was determined, including the chromosome, start and end point, strand position, and genic/intergenic position (S2 Table). MIR genes were observed in all chromosomes, and chromosome 2 contained the highest number of MIR genes (36 genes). A total of 38 precursors were found either in antiparallel clusters or clustered with a maximum distance of 10 kb between the two miRNAs, but most were widespread throughout the chromosomes. A total of 193 precursors were identified in the intergenic regions, and the other 43 precursors were found within genes (S2 Table).

The precursor sizes varied from 68 to 338 nt, and the AU (Adenine+Uracil) content ranged from 41% to 69% (S3 Table). The thermodynamic aspects of the precursors—Minimal Free Energy (MFE), adjusted MFE (AMFE), MFE index (MFEI), Minimal Free Energy of the thermodynamic ensemble (MFEE), Ensemble Diversity (Diversity) and frequency of the MFE structure in the ensemble (Frequency)—were measured (S3 Table). The MFE ranged from -21.9 to -97.5 kcal mol^-1^, with a mean of -56.4 kcal mol^-1^; the AMFE ranged from -21.4 to -59.6 kcal mol^-1^, with a mean of -36.46 kcal mol^-1^; and the MFEI varied from 0.7 to 1.7, with a mean of 0.88.

**Fig 7:**
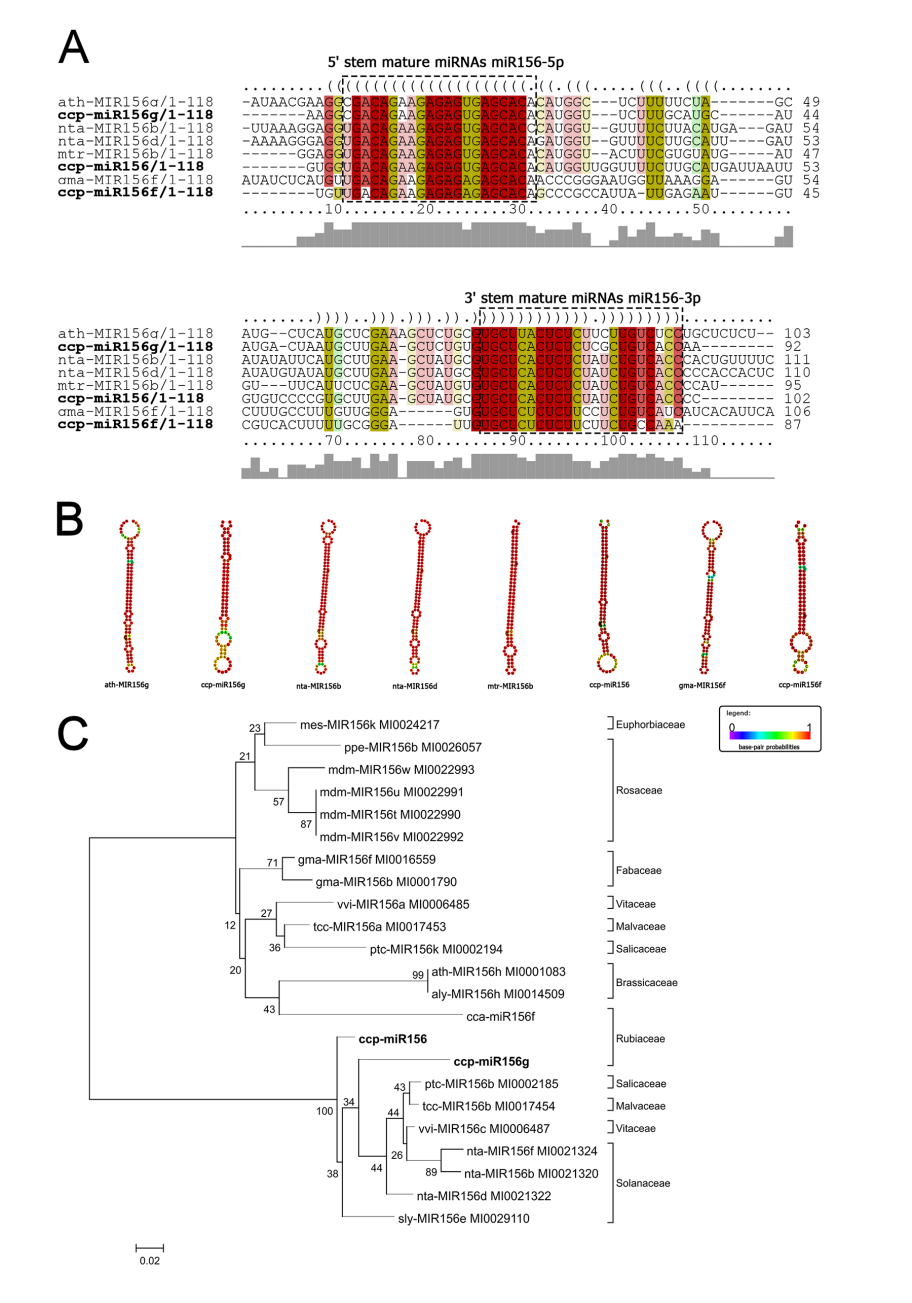
**Alignment of pre-miRNA sequences (a), comparison of secondary structures (b) and phylogenetic tree (c) of ccp-MIR156 miRNAs and their orthologues.** ccp- *Coffea canephora*, ath–*Arabidopsis thaliana*, nta–*Nicotiana tabacum*, mtr–*Medicago truncatula*, gma–*Glycine max*, mes–*Manihot esculenta*, ppe–*Prunus persica*, mdm–*Malus domestica*, vvi–*Vitis vinifera*, tcc—*Theobroma cacao*, ptc–*Populus trichocarpa*, aly–*Arabidopsis lyrata*, sly–*Solanum lycopersicum*. The evolutionary history was inferred using the Neighbor-Joining method[46]. The bootstrap consensus tree inferred from 5000 replicates is taken to represent the evolutionary history of the taxa analyzed. Branches corresponding to partitions reproduced in less than 50% bootstrap replicates are collapsed. The percentage of replicate trees in which the associated taxa clustered together in the bootstrap test (5000 replicates) are shown next to the branches. The tree is drawn to scale, with branch lengths in the same units as those of the evolutionary distances used to infer the phylogenetic tree. The evolutionary distances were computed using the Kimura 2-parameter method [3] and are in the units of the number of base substitutions per site[47]. The analysis involved 23 nucleotide sequences. All positions containing gaps and missing data were eliminated. There were a total of 68 positions in the final dataset.

**Fig 8:**
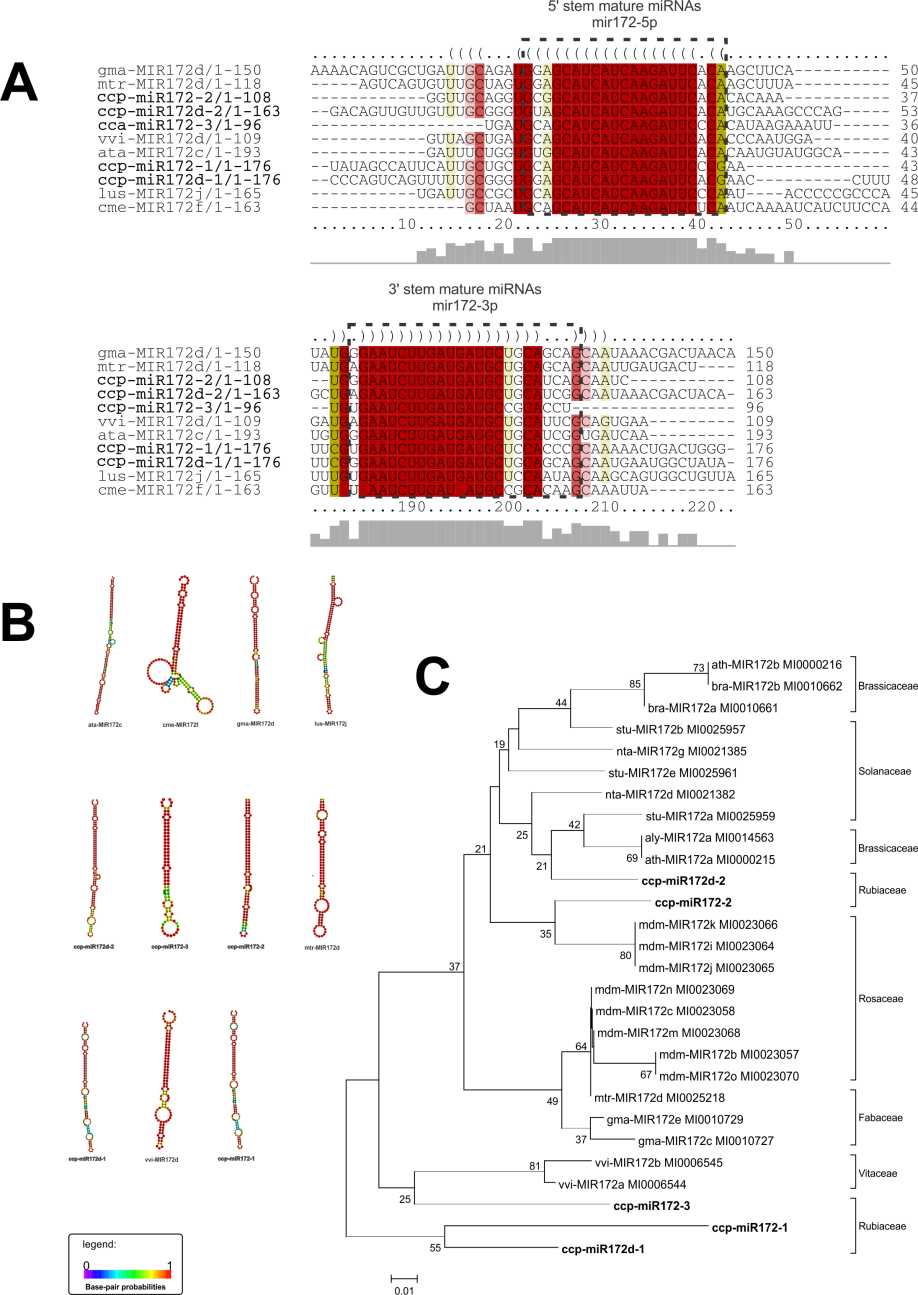
**Alignment of pre-miRNA sequences (a), comparison of secondary structures (b) and phylogenetic tree (c) of ccp-MIR172 miRNAs and their orthologues.** ccp- *Coffea canephora*, ath–*Arabidopsis thaliana*, cme—*Cucumis melo*, gma–*Glycine max*, lus—*Linum usitatissimum*, mtr–*Medicago truncatula*, vvi–*Vitis vinifera*, bra–*Brassica rapa*, stu–*Solanum tuberosum*, nta–*Nicotiana tabacum*, aly–*Arabidopsis lyrata*, mdm–*Malus domestica*. The evolutionary history was inferred using the Neighbor-Joining method[46]. The bootstrap consensus tree inferred from 5000 replicates is taken to represent the evolutionary history of the taxa analyzed. Branches corresponding to partitions reproduced in less than 50% bootstrap replicates are collapsed. The percentage of replicate trees in which the associated taxa clustered together in the bootstrap test (5000 replicates) are shown next to the branches. The tree is drawn to scale, with branch lengths in the same units as those of the evolutionary distances used to infer the phylogenetic tree. The evolutionary distances were computed using the Kimura 2-parameter method and are in the units of the number of base substitutions per site[47]. The analysis involved 28 nucleotide sequences. All positions containing gaps and missing data were eliminated. There were a total of 46 positions in the final dataset.

**Fig 9:**
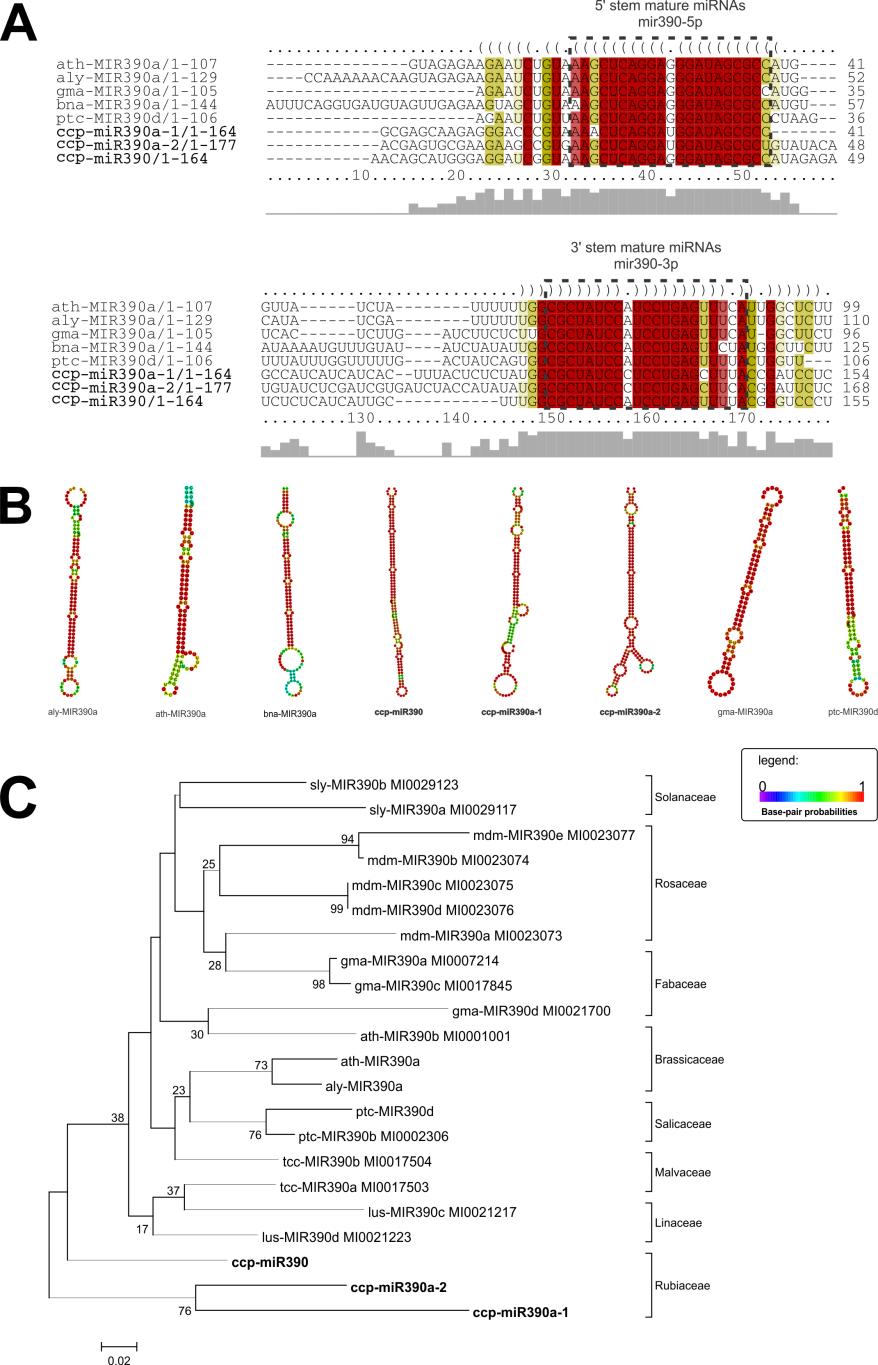
**Alignment of pre-miRNA sequences (a), comparison of secondary structures (b) and phylogenetic tree (c) of ccp-MIR390 miRNAs and their orthologues.** ccp- *Coffea canephora*, aly–*Arabidopsis lyrata*, ath–*Arabidopsis thaliana*, bna—Brassica napus, gma–*Glycine max*, ptc–*Populus trichocarpa*, sly–*Solanum lycopersicum*, mdm–*Malus domestica*, tcc*—Theobroma cacao*, lus—*Linum usitatissimum*. The evolutionary history was inferred using the Neighbor-Joining method[46]. The optimal tree with the sum of branch length = 1.87754489 is shown. The percentage of replicate trees in which the associated taxa clustered together in the bootstrap test (5000 replicates) are shown next to the branches. The tree is drawn to scale, with branch lengths in the same units as those of the evolutionary distances used to infer the phylogenetic tree. The evolutionary distances were computed using the Kimura 2-parameter method and are in the units of the number of base substitutions per site[47]. The analysis involved 22 nucleotide sequences. All positions containing gaps and missing data were eliminated. There were a total of 65 positions in the final dataset.

We chose some of the highly conserved MIR families–*MIR156*, *MIR172*, and *MIR390* –for further characterization. We analyzed the conservation of their sequences and structure as well as their phylogenetic distributions. For each of these MIR families, multiple sequence alignment and secondary structure prediction were performed to verify the primary and secondary conservation relative to other plant species orthologs (Figs 7–9). These MIR families presented high conservation between their primary and secondary structures and their orthologs (Figs 7–9). A phylogenetic tree was created to verify the evolutionary distribution of each MIR family (Figs 7–9).

We also identified potential miRNA target genes using psRNATarget [67] based on the *C*. *canephora* genome. In total, 2239 genes were identified as potential targets of the miRNAs, many of which were targeted by more than one miRNA (S4 Table).

**Fig 10:**
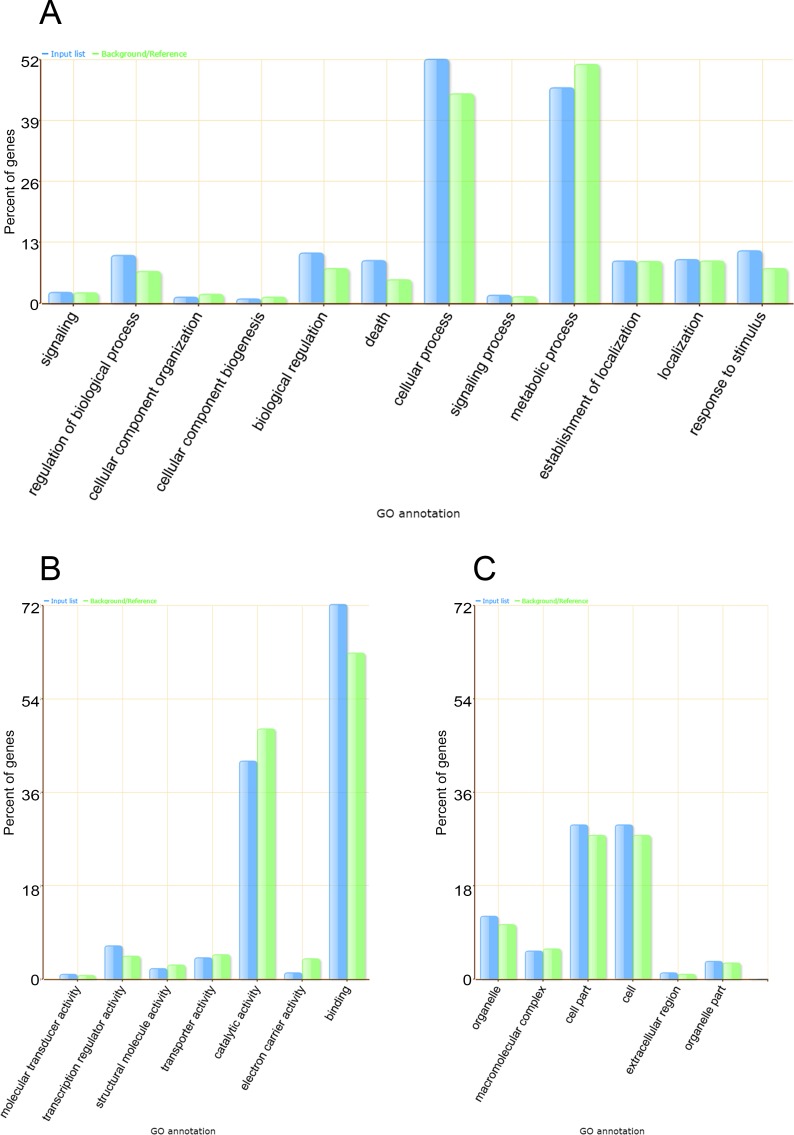
SEA (Singular Enrichment Analysis) of the GO terms of the predicted targets of the ccp-miRNAs. (A) Biological process, (B) molecular function and (C) cellular component.

To classify and group the Gene Ontology (GO) classes of the miRNA targets, the web tool SEA (Singular Enrichment Analysis) from agriGO (http://bioinfo.cau.edu.cn/agriGO/index.php) was used [53]. A total of 1356 GO terms were annotated for the target genes in *C*. *canephora*, and these were summarized in 57 main terms. The genes belonging to the 25 overrepresented terms among the three GO categories, namely the biological process (Fig 10A), molecular function (Fig 10B), and cellular component (Fig 10C) categories, are presented.

We further identified the putative targets of ccp-MIR156, ccp-MIR172, and ccp-MIR390 in the RNA-seq libraries of stem and leaf tissues. The complete list of the targets assigned to these miRNAs is presented in S5 Table.

## Discussion

### Duplication events and domain and catalytic site configurations reveal insights into the sRNA pathway core members in *C*. *canephora*

Duplication of DCL2 has been observed in several species [56, 68, 69]. The largest of the six CcDCL2 members, CcDCL2.1, is located on chromosome 9 and is missing its DsRB (DSRM) domain. DCL2 usually contains only one DsRB (DSRM) domain, but in the four tomato DCL2s, only one member (SlDCL2d) possesses a DsRB (DSRM) domain [55]. The shortest CcDCL2 identified, CcDCL2.5 (354 aa), is located on chromosome 6, along with CcDCL2.6 (762 aa). Both of these proteins are truncated. Similar findings were observed for CcDCL2.2, CcDCL2.3, and CcDCL2.4, which are located sequentially on chromosome 2 and are also incomplete according to the current version of the genome annotation.

Expression analyses demonstrated that at least four DCL2-like genes are active in coffee (Fig 6 and S3 Fig), including the only complete sequence, CcDCL2.1. The other two DCL2 genes that are expressed are DCL2.4 (Cc02_g14930) and DCL2.6 (Cc06_g19980) (Fig 6). In addition to that, a total of seven isoforms were assigned to the same locus (Cc02_g14900) (S3 Fig). This might indicate misannotation of the three DCL2 assigned to the sequential loci at Chromosome 2 (Cc02_g14900, Cc02_g14910 and Cc02_g14920), which are probably exons of a unique gene. Finally, DCL2.5 (Cc06_g19770), which is the most incomplete DCL2 annotated in the genome, is not expressed in either tissue and could not be confirmed. Although it remains unclear how many DCL-like proteins are present and where on the genome their complete sequence can be found, an expansion of the DCL-like proteins appears to have occurred in *C*. *canephora* through the duplication of the DCL2-like family.

DCL-like proteins might contain the characteristic catalytic residues of RNase III domain-containing proteins [59]. The RNase III domains bind dsRNA and are responsible for cleavage and processing; therefore, they are essential to sRNA generation [58]. Only the incomplete CcDCL2 (CcDCL2.2-CcDCL2.6) proteins did not present the conserved residues (EDDE—Glu-Asp-Asp- Glu) in one or both RNAse III domains, reinforcing the need for further investigation into these short CcDCL2-like proteins.

The presence of CcAGO10, CcAGO2, and CcAGO4 paralogs indicates the occurrence of duplication events in the *C*. *canephora* genome. Gene duplication is one possible reason for the expansion of AGO proteins. The expansion of the AGO family in flowering plants suggests functional diversification of the AGO proteins [61].

PIWI domains contain the three conserved metal-chelating residue motif aspartate, aspartate, histidine (DDH). The DDH motif functions as a catalytic triad. A conserved histidine found at position 798 of AtAGO1 is also important for the catalytic function of the AGO proteins [62]. The four CcAGO proteins that possess the DDH/H motif (CcAGO1, CcAGO5, CcAGO7, and CcAGO10.1) potentially act as the slicer of RISC (Table 5). CcAGO2.1 and CcAGO2.2 showed a third aspartate residue instead of histidine, which was also observed in SlAGO2 [55], AtAGO2 and AtAGO3 [56]; GmAGO3a and SbAGO2 [34]; and OsAGO2 and OsAGO3 [56]. The absence of catalytic amino acids could prevent the processing of target RNA by cleavage; therefore, accessory factors for mediating mRNA turnover could be required [56]. However, the presence of a third aspartate in the triad restores the catalytic activity to function as slicer components of the silencing effector complexes in Arabidopsis and rice AGO2 and AGO3 [56].

In another four CcAGOs (CcAGO4.2, CcAGO4.3, CcAGO10.2, and CcAGO16), the conserved H798 residue has been replaced (Table 5). Previous studies showed variability in the H798 residue in monocots [54, 56], while in tomato (*S*. *lycopersicum*), the H798 sites in the AGO4 group (SlAGO4a, b, c, d and SlAGO6) were replaced by proline [55]. In *C*. *canephora*, which is closely related to *Solanaceae*, the H798 residue was also replaced in the AGO4 members, but in CcAGO10.2 and CcAGO16, the H798 residue was replaced by glutamine and serine, respectively.

CcAGO4.1 presented neither of the residues required for catalytic activity, which could represent either functionalization or loss of function. *CcAGO4*.*1* expression was not found in the RNA-seq libraries, corroborating the hypothesis that this protein is not active due to a lack of effective catalytic residues. However, AGO4 proteins can function either dependent on or independent of their catalytic activity [70]. The expression of *CcAGO4*.*2* and *CcAGO4*.*3* indicates that Transcriptional Gene Silencing (TGS) guided by RNA is upregulated in coffee because AGO4 has been implicated in RNA-Directed DNA Methylation (RdDM) [71].

In the RDR-like proteins, the RdRP domain contains a DxDGD catalytic motif [72]. RDR1, RDR2, and RDR6 (RDRα clade) share a DLDGD catalytic motif, whereas RDR3 (RDRγ clade) possesses a DFDGD motif [63, 72]. The putative catalytic domains of the CcRDRs presented with the respective expected motifs of the α (CcRDR1.1–1.4, CcRDR2, and CcRDR6) and γ (CcRDR3.1 and CcRDR3.2) clades (Fig 4). Additionally, the RDRα clade displays a conserved subsequence (C/A)SG(S/G) upstream of the DLDGD motif [72], and all CcRDR1s and CcRDR2 present the CSGS sequence, whereas CcRDR6 possessed an ASGS sequence.

Interestingly, four RDR1 genes were found in *C*. *canephora*, all of which were located sequentially on chromosome 11 (Table 2). RDR1 is involved in plant defenses against biotic and abiotic components [17, 73]. Most enriched GO terms in *C*. *canephora* belong to the defense response class [39]. It was also observed that the *C*. *canephora* genome includes several species-specific gene family expansions, including the defense-related genes [39]; this could also be the case for the RDR1 genes.

### The *C*. *canephora* genome possesses several conserved and non-conserved *MIR* loci that target major cellular processes

Using a robust pipeline, we were able to significantly enrich the number of predicted miRNAs annotated in *Coffea spp* [35–39]. We identified 235 precursors and 317 mature sequences, whereas previous analyses of the coffee genome identified only 92 precursors [39]. The precursors belonged to 113 MIR families, representing a considerable increase relative to the 33 families originally described in the coffee genome report [39]. Our stringent and robust pipeline predicted sequences that were real miRNA precursors and identified more paralogous loci for several families already described.

The major MIR family was MIR171, with a total of 15 pre-miRNAs. Many highly conserved MIR families among plants were identified, including MIR171, MIR172, MIR156, MIR159, MIR160, MIR164, MIR167, MIR169, MIR390, and several others [74]. In contrast, some of the precursors identified belong to MIR families annotated for one species in miRBase v.21, such as ptc-MIR6476a (*Populus trichocarpa*) and stu-MIR8001b (*Solanum tuberosum*) [75, 76].

Some of the most conserved families in plants, MIR156, MIR172, and MIR390 [43], have been identified in several species [33, 43, 75–77] and play central roles in plant development and stress responses. For instance, miR156 targets SQUAMOSA PROMOTER BINDING PROTEIN-LIKE (SPL) transcription factor family members, and miR156-SPL networks define an essential regulatory module that controls phase transitions, leaf trichome development, male fertility, embryonic patterning, and anthocyanin biosynthesis [78–82]. In the *C*. *canephora* genome, miR156 has 24 putative targets (S4 Table). Based on the transcriptomes of the stem and leaf tissue samples, we found that miR156 potentially targets SPL-6 and SPL-12 in both tissues (S5 Table). In total, 15 putative targets were identified in the stems and leaves, some of which were identified either in both tissues or in only one (S5 Table).

The MIR172 family consists of five precursors and ten mature miRNAs (S2 Table). This highly conserved family is found in several species and is related to the regulation of flowering time and floral organ identity by targeting APETALA2-like transcription factors in Arabidopsis [83, 84]. miR172 acts downstream of miR156 to regulate phase transition [84], as an increase in miR156 levels corresponds to lower expression of miR172 and vice versa in several species [84–87]. In the *C*. *canephora* genome, 118 putative targets for miR172 were identified (S4 Table). Based on the transcriptome data, a total of 66 putative targets were identified, including AP2 in stem tissue (S5 Table).

miR390 is involved in the regulation of development and the response to several stresses [88–91]. Among its targets, miR390 regulates the Auxin Response Factor (ARF) by mediating non-protein coding Trans-Acting siRNA locus 3 (TAS3) generation in an AGO7-dependent manner [92]. miR390 also targets Leucine-Rich Repeat Receptor-like kinases (LRK) and regulates a LRK protein in *Oryza sativa* in response to cadmium stress [91]. In the *C*. *canephora* genome, 11 putative targets were identified (S4 Table). Four putative targets were identified in the transcriptomes of stems and leaves (S5 Table), among which a LRK (RKF1) was identified in both tissues (S5 Table).

The ccp-MIR156, ccp-MIR172, and ccp-MIR390 members were highly conserved in their primary and secondary structures relative to their respective orthologs from other species and relative to their distributions within the phylogenetic tree in a clade of Eudicotyledons, consistent with plant phylogeny (Figs 7–9) [93].

The GO terms of the putative *C*. *canephora* miRNA targets were categorized and compared with the GO terms of the whole genome as background (Fig 10). In total, 1356 GO terms were assigned to the putative targets, including a total of 14975 GO terms annotated to the genome. The main overrepresented subcategories belonging to the ‘Biological Process’ category were ‘cellular process’ and ‘metabolic process’. In the ‘Cellular Component’ category, the main overrepresented terms were ‘cell part’ and ‘cell’. In the ‘Molecular Function’ category, the main overrepresented terms were ‘catalytic activity’ and ‘binding’. Interestingly, the main categories of the potential targets were also the main categories annotated for the genome (green bars–Fig 10). Therefore, one can infer that miRNAs in *C*. *canephora* target major cellular processes.

Considering the importance of this pioneering work, we elucidated several aspects of sRNAs in *C*. *canephora*, which offers a significant step towards a better understanding of the transcriptional and post-transcriptional regulation of this major crop. An understanding of the sRNA pathways in coffee provides insights for plant breeding through genetic engineering technology.

## Supporting information

S1 Fig Alignment of a DCL1 identified in our analysis in RNAseq libraries with the *C*. *canephora* genome in the Genome Browser on the Coffee Genome Hub (coffee-genome.org). The alignment demonstrates that the DCL1 gene is present in the genome assembly, but it was not previously annotated as a protein-coding gene.
(TIF)

S2 Fig Multiple alignment of the CcAGO proteins for analysis of conservation of the active site amino acids in the conserved PIWI domain (PF02171). Aminoacids corresponding to the Aspartate-Aspartate-Histidine (DDH) motif at the positions 760, 845, and 986,and an extra Histidine at the position 798 of the AtAGO1 (DDH/H798) [62] are highlighted. Four proteins (CcAGO1, CcAGO5, CcAGO7, and CcAGO10.1) showed the conserved DDH/H798 residues. In four CcAGOs, the DDH catalytic motif was conserved, but the H798 was replaced by a serine (CcAGO16), proline (CcAGO4.2 and CcAGO4.3) or glutamine (CcAGO10.2). Two CcAGO proteins possessed an aspartate residue in place of the third histidine of the DDH motif (CcAGO2.1 and CcAGO2.2). The CcAGO4.1 contains neither the catalytic DDH motif nor the H798 residue.
(TIF)

S3 Fig Expression profile of the 7 isoforms of CcDCL2 assigned to the same locus in the Chromosome 2 (Cc02_g14900) identified in the RNAseq libraries. It was analyzed the CcDCL2 expression in three developmental stages of *C*. *canephora* leaf—young, expanded (exp in the figure) and old—and stem (Available at https://www.ncbi.nlm.nih.gov/sra/?term=ERP003741). FPKM stands for Fragments Per Kilobase Million.
(TIF)

S1 Table Proteins associated with the sRNA pathways in the *Coffea canephora* genome. Protein name, literature reference of the first description in plants, the *C*. *canephora* ortholog, locus name and position, and respective protein length.
(DOCX)

S2 Table Identification of pre-miRNAs in *Coffea canephora*. Precursor names, chromosome numbers, start and end positions, strand and genic/intergenic locations, mature 5p and/or 3p miRNAs, start and end positions in the precursor, and mature miRNA sizes.
(DOCX)

S3 Table Structural characteristics and thermodynamic aspects of the precursors of the pre-miRNA of *Coffea canephora*. Minimal Free Energy (MFE), adjusted MFE (AMFE), MFE index (MFEI), Minimal Free Energy of the thermodynamic ensemble (MFEE), Ensemble Diversity (Diversity), and frequency of the MFE structure in the ensemble (Frequency).
(DOCX)

S4 Table Target prediction of the mature miRNAs in the *C*. *canephora* genome with psRNATarget. miRNA names, Target ID (Locus Name) in *C*. *canephora*, Expectation scoring, unpaired energy (UPE) required to open the secondary structure around the miRNA target site, the start and end position on the miRNA and the Target, the sequence alignment of the miRNA and Target sequences, and the type of inhibition method.
(DOCX)

S5 Table The putative targets of ccp-MIR156, ccp-MIR172, and ccp-MIR390 in the RNA-seq libraries of the stem and leaf tissues of *Coffea canephora*. miRNA in the respective tissue, target description, and number of associated GO terms.
(DOCX)

## References

[1] Brodersen P, Voinnet O. The diversity of RNA silencing pathways in plants. Trends in Genetics. 2006;22(5):268–80. doi: 10.1016/j.tig.2006.03.003 16567016 10.1016/j.tig.2006.03.003

[2] Axtell MJ. Classification and comparison of small RNAs from plants. Annu Rev Plant Biol. 2013;64(1):137–59. 23330790 10.1146/annurev-arplant-050312-120043

[3] Chen X. Small RNAs and Their Roles in Plant Development. Annual Review of Cell and Developmental Biology. 2009;25(1):21–44. 10.1146/annurev.cellbio.042308.113417 PMC5135726 19575669

[4] Borges F, Martienssen RA. The expanding world of small RNAs in plants. Nat Rev Mol Cell Biol. 2015;16(12):727–41. Epub 2015/11/05. PubMed Central PMCID: PMCPmc4948178. doi: 10.1038/nrm4085 26530390 10.1038/nrm4085 PMC4948178

[5] Kim YJ, Zheng B, Yu Y, Won SY, Mo B, Chen X. The role of Mediator in small and long noncoding RNA production in Arabidopsis thaliana. The EMBO journal. 2011;30(5):814–22. Epub 2011/01/22. PubMed Central PMCID: PMCPmc3049218. doi: 10.1038/emboj.2011.3 21252857 10.1038/emboj.2011.3 PMC3049218

[6] Tang G. Plant microRNAs: an insight into their gene structures and evolution. Semin Cell Dev Biol. 2010;21(8):782–9. doi: 10.1016/j.semcdb.2010.07.009 20691276 10.1016/j.semcdb.2010.07.009

[7] Yu B, Bi L, Zheng B, Ji L, Chevalier D, Agarwal M, et al The FHA domain proteins DAWDLE in Arabidopsis and SNIP1 in humans act in small RNA biogenesis. Proc Natl Acad Sci U S A. 2008;105(29):10073–8. PubMed Central PMCID: PMC2481372. doi: 10.1073/pnas.0804218105 18632581 10.1073/pnas.0804218105 PMC2481372

[8] Kurihara Y, Takashi Y, Watanabe Y. The interaction between DCL1 and HYL1 is important for efficient and precise processing of pri-miRNA in plant microRNA biogenesis. RNA (New York, NY). 2006;12(2):206–12. Epub 2006/01/24. PubMed Central PMCID: PMCPmc1370900. 10.1261/rna.2146906 PMC1370900 16428603

[9] Dong Z, Han MH, Fedoroff N. The RNA-binding proteins HYL1 and SE promote accurate in vitro processing of pri-miRNA by DCL1. Proc Natl Acad Sci U S A. 2008;105(29):9970–5. PubMed Central PMCID: PMC2481344. doi: 10.1073/pnas.0803356105 18632569 10.1073/pnas.0803356105 PMC2481344

[10] Lobbes D, Rallapalli G, Schmidt DD, Martin C, Clarke J. SERRATE: a new player on the plant microRNA scene. EMBO reports. 2006;7(10):1052–8. Epub 2006/09/16. PubMed Central PMCID: PMCPmc1618363. doi: 10.1038/sj.embor.7400806 16977334 10.1038/sj.embor.7400806 PMC1618363

[11] Ren G, Xie M, Dou Y, Zhang S, Zhang C, Yu B. Regulation of miRNA abundance by RNA binding protein TOUGH in Arabidopsis. Proc Natl Acad Sci U S A. 2012;109(31):12817–21. Epub 2012/07/18. PubMed Central PMCID: PMCPmc3412041. doi: 10.1073/pnas.1204915109 22802657 10.1073/pnas.1204915109 PMC3412041

[12] Rogers K, Chen X. Biogenesis, Turnover, and Mode of Action of Plant MicroRNAs. Plant Cell. 2013;25(7):2383–99. doi: 10.1105/tpc.113.113159 23881412 10.1105/tpc.113.113159 PMC3753372

[13] Yamaguchi A, Abe M. Regulation of reproductive development by non-coding RNA in Arabidopsis: to flower or not to flower. J Plant Res. 2012;125(6):693–704. PubMed Central PMCID: PMC3485539. doi: 10.1007/s10265-012-0513-7 22836383 10.1007/s10265-012-0513-7 PMC3485539

[14] Hu W, Wang T, Xu J, Li H. MicroRNA mediates DNA methylation of target genes. Biochemical and Biophysical Research Communications. 2014;444(4):676–81. doi: 10.1016/j.bbrc.2014.01.171 24508262 10.1016/j.bbrc.2014.01.171

[15] Li J, Yang Z, Yu B, Liu J, Chen X. Methylation protects miRNAs and siRNAs from a 3'-end uridylation activity in Arabidopsis. Current biology: CB. 2005;15(16):1501–7. Epub 2005/08/23. doi: 10.1016/j.cub.2005.07.029 16111943 10.1016/j.cub.2005.07.029 PMC5127709

[16] Zeng Y, Cullen BR. Structural requirements for pre-microRNA binding and nuclear export by Exportin 5. Nucleic acids research. 2004;32(16):4776–85. Epub 2004/09/10. PubMed Central PMCID: PMCPmc519115. doi: 10.1093/nar/gkh824 15356295 10.1093/nar/gkh824 PMC519115

[17] Bologna NG, Voinnet O. The Diversity, Biogenesis, and Activities of Endogenous Silencing Small RNAs in Arabidopsis. Annual Review of Plant Biology. 2014;65(1):473–503. 10.1146/annurev-arplant-050213-035728 24579988

[18] Liu J, Valencia-Sanchez MA, Hannon GJ, Parker R. MicroRNA-dependent localization of targeted mRNAs to mammalian P-bodies. Nature cell biology. 2005;7(7):719–23. Epub 2005/06/07. PubMed Central PMCID: PMCPmc1855297. doi: 10.1038/ncb1274 15937477 10.1038/ncb1274 PMC1855297

[19] Matzke MA, Mosher RA. RNA-directed DNA methylation: an epigenetic pathway of increasing complexity. Nat Rev Genet. 2014;15(6):394–408. Epub 2014/05/09. doi: 10.1038/nrg3683 24805120 10.1038/nrg3683

[20] Schiebel W, Haas B, Marinkovic S, Klanner A, Sanger HL. RNA-directed RNA polymerase from tomato leaves. II. Catalytic in vitro properties. The Journal of biological chemistry. 1993;268(16):11858–67. 7685023

[21] Moissiard G, Parizotto EA, Himber C, Voinnet O. Transitivity in Arabidopsis can be primed, requires the redundant action of the antiviral Dicer-like 4 and Dicer-like 2, and is compromised by viral-encoded suppressor proteins. RNA (New York, NY). 2007;13(8):1268–78. Epub 2007/06/27. PubMed Central PMCID: PMCPmc1924903. 10.1261/rna.541307 PMC1924903 17592042

[22] Cao X, Jacobsen SE. Role of the arabidopsis DRM methyltransferases in de novo DNA methylation and gene silencing. 2002;(0960–9822 (Print)). 10.1016/s0960-9822(02)00925-9 12121623

[23] Law JA, Jacobsen SE. Establishing, maintaining and modifying DNA methylation patterns in plants and animals. Nat Rev Genet. 2010;11(3):204–20. doi: 10.1038/nrg2719 20142834 10.1038/nrg2719 PMC3034103

[24] Onodera Y, Haag JR, Ream T, Costa Nunes P, Pontes O, Pikaard CS. Plant nuclear RNA polymerase IV mediates siRNA and DNA methylation-dependent heterochromatin formation. Cell. 2005;120(5):613–22. doi: 10.1016/j.cell.2005.02.007 15766525 10.1016/j.cell.2005.02.007

[25] Wierzbicki AT, Cocklin R, Mayampurath A, Lister R, Rowley MJ, Gregory BD, et al Spatial and functional relationships among Pol V-associated loci, Pol IV-dependent siRNAs, and cytosine methylation in the Arabidopsis epigenome. Genes & Development. 2012;26(16):1825–36. 22855789 10.1101/gad.197772.112 PMC3426761

[26] Zhou M, Law JA. RNA Pol IV and V in gene silencing: Rebel polymerases evolving away from Pol II's rules. Current opinion in plant biology. 2015;27:154–64. doi: 10.1016/j.pbi.2015.07.005 26344361 10.1016/j.pbi.2015.07.005 PMC4618083

[27] Wierzbicki AT, Ream TS, Haag JR, Pikaard CS. RNA polymerase V transcription guides ARGONAUTE4 to chromatin. Nature genetics. 2009;41(5):630–4. PubMed Central PMCID: PMC2674513. doi: 10.1038/ng.365 19377477 10.1038/ng.365 PMC2674513

[28] Matzke MA, Kanno T, Matzke AJM. RNA-Directed DNA Methylation: The Evolution of a Complex Epigenetic Pathway in Flowering Plants. Annual Review of Plant Biology. 2015;66(1):243–67. 10.1146/annurev-arplant-043014-114633 25494460

[29] Speth C, Willing EM, Rausch S, Schneeberger K, Laubinger S. RACK1 scaffold proteins influence miRNA abundance in Arabidopsis. The Plant journal: for cell and molecular biology. 2013;76(3):433–45. 23941160 10.1111/tpj.12308

[30] Jeong IS, Aksoy E, Fukudome A, Akhter S, Hiraguri A, Fukuhara T, et al Arabidopsis C-terminal domain phosphatase-like 1 functions in miRNA accumulation and DNA methylation. PLoS One. 2013;8(9):e74739 PubMed Central PMCID: PMC3776750. doi: 10.1371/journal.pone.0074739 24058624 10.1371/journal.pone.0074739 PMC3776750

[31] Karlsson P, Christie MD, Seymour DK, Wang H, Wang X, Hagmann J, et al KH domain protein RCF3 is a tissue-biased regulator of the plant miRNA biogenesis cofactor HYL1. Proceedings of the National Academy of Sciences. 2015;112(45):14096–101. 10.1073/pnas.1512865112 PMC4653147 26512101

[32] Kapoor M, Arora R, Lama T, Nijhawan A, Khurana J, Tyagi A, et al Genome-wide identification, organization and phylogenetic analysis of Dicer-like, Argonaute and RNA-dependent RNA Polymerase gene families and their expression analysis during reproductive development and stress in rice. BMC Genomics. 2008;9(1):451. 18826656 10.1186/1471-2164-9-451 PMC2576257

[33] de Sousa Cardoso TC, Portilho LG, de Oliveira CL, McKeown PC, Maluf WR, Gomes LA, et al Genome-wide identification and in silico characterisation of microRNAs, their targets and processing pathway genes in Phaseolus vulgaris L. Plant Biol 2016;18(2):206–19. doi: 10.1111/plb.12377 26250338 10.1111/plb.12377

[34] Liu X, Lu T, Dou Y, Yu B, Zhang C. Identification of RNA silencing components in soybean and sorghum. BMC Bioinformatics. 2014;15(1):4. 24387046 10.1186/1471-2105-15-4 PMC3882329

[35] Loss-Morais G, Ferreira DCR, Margis R, Alves-Ferreira M, Corrêa RL. Identification of novel and conserved microRNAs in Coffea canephora and Coffea arabica. Genetics and Molecular Biology. 2014;37(4):671–82. doi: 10.1590/S1415-47572014005000020 25505842 10.1590/S1415-47572014005000020 PMC4261967

[36] Rebijith KB, Asokan R, Ranjitha HH, Krishna V, Nirmalbabu K. In silico mining of novel microRNAs from coffee (Coffea arabica) using expressed sequence tags. Journal of Horticultural Science and Biotechnology 2013;88(3):325–37.

[37] Akter A, Islam MM, Mondal SI, Mahmud Z, Jewel NA, Ferdous S, et al Computational identification of miRNA and targets from expressed sequence tags of coffee (Coffea arabica). Saudi Journal of Biological Sciences. 2014;21(1):3–12. doi: 10.1016/j.sjbs.2013.04.007 24596494 10.1016/j.sjbs.2013.04.007 PMC3937464

[38] Chaves SS, Fernandes-Brum CN, Silva GF, Ferrara-Barbosa BC, Paiva LV, Nogueira FT, et al New Insights on Coffea miRNAs: Features and Evolutionary Conservation. Appl Biochem Biotechnol. 2015;177(4):879–908. doi: 10.1007/s12010-015-1785-x 26277190 10.1007/s12010-015-1785-x

[39] Denoeud F, Carretero-Paulet L, Dereeper A, Droc G, Guyot R, Pietrella M, et al The coffee genome provides insight into the convergent evolution of caffeine biosynthesis. Science. 2014;345(6201):1181–4. doi: 10.1126/science.1255274 25190796 10.1126/science.1255274

[40] de Souza Gomes M, Muniyappa MK, Carvalho SG, Guerra-Sa R, Spillane C. Genome-wide identification of novel microRNAs and their target genes in the human parasite Schistosoma mansoni. Genomics. 2011;98(2):96–111. Epub 2011/06/07. doi: 10.1016/j.ygeno.2011.05.007 21640815 10.1016/j.ygeno.2011.05.007

[41] Smit AFA, Hubley R, Green P. RepeatMasker at http://repeatmasker.org. Accessed 20 January 2016.

[42] Gardner PP, Daub J, Tate JG, Nawrocki EP, Kolbe DL, Lindgreen S, et al. Rfam: updates to the RNA families database. 2009;(1362–4962 (Electronic)). D—NLM: PMC2686503 EDAT- 2008/10/28 09:00 MHDA- 2009/03/04 09:00 CRDT- 2008/10/28 09:00 PHST- 2008/10/25 [aheadofprint] AID—gkn766 [pii] AID. 10.1093/nar/gkn766 PMC2686503 18953034

[43] Zhang B, Pan X, Cannon CH, Cobb GP, Anderson TA. Conservation and divergence of plant microRNA genes. The Plant journal: for cell and molecular biology. 2006;46(2):243–59. Epub 2006/04/21. 16623887 10.1111/j.1365-313X.2006.02697.x

[44] Zhang BH, Pan XP, Cox SB, Cobb GP, Anderson TA. Evidence that miRNAs are different from other RNAs. Cell Mol Life Sci. 2006;63(2):246–54. doi: 10.1007/s00018-005-5467-7 16395542 10.1007/s00018-005-5467-7 PMC11136112

[45] Larkin MA, Blackshields G, Brown NP, Chenna R, McGettigan PA, McWilliam H, et al Clustal W and Clustal X version 2.0. Bioinformatics (Oxford, England). 2007;23(21):2947–8. Epub 2007/09/12. 10.1093/bioinformatics/btm404 17846036

[46] Saitou N, Nei M. The neighbor-joining method: a new method for reconstructing phylogenetic trees. Molecular biology and evolution. 1987;4(4):406–25. Epub 1987/07/01. 3447015 10.1093/oxfordjournals.molbev.a040454

[47] Kimura M. A simple method for estimating evolutionary rates of base substitutions through comparative studies of nucleotide sequences. Journal of molecular evolution. 1980;16(2):111–20. Epub 1980/12/01. 7463489 10.1007/BF01731581

[48] Tamura K, Peterson D, Peterson N, Stecher G, Nei M, Kumar S. MEGA5: molecular evolutionary genetics analysis using maximum likelihood, evolutionary distance, and maximum parsimony methods. Molecular biology and evolution. 2011;28(10):2731–9. Epub 2011/05/07. PubMed Central PMCID: PMCPmc3203626. doi: 10.1093/molbev/msr121 21546353 10.1093/molbev/msr121 PMC3203626

[49] Bailey TL, Elkan C. Fitting a mixture model by expectation maximization to discover motifs in biopolymers. Proceedings / International Conference on Intelligent Systems for Molecular Biology; ISMB International Conference on Intelligent Systems for Molecular Biology. 1994;2:28–36. Epub 1994/01/01. 7584402

[50] Grabherr MG, Haas BJ, Yassour M, Levin JZ, Thompson DA, Amit I, et al Full-length transcriptome assembly from RNA-Seq data without a reference genome. Nature biotechnology. 2011;29(7):644–52. Epub 2011/05/17. PubMed Central PMCID: PMC3571712. doi: 10.1038/nbt.1883 21572440 10.1038/nbt.1883 PMC3571712

[51] Huang X, Madan A. CAP3: A DNA sequence assembly program. Genome research. 1999;9(9):868–77. Epub 1999/10/06. PubMed Central PMCID: PMCPmc310812. 10508846 10.1101/gr.9.9.868 PMC310812

[52] Dai X, Zhao PX. psRNATarget: a plant small RNA target analysis server. Nucleic acids research. 2011;39. 10.1093/nar/gkr319 PMC3125753 21622958

[53] Du Z, Zhou X, Ling Y, Zhang Z, Su Z. agriGO: a GO analysis toolkit for the agricultural community. Nucleic acids research. 2010;38(Web Server issue):W64–70. Epub 2010/05/04. PubMed Central PMCID: PMCPmc2896167. doi: 10.1093/nar/gkq310 20435677 10.1093/nar/gkq310 PMC2896167

[54] Qian Y, Cheng Y, Cheng X, Jiang H, Zhu S, Cheng B. Identification and characterization of Dicer-like, Argonaute and RNA-dependent RNA polymerase gene families in maize. Plant cell reports. 2011;30(7):1347–63. Epub 2011/03/16. doi: 10.1007/s00299-011-1046-6 21404010 10.1007/s00299-011-1046-6

[55] Bai M, Yang GS, Chen WT, Mao ZC, Kang HX, Chen GH, et al Genome-wide identification of Dicer-like, Argonaute and RNA-dependent RNA polymerase gene families and their expression analyses in response to viral infection and abiotic stresses in Solanum lycopersicum. Gene. 2012;501(1):52–62. Epub 2012/03/13. doi: 10.1016/j.gene.2012.02.009 22406496 10.1016/j.gene.2012.02.009

[56] Kapoor M, Arora R, Lama T, Nijhawan A, Khurana JP, Tyagi AK, et al Genome-wide identification, organization and phylogenetic analysis of Dicer-like, Argonaute and RNA-dependent RNA Polymerase gene families and their expression analysis during reproductive development and stress in rice. BMC Genomics. 2008;9(1):1–17. 18826656 10.1186/1471-2164-9-451 PMC2576257

[57] Liu B, Li P, Li X, Liu C, Cao S, Chu C, et al Loss of function of OsDCL1 affects microRNA accumulation and causes developmental defects in rice. Plant Physiol. 2005;139. 10.1104/pp.105.063420 PMC1203379 16126864

[58] Margis R, Fusaro AF, Smith NA, Curtin SJ, Watson JM, Finnegan EJ, et al The evolution and diversification of Dicers in plants. FEBS Letters. 2006;580(10):2442–50. doi: 10.1016/j.febslet.2006.03.072 16638569 10.1016/j.febslet.2006.03.072

[59] Ji X. The Mechanism of RNase III Action: How Dicer Dices In: Paddison PJ, Vogt PK, editors. RNA Interference. Berlin, Heidelberg: Springer Berlin Heidelberg; 2008 p. 99–116. 10.1007/978-3-540-75157-1_5 18268841

[60] Vaucheret H. Plant ARGONAUTES. Trends Plant Sci. 2008;13(7):350–8. doi: 10.1016/j.tplants.2008.04.007 18508405 10.1016/j.tplants.2008.04.007

[61] Zhang H, Xia R, Meyers BC, Walbot V. Evolution, functions, and mysteries of plant ARGONAUTE proteins. Current opinion in plant biology. 2015;27:84–90. doi: 10.1016/j.pbi.2015.06.011 26190741 10.1016/j.pbi.2015.06.011

[62] Baumberger N, Baulcombe DC. Arabidopsis ARGONAUTE1 is an RNA Slicer that selectively recruits microRNAs and short interfering RNAs. Proc Natl Acad Sci USA. 2005;102. 10.1073/pnas.0505461102 PMC1182554 16081530

[63] Wassenegger M, Krczal G. Nomenclature and functions of RNA-directed RNA polymerases. Trends Plant Sci. 2006;11(3):142–51. doi: 10.1016/j.tplants.2006.01.003 16473542 10.1016/j.tplants.2006.01.003

[64] Voinnet O. Use, tolerance and avoidance of amplified RNA silencing by plants. Trends Plant Sci. 2008;13(7):317–28. Epub 2008/06/21. doi: 10.1016/j.tplants.2008.05.004 18565786 10.1016/j.tplants.2008.05.004

[65] Wang XB, Wu Q, Ito T, Cillo F, Li WX, Chen X, et al RNAi-mediated viral immunity requires amplification of virus-derived siRNAs in Arabidopsis thaliana. Proc Natl Acad Sci U S A. 2010;107(1):484–9. Epub 2009/12/08. PubMed Central PMCID: PMCPmc2806737. doi: 10.1073/pnas.0904086107 19966292 10.1073/pnas.0904086107 PMC2806737

[66] Willmann MR, Endres MW, Cook RT, Gregory BD. The Functions of RNA-Dependent RNA Polymerases in Arabidopsis. The Arabidopsis Book / American Society of Plant Biologists. 2011;9:e0146. 10.1199/tab.0146 PMC3268507 22303271

[67] Dai XB, Zhao PX. psRNATarget: a plant small RNA target analysis server. Nucleic Acids Research. 2011;39:W155–W9. doi: 10.1093/nar/gkr319 21622958 10.1093/nar/gkr319 PMC3125753

[68] Liu H, Guo S, Xu Y, Li C, Zhang Z, Zhang D, et al OsmiR396d-Regulated OsGRFs Function in Floral Organogenesis in Rice through Binding to Their Targets OsJMJ706 and OsCR4. Plant Physiology. 2014;165(1):160–74. doi: 10.1104/pp.114.235564 24596329 10.1104/pp.114.235564 PMC4012577

[69] Tworak A, Urbanowicz A, Podkowinski J, Kurzynska-Kokorniak A, Koralewska N, Figlerowicz M. Six Medicago truncatula Dicer-like protein genes are expressed in plant cells and upregulated in nodules. Plant cell reports. 2016;35(5):1043–52. doi: 10.1007/s00299-016-1936-8 26825594 10.1007/s00299-016-1936-8 PMC4833791

[70] Qi Y, He X, Wang XJ, Kohany O, Jurka J, Hannon GJ. Distinct catalytic and non-catalytic roles of ARGONAUTE4 in RNA-directed DNA methylation. Nature. 2006;443(7114):1008–12. Epub 2006/09/26. doi: 10.1038/nature05198 16998468 10.1038/nature05198

[71] Zilberman D, Cao X, Jacobsen SE. ARGONAUTE4 control of locus-specific siRNA accumulation and DNA and histone methylation. Science. 2003;299(5607):716–9. Epub 2003/01/11. doi: 10.1126/science.1079695 12522258 10.1126/science.1079695

[72] Zong J, Yao X, Yin J, Zhang D, Ma H. Evolution of the RNA-dependent RNA polymerase (RdRP) genes: Duplications and possible losses before and after the divergence of major eukaryotic groups. Gene. 2009;447(1):29–39. doi: 10.1016/j.gene.2009.07.004 19616606 10.1016/j.gene.2009.07.004

[73] Zhang C, Wu Z, Li Y, Wu J. Biogenesis, Function, and Applications of Virus-Derived Small RNAs in Plants. Frontiers in Microbiology. 2015;6:1237 doi: 10.3389/fmicb.2015.01237 26617580 10.3389/fmicb.2015.01237 PMC4637412

[74] Axtell MJ, Bartel DP. Antiquity of MicroRNAs and Their Targets in Land Plants. The Plant Cell. 2005;17(6):1658–73. doi: 10.1105/tpc.105.032185 15849273 10.1105/tpc.105.032185 PMC1143068

[75] Puzey JR, Karger A, Axtell M, Kramer EM. Deep annotation of Populus trichocarpa microRNAs from diverse tissue sets. PLoS One. 2012;7(3):e33034 PubMed Central PMCID: PMC3307732. doi: 10.1371/journal.pone.0033034 22442676 10.1371/journal.pone.0033034 PMC3307732

[76] Zhang R, Marshall D, Bryan GJ, Hornyik C. Identification and Characterization of miRNA Transcriptome in Potato by High-Throughput Sequencing. PLoS ONE. 2013;8(2):e57233 doi: 10.1371/journal.pone.0057233 23437348 10.1371/journal.pone.0057233 PMC3578796

[77] Liang G, Li Y, He H, Wang F, Yu D. Identification of miRNAs and miRNA-mediated regulatory pathways in Carica papaya. Planta. 2013;238(4):739–52. doi: 10.1007/s00425-013-1929-6 23851604 10.1007/s00425-013-1929-6

[78] Wang JW, Czech B, Weigel D. miR156-regulated SPL transcription factors define an endogenous flowering pathway in Arabidopsis thaliana. Cell. 2009;138(4):738–49. Epub 2009/08/26. doi: 10.1016/j.cell.2009.06.014 19703399 10.1016/j.cell.2009.06.014

[79] Wang Y, Wang Z, Amyot L, Tian L, Xu Z, Gruber MY, et al Ectopic expression of miR156 represses nodulation and causes morphological and developmental changes in Lotus japonicus. Molecular genetics and genomics: MGG. 2015;290(2):471–84. Epub 2014/10/09. PubMed Central PMCID: PMCPmc4361721. doi: 10.1007/s00438-014-0931-4 25293935 10.1007/s00438-014-0931-4 PMC4361721

[80] Xing S, Salinas M, Hohmann S, Berndtgen R, Huijser P. miR156-targeted and nontargeted SBP-box transcription factors act in concert to secure male fertility in Arabidopsis. Plant Cell. 2010;22(12):3935–50. Epub 2010/12/24. PubMed Central PMCID: PMCPmc3027167. doi: 10.1105/tpc.110.079343 21177480 10.1105/tpc.110.079343 PMC3027167

[81] Yu N, Cai WJ, Wang S, Shan CM, Wang LJ, Chen XY. Temporal control of trichome distribution by microRNA156-targeted SPL genes in Arabidopsis thaliana. Plant Cell. 2010;22(7):2322–35. Epub 2010/07/14. PubMed Central PMCID: PMCPmc2929091. doi: 10.1105/tpc.109.072579 20622149 10.1105/tpc.109.072579 PMC2929091

[82] Ostria-Gallardo E, Ranjan A, Chitwood DH, Kumar R, Townsley BT, Ichihashi Y, et al Transcriptomic analysis suggests a key role for SQUAMOSA PROMOTER BINDING PROTEIN LIKE, NAC and YUCCA genes in the heteroblastic development of the temperate rainforest tree Gevuina avellana (Proteaceae). New Phytologist. 2016;210(2):694–708. doi: 10.1111/nph.13776 26680017 10.1111/nph.13776

[83] Aukerman MJ, Sakai H. Regulation of flowering time and floral organ identity by a MicroRNA and its APETALA2-like target genes. Plant Cell. 2003;15(11):2730–41. Epub 2003/10/14. PubMed Central PMCID: PMCPmc280575. doi: 10.1105/tpc.016238 14555699 10.1105/tpc.016238 PMC280575

[84] Wu G, Park MY, Conway SR, Wang JW, Weigel D, Poethig RS. The sequential action of miR156 and miR172 regulates developmental timing in Arabidopsis. Cell. 2009;138. 10.1016/j.cell.2009.06.031 PMC2732587 19703400

[85] Belli Kullan J, Lopes Paim Pinto D, Bertolini E, Fasoli M, Zenoni S, Tornielli GB, et al miRVine: a microRNA expression atlas of grapevine based on small RNA sequencing. BMC Genomics. 2015;16(1):1–23. 25981679 10.1186/s12864-015-1610-5 PMC4434875

[86] Chuck G, Cigan AM, Saeteurn K, Hake S. The heterochronic maize mutant Corngrass1 results from overexpression of a tandem microRNA. Nature genetics. 2007;39. 10.1038/ng2001 17369828

[87] Zhu QH, Helliwell CA. Regulation of flowering time and floral patterning by miR172. J Exp Bot. 2011;62(2):487–95. Epub 2010/10/19. doi: 10.1093/jxb/erq295 20952628 10.1093/jxb/erq295

[88] Sunkar R, Girke T, Jain PK, Zhu JK. Cloning and characterization of microRNAs from rice. Plant Cell. 2005;17(5):1397–411. Epub 2005/04/05. PubMed Central PMCID: PMCPmc1091763. doi: 10.1105/tpc.105.031682 15805478 10.1105/tpc.105.031682 PMC1091763

[89] Sunkar R, Li YF, Jagadeeswaran G. Functions of microRNAs in plant stress responses. Trends Plant Sci. 2012;17(4):196–203. doi: 10.1016/j.tplants.2012.01.010 22365280 10.1016/j.tplants.2012.01.010

[90] Chen L, Wang T, Zhao M, Tian Q, Zhang WH. Identification of aluminum-responsive microRNAs in Medicago truncatula by genome-wide high-throughput sequencing. Planta. 2012;235(2):375–86. Epub 2011/09/13. doi: 10.1007/s00425-011-1514-9 21909758 10.1007/s00425-011-1514-9

[91] Ding Y, Ye Y, Jiang Z, Wang Y, Zhu C. MicroRNA390 Is Involved in Cadmium Tolerance and Accumulation in Rice. Front Plant Sci. 2016;7:235 Epub 2016/03/15. PubMed Central PMCID: PMCPmc4772490. doi: 10.3389/fpls.2016.00235 26973678 10.3389/fpls.2016.00235 PMC4772490

[92] Montgomery TA, Howell MD, Cuperus JT, Li D, Hansen JE, Alexander AL, et al Specificity of ARGONAUTE7-miR390 interaction and dual functionality in TAS3 trans-acting siRNA formation. Cell. 2008;133(1):128–41. Epub 2008/03/18. doi: 10.1016/j.cell.2008.02.033 18342362 10.1016/j.cell.2008.02.033

[93] Stevens PF. Angiosperm Phylogeny Website. Version 12, July 2012 [and more or less continuously updated since]. http://wwwmobotorg/MOBOT/research/APweb/. 2001 onwards.

