# Supplementary figures and images for: A genome-wide analysis of the RNA-guided silencing pathway in coffee reveals insights into its regulatory mechanisms
Source: PLoS One. 2017 Apr 27;12(4):e0176333. doi: 10.1371/journal.pone.0176333 (PMC5407642; doi:10.1371/journal.pone.0176333)

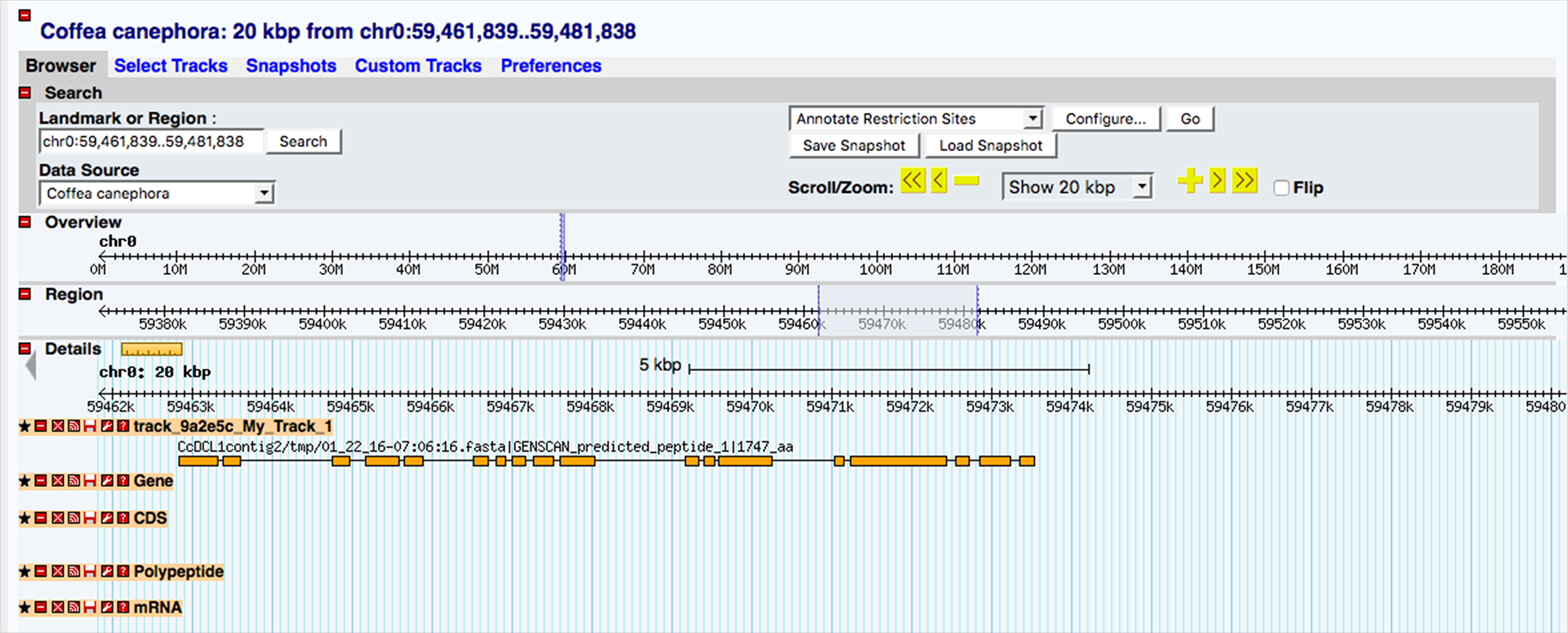

Supplement: S1 Fig — The alignment demonstrates that the DCL1 gene is present in the genome assembly, but it was not previously annotated as a protein-coding gene. (TIF) [file pone.0176333.s001.tif]

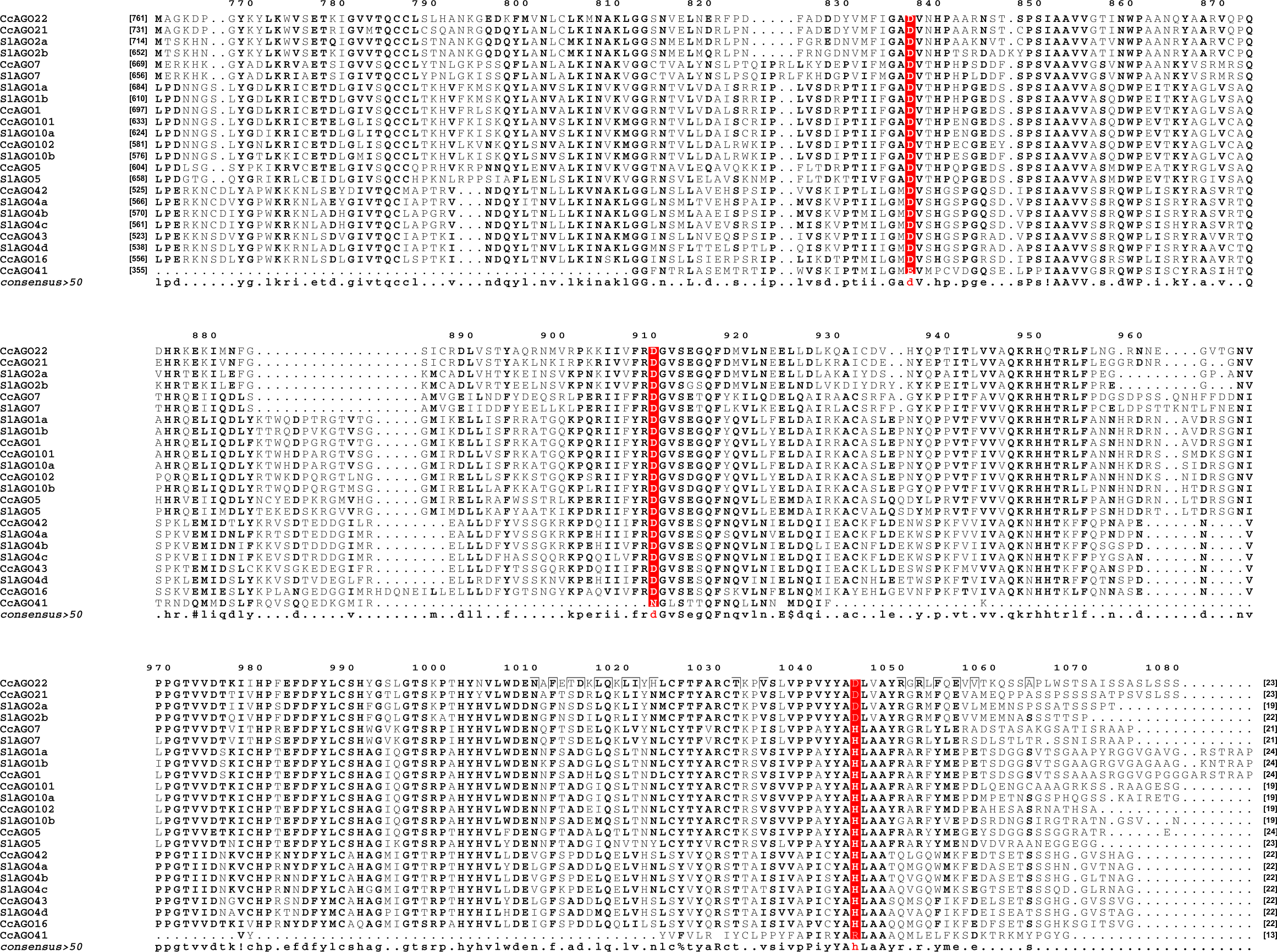

Supplement: S2 Fig — Aminoacids corresponding to the Aspartate-Aspartate-Histidine (DDH) motif at the positions 760, 845, and 986,and an extra Histidine at the position 798 of the AtAGO1 (DDH/H798) [62] are highlighted. Four proteins (CcAGO1, CcAGO5, CcAGO7, and CcAGO10.1) showed the conserved DDH/H798 residues. In four CcAGOs, the DDH catalytic motif was conserved, but the H798 was replaced by a serine (CcAGO16), proline (CcAGO4.2 and CcAGO4.3) or glutamine (CcAGO10.2). Two CcAGO proteins possessed an aspartate residue in place of the third histidine of the DDH motif (CcAGO2.1 and CcAGO2.2). The CcAGO4.1 contains neither the catalytic DDH motif nor the H798 residue. (TIF) [file pone.0176333.s002.tif]

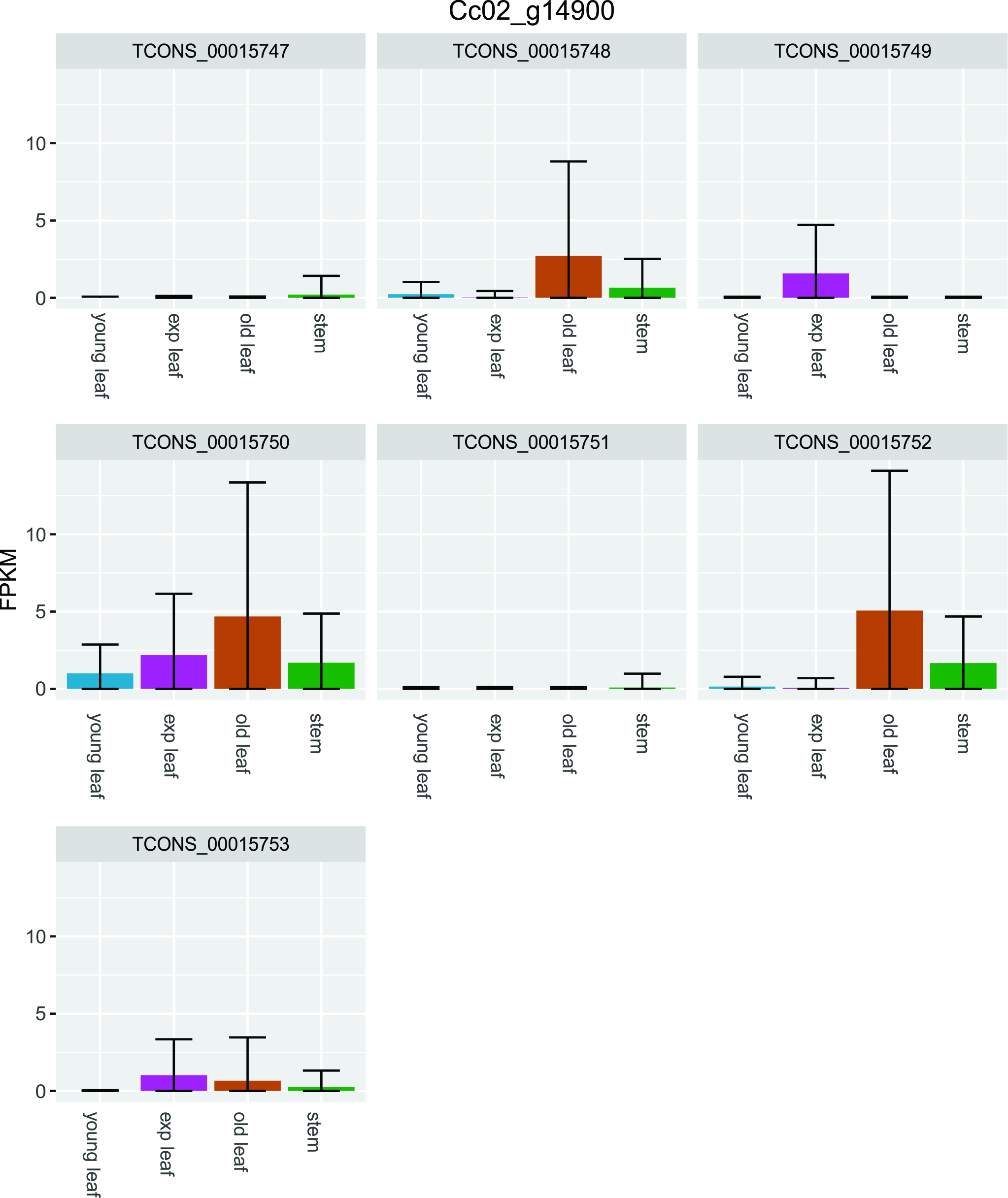

Supplement: S3 Fig — It was analyzed the CcDCL2 expression in three developmental stages of C. canephora leaf—young, expanded (exp in the figure) and old—and stem (Available at https://www.ncbi.nlm.nih.gov/sra/?term=ERP003741). FPKM stands for Fragments Per Kilobase Million. (TIF) [file pone.0176333.s003.tif]
