# Supplementary material for: A genome-wide analysis of the RNA-guided silencing pathway in coffee reveals insights into its regulatory mechanisms
Source: PLoS One. 2017 Apr 27;12(4):e0176333. doi: 10.1371/journal.pone.0176333 (PMC5407642; doi:10.1371/journal.pone.0176333)
Supplement: S1 Table — Protein name, literature reference of the first description in plants, the C. canephora ortholog, locus name and position, and respective protein length. (DOCX) [file pone.0176333.s004.docx]

| Protein | Reference | *C. Canephora*  orthologue | Locus Name | Locus Position | Protein lenght |
| --- | --- | --- | --- | --- | --- |
| CBP20 | Kim et al. (2008) | CcCBP2a | Cc10_g09490 | chr10:9716949..9718869 | 164 |
|  |  | CcCBP2b | Cc10_g09480 | chr10:9713646..9716013 | 125 |
| CBP80 (ABH1) | Kim et al. (2008) | CcCBP1 | Cc05_g16390 | chr5:29002268..29014872 | 863 |
| NOT2b | Wang et al. (2013) | CcpOT2Ba | Cc01_g12100 | chr1:30809877..30816560 | 664 |
|  |  | CcpOT2Bb | Cc08_g14100 | chr8:29149492..29153786 | 525 |
| STA1 | Ben Chaabane et al. (2012) | CcSTA1 | Cc07_g08310 | chr7:6099394..6102468 | 1025 |
| SIC | Zhan et al. (2012) | CcSIC | Cc00_g15770 | chr0:109129392..109130695 | 349 |
| MOS2 | Wu et al. (2013) | CcMOS2a | Cc04_g13510 | chr4:17871067..17872503 | 479 |
|  |  | CcMOS2b | Cc04_g13460 | chr4:17694276..17695712 | 479 |
|  |  | CcMOS2c | Cc02_g26180 | chr2:23672590..23672970 | 127 |
|  |  | CcMOS2d | Cc11_g00690 | chr11:2125784..2126200 | 139 |
| RACK1 | Speth et al. (2013) | CcRACK1 | Cc04_g06620 | chr4:5017989..5027977 | 1396 |
| CPL1 | Jeong et al. (2013) | CcCPL1 | Cc02_g13040 | chr2:11261162..11270645 | 969 |
| SDN1 | Ramachandran and Chen (2008) | CcSDN | Cc07_g06390 | chr7:4636425..4640510 | 495 |
| HSP90 | Iki et al. (2010) | CcHSP83a | Cc02_g02350 | chr2:1933794..1936399 | 706 |
|  |  | CcHSP83b | Cc08_g15170 | chr8:29988829..29991693 | 664 |
|  |  | CcHSP83c | Cc05_g15320 | chr5:28235995..28238998 | 682 |
|  |  | CcHSP83d | Cc10_g01540 | chr10:1126288..1131546 | 816 |
|  |  | CcHSP90a | Cc11_g11420 | chr11:28725834..28731525 | 800 |
|  |  | CcHSP90b | Cc00_g08170 | chr0:68539588..68546659 | 682 |
|  |  | CcHSP83e | Cc02_g32080 | chr2:42452038..42453970 | 160 |
|  |  | CcHSP83f | Cc00_g32810 | chr0:192677035..192677250 | 72 |
|  |  | CcHSP90c | Cc10_g10490 | chr10:12650326..12651022 | 98 |
| SQN | Iki et al. (2011) | CcSQNa | Cc02_g18500 | chr2:16815809..16820731 | 362 |
|  |  | CcSQNb | Cc07_g05790 | chr7:4071365..4075493 | 351 |
| FBW2 | Earley et al. (2010) | CcFBW2a | Cc10_g06830 | chr10:5642938..5644368 | 314 |
|  |  | CcFBW2b | Cc07_g14850 | chr7:11493701..11495351 | 319 |
|  |  | CcFBW2c | Cc02_g30110 | chr2:34065911..34067707 | 369 |
| XRN4 | German et al. (2008) | CcFBW2a | Cc10_g06830 | chr10:5642938..5644368 | 314 |
|  |  | CcFBW2b | Cc07_g14850 | chr7:11493701..11495351 | 319 |
|  |  | CcFBW2c | Cc02_g30110 | chr2:34065911..34067707 | 369 |
| DRB4 | Fukudome et al. (2011) | CcDRB4 | Cc06_g07300 | chr6:5823558..5828818 | 489 |
|  |  | CcDRB4 | Cc00_g06240 | chr0:49689589..49691881 | 284 |
|  |  | CcDRB4 | Cc07_g08100 | chr7:5960006..5964708 | 358 |
|  |  | CcDRB4 | Cc04_g06500 | chr4:4923761..4925559 | 302 |
| AMP1 | Li et al. (2013) | CcpMP1a | Cc06_g07060 | chr6:5658580..5663662 | 697 |
|  |  | CcpMP1b | Cc06_g10580 | chr6:8626239..8633888 | 705 |
| SUO | Yang et al. (2011) | CcSUOa | Cc04_g13000 | chr4:15246252..15254194 | 1609 |
|  |  | CcSUOb | Cc06_g18990 | chr6:19837606..19839610 | 186 |
| HMG1 | Brodersen et al. (2012) | CcHMG1a | Cc07_g12220 | chr7:9027895..9031232 | 605 |
|  |  | CcHMG1b | Cc10_g00230 | chr10:221651..225100 | 573 |
| VCS | Xu and Chua (2011); Motomura et al, (2012) | CcVCS | Cc05_g15500 | chr5:28347339..28357579 | 1457 |
| DCP2 | Xu and Chua (2011); Motomura et al, (2012) | CcDCP2 | Cc02_g11340 | chr2:9315991..9323302 | 323 |
| DCP1 | Xu and Chua (2011); Motomura et al, (2012) | CcDCP1a | Cc01_g08840 | chr1:27450686..27458554 | 371 |
|  |  | CcDCP1b | Cc06_g06200 | chr6:4882013..4885571 | 377 |
| DCP5 | Xu and Chua (2011) | CcDCP5a | Cc11_g10750 | chr11:28134470..28139535 | 603 |
|  |  | CcDCP5b | Cc08_g06000 | chr8:11099183..11110514 | 549 |
|  |  | CcDCP5c | Cc04_g07630 | chr4:6033872..6041183 | 719 |
| SGS3 | Yoshikawa et al. (2013) | CcSGS3a | Cc00_g00830 | chr0:2001507..2005162 | 510 |
|  |  | CcSGS3b | Cc00_g17640 | chr0:116948211..116948729 | 137 |
| SDE5 | Hernandez-Pinzon et al. (2007) | CcSDE5 | Cc05_g12220 | chr5:26107836..26112896 | 469 |
|  |  |  |  |  |  |
| DRM1 | Cao and Jacobsen (2002) | - | - | - |  |
| DRM2 | Cao and Jacobsen (2002) | CcDRM2a | Cc09_g04720 | chr9:4145815..4154188 | 600 |
|  |  | CcDRM2b | Cc11_g15320 | chr11:31810504..31814616 | 636 |
|  |  | CcDRM2c | Cc03_g00380 | chr3:232974..241612 | 754 |
|  |  | CcDRM2d | Cc04_g13650 | chr4:18811123..18811937 | 114 |
| CMT3 | Lindroth et al. (2001) | CcCMT3 | Cc01_g01020 | chr1:1605918..1613970 | 888 |
| MET1 | Kankel et al. (2003) | CcMET1a | Cc07_g19880 | chr7:19879874..19885755 | 1499 |
|  |  | CcMET1b | Cc02_g23510 | chr2:20674668..20681302 | 1497 |
| DDM1 | Brzeski and Jerzmanowski (2003) | CcDDM1 | Cc07_g09410 | chr7:6901156..6907435 | 757 |
| Pol IV (NRPD1) | Onodera et al. (2005) | CcPol IV (NRPD1) | Cc08_g13910 | chr8:28953357..28965448 | 1512 |
| Pol IV (NRPD2) | Onodera et al. (2005) | CcPol IV (NRPD2a) | Cc08_g05510 | chr8:9979964..9988527 | 1231 |
|  |  | CcPol IV (NRPD2b) | Cc01_g06880 | chr1:24215568..24222564 | 1214 |
| Pol V (NRPE1) | Pontes et al. (2006) | CcPol V (NRPE1) | Cc02_g30260 | chr2:34749218..34768492 | 1947 |
| CLSY1 | Smith et al. (2007) | CcCLSY1 | Cc02_g05980 | chr2:4667892..4673102 | 1278 |
| DRD1 | Pontes et al. (2006) | CcDRD1 | Cc00_g19470 | chr0:125203470..125207481 | 735 |
|  |  | CcDRD1 | Cc02_g17200 | chr2:15848149..15857128 | 907 |
| DMS3 | Wierzbicki et al. (2009) | CcDMS3 | Cc09_g00180 | chr9:120293..126127 | 403 |
| RDM1 | Gao et al. (2010); Law et al. (2011) | CcRDM1 | Cc10_g08560 | chr10:7892737..7893906 | 175 |
| SHH1 | Law et al. (2011) | CcSHH2 | Cc04_g02640 | chr4:2044549..2048483 | 269 |
|  |  | CcSHH1 | Cc08_g09850 | chr8:24669321..24674468 | 256 |
| RCF3 | Karlsson et al. (2015) |  | Cc01_g04120 | chr1:11014076..11020408 | 646 |
|  |  |  | Cc00_g15730 | chr0:108901695..108906987 | 667 |
|  |  |  | Cc07_g03520 | chr7:2447752..2450618 | 560 |
|  |  |  | Cc11_g12790 | chr11:29789123..29792081 | 536 |
|  |  |  | Cc01_g04120 | chr1:11014076..11020408 | 646 |
| CPL2 | Karlsson et al. (2016) |  | Cc11_g02780 | chr11:10677935..10682194 | 337 |
| THO2 | Francisco-Mangilet et al. (2015) |  | no rusults |  |  |
| CMA33/XCT | Fang et al. (2015) |  | Cc11_g02780 | chr11:10677935..10682194 | 338 |
| URT1 | Tu et al. (2015) |  | Cc00_g19600 | chr0:126130302..126137060 | 777 |
| GRP7 | Köster et al. (2014) |  | Cc02_g21970 | chr2:19460713..19461628 | 207 |
|  |  |  | Cc00_g34700 | chr0:200714167..200714938 | 161 |
