# Supplementary material for: A genome-wide analysis of the RNA-guided silencing pathway in coffee reveals insights into its regulatory mechanisms
Source: PLoS One. 2017 Apr 27;12(4):e0176333. doi: 10.1371/journal.pone.0176333 (PMC5407642; doi:10.1371/journal.pone.0176333)
Supplement: S2 Table — Precursor names, chromosome numbers, start and end positions, strand and genic/intergenic locations, mature 5p and/or 3p miRNAs, start and end positions in the precursor, and mature miRNA sizes. (DOCX) [file pone.0176333.s005.docx]

| Precursor name | Chromossome | Start | End | Strand | Genomic Location | Mature Name | Mature Sequence | Start and end  in precursor | Size of the  miRNA |
| --- | --- | --- | --- | --- | --- | --- | --- | --- | --- |
| ccp-miR1023e | chr11 | 29816068 | 29816402 | minus | intergenic | ccp-miR1023e-5p | ACACUCUCUCCGUCUCACUUU | 2-22 | 21 |
| ccp-miR1030i | chr1 | 29153533 | 29153834 | plus | intergenic | ccp-miR1030i-5p | CCUGCAUCUGCACCCGCACCU | 2-22 | 21 |
| ccp-miR1060 | chr5 | 15349464 | 15349571 | plus | intergenic | ccp-miR1060-5p | UUUGUCAUAGGAUUACAUAUA | 22-42 | 21 |
| ccp-miR1078 | chr3 | 28074406 | 28074511 | minus | intergenic | ccp-miR1078-5p | CUUGAUUGAUUCAAUUGAUUU | 14-34 | 21 |
| ccp-miR1089 | chr1 | 26510757 | 26510887 | minus | intergenic | ccp-miR1089-5p | AGAUCAUCUUGCAUUGUUUGA | 28-48 | 21 |
| ccp-miR1127-1 | chr3 | 31292890 | 31293028 | plus | intergenic | ccp-miR1127-1-3p | AACUACUCCCUCCGUCCCGUU | 92-112 | 21 |
| ccp-miR1127-2 | chr4 | 25187456 | 25187738 | plus | intergenic | ccp-miR1127-2-5p | AACUACUCCCUCCGUCUCAUA | 1-21 | 21 |
| ccp-miR1128-1 | chr2 | 12870351 | 12870633 | minus | intergenic | ccp-miR1128-1-5p | UACUACUCCCUCCGUCCCACU | 11-31 | 21 |
| ccp-miR1128-2 | chr1 | 19493752 | 19494033 | minus | intergenic | ccp-miR1128-2-3p | UACUACUCCCUCCGUCCCACU | 218-238 | 21 |
| ccp-miR1128-3 | chr7 | 7667758 | 7667880 | minus | intergenic | ccp-miR1128-3-3p | UACUACUCCCUCCGUCCCAUU | 77-97 | 21 |
| ccp-miR1128-4 | chr3 | 21492797 | 21493078 | plus | intergenic | ccp-miR1128-4-5p | UACUACUCCCUCCGUCCCAUU | 7-27 | 21 |
| ccp-miR1435 | chr11 | 20107604 | 20107711 | plus | genic | ccp-miR1435-3p | UUUCUUAAAACAAACUUUUU | 57-76 | 20 |
| ccp-miR1438-1 | chr4 | 6900340 | 6900608 | plus | genic | ccp-miR1438-1-3p | AGGGUAAUUUUGACAUUUUUAG | 211-232 | 22 |
| ccp-miR1438-2 | chr0 | 195826857 | 195827087 | minus | intergenic | ccp-miR1438-2-3p | AGGGUAAUUUUGACAUUUUUAG | 191-212 | 22 |
| ccp-miR1438-3 | chr7 | 14373657 | 14373851 | minus | genic | ccp-miR1438-3-5p | AGGGUAAUUUUGACAUUUUUAG | 18-39 | 22 |
| ccp-miR1446 | chr4 | 4231562 | 4231691 | minus | intergenic | ccp-miR1446-5p | UGAACUCUCUCCCUCAACGGCU | 19-40 | 22 |
| ccp-miR1510b | chr8 | 17939817 | 17939884 | minus | intergenic | ccp-miR1510b-3p | GUUGUUUUACCUAUUCCACUCAUU | 42-65 | 24 |
| ccp-miR1515a | chr7 | 16885396 | 16885514 | minus | intergenic | ccp-miR1515a-3p | UCAUUUUGUGUGCCAUGAUCCA | 42-63 | 22 |
| ccp-miR1520e | chr8 | 20767280 | 20767566 | minus | intergenic | ccp-miR1520e-5p | CAAUAAGACCAUGAGAUUUGACAG | 120-143 | 24 |
| ccp-miR1526 | chr2 | 22077816 | 22077933 | minus | intergenic | ccp-miR1526-5p | CCGGAAGAAGCAAAUUAAGUAA | 34-55 | 22 |
| ccp-miR156 | chr0 | 173196199 | 173196300 | plus | intergenic | ccp-miR156-3p | UGCUCACUCUCUAUCUGUCACC | 79-100 | 22 |
|  |  |  |  |  |  | ccp-miR156-5p | UGACAGAAGAGAGUGAGCACA | 5-25 | 21 |
| ccp-miR156f | chr5 | 28049004 | 28049090 | minus | intergenic | ccp-miR156f-5p | UUGACAGAAGAGAGAGAGCACA | 3-24 | 22 |
| ccp-miR156g | chr6 | 9429886 | 9429977 | plus | intergenic | ccp-miR156g-5p | CGACAGAAGAGAGUGAGCAC | 5-24 | 20 |
| ccp-miR157 | chr4 | 5161806 | 5161889 | minus | intergenic | ccp-miR157-3p | GCUCUCUAUGCUUCUGUCA | 66-84 | 19 |
|  |  |  |  |  |  | ccp-miR157-5p | UUGACAGAAGAUAGAGAGCAC | 1-21 | 21 |
| ccp-miR157a-1 | chr7 | 6213187 | 6213288 | minus | intergenic | ccp-miR157a-1-3p | GCUCUCUAGUCUUCUGUCAUCA | 77-98 | 22 |
|  |  |  |  |  |  | ccp-miR157a-1-5p | UUGACAGAAGAUAGAGAGCAC | 9-29 | 21 |
| ccp-miR157a-2 | chr7 | 122554 | 122638 | minus | intergenic | ccp-miR157a-2-5p | UUGACAGAAGAUAGAGAGCAC | 1-21 | 21 |
| ccp-miR159a | chr4 | 5047095 | 5047282 | minus | intergenic | ccp-miR159a-3p | UUUGGAUUGAAGGGAGCUCUA | 161-181 | 21 |
|  |  |  |  |  |  | ccp-miR159a-5p | GAGCUCCUUGAAGUCCAAUAG | 8-28 | 21 |
| ccp-miR159b | chr1 | 25322422 | 25322501 | plus | intergenic | ccp-miR159b-3p | UUGGAGUGAAGGGAGCUCCAG | 43-63 | 21 |
| ccp-miR160 | chr9 | 1683434 | 1683517 | plus | intergenic | ccp-miR160-3p | GCGUAUGAGGAGCCAUGCAUA | 64-84 | 21 |
|  |  |  |  |  |  | ccp-miR160-5p | UGCCUGGCUCCCUGUAUGCCAUU | 2-24 | 23 |
| ccp-miR160a-1 | chr5 | 24196320 | 24196417 | minus | genic | ccp-miR160a-1-3p | GCAUCAGAGGAGUCAGGCAGG | 73-93 | 21 |
|  |  |  |  |  |  | ccp-miR160a-1-5p | UGCCUGGCUCCCUGGAUGCCAUC | 8-30 | 23 |
| ccp-miR160a-2 | chr5 | 24201506 | 24201589 | minus | genic | ccp-miR160a-2-5p | UGCCUGGCUCCCUGGAUGCCAUC | 1-23 | 23 |
| ccp-miR160h | chr9 | 8792199 | 8792288 | plus | intergenic | ccp-miR160h-5p | UGCCUGGCUCCCUGCAUGCCA | 4-24 | 21 |
| ccp-miR162a | chr4 | 6151819 | 6151993 | plus | intergenic | ccp-miR162a-3p | UCGAUAAACCUCUGCAUCCAG | 119-139 | 21 |
| genic |  |  |  |  |  | ccp-miR162a-5p | UGGAGGCAGCGGUUCAUCGAUC | 27-48 | 22 |
| ccp-miR164-1 | chr4 | 723581 | 723695 | plus | intergenic | ccp-miR164-1-3p | CAUGUGCCCUGCUUCUCCACC | 75-95 | 21 |
|  |  |  |  |  |  | ccp-miR164-1-5p | UGGCAUGUGCCCUGCUUCUCCA | 24-45 | 22 |
| ccp-miR164-2 | chr4 | 723588 | 723687 | minus | intergenic | ccp-miR164-2-3p | CAUGUGCCCUGAUCCUCCAUC | 66-86 | 21 |
|  |  |  |  |  |  | ccp-miR164-2-5p | UGGAGAAGCAGGGCACAUGCC | 15-35 | 21 |
| ccp-miR164-3 | chr2 | 47655449 | 47655564 | minus | intergenic | ccp-mir164-3-3p | CACGUGCUCCCCUUCUCCAAC | 78-98 | 21 |
|  |  |  |  |  |  | ccp-mir164-3-5p | UGGAGAAGCAGGGCACGUGCAA | 21-42 | 22 |
| ccp-miR164a | chr6 | 513551 | 513767 | minus | intergenic | ccp-miR164a-5p | UGGAGAAGCAGGGCACGUGCA | 11-31 | 21 |
| ccp-miR164c | chr2 | 47655449 | 47655564 | plus | intergenic | ccp-miR164c-3p | CACGUGCCCUGCUUCUCCAAC | 78-98 | 21 |
|  |  |  |  |  |  | ccp-miR164c-5p | UGGAGAAGGGGAGCACGUGCA | 21-41 | 21 |
| ccp-miR166-1 | chr2 | 54264944 | 54265104 | plus | genic | ccp-miR166-1-3p | UCGGACCAGGCUUCAUUCCCCC | 133-154 | 22 |
| ccp-miR166-2 | chr3 | 3908880 | 3908989 | minus | intergenic | ccp-miR166-2-3p | UCGGACCAGGCUUCAUUCCCCC | 82-103 | 22 |
| ccp-miR166-3 | chr8 | 31212095 | 31212273 | plus | intergenic | ccp-miR166-3-3p | UCGGACCAGGCUUCAUU | 163-179 | 17 |
|  |  |  |  |  |  | ccp-miR166-3-5p | GGAAUGUUGUCUGGUGCGAGG | 22-42 | 21 |
| ccp-miR166-4 | chr6 | 5768093 | 5768290 | minus | intergenic | ccp-miR166-4-3p | UCGGACCAGGCUUCAUUCCUC | 160-180 | 21 |
|  |  |  |  |  |  | ccp-miR166-4-5p | AAUGAAGUUUGAUCCAAGAUC | 22-42 | 21 |
| ccp-miR166a-1 | chr2 | 19855688 | 19855841 | plus | intergenic | ccp-miR166a-1-3p | UCGGACCAGGCUUCAUUCCCC | 124-144 | 21 |
| ccp-miR166a-2 | chr1 | 35697997 | 35698117 | plus | genic | ccp-miR166a-2-3p | UCGGACCAGGCUUCAUUCCCC | 93-113 | 21 |
| ccp-miR166a-3 | chr1 | 35697998 | 35698116 | minus | intergenic | ccp-miR166a-3-3p | UCGAACCAGACGACAUUCCCC | 92-112 | 21 |
| ccp-miR167-1 | chr1 | 35954231 | 35954325 | minus | intergenic | ccp-miR167-1-3p | GGUCAUGCUCUGACAGCCUCACU | 65-87 | 23 |
|  |  |  |  |  |  | ccp-miR167-1-5p | UGAAGCUGCCAGCAUGAUCUA | 11-31 | 21 |
| ccp-miR167-2 | chr3 | 3324014 | 3324110 | minus | genic | ccp-miR167-2-3p | AUCAUGCUGGCAGCUUCAACUGAU | 66-89 | 24 |
|  |  |  |  |  |  | ccp-miR167-2-5p | UGAAGCUACCA-CAUGAUCUGAU | 15-36 | 22 |
| ccp-miR167-3 | chr3 | 3324027 | 3324098 | plus | intergenic | ccp-miR167-3-3p | UGAAGCUGCCAGCAUGAUCUAA | 2-23 | 22 |
|  |  |  |  |  |  | ccp-miR167-3-5p | GAUCAUGUGGUAGCUUCACC | 53-72 | 20 |
| ccp-miR167-4 | chr9 | 6898984 | 6899319 | plus | intergenic | ccp-miR167-4-5p | UGAAGCUGCCAGCAUGAUCUGG | 12-33 | 22 |
| ccp-miR167-5 | chr1 | 35945445 | 35945532 | minus | intergenic | ccp-miR167-5-3p | GAUCUUGCGGUAGCCUCACGA | 60-80 | 21 |
|  |  |  |  |  |  | ccp-miR167-5-5p | UGAAGCUGCCAACACGAUCUUA | 12-33 | 21 |
| ccp-miR167a | chr2 | 36665766 | 36665844 | minus | intergenic | ccp-miR167a-3p | AGAUCAUGCGGUAGUUUCACC | 55-75 | 21 |
|  |  |  |  |  |  | ccp-miR167a-5p | UGAAGCUGCCAGCAUGAUCUGA | 6-27 | 22 |
| ccp-miR167h | chr2 | 36665750 | 36665860 | plus | intergenic | ccp-miR167h-3p | AUCAUGCUGGCAGCUUCAACUACG | 73-96 | 24 |
| ccp-miR168a | chr8 | 6480115 | 6480260 | plus | intergenic | ccp-miR168a-3p | CCCGCCUUGCAUCAACUGAAU | 106-126 | 21 |
|  |  |  |  |  |  | ccp-miR168a-5p | UCGCUUGGUGCAGGUCGGGAA | 23-43 | 21 |
| ccp-miR169-1 | chr7 | 2455308 | 2455465 | plus | intergenic | ccp-miR169-1-3p | GGCAAGUUGUUAUUGGCUACA | 102-122 | 21 |
|  |  |  |  |  |  | ccp-miR169-1-5p | CAGCCAAGGAUGACUUGCCGG | 46-66 | 21 |
| ccp-miR169-2 | chr0 | 146466173 | 146466289 | plus | intergenic | ccp-miR169-2-3p | GGCAAGUUGUCUUUGGCUACA | 97-117 | 21 |
|  |  |  |  |  |  | ccp-miR169-2-5p | CAGCCAAGGAUGACUUGCCGA | 3-23 | 21 |
| ccp-miR169-3 | chr0 | 585806 | 585966 | minus | intergenic | ccp-miR169-3-3p | GGCAAGUUGUCCUUGGCUACG | 106-126 | 21 |
|  |  |  |  |  |  | ccp-miR169-3-5p | CAGCCAAGGAUGACUUGCCGG | 41-61 | 21 |
| ccp-miR169-4 | chr2 | 9919579 | 9919690 | minus | intergenic | ccp-miR169-4-3p | AAGCCAAGGAUCGGUUGCCUC | 55-75 | 21 |
| ccp-miR169a | chr11 | 29783318 | 29783399 | minus | genic | ccp-miR169a-3p | CAGCCAAGGAUGACUU | 67-82 | 16 |
| ccp-miR169d | chr6 | 32494985 | 32495167 | plus | intergenic | ccp-miR169d-5p | AGCCAAGGAUGAAUUGCCGGC | 47-67 | 21 |
| ccp-miR169e | chr7 | 5600973 | 5601167 | minus | intergenic | ccp-miR169e-5p | UAGCCAAGGAUGGCUUGCCUCU | 56-77 | 22 |
| ccp-miR169f-1 | chr4 | 7978815 | 7979029 | minus | intergenic | ccp-miR169f-1-5p | UAGCCAAGGAUGACUUGCCUA | 44-64 | 21 |
| ccp-miR169f-2 | chr4 | 7990124 | 7990326 | minus | intergenic | ccp-miR169f-2-5p | AAGCCAAGGAUGACUUGCCUA | 41-61 | 21 |
| ccp-miR169g | chr7 | 7331818 | 7332001 | plus | intergenic | ccp-miR169g-5p | UAGCCAAGGAUGACUUGCCUGC | 50-71 | 22 |
| ccp-miR171-10 | chr6 | 6553653 | 6553754 | minus | intergenic | ccp-miR171-10-3p | AGAUUGAGCCGCGCCAAUAUC | 75-95 | 21 |
|  |  |  |  |  |  | ccp-miR171-10-5p | UAUUGGUGAGGUUCAAUCCGA | 11-31 | 21 |
| ccp-miR171-11 | chr6 | 6553651 | 6553755 | plus | genic | ccp-miR171-11-3p | GAUUGAACCUCACCAAUAUCG | 77-97 | 21 |
|  |  |  |  |  |  | ccp-miR171-11-5p | GAUAUUGGCGCGGCUCAAUC | 10-29 | 21 |
| ccp-miR171-12 | chr3 | 18565902 | 18565992 | minus | intergenic | ccp-miR-171-12-3p | UGAUUGAACCGUGCCAACAUC | 65-85 | 21 |
|  |  |  |  |  |  | ccp-miR171-12-5p | CGAUAUUGGCACGGCUCAAUC | 6-26 | 21 |
| ccp-miR171-13 | chr5 | 18097401 | 18097493 | minus | intergenic | ccp-miR171-13-3p | AUUGAACCGCACCAAUAUCCC | 67-87 | 21 |
|  |  |  |  |  |  | ccp-miR171-13-5p | ACUACGUGAUAUUGGCACGGCUC | 2-24 | 23 |
| ccp-miR171-1 | chr7 | 8354901 | 8355046 | plus | intergenic | ccp-miR171-1-3p | UGAUUGAGCCGUGCCAAUAUC | 108-128 | 21 |
|  |  |  |  |  |  | ccp-miR171-1-5p | UAUUGGCCUGGUUCACUCAGA | 22-42 | 21 |
| ccp-miR171-2 | chr7 | 8354901 | 8355046 | minus | intergenic | ccp-miR171-2-3p | GAUAUUGGCACGGCUCAAUCA | 107-127 | 21 |
|  |  |  |  |  |  | ccp-miR171-2-5p | GAUAUUGGCACGGCUCAAUC | 19-39 | 21 |
| ccp-miR171-3 | chr10 | 844792 | 844960 | plus | intergenic | ccp-miR171-3-3p | UGAUUGAGCCGUGCCAAUAUC | 130-150 | 21 |
|  |  |  |  |  |  | ccp-miR171-3-5p | UGUGAUAUUGGUCCGGUUCACU | 16-37 | 22 |
| ccp-miR171-4 | chr3 | 18565902 | 18565992 | plus | intergenic | ccp-miR171-4-3p | UGAUUGAGCCGUGCCAAUAUC | 65-85 | 21 |
|  |  |  |  |  |  | ccp-miR171-4-5p | UGUUGGCACGGUUCAAUCACA | 9-29 | 21 |
| ccp-miR171-5 | chr4 | 4652784 | 4652876 | plus | intergenic | ccp-miR171-5-3p | UGAUUGAGCCGCGCCAAUAUC | 66-86 | 21 |
|  |  |  |  |  |  | ccp-miR171-5-5p | AGAUAUUGGUCCGGUUCAAUC | 7-27 | 21 |
| ccp-miR171-6 | chr4 | 4652782 | 4652878 | minus | intergenic | ccp-miR171-6-3p | UGAUUGAACCGGACCAAUAUC | 68-88 | 21 |
|  |  |  |  |  |  | ccp-miR171-6-5p | GAUAUUGGCGCGGCUCAAUC | 10-29 | 20 |
| ccp-miR171-7 | chr5 | 18097410 | 18097485 | plus | intergenic | ccp-miR171-7-3p | UUGAGCCGUGCCAAUAUC | 59-76 | 18 |
|  |  |  |  |  |  | ccp-miR171-7-5p | UAUUGGUGCGGUUCAAUGGCA | 2-22 | 21 |
| ccp-miR171-8 | chr9 | 1285045 | 1285122 | plus | intergenic | ccp-miR171-8-3p | UGAUUGAGCCGUGCCAAUAUC | 58-78 | 21 |
|  |  |  |  |  |  | ccp-miR171-8-5p | UAUUGGUGCGGUUCAAUUAGA | 2-22 | 21 |
| ccp-miR171-9 | chr5 | 18036735 | 18036824 | plus | intergenic | ccp-miR171-9-3p | UGAUUGAGCCGCGUCAAUAUC | 67-87 | 21 |
|  |  |  |  |  |  | ccp-miR171-9-5p | AGGUAUUGAUGCGCCUCAAUC | 2-22 | 21 |
| ccp-miR171b-1 | chr9 | 1285009 | 1285159 | minus | intergenic | ccp-miR171b-1-3p | UAAUUGAACCGCACCAAUAUC | 96-116 | 21 |
|  |  |  |  |  |  | ccp-miR171b-1-5p | UAUUGGCACGGCUCAAUCAAA | 40-60 | 21 |
| ccp-miR171b-2 | chr5 | 18036729 | 18036830 | minus | intergenic | ccp-miR171b-2-3p | UUGAGGCGCAUCAAUACCUCU | 77-97 | 21 |
|  |  |  |  |  |  | ccp-miR171b-2-5p | GAUAUUGACGCGGCUCAAUC | 10-29 | 20 |
| ccp-miR171f | chr2 | 943789 | 943944 | plus | intergenic | ccp-miR171f-3p | AUGAGCCGAACCAAUAUCACU | 118-138 | 21 |
| ccp-miR172-1 | chr2 | 6092599 | 6092774 | plus | intergenic | ccp-miR172-1-3p | AGAAUCUUGAUGAUGCUCCAC |  | 21 |
|  |  |  |  |  |  | ccp-miR172-1-5p | GCAGCAUCAUCAAGAUUCCCG | 21-41 | 21 |
| ccp-miR172-2 | chr2 | 8974709 | 8974816 | plus | intergenic | ccp-miR172-2-3p | GGAAUCUUGAUGAUGCUGCAUCAG | 80-103 | 24 |
|  |  |  |  |  |  | ccp-miR172-2-5p | GCGGCAUCAUCAAGAUUCACACA | 11-33 | 23 |
| ccp-miR172-3 | chr2 | 8974715 | 8974810 | minus | genic | ccp-miR172-3-3p | UGAAUCUUGAUGAUGCCGCAC | 74-94 | 21 |
|  |  |  |  |  |  | ccp-miR172-3-5p | CAGCAUCAUCAAGAUUCCCA |  | 20 |
| ccp-miR172d-1 | chr2 | 6092599 | 6092774 | minus | intergenic | ccp-miR172d-1-3p | GGAAUCUUGAUGAUGCUGCAGCAG | 138-161 | 24 |
|  |  |  |  |  |  | ccp-miR172d-1-5p | GUGGAGCAUCAUCAAGAUUCACGA | 19-42 | 24 |
| ccp-miR172d-2 | chr1 | 29319096 | 29319258 | plus | intergenic | ccp-miR172d-2-3p | UGAGAAUCUUGAUGAUGCUGCAU | 123-145 | 23 |
|  |  |  |  |  |  | ccp-miR172d-2-5p | GUGUAGCAUCAUCAAGAUUCACAU | 19-42 | 24 |
| ccp-miR174e | chr7 | 25162633 | 25162947 | minus | intergenic | ccp-miR174e-3p | UUUAUGGAACAGAGGGAGUAG | 279-299 | 21 |
| ccp-miR1863b-1 | chr2 | 2265859 | 2266030 | minus | intergenic | ccp-miR1863b-1-3p | AGCUCUGAUACCAUGUUAACUCAC | 92-115 | 24 |
| ccp-miR1863b-2 | chr9 | 22171846 | 22171928 | plus | intergenic | ccp-miR1863b-2-5p | AGCUCUGAUACCAACUUAUCCGUU | 3-26 | 24 |
| ccp-miR1878 | chr0 | 18237263 | 18237346 | plus | intergenic | ccp-miR1878-3p | AUUUGUAGUGUUCAAAUAGAGCUU | 31-54 | 24 |
| ccp-miR1885a | chr10 | 26564027 | 26564122 | minus | genic | ccp-miR1885a-3p | CAUCAAUGAAAAGUAUUAUUGU | 60-81 | 22 |
| ccp-miR1919 | chr11 | 5265960 | 5266116 | minus | intergenic | ccp-miR1919-5p | UGUCGCAGGUGACUUUCGCCU | 50-70 | 21 |
| ccp-miR2105 | chr3 | 521229 | 521414 | plus | intergenic | ccp-miR2105-3p | UUGUGAUGUGAAUGAUUACA | 161-180 | 20 |
| ccp-miR2111 | chr6 | 1734988 | 1735064 | plus | intergenic | ccp-miR2111-3p | UCCUCAGGAUACAGAUUACCU | 57-77 | 21 |
|  |  |  |  |  |  | ccp-miR2111-5p | UAAUCUGCAUCCUGAGGUUUA | 5-25 | 21 |
| ccp-miR2275d-1 | chr11 | 31425012 | 31425093 | plus | intergenic | ccp-miR2275d-1-5p | AGAGUUGGAGAAAAGAUAACC | 16-36 | 21 |
| ccp-miR2275d-2 | chr7 | 12768137 | 12768247 | plus | genic | ccp-miR2275d-2-3p | CUUGUUUUUCUCCUUUAUCUCU | 90-111 | 22 |
| ccp-miR2592-1 | chr8 | 16592820 | 16592911 | minus | intergenic | ccp-miR2592-1-5p | GAGUAAUUCGAACUUGAUAAGG | 22-43 | 22 |
| ccp-miR2592-2 | chr5 | 22865988 | 22866270 | minus | intergenic | ccp-miR2592-2-5p | GACUAGGACCAAAGUAUUUCC | 40-60 | 21 |
| ccp-miR2592s-1 | chr3 | 23902716 | 23902942 | minus | genic | ccp-miR2592s-1-3p | AAAUGCUUGAUUAAUGUUGUU | 176-196 | 21 |
| ccp-miR2592s-2 | chr6 | 31877069 | 31877347 | minus | intergenic | ccp-miR2592s-2-3p | AAAUGCUUGAUUAAUGUUGUU | 187-207 | 21 |
| ccp-miR2612 | chr7 | 17523955 | 17524195 | plus | genic | ccp-miR2612-5p | UGAUAGUGUCAACUAUUACCU | 5-25 | 21 |
| ccp-miR2642 | chr7 | 29550665 | 29550950 | minus | intergenic | ccp-miR2642-3p | AUGAGUUUCUUCAAAUCAUUU | 173-193 | 21 |
| ccp-miR2657 | chr4 | 28005954 | 28006121 | plus | genic | ccp-miR2657-3p | UGUUAUUUCAACGGUUUUGUUA | 85-106 | 22 |
| ccp-miR2669a | chr5 | 25191368 | 25191488 | plus | genic | ccp-miR2669a-5p | AAAGUUCAGUCUUCAAAAUGUC | 8-29 | 22 |
| ccp-miR2673a-1 | chr1 | 37201592 | 37201736 | plus | intergenic | ccp-miR2673a-1-5p | CCUCUUCCUCUUCCUCUUUCAU | 3-24 | 22 |
| ccp-miR2673a-2 | chr2 | 15061839 | 15062051 | minus | intergenic | ccp-miR2673a-2-3p | CCUCUUCCUCUUCCUCUUCCGC | 132-153 | 22 |
| ccp-miR2873b-1 | chr8 | 7247424 | 7247593 | plus | intergenic | ccp-miR2873b-1-5p | UUGGACUUGAGAUUUAGAAUA | 25-45 | 21 |
| ccp-miR2873b-2 | chr7 | 15656034 | 15656321 | plus | intergenic | ccp-miR2873b-2-5p | UUGGACUUGAGAUUUGGAGGU | 84-104 | 21 |
| ccp-miR2923 | chr3 | 24214525 | 24214640 | minus | intergenic | ccp-miR2923-3p | AGACAAAAAUAUAGACACCAAA | 73-94 | 22 |
| ccp-miR319-1 | chr1 | 33446228 | 33446405 | plus | intergenic | ccp-miR319-1-3p | UUGGACUGAAGGGAGCUCCCU | 157-177 | 21 |
|  |  |  |  |  |  | ccp-miR319-1-5p | GAGCUUUCUUCAGUCCACUCA | 6-26 | 21 |
| ccp-miR319a-1 | chr4 | 5529729 | 5529967 | minus | intergenic | ccp-miR319a-1-3p | UUGGACUGAAGGGAGCUCCCC | 211-231 | 21 |
| ccp-miR319a-2 | chr11 | 26328965 | 26329154 | minus | genic | ccp-miR319a-2-3p | CUUGGACUGAAGGGAGCUCC | 171-190 | 20 |
|  |  |  |  |  |  | ccp-miR319a-2-5p | GAGCUCUCUCCAGUCCAGUC | 3-22 | 22 |
| ccp-miR319a-3 | chr8 | 30318341 | 30318522 | minus | intergenic | ccp-miR319a-3-3p | UUGGACUGAAGGGAGCUCCCU | 154-174 | 21 |
|  |  |  |  |  |  | ccp-miR319a-3-5p | AGAGCUUUCUUCAGUCCACUC | 14-34 | 21 |
| ccp-miR319c-1 | chr4 | 5038386 | 5038578 | minus | intergenic | ccp-miR319c-1-3p | UUGGACUGAAGGGUUUCCUUC | 164-184 | 21 |
|  |  |  |  |  |  | ccp-miR319c-1-5p | AUCCAAUGAUGCAGGAGCCGG | 120-140 | 21 |
| ccp-miR319c-2 | chr2 | 4029379 | 4029551 | minus | intergenic | ccp-miR319c-2-3p | CUUGGACUGAAGGGAGCUCCC | 153-173 | 21 |
|  |  |  |  |  |  | ccp-miR319c-2-5p | AGAGCUUCCUUCAGCCCACUC | 4-24 | 21 |
| ccp-miR3439 | chr4 | 22473611 | 22473923 | minus | genic | ccp-miR3439-5p | UUGGGGUUUGGAAAUCAAGUU | 68-88 | 21 |
| ccp-miR3627-1 | chr7 | 1958192 | 1958320 | plus | intergenic | ccp-miR3627-1-5p | UUGUCGCAGGAGAUAUGGCACU | 24-45 | 22 |
| ccp-miR3627-2 | chr7 | 1961562 | 1961715 | minus | intergenic | ccp-miR3627-2-3p | GGUGCCAUUCCUCCUGCGACAC | 114-135 | 22 |
|  |  |  |  |  |  | ccp-miR3627-2-5p | UGUCGCAGGAGCAAUGGCGCUU | 23-44 | 22 |
| ccp-miR390 | chr1 | 32829582 | 32829745 | plus | intergenic | ccp-miR390-3p | CGCUAUCCAUCCUGAGUUUUA | 126-146 | 21 |
|  |  |  |  |  |  | ccp-miR390-5p | AAGCUCAGGAGGGAUAGCGCC | 22-42 | 21 |
| ccp-miR390a-1 | chr1 | 32829582 | 32829745 | minus | genic | ccp-miR390a-1-3p | CGCUAUCCCUCCUGAGCUUUA | 125-145 | 21 |
|  |  |  |  |  |  | ccp-miR390a-1-5p | AAACUCAGGAUGGAUAGCGCC | 21-41 | 21 |
| ccp-miR390a-2 | chr6 | 718637 | 718813 | plus | intergenic | ccp-miR390a-2-3p | CGCUAUCCCUCCUGAGCUUCA | 139-159 | 21 |
|  |  |  |  |  |  | ccp-miR390a-2-5p | AAGCUCAGGAUGGAUAGCGCU | 21-41 | 21 |
| ccp-miR393a | chr2 | 5587560 | 5587699 | minus | intergenic | ccp-miR393a-3p | AUCAUGCUAUCCUUUUGGAUA | 114-134 | 21 |
|  |  |  |  |  |  | ccp-miR393a-5p | CAAAGGGAUCGCAUUGAUCCU | 1-21 | 21 |
| ccp-miR393b | chr2 | 5587549 | 5587720 | plus | intergenic | ccp-miR393b-3p | UCAAUGCGAUCCCUUUGGAUG | 135-155 | 21 |
| ccp-miR394 | chr10 | 765119 | 765281 | plus | intergenic | ccp-miR394-3p | AGGUGGGCAUACUGCCAACU | 119-138 | 20 |
|  |  |  |  |  |  | ccp-miR394-5p | UUGGCAUUCUGUCCACCUCCAU | 27-48 | 22 |
| ccp-miR394a | chr0 | 23552492 | 23552573 | plus | intergenic | ccp-miR394a-5p | UUGGCAUUCUGUCGACCUUCAU | 12-33 | 22 |
| ccp-miR395a-1 | chr11 | 30226771 | 30226861 | minus | genic | ccp-miR395a-1-3p | CUGAAGUGUUUGGGGGAACUCC | 62-83 | 22 |
| ccp-miR395a-2 | chr0 | 187220918 | 187221050 | minus | intergenic | ccp-miR395a-2-3p | CUGAAGUGUUUGGGGGAACUCC | 95-116 | 22 |
| ccp-miR395a-3 | chr11 | 30224784 | 30224875 | minus | genic | ccp-miR395a-3-3p | CUGAAGUGUUUGGGGGAACUCC | 63-84 | 22 |
| ccp-miR395b-1 | chr6 | 21798046 | 21798164 | plus | intergenic | ccp-miR395b-1-3p | GUGAAGUGUUUGGGGGAACUC | 78-98 | 21 |
| ccp-miR395b-2 | chr9 | 20573205 | 20573336 | minus | intergenic | ccp-miR395b-2-3p | GUGAAGUGUUCGAGGGAACUC | 94-114 | 21 |
| ccp-miR395h | chr2 | 50455485 | 50455800 | plus | intergenic | ccp-miR395h-5p | AUGAAGUGUUUUGGAGUACUU | 115-135 | 21 |
| ccp-miR395t-1 | chr1 | 15000177 | 15000490 | plus | intergenic | ccp-miR395t-1-3p | GUGAAGUGUUUUGGGAAGCUA | 174-194 | 21 |
| ccp-miR395t-2 | chr6 | 28451037 | 28451274 | minus | intergenic | ccp-miR395t-2-3p | GUGAAGUGUUUGGGGAAACUA | 106-126 | 21 |
| ccp-miR395x | chr9 | 18880070 | 18880316 | minus | genic | ccp-miR395x-5p | GUGAAGUGUUUGGAGAAGCUA | 81-101 | 21 |
| ccp-miR396-1 | chr6 | 6513958 | 6514096 | plus | intergenic | ccp-miR396-1-3p | GUUCAAGAAAGCUGUGGAAAA | 111-131 | 21 |
|  |  |  |  |  |  | ccp-miR396-1-5p | UCCCACAGCUUCCUUGAACUU | 11-31 | 21 |
| ccp-miR396-2 | chr4 | 3379786 | 3379888 | minus | intergenic | ccp-miR3962-2-5p | AGCUUUCUUGAGCUUCUUGAUUAGC | 19-44 | 25 |
|  |  |  |  |  |  | ccp-miR396-2-3p | GUUCAAGAAAGCUGUGGAAAA | 73-93 | 21 |
| ccp-miR396a | chr6 | 6505948 | 6506102 | plus | intergenic | ccp-miR396a-3p | GUUCAAUAAAGCUGUGGGAUG | 120-140 | 21 |
|  |  |  |  |  |  | ccp-miR396a-5p | UUCCACAGCUUUCUUGAACUG | 31-51 | 21 |
| ccp-miR396b-1 | chr4 | 3379786 | 3379888 | plus | genic | ccp-miR396b-1-3p | GCUCAAGAAAGCUGCGGGAAA | 73-93 | 21 |
|  |  |  |  |  |  | ccp-miR396b-1-5p | UUCCACAGCUUUCUUGAACUU | 13-33 | 21 |
| ccp-miR396b-2 | chr6 | 6513962 | 6514109 | minus | intergenic | ccp-miR396b-2-5p | UUCCACAGCUUUCUUGAACUU | 24-44 | 21 |
| ccp-miR396c | chr10 | 6647260 | 6647465 | plus | genic | ccp-miR396c-3p | UUCCACAGGCUUUCUUGAACGA | 156-177 | 22 |
| ccp-miR397a-1 | chr5 | 26533034 | 26533140 | minus | genic | ccp-miR397a-1-3p | UCUGCGCUGCACCCAAUCAUG | 72-92 | 21 |
|  |  |  |  |  |  | ccp-miR397a-1-5p | UCAUUGAGUGCAGCGUUGAUG | 10-30 | 21 |
| ccp-miR397a-3 | chr5 | 26533041 | 26533141 | plus | intergenic | ccp-miR397a-3-3p | CAUCAACGCUGCACUCAAUGA | 71-91 | 21 |
|  |  |  |  |  |  | ccp-miR397a-3-5p | AUUGGGUGCAGCGCAGAUGAA | 13-33 | 21 |
| ccp-miR398 | chr2 | 34102348 | 34102475 | minus | intergenic | ccp-miR398-3p | UGUGUUCUCAUGUUGCCCCUG | 90-100 | 21 |
|  |  |  |  |  |  | ccp-miR398-5p | GGGGCGACCUGAGAACACAUU | 21-41 | 21 |
| ccp-miR398a | chr11 | 29416868 | 29417016 | plus | genic | ccp-miR398a-3p | UGUGUUCUCAGGUCACCCCUU | 108-128 | 21 |
| ccp-miR398b | chr2 | 34102348 | 34102475 | plus | intergenic | ccp-miR398b-3p | UGUGUUCUCAGGUCGCCCCUG | 90-110 | 21 |
|  |  |  |  |  |  | ccp-miR398b-5p | GGGGCAACAUGAGAACACAUA | 21-41 | 21 |
| ccp-miR399-1 | chr2 | 13906321 | 13906411 | plus | genic | ccp-miR399-1-3p | UGCCAAAGGAGAAUUGCUCUG | 61-81 | 21 |
|  |  |  |  |  |  | ccp-miR399-1-5p | CAUAGCGAUUCUCCUUUGUCA | 10-30 | 21 |
| ccp-miR399-2 | chr2 | 13865584 | 13865687 | plus | intergenic | ccp-miR399-2-3p | UGCCAAAGGAGAAUUGCCCUG | 75-95 | 21 |
|  |  |  |  |  |  | ccp-miR399-2-5p | GUGUGAUUCUCCUUUGGCAUG | 12-32 | 21 |
| ccp-miR399-3 | chr2 | 13915821 | 13915936 | plus | genic | ccp-miR399-3-3p | UGCCAAAGGAGAAUUGCCCUG | 89-109 | 21 |
|  |  |  |  |  |  | ccp-miR399-3-5p | CAGGGCCGUUCUCCUUUGGCA | 27-47 | 21 |
| ccp-miR399-4 | chr2 | 13918946 | 13919084 | minus | intergenic | ccp-miR399-4-3p | UGCCAAAGGAGAUUUGCCCCG | 102-122 | 21 |
|  |  |  |  |  |  | ccp-miR399-4-5p | GGCAAAUACUCCUUUGGCAGGC | 21-42 | 22 |
| ccp-miR399-5 | chr2 | 13865584 | 13865687 | minus | intergenic | ccp-miR399-5-3p | UGCCAAAGGAGAAUCACACUG | 75-95 | 21 |
|  |  |  |  |  |  | ccp-miR399-5-5p | CAGGGCAAUUCUCCUUUGGCA | 10-30 | 21 |
| ccp-miR399-6 | chr2 | 13889186 | 13889326 | minus | intergenic | ccp-miR399-6-3p | UGCCAAAGGAGAUUUGUCCCG | 109-129 | 21 |
|  |  |  |  |  |  | ccp-miR399-6-5p | GGGCAACUAGUCCUCUGGCAGA | 15-36 | 22 |
| ccp-miR399a | chr2 | 13915825 | 13915955 | minus | genic | ccp-miR399a-3p | UGCCAAAGGAGAACGGCCCUG | 89-109 | 21 |
| ccp-miR399f | chr2 | 13862017 | 13862175 | minus | intergenic | ccp-miR399f-3p | UGCCAAAGGAGAUUUGCCCGG | 126-146 | 21 |
|  |  |  |  |  |  | ccp-miR399f-5p | GGGCAACUACUCCUUUGGCAG | 16-36 | 21 |
| ccp-miR399j | chr8 | 28925034 | 28925174 | minus | intergenic | ccp-miR399j-3p | UGCCAAAGGAGAGUUGCCCUA | 97-117 | 21 |
| ccp-miR403 | chr1 | 24017140 | 24017249 | minus | intergenic | ccp-miR403-3p | UUAGAUUCACGCACAAACUCG | 78-98 | 21 |
|  |  |  |  |  |  | ccp-miR403-5p | GUUUGUGCGUGAAUCUAACGC | 8-28 | 21 |
| ccp-miR403a | chr1 | 24017142 | 24017255 | plus | intergenic | ccp-miR403a-3p | UUAGAUUCACGCACAAACCUG | 84-104 | 21 |
|  |  |  |  |  |  | ccp-miR403a-5p | GUUUGUGCGUGAAUCUAACGC | 14-34 | 21 |
| ccp-miR408 | chr2 | 49819163 | 49819329 | plus | intergenic | ccp-miR408-3p | UGCUCUGCCUCGUCCCCGUCU | 115-135 | 21 |
|  |  |  |  |  |  | ccp-miR408-5p | CAGGGAAGAGGCAGUGCACGG | 35-55 | 21 |
| ccp-miR408b | chr11 | 193544 | 193636 | plus | intergenic | ccp-miR408b-3p | UGCUUUUCCCUUUUCCCUCUC | 65-85 | 21 |
|  |  |  |  |  |  | ccp-miR408b-5p | ACAGGGAAGAGGAACAGCAUA | 11-31 | 21 |
| ccp-miR4245 | chr9 | 2118156 | 2118329 | minus | intergenic | ccp-miR4245-5p | ACAAAGUUUGAUCCUGACAAG | 24-44 | 21 |
| ccp-miR4246 | chr3 | 21136407 | 21136672 | minus | intergenic | ccp-miR4246-5p | AAAUCCAAUUUUGAUUGUUUAA | 103-124 | 22 |
| ccp-miR426-1 | chr8 | 26099157 | 26099494 | minus | intergenic | ccp-miR426-1-5p | UUUUGGAAAUUUGUAAUUACU | 43-65 | 21 |
| ccp-miR426-2 | chr6 | 24893239 | 24893503 | minus | intergenic | ccp-miR426-2-5p | UUUUGGAAAUUUCUCCAUACA | 45-65 | 21 |
| ccp-miR4342-1 | chr10 | 16830303 | 16830568 | plus | intergenic | ccp-miR4342-1-3p | AAUCGACUUAGAAUGCUUAAUGGU | 129-152 | 24 |
| ccp-miR4342-2 | chr11 | 14213037 | 14213279 | minus | genic | ccp-miR4342-2-5p | AAUCGACUUAGAAUGCUUAAUGGU | 17-40 | 24 |
| ccp-miR477i-1 | chr8 | 30572428 | 30572572 | plus | intergenic | ccp-miR477i-1-5p | ACUCUCCCUCAAGGGCUUCCC | 21-41 | 21 |
| ccp-miR477i-2 | chr8 | 30572644 | 30572749 | plus | intergenic | ccp-miR477i-2-3p | GAGGCGCUUGGGGAGAGUGGA | 70-90 | 21 |
|  |  |  |  |  |  | ccp-miR477i-2-5p | ACUCUCCCUCAAGGGCUUCCG | 18-38 | 21 |
| ccp-miR482a-1 | chr10 | 8614558 | 8614677 | plus | intergenic | ccp-miR482a-1-3p | GGAAUUGGUGGAUUGUCAAGC | 70-90 | 21 |
| ccp-miR482a-2 | chr11 | 26816042 | 26816187 | minus | genic | ccp-miR482a-2-3p | UUUCCUAGCCCGCCCAUUCCUA | 86-107 | 22 |
|  |  |  |  |  |  | ccp-miR482a-2-5p | GAAGUUUUGGGAAUGGGCUGCU | 34-55 | 22 |
| ccp-miR482a-3 | chr11 | 26964580 | 26964757 | minus | intergenic | ccp-miR482a-3-3p | UCUUCCCUAGUCCUCCCAUCCC | 115-136 | 22 |
|  |  |  |  |  |  | ccp-miR482a-3-5p | GAUGGGUGACCGGGGAAGAUUU | 46-67 | 22 |
| ccp-miR482a-4 | chr11 | 26897301 | 26897477 | minus | genic | ccp-miR482a-4-3p | UCUUCCCUAGUCCUCCCAUCCC | 114-135 | 22 |
|  |  |  |  |  |  | ccp-miR482a-4-5p | GAUGGGUGACUCGGGCAGAUUU | 46-67 | 22 |
| ccp-miR482a-5 | chr11 | 26937225 | 26937400 | minus | genic | ccp-miR482a-5-3p | UCUUCCCUAGUCCUCCCAUCCC | 113-134 | 22 |
|  |  |  |  |  |  | ccp-miR482a-5-5p | GAUGGGUGACUGGGGAAGGUUU | 46-67 | 22 |
| ccp-miR482b-1 | chr11 | 26937500 | 26937668 | plus | genic | ccp-miR482b-1-3p | CUUCCCAAACCUCCCAUUUCCU | 113-134 | 22 |
|  |  |  |  |  |  | ccp-miR482b-1-5p | GGAAUGGGAGGACUGGGAAAG | 36-56 | 21 |
| ccp-miR482b-2 | chr11 | 26964874 | 26965040 | plus | intergenic | ccp-miR482b-2-5p | GGAAUGGGAGGACUGGGAAAG | 33-53 | 21 |
| ccp-miR5013 | chr4 | 7692627 | 7692906 | minus | intergenic | ccp-miR5013-5p | UUUGUGACAUAAAGGUGCUUC | 64-84 | 21 |
| ccp-miR5014a | chr6 | 3917121 | 3917372 | plus | intergenic | ccp-miR5014a-5p | UUGUACAAAUUUAAUUGUAUA | 110-130 | 21 |
| ccp-miR5020a | chr2 | 38978268 | 38978511 | plus | intergenic | ccp-miR5020a-3p | UGGAAGAAUGUAAGACUUGCC | 114-134 | 21 |
| ccp-miR5042-1 | chr1 | 18039557 | 18039628 | plus | intergenic | ccp-miR5042-1-3p | UGGGGCUUGGUCGAAGAUAGU | 33-53 | 21 |
| ccp-miR5042-2 | chr6 | 26783087 | 26783165 | plus | intergenic | ccp-miR5042-2-3p | UGGGGCUUGGUCGAAGAUAGUU | 37-58 | 22 |
| ccp-miR5167b | chr7 | 4426133 | 4426308 | minus | intergenic | ccp-miR5167b-5p | UCUAGUUAAAGUAAUUCAACA | 45-65 | 21 |
| ccp-miR5210 | chr7 | 11624936 | 11625151 | minus | intergenic | ccp-miR5210-5p | UAAAUGUGAUGGAAUUAGGGGU | 5-26 | 22 |
| ccp-miR5272f | chr5 | 22181079 | 22181171 | plus | intergenic | ccp-miR5272f-3p | GAAUUGAUUCUGUUUGGAGACAUU | 36-59 | 24 |
| ccp-miR530 | chr6 | 3344235 | 3344462 | plus | intergenic | ccp-miR530-3p | AGGUGCAGAUGCUGAUGCAG | 199-218 | 20 |
|  |  |  |  |  |  | ccp-miR530-5p | UCUGCAUUUGCACCUGCACCU | 9-29 | 21 |
| ccp-miR5368 | chr1 | 10327108 | 10327278 | plus | intergenic | ccp-miR5368-3p | GGACAGUCUCAGGUAGACA | 151-169 | 19 |
| ccp-miR5640 | chr11 | 31464279 | 31464366 | plus | genic | ccp-miR5640-5p | UGAGAGAAGGAAGUAGACUCU | 60-80 | 21 |
| ccp-miR5648 | chr5 | 2541956 | 2542185 | plus | intergenic | ccp-miR5648-5p | UUCUGAAAUGUUUGGCUUUGCUUU | 25-48 | 22 |
| ccp-miR5653-1 | chr6 | 3759190 | 3759360 | minus | intergenic | ccp-miR5653-1-3p | UGGGUUGAGUUGGAUUGACUUGGC | 120-143 | 24 |
| ccp-miR5653-2 | chr5 | 24535377 | 24535521 | plus | genic | ccp-miR5653-2-3p | UGGGUUGAGUUGAGUUGAGUUGAG | 115-138 | 24 |
| ccp-miR5658 | chr9 | 185793 | 186063 | minus | intergenic | ccp-miR5658-3p | AUGAUGAUGAUGAUGAUGAGC | 247-267 | 21 |
| ccp-miR5741a-1 | chr10 | 23557873 | 23558082 | plus | intergenic | ccp-miR5741a-1-3p | UAGGGACUAAAUUGAUGAAAA | 179-199 | 21 |
| ccp-miR5741a-2 | chr6 | 35517293 | 35517503 | minus | genic | ccp-miR5741a-2-3p | UAGGGACUAAACUGAUAGUUG | 142-162 | 21 |
| ccp-miR5741a-1 | chr6 | 7104724 | 7104916 | plus | intergenic | ccp-miR5741a-3p | UAGGGACUAAACUGAUAGUUG | 157-177 | 21 |
| ccp-miR5780 | chr8 | 24603552 | 24603840 | plus | intergenic | ccp-miR5780-3p | UGUUUUGAGUGUUUGAUAAAUG | 177-198 | 22 |
| ccp-miR5780d-3 | chr3 | 2848962 | 2849102 | plus | intergenic | ccp-miR5780d-3-3p | UGUUUUGAGUGUUUGAUAAAUG | 85-106 | 22 |
| ccp-miR5780d-4 | chr2 | 24973474 | 24973633 | minus | intergenic | ccp-miR5780d-4-3p | UGUUUUGAGUGUUUGAUAAAUG | 95-116 | 22 |
| ccp-miR6142 | chr7 | 28896026 | 28896247 | plus | intergenic | ccp-miR6142-5p | GACGAUUUUUUAAGAUAUGACUAC | 10-33 | 24 |
| ccp-miR6188 | chr4 | 2193386 | 2193555 | minus | intergenic | ccp-miR6188-3p | GGUGGAUCAAUGAACCCAGCUC | 113-134 | 22 |
| ccp-miR6198 | chr11 | 13126325 | 13126524 | minus | intergenic | ccp-miR6198-5p | GCUCUGUCUUGGCUGGACAUGC | 79-100 | 22 |
| ccp-miR6253 | chr6 | 17942233 | 17942340 | plus | intergenic | ccp-miR6253-3p | GAGGAAAGUGGAGUGUUGGGUU | 47-68 | 22 |
| ccp-miR6281 | chr7 | 1859587 | 1859749 | minus | intergenic | ccp-miR6281-3p | GUUAGAGAGAGAGAGAGAGAG | 101-121 | 21 |
| ccp-miR6440b | chr8 | 31436307 | 31436626 | minus | intergenic | ccp-miR6440b-5p | GAGUUUGACCGAAUUCGAGUC | 125-145 | 21 |
| ccp-miR6459a-2 | chr7 | 3634303 | 3634422 | minus | intergenic | ccp-miR6459a-2-5p | AGCUCAAGCUCAAAUUCGAUU | 38-58 | 21 |
| ccp-miR6459a-3 | chr11 | 26934027 | 26934195 | plus | intergenic | ccp-miR6459a-3-5p | AGCUCAAGCUCAAACUCGAGA | 4-24 | 21 |
| ccp-miR6459a-4 | chr11 | 26934027 | 26934195 | minus | intergenic | ccp-miR6459a-4-3p | UCGAGUUUGAGCUUGAGCUUU | 148-168 | 21 |
| ccp-miR6462c | chr2 | 13290869 | 13290972 | minus | intergenic | ccp-miR6462c-5p | AAGGGACAAAAAUGGAGGAAGA | 51-72 | 22 |
| ccp-miR6476a | chr1 | 30315085 | 30315167 | plus | intergenic | ccp-miR6476a-3p | UCAGUGGAGAUGAAACAUUU | 50-69 | 20 |
| ccp-miR7122a | chr3 | 3950532 | 3950687 | minus | intergenic | ccp-miR7122a-3p | CCGCGUUUCUUUGUAUGAAGA | 118-138 | 21 |
|  |  |  |  |  |  | ccp-miR7122a-5p | UUAUACAGAGAAACCGCGGUUG | 22-43 | 22 |
| ccp-miR7494b | chr1 | 7524504 | 7524684 | plus | intergenic | ccp-miR7494b-3p | AGAGGGAGAGGAAGAAGAGAAAA | 149-171 | 23 |
| ccp-miR7502f | chr4 | 18174388 | 18174715 | minus | intergenic | ccp-miR7502f-3p | UUUAGCAGUAGAGAUGGAUGG | 193-213 | 21 |
| ccp-miR7504a | chr9 | 18992778 | 18992935 | minus | intergenic | ccp-miR7504a-3p | UUUGUUUUUGUCCAAAAUUUCA | 137-158 | 22 |
| ccp-miR7504b | chr9 | 11415604 | 11415806 | minus | intergenic | ccp-miR7504b-5p | AGGAGGAAAAAUGCCAUUAGUCAU | 71-94 | 24 |
| ccp-miR7530 | chr7 | 2329176 | 2329311 | plus | intergenic | ccp-miR7530-5p | CCUUCCUCUCUUCUCCCUCUUC | 10-31 | 22 |
| ccp-miR7696c | chr3 | 3355498 | 3355712 | plus | intergenic | ccp-miR7696c-3p | UUUUGAAUUAUGCGAAUUUGG | 101-121 | 21 |
| ccp-miR7743 | chr11 | 9606014 | 9606137 | plus | intergenic | ccp-miR7743-5p | UUUGAACUUUUGUAAUGGUUCUCA | 1-24 | 24 |
| ccp-miR7822 | chr2 | 28308641 | 28308971 | minus | intergenic | ccp-miR7822-3p | UUUGAAAUUGAAUAAGUGGUG | 269-289 | 21 |
| ccp-miR7828 | chr10 | 6139145 | 6139446 | plus | genic | ccp-miR7828-3p | GAUGACAUGGGCAUCAAAAUG | 194-214 | 21 |
| ccp-miR7982a-1 | chr0 | 184187607 | 184187867 | plus | intergenic | ccp-miR7982a-1-5p | AAGUUGGAUGCUUAUGAUAUAUAU | 91-114 | 24 |
| ccp-miR7982a-2 | chr11 | 14325725 | 14325974 | minus | intergenic | ccp-miR7982a-2-5p | AAGUUGGAUGCUUAUGAUAUAUAU | 91-114 | 24 |
| ccp-miR7997c | chr4 | 22390024 | 22390132 | minus | genic | ccp-miR7997c-3p | AUAUUGCUCUGAUUCCCCAAAAAU | 54-77 | 24 |
| ccp-miR8001b | chr9 | 2865688 | 2865962 | minus | intergenic | ccp-miR8001b-5p | GGAUUUUCAAACUACUCCCUACAA | 68-91 | 24 |
| ccp-miR8044 | chr1 | 7998394 | 7998586 | plus | genic | ccp-miR8044-5p | UUUCAAAUAUCUUUGGAGAUU | 152-172 | 21 |
| ccp-miR821b | chr6 | 33303831 | 33304093 | plus | intergenic | ccp-miR821b-5p | AAGUUAUGAACAUAAAAAAUG | 15-35 | 21 |
| ccp-miR827 | chr7 | 3728493 | 3728816 | minus | intergenic | ccp-miR827-5p | UUUUGUUGGUGAUCAUCUAAGG | 134-155 | 22 |
| ccp-miR828a | chr2 | 54112346 | 54112487 | plus | intergenic | ccp-miR828a-3p | AGAUACUCAUUUGAACAAGAUG | 117-138 | 22 |
|  |  |  |  |  |  | ccp-miR828a-5p | UCUUGCUCAAAUGAGUAUUCCA | 7-28 | 22 |
| ccp-miR837 | chr1 | 28708356 | 28708624 | plus | intergenic | ccp-miR837-5p | CAUUGUUUCUUGUUUUUUUAA | 119-139 | 21 |
| ccp-miR845 | chr1 | 27960912 | 27960984 | minus | intergenic | ccp-miR845-3p | UGCUCUGAUACCACUUGUUGG | 42-62 | 21 |
| ccp-miR845b | chr5 | 13132719 | 13132858 | minus | genic | ccp-miR845b-5p | UCAAUUGGUAUCAGAGCUUGG | 64-84 | 21 |
| ccp-miR856 | chr7 | 1567108 | 1567287 | plus | intergenic | ccp-miR856-3p | UGAUGUUAUCUGUGGUACAAA | 79-99 | 21 |
| ccp-miR8578 | chr6 | 25402478 | 25402755 | plus | intergenic | ccp-miR8578-5p | UUUAUGAGAGAUCUUUCCAAC | 10-30 | 21 |
| ccp-miR8691 | chr8 | 16383158 | 16383444 | minus | intergenic | ccp-miR8691-5p | AGAUGAUGAGAAGGGUAGGUCAAU | 78-101 | 24 |
| ccp-miR8709a | chr8 | 26524427 | 26524750 | minus | genic | ccp-miR8709a-3p | AUAUUAAAACUAAACAUGAACAAA | 292-315 | 24 |
| ccp-miR8746-1 | chr3 | 19975043 | 19975133 | minus | intergenic | ccp-miR8746-5p | UCCAUAUUUCACAAUCUCUCC | 4-24 | 21 |
| ccp-miR902a | chr1 | 1215198 | 1215365 | plus | intergenic | ccp-miR902a-3p | AUGAAGGUCAGCAUCAUAGC | 95-114 | 20 |
| ccp-miR9557 | chr1 | 19836896 | 19837036 | minus | intergenic | ccp-miR9557-3p | GCUGAGUUUGAACACAAAAUU | 84-104 | 21 |
| ccp-miR9559 | chr6 | 2025819 | 2025970 | minus | intergenic | ccp-miR9559-5p | UUUGGAUUUUGGUCAUUUAAG | 71-85 | 21 |
| ccp-miR9568 | chr10 | 26732996 | 26733129 | plus | intergenic | ccp-miR9568-3p | UCAUCGUAAGAGAUCCGAAUU | 103-123 | 21 |
| ccp-miR9752 | chr1 | 2816120 | 2816222 | plus | intergenic | ccp-miR9752-3p | UGCUUCUUCAUUUUCCUGUUA | 48-68 | 21 |
| ccp-miR9773 | chr0 | 41466328 | 41466541 | plus | intergenic | ccp-miR9773-5p | UUUGUUUUUAUGUUAUUUCGUACU | 48-71 | 24 |
