# Supplementary material for: A genome-wide analysis of the RNA-guided silencing pathway in coffee reveals insights into its regulatory mechanisms
Source: PLoS One. 2017 Apr 27;12(4):e0176333. doi: 10.1371/journal.pone.0176333 (PMC5407642; doi:10.1371/journal.pone.0176333)
Supplement: S3 Table — Minimal Free Energy (MFE), adjusted MFE (AMFE), MFE index (MFEI), Minimal Free Energy of the thermodynamic ensemble (MFEE), Ensemble Diversity (Diversity), and frequency of the MFE structure in the ensemble (Frequency). (DOCX) [file pone.0176333.s006.docx]

| pre-miRNAs | Lenght | GC (%) | AU (%) | UA-ratio | GC-ratio | MFE | MFE-ensemble | Frequency | Diversity | AMFE | MFEI |
| --- | --- | --- | --- | --- | --- | --- | --- | --- | --- | --- | --- |
| ccp-miR1023e | 335 | 30,14925 | 69,85075 | 1,25 | 0,7413793 | -72,8 | -54,77 | 1,13E-05 | 82,89 | -21,7313 | -0,72079 |
| ccp-miR1030i | 302 | 38,74172 | 61,25828 | 0,7961165 | 1,8536585 | -89,8 | -76,4 | 8,04E-05 | 50,75 | -29,7351 | -0,76752 |
| ccp-miR1060 | 108 | 33,33333 | 66,66667 | 0,9459459 | 1,25 | -27,8 | -27,7 | 0,2369 | 3,21 | -25,7407 | -0,77222 |
| ccp-miR1078 | 106 | 33,96226 | 66,03774 | 1,5925926 | 2 | -28,4 | -27,1 | 0,0513727 | 9,8 | -26,7925 | -0,78889 |
| ccp-miR1089 | 131 | 32,82443 | 67,17557 | 1,2 | 1,047619 | -31,8 | -23,55 | 0,01537 | 35,78 | -24,2748 | -0,73953 |
| ccp-miR1127-1 | 139 | 41,00719 | 58,99281 | 1,05 | 1,1111111 | -68,8 | -66,6 | 0,0701582 | 10,2 | -49,4964 | -1,20702 |
| ccp-miR1127-2 | 283 | 32,86219 | 67,13781 | 1,0212766 | 1,1136364 | -77,4 | -62,49 | 0,00051328 | 53,09 | -27,3498 | -0,83226 |
| ccp-miR1128-1 | 283 | 31,09541 | 68,90459 | 1,1195652 | 0,7959184 | -87,7 | -86,1 | 0,00038127 | 43,49 | -30,9894 | -0,99659 |
| ccp-miR1128-2 | 282 | 36,52482 | 63,47518 | 1,2658228 | 0,9074074 | -91 | -79,31 | 0,00011088 | 42,82 | -32,2695 | -0,8835 |
| ccp-miR1128-3 | 123 | 35,77236 | 64,22764 | 0,8809524 | 1,3157895 | -35,3 | -32,3 | 0,0404975 | 14,31 | -28,6992 | -0,80227 |
| ccp-miR1128-4 | 282 | 34,39716 | 65,60284 | 1,2560976 | 1,3095238 | -75,2 | -67,05 | 9,03E-05 | 71,17 | -26,6667 | -0,77526 |
| ccp-miR1435 | 108 | 35,18519 | 64,81481 | 1,2580645 | 0,9 | -48,9 | -45,75 | 0,0369354 | 15,06 | -45,2778 | -1,28684 |
| ccp-miR1438-1 | 269 | 30,85502 | 69,14498 | 0,9787234 | 1,9642857 | -87,2 | -65,5 | 0,00056018 | 59,41 | -32,4164 | -1,0506 |
| ccp-miR1438-2 | 231 | 30,30303 | 69,69697 | 0,9166667 | 2,1818182 | -56,6 | -43,6 | 0,00044202 | 50,84 | -24,5022 | -0,80857 |
| ccp-miR1438-3 | 195 | 32,30769 | 67,69231 | 1,1290323 | 1,3333333 | -47,5 | -46,3 | 0,00830975 | 16,75 | -24,359 | -0,75397 |
| ccp-miR1446 | 130 | 46,15385 | 53,84615 | 1,2580645 | 0,9354839 | -55,2 | -48,7 | 0,0114688 | 18,95 | -42,4615 | -0,92 |
| ccp-miR1510b | 68 | 41,17647 | 58,82353 | 1,1052632 | 0,6470588 | -26,1 | -26,1 | 0,247921 | 3,67 | -38,3824 | -0,93214 |
| ccp-miR1515a | 119 | 47,89916 | 52,10084 | 1,0666667 | 0,8387097 | -40,2 | -39,3 | 0,0201383 | 14,13 | -33,7815 | -0,70526 |
| ccp-miR1520e | 287 | 30,31359 | 69,68641 | 1,173913 | 1,1219512 | -77,4 | -75,6 | 0,00064402 | 19,33 | -26,9686 | -0,88966 |
| ccp-miR1526 | 118 | 32,20339 | 67,79661 | 1 | 1,5333333 | -29,3 | -26,3 | 0,0614738 | 15,48 | -24,8305 | -0,77105 |
| ccp-miR156 | 102 | 49,01961 | 50,98039 | 1,6 | 1,0833333 | -55,3 | -55,3 | 0,305147 | 3,68 | -54,2157 | -1,106 |
| ccp-miR156f | 87 | 47,12644 | 52,87356 | 1,3 | 1,05 | -45,1 | -42,1 | 0,220467 | 4,02 | -51,8391 | -1,1 |
| ccp-miR156g | 92 | 47,82609 | 52,17391 | 1 | 1 | -47 | -47 | 0,331285 | 5,33 | -51,087 | -1,06818 |
| ccp-miR157 | 84 | 44,04762 | 55,95238 | 1,2380952 | 1,0555556 | -44,6 | -43,1 | 0,220077 | 3,07 | -53,0952 | -1,20541 |
| ccp-miR157a-1 | 102 | 44,11765 | 55,88235 | 1,1923077 | 1,0454545 | -46 | -45 | 0,0735815 | 5,37 | -45,098 | -1,02222 |
| ccp-miR157a-2 | 85 | 41,17647 | 58,82353 | 1,0833333 | 1,0588235 | -38,1 | -38,1 | 0,254522 | 2,3 | -44,8235 | -1,08857 |
| ccp-miR159a | 188 | 47,87234 | 52,12766 | 1,45 | 1,3684211 | -87,5 | -84,5 | 0,00770099 | 14,96 | -46,5426 | -0,97222 |
| ccp-miR159b | 80 | 56,25 | 43,75 | 0,75 | 1,6470588 | -31,5 | -31,5 | 0,249483 | 3,69 | -39,375 | -0,7 |
| ccp-miR160 | 84 | 54,7619 | 45,2381 | 0,9 | 1 | -47,1 | -46,8 | 0,126088 | 3,97 | -56,0714 | -1,02391 |
| ccp-miR160a-1 | 98 | 48,97959 | 51,02041 | 1,173913 | 1 | -52,9 | -52,9 | 0,1335 | 5,41 | -53,9796 | -1,10208 |
| ccp-miR160a-2 | 84 | 48,80952 | 51,19048 | 1,15 | 1,05 | -44,1 | -44,1 | 0,119629 | 5,36 | -52,5 | -1,07561 |
| ccp-miR160h | 90 | 58,88889 | 41,11111 | 1,1764706 | 1,2083333 | -42,8 | -42,8 | 0,0642552 | 8,89 | -47,5556 | -0,80755 |
| ccp-miR162a | 175 | 42,85714 | 57,14286 | 0,7241379 | 1,2058824 | -54,38 | -54,38 | 0,0586866 | 10,07 | -31,0743 | -0,72507 |
| ccp-miR164-1 | 115 | 46,95652 | 53,04348 | 1,1034483 | 1 | -59,1 | -58,9 | 0,0343555 | 9,51 | -51,3913 | -1,09444 |
| ccp-miR164-2 | 100 | 51 | 49 | 0,96 | 0,9615385 | -52,4 | -52,4 | 0,111309 | 5,6 | -52,4 | -1,02745 |
| ccp-miR164-3 | 116 | 53,44828 | 46,55172 | 1,5714286 | 0,7714286 | -59,5 | -58,7 | 0,697419 | 1,47 | -51,2931 | -0,95968 |
| ccp-miR164a | 217 | 42,85714 | 57,14286 | 1,6956522 | 1,325 | -65,3 | -54,2 | 0,00704004 | 44,7 | -30,0922 | -0,70215 |
| ccp-miR164c | 116 | 53,44828 | 46,55172 | 0,6363636 | 1,2962963 | -52,8 | -52,7 | 0,112479 | 3,64 | -45,4955 | -0,95283 |
| ccp-miR166-1 | 161 | 42,85714 | 57,14286 | 1,7058824 | 0,9714286 | -61,5 | -61 | 0,0220494 | 15,76 | -38,1988 | -0,8913 |
| ccp-miR166-2 | 110 | 46,36364 | 53,63636 | 0,84375 | 0,9615385 | -52,2 | -49,9 | 0,251641 | 8,19 | -47,4545 | -1,02353 |
| ccp-miR166-3 | 179 | 43,01676 | 56,98324 | 1,55 | 0,7906977 | -56,9 | -53,4 | 0,0148008 | 20,25 | -31,7877 | -0,73896 |
| ccp-miR166-4 | 198 | 40,40404 | 59,59596 | 1,2264151 | 0,8604651 | -68,4 | -67,5 | 0,103022 | 22,54 | -34,5455 | -0,855 |
| ccp-miR166a-1 | 154 | 51,2987 | 48,7013 | 1,1428571 | 0,975 | -76,1 | -71,1 | 0,0427242 | 11,48 | -49,4156 | -0,96329 |
| ccp-miR166a-2 | 121 | 48,76033 | 51,23967 | 1,3846154 | 1,2692308 | -61,8 | -61,2 | 0,0886292 | 7,39 | -51,0744 | -1,04746 |
| ccp-miR166a-3 | 119 | 48,7395 | 51,2605 | 0,6944444 | 0,8125 | -50,2 | -41 | 0,0630602 | 9,12 | -42,1849 | -0,86552 |
| ccp-miR167-1 | 95 | 46,31579 | 53,68421 | 1,04 | 0,9130435 | -42,6 | -42,2 | 0,161074 | 3,89 | -44,8421 | -0,96818 |
| ccp-miR167-2 | 97 | 43,29897 | 56,70103 | 0,71875 | 0,9090909 | -38,2 | -33,8 | 0,0255014 | 11,94 | -39,3814 | -0,90952 |
| ccp-miR167-3 | 72 | 44,44444 | 55,55556 | 1,2222222 | 0,8823529 | -33,6 | -26,4 | 0,106814 | 9,49 | -46,6667 | -1,05 |
| ccp-miR167-4 | 336 | 35,11905 | 64,88095 | 1,1584158 | 1,0701754 | -96,4 | -82,87 | 3,46E-05 | 67,58 | -28,6905 | -0,81695 |
| ccp-miR167-5 | 88 | 46,59091 | 53,40909 | 0,7407407 | 0,7826087 | -31,7 | -31,7 | 0,517526 | 1,5 | -36,0227 | -0,77317 |
| ccp-miR167a | 79 | 45,56962 | 54,43038 | 1,3888889 | 0,8 | -47,1 | -47,1 | 0,797573 | 0,45 | -59,6203 | -1,30833 |
| ccp-miR167h | 111 | 47,74775 | 52,25225 | 0,7575758 | 1,0384615 | -50,5 | -50,5 | 0,332345 | 3,64 | -45,4955 | -0,95283 |
| ccp-miR168a | 146 | 47,94521 | 52,05479 | 1,4516129 | 1,1212121 | -59,7 | -58,4 | 0,0417161 | 10,23 | -40,8904 | -0,85286 |
| ccp-miR169-1 | 158 | 43,03797 | 56,96203 | 1,4324324 | 1,3448276 | -70 | -63,6 | 0,00405343 | 23,5 | -44,3038 | -1,02941 |
| ccp-miR169-2 | 117 | 45,29915 | 54,70085 | 1,2857143 | 1,3043478 | -43,3 | -40,9 | 0,0454064 | 17,69 | -37,0085 | -0,81698 |
| ccp-miR169-3 | 161 | 43,47826 | 56,52174 | 1,2195122 | 1,2580645 | -76,4 | -71,6 | 0,0433534 | 14,98 | -47,4534 | -1,09143 |
| ccp-miR169-4 | 112 | 42,85714 | 57,14286 | 1,2857143 | 1,5263158 | -34,2 | -32,8 | 0,0314168 | 18,36 | -30,5357 | -0,7125 |
| ccp-miR169a | 82 | 45,12195 | 54,87805 | 1,25 | 0,9473684 | -32,2 | -30,5 | 0,0434773 | 6,48 | -39,2683 | -0,87027 |
| ccp-miR169d | 183 | 42,0765 | 57,9235 | 1,4651163 | 1,0810811 | -76,2 | -70,9 | 0,0120702 | 20,28 | -41,6393 | -0,98961 |
| ccp-miR169e | 195 | 44,10256 | 55,89744 | 1,3191489 | 1,097561 | -81,5 | -80,1 | 0,0954384 | 18,2 | -41,7949 | -0,94767 |
| ccp-miR169f-1 | 215 | 43,25581 | 56,74419 | 1,1403509 | 0,9375 | -82,7 | -81,5 | 0,00518069 | 24,18 | -38,4651 | -0,88925 |
| ccp-miR169f-2 | 203 | 41,87192 | 58,12808 | 1,1454545 | 0,8478261 | -64,5 | -53,98 | 0,00178028 | 31,38 | -31,7734 | -0,75882 |
| ccp-miR169g | 184 | 50,54348 | 49,45652 | 1,3333333 | 1,0666667 | -88,7 | -86 | 0,00809692 | 13,59 | -48,2065 | -0,95376 |
| ccp-miR171-1 | 146 | 37,67123 | 62,32877 | 1,0222222 | 1,037037 | -51,6 | -51,3 | 0,0415191 | 10,98 | -35,3425 | -0,93818 |
| ccp-miR171-10 | 102 | 46,07843 | 53,92157 | 0,9642857 | 1,1363636 | -49,2 | -49,2 | 0,103072 | 2,96 | -48,2353 | -1,04681 |
| ccp-miR171-11 | 105 | 45,71429 | 54,28571 | 0,9655172 | 0,8461538 | -48,4 | -45,8 | 0,097688 | 5,09 | -46,0952 | -1,00833 |
| ccp-miR171-12 | 91 | 45,05495 | 54,94505 | 0,6666667 | 1,05 | -36,9 | -36,9 | 0,255717 | 2,6 | -40,5495 | -0,9 |
| ccp-miR171-13 | 93 | 45,16129 | 54,83871 | 0,8214286 | 0,68 | -31,6 | -30,9 | 0,246623 | 2,4 | -33,9785 | -0,75238 |
| ccp-miR171-2 | 146 | 37,67123 | 62,32877 | 0,9782609 | 0,9642857 | -43,7 | -41 | 0,0212712 | 16,04 | -29,9315 | -0,79455 |
| ccp-miR171-3 | 169 | 43,19527 | 56,80473 | 1,3414634 | 1,0277778 | -55,7 | -40,49 | 0,0110555 | 30,47 | -32,9586 | -0,76301 |
| ccp-miR171-4 | 91 | 45,05495 | 54,94505 | 1,5 | 0,952381 | -45,9 | -45,9 | 0,547441 | 1,61 | -50,4396 | -1,11951 |
| ccp-miR171-5 | 93 | 53,76344 | 46,23656 | 1,2631579 | 0,6129032 | -39,8 | -39,8 | 0,595525 | 1,29 | -42,7957 | -0,796 |
| ccp-miR171-6 | 97 | 52,57732 | 47,42268 | 0,7037037 | 1,6842105 | -39 | -38,8 | 0,0618017 | 2,76 | -40,2062 | -0,76471 |
| ccp-miR171-7 | 76 | 46,05263 | 53,94737 | 1,1578947 | 1,3333333 | -42,7 | -42,7 | 0,310547 | 1,47 | -56,1842 | -1,22 |
| ccp-miR171-8 | 78 | 44,87179 | 55,12821 | 1,2631579 | 1,0588235 | -37,7 | -36,8 | 0,410015 | 3,49 | -48,3333 | -1,07714 |
| ccp-miR171-9 | 90 | 41,11111 | 58,88889 | 1,5238095 | 1,1764706 | -41,7 | -40,8 | 0,375115 | 3,29 | -46,3333 | -1,12703 |
| ccp-miR171b-1 | 151 | 41,0596 | 58,9404 | 0,893617 | 0,9375 | -48,9 | -47,4 | 0,0690656 | 9,34 | -32,3841 | -0,78871 |
| ccp-miR171b-2 | 102 | 40,19608 | 59,80392 | 0,6486486 | 0,8636364 | -33,1 | -33,1 | 0,320379 | 3 | -32,451 | -0,80732 |
| ccp-miR171f | 156 | 49,35897 | 50,64103 | 0,7954545 | 1,2647059 | -62,7 | -57 | 0,00670043 | 23,22 | -40,1923 | -0,81429 |
| ccp-miR172-1 | 176 | 44,31818 | 55,68182 | 0,6896552 | 0,7333333 | -56,5 | -54,1 | 0,00942203 | 18,45 | -32,1023 | -0,72436 |
| ccp-miR172-2 | 108 | 41,66667 | 58,33333 | 1,1 | 1,25 | -55,9 | -55,5 | 0,141375 | 4,84 | -51,7593 | -1,24222 |
| ccp-miR172-3 | 96 | 39,58333 | 60,41667 | 0,9333333 | 0,8095238 | -37,1 | -37,1 | 0,0876695 | 7,71 | -38,6458 | -0,97632 |
| ccp-miR172d-1 | 176 | 44,31818 | 55,68182 | 1,45 | 1,3636364 | -68,9 | -54,87 | 0,00148701 | 20,87 | -39,1477 | -0,88333 |
| ccp-miR172d-2 | 163 | 38,65031 | 61,34969 | 1,173913 | 1,4230769 | -68,1 | -68,1 | 0,245095 | 9,01 | -41,7791 | -1,08095 |
| ccp-miR174e | 315 | 33,33333 | 66,66667 | 1,0192308 | 1,6923077 | -78,7 | -60,8 | 3,86E-05 | 69,73 | -24,9841 | -0,74952 |
| ccp-miR1863b-1 | 172 | 33,13953 | 66,86047 | 1,0175439 | 0,8387097 | -97,5 | -96,4 | 0,183207 | 13,49 | -56,686 | -1,71053 |
| ccp-miR1863b-2 | 83 | 40,96386 | 59,03614 | 0,8846154 | 1 | -48,9 | -48,9 | 0,855629 | 0,31 | -58,9157 | -1,43824 |
| ccp-miR1878 | 84 | 35,71429 | 64,28571 | 1,4545455 | 1,1428571 | -21,9 | -21,9 | 0,189724 | 7,1 | -26,0714 | -0,73 |
| ccp-miR1885a | 96 | 36,45833 | 63,54167 | 1,2592593 | 0,9444444 | -25,2 | -25,2 | 0,0883124 | 6,82 | -26,25 | -0,72 |
| ccp-miR1919 | 157 | 52,2293 | 47,7707 | 1,34375 | 0,952381 | -86,1 | -84 | 0,0189259 | 8,41 | -54,8408 | -1,05 |
| ccp-miR2105 | 186 | 36,55914 | 63,44086 | 1,5652174 | 1,5185185 | -48,3 | -23,18 | 0,00167606 | 55,64 | -25,9677 | -0,71029 |
| ccp-miR2111 | 77 | 38,96104 | 61,03896 | 1,4736842 | 1,3076923 | -34,4 | -33,5 | 0,0430841 | 7,1 | -44,6753 | -1,14667 |
| ccp-miR2275d-1 | 82 | 36,58537 | 63,41463 | 0,9259259 | 0,7647059 | -22 | -22 | 0,157031 | 6,75 | -26,8293 | -0,73333 |
| ccp-miR2275d-2 | 111 | 36,93694 | 63,06306 | 1,9166667 | 0,8636364 | -35,8 | -33,6 | 0,0840666 | 8,06 | -32,2523 | -0,87317 |
| ccp-miR2592-1 | 92 | 36,95652 | 63,04348 | 0,9333333 | 1,2666667 | -27,2 | -25,4 | 0,0895997 | 16,13 | -29,5652 | -0,8 |
| ccp-miR2592-2 | 283 | 43,4629 | 56,5371 | 1,0253165 | 1,6170213 | -92,8 | -85,5 | 0,00203267 | 38,54 | -32,7915 | -0,75447 |
| ccp-miR2592s-1 | 227 | 37,88546 | 62,11454 | 1,203125 | 1,5294118 | -60,7 | -42 | 0,00060427 | 69,58 | -26,7401 | -0,70581 |
| ccp-miR2592s-2 | 279 | 35,84229 | 64,15771 | 1,1058824 | 1,6315789 | -71,4 | -67,5 | 4,30E-05 | 39,89 | -25,5914 | -0,714 |
| ccp-miR2612 | 241 | 33,19502 | 66,80498 | 1,515625 | 1,1052632 | -57 | -53,2 | 0,00157467 | 37,9 | -23,6515 | -0,7125 |
| ccp-miR2642 | 286 | 30,41958 | 69,58042 | 1,3411765 | 1,2894737 | -71,1 | -67,2 | 9,48E-05 | 25,24 | -24,8601 | -0,81724 |
| ccp-miR2657 | 168 | 38,09524 | 61,90476 | 1,212766 | 1,0645161 | -49,7 | -45,9 | 0,00548446 | 18,9 | -29,5833 | -0,77656 |
| ccp-miR2669a | 121 | 38,01653 | 61,98347 | 1,027027 | 2,0666667 | -37,2 | -33,8 | 0,0348931 | 13,3 | -30,7438 | -0,8087 |
| ccp-miR2673a-1 | 145 | 47,58621 | 52,41379 | 0,9 | 0,6428571 | -50,5 | -47,6 | 0,00674203 | 29,98 | -34,8276 | -0,73188 |
| ccp-miR2673a-2 | 213 | 40,37559 | 59,62441 | 1,6458333 | 0,72 | -62,6 | -61,1 | 0,0134032 | 19 | -29,3897 | -0,72791 |
| ccp-miR2873b-1 | 170 | 31,17647 | 68,82353 | 1,1272727 | 1,4090909 | -39,7 | -33,5 | 0,011359 | 24,32 | -23,3529 | -0,74906 |
| ccp-miR2873b-2 | 288 | 36,80556 | 63,19444 | 1 | 1,65 | -89,6 | -83,4 | 4,15E-05 | 31,67 | -31,1111 | -0,84528 |
| ccp-miR2923 | 116 | 31,03448 | 68,96552 | 1,1052632 | 1,1176471 | -27,2 | -25,6 | 0,0110334 | 13,62 | -23,4483 | -0,75556 |
| ccp-miR319-1 | 178 | 48,8764 | 51,1236 | 1,1666667 | 1,3513514 | -88,1 | -84,9 | 0,123917 | 6,58 | -49,4944 | -1,01264 |
| ccp-miR319a-1 | 239 | 48,95397 | 51,04603 | 1,2592593 | 1,2075472 | -82,7 | -78,9 | 0,00084721 | 38,11 | -34,6025 | -0,70684 |
| ccp-miR319a-2 | 190 | 45,78947 | 54,21053 | 1,2391304 | 1,4166667 | -89,5 | -87 | 0,0426307 | 12,61 | -47,1053 | -1,02874 |
| ccp-miR319a-3 | 182 | 41,75824 | 58,24176 | 1,1632653 | 1,1714286 | -79 | -78,6 | 0,077041 | 10,93 | -43,4066 | -1,03947 |
| ccp-miR319c-1 | 193 | 49,74093 | 50,25907 | 1,6216216 | 1,5945946 | -76,1 | -75,7 | 0,0460611 | 14,33 | -39,4301 | -0,79271 |
| ccp-miR319c-2 | 173 | 48,55491 | 51,44509 | 1,1190476 | 1,2702703 | -84,3 | -84,3 | 0,0391576 | 13,67 | -48,7283 | -1,00357 |
| ccp-miR3439 | 313 | 32,26837 | 67,73163 | 1,3555556 | 1,5897436 | -73,8 | -71,4 | 0,00018882 | 45,61 | -23,5783 | -0,73069 |
| ccp-miR3627-1 | 129 | 52,71318 | 47,28682 | 1,2592593 | 1,0606061 | -71,6 | -71,6 | 0,209698 | 5,05 | -55,5039 | -1,05294 |
| ccp-miR3627-2 | 154 | 47,4026 | 52,5974 | 0,8837209 | 0,972973 | -64,7 | -57,3 | 0,00890535 | 26,49 | -42,013 | -0,8863 |
| ccp-miR390 | 164 | 49,39024 | 50,60976 | 1,3714286 | 1,3823529 | -84,6 | -84,6 | 0,0486256 | 9,19 | -51,5854 | -1,04444 |
| ccp-miR390a-1 | 164 | 49,39024 | 50,60976 | 0,7291667 | 0,7234043 | -61,1 | -61,1 | 0,0534317 | 16,63 | -37,2561 | -0,75432 |
| ccp-miR390a-2 | 177 | 45,76271 | 54,23729 | 0,8113208 | 1,1315789 | -79,1 | -78,4 | 0,0564087 | 7,19 | -44,6893 | -0,97654 |
| ccp-miR393a | 140 | 38,57143 | 61,42857 | 1,4571429 | 0,9285714 | -43,2 | -41,6 | 0,00955298 | 14,49 | -30,8571 | -0,8 |
| ccp-miR393b | 172 | 40,11628 | 59,88372 | 0,7758621 | 1,0294118 | -52,6 | -43,9 | 0,00562242 | 23,27 | -30,5814 | -0,76232 |
| ccp-miR394 | 163 | 40,4908 | 59,5092 | 1,255814 | 1 | -63,2 | -63,2 | 0,158586 | 7,96 | -38,773 | -0,95758 |
| ccp-miR394a | 82 | 42,68293 | 57,31707 | 0,9583333 | 1,0588235 | -39,8 | -37,4 | 0,16875 | 3,12 | -48,5366 | -1,13714 |
| ccp-miR395a-1 | 91 | 50,54945 | 49,45055 | 1,8125 | 0,9166667 | -43,8 | -42,9 | 0,302653 | 3,14 | -48,1319 | -0,95217 |
| ccp-miR395a-2 | 133 | 45,86466 | 54,13534 | 1,4 | 1,1034483 | -56,6 | -55,2 | 0,0165251 | 10,82 | -42,5564 | -0,92787 |
| ccp-miR395a-3 | 92 | 50 | 50 | 1,875 | 1,0909091 | -33,1 | -32 | 0,0567042 | 8,11 | -35,9783 | -0,71957 |
| ccp-miR395b-1 | 119 | 39,4958 | 60,5042 | 1,25 | 1,0434783 | -45,9 | -40,1 | 0,0169802 | 20,93 | -38,5714 | -0,9766 |
| ccp-miR395b-2 | 132 | 46,21212 | 53,78788 | 0,7317073 | 0,9677419 | -54 | -48,3 | 0,0982951 | 6,52 | -40,9091 | -0,88525 |
| ccp-miR395h | 316 | 36,07595 | 63,92405 | 1,2444444 | 1,7142857 | -83,7 | -79,9 | 0,00011438 | 44,81 | -26,4873 | -0,73421 |
| ccp-miR395t-1 | 314 | 37,89809 | 62,10191 | 1,1666667 | 1,0517241 | -86,9 | -85,7 | 0,00027639 | 34,43 | -27,6752 | -0,73025 |
| ccp-miR395t-2 | 238 | 37,81513 | 62,18487 | 1,3870968 | 0,9565217 | -63,3 | -52,8 | 0,00059474 | 42,56 | -26,5966 | -0,70333 |
| ccp-miR395x | 247 | 34,41296 | 65,58704 | 1,3823529 | 1,2972973 | -61,2 | -44,1 | 0,00055911 | 59,89 | -24,7773 | -0,72 |
| ccp-miR396-1 | 139 | 44,60432 | 55,39568 | 0,6382979 | 1,5833333 | -48,1 | -48 | 0,0695021 | 13,64 | -34,6043 | -0,77581 |
| ccp-miR396-2 | 103 | 44,66019 | 55,33981 | 1,1923077 | 1,5555556 | -52,7 | -52,3 | 0,263127 | 3,92 | -51,165 | -1,14565 |
| ccp-miR396a | 155 | 44,51613 | 55,48387 | 1,3243243 | 1,2258065 | -59,8 | -57,3 | 0,00805568 | 21,05 | -38,5806 | -0,86667 |
| ccp-miR396b-1 | 103 | 44,66019 | 55,33981 | 0,8387097 | 0,6428571 | -45 | -45 | 0,532565 | 2,91 | -43,6893 | -0,97826 |
| ccp-miR396b-2 | 148 | 42,56757 | 57,43243 | 1,5757576 | 0,6153846 | -45,9 | -38,78 | 0,0136644 | 20,5 | -31,0135 | -0,72857 |
| ccp-miR396c | 206 | 55,82524 | 44,17476 | 1,0222222 | 1,1296296 | -84,6 | -84,3 | 0,00257714 | 37,56 | -41,068 | -0,73565 |
| ccp-miR397a-1 | 107 | 37,38318 | 62,61682 | 1,68 | 1 | -40,7 | -40,7 | 0,413355 | 5,2 | -38,0374 | -1,0175 |
| ccp-miR397a-3 | 101 | 36,63366 | 63,36634 | 0,6410256 | 0,9473684 | -37,5 | -37,2 | 0,251949 | 6,64 | -37,1287 | -1,01351 |
| ccp-miR398 | 128 | 43,75 | 56,25 | 1,1818182 | 1 | -63,6 | -63,6 | 0,100939 | 4,09 | -49,6875 | -1,13571 |
| ccp-miR398a | 149 | 49,66443 | 50,33557 | 0,9230769 | 0,9473684 | -66,1 | -61,7 | 0,0347774 | 10,18 | -44,3624 | -0,89324 |
| ccp-miR398b | 128 | 43,75 | 56,25 | 0,8461538 | 1 | -58 | -58 | 0,147905 | 5,15 | -45,3125 | -1,03571 |
| ccp-miR399-1 | 91 | 40,65934 | 59,34066 | 0,9285714 | 0,6818182 | -44,2 | -44,2 | 0,497793 | 1,42 | -48,5714 | -1,19459 |
| ccp-miR399-2 | 104 | 39,42308 | 60,57692 | 1,0322581 | 1,1578947 | -43,3 | -43,3 | 0,146808 | 7,08 | -41,6346 | -1,0561 |
| ccp-miR399-3 | 116 | 44,82759 | 55,17241 | 1 | 1,2608696 | -54,5 | -51,3 | 0,0472262 | 10,77 | -46,9828 | -1,04808 |
| ccp-miR399-4 | 139 | 48,20144 | 51,79856 | 1,1176471 | 0,8108108 | -59,3 | -55,17 | 0,069912 | 11,32 | -42,6619 | -0,88507 |
| ccp-miR399-5 | 104 | 39,42308 | 60,57692 | 0,96875 | 0,8636364 | -45,5 | -45,4 | 0,439884 | 1,83 | -43,75 | -1,10976 |
| ccp-miR399-6 | 141 | 43,26241 | 56,73759 | 1,2222222 | 1,0333333 | -44,2 | -43,2 | 0,0182455 | 15,34 | -31,3475 | -0,72459 |
| ccp-miR399a | 131 | 44,27481 | 55,72519 | 0,825 | 0,8709677 | -57 | -53,8 | 0,0552043 | 8 | -43,5115 | -0,98276 |
| ccp-miR399f | 159 | 44,02516 | 55,97484 | 1,0227273 | 1 | -66,4 | -65,8 | 0,0498729 | 9,43 | -41,761 | -0,94857 |
| ccp-miR399j | 141 | 45,39007 | 54,60993 | 1,0263158 | 0,8823529 | -61,6 | -61,4 | 0,11783 | 11,56 | -43,6879 | -0,9625 |
| ccp-miR403 | 110 | 40,90909 | 59,09091 | 1,0967742 | 0,9565217 | -42,7 | -40,8 | 0,0412042 | 17,61 | -38,8182 | -0,94889 |
| ccp-miR403a | 114 | 42,98246 | 57,01754 | 0,9117647 | 1,0416667 | -43,6 | -39,2 | 0,0404369 | 12,51 | -38,2456 | -0,8898 |
| ccp-miR408 | 167 | 48,50299 | 51,49701 | 0,755102 | 0,9285714 | -67,7 | -66,1 | 0,0467817 | 8,26 | -40,5389 | -0,8358 |
| ccp-miR408b | 93 | 38,70968 | 61,29032 | 1,5909091 | 0,7142857 | -28,7 | -25,6 | 0,0315582 | 10,13 | -30,8602 | -0,79722 |
| ccp-miR4245 | 174 | 34,48276 | 65,51724 | 1,1509434 | 1,0689655 | -48,1 | -46,8 | 0,0365796 | 17,78 | -27,6437 | -0,80167 |
| ccp-miR4246 | 266 | 33,83459 | 66,16541 | 1,2564103 | 1,0930233 | -80,3 | -73,8 | 0,00050867 | 31,55 | -30,188 | -0,89222 |
| ccp-miR426-1 | 338 | 31,95266 | 68,04734 | 0,8852459 | 1 | -76,9 | -59,6 | 3,16E-05 | 67,7 | -22,7515 | -0,71204 |
| ccp-miR426-2 | 265 | 35,09434 | 64,90566 | 1,5671642 | 2,1 | -68,7 | -62,1 | 0,00027851 | 43,09 | -25,9245 | -0,73871 |
| ccp-miR4342-1 | 266 | 48,1203 | 51,8797 | 1,0909091 | 0,9692308 | -89,8 | -78,82 | 0,00102541 | 7,49 | -34,5833 | -0,79808 |
| ccp-miR4342-2 | 243 | 46,50206 | 53,49794 | 1,0967742 | 1,1320755 | -84,8 | -68,21 | 0,0139048 | 45,15 | -34,8971 | -0,75044 |
| ccp-miR477i-1 | 145 | 53,10345 | 46,89655 | 1,125 | 0,974359 | -63,9 | -59,6 | 0,135968 | 7,26 | -44,069 | -0,82987 |
| ccp-miR477i-2 | 106 | 52,83019 | 47,16981 | 1,2727273 | 1,0740741 | -50,9 | -50,9 | 0,284945 | 10,13 | -48,0189 | -0,90893 |
| ccp-miR482a-1 | 120 | 43,33333 | 56,66667 | 1,2666667 | 1,3636364 | -41,5 | -38,9 | 0,0607858 | 7,49 | -34,5833 | -0,79808 |
| ccp-miR482a-2 | 146 | 48,63014 | 51,36986 | 1,34375 | 1,21875 | -74,5 | -74,5 | 0,0350813 | 7,7 | -51,0274 | -1,0493 |
| ccp-miR482a-3 | 178 | 46,62921 | 53,37079 | 1,5 | 1,4411765 | -89 | -88,9 | 0,0261311 | 8,95 | -50 | -1,07229 |
| ccp-miR482a-4 | 177 | 43,50282 | 56,49718 | 1,4390244 | 1,2647059 | -73,7 | -71,6 | 0,0209346 | 11,77 | -41,6384 | -0,95714 |
| ccp-miR482a-5 | 176 | 43,75 | 56,25 | 1,4146341 | 1,40625 | -82,7 | -81,8 | 0,0321308 | 12,18 | -46,9886 | -1,07403 |
| ccp-miR482b-1 | 169 | 37,27811 | 62,72189 | 0,7377049 | 0,8529412 | -62,7 | -55 | 0,00875338 | 18,96 | -37,1006 | -0,99524 |
| ccp-miR482b-2 | 167 | 40,71856 | 59,28144 | 0,7678571 | 0,8888889 | -58,8 | -53,2 | 0,00630613 | 23,13 | -35,2096 | -0,86471 |
| ccp-miR5013 | 280 | 32,85714 | 67,14286 | 1 | 1,875 | -67,9 | -50,68 | 6,13E-05 | 56,26 | -24,25 | -0,73804 |
| ccp-miR5014a | 252 | 32,14286 | 67,85714 | 0,8191489 | 1,025 | -58 | -45,4 | 0,00010732 | 50,09 | -23,0159 | -0,71605 |
| ccp-miR5020a | 244 | 30,32787 | 69,67213 | 1,1518987 | 1,3125 | -61,2 | -61,2 | 0,0101252 | 20,93 | -25,082 | -0,82703 |
| ccp-miR5042-1 | 72 | 48,61111 | 51,38889 | 1,1764706 | 1,6923077 | -25,8 | -23,4 | 0,170109 | 9,25 | -35,8333 | -0,73714 |
| ccp-miR5042-2 | 79 | 46,83544 | 53,16456 | 1,1 | 1,4666667 | -28,3 | -25,4 | 0,0653589 | 7,69 | -35,8228 | -0,76486 |
| ccp-miR5167b | 176 | 34,09091 | 65,90909 | 1,32 | 1,3076923 | -45,6 | -37,9 | 0,00022601 | 35,36 | -25,9091 | -0,76 |
| ccp-miR5210 | 216 | 31,94444 | 68,05556 | 0,8375 | 1,4642857 | -70 | -67 | 0,00334239 | 18,74 | -32,4074 | -1,01449 |
| ccp-miR5272f | 93 | 43,01075 | 56,98925 | 0,8275862 | 1,6666667 | -31 | -27,4 | 0,0361345 | 13,4 | -33,3333 | -0,775 |
| ccp-miR530 | 228 | 41,66667 | 58,33333 | 0,9850746 | 0,8269231 | -69,2 | -62,2 | 0,00264004 | 40,21 | -30,3509 | -0,72842 |
| ccp-miR5368 | 171 | 57,30994 | 42,69006 | 1,0277778 | 1,2272727 | -72,5 | -70,1 | 0,0419346 | 17,15 | -42,3977 | -0,7398 |
| ccp-miR5640 | 88 | 31,81818 | 68,18182 | 1 | 1,3333333 | -22,3 | -22,3 | 0,329659 | 4,91 | -25,3409 | -0,79643 |
| ccp-miR5648 | 230 | 37,3913 | 62,6087 | 1,0869565 | 1,4571429 | -65,6 | -49,4 | 0,0001656 | 57,12 | -28,5217 | -0,76279 |
| ccp-miR5653-1 | 171 | 30,40936 | 69,59064 | 0,8307692 | 0,7931034 | -43,6 | -42,5 | 0,0860843 | 13,46 | -25,4971 | -0,83846 |
| ccp-miR5653-2 | 145 | 34,48276 | 65,51724 | 1,2093023 | 1,0833333 | -35,8 | -33,8 | 0,00313938 | 22,31 | -24,6897 | -0,716 |
| ccp-miR5658 | 271 | 43,54244 | 56,45756 | 0,9125 | 1,0701754 | -84,1 | -56,74 | 0,00036482 | 66,13 | -31,0332 | -0,71271 |
| ccp-miR5741a-1 | 210 | 33,80952 | 66,19048 | 1,0144928 | 1,3666667 | -52,2 | -51,1 | 0,00330027 | 30,05 | -24,8571 | -0,73521 |
| ccp-miR5741a-1 | 193 | 30,56995 | 69,43005 | 1,0615385 | 1,4583333 | -44 | -35 | 0,00339258 | 30,5 | -22,7979 | -0,74576 |
| ccp-miR5741a-2 | 211 | 33,17536 | 66,82464 | 1,0142857 | 1,9166667 | -51,7 | -47,4 | 0,00455352 | 49,54 | -24,5024 | -0,73857 |
| ccp-miR5780 | 289 | 32,87197 | 67,12803 | 1,1555556 | 1,4358974 | -67,4 | -47,93 | 4,36E-05 | 63,98 | -23,3218 | -0,70947 |
| ccp-miR5780d-3 | 141 | 30,49645 | 69,50355 | 1,2790698 | 1,6875 | -33,1 | -33 | 0,0221398 | 11,59 | -23,4752 | -0,76977 |
| ccp-miR5780d-4 | 160 | 31,875 | 68,125 | 1,18 | 1,6842105 | -43,8 | -43,8 | 0,00534369 | 26,06 | -27,375 | -0,85882 |
| ccp-miR6142 | 222 | 32,88288 | 67,11712 | 0,8395062 | 1,0857143 | -51,2 | -41,1 | 0,00329727 | 49,31 | -23,0631 | -0,70137 |
| ccp-miR6188 | 170 | 57,05882 | 42,94118 | 1,28125 | 0,9019608 | -68,1 | -61,9 | 0,00247564 | 33,27 | -40,0588 | -0,70206 |
| ccp-miR6198 | 200 | 45,5 | 54,5 | 0,5352113 | 1,6764706 | -67,2 | -50,8 | 0,00492785 | 39,51 | -33,6 | -0,73846 |
| ccp-miR6253 | 108 | 50 | 50 | 1,5714286 | 1,25 | -48,6 | -33,1 | 0,0417507 | 21,45 | -45 | -0,9 |
| ccp-miR6281 | 163 | 36,80982 | 63,19018 | 0,9807692 | 1,2222222 | -45 | -32,2 | 0,00172867 | 46,97 | -27,6074 | -0,75 |
| ccp-miR6440b | 320 | 32,1875 | 67,8125 | 1,0092593 | 1,06 | -80,4 | -42,9 | 0,00012059 | 94,16 | -25,125 | -0,78058 |
| ccp-miR6459a-2 | 120 | 53,33333 | 46,66667 | 1,1538462 | 1,0645161 | -55,6 | -55,4 | 0,128401 | 9,51 | -46,3333 | -0,86875 |
| ccp-miR6459a-3 | 169 | 40,23669 | 59,76331 | 1,1489362 | 0,9428571 | -61,9 | -58,5 | 0,00240695 | 41,67 | -36,6272 | -0,91029 |
| ccp-miR6459a-4 | 169 | 40,23669 | 59,76331 | 0,8703704 | 1,0606061 | -67,4 | -62,2 | 0,00218376 | 42 | -39,8817 | -0,99118 |
| ccp-miR6462c | 104 | 42,30769 | 57,69231 | 0,6216216 | 1,3157895 | -31 | -24,1 | 0,0387916 | 25,79 | -29,8077 | -0,70455 |
| ccp-miR6476a | 83 | 38,55422 | 61,44578 | 1,4285714 | 1 | -26,4 | -25,7 | 0,205497 | 4,56 | -31,8072 | -0,825 |
| ccp-miR7122a | 156 | 36,53846 | 63,46154 | 1,75 | 1,375 | -60,7 | -60,7 | 0,0508983 | 11,93 | -38,9103 | -1,06491 |
| ccp-miR7494b | 181 | 49,17127 | 50,82873 | 0,6428571 | 1,1707317 | -73,6 | -69 | 0,00115347 | 24,62 | -40,663 | -0,82697 |
| ccp-miR7502f | 328 | 33,53659 | 66,46341 | 1,2020202 | 0,9642857 | -84,4 | -78,6 | 5,80E-05 | 47,36 | -25,7317 | -0,76727 |
| ccp-miR7504a | 158 | 32,91139 | 67,08861 | 1,0784314 | 1,08 | -45,2 | -45 | 0,00771912 | 16,78 | -28,6076 | -0,86923 |
| ccp-miR7504b | 203 | 30,54187 | 69,45813 | 1,0735294 | 1,6956522 | -47,1 | -44,1 | 0,00382132 | 35,57 | -23,202 | -0,75968 |
| ccp-miR7530 | 136 | 52,20588 | 47,79412 | 1,1666667 | 0,6511628 | -53,3 | -43,07 | 0,0126904 | 24,54 | -39,1912 | -0,7507 |
| ccp-miR7696c | 215 | 30,23256 | 69,76744 | 1,0547945 | 1,4074074 | -68,2 | -62 | 0,000889 | 79,69 | -31,7209 | -1,04923 |
| ccp-miR7743 | 124 | 38,70968 | 61,29032 | 1,5333333 | 1,5263158 | -35 | -34,5 | 0,0441343 | 14,47 | -28,2258 | -0,72917 |
| ccp-miR7822 | 331 | 35,64955 | 64,35045 | 1,2659574 | 2,025641 | -88,8 | -87,4 | 9,92E-05 | 44,67 | -26,8278 | -0,75254 |
| ccp-miR7828 | 302 | 39,7351 | 60,2649 | 1,091954 | 1,2222222 | -85,7 | -82,8 | 0,00090187 | 37,73 | -28,3775 | -0,71417 |
| ccp-miR7982a-1 | 261 | 32,95019 | 67,04981 | 1,2151899 | 1,8666667 | -63,2 | -49,7 | 9,62E-05 | 53,22 | -24,2146 | -0,73488 |
| ccp-miR7982a-2 | 250 | 37,6 | 62,4 | 1,2941176 | 1,8484848 | -70,6 | -68,7 | 0,00049364 | 29,34 | -28,24 | -0,75106 |
| ccp-miR7997c | 109 | 34,86239 | 65,13761 | 1,3666667 | 1,2352941 | -29,1 | -28,5 | 0,0960657 | 9,92 | -26,6972 | -0,76579 |
| ccp-miR8001b | 275 | 35,27273 | 64,72727 | 1,4054054 | 1,6216216 | -77,3 | -76,5 | 0,00019054 | 24,47 | -28,1091 | -0,79691 |
| ccp-miR8044 | 193 | 31,60622 | 68,39378 | 0,7837838 | 1,1034483 | -43,5 | -35,2 | 0,00072751 | 34,15 | -22,5389 | -0,71311 |
| ccp-miR821b | 263 | 40,30418 | 59,69582 | 1,2112676 | 1,4651163 | -78,6 | -75,4 | 0,00285385 | 23,42 | -29,8859 | -0,74151 |
| ccp-miR827 | 324 | 33,95062 | 66,04938 | 1,3777778 | 1,3404255 | -82,1 | -59 | 0,00030386 | 64,8 | -25,3395 | -0,74636 |
| ccp-miR828a | 142 | 40,84507 | 59,15493 | 0,9534884 | 1,1481481 | -45,6 | -43,7 | 0,0154286 | 17,12 | -32,1127 | -0,78621 |
| ccp-miR837 | 269 | 33,829 | 66,171 | 1,2531646 | 1,1162791 | -70,9 | -65,7 | 0,00062667 | 39,2 | -26,3569 | -0,77912 |
| ccp-miR845 | 73 | 41,09589 | 58,90411 | 0,9545455 | 0,875 | -29,6 | -29,6 | 0,571645 | 1,33 | -40,5479 | -0,98667 |
| ccp-miR845b | 140 | 35,71429 | 64,28571 | 1,6470588 | 0,9230769 | -37,3 | -36 | 0,158994 | 15,69 | -26,6429 | -0,746 |
| ccp-miR856 | 180 | 39,44444 | 60,55556 | 0,9818182 | 0,9722222 | -51,5 | -44,5 | 0,00487609 | 24,65 | -28,6111 | -0,72535 |
| ccp-miR8578 | 278 | 35,61151 | 64,38849 | 1,1058824 | 1,2 | -70,1 | -64,5 | 0,00041148 | 37,39 | -25,2158 | -0,70808 |
| ccp-miR8691 | 287 | 32,05575 | 67,94425 | 1,1666667 | 2,0666667 | -65,3 | -59,3 | 3,14E-05 | 53,74 | -22,7526 | -0,70978 |
| ccp-miR8709a | 324 | 33,95062 | 66,04938 | 1,2061856 | 0,9642857 | -84,5 | -62,7 | 9,59E-05 | 68,8 | -26,0802 | -0,76818 |
| ccp-miR8746-1 | 91 | 48,35165 | 51,64835 | 1,1363636 | 1,3157895 | -37 | -37 | 0,221261 | 13,07 | -40,6593 | -0,84091 |
| ccp-miR8746-2 | 91 | 48,35165 | 51,64835 | 1,1363636 | 1,3157895 | -37 | -37 | 0,221261 | 13,07 | -40,6593 | -0,84091 |
| ccp-miR902a | 168 | 49,40476 | 50,59524 | 0,8888889 | 1,1282051 | -63,5 | -63,5 | 0,0782618 | 6,78 | -37,7976 | -0,76506 |
| ccp-miR9557 | 141 | 31,20567 | 68,79433 | 1,1555556 | 1,2 | -39 | -35,7 | 0,00490979 | 18,25 | -27,6596 | -0,88636 |
| ccp-miR9559 | 152 | 31,57895 | 68,42105 | 1,4186047 | 2 | -33,9 | -21,95 | 0,00754809 | 35,11 | -22,3026 | -0,70625 |
| ccp-miR9568 | 134 | 36,56716 | 63,43284 | 1,1794872 | 1,0416667 | -37,9 | -34,9 | 0,0149627 | 21,43 | -28,2836 | -0,77347 |
| ccp-miR9752 | 103 | 43,68932 | 56,31068 | 1,6363636 | 0,875 | -35,9 | -35,9 | 0,322932 | 4,21 | -34,8544 | -0,79778 |
| ccp-miR9773 | 214 | 30,37383 | 69,62617 | 1,1911765 | 1,6 | -45,7 | -34,6 | 0,00040433 | 49,91 | -21,3551 | -0,70308 |
