# Supplementary material for: A genome-wide analysis of the RNA-guided silencing pathway in coffee reveals insights into its regulatory mechanisms
Source: PLoS One. 2017 Apr 27;12(4):e0176333. doi: 10.1371/journal.pone.0176333 (PMC5407642; doi:10.1371/journal.pone.0176333)
Supplement: S4 Table — miRNA names, Target ID (Locus Name) in C. canephora, Expectation scoring, unpaired energy (UPE) required to open the secondary structure around the miRNA target site, the start and end position on the miRNA and the Target, the sequence alignment of the miRNA and Target sequences, and the type of inhibition method. (DOCX) [file pone.0176333.s007.docx]

| **miRNA** | **Target ID** | **Expectation** | **UPE** | **miRNA**  **start** | **miRNA**  **end** | **Target**  **start** | **Target**  **end** | **miRNA aligned fragment** | **Target_aligned_fragment** | **Inhibition** |
| --- | --- | --- | --- | --- | --- | --- | --- | --- | --- | --- |
| ccp-miR1023e-5p | Cc00_g28650 | 3.0 | 12.384 | 1 | 21 | 1452 | 1472 | ACACUCUCUCCGUCUCACUUU | AAAGUGUGACGGAGGGAGUAU | Cleavage |
| ccp-miR1023e-5p | Cc01_g19050 | 3.0 | 17.441 | 1 | 20 | 3707 | 3726 | ACACUCUCUCCGUCUCACUU | CAGUGAGACGGCGAGAGUGA | Translation |
| ccp-miR1023e-5p | Cc03_g00490 | 3.0 | 14.059 | 1 | 21 | 1343 | 1363 | ACACUCUCUCCGUCUCACUUU | GAAGAGAGAGAGAGAGAGUGU | Translation |
| ccp-miR1023e-5p | Cc05_g06680 | 3.0 | 16.305 | 1 | 21 | 151 | 171 | ACACUCUCUCCGUCUCACUUU | GAAGUGGGAGGGAGGGAGUGA | Cleavage |
| ccp-miR1030i-5p | Cc00_g26210 | 3.0 | 6.709 | 1 | 20 | 598 | 617 | CCUGCAUCUGCACCCGCACC | GGUGUUGGUGGAGGUGCAGG | Translation |
| ccp-miR1030i-5p | Cc02_g30270 | 2.5 | 14.659 | 1 | 21 | 588 | 608 | CCUGCAUCUGCACCCGCACCU | GGGUGCUGGUGCAGAUACAGG | Cleavage |
| ccp-miR1030i-5p | Cc06_g05860 | 3.0 | 22.639 | 1 | 21 | 1365 | 1385 | CCUGCAUCUGCACCCGCACCU | AGAUGACGGUGCAGAUGCAGG | Cleavage |
| ccp-miR1030i-5p | Cc09_g01080 | 3.0 | 12.456 | 1 | 21 | 2143 | 2163 | CCUGCAUCUGCACCCGCACCU | GGAUGCGGAUGAAGAUGCAGG | Translation |
| ccp-miR1030i-5p | Cc10_g02120 | 3.0 | 11.724 | 1 | 20 | 182 | 201 | CCUGCAUCUGCACCCGCACC | GGUGGGGGUGGGGAUGUAGG | Translation |
| ccp-miR1060-5p | Cc00_g24100 | 3.0 | 18.187 | 1 | 20 | 412 | 431 | UUUGUCAUAGGAUUACAUAU | GUACGUAAUCCUCUGACGAA | Cleavage |
| ccp-miR1060-5p | Cc02_g01300 | 3.0 | 16.137 | 1 | 20 | 248 | 267 | UUUGUCAUAGGAUUACAUAU | AUAUGAAAUAUUAUGACGAA | Translation |
| ccp-miR1060-5p | Cc02_g18330 | 3.0 | 11.173 | 1 | 20 | 26 | 44 | UUUGUCAUAGGAUUACAUAU | AUAUGUAAUCC-GUGACGAA | Translation |
| ccp-miR1060-5p | Cc05_g04310 | 3.0 | 13.684 | 1 | 21 | 1101 | 1121 | UUUGUCAUAGGAUUACAUAUA | UAUAUGUAAUUUGGUGAUAAA | Translation |
| ccp-miR1060-5p | Cc06_g11030 | 3.0 | 13.998 | 1 | 20 | 830 | 849 | UUUGUCAUAGGAUUACAUAU | AUUUGUGAUCCGAUGACAGA | Translation |
| ccp-miR1078-5p | Cc00_g03540 | 3.0 | 16.29 | 1 | 21 | 394 | 414 | CUUGAUUGAUUCAAUUGAUUU | GAGUCAAAUGAAUCAAUGAAG | Cleavage |
| ccp-miR1078-5p | Cc01_g07910 | 3.0 | 17.26 | 1 | 20 | 78 | 96 | CUUGAUUGAUUCAAUUGAUU | AAUUGAUUGA-UCAAUCAAG | Translation |
| ccp-miR1078-5p | Cc01_g11760 | 2.5 | 10.487 | 1 | 20 | 789 | 808 | CUUGAUUGAUUCAAUUGAUU | AGUUAAAUGGAUCAAUCAAG | Cleavage |
| ccp-miR1078-5p | Cc01_g12950 | 3.0 | 15.871 | 1 | 20 | 492 | 511 | CUUGAUUGAUUCAAUUGAUU | CAUUAGCUGAAUCAAUCAAG | Cleavage |
| ccp-miR1078-5p | Cc02_g00400 | 2.5 | 14.04 | 1 | 21 | 140 | 160 | CUUGAUUGAUUCAAUUGAUUU | AAAUCAUUUGGAUCAAUCAAA | Cleavage |
| ccp-miR1078-5p | Cc02_g14040 | 3.0 | 15.329 | 1 | 20 | 983 | 1002 | CUUGAUUGAUUCAAUUGAUU | GAUCGUUUGAAUCAGUCAGG | Cleavage |
| ccp-miR1078-5p | Cc02_g19490 | 3.0 | 12.713 | 1 | 20 | 295 | 314 | CUUGAUUGAUUCAAUUGAUU | AAUCAAUUGAAUUUAUCAAC | Cleavage |
| ccp-miR1078-5p | Cc02_g23190 | 3.0 | 18.385 | 1 | 20 | 593 | 611 | CUUGAUUGAUUCAAUUGAUU | AAUCAAUUGGAU-GAUCAAG | Cleavage |
| ccp-miR1078-5p | Cc02_g25790 | 3.0 | 15.253 | 1 | 21 | 1996 | 2016 | CUUGAUUGAUUCAAUUGAUUU | GAAUUAAUUGAACCGAUCAAA | Translation |
| ccp-miR1078-5p | Cc03_g00260 | 3.0 | 20.285 | 1 | 21 | 350 | 370 | CUUGAUUGAUUCAAUUGAUUU | GAAUCAGUUGAAUCAGCUAAG | Cleavage |
| ccp-miR1078-5p | Cc03_g15110 | 2.5 | 10.02 | 1 | 21 | 267 | 288 | CUUGAUUGAUUC-AAUUGAUUU | AAAUCAAUUUGAAUCGAUCAAG | Cleavage |
| ccp-miR1078-5p | Cc06_g10150 | 3.0 | 10.399 | 1 | 21 | 569 | 589 | CUUGAUUGAUUCAAUUGAUUU | AGAUCAAUUUAACCAGUCAAG | Translation |
| ccp-miR1078-5p | Cc06_g16740 | 2.5 | 14.09 | 1 | 21 | 3103 | 3123 | CUUGAUUGAUUCAAUUGAUUU | AUAUUAAUCGAAUCAAUCAAG | Cleavage |
| ccp-miR1078-5p | Cc07_g07550 | 2.0 | 13.853 | 1 | 20 | 1348 | 1367 | CUUGAUUGAUUCAAUUGAUU | AAUCAAUUGAAUCAAUUGAC | Cleavage |
| ccp-miR1078-5p | Cc09_g00840 | 2.5 | 11.564 | 1 | 20 | 158 | 176 | CUUGAUUGAUUCAAUUGAUU | AAUCAAUU-AAUCAGUCAAG | Cleavage |
| ccp-miR1078-5p | Cc10_g02400 | 2.5 | 12.441 | 1 | 21 | 2072 | 2092 | CUUGAUUGAUUCAAUUGAUUU | GGAUCAUUUGAAUAAAUCAAG | Cleavage |
| ccp-miR1078-5p | Cc10_g07640 | 2.5 | 12.461 | 1 | 21 | 1455 | 1474 | CUUGAUUGAUUCAAUUGAUUU | AAAUCAAUUGAAUCAA-CAAG | Cleavage |
| ccp-miR1089-5p | Cc00_g22230 | 3.0 | 15.542 | 1 | 21 | 1338 | 1358 | AGAUCAUCUUGCAUUGUUUGA | UCAAAGGAUACAGGAUGAUCU | Cleavage |
| ccp-miR1089-5p | Cc00_g25340 | 1.0 | 18.639 | 1 | 21 | 174 | 194 | AGAUCAUCUUGCAUUGUUUGA | UCAAACAGUGCAAGAUGGUCU | Cleavage |
| ccp-miR1089-5p | Cc01_g07930 | 2.5 | 22.97 | 1 | 21 | 1524 | 1544 | AGAUCAUCUUGCAUUGUUUGA | UGAAACAAUGCAGAAUGAUCU | Cleavage |
| ccp-miR1089-5p | Cc01_g07940 | 3.0 | 14.956 | 1 | 21 | 1467 | 1487 | AGAUCAUCUUGCAUUGUUUGA | UCAGACAGUACUAGAUGAUCU | Translation |
| ccp-miR1089-5p | Cc01_g08040 | 2.0 | 17.044 | 1 | 21 | 2096 | 2116 | AGAUCAUCUUGCAUUGUUUGA | UCAAGCAAUGCAAGAUGAGCU | Cleavage |
| ccp-miR1089-5p | Cc01_g08070 | 1.0 | 13.651 | 1 | 21 | 2409 | 2429 | AGAUCAUCUUGCAUUGUUUGA | UCAAACGAUGCAAGAUGGUCU | Cleavage |
| ccp-miR1089-5p | Cc01_g08110 | 1.0 | 16.975 | 1 | 21 | 5073 | 5093 | AGAUCAUCUUGCAUUGUUUGA | UCAAACGAUGCAAGAUGGUCU | Cleavage |
| ccp-miR1089-5p | Cc02_g12180 | 2.5 | 19.113 | 1 | 20 | 723 | 742 | AGAUCAUCUUGCAUUGUUUG | CAAAAAAUGGAAGAUGAUUU | Translation |
| ccp-miR1089-5p | Cc03_g05200 | 2.5 | 21.303 | 1 | 20 | 239 | 258 | AGAUCAUCUUGCAUUGUUUG | CGAACAAUGGAGGAUGGUCU | Translation |
| ccp-miR1089-5p | Cc04_g06740 | 3.0 | 24.112 | 1 | 20 | 3114 | 3133 | AGAUCAUCUUGCAUUGUUUG | AAAACAAUGGAGGAUGGUCU | Translation |
| ccp-miR1089-5p | Cc06_g02350 | 3.0 | 21.402 | 1 | 21 | 789 | 809 | AGAUCAUCUUGCAUUGUUUGA | UCAAACAGCUCAGGAUGAUCU | Cleavage |
| ccp-miR1089-5p | Cc06_g16580 | 3.0 | 17.868 | 1 | 20 | 4932 | 4951 | AGAUCAUCUUGCAUUGUUUG | UGGGAAAUGCAAGAUGAUCU | Cleavage |
| ccp-miR1089-5p | Cc07_g05370 | 2.5 | 18.728 | 1 | 20 | 1200 | 1219 | AGAUCAUCUUGCAUUGUUUG | CAAGCAACGCUAGAUGAUCU | Translation |
| ccp-miR1089-5p | Cc08_g13760 | 3.0 | 20.611 | 1 | 20 | 782 | 801 | AGAUCAUCUUGCAUUGUUUG | CCAGCAUUGCAAGAUGAUUU | Cleavage |
| ccp-miR1089-5p | Cc09_g03190 | 3.0 | 14.734 | 1 | 20 | 1308 | 1327 | AGAUCAUCUUGCAUUGUUUG | CAGACAAAGCAAGAUGACCU | Cleavage |
| ccp-miR1089-5p | Cc10_g06690 | 3.0 | 14.946 | 1 | 20 | 427 | 446 | AGAUCAUCUUGCAUUGUUUG | CAGGCAAUGCUAGAUGGUUU | Translation |
| ccp-miR1089-5p | Cc10_g11110 | 3.0 | 18.409 | 1 | 21 | 371 | 391 | AGAUCAUCUUGCAUUGUUUGA | UGGAACAAUCCAAGAUGAUUU | Cleavage |
| ccp-miR1089-5p | Cc10_g11540 | 3.0 | 16.154 | 1 | 20 | 669 | 688 | AGAUCAUCUUGCAUUGUUUG | CAAACAGUACAAGAUGGUCG | Cleavage |
| ccp-miR1089-5p | Cc11_g13250 | 2.5 | 16.679 | 1 | 20 | 577 | 596 | AGAUCAUCUUGCAUUGUUUG | AAAACAAUGCAAGAUUAUCU | Cleavage |
| ccp-miR1127-1-3p | Cc01_g09350 | 3.0 | 17.022 | 1 | 21 | 631 | 651 | AACUACUCCCUCCGUCCCGUU | AAUGGGACGGAGGAAGUAUUU | Cleavage |
| ccp-miR1127-1-3p | Cc02_g30450 | 3.0 | 19.337 | 1 | 20 | 533 | 552 | AACUACUCCCUCCGUCCCGU | AAGGAACGGAGGGAGUAGUA | Cleavage |
| ccp-miR1127-1-3p | Cc09_g00770 | 2.0 | 19.025 | 1 | 21 | 2261 | 2281 | AACUACUCCCUCCGUCCCGUU | AACAGGACGGACGGAGUAGUU | Translation |
| ccp-miR1127-1-3p | Cc10_g13080 | 2.0 | 15.712 | 1 | 20 | 83 | 102 | AACUACUCCCUCCGUCCCGU | AUAGGACGGAGGGAGUGGUU | Cleavage |
| ccp-miR1127-1-3p | Cc11_g15390 | 3.0 | 17.143 | 1 | 20 | 1043 | 1062 | AACUACUCCCUCCGUCCCGU | GCGGGAUGGAGAGGGUGGUU | Translation |
| ccp-miR1127-2-5p | Cc01_g09350 | 3.0 | 17.022 | 1 | 20 | 632 | 651 | AACUACUCCCUCCGUCUCAU | AUGGGACGGAGGAAGUAUUU | Cleavage |
| ccp-miR1127-2-5p | Cc02_g18180 | 2.5 | 18.564 | 1 | 20 | 9714 | 9733 | AACUACUCCCUCCGUCUCAU | AUGAGACGGAGGCAGUUGUU | Cleavage |
| ccp-miR1127-2-5p | Cc06_g19490 | 3.0 | 14.861 | 1 | 20 | 1341 | 1360 | AACUACUCCCUCCGUCUCAU | AGGAGAUGGAGGGAGUAGAU | Cleavage |
| ccp-miR1127-2-5p | Cc10_g13080 | 2.0 | 15.712 | 1 | 21 | 82 | 102 | AACUACUCCCUCCGUCUCAUA | UAUAGGACGGAGGGAGUGGUU | Cleavage |
| ccp-miR1128-1-5p | Cc02_g30450 | 3.0 | 19.337 | 1 | 20 | 533 | 552 | UACUACUCCCUCCGUCCCAC | AAGGAACGGAGGGAGUAGUA | Cleavage |
| ccp-miR1128-2-3p | Cc02_g30450 | 3.0 | 19.337 | 1 | 20 | 533 | 552 | UACUACUCCCUCCGUCCCAC | AAGGAACGGAGGGAGUAGUA | Cleavage |
| ccp-miR1128-3-3p | Cc02_g30450 | 2.0 | 19.337 | 1 | 20 | 533 | 552 | UACUACUCCCUCCGUCCCAU | AAGGAACGGAGGGAGUAGUA | Cleavage |
| ccp-miR1128-3-3p | Cc03_g02340 | 2.5 | 19.574 | 1 | 21 | 556 | 576 | UACUACUCCCUCCGUCCCAUU | AAUGGGAUGGAGGGGGUUGUA | Cleavage |
| ccp-miR1128-3-3p | Cc10_g13080 | 2.5 | 15.712 | 1 | 20 | 83 | 102 | UACUACUCCCUCCGUCCCAU | AUAGGACGGAGGGAGUGGUU | Cleavage |
| ccp-miR1128-4-5p | Cc02_g30450 | 2.0 | 19.337 | 1 | 20 | 533 | 552 | UACUACUCCCUCCGUCCCAU | AAGGAACGGAGGGAGUAGUA | Cleavage |
| ccp-miR1128-4-5p | Cc03_g02340 | 2.5 | 19.574 | 1 | 21 | 556 | 576 | UACUACUCCCUCCGUCCCAUU | AAUGGGAUGGAGGGGGUUGUA | Cleavage |
| ccp-miR1128-4-5p | Cc10_g13080 | 2.5 | 15.712 | 1 | 20 | 83 | 102 | UACUACUCCCUCCGUCCCAU | AUAGGACGGAGGGAGUGGUU | Cleavage |
| ccp-miR1435-3p | Cc00_g05370 | 3.0 | 16.357 | 1 | 20 | 861 | 880 | UUUCUUAAAACAAACUUUUU | AAGAGGGAUGUUUUAAGAAA | Cleavage |
| ccp-miR1435-3p | Cc01_g06760 | 3.0 | 11.052 | 1 | 20 | 977 | 996 | UUUCUUAAAACAAACUUUUU | AAGGAAUUUGUAUUAAGAAA | Translation |
| ccp-miR1435-3p | Cc01_g13570 | 3.0 | 14.812 | 1 | 20 | 2256 | 2275 | UUUCUUAAAACAAACUUUUU | GAAAAGUUUGUUCUGAGAAU | Cleavage |
| ccp-miR1435-3p | Cc02_g06600 | 3.0 | 19.526 | 1 | 20 | 396 | 415 | UUUCUUAAAACAAACUUUUU | AAGAACUUUGUUUUAGGAAU | Cleavage |
| ccp-miR1435-3p | Cc02_g10120 | 3.0 | 22.943 | 1 | 20 | 79 | 98 | UUUCUUAAAACAAACUUUUU | GACAAGUUUGUCUUAAGAGA | Translation |
| ccp-miR1435-3p | Cc02_g23780 | 3.0 | 15.307 | 1 | 20 | 2014 | 2033 | UUUCUUAAAACAAACUUUUU | AAAAGCUGUGUUUUAAGAGA | Cleavage |
| ccp-miR1435-3p | Cc02_g28320 | 1.5 | 17.369 | 1 | 20 | 591 | 610 | UUUCUUAAAACAAACUUUUU | AGAAGGUUUGUUUUAGGAAA | Cleavage |
| ccp-miR1435-3p | Cc04_g07510 | 2.5 | 13.518 | 1 | 20 | 2209 | 2228 | UUUCUUAAAACAAACUUUUU | AAAGAUUUUGUUUUGAGAGA | Cleavage |
| ccp-miR1435-3p | Cc04_g08460 | 3.0 | 18.877 | 1 | 20 | 913 | 932 | UUUCUUAAAACAAACUUUUU | CAAAAGCUUGUUUUGAGAAG | Cleavage |
| ccp-miR1435-3p | Cc06_g07330 | 3.0 | 20.215 | 1 | 20 | 2886 | 2905 | UUUCUUAAAACAAACUUUUU | AAAGAGGUUCUUUUAAGAGA | Translation |
| ccp-miR1435-3p | Cc07_g09800 | 3.0 | 16.823 | 1 | 20 | 2406 | 2425 | UUUCUUAAAACAAACUUUUU | AAGAAGUUUGUGGUAGGAAA | Translation |
| ccp-miR1435-3p | Cc07_g16800 | 3.0 | 21.712 | 1 | 20 | 180 | 199 | UUUCUUAAAACAAACUUUUU | AAAAAGUUUGAUUUAUGGAA | Translation |
| ccp-miR1435-3p | Cc08_g00150 | 3.0 | 18.906 | 1 | 20 | 410 | 429 | UUUCUUAAAACAAACUUUUU | ACAAAGCAUGUUUUAAGAAA | Cleavage |
| ccp-miR1435-3p | Cc09_g09120 | 3.0 | 16.309 | 1 | 20 | 477 | 496 | UUUCUUAAAACAAACUUUUU | AAGAGGGAUGUUUUAAGAAA | Cleavage |
| ccp-miR1435-3p | Cc09_g10270 | 3.0 | 8.741 | 1 | 20 | 33 | 52 | UUUCUUAAAACAAACUUUUU | GAAGAGUUUCUUGUAAGAAA | Translation |
| ccp-miR1435-3p | Cc11_g11050 | 2.5 | 15.573 | 1 | 20 | 1486 | 1505 | UUUCUUAAAACAAACUUUUU | GAAAGGUUUGUUUUGAGGAG | Cleavage |
| ccp-miR1438-1-3p | Cc00_g11780 | 3.0 | 8.27 | 1 | 20 | 525 | 544 | AGGGUAAUUUUGACAUUUUU | AAAAAUGUCAAAAUUAGCUG | Cleavage |
| ccp-miR1438-1-3p | Cc01_g08770 | 3.0 | 14.96 | 1 | 20 | 205 | 224 | AGGGUAAUUUUGACAUUUUU | AAAAAUGUCAAGAAUAUUCU | Cleavage |
| ccp-miR1438-1-3p | Cc04_g08880 | 3.0 | 18.354 | 1 | 22 | 1022 | 1043 | AGGGUAAUUUUGACAUUUUUAG | CCAAAGAGUUCAAGAUUACCCU | Cleavage |
| ccp-miR1438-1-3p | Cc05_g05690 | 3.0 | 18.185 | 1 | 20 | 4362 | 4381 | AGGGUAAUUUUGACAUUUUU | AGAAACGUUAAAAUUGCUCU | Cleavage |
| ccp-miR1438-1-3p | Cc06_g11250 | 3.0 | 19.064 | 1 | 20 | 930 | 949 | AGGGUAAUUUUGACAUUUUU | AGGAGUGUCAAUAUUAUCCU | Translation |
| ccp-miR1438-1-3p | Cc06_g21680 | 3.0 | 21.788 | 1 | 20 | 1538 | 1557 | AGGGUAAUUUUGACAUUUUU | AGAAAUUUCAGGAUUAUCCU | Cleavage |
| ccp-miR1438-1-3p | Cc09_g01740 | 3.0 | 13.687 | 1 | 20 | 857 | 876 | AGGGUAAUUUUGACAUUUUU | AUGAAUGUCAAAGUUAUCUU | Cleavage |
| ccp-miR1438-1-3p | Cc10_g03210 | 3.0 | 14.021 | 1 | 20 | 252 | 271 | AGGGUAAUUUUGACAUUUUU | AAAAUUGUCAAAUUUAUUCU | Cleavage |
| ccp-miR1438-1-3p | Cc10_g07030 | 3.0 | 21.662 | 1 | 20 | 1616 | 1635 | AGGGUAAUUUUGACAUUUUU | CAAAAUAUUAAAAUUGCCCU | Cleavage |
| ccp-miR1438-1-3p | Cc10_g07040 | 3.0 | 20.104 | 1 | 20 | 1290 | 1309 | AGGGUAAUUUUGACAUUUUU | CAAAAUAUUAAAAUUGCCCU | Cleavage |
| ccp-miR1438-1-3p | Cc11_g07530 | 3.0 | 11.884 | 1 | 22 | 1132 | 1153 | AGGGUAAUUUUGACAUUUUUAG | CUGAAGGUGUUAAGAUUAUCCU | Cleavage |
| ccp-miR1438-2-3p | Cc00_g11780 | 3.0 | 8.27 | 1 | 20 | 525 | 544 | AGGGUAAUUUUGACAUUUUU | AAAAAUGUCAAAAUUAGCUG | Cleavage |
| ccp-miR1438-2-3p | Cc01_g08770 | 3.0 | 14.96 | 1 | 20 | 205 | 224 | AGGGUAAUUUUGACAUUUUU | AAAAAUGUCAAGAAUAUUCU | Cleavage |
| ccp-miR1438-2-3p | Cc04_g08880 | 3.0 | 18.354 | 1 | 22 | 1022 | 1043 | AGGGUAAUUUUGACAUUUUUAG | CCAAAGAGUUCAAGAUUACCCU | Cleavage |
| ccp-miR1438-2-3p | Cc05_g05690 | 3.0 | 18.185 | 1 | 20 | 4362 | 4381 | AGGGUAAUUUUGACAUUUUU | AGAAACGUUAAAAUUGCUCU | Cleavage |
| ccp-miR1438-2-3p | Cc06_g11250 | 3.0 | 19.064 | 1 | 20 | 930 | 949 | AGGGUAAUUUUGACAUUUUU | AGGAGUGUCAAUAUUAUCCU | Translation |
| ccp-miR1438-2-3p | Cc06_g21680 | 3.0 | 21.788 | 1 | 20 | 1538 | 1557 | AGGGUAAUUUUGACAUUUUU | AGAAAUUUCAGGAUUAUCCU | Cleavage |
| ccp-miR1438-2-3p | Cc09_g01740 | 3.0 | 13.687 | 1 | 20 | 857 | 876 | AGGGUAAUUUUGACAUUUUU | AUGAAUGUCAAAGUUAUCUU | Cleavage |
| ccp-miR1438-2-3p | Cc10_g03210 | 3.0 | 14.021 | 1 | 20 | 252 | 271 | AGGGUAAUUUUGACAUUUUU | AAAAUUGUCAAAUUUAUUCU | Cleavage |
| ccp-miR1438-2-3p | Cc10_g07030 | 3.0 | 21.662 | 1 | 20 | 1616 | 1635 | AGGGUAAUUUUGACAUUUUU | CAAAAUAUUAAAAUUGCCCU | Cleavage |
| ccp-miR1438-2-3p | Cc10_g07040 | 3.0 | 20.104 | 1 | 20 | 1290 | 1309 | AGGGUAAUUUUGACAUUUUU | CAAAAUAUUAAAAUUGCCCU | Cleavage |
| ccp-miR1438-2-3p | Cc11_g07530 | 3.0 | 11.884 | 1 | 22 | 1132 | 1153 | AGGGUAAUUUUGACAUUUUUAG | CUGAAGGUGUUAAGAUUAUCCU | Cleavage |
| ccp-miR1438-3-5p | Cc00_g11780 | 3.0 | 8.27 | 1 | 20 | 525 | 544 | AGGGUAAUUUUGACAUUUUU | AAAAAUGUCAAAAUUAGCUG | Cleavage |
| ccp-miR1438-3-5p | Cc01_g08770 | 3.0 | 14.96 | 1 | 20 | 205 | 224 | AGGGUAAUUUUGACAUUUUU | AAAAAUGUCAAGAAUAUUCU | Cleavage |
| ccp-miR1438-3-5p | Cc04_g08880 | 3.0 | 18.354 | 1 | 22 | 1022 | 1043 | AGGGUAAUUUUGACAUUUUUAG | CCAAAGAGUUCAAGAUUACCCU | Cleavage |
| ccp-miR1438-3-5p | Cc05_g05690 | 3.0 | 18.185 | 1 | 20 | 4362 | 4381 | AGGGUAAUUUUGACAUUUUU | AGAAACGUUAAAAUUGCUCU | Cleavage |
| ccp-miR1438-3-5p | Cc06_g11250 | 3.0 | 19.064 | 1 | 20 | 930 | 949 | AGGGUAAUUUUGACAUUUUU | AGGAGUGUCAAUAUUAUCCU | Translation |
| ccp-miR1438-3-5p | Cc06_g21680 | 3.0 | 21.788 | 1 | 20 | 1538 | 1557 | AGGGUAAUUUUGACAUUUUU | AGAAAUUUCAGGAUUAUCCU | Cleavage |
| ccp-miR1438-3-5p | Cc09_g01740 | 3.0 | 13.687 | 1 | 20 | 857 | 876 | AGGGUAAUUUUGACAUUUUU | AUGAAUGUCAAAGUUAUCUU | Cleavage |
| ccp-miR1438-3-5p | Cc10_g03210 | 3.0 | 14.021 | 1 | 20 | 252 | 271 | AGGGUAAUUUUGACAUUUUU | AAAAUUGUCAAAUUUAUUCU | Cleavage |
| ccp-miR1438-3-5p | Cc10_g07030 | 3.0 | 21.662 | 1 | 20 | 1616 | 1635 | AGGGUAAUUUUGACAUUUUU | CAAAAUAUUAAAAUUGCCCU | Cleavage |
| ccp-miR1438-3-5p | Cc10_g07040 | 3.0 | 20.104 | 1 | 20 | 1290 | 1309 | AGGGUAAUUUUGACAUUUUU | CAAAAUAUUAAAAUUGCCCU | Cleavage |
| ccp-miR1438-3-5p | Cc11_g07530 | 3.0 | 11.884 | 1 | 22 | 1132 | 1153 | AGGGUAAUUUUGACAUUUUUAG | CUGAAGGUGUUAAGAUUAUCCU | Cleavage |
| ccp-miR1446-5p | Cc11_g16400 | 1.0 | 19.351 | 1 | 22 | 705 | 726 | UGAACUCUCUCCCUCAACGGCU | AGCCUUUGAGGGAGAGAGUUCA | Cleavage |
| ccp-miR1510b-3p | Cc00_g03300 | 3.0 | 17.144 | 1 | 23 | 538 | 560 | GUUGUUUUACCUAUUCCACUCAU | AUGGGUGGGAUUGGCAAAACAAC | Translation |
| ccp-miR1510b-3p | Cc00_g16840 | 2.5 | 17.426 | 1 | 23 | 622 | 644 | GUUGUUUUACCUAUUCCACUCAU | AUGGGUGGAUUAGGCAAAACAAC | Translation |
| ccp-miR1510b-3p | Cc00_g19440 | 3.0 | 22.165 | 1 | 23 | 1 | 23 | GUUGUUUUACCUAUUCCACUCAU | AUGCCUGGGAUAGGUAAGACAAC | Cleavage |
| ccp-miR1510b-3p | Cc00_g24830 | 2.5 | 13.078 | 1 | 23 | 1 | 23 | GUUGUUUUACCUAUUCCACUCAU | AUGGGUGGAUUAGGCAAAACAAC | Translation |
| ccp-miR1510b-3p | Cc01_g01600 | 2.5 | 21.287 | 1 | 23 | 538 | 560 | GUUGUUUUACCUAUUCCACUCAU | AUGGGUGGAUUAGGCAAAACAAC | Translation |
| ccp-miR1510b-3p | Cc01_g03180 | 2.5 | 14.136 | 1 | 23 | 1 | 23 | GUUGUUUUACCUAUUCCACUCAU | AUGGGUGGAAUCGGGAAAACAAC | Translation |
| ccp-miR1510b-3p | Cc01_g05220 | 2.5 | 20.729 | 1 | 23 | 547 | 569 | GUUGUUUUACCUAUUCCACUCAU | AUGGGUGGAUUAGGCAAAACAAC | Translation |
| ccp-miR1510b-3p | Cc01_g07930 | 2.5 | 19.682 | 1 | 23 | 1936 | 1958 | GUUGUUUUACCUAUUCCACUCAU | AUGCCUGGAAUAGGUAAGACAAC | Cleavage |
| ccp-miR1510b-3p | Cc01_g07950 | 3.0 | 19.297 | 1 | 23 | 679 | 701 | GUUGUUUUACCUAUUCCACUCAU | AUGCCUGGAAUAGGUAAGACGAC | Cleavage |
| ccp-miR1510b-3p | Cc02_g26510 | 3.0 | 19.638 | 1 | 23 | 616 | 638 | GUUGUUUUACCUAUUCCACUCAU | AUGGGAGGUAUAGGUAAGACAAC | Cleavage |
| ccp-miR1510b-3p | Cc02_g28600 | 3.0 | 24.33 | 1 | 23 | 625 | 647 | GUUGUUUUACCUAUUCCACUCAU | AUGGGUGGAAUUGGCAAGACAAC | Translation |
| ccp-miR1510b-3p | Cc03_g06140 | 3.0 | 17.897 | 1 | 24 | 842 | 865 | GUUGUUUUACCUAUUCCACUCAUU | AAUGGGUGGAAUCGGGAAGACAAC | Translation |
| ccp-miR1510b-3p | Cc03_g06170 | 3.0 | 23.666 | 1 | 24 | 1003 | 1026 | GUUGUUUUACCUAUUCCACUCAUU | AAUGGGUGGAAUCGGCAAGACAAC | Translation |
| ccp-miR1510b-3p | Cc03_g06190 | 2.0 | 11.574 | 1 | 24 | 645 | 668 | GUUGUUUUACCUAUUCCACUCAUU | AAUGGGUGGAAUCGGUAAAACAAU | Cleavage |
| ccp-miR1510b-3p | Cc03_g06200 | 2.5 | 19.189 | 1 | 24 | 1864 | 1887 | GUUGUUUUACCUAUUCCACUCAUU | AAUGGGUGGAAUCGGCAAAACAAC | Translation |
| ccp-miR1510b-3p | Cc03_g09200 | 2.5 | 14.451 | 1 | 20 | 1744 | 1763 | GUUGUUUUACCUAUUCCACU | CCUGGAAUAGGUAAGACAAC | Cleavage |
| ccp-miR1510b-3p | Cc03_g14580 | 3.0 | 21.245 | 1 | 24 | 633 | 656 | GUUGUUUUACCUAUUCCACUCAUU | AAUGGGUGGAUUGGGGAAAACAAC | Translation |
| ccp-miR1510b-3p | Cc04_g03730 | 2.5 | 19.798 | 1 | 23 | 2608 | 2630 | GUUGUUUUACCUAUUCCACUCAU | GUUAGUGGAGCCGGUAAAACAAC | Cleavage |
| ccp-miR1510b-3p | Cc04_g03740 | 2.5 | 19.798 | 1 | 23 | 2973 | 2995 | GUUGUUUUACCUAUUCCACUCAU | GUUAGUGGAGCCGGUAAAACAAC | Cleavage |
| ccp-miR1510b-3p | Cc04_g10100 | 3.0 | 18.537 | 1 | 24 | 726 | 749 | GUUGUUUUACCUAUUCCACUCAUU | AAAGGGUGGAAUAGGCAAAAUGAU | Translation |
| ccp-miR1510b-3p | Cc05_g06490 | 2.5 | 12.225 | 1 | 24 | 420 | 443 | GUUGUUUUACCUAUUCCACUCAUU | GAUGGGUGGCGUGGGUAAAACAAC | Cleavage |
| ccp-miR1510b-3p | Cc07_g16580 | 2.5 | 16.377 | 1 | 20 | 3672 | 3691 | GUUGUUUUACCUAUUCCACU | AGUGGGAUGGGUGAAGCAGC | Cleavage |
| ccp-miR1510b-3p | Cc07_g17810 | 3.0 | 13.86 | 1 | 23 | 1690 | 1712 | GUUGUUUUACCUAUUCCACUCAU | AUGGGUGGACUUGGUAAGACAAC | Cleavage |
| ccp-miR1510b-3p | Cc08_g06850 | 1.0 | 23.294 | 1 | 24 | 678 | 701 | GUUGUUUUACCUAUUCCACUCAUU | AAUGAGUGGAAUAGGUAAGACGAC | Cleavage |
| ccp-miR1510b-3p | Cc10_g07420 | 3.0 | 17.959 | 1 | 20 | 780 | 799 | GUUGUUUUACCUAUUCCACU | UCUGGAAGAGGUAAAACAAC | Cleavage |
| ccp-miR1510b-3p | Cc11_g01160 | 3.0 | 16.409 | 1 | 24 | 648 | 671 | GUUGUUUUACCUAUUCCACUCAUU | AAUGCCUGGAAUCGGUAAAACAAC | Cleavage |
| ccp-miR1510b-3p | Cc11_g01970 | 2.5 | 24.317 | 1 | 23 | 937 | 959 | GUUGUUUUACCUAUUCCACUCAU | AUGGGUGGUAUAGGCAAAACAAC | Translation |
| ccp-miR1510b-3p | Cc11_g02040 | 3.0 | 19.21 | 1 | 23 | 598 | 620 | GUUGUUUUACCUAUUCCACUCAU | AUGGGUGGUAUAGGCAAAACGAC | Translation |
| ccp-miR1510b-3p | Cc11_g02900 | 3.0 | 21.945 | 1 | 23 | 586 | 608 | GUUGUUUUACCUAUUCCACUCAU | AUGGGUGGUAUAGGCAAAACGAC | Translation |
| ccp-miR1510b-3p | Cc11_g06550 | 1.0 | 19.36 | 1 | 24 | 549 | 572 | GUUGUUUUACCUAUUCCACUCAUU | GAUGGGUGGAAUAGGUAAGACAAC | Cleavage |
| ccp-miR1510b-3p | Cc11_g07560 | 3.0 | 18.658 | 1 | 23 | 703 | 725 | GUUGUUUUACCUAUUCCACUCAU | AUGCCAGGAAUAGGUAAAACAAC | Cleavage |
| ccp-miR1510b-3p | Cc11_g08750 | 3.0 | 20.35 | 1 | 24 | 1728 | 1751 | GUUGUUUUACCUAUUCCACUCAUU | AAUGCCUGGAGUAGGUAAGACAAC | Cleavage |
| ccp-miR1510b-3p | Cc11_g08760 | 3.0 | 21.208 | 1 | 23 | 1750 | 1772 | GUUGUUUUACCUAUUCCACUCAU | AUGCCUGGAGUAGGUAAGACAAC | Cleavage |
| ccp-miR1510b-3p | Cc11_g08970 | 2.5 | 18.022 | 1 | 24 | 1088 | 1111 | GUUGUUUUACCUAUUCCACUCAUU | GAUGGGUGGCAUUGGUAAAACAAC | Cleavage |
| ccp-miR1515a-3p | Cc00_g16310 | 3.0 | 15.373 | 1 | 20 | 523 | 542 | UCAUUUUGUGUGCCAUGAUC | UAUUAUGGCAUCCAAAAUGA | Translation |
| ccp-miR1515a-3p | Cc00_g19560 | 3.0 | 20.417 | 1 | 22 | 908 | 929 | UCAUUUUGUGUGCCAUGAUCCA | UGGAUCAUAGCAUAAAGAAUGA | Cleavage |
| ccp-miR1515a-3p | Cc02_g07970 | 3.0 | 19.806 | 1 | 20 | 1545 | 1564 | UCAUUUUGUGUGCCAUGAUC | GAUCUUUGCAUACAGAAUGA | Cleavage |
| ccp-miR1515a-3p | Cc02_g32270 | 3.0 | 23.368 | 1 | 20 | 174 | 193 | UCAUUUUGUGUGCCAUGAUC | GGUCAUGGCACACCAGAUGG | Cleavage |
| ccp-miR1515a-3p | Cc04_g15940 | 3.0 | 12.72 | 1 | 20 | 697 | 716 | UCAUUUUGUGUGCCAUGAUC | GAUUGUGGCACACAAGAUUA | Cleavage |
| ccp-miR1515a-3p | Cc06_g20540 | 3.0 | 12.642 | 1 | 20 | 691 | 710 | UCAUUUUGUGUGCCAUGAUC | GAUUGUGGCACACAAGAUUA | Cleavage |
| ccp-miR1515a-3p | Cc07_g19540 | 3.0 | 19.1 | 1 | 20 | 1457 | 1476 | UCAUUUUGUGUGCCAUGAUC | GAUUAUGACACUCAAAGUGA | Translation |
| ccp-miR1515a-3p | Cc10_g11320 | 2.5 | 18.165 | 1 | 22 | 899 | 920 | UCAUUUUGUGUGCCAUGAUCCA | UUGAUUACGGUACACAAAAUGG | Cleavage |
| ccp-miR1515a-3p | Cc11_g01750 | 3.0 | 19.763 | 1 | 22 | 996 | 1017 | UCAUUUUGUGUGCCAUGAUCCA | UGGAUCAUAGCAUAAAGAAUGA | Cleavage |
| ccp-miR1520e-5p | Cc00_g00110 | 3.0 | 16.384 | 1 | 23 | 1384 | 1406 | CAAUAAGACCAUGAGAUUUGACA | UUUCAUAUCUUAAGGUCUUGUUG | Translation |
| ccp-miR1520e-5p | Cc00_g02390 | 3.0 | 17.917 | 1 | 24 | 524 | 547 | CAAUAAGACCAUGAGAUUUGACAG | UUGGCGAAUCUCAUCGUCAUAUUG | Translation |
| ccp-miR1520e-5p | Cc00_g08660 | 2.5 | 20.36 | 1 | 20 | 3386 | 3405 | CAAUAAGACCAUGAGAUUUG | CAACUCUUAUGGUCUUAUUC | Cleavage |
| ccp-miR1520e-5p | Cc00_g27280 | 3.0 | 17.541 | 1 | 20 | 1629 | 1648 | CAAUAAGACCAUGAGAUUUG | CAAGUCUUCUGGACUUAUUG | Cleavage |
| ccp-miR1520e-5p | Cc03_g14180 | 3.0 | 11.528 | 1 | 22 | 1565 | 1586 | CAAUAAGACCAUGAGAUUUGAC | GACGAAUAUCAUGUUUUUAUUG | Translation |
| ccp-miR1526-5p | Cc02_g09180 | 3.0 | 16.728 | 1 | 20 | 75 | 94 | CCGGAAGAAGCAAAUUAAGU | AUUUGAUUUGCUUCGUCUGG | Cleavage |
| ccp-miR1526-5p | Cc04_g06580 | 3.0 | 19.829 | 1 | 22 | 1254 | 1276 | CCGGAAGAAGC-AAAUUAAGUAA | UGGCUUGAUUUUGCUUCUUUCGG | Cleavage |
| ccp-miR1526-5p | Cc09_g04110 | 2.5 | 11.009 | 1 | 20 | 2704 | 2723 | CCGGAAGAAGCAAAUUAAGU | ACUGUAUUUGUUUCUUCCGG | Cleavage |
| ccp-miR156-5p | Cc02_g13600 | 2.0 | 13.696 | 1 | 21 | 737 | 757 | UGACAGAAGAGAGUGAGCACA | UAUGCUCUCUCUCUUCUGUCA | Cleavage |
| ccp-miR156-5p | Cc02_g24550 | 1.0 | 21.498 | 1 | 21 | 958 | 978 | UGACAGAAGAGAGUGAGCACA | UGUGCUCUCUCUCUUCUGUCA | Cleavage |
| ccp-miR156-5p | Cc05_g07500 | 2.0 | 16.449 | 1 | 21 | 1027 | 1047 | UGACAGAAGAGAGUGAGCACA | UUUGCUCUCUCUCUUCUGUCA | Cleavage |
| ccp-miR156-5p | Cc05_g11850 | 1.5 | 15.185 | 1 | 20 | 998 | 1017 | UGACAGAAGAGAGUGAGCAC | UUGCUUACUCUCUUCUGUCA | Cleavage |
| ccp-miR156-5p | Cc06_g21540 | 3.0 | 10.983 | 1 | 20 | 16 | 35 | UGACAGAAGAGAGUGAGCAC | CUGCUCAUGCUUUUCUGUCA | Cleavage |
| ccp-miR156-5p | Cc06_g23710 | 1.0 | 14.918 | 1 | 21 | 1354 | 1374 | UGACAGAAGAGAGUGAGCACA | UGUGCUCUCUCUCUUCUGUCA | Cleavage |
| ccp-miR156-5p | Cc07_g01140 | 3.0 | 12.684 | 1 | 21 | 596 | 616 | UGACAGAAGAGAGUGAGCACA | UGCGCUCUCUCUUUUCUGUCG | Cleavage |
| ccp-miR156-5p | Cc11_g11740 | 1.0 | 15.162 | 1 | 20 | 1293 | 1312 | UGACAGAAGAGAGUGAGCAC | GUGCUCUCUCUCUUCUGUCA | Cleavage |
| ccp-miR156-5p | Cc11_g15480 | 3.0 | 17.287 | 1 | 21 | 1077 | 1097 | UGACAGAAGAGAGUGAGCACA | UGUGUUCUCUAUUUUCUGUCA | Translation |
| ccp-miR156-5p | Cc11_g16990 | 1.0 | 17.813 | 1 | 20 | 1349 | 1368 | UGACAGAAGAGAGUGAGCAC | GUGCUCUCUCUCUUCUGUCA | Cleavage |
| ccp-miR156-5p | Cc11_g17130 | 1.0 | 12.9 | 1 | 20 | 1053 | 1072 | UGACAGAAGAGAGUGAGCAC | GUGCUCUCUCUCUUCUGUCA | Cleavage |
| ccp-miR156f-5p | Cc02_g13600 | 0.0 | 13.556 | 1 | 22 | 737 | 758 | UUGACAGAAGAGAGAGAGCACA | UAUGCUCUCUCUCUUCUGUCAA | Cleavage |
| ccp-miR156f-5p | Cc02_g24550 | 1.0 | 21.591 | 1 | 22 | 958 | 979 | UUGACAGAAGAGAGAGAGCACA | UGUGCUCUCUCUCUUCUGUCAU | Cleavage |
| ccp-miR156f-5p | Cc02_g34300 | 3.0 | 3.186 | 1 | 22 | 186 | 207 | UUGACAGAAGAGAGAGAGCACA | UUUUCUCUCUCUUUACUGUCAG | Cleavage |
| ccp-miR156f-5p | Cc02_g39180 | 3.0 | 20.902 | 1 | 21 | 738 | 758 | UUGACAGAAGAGAGAGAGCAC | GUGCUCUUCCUCUGUUGUCAA | Cleavage |
| ccp-miR156f-5p | Cc03_g06400 | 3.0 | 7.432 | 1 | 19 | 144 | 163 | UUGACAGAAGAGAG-AGAGC | CCUCUACUCUCUUCUGUCAA | Cleavage |
| ccp-miR156f-5p | Cc04_g02130 | 3.0 | 16.823 | 1 | 21 | 813 | 833 | UUGACAGAAGAGAGAGAGCAC | GUGCCCUCUUUCUUCUGUCCA | Cleavage |
| ccp-miR156f-5p | Cc05_g07500 | 1.0 | 16.576 | 1 | 22 | 1027 | 1048 | UUGACAGAAGAGAGAGAGCACA | UUUGCUCUCUCUCUUCUGUCAU | Cleavage |
| ccp-miR156f-5p | Cc05_g11850 | 2.5 | 15.106 | 1 | 20 | 999 | 1018 | UUGACAGAAGAGAGAGAGCA | UGCUUACUCUCUUCUGUCAU | Cleavage |
| ccp-miR156f-5p | Cc05_g15990 | 3.0 | 14.462 | 1 | 20 | 580 | 599 | UUGACAGAAGAGAGAGAGCA | UGGUCUAUCUUUUUUGUCAA | Cleavage |
| ccp-miR156f-5p | Cc06_g05440 | 3.0 | 17.875 | 1 | 20 | 2374 | 2393 | UUGACAGAAGAGAGAGAGCA | UGCUUUCUCUCCUUUGUUAG | Translation |
| ccp-miR156f-5p | Cc06_g23710 | 0.0 | 15.544 | 1 | 22 | 1354 | 1375 | UUGACAGAAGAGAGAGAGCACA | UGUGCUCUCUCUCUUCUGUCAA | Cleavage |
| ccp-miR156f-5p | Cc07_g01140 | 2.0 | 11.93 | 1 | 22 | 596 | 617 | UUGACAGAAGAGAGAGAGCACA | UGCGCUCUCUCUUUUCUGUCGA | Cleavage |
| ccp-miR156f-5p | Cc07_g11070 | 3.0 | 3.221 | 1 | 22 | 269 | 290 | UUGACAGAAGAGAGAGAGCACA | UUUUCUCUCUCUUUACUGUCAG | Cleavage |
| ccp-miR156f-5p | Cc08_g02820 | 3.0 | 2.936 | 1 | 20 | 30 | 49 | UUGACAGAAGAGAGAGAGCA | UCCUCUGUCUCUUCUGUCAC | Cleavage |
| ccp-miR156f-5p | Cc08_g05960 | 3.0 | 18.084 | 1 | 21 | 813 | 833 | UUGACAGAAGAGAGAGAGCAC | GCGCGCUCCCUCUUCUGUCAA | Cleavage |
| ccp-miR156f-5p | Cc08_g09340 | 3.0 | 11.02 | 1 | 20 | 31 | 50 | UUGACAGAAGAGAGAGAGCA | UGUUCUUUCUCUUUUGUCUA | Cleavage |
| ccp-miR156f-5p | Cc09_g01870 | 3.0 | 17.925 | 1 | 22 | 1229 | 1250 | UUGACAGAAGAGAGAGAGCACA | UGUGGUGUUUCUCUUUUGUCAA | Cleavage |
| ccp-miR156f-5p | Cc11_g08040 | 2.5 | 17.717 | 1 | 20 | 1260 | 1279 | UUGACAGAAGAGAGAGAGCA | UGUUUUCUCUCAUCUGUUAA | Translation |
| ccp-miR156f-5p | Cc11_g11740 | 0.0 | 14.412 | 1 | 21 | 1293 | 1313 | UUGACAGAAGAGAGAGAGCAC | GUGCUCUCUCUCUUCUGUCAA | Cleavage |
| ccp-miR156f-5p | Cc11_g15480 | 2.0 | 16.788 | 1 | 22 | 1077 | 1098 | UUGACAGAAGAGAGAGAGCACA | UGUGUUCUCUAUUUUCUGUCAA | Cleavage |
| ccp-miR156f-5p | Cc11_g16990 | 0.0 | 18.551 | 1 | 21 | 1349 | 1369 | UUGACAGAAGAGAGAGAGCAC | GUGCUCUCUCUCUUCUGUCAA | Cleavage |
| ccp-miR156f-5p | Cc11_g17130 | 1.0 | 12.427 | 1 | 21 | 1053 | 1073 | UUGACAGAAGAGAGAGAGCAC | GUGCUCUCUCUCUUCUGUCAU | Cleavage |
| ccp-miR156g-5p | Cc02_g13600 | 3.0 | 13.696 | 1 | 20 | 738 | 757 | CGACAGAAGAGAGUGAGCAC | AUGCUCUCUCUCUUCUGUCA | Cleavage |
| ccp-miR156g-5p | Cc02_g24550 | 2.0 | 21.498 | 1 | 20 | 959 | 978 | CGACAGAAGAGAGUGAGCAC | GUGCUCUCUCUCUUCUGUCA | Cleavage |
| ccp-miR156g-5p | Cc05_g07500 | 3.0 | 16.449 | 1 | 20 | 1028 | 1047 | CGACAGAAGAGAGUGAGCAC | UUGCUCUCUCUCUUCUGUCA | Cleavage |
| ccp-miR156g-5p | Cc05_g11850 | 2.5 | 15.185 | 1 | 20 | 998 | 1017 | CGACAGAAGAGAGUGAGCAC | UUGCUUACUCUCUUCUGUCA | Cleavage |
| ccp-miR156g-5p | Cc06_g23710 | 2.0 | 14.918 | 1 | 20 | 1355 | 1374 | CGACAGAAGAGAGUGAGCAC | GUGCUCUCUCUCUUCUGUCA | Cleavage |
| ccp-miR156g-5p | Cc07_g01140 | 2.5 | 12.684 | 1 | 20 | 597 | 616 | CGACAGAAGAGAGUGAGCAC | GCGCUCUCUCUUUUCUGUCG | Cleavage |
| ccp-miR156g-5p | Cc07_g08950 | 3.0 | 15.528 | 1 | 20 | 772 | 791 | CGACAGAAGAGAGUGAGCAC | GUGUGCAAUCUUUUCUGUCG | Cleavage |
| ccp-miR156g-5p | Cc11_g11740 | 2.0 | 15.162 | 1 | 20 | 1293 | 1312 | CGACAGAAGAGAGUGAGCAC | GUGCUCUCUCUCUUCUGUCA | Cleavage |
| ccp-miR156g-5p | Cc11_g16990 | 2.0 | 17.813 | 1 | 20 | 1349 | 1368 | CGACAGAAGAGAGUGAGCAC | GUGCUCUCUCUCUUCUGUCA | Cleavage |
| ccp-miR156g-5p | Cc11_g17130 | 2.0 | 12.9 | 1 | 20 | 1053 | 1072 | CGACAGAAGAGAGUGAGCAC | GUGCUCUCUCUCUUCUGUCA | Cleavage |
| ccp-miR157-5p | Cc00_g17180 | 3.0 | 15.577 | 1 | 20 | 3325 | 3344 | UUGACAGAAGAUAGAGAGCA | UACUCUCUAUCUUCUGUUGU | Cleavage |
| ccp-miR157-5p | Cc02_g09800 | 3.0 | 15.688 | 1 | 20 | 2517 | 2535 | UUGACAGAAGAUAGAGAGCA | UGUUCUUUAU-UUCUGUCAA | Translation |
| ccp-miR157-5p | Cc02_g13600 | 1.0 | 13.556 | 1 | 20 | 739 | 758 | UUGACAGAAGAUAGAGAGCA | UGCUCUCUCUCUUCUGUCAA | Cleavage |
| ccp-miR157-5p | Cc02_g24550 | 2.0 | 21.591 | 1 | 21 | 959 | 979 | UUGACAGAAGAUAGAGAGCAC | GUGCUCUCUCUCUUCUGUCAU | Cleavage |
| ccp-miR157-5p | Cc05_g07500 | 2.0 | 16.576 | 1 | 20 | 1029 | 1048 | UUGACAGAAGAUAGAGAGCA | UGCUCUCUCUCUUCUGUCAU | Cleavage |
| ccp-miR157-5p | Cc06_g23710 | 1.0 | 15.544 | 1 | 21 | 1355 | 1375 | UUGACAGAAGAUAGAGAGCAC | GUGCUCUCUCUCUUCUGUCAA | Cleavage |
| ccp-miR157-5p | Cc07_g01140 | 3.0 | 11.93 | 1 | 21 | 597 | 617 | UUGACAGAAGAUAGAGAGCAC | GCGCUCUCUCUUUUCUGUCGA | Cleavage |
| ccp-miR157-5p | Cc11_g11740 | 1.0 | 14.412 | 1 | 21 | 1293 | 1313 | UUGACAGAAGAUAGAGAGCAC | GUGCUCUCUCUCUUCUGUCAA | Cleavage |
| ccp-miR157-5p | Cc11_g15480 | 1.0 | 16.788 | 1 | 21 | 1078 | 1098 | UUGACAGAAGAUAGAGAGCAC | GUGUUCUCUAUUUUCUGUCAA | Cleavage |
| ccp-miR157-5p | Cc11_g16990 | 1.0 | 18.551 | 1 | 21 | 1349 | 1369 | UUGACAGAAGAUAGAGAGCAC | GUGCUCUCUCUCUUCUGUCAA | Cleavage |
| ccp-miR157-5p | Cc11_g17130 | 2.0 | 12.427 | 1 | 21 | 1053 | 1073 | UUGACAGAAGAUAGAGAGCAC | GUGCUCUCUCUCUUCUGUCAU | Cleavage |
| ccp-miR157a-1-3p | Cc05_g09340 | 3.0 | 17.451 | 1 | 20 | 187 | 206 | GCUCUCUAGUCUUCUGUCAU | CUGAGAGAAGGCUGGAGAGC | Cleavage |
| ccp-miR157a-1-3p | Cc06_g03440 | 3.0 | 19.87 | 1 | 21 | 13 | 33 | GCUCUCUAGUCUUCUGUCAUC | GGUGACUGGAGACUGGAGAGU | Cleavage |
| ccp-miR157a-1-3p | Cc06_g14510 | 3.0 | 20.093 | 1 | 21 | 2683 | 2703 | GCUCUCUAGUCUUCUGUCAUC | GAUGACAGUAGACUAGAUAGU | Cleavage |
| ccp-miR157a-1-3p | Cc07_g12880 | 3.0 | 15.474 | 1 | 21 | 1179 | 1199 | GCUCUCUAGUCUUCUGUCAUC | GCUGAGAGAAGGCUGGAGAGC | Cleavage |
| ccp-miR157a-1-5p | Cc00_g17180 | 3.0 | 15.577 | 1 | 20 | 3325 | 3344 | UUGACAGAAGAUAGAGAGCA | UACUCUCUAUCUUCUGUUGU | Cleavage |
| ccp-miR157a-1-5p | Cc02_g09800 | 3.0 | 15.688 | 1 | 20 | 2517 | 2535 | UUGACAGAAGAUAGAGAGCA | UGUUCUUUAU-UUCUGUCAA | Translation |
| ccp-miR157a-1-5p | Cc02_g13600 | 1.0 | 13.556 | 1 | 20 | 739 | 758 | UUGACAGAAGAUAGAGAGCA | UGCUCUCUCUCUUCUGUCAA | Cleavage |
| ccp-miR157a-1-5p | Cc02_g24550 | 2.0 | 21.591 | 1 | 21 | 959 | 979 | UUGACAGAAGAUAGAGAGCAC | GUGCUCUCUCUCUUCUGUCAU | Cleavage |
| ccp-miR157a-1-5p | Cc05_g07500 | 2.0 | 16.576 | 1 | 20 | 1029 | 1048 | UUGACAGAAGAUAGAGAGCA | UGCUCUCUCUCUUCUGUCAU | Cleavage |
| ccp-miR157a-1-5p | Cc06_g23710 | 1.0 | 15.544 | 1 | 21 | 1355 | 1375 | UUGACAGAAGAUAGAGAGCAC | GUGCUCUCUCUCUUCUGUCAA | Cleavage |
| ccp-miR157a-1-5p | Cc07_g01140 | 3.0 | 11.93 | 1 | 21 | 597 | 617 | UUGACAGAAGAUAGAGAGCAC | GCGCUCUCUCUUUUCUGUCGA | Cleavage |
| ccp-miR157a-1-5p | Cc11_g11740 | 1.0 | 14.412 | 1 | 21 | 1293 | 1313 | UUGACAGAAGAUAGAGAGCAC | GUGCUCUCUCUCUUCUGUCAA | Cleavage |
| ccp-miR157a-1-5p | Cc11_g15480 | 1.0 | 16.788 | 1 | 21 | 1078 | 1098 | UUGACAGAAGAUAGAGAGCAC | GUGUUCUCUAUUUUCUGUCAA | Cleavage |
| ccp-miR157a-1-5p | Cc11_g16990 | 1.0 | 18.551 | 1 | 21 | 1349 | 1369 | UUGACAGAAGAUAGAGAGCAC | GUGCUCUCUCUCUUCUGUCAA | Cleavage |
| ccp-miR157a-1-5p | Cc11_g17130 | 2.0 | 12.427 | 1 | 21 | 1053 | 1073 | UUGACAGAAGAUAGAGAGCAC | GUGCUCUCUCUCUUCUGUCAU | Cleavage |
| ccp-miR157a-2-5p | Cc00_g17180 | 3.0 | 15.577 | 1 | 20 | 3325 | 3344 | UUGACAGAAGAUAGAGAGCA | UACUCUCUAUCUUCUGUUGU | Cleavage |
| ccp-miR157a-2-5p | Cc02_g09800 | 3.0 | 15.688 | 1 | 20 | 2517 | 2535 | UUGACAGAAGAUAGAGAGCA | UGUUCUUUAU-UUCUGUCAA | Translation |
| ccp-miR157a-2-5p | Cc02_g13600 | 1.0 | 13.556 | 1 | 20 | 739 | 758 | UUGACAGAAGAUAGAGAGCA | UGCUCUCUCUCUUCUGUCAA | Cleavage |
| ccp-miR157a-2-5p | Cc02_g24550 | 2.0 | 21.591 | 1 | 21 | 959 | 979 | UUGACAGAAGAUAGAGAGCAC | GUGCUCUCUCUCUUCUGUCAU | Cleavage |
| ccp-miR157a-2-5p | Cc05_g07500 | 2.0 | 16.576 | 1 | 20 | 1029 | 1048 | UUGACAGAAGAUAGAGAGCA | UGCUCUCUCUCUUCUGUCAU | Cleavage |
| ccp-miR157a-2-5p | Cc06_g23710 | 1.0 | 15.544 | 1 | 21 | 1355 | 1375 | UUGACAGAAGAUAGAGAGCAC | GUGCUCUCUCUCUUCUGUCAA | Cleavage |
| ccp-miR157a-2-5p | Cc07_g01140 | 3.0 | 11.93 | 1 | 21 | 597 | 617 | UUGACAGAAGAUAGAGAGCAC | GCGCUCUCUCUUUUCUGUCGA | Cleavage |
| ccp-miR157a-2-5p | Cc11_g11740 | 1.0 | 14.412 | 1 | 21 | 1293 | 1313 | UUGACAGAAGAUAGAGAGCAC | GUGCUCUCUCUCUUCUGUCAA | Cleavage |
| ccp-miR157a-2-5p | Cc11_g15480 | 1.0 | 16.788 | 1 | 21 | 1078 | 1098 | UUGACAGAAGAUAGAGAGCAC | GUGUUCUCUAUUUUCUGUCAA | Cleavage |
| ccp-miR157a-2-5p | Cc11_g16990 | 1.0 | 18.551 | 1 | 21 | 1349 | 1369 | UUGACAGAAGAUAGAGAGCAC | GUGCUCUCUCUCUUCUGUCAA | Cleavage |
| ccp-miR157a-2-5p | Cc11_g17130 | 2.0 | 12.427 | 1 | 21 | 1053 | 1073 | UUGACAGAAGAUAGAGAGCAC | GUGCUCUCUCUCUUCUGUCAU | Cleavage |
| ccp-miR159a-3p | Cc00_g15150 | 3.0 | 19.353 | 1 | 20 | 821 | 839 | UUUGGAUUGAAGGGAGCUCU | AGAGCUCUCU-CAAUUCAAA | Translation |
| ccp-miR159a-3p | Cc00_g15430 | 3.0 | 19.104 | 1 | 21 | 1609 | 1629 | UUUGGAUUGAAGGGAGCUCUA | UUGAGCUUUUUUCAAUCCAGA | Cleavage |
| ccp-miR159a-3p | Cc00_g25690 | 3.0 | 21.369 | 1 | 21 | 1894 | 1914 | UUUGGAUUGAAGGGAGCUCUA | UUGAGCAUCUUUCAAUCCAAA | Cleavage |
| ccp-miR159a-3p | Cc00_g32840 | 2.5 | 20.296 | 1 | 21 | 1186 | 1206 | UUUGGAUUGAAGGGAGCUCUA | UUGAGCUUCUUUCAAUCCAGA | Cleavage |
| ccp-miR159a-3p | Cc01_g07440 | 2.0 | 22.12 | 1 | 21 | 1327 | 1347 | UUUGGAUUGAAGGGAGCUCUA | UGGAGCUCCCUUCACUCCAAA | Cleavage |
| ccp-miR159a-3p | Cc01_g13090 | 2.5 | 19.31 | 1 | 21 | 284 | 304 | UUUGGAUUGAAGGGAGCUCUA | UGGAGCUCCCUUCACUUCAAA | Cleavage |
| ccp-miR159a-3p | Cc01_g17950 | 3.0 | 15.793 | 1 | 20 | 513 | 532 | UUUGGAUUGAAGGGAGCUCU | AGAGUUACAUUCAAUUCAAA | Cleavage |
| ccp-miR159a-3p | Cc02_g24840 | 2.0 | 22.197 | 1 | 21 | 121 | 141 | UUUGGAUUGAAGGGAGCUCUA | UGGAGCUCCAUUCGAUCCAAA | Cleavage |
| ccp-miR159a-3p | Cc03_g14760 | 2.0 | 22.168 | 1 | 21 | 2516 | 2536 | UUUGGAUUGAAGGGAGCUCUA | UUGAGCUUCUUUCAAUCCAAA | Cleavage |
| ccp-miR159a-3p | Cc04_g03940 | 3.0 | 20.005 | 1 | 20 | 1921 | 1940 | UUUGGAUUGAAGGGAGCUCU | UGGGUUCUCUUUAAUCCAAA | Cleavage |
| ccp-miR159a-3p | Cc04_g06650 | 0.0 | 17.871 | 1 | 21 | 704 | 724 | UUUGGAUUGAAGGGAGCUCUA | UAGAGCUCCCUUCAAUCCAAA | Cleavage |
| ccp-miR159a-3p | Cc05_g11450 | 3.0 | 7.78 | 1 | 21 | 164 | 184 | UUUGGAUUGAAGGGAGCUCUA | UGGAGCCCCCUUCAAACCAAA | Cleavage |
| ccp-miR159a-3p | Cc05_g13920 | 1.5 | 13.906 | 1 | 20 | 1296 | 1315 | UUUGGAUUGAAGGGAGCUCU | AGAGCCCCCUUCAGUCCAAA | Cleavage |
| ccp-miR159a-3p | Cc06_g02810 | 3.0 | 22.627 | 1 | 21 | 165 | 185 | UUUGGAUUGAAGGGAGCUCUA | UAGGACUCCUCUCAAUCCAAA | Translation |
| ccp-miR159a-3p | Cc06_g19740 | 3.0 | 21.111 | 1 | 21 | 1825 | 1845 | UUUGGAUUGAAGGGAGCUCUA | UUGAGCCUCUUUCAAUCCAAA | Cleavage |
| ccp-miR159a-3p | Cc07_g11950 | 3.0 | 17.319 | 1 | 21 | 2626 | 2646 | UUUGGAUUGAAGGGAGCUCUA | UGGAGCUGUUUUCGAUCCAAA | Cleavage |
| ccp-miR159a-3p | Cc08_g12080 | 2.0 | 20.695 | 1 | 21 | 103 | 123 | UUUGGAUUGAAGGGAGCUCUA | UGGAGCUCCAUUCGAUCCAAA | Cleavage |
| ccp-miR159a-5p | Cc02_g39320 | 3.0 | 18.403 | 1 | 21 | 1298 | 1318 | GAGCUCCUUGAAGUCCAAUAG | CUAUUGCAGUUCAUGGAGCUC | Cleavage |
| ccp-miR159a-5p | Cc04_g06650 | 0.0 | 12.958 | 1 | 21 | 857 | 877 | GAGCUCCUUGAAGUCCAAUAG | CUAUUGGACUUCAAGGAGCUC | Cleavage |
| ccp-miR159a-5p | Cc07_g18420 | 3.0 | 19.761 | 1 | 21 | 413 | 433 | GAGCUCCUUGAAGUCCAAUAG | UUGUUGGACUUCCAGGAGUUG | Translation |
| ccp-miR159a-5p | Cc08_g00250 | 2.5 | 20.4 | 1 | 20 | 1134 | 1153 | GAGCUCCUUGAAGUCCAAUA | UACUGGACUUCUAGGAGUUC | Translation |
| ccp-miR159a-5p | Cc11_g05050 | 3.0 | 23.379 | 1 | 21 | 493 | 513 | GAGCUCCUUGAAGUCCAAUAG | UUAUUGCUUUUUAAGGAGCUC | Cleavage |
| ccp-miR159b-3p | Cc00_g04720 | 3.0 | 20.982 | 1 | 20 | 616 | 635 | UUGGAGUGAAGGGAGCUCCA | UGGAGCUUUUUACACUCCAG | Translation |
| ccp-miR159b-3p | Cc01_g07440 | 0.0 | 21.661 | 1 | 21 | 1326 | 1346 | UUGGAGUGAAGGGAGCUCCAG | CUGGAGCUCCCUUCACUCCAA | Cleavage |
| ccp-miR159b-3p | Cc01_g13090 | 0.5 | 18.959 | 1 | 21 | 283 | 303 | UUGGAGUGAAGGGAGCUCCAG | CUGGAGCUCCCUUCACUUCAA | Cleavage |
| ccp-miR159b-3p | Cc02_g24840 | 3.0 | 22.34 | 1 | 21 | 120 | 140 | UUGGAGUGAAGGGAGCUCCAG | CUGGAGCUCCAUUCGAUCCAA | Translation |
| ccp-miR159b-3p | Cc02_g33740 | 3.0 | 16.566 | 1 | 20 | 268 | 287 | UUGGAGUGAAGGGAGCUCCA | GUGAGCUUCCUUCGCUCCAA | Cleavage |
| ccp-miR159b-3p | Cc02_g36110 | 2.5 | 18.648 | 1 | 21 | 2207 | 2227 | UUGGAGUGAAGGGAGCUCCAG | UUGGCGCUUCCUUUAUUCCAA | Cleavage |
| ccp-miR159b-3p | Cc04_g06650 | 2.5 | 17.825 | 1 | 20 | 704 | 723 | UUGGAGUGAAGGGAGCUCCA | UAGAGCUCCCUUCAAUCCAA | Cleavage |
| ccp-miR159b-3p | Cc06_g15350 | 3.0 | 14.4 | 1 | 20 | 1464 | 1483 | UUGGAGUGAAGGGAGCUCCA | UGGAGCUGACUUCACUUCGA | Cleavage |
| ccp-miR159b-3p | Cc07_g02320 | 2.5 | 16.959 | 1 | 20 | 1784 | 1803 | UUGGAGUGAAGGGAGCUCCA | UUGAGCUAUCUUCACUCCAA | Cleavage |
| ccp-miR159b-3p | Cc08_g12080 | 3.0 | 20.718 | 1 | 21 | 102 | 122 | UUGGAGUGAAGGGAGCUCCAG | CUGGAGCUCCAUUCGAUCCAA | Translation |
| ccp-miR159b-3p | Cc10_g10450 | 3.0 | 10.694 | 1 | 21 | 2111 | 2131 | UUGGAGUGAAGGGAGCUCCAG | CUGCAGCAUCCUUCACUUCAA | Cleavage |
| ccp-miR160-3p | Cc11_g16830 | 2.0 | 13.667 | 1 | 20 | 2118 | 2137 | GCGUAUGAGGAGCCAUGCAU | AUGCAUGUUUUCUCAUACGC | Cleavage |
| ccp-miR160-5p | Cc01_g11020 | 0.0 | 21.799 | 1 | 20 | 1309 | 1328 | UGCCUGGCUCCCUGUAUGCC | GGCAUACAGGGAGCCAGGCA | Cleavage |
| ccp-miR160-5p | Cc02_g11300 | 0.0 | 22.211 | 1 | 20 | 1886 | 1905 | UGCCUGGCUCCCUGUAUGCC | GGCAUACAGGGAGCCAGGCA | Cleavage |
| ccp-miR160a-1-3p | Cc01_g01000 | 3.0 | 18.146 | 1 | 21 | 1865 | 1885 | GCAUCAGAGGAGUCAGGCAGG | CCUGCUUGAUUCCUUUGCUGC | Cleavage |
| ccp-miR160a-1-3p | Cc01_g19440 | 3.0 | 18.379 | 1 | 21 | 1872 | 1892 | GCAUCAGAGGAGUCAGGCAGG | CUUGCUUGCUUCUUCUGAUGC | Cleavage |
| ccp-miR160a-1-3p | Cc02_g20590 | 3.0 | 17.85 | 1 | 21 | 2559 | 2579 | GCAUCAGAGGAGUCAGGCAGG | UCUUCUUGACUCCUCUGGUGA | Cleavage |
| ccp-miR160a-1-3p | Cc03_g03740 | 3.0 | 23.192 | 1 | 21 | 4385 | 4405 | GCAUCAGAGGAGUCAGGCAGG | UCUUUCUGAUUCUUCUGGUGC | Cleavage |
| ccp-miR160a-1-3p | Cc03_g09900 | 2.0 | 20.397 | 1 | 21 | 321 | 341 | GCAUCAGAGGAGUCAGGCAGG | CCUGCUUGAUACCUCUGAUGC | Translation |
| ccp-miR160a-1-3p | Cc05_g15020 | 3.0 | 20.224 | 1 | 21 | 704 | 724 | GCAUCAGAGGAGUCAGGCAGG | UCUGCUUGCUUCCACUGAUGC | Cleavage |
| ccp-miR160a-1-3p | Cc05_g15750 | 3.0 | 12.035 | 1 | 20 | 4 | 23 | GCAUCAGAGGAGUCAGGCAG | CUGGCUGGUUCAUCUGAUGC | Translation |
| ccp-miR160a-1-3p | Cc06_g07870 | 3.0 | 16.774 | 1 | 20 | 2103 | 2122 | GCAUCAGAGGAGUCAGGCAG | AUGCCUAAUUUCUCUGAUGC | Cleavage |
| ccp-miR160a-1-3p | Cc07_g08040 | 2.5 | 17.27 | 1 | 20 | 1391 | 1410 | GCAUCAGAGGAGUCAGGCAG | CUGCGUGACUUAUCUGAUGC | Translation |
| ccp-miR160a-1-3p | Cc07_g13300 | 3.0 | 19.396 | 1 | 20 | 1297 | 1316 | GCAUCAGAGGAGUCAGGCAG | AUGUUUGAUUUCUCUGAUGC | Cleavage |
| ccp-miR160a-1-5p | Cc01_g11020 | 1.0 | 21.799 | 1 | 20 | 1309 | 1328 | UGCCUGGCUCCCUGGAUGCC | GGCAUACAGGGAGCCAGGCA | Cleavage |
| ccp-miR160a-1-5p | Cc02_g11300 | 1.0 | 22.211 | 1 | 20 | 1886 | 1905 | UGCCUGGCUCCCUGGAUGCC | GGCAUACAGGGAGCCAGGCA | Cleavage |
| ccp-miR160a-2-5p | Cc01_g11020 | 1.0 | 21.799 | 1 | 20 | 1309 | 1328 | UGCCUGGCUCCCUGGAUGCC | GGCAUACAGGGAGCCAGGCA | Cleavage |
| ccp-miR160a-2-5p | Cc02_g11300 | 1.0 | 22.211 | 1 | 20 | 1886 | 1905 | UGCCUGGCUCCCUGGAUGCC | GGCAUACAGGGAGCCAGGCA | Cleavage |
| ccp-miR160h-5p | Cc01_g11020 | 1.0 | 21.799 | 1 | 20 | 1309 | 1328 | UGCCUGGCUCCCUGCAUGCC | GGCAUACAGGGAGCCAGGCA | Cleavage |
| ccp-miR160h-5p | Cc02_g11300 | 1.0 | 22.211 | 1 | 20 | 1886 | 1905 | UGCCUGGCUCCCUGCAUGCC | GGCAUACAGGGAGCCAGGCA | Cleavage |
| ccp-miR162a-3p | Cc11_g07660 | 2.5 | 21.029 | 1 | 21 | 1835 | 1855 | UCGAUAAACCUCUGCAUCCAG | UUGGAAGCACAGGUUUGUCGA | Cleavage |
| ccp-miR162a-5p | Cc02_g04620 | 3.0 | 14.807 | 1 | 21 | 2344 | 2364 | UGGAGGCAGCGGUUCAUCGAU | AUUGCUAAACUGCUGCCUCCA | Cleavage |
| ccp-miR162a-5p | Cc02_g18300 | 3.0 | 14.253 | 1 | 20 | 617 | 636 | UGGAGGCAGCGGUUCAUCGA | UCGAAGGAGCGUUGCCUCCA | Cleavage |
| ccp-miR162a-5p | Cc02_g29020 | 3.0 | 17.622 | 1 | 22 | 1600 | 1621 | UGGAGGCAGCGGUUCAUCGAUC | GCUAGAUGAAAUGCUGCUUCCA | Cleavage |
| ccp-miR164-1-3p | Cc06_g13290 | 3.0 | 19.775 | 1 | 20 | 1426 | 1445 | CAUGUGCCCUGCUUCUCCAC | GUGAGGAUGCAGGGCGCAUG | Cleavage |
| ccp-miR164-1-5p | Cc06_g11150 | 2.5 | 15.647 | 1 | 20 | 1148 | 1167 | UGGCAUGUGCCCUGCUUCUC | GGGAUGCAGGGUACGUGCCA | Cleavage |
| ccp-miR164-1-5p | Cc07_g03550 | 1.0 | 21.686 | 1 | 20 | 297 | 316 | UGGCAUGUGCCCUGCUUCUC | GAGGAGCAGGGCGCAUGCCA | Cleavage |
| ccp-miR164-2-3p | Cc00_g10800 | 3.0 | 21.85 | 1 | 20 | 2042 | 2061 | CAUGUGCCCUGAUCCUCCAU | AUGGGGGACCACGGCAUAUG | Translation |
| ccp-miR164-2-3p | Cc05_g10130 | 2.5 | 21.093 | 1 | 21 | 920 | 940 | CAUGUGCCCUGAUCCUCCAUC | GAUGGACGAUCAGGGGACAUG | Cleavage |
| ccp-miR164-2-5p | Cc00_g17220 | 3.0 | 19.709 | 1 | 20 | 1189 | 1207 | UGGAGAAGCAGGGCACAUGC | GCAUG-GUCCUGCUUUUCCA | Cleavage |
| ccp-miR164-2-5p | Cc00_g29790 | 3.0 | 15.764 | 1 | 20 | 72 | 91 | UGGAGAAGCAGGGCACAUGC | ACAUGUGCCAUGUUUCUUCA | Translation |
| ccp-miR164-2-5p | Cc02_g16120 | 3.0 | 15.764 | 1 | 20 | 72 | 91 | UGGAGAAGCAGGGCACAUGC | ACAUGUGCCAUGUUUCUUCA | Translation |
| ccp-miR164-2-5p | Cc03_g06590 | 1.5 | 17.366 | 1 | 20 | 1102 | 1121 | UGGAGAAGCAGGGCACAUGC | GCAUGUGCCUUGCUUCUCCU | Cleavage |
| ccp-miR164-2-5p | Cc03_g06600 | 1.5 | 14.869 | 1 | 20 | 1201 | 1220 | UGGAGAAGCAGGGCACAUGC | GCAUGUGCCUUGCUUCUCCU | Cleavage |
| ccp-miR164-2-5p | Cc03_g06610 | 1.5 | 20.557 | 1 | 20 | 1039 | 1058 | UGGAGAAGCAGGGCACAUGC | GCAUGUGCCUUGCUUCUCCU | Cleavage |
| ccp-miR164-2-5p | Cc03_g06640 | 1.5 | 22.519 | 1 | 20 | 1099 | 1118 | UGGAGAAGCAGGGCACAUGC | GCAUGUGCCUUGCUUCUCCU | Cleavage |
| ccp-miR164-2-5p | Cc03_g06650 | 1.5 | 14.893 | 1 | 20 | 1201 | 1220 | UGGAGAAGCAGGGCACAUGC | GCAUGUGCCUUGCUUCUCCU | Cleavage |
| ccp-miR164-2-5p | Cc03_g06660 | 1.5 | 22.896 | 1 | 20 | 1522 | 1541 | UGGAGAAGCAGGGCACAUGC | GCAUGUGCCUUGCUUCUCCU | Cleavage |
| ccp-miR164-2-5p | Cc03_g06680 | 1.5 | 20.957 | 1 | 20 | 1842 | 1861 | UGGAGAAGCAGGGCACAUGC | GCAUGUGCCUUGCUUCUCCU | Cleavage |
| ccp-miR164-2-5p | Cc03_g06700 | 1.5 | 20.945 | 1 | 20 | 1318 | 1337 | UGGAGAAGCAGGGCACAUGC | GCAUGUGCCUUGCUUCUCCU | Cleavage |
| ccp-miR164-2-5p | Cc04_g16290 | 3.0 | 20.221 | 1 | 21 | 650 | 670 | UGGAGAAGCAGGGCACAUGCC | GCCACGUGCACUGCUUCUCCA | Cleavage |
| ccp-miR164-2-5p | Cc05_g11720 | 0.5 | 12.886 | 1 | 20 | 702 | 721 | UGGAGAAGCAGGGCACAUGC | GCAUGUGCCCUGUUUCUCCA | Cleavage |
| ccp-miR164-2-5p | Cc05_g15960 | 1.0 | 11.099 | 1 | 20 | 609 | 628 | UGGAGAAGCAGGGCACAUGC | GCAAGUGCCCUGCUUCUCCA | Cleavage |
| ccp-miR164-2-5p | Cc07_g15660 | 3.0 | 24.226 | 1 | 20 | 2913 | 2932 | UGGAGAAGCAGGGCACAUGC | GCAAUUGCCCGGCUUCUCCA | Translation |
| ccp-miR164-2-5p | Cc10_g06760 | 3.0 | 16.738 | 1 | 21 | 1276 | 1296 | UGGAGAAGCAGGGCACAUGCC | GGCAUUUGAUUUGCUUCUCCA | Cleavage |
| ccp-mir164-3-3p | Cc00_g22920 | 2.5 | 13.674 | 1 | 21 | 976 | 996 | CACGUGCUCCCCUUCUCCAAC | GUUGGAGACUGGGAGCAUGUG | Cleavage |
| ccp-mir164-3-3p | Cc02_g26750 | 2.5 | 13.842 | 1 | 21 | 1523 | 1543 | CACGUGCUCCCCUUCUCCAAC | GUUGGAGACUGGGAGCAUGUG | Cleavage |
| ccp-mir164-3-3p | Cc09_g03730 | 3.0 | 14.71 | 1 | 20 | 696 | 715 | CACGUGCUCCCCUUCUCCAA | UUGGGGAGGGGGGGUGUGUG | Cleavage |
| ccp-mir164-3-5p | Cc01_g21260 | 3.0 | 21.575 | 1 | 20 | 1322 | 1341 | UGGAGAAGCAGGGCACGUGC | GCUCCUGCUUUGCUUCUCCA | Cleavage |
| ccp-mir164-3-5p | Cc02_g24740 | 3.0 | 18.126 | 1 | 20 | 2551 | 2570 | UGGAGAAGCAGGGCACGUGC | GCACGUGCUGUGCUUCUUCU | Translation |
| ccp-mir164-3-5p | Cc03_g06590 | 2.0 | 17.366 | 1 | 21 | 1101 | 1121 | UGGAGAAGCAGGGCACGUGCA | UGCAUGUGCCUUGCUUCUCCU | Cleavage |
| ccp-mir164-3-5p | Cc03_g06600 | 2.0 | 14.869 | 1 | 20 | 1201 | 1220 | UGGAGAAGCAGGGCACGUGC | GCAUGUGCCUUGCUUCUCCU | Cleavage |
| ccp-mir164-3-5p | Cc03_g06610 | 2.0 | 20.557 | 1 | 21 | 1038 | 1058 | UGGAGAAGCAGGGCACGUGCA | UGCAUGUGCCUUGCUUCUCCU | Cleavage |
| ccp-mir164-3-5p | Cc03_g06640 | 2.0 | 22.519 | 1 | 21 | 1098 | 1118 | UGGAGAAGCAGGGCACGUGCA | UGCAUGUGCCUUGCUUCUCCU | Cleavage |
| ccp-mir164-3-5p | Cc03_g06650 | 2.0 | 14.893 | 1 | 20 | 1201 | 1220 | UGGAGAAGCAGGGCACGUGC | GCAUGUGCCUUGCUUCUCCU | Cleavage |
| ccp-mir164-3-5p | Cc03_g06660 | 2.0 | 22.896 | 1 | 21 | 1521 | 1541 | UGGAGAAGCAGGGCACGUGCA | UGCAUGUGCCUUGCUUCUCCU | Cleavage |
| ccp-mir164-3-5p | Cc03_g06680 | 2.0 | 20.957 | 1 | 21 | 1841 | 1861 | UGGAGAAGCAGGGCACGUGCA | UGCAUGUGCCUUGCUUCUCCU | Cleavage |
| ccp-mir164-3-5p | Cc03_g06700 | 2.0 | 20.945 | 1 | 21 | 1317 | 1337 | UGGAGAAGCAGGGCACGUGCA | UGCAUGUGCCUUGCUUCUCCU | Cleavage |
| ccp-mir164-3-5p | Cc04_g16290 | 2.0 | 20.221 | 1 | 20 | 651 | 670 | UGGAGAAGCAGGGCACGUGC | CCACGUGCACUGCUUCUCCA | Cleavage |
| ccp-mir164-3-5p | Cc05_g11720 | 1.0 | 12.886 | 1 | 20 | 702 | 721 | UGGAGAAGCAGGGCACGUGC | GCAUGUGCCCUGUUUCUCCA | Cleavage |
| ccp-mir164-3-5p | Cc05_g15960 | 1.0 | 11.099 | 1 | 20 | 609 | 628 | UGGAGAAGCAGGGCACGUGC | GCAAGUGCCCUGCUUCUCCA | Cleavage |
| ccp-mir164-3-5p | Cc07_g01520 | 3.0 | 23.575 | 1 | 22 | 1229 | 1250 | UGGAGAAGCAGGGCACGUGCAA | UGGCACUUGACCAGCUUCUCCA | Translation |
| ccp-mir164-3-5p | Cc07_g15660 | 3.0 | 24.226 | 1 | 20 | 2913 | 2932 | UGGAGAAGCAGGGCACGUGC | GCAAUUGCCCGGCUUCUCCA | Translation |
| ccp-miR164a-5p | Cc01_g21260 | 3.0 | 21.575 | 1 | 20 | 1322 | 1341 | UGGAGAAGCAGGGCACGUGC | GCUCCUGCUUUGCUUCUCCA | Cleavage |
| ccp-miR164a-5p | Cc02_g24740 | 3.0 | 18.126 | 1 | 20 | 2551 | 2570 | UGGAGAAGCAGGGCACGUGC | GCACGUGCUGUGCUUCUUCU | Translation |
| ccp-miR164a-5p | Cc03_g06590 | 2.0 | 17.366 | 1 | 21 | 1101 | 1121 | UGGAGAAGCAGGGCACGUGCA | UGCAUGUGCCUUGCUUCUCCU | Cleavage |
| ccp-miR164a-5p | Cc03_g06600 | 2.0 | 14.869 | 1 | 20 | 1201 | 1220 | UGGAGAAGCAGGGCACGUGC | GCAUGUGCCUUGCUUCUCCU | Cleavage |
| ccp-miR164a-5p | Cc03_g06610 | 2.0 | 20.557 | 1 | 21 | 1038 | 1058 | UGGAGAAGCAGGGCACGUGCA | UGCAUGUGCCUUGCUUCUCCU | Cleavage |
| ccp-miR164a-5p | Cc03_g06640 | 2.0 | 22.519 | 1 | 21 | 1098 | 1118 | UGGAGAAGCAGGGCACGUGCA | UGCAUGUGCCUUGCUUCUCCU | Cleavage |
| ccp-miR164a-5p | Cc03_g06650 | 2.0 | 14.893 | 1 | 20 | 1201 | 1220 | UGGAGAAGCAGGGCACGUGC | GCAUGUGCCUUGCUUCUCCU | Cleavage |
| ccp-miR164a-5p | Cc03_g06660 | 2.0 | 22.896 | 1 | 21 | 1521 | 1541 | UGGAGAAGCAGGGCACGUGCA | UGCAUGUGCCUUGCUUCUCCU | Cleavage |
| ccp-miR164a-5p | Cc03_g06680 | 2.0 | 20.957 | 1 | 21 | 1841 | 1861 | UGGAGAAGCAGGGCACGUGCA | UGCAUGUGCCUUGCUUCUCCU | Cleavage |
| ccp-miR164a-5p | Cc03_g06700 | 2.0 | 20.945 | 1 | 21 | 1317 | 1337 | UGGAGAAGCAGGGCACGUGCA | UGCAUGUGCCUUGCUUCUCCU | Cleavage |
| ccp-miR164a-5p | Cc04_g16290 | 2.0 | 20.221 | 1 | 20 | 651 | 670 | UGGAGAAGCAGGGCACGUGC | CCACGUGCACUGCUUCUCCA | Cleavage |
| ccp-miR164a-5p | Cc05_g11720 | 1.0 | 12.886 | 1 | 20 | 702 | 721 | UGGAGAAGCAGGGCACGUGC | GCAUGUGCCCUGUUUCUCCA | Cleavage |
| ccp-miR164a-5p | Cc05_g15960 | 1.0 | 11.099 | 1 | 20 | 609 | 628 | UGGAGAAGCAGGGCACGUGC | GCAAGUGCCCUGCUUCUCCA | Cleavage |
| ccp-miR164a-5p | Cc07_g01520 | 3.0 | 23.575 | 1 | 20 | 1231 | 1250 | UGGAGAAGCAGGGCACGUGC | GCACUUGACCAGCUUCUCCA | Translation |
| ccp-miR164a-5p | Cc07_g15660 | 3.0 | 24.226 | 1 | 20 | 2913 | 2932 | UGGAGAAGCAGGGCACGUGC | GCAAUUGCCCGGCUUCUCCA | Translation |
| ccp-miR164c-5p | Cc02_g17260 | 3.0 | 16.954 | 1 | 20 | 70 | 89 | UGGAGAAGGGGAGCACGUGC | GAAAGUGCUCACCUUCUCCA | Translation |
| ccp-miR164c-5p | Cc02_g37530 | 2.5 | 13.628 | 1 | 20 | 451 | 470 | UGGAGAAGGGGAGCACGUGC | GCCUGUGUUCCUCUUCUCCA | Cleavage |
| ccp-miR166-1-3p | Cc01_g08870 | 3.0 | 22.544 | 1 | 22 | 1362 | 1383 | UCGGACCAGGCUUCAUUCCCCC | GAGGGAAAGAGGCCUGGUUCGC | Cleavage |
| ccp-miR166-1-3p | Cc01_g11100 | 2.0 | 19.111 | 1 | 20 | 1559 | 1578 | UCGGACCAGGCUUCAUUCCC | UGGGAUGAAGCCUGGUCCGG | Cleavage |
| ccp-miR166-1-3p | Cc08_g09740 | 2.0 | 24.014 | 1 | 20 | 623 | 642 | UCGGACCAGGCUUCAUUCCC | UGGGAUGAAGCCUGGUCCGG | Cleavage |
| ccp-miR166-1-3p | Cc09_g08040 | 2.0 | 23.827 | 1 | 20 | 579 | 598 | UCGGACCAGGCUUCAUUCCC | UGGGAUGAAGCCUGGUCCGG | Cleavage |
| ccp-miR166-2-3p | Cc01_g08870 | 3.0 | 22.544 | 1 | 22 | 1362 | 1383 | UCGGACCAGGCUUCAUUCCCCC | GAGGGAAAGAGGCCUGGUUCGC | Cleavage |
| ccp-miR166-2-3p | Cc01_g11100 | 2.0 | 19.111 | 1 | 20 | 1559 | 1578 | UCGGACCAGGCUUCAUUCCC | UGGGAUGAAGCCUGGUCCGG | Cleavage |
| ccp-miR166-2-3p | Cc08_g09740 | 2.0 | 24.014 | 1 | 20 | 623 | 642 | UCGGACCAGGCUUCAUUCCC | UGGGAUGAAGCCUGGUCCGG | Cleavage |
| ccp-miR166-2-3p | Cc09_g08040 | 2.0 | 23.827 | 1 | 20 | 579 | 598 | UCGGACCAGGCUUCAUUCCC | UGGGAUGAAGCCUGGUCCGG | Cleavage |
| ccp-miR166-3-5p | Cc00_g10000 | 3.0 | 22.36 | 1 | 21 | 291 | 311 | GGAAUGUUGUCUGGUGCGAGG | UUUUGCAUCAGAUAACGUUCU | Cleavage |
| ccp-miR166-3-5p | Cc01_g12100 | 3.0 | 14.785 | 1 | 21 | 531 | 551 | GGAAUGUUGUCUGGUGCGAGG | UUUCACAUCAAACAACAUUCC | Translation |
| ccp-miR166-3-5p | Cc01_g13440 | 3.0 | 11.186 | 1 | 21 | 253 | 273 | GGAAUGUUGUCUGGUGCGAGG | UCUUGCGCCAACCAACAUUCC | Translation |
| ccp-miR166-3-5p | Cc06_g15920 | 3.0 | 22.401 | 1 | 21 | 873 | 893 | GGAAUGUUGUCUGGUGCGAGG | CUUUGCACUAGAUGAUAUUCC | Cleavage |
| ccp-miR166-3-5p | Cc08_g11430 | 3.0 | 19.628 | 1 | 21 | 3235 | 3255 | GGAAUGUUGUCUGGUGCGAGG | UUUUGCGGCGGACAACAUUCC | Cleavage |
| ccp-miR166-4-3p | Cc00_g09120 | 3.0 | 19.246 | 1 | 20 | 5200 | 5218 | UCGGACCAGGCUUCAUUCCU | AGGAAGGAAGC-UGGUCCGA | Translation |
| ccp-miR166-4-3p | Cc01_g11100 | 2.0 | 19.111 | 1 | 20 | 1559 | 1578 | UCGGACCAGGCUUCAUUCCU | UGGGAUGAAGCCUGGUCCGG | Cleavage |
| ccp-miR166-4-3p | Cc08_g09740 | 2.0 | 24.014 | 1 | 20 | 623 | 642 | UCGGACCAGGCUUCAUUCCU | UGGGAUGAAGCCUGGUCCGG | Cleavage |
| ccp-miR166-4-3p | Cc09_g08040 | 2.0 | 23.827 | 1 | 20 | 579 | 598 | UCGGACCAGGCUUCAUUCCU | UGGGAUGAAGCCUGGUCCGG | Cleavage |
| ccp-miR166-4-5p | Cc02_g00470 | 3.0 | 18.034 | 1 | 20 | 1263 | 1282 | AAUGAAGUUUGAUCCAAGAU | AUCUUGGAACAAUCUUUGUU | Cleavage |
| ccp-miR166-4-5p | Cc03_g04310 | 3.0 | 17.383 | 1 | 20 | 700 | 719 | AAUGAAGUUUGAUCCAAGAU | AUGUUGGAUUGGAUUUCAUU | Cleavage |
| ccp-miR166-4-5p | Cc03_g13490 | 2.5 | 19.03 | 1 | 20 | 1106 | 1124 | AAUGAAGUUUGAUCCAAGAU | GUCUUGGAUCAA-CUUCAUU | Cleavage |
| ccp-miR166-4-5p | Cc03_g15710 | 3.0 | 18.034 | 1 | 20 | 1018 | 1037 | AAUGAAGUUUGAUCCAAGAU | AUCUUGGAACAAUCUUUGUU | Cleavage |
| ccp-miR166-4-5p | Cc05_g00690 | 3.0 | 18.034 | 1 | 20 | 1018 | 1037 | AAUGAAGUUUGAUCCAAGAU | AUCUUGGAACAAUCUUUGUU | Cleavage |
| ccp-miR166-4-5p | Cc05_g07220 | 3.0 | 20.129 | 1 | 21 | 1424 | 1444 | AAUGAAGUUUGAUCCAAGAUC | GAUCUGGGAUCUGAUUUCAUU | Translation |
| ccp-miR166-4-5p | Cc07_g09530 | 3.0 | 18.034 | 1 | 20 | 840 | 859 | AAUGAAGUUUGAUCCAAGAU | AUCUUGGAACAAUCUUUGUU | Cleavage |
| ccp-miR166-4-5p | Cc09_g07670 | 3.0 | 22.165 | 1 | 20 | 1093 | 1113 | AAUGAAGUUUGAU-CCAAGAU | AUCUUGGUAUCAAUCUUCAUU | Cleavage |
| ccp-miR166a-1-3p | Cc01_g08870 | 3.0 | 22.544 | 1 | 20 | 1364 | 1383 | UCGGACCAGGCUUCAUUCCC | GGGAAAGAGGCCUGGUUCGC | Cleavage |
| ccp-miR166a-1-3p | Cc01_g11100 | 2.0 | 19.111 | 1 | 20 | 1559 | 1578 | UCGGACCAGGCUUCAUUCCC | UGGGAUGAAGCCUGGUCCGG | Cleavage |
| ccp-miR166a-1-3p | Cc08_g09740 | 2.0 | 24.014 | 1 | 20 | 623 | 642 | UCGGACCAGGCUUCAUUCCC | UGGGAUGAAGCCUGGUCCGG | Cleavage |
| ccp-miR166a-1-3p | Cc09_g08040 | 2.0 | 23.827 | 1 | 20 | 579 | 598 | UCGGACCAGGCUUCAUUCCC | UGGGAUGAAGCCUGGUCCGG | Cleavage |
| ccp-miR166a-2-3p | Cc01_g08870 | 3.0 | 22.544 | 1 | 20 | 1364 | 1383 | UCGGACCAGGCUUCAUUCCC | GGGAAAGAGGCCUGGUUCGC | Cleavage |
| ccp-miR166a-2-3p | Cc01_g11100 | 2.0 | 19.111 | 1 | 20 | 1559 | 1578 | UCGGACCAGGCUUCAUUCCC | UGGGAUGAAGCCUGGUCCGG | Cleavage |
| ccp-miR166a-2-3p | Cc08_g09740 | 2.0 | 24.014 | 1 | 20 | 623 | 642 | UCGGACCAGGCUUCAUUCCC | UGGGAUGAAGCCUGGUCCGG | Cleavage |
| ccp-miR166a-2-3p | Cc09_g08040 | 2.0 | 23.827 | 1 | 20 | 579 | 598 | UCGGACCAGGCUUCAUUCCC | UGGGAUGAAGCCUGGUCCGG | Cleavage |
| ccp-miR166a-3-3p | Cc04_g05060 | 3.0 | 21.56 | 1 | 20 | 231 | 249 | UCGAACCAGACGACAUUCCC | GGUAAUG-CGUCUGGUUCGA | Cleavage |
| ccp-miR167-1-3p | Cc06_g03180 | 3.0 | 19.722 | 1 | 20 | 858 | 877 | GGUCAUGCUCUGACAGCCUC | GAUGUUGUUGGAGCAUGAUC | Cleavage |
| ccp-miR167-1-5p | Cc00_g03550 | 3.0 | 17.486 | 1 | 20 | 408 | 427 | UGAAGCUGCCAGCAUGAUCU | UGGUUAUGCUAGCAGCUUCA | Translation |
| ccp-miR167-1-5p | Cc00_g03560 | 3.0 | 17.324 | 1 | 20 | 495 | 514 | UGAAGCUGCCAGCAUGAUCU | UGGUUAUGCUAGCAGCUUCA | Translation |
| ccp-miR167-1-5p | Cc01_g16410 | 3.0 | 17.681 | 1 | 20 | 2327 | 2346 | UGAAGCUGCCAGCAUGAUCU | AGGUUAUGCUGGCUGUUUCA | Cleavage |
| ccp-miR167-1-5p | Cc03_g11570 | 3.0 | 17.486 | 1 | 20 | 294 | 313 | UGAAGCUGCCAGCAUGAUCU | UGGUUAUGCUAGCAGCUUCA | Translation |
| ccp-miR167-1-5p | Cc03_g11920 | 3.0 | 14.872 | 1 | 20 | 327 | 346 | UGAAGCUGCCAGCAUGAUCU | UGGUUAUGCUAGCAGCUUCA | Translation |
| ccp-miR167-1-5p | Cc03_g12140 | 3.0 | 19.871 | 1 | 20 | 399 | 418 | UGAAGCUGCCAGCAUGAUCU | UGGUUAUGCUAGCAGCUUCA | Translation |
| ccp-miR167-1-5p | Cc08_g10510 | 2.5 | 20.906 | 1 | 20 | 677 | 696 | UGAAGCUGCCAGCAUGAUCU | AAAUGAUGCUGGUAGCUUCA | Cleavage |
| ccp-miR167-1-5p | Cc08_g14790 | 3.0 | 17.905 | 1 | 20 | 1970 | 1989 | UGAAGCUGCCAGCAUGAUCU | AGUUCAUGAUGGUAGCUUUA | Cleavage |
| ccp-miR167-1-5p | Cc09_g03270 | 3.0 | 19.159 | 1 | 20 | 1920 | 1939 | UGAAGCUGCCAGCAUGAUCU | AGAUUAUGCUGGUGGCUACA | Cleavage |
| ccp-miR167-1-5p | Cc10_g06820 | 3.0 | 19.988 | 1 | 20 | 630 | 649 | UGAAGCUGCCAGCAUGAUCU | AGAUUGUGCUGAGAGCUUCA | Translation |
| ccp-miR167-2-3p | Cc01_g20800 | 2.0 | 18.281 | 1 | 20 | 331 | 350 | AUCAUGCUGGCAGCUUCAAC | GUUGCAGUUGCCAGCAUGGU | Cleavage |
| ccp-miR167-2-3p | Cc02_g13490 | 3.0 | 12.343 | 1 | 21 | 586 | 607 | AUCAUGC-UGGCAGCUUCAACU | AGUUGAAGUUGCUAUGCAUGAU | Cleavage |
| ccp-miR167-2-3p | Cc05_g00360 | 3.0 | 21.28 | 1 | 20 | 244 | 263 | AUCAUGCUGGCAGCUUCAAC | GCUGAAGCUGUCAGGAUGAU | Cleavage |
| ccp-miR167-2-3p | Cc08_g05990 | 3.0 | 13.645 | 1 | 24 | 892 | 915 | AUCAUGCUGGCAGCUUCAACUGAU | AUUAGGUGCAGCUGCCAGCAUGAG | Cleavage |
| ccp-miR167-2-3p | Cc10_g01630 | 3.0 | 21.716 | 1 | 21 | 146 | 166 | AUCAUGCUGGCAGCUUCAACU | AGUCGAGGCUGCCUGCAUGGU | Cleavage |
| ccp-miR167-2-3p | Cc10_g12790 | 3.0 | 17.516 | 1 | 20 | 1606 | 1625 | AUCAUGCUGGCAGCUUCAAC | GAUGAAGCUGACCGCAUGAU | Translation |
| ccp-miR167-2-5p | Cc02_g20540 | 3.0 | 14.81 | 1 | 22 | 1776 | 1797 | UGAAGCUACCACAUGAUCUGAU | ACCUGUUCUUGUGGUAGCUUCA | Cleavage |
| ccp-miR167-2-5p | Cc11_g08910 | 3.0 | 19.227 | 1 | 20 | 473 | 492 | UGAAGCUACCACAUGAUCUG | CAGGUCAGGUGAUGGCUUCA | Translation |
| ccp-miR167-3-3p | Cc00_g03550 | 3.0 | 17.486 | 1 | 20 | 408 | 427 | UGAAGCUGCCAGCAUGAUCU | UGGUUAUGCUAGCAGCUUCA | Translation |
| ccp-miR167-3-3p | Cc00_g03560 | 3.0 | 17.324 | 1 | 20 | 495 | 514 | UGAAGCUGCCAGCAUGAUCU | UGGUUAUGCUAGCAGCUUCA | Translation |
| ccp-miR167-3-3p | Cc01_g16410 | 3.0 | 17.681 | 1 | 20 | 2327 | 2346 | UGAAGCUGCCAGCAUGAUCU | AGGUUAUGCUGGCUGUUUCA | Cleavage |
| ccp-miR167-3-3p | Cc03_g11570 | 3.0 | 17.486 | 1 | 20 | 294 | 313 | UGAAGCUGCCAGCAUGAUCU | UGGUUAUGCUAGCAGCUUCA | Translation |
| ccp-miR167-3-3p | Cc03_g11920 | 3.0 | 14.872 | 1 | 20 | 327 | 346 | UGAAGCUGCCAGCAUGAUCU | UGGUUAUGCUAGCAGCUUCA | Translation |
| ccp-miR167-3-3p | Cc03_g12140 | 3.0 | 19.871 | 1 | 20 | 399 | 418 | UGAAGCUGCCAGCAUGAUCU | UGGUUAUGCUAGCAGCUUCA | Translation |
| ccp-miR167-3-3p | Cc08_g10510 | 2.5 | 20.906 | 1 | 20 | 677 | 696 | UGAAGCUGCCAGCAUGAUCU | AAAUGAUGCUGGUAGCUUCA | Cleavage |
| ccp-miR167-3-3p | Cc08_g14790 | 3.0 | 17.905 | 1 | 22 | 1968 | 1989 | UGAAGCUGCCAGCAUGAUCUAA | UCAGUUCAUGAUGGUAGCUUUA | Cleavage |
| ccp-miR167-3-3p | Cc09_g03270 | 3.0 | 19.159 | 1 | 20 | 1920 | 1939 | UGAAGCUGCCAGCAUGAUCU | AGAUUAUGCUGGUGGCUACA | Cleavage |
| ccp-miR167-3-3p | Cc10_g06820 | 3.0 | 19.988 | 1 | 20 | 630 | 649 | UGAAGCUGCCAGCAUGAUCU | AGAUUGUGCUGAGAGCUUCA | Translation |
| ccp-miR167-3-5p | Cc01_g20860 | 3.0 | 16.195 | 1 | 20 | 435 | 454 | GAUCAUGUGGUAGCUUCACC | GCUGAAGAUGCCACAUGAUU | Cleavage |
| ccp-miR167-3-5p | Cc07_g10380 | 3.0 | 22.054 | 1 | 20 | 1435 | 1454 | GAUCAUGUGGUAGCUUCACC | GGUUAAGUUAUUAUAUGAUC | Cleavage |
| ccp-miR167-4-5p | Cc00_g03550 | 3.0 | 17.486 | 1 | 20 | 408 | 427 | UGAAGCUGCCAGCAUGAUCU | UGGUUAUGCUAGCAGCUUCA | Translation |
| ccp-miR167-4-5p | Cc00_g03560 | 3.0 | 17.324 | 1 | 20 | 495 | 514 | UGAAGCUGCCAGCAUGAUCU | UGGUUAUGCUAGCAGCUUCA | Translation |
| ccp-miR167-4-5p | Cc01_g16410 | 3.0 | 17.681 | 1 | 22 | 2325 | 2346 | UGAAGCUGCCAGCAUGAUCUGG | CGAGGUUAUGCUGGCUGUUUCA | Cleavage |
| ccp-miR167-4-5p | Cc03_g11570 | 3.0 | 17.486 | 1 | 20 | 294 | 313 | UGAAGCUGCCAGCAUGAUCU | UGGUUAUGCUAGCAGCUUCA | Translation |
| ccp-miR167-4-5p | Cc03_g11920 | 3.0 | 14.872 | 1 | 20 | 327 | 346 | UGAAGCUGCCAGCAUGAUCU | UGGUUAUGCUAGCAGCUUCA | Translation |
| ccp-miR167-4-5p | Cc03_g12140 | 3.0 | 19.871 | 1 | 20 | 399 | 418 | UGAAGCUGCCAGCAUGAUCU | UGGUUAUGCUAGCAGCUUCA | Translation |
| ccp-miR167-4-5p | Cc08_g10510 | 2.5 | 20.906 | 1 | 21 | 676 | 696 | UGAAGCUGCCAGCAUGAUCUG | CAAAUGAUGCUGGUAGCUUCA | Cleavage |
| ccp-miR167-4-5p | Cc08_g14790 | 3.0 | 17.905 | 1 | 22 | 1968 | 1989 | UGAAGCUGCCAGCAUGAUCUGG | UCAGUUCAUGAUGGUAGCUUUA | Cleavage |
| ccp-miR167-4-5p | Cc09_g03270 | 3.0 | 19.159 | 1 | 20 | 1920 | 1939 | UGAAGCUGCCAGCAUGAUCU | AGAUUAUGCUGGUGGCUACA | Cleavage |
| ccp-miR167-4-5p | Cc10_g06820 | 3.0 | 19.988 | 1 | 20 | 630 | 649 | UGAAGCUGCCAGCAUGAUCU | AGAUUGUGCUGAGAGCUUCA | Translation |
| ccp-miR167a-1-3p | Cc08_g14830 | 3.0 | 19.833 | 1 | 20 | 823 | 842 | GAUCUUGCGGUAGCCUCACG | CGUGAUGCUUCCGCAAGAUG | Translation |
| ccp-miR167a-2-3p | Cc11_g12320 | 3.0 | 20.883 | 1 | 20 | 1007 | 1026 | AGAUCAUGCGGUAGUUUCAC | GAGAAAUUACCUCAUGAUUU | Translation |
| ccp-miR167a-2-5p | Cc00_g03550 | 3.0 | 17.486 | 1 | 20 | 408 | 427 | UGAAGCUGCCAGCAUGAUCU | UGGUUAUGCUAGCAGCUUCA | Translation |
| ccp-miR167a-2-5p | Cc00_g03560 | 3.0 | 17.324 | 1 | 20 | 495 | 514 | UGAAGCUGCCAGCAUGAUCU | UGGUUAUGCUAGCAGCUUCA | Translation |
| ccp-miR167a-2-5p | Cc01_g16410 | 3.0 | 17.681 | 1 | 20 | 2327 | 2346 | UGAAGCUGCCAGCAUGAUCU | AGGUUAUGCUGGCUGUUUCA | Cleavage |
| ccp-miR167a-2-5p | Cc03_g11570 | 3.0 | 17.486 | 1 | 20 | 294 | 313 | UGAAGCUGCCAGCAUGAUCU | UGGUUAUGCUAGCAGCUUCA | Translation |
| ccp-miR167a-2-5p | Cc03_g11920 | 3.0 | 14.872 | 1 | 20 | 327 | 346 | UGAAGCUGCCAGCAUGAUCU | UGGUUAUGCUAGCAGCUUCA | Translation |
| ccp-miR167a-2-5p | Cc03_g12140 | 3.0 | 19.871 | 1 | 20 | 399 | 418 | UGAAGCUGCCAGCAUGAUCU | UGGUUAUGCUAGCAGCUUCA | Translation |
| ccp-miR167a-2-5p | Cc08_g10510 | 2.5 | 20.906 | 1 | 21 | 676 | 696 | UGAAGCUGCCAGCAUGAUCUG | CAAAUGAUGCUGGUAGCUUCA | Cleavage |
| ccp-miR167a-2-5p | Cc08_g14790 | 3.0 | 17.905 | 1 | 22 | 1968 | 1989 | UGAAGCUGCCAGCAUGAUCUGA | UCAGUUCAUGAUGGUAGCUUUA | Cleavage |
| ccp-miR167a-2-5p | Cc09_g03270 | 3.0 | 19.159 | 1 | 20 | 1920 | 1939 | UGAAGCUGCCAGCAUGAUCU | AGAUUAUGCUGGUGGCUACA | Cleavage |
| ccp-miR167a-2-5p | Cc10_g06820 | 3.0 | 19.988 | 1 | 20 | 630 | 649 | UGAAGCUGCCAGCAUGAUCU | AGAUUGUGCUGAGAGCUUCA | Translation |
| ccp-miR167h-1-5p | Cc08_g01310 | 3.0 | 16.739 | 1 | 20 | 2026 | 2045 | UGAAGCUGCCAACACGAUCU | AGCUUGUGUUGGCAGCUUUU | Cleavage |
| ccp-miR167h-2-3p | Cc01_g20800 | 2.0 | 18.281 | 1 | 24 | 327 | 350 | AUCAUGCUGGCAGCUUCAACUACG | CGUUGUUGCAGUUGCCAGCAUGGU | Cleavage |
| ccp-miR167h-2-3p | Cc02_g13490 | 3.0 | 12.343 | 1 | 21 | 586 | 607 | AUCAUGC-UGGCAGCUUCAACU | AGUUGAAGUUGCUAUGCAUGAU | Cleavage |
| ccp-miR167h-2-3p | Cc05_g00360 | 3.0 | 21.28 | 1 | 20 | 244 | 263 | AUCAUGCUGGCAGCUUCAAC | GCUGAAGCUGUCAGGAUGAU | Cleavage |
| ccp-miR167h-2-3p | Cc08_g05990 | 3.0 | 13.645 | 1 | 22 | 894 | 915 | AUCAUGCUGGCAGCUUCAACUA | UAGGUGCAGCUGCCAGCAUGAG | Cleavage |
| ccp-miR167h-2-3p | Cc10_g01630 | 3.0 | 21.716 | 1 | 21 | 146 | 166 | AUCAUGCUGGCAGCUUCAACU | AGUCGAGGCUGCCUGCAUGGU | Cleavage |
| ccp-miR167h-2-3p | Cc10_g12790 | 3.0 | 17.516 | 1 | 22 | 1604 | 1625 | AUCAUGCUGGCAGCUUCAACUA | UUGAUGAAGCUGACCGCAUGAU | Translation |
| ccp-miR168a-3p | Cc08_g06450 | 3.0 | 18.101 | 1 | 21 | 640 | 659 | CCCGCCUUGCAUCAACUGAAU | AUUCAGUUGAUG-AGGGUGGG | Translation |
| ccp-miR168a-5p | Cc04_g00050 | 3.0 | 21.483 | 1 | 20 | 1835 | 1854 | UCGCUUGGUGCAGGUCGGGA | UUUCAGCCUGCACCAAGCGG | Cleavage |
| ccp-miR168a-5p | Cc04_g08880 | 3.0 | 23.239 | 1 | 21 | 548 | 568 | UCGCUUGGUGCAGGUCGGGAA | UUCCCGAGCUGCAUCAAGCAA | Cleavage |
| ccp-miR169-1-3p | Cc10_g06710 | 3.0 | 17.213 | 1 | 21 | 902 | 922 | GGCAAGUUGUUAUUGGCUACA | UUUGGCAAAUAAUAACUUGCC | Cleavage |
| ccp-miR169-1-5p | Cc00_g18240 | 2.5 | 16.673 | 1 | 21 | 3143 | 3163 | CAGCCAAGGAUGACUUGCCGG | UUGGCAACACAUCCUUGGCUG | Cleavage |
| ccp-miR169-1-5p | Cc00_g19380 | 2.5 | 13.826 | 1 | 21 | 1303 | 1323 | CAGCCAAGGAUGACUUGCCGG | UUGGCAAUUCAUCCUUGGCUU | Cleavage |
| ccp-miR169-1-5p | Cc01_g14140 | 3.0 | 21.037 | 1 | 20 | 1259 | 1278 | CAGCCAAGGAUGACUUGCCG | CUGCUAGUCAUUUUUGGCUG | Cleavage |
| ccp-miR169-1-5p | Cc01_g20910 | 3.0 | 23.611 | 1 | 20 | 1204 | 1223 | CAGCCAAGGAUGACUUGCCG | CAGCAAGUCAUCUUUGGCAG | Cleavage |
| ccp-miR169-1-5p | Cc03_g02470 | 2.5 | 20.884 | 1 | 21 | 1501 | 1521 | CAGCCAAGGAUGACUUGCCGG | UCAGCGAGUCACCCUUGGCUG | Translation |
| ccp-miR169-1-5p | Cc04_g01480 | 1.5 | 17.033 | 1 | 20 | 1532 | 1551 | CAGCCAAGGAUGACUUGCCG | UGGGAAGUCAUCCUUGGCUG | Cleavage |
| ccp-miR169-1-5p | Cc08_g01380 | 3.0 | 22.141 | 1 | 21 | 2263 | 2283 | CAGCCAAGGAUGACUUGCCGG | UUGGCAGGACAUCCUUGGCUA | Cleavage |
| ccp-miR169-2-3p | Cc02_g25530 | 2.5 | 17.108 | 1 | 20 | 2391 | 2410 | GGCAAGUUGUCUUUGGCUAC | GUACUCAAAGACAACUUGCA | Cleavage |
| ccp-miR169-2-3p | Cc04_g13000 | 3.0 | 21.334 | 1 | 19 | 5098 | 5117 | GGCAAGUUGUCUUUG-GCUA | UGGCACAAAGACAACUUGCU | Cleavage |
| ccp-miR169-2-3p | Cc05_g02150 | 2.0 | 14.751 | 1 | 20 | 277 | 296 | GGCAAGUUGUCUUUGGCUAC | GUAGCCAAAGACAAAUUGUC | Cleavage |
| ccp-miR169-2-5p | Cc00_g18240 | 2.5 | 16.673 | 1 | 21 | 3143 | 3163 | CAGCCAAGGAUGACUUGCCGA | UUGGCAACACAUCCUUGGCUG | Cleavage |
| ccp-miR169-2-5p | Cc00_g19380 | 2.5 | 13.826 | 1 | 21 | 1303 | 1323 | CAGCCAAGGAUGACUUGCCGA | UUGGCAAUUCAUCCUUGGCUU | Cleavage |
| ccp-miR169-2-5p | Cc01_g14140 | 3.0 | 21.037 | 1 | 20 | 1259 | 1278 | CAGCCAAGGAUGACUUGCCG | CUGCUAGUCAUUUUUGGCUG | Cleavage |
| ccp-miR169-2-5p | Cc01_g20910 | 3.0 | 23.611 | 1 | 20 | 1204 | 1223 | CAGCCAAGGAUGACUUGCCG | CAGCAAGUCAUCUUUGGCAG | Cleavage |
| ccp-miR169-2-5p | Cc03_g02470 | 2.5 | 20.884 | 1 | 21 | 1501 | 1521 | CAGCCAAGGAUGACUUGCCGA | UCAGCGAGUCACCCUUGGCUG | Translation |
| ccp-miR169-2-5p | Cc04_g01480 | 1.5 | 17.033 | 1 | 20 | 1532 | 1551 | CAGCCAAGGAUGACUUGCCG | UGGGAAGUCAUCCUUGGCUG | Cleavage |
| ccp-miR169-2-5p | Cc08_g01380 | 3.0 | 22.141 | 1 | 21 | 2263 | 2283 | CAGCCAAGGAUGACUUGCCGA | UUGGCAGGACAUCCUUGGCUA | Cleavage |
| ccp-miR169-3-3p | Cc04_g08530 | 3.0 | 18.977 | 1 | 21 | 1393 | 1413 | GGCAAGUUGUCCUUGGCUACG | UGUAGCCAAGGAGGAUUUGUU | Translation |
| ccp-miR169-3-3p | Cc05_g02150 | 3.0 | 14.751 | 1 | 20 | 277 | 296 | GGCAAGUUGUCCUUGGCUAC | GUAGCCAAAGACAAAUUGUC | Cleavage |
| ccp-miR169-3-5p | Cc00_g18240 | 2.5 | 16.673 | 1 | 21 | 3143 | 3163 | CAGCCAAGGAUGACUUGCCGG | UUGGCAACACAUCCUUGGCUG | Cleavage |
| ccp-miR169-3-5p | Cc00_g19380 | 2.5 | 13.826 | 1 | 21 | 1303 | 1323 | CAGCCAAGGAUGACUUGCCGG | UUGGCAAUUCAUCCUUGGCUU | Cleavage |
| ccp-miR169-3-5p | Cc01_g14140 | 3.0 | 21.037 | 1 | 20 | 1259 | 1278 | CAGCCAAGGAUGACUUGCCG | CUGCUAGUCAUUUUUGGCUG | Cleavage |
| ccp-miR169-3-5p | Cc01_g20910 | 3.0 | 23.611 | 1 | 20 | 1204 | 1223 | CAGCCAAGGAUGACUUGCCG | CAGCAAGUCAUCUUUGGCAG | Cleavage |
| ccp-miR169-3-5p | Cc03_g02470 | 2.5 | 20.884 | 1 | 21 | 1501 | 1521 | CAGCCAAGGAUGACUUGCCGG | UCAGCGAGUCACCCUUGGCUG | Translation |
| ccp-miR169-3-5p | Cc04_g01480 | 1.5 | 17.033 | 1 | 20 | 1532 | 1551 | CAGCCAAGGAUGACUUGCCG | UGGGAAGUCAUCCUUGGCUG | Cleavage |
| ccp-miR169-3-5p | Cc08_g01380 | 3.0 | 22.141 | 1 | 21 | 2263 | 2283 | CAGCCAAGGAUGACUUGCCGG | UUGGCAGGACAUCCUUGGCUA | Cleavage |
| ccp-miR169-4-3p | Cc00_g19380 | 3.0 | 13.826 | 1 | 20 | 1304 | 1323 | AAGCCAAGGAUCGGUUGCCU | UGGCAAUUCAUCCUUGGCUU | Cleavage |
| ccp-miR169-4-3p | Cc02_g28060 | 2.5 | 17.885 | 1 | 20 | 1235 | 1254 | AAGCCAAGGAUCGGUUGCCU | AGGCAAUUCAUUCUUGGCUU | Cleavage |
| ccp-miR169-4-3p | Cc05_g06880 | 3.0 | 18.599 | 1 | 21 | 804 | 824 | AAGCCAAGGAUCGGUUGCCUC | GGGUCAACCGAUUAUUGGCUU | Cleavage |
| ccp-miR169-4-3p | Cc06_g06710 | 2.0 | 22.167 | 1 | 20 | 1268 | 1287 | AAGCCAAGGAUCGGUUGCCU | AGGCAACUCAUUCUUGGCUU | Cleavage |
| ccp-miR169-4-3p | Cc06_g18980 | 3.0 | 22.056 | 1 | 20 | 166 | 185 | AAGCCAAGGAUCGGUUGCCU | AAGCAACUGCUCUUUGGCUU | Translation |
| ccp-miR169-4-3p | Cc07_g04880 | 3.0 | 15.524 | 1 | 20 | 2312 | 2331 | AAGCCAAGGAUCGGUUGCCU | AGGAAACUCGUCCUUGGCUU | Cleavage |
| ccp-miR169d-5p | Cc00_g12500 | 2.5 | 10.201 | 1 | 20 | 674 | 693 | AGCCAAGGAUGAAUUGCCGG | CCGGCCCUUUAUCCUUGGCU | Cleavage |
| ccp-miR169d-5p | Cc00_g18240 | 3.0 | 18.549 | 1 | 21 | 3142 | 3162 | AGCCAAGGAUGAAUUGCCGGC | GUUGGCAACACAUCCUUGGCU | Cleavage |
| ccp-miR169d-5p | Cc00_g19380 | 1.0 | 13.792 | 1 | 20 | 1303 | 1322 | AGCCAAGGAUGAAUUGCCGG | UUGGCAAUUCAUCCUUGGCU | Cleavage |
| ccp-miR169d-5p | Cc02_g28060 | 1.5 | 18.568 | 1 | 20 | 1234 | 1253 | AGCCAAGGAUGAAUUGCCGG | CAGGCAAUUCAUUCUUGGCU | Cleavage |
| ccp-miR169e-5p | Cc01_g11880 | 3.0 | 17.192 | 1 | 20 | 1237 | 1256 | UAGCCAAGGAUGGCUUGCCU | AGGCAGGGCAUCCUUGGUUU | Cleavage |
| ccp-miR169e-5p | Cc02_g05590 | 3.0 | 14.422 | 1 | 21 | 924 | 943 | UAGCCAAGGAUGGCUUGCCUC | GAGGCAAGUCA-CUUUGGCUA | Translation |
| ccp-miR169e-5p | Cc02_g05980 | 2.5 | 21.143 | 1 | 21 | 3279 | 3299 | UAGCCAAGGAUGGCUUGCCUC | GGGGCAAUCCAUCCUUGGUUG | Cleavage |
| ccp-miR169e-5p | Cc02_g28060 | 3.0 | 17.885 | 1 | 20 | 1235 | 1254 | UAGCCAAGGAUGGCUUGCCU | AGGCAAUUCAUUCUUGGCUU | Cleavage |
| ccp-miR169e-5p | Cc04_g01480 | 3.0 | 17.033 | 1 | 22 | 1530 | 1551 | UAGCCAAGGAUGGCUUGCCUCU | AGUGGGAAGUCAUCCUUGGCUG | Cleavage |
| ccp-miR169e-5p | Cc06_g06710 | 3.0 | 22.167 | 1 | 22 | 1266 | 1287 | UAGCCAAGGAUGGCUUGCCUCU | AAAGGCAACUCAUUCUUGGCUU | Cleavage |
| ccp-miR169e-5p | Cc06_g22210 | 3.0 | 11.401 | 1 | 20 | 696 | 715 | UAGCCAAGGAUGGCUUGCCU | AGGUAAGACAACUUUGGCUA | Translation |
| ccp-miR169e-5p | Cc08_g01380 | 2.5 | 22.141 | 1 | 20 | 2264 | 2283 | UAGCCAAGGAUGGCUUGCCU | UGGCAGGACAUCCUUGGCUA | Cleavage |
| ccp-miR169e-5p | Cc09_g02390 | 3.0 | 21.209 | 1 | 22 | 474 | 495 | UAGCCAAGGAUGGCUUGCCUCU | ACAGGCAGGACAUCCUUGGUUU | Cleavage |
| ccp-miR169f-1-5p | Cc00_g11560 | 3.0 | 19.109 | 1 | 20 | 167 | 186 | UAGCCAAGGAUGACUUGCCU | AGGCAAUUCAACUUUGGCUG | Translation |
| ccp-miR169f-1-5p | Cc00_g19380 | 3.0 | 13.826 | 1 | 21 | 1303 | 1323 | UAGCCAAGGAUGACUUGCCUA | UUGGCAAUUCAUCCUUGGCUU | Cleavage |
| ccp-miR169f-1-5p | Cc01_g11880 | 3.0 | 17.192 | 1 | 20 | 1237 | 1256 | UAGCCAAGGAUGACUUGCCU | AGGCAGGGCAUCCUUGGUUU | Cleavage |
| ccp-miR169f-1-5p | Cc02_g05590 | 2.5 | 14.422 | 1 | 20 | 925 | 943 | UAGCCAAGGAUGACUUGCCU | AGGCAAGUCA-CUUUGGCUA | Translation |
| ccp-miR169f-1-5p | Cc02_g19530 | 3.0 | 22.558 | 1 | 21 | 1496 | 1516 | UAGCCAAGGAUGACUUGCCUA | UAGGCAUGUCCUUUUUGGCUA | Translation |
| ccp-miR169f-1-5p | Cc02_g28060 | 2.5 | 17.885 | 1 | 20 | 1235 | 1254 | UAGCCAAGGAUGACUUGCCU | AGGCAAUUCAUUCUUGGCUU | Cleavage |
| ccp-miR169f-1-5p | Cc04_g01480 | 2.5 | 17.033 | 1 | 20 | 1532 | 1551 | UAGCCAAGGAUGACUUGCCU | UGGGAAGUCAUCCUUGGCUG | Cleavage |
| ccp-miR169f-1-5p | Cc06_g06710 | 2.5 | 22.167 | 1 | 20 | 1268 | 1287 | UAGCCAAGGAUGACUUGCCU | AGGCAACUCAUUCUUGGCUU | Cleavage |
| ccp-miR169f-1-5p | Cc06_g22210 | 3.0 | 11.401 | 1 | 20 | 696 | 715 | UAGCCAAGGAUGACUUGCCU | AGGUAAGACAACUUUGGCUA | Translation |
| ccp-miR169f-1-5p | Cc08_g01380 | 2.5 | 22.141 | 1 | 21 | 2263 | 2283 | UAGCCAAGGAUGACUUGCCUA | UUGGCAGGACAUCCUUGGCUA | Cleavage |
| ccp-miR169f-1-5p | Cc09_g02390 | 3.0 | 21.209 | 1 | 20 | 476 | 495 | UAGCCAAGGAUGACUUGCCU | AGGCAGGACAUCCUUGGUUU | Cleavage |
| ccp-miR169f-2-5p | Cc00_g19380 | 2.0 | 13.826 | 1 | 21 | 1303 | 1323 | AAGCCAAGGAUGACUUGCCUA | UUGGCAAUUCAUCCUUGGCUU | Cleavage |
| ccp-miR169f-2-5p | Cc01_g11880 | 2.0 | 17.192 | 1 | 20 | 1237 | 1256 | AAGCCAAGGAUGACUUGCCU | AGGCAGGGCAUCCUUGGUUU | Cleavage |
| ccp-miR169f-2-5p | Cc02_g28060 | 1.5 | 17.885 | 1 | 20 | 1235 | 1254 | AAGCCAAGGAUGACUUGCCU | AGGCAAUUCAUUCUUGGCUU | Cleavage |
| ccp-miR169f-2-5p | Cc02_g35530 | 3.0 | 17.954 | 1 | 20 | 1376 | 1394 | AAGCCAAGGAUGACUUGCCU | AGGCAA-UCAUACUUGGCUU | Translation |
| ccp-miR169f-2-5p | Cc02_g35600 | 3.0 | 14.926 | 1 | 20 | 257 | 275 | AAGCCAAGGAUGACUUGCCU | AGGCAA-UCAUACUUGGCUU | Translation |
| ccp-miR169f-2-5p | Cc04_g01480 | 3.0 | 17.033 | 1 | 20 | 1532 | 1551 | AAGCCAAGGAUGACUUGCCU | UGGGAAGUCAUCCUUGGCUG | Cleavage |
| ccp-miR169f-2-5p | Cc06_g06710 | 1.5 | 22.167 | 1 | 20 | 1268 | 1287 | AAGCCAAGGAUGACUUGCCU | AGGCAACUCAUUCUUGGCUU | Cleavage |
| ccp-miR169f-2-5p | Cc07_g04880 | 2.5 | 15.524 | 1 | 20 | 2312 | 2331 | AAGCCAAGGAUGACUUGCCU | AGGAAACUCGUCCUUGGCUU | Cleavage |
| ccp-miR169f-2-5p | Cc07_g20360 | 2.5 | 17.023 | 1 | 20 | 1149 | 1168 | AAGCCAAGGAUGACUUGCCU | AGGCAAGUCAGACUUGGUUU | Translation |
| ccp-miR169f-2-5p | Cc09_g02390 | 2.0 | 21.209 | 1 | 20 | 476 | 495 | AAGCCAAGGAUGACUUGCCU | AGGCAGGACAUCCUUGGUUU | Cleavage |
| ccp-miR169g-5p | Cc00_g11560 | 3.0 | 19.109 | 1 | 20 | 167 | 186 | UAGCCAAGGAUGACUUGCCU | AGGCAAUUCAACUUUGGCUG | Translation |
| ccp-miR169g-5p | Cc00_g19380 | 3.0 | 13.826 | 1 | 20 | 1304 | 1323 | UAGCCAAGGAUGACUUGCCU | UGGCAAUUCAUCCUUGGCUU | Cleavage |
| ccp-miR169g-5p | Cc01_g11880 | 3.0 | 17.192 | 1 | 22 | 1235 | 1256 | UAGCCAAGGAUGACUUGCCUGC | GCAGGCAGGGCAUCCUUGGUUU | Cleavage |
| ccp-miR169g-5p | Cc02_g05590 | 2.5 | 14.422 | 1 | 20 | 925 | 943 | UAGCCAAGGAUGACUUGCCU | AGGCAAGUCA-CUUUGGCUA | Translation |
| ccp-miR169g-5p | Cc02_g19530 | 3.0 | 22.558 | 1 | 21 | 1496 | 1516 | UAGCCAAGGAUGACUUGCCUG | UAGGCAUGUCCUUUUUGGCUA | Translation |
| ccp-miR169g-5p | Cc02_g28060 | 2.5 | 17.885 | 1 | 21 | 1234 | 1254 | UAGCCAAGGAUGACUUGCCUG | CAGGCAAUUCAUUCUUGGCUU | Cleavage |
| ccp-miR169g-5p | Cc04_g01480 | 2.5 | 17.033 | 1 | 20 | 1532 | 1551 | UAGCCAAGGAUGACUUGCCU | UGGGAAGUCAUCCUUGGCUG | Cleavage |
| ccp-miR169g-5p | Cc06_g06710 | 2.5 | 22.167 | 1 | 20 | 1268 | 1287 | UAGCCAAGGAUGACUUGCCU | AGGCAACUCAUUCUUGGCUU | Cleavage |
| ccp-miR169g-5p | Cc06_g22210 | 3.0 | 11.401 | 1 | 20 | 696 | 715 | UAGCCAAGGAUGACUUGCCU | AGGUAAGACAACUUUGGCUA | Translation |
| ccp-miR169g-5p | Cc08_g01380 | 2.5 | 22.141 | 1 | 20 | 2264 | 2283 | UAGCCAAGGAUGACUUGCCU | UGGCAGGACAUCCUUGGCUA | Cleavage |
| ccp-miR169g-5p | Cc09_g02390 | 3.0 | 21.209 | 1 | 21 | 475 | 495 | UAGCCAAGGAUGACUUGCCUG | CAGGCAGGACAUCCUUGGUUU | Cleavage |
| ccp-miR171-10-3p | Cc02_g24390 | 1.0 | 21.288 | 1 | 21 | 1431 | 1451 | AGAUUGAGCCGCGCCAAUAUC | GAUAUUGGCGCGGCUCAAUCA | Cleavage |
| ccp-miR171-10-3p | Cc08_g12670 | 1.0 | 16.081 | 1 | 21 | 1948 | 1968 | AGAUUGAGCCGCGCCAAUAUC | GAUAUUGGCGCGGCUCAAUCA | Cleavage |
| ccp-miR171-10-3p | Cc10_g10660 | 3.0 | 24.505 | 1 | 20 | 1151 | 1170 | AGAUUGAGCCGCGCCAAUAU | AUCUUGGUGUGGCUCAGUUU | Cleavage |
| ccp-miR171-10-5p | Cc00_g16930 | 2.5 | 19.621 | 1 | 20 | 258 | 277 | UAUUGGUGAGGUUCAAUCCG | CAGGUUGAACCUUACUAAUA | Cleavage |
| ccp-miR171-10-5p | Cc00_g28410 | 3.0 | 10.371 | 1 | 20 | 35 | 55 | UAUUGGUGAGGUUCA-AUCCG | CGGAUGUGAUCCUCACCAAUA | Cleavage |
| ccp-miR171-10-5p | Cc00_g34440 | 3.0 | 16.282 | 1 | 20 | 78 | 97 | UAUUGGUGAGGUUCAAUCCG | CAGAUUGAACCUCACAAAUG | Cleavage |
| ccp-miR171-10-5p | Cc04_g04750 | 2.5 | 11.287 | 1 | 20 | 291 | 310 | UAUUGGUGAGGUUCAAUCCG | UGGAUGGAACAUCACCAAUA | Translation |
| ccp-miR171-10-5p | Cc04_g06920 | 3.0 | 17.306 | 1 | 20 | 2796 | 2814 | UAUUGGUGAGGUUCAAUCCG | CGGAUUGAACCU-ACCAAUC | Cleavage |
| ccp-miR171-10-5p | Cc05_g08390 | 3.0 | 10.347 | 1 | 20 | 35 | 55 | UAUUGGUGAGGUUCA-AUCCG | CGGAUGUGAUCCUCACCAAUA | Cleavage |
| ccp-miR171-10-5p | Cc05_g15790 | 3.0 | 19.366 | 1 | 20 | 3364 | 3383 | UAUUGGUGAGGUUCAAUCCG | UGGGUUGAACCUCUCCAAUG | Cleavage |
| ccp-miR171-10-5p | Cc06_g03420 | 3.0 | 19.382 | 1 | 21 | 1329 | 1349 | UAUUGGUGAGGUUCAAUCCGA | UUGUGUUGAAGCUCACCAAUA | Translation |
| ccp-miR171-10-5p | Cc07_g03010 | 2.5 | 15.405 | 1 | 20 | 1753 | 1772 | UAUUGGUGAGGUUCAAUCCG | UGGAUUGAGCUUCACCAAUU | Cleavage |
| ccp-miR171-11-3p | Cc01_g06780 | 3.0 | 14.184 | 1 | 21 | 1191 | 1211 | GAUUGAACCUCACCAAUAUCG | UGAUGAUGGUGAGGUUCAAAC | Cleavage |
| ccp-miR171-11-3p | Cc08_g10660 | 2.0 | 10.821 | 1 | 20 | 1617 | 1636 | GAUUGAACCUCACCAAUAUC | AAUAUUGGUGGGGUUUAAUC | Cleavage |
| ccp-miR171-11-5p | Cc01_g06720 | 3.0 | 20.341 | 1 | 20 | 442 | 461 | GAUAUUGGCGCGGCUCAAUC | GAUGGAGCUGCAUCAAUAUC | Translation |
| ccp-miR171-11-5p | Cc01_g07920 | 3.0 | 15.544 | 1 | 20 | 2034 | 2053 | GAUAUUGGCGCGGCUCAAUC | GAUUGAGGUGCGUCAGUGUC | Cleavage |
| ccp-miR171-11-5p | Cc04_g09230 | 3.0 | 14.069 | 1 | 20 | 2370 | 2389 | GAUAUUGGCGCGGCUCAAUC | GAUUUAGCUUUGCCAAUAUC | Translation |
| ccp-miR-171-12-3p | Cc02_g22520 | 3.0 | 12.758 | 1 | 21 | 2738 | 2758 | UGAUUGAACCGUGCCAACAUC | GAUUUUGGCACGCUUCAGUCG | Translation |
| ccp-miR-171-12-3p | Cc02_g24390 | 2.5 | 21.288 | 1 | 21 | 1431 | 1451 | UGAUUGAACCGUGCCAACAUC | GAUAUUGGCGCGGCUCAAUCA | Cleavage |
| ccp-miR-171-12-3p | Cc06_g18160 | 2.5 | 11.51 | 1 | 21 | 1724 | 1744 | UGAUUGAACCGUGCCAACAUC | GAUGUUGGUACCAUUCAAUCA | Translation |
| ccp-miR-171-12-3p | Cc06_g18170 | 2.5 | 13.872 | 1 | 21 | 1688 | 1708 | UGAUUGAACCGUGCCAACAUC | GAUGUUGGUACCAUUCAAUCA | Translation |
| ccp-miR-171-12-3p | Cc08_g12670 | 2.5 | 16.081 | 1 | 21 | 1948 | 1968 | UGAUUGAACCGUGCCAACAUC | GAUAUUGGCGCGGCUCAAUCA | Cleavage |
| ccp-miR171-12-5p | Cc01_g10640 | 3.0 | 20.001 | 1 | 20 | 502 | 521 | CGAUAUUGGCACGGCUCAAU | UUUGUGUCGUGCCAAUAUUG | Cleavage |
| ccp-miR171-12-5p | Cc08_g06900 | 3.0 | 20.774 | 1 | 20 | 296 | 315 | CGAUAUUGGCACGGCUCAAU | AUUGGGCCUUGCUAAUAUCA | Cleavage |
| ccp-miR171-13-3p | Cc02_g24390 | 2.5 | 20.699 | 1 | 21 | 1429 | 1449 | AUUGAACCGCACCAAUAUCCC | GGGAUAUUGGCGCGGCUCAAU | Translation |
| ccp-miR171-13-3p | Cc08_g12670 | 2.5 | 15.819 | 1 | 21 | 1946 | 1966 | AUUGAACCGCACCAAUAUCCC | GGGAUAUUGGCGCGGCUCAAU | Translation |
| ccp-miR171-1-3p | Cc02_g24390 | 0.5 | 21.288 | 1 | 21 | 1431 | 1451 | UGAUUGAGCCGUGCCAAUAUC | GAUAUUGGCGCGGCUCAAUCA | Cleavage |
| ccp-miR171-1-3p | Cc07_g04680 | 2.5 | 14.942 | 1 | 21 | 364 | 384 | UGAUUGAGCCGUGCCAAUAUC | GAUAUUGCAAUGGCUCAAUCA | Cleavage |
| ccp-miR171-1-3p | Cc07_g18230 | 3.0 | 22.752 | 1 | 21 | 1483 | 1503 | UGAUUGAGCCGUGCCAAUAUC | GAAAUUGACAGGGCUCAAUCA | Translation |
| ccp-miR171-1-3p | Cc08_g10660 | 3.0 | 11.037 | 1 | 20 | 1618 | 1637 | UGAUUGAGCCGUGCCAAUAU | AUAUUGGUGGGGUUUAAUCA | Translation |
| ccp-miR171-1-3p | Cc08_g12670 | 0.5 | 16.081 | 1 | 21 | 1948 | 1968 | UGAUUGAGCCGUGCCAAUAUC | GAUAUUGGCGCGGCUCAAUCA | Cleavage |
| ccp-miR171-1-3p | Cc08_g15610 | 2.0 | 18.097 | 1 | 20 | 3527 | 3546 | UGAUUGAGCCGUGCCAAUAU | AUAUUGGCAUGGCUCUAUCA | Cleavage |
| ccp-miR171-2-3p | Cc04_g09230 | 2.5 | 14.069 | 1 | 21 | 2369 | 2389 | GAUAUUGGCACGGCUCAAUCA | UGAUUUAGCUUUGCCAAUAUC | Translation |
| ccp-miR171-2-3p | Cc08_g06900 | 3.0 | 21.811 | 1 | 20 | 295 | 314 | GAUAUUGGCACGGCUCAAUC | AAUUGGGCCUUGCUAAUAUC | Translation |
| ccp-miR171-2-5p | Cc04_g09230 | 2.5 | 14.069 | 1 | 20 | 2370 | 2389 | GAUAUUGGCACGGCUCAAUC | GAUUUAGCUUUGCCAAUAUC | Translation |
| ccp-miR171-2-5p | Cc08_g06900 | 3.0 | 21.811 | 1 | 20 | 295 | 314 | GAUAUUGGCACGGCUCAAUC | AAUUGGGCCUUGCUAAUAUC | Translation |
| ccp-miR171-3-3p | Cc02_g24390 | 0.5 | 21.288 | 1 | 21 | 1431 | 1451 | UGAUUGAGCCGUGCCAAUAUC | GAUAUUGGCGCGGCUCAAUCA | Cleavage |
| ccp-miR171-3-3p | Cc07_g04680 | 2.5 | 14.942 | 1 | 21 | 364 | 384 | UGAUUGAGCCGUGCCAAUAUC | GAUAUUGCAAUGGCUCAAUCA | Cleavage |
| ccp-miR171-3-3p | Cc07_g18230 | 3.0 | 22.752 | 1 | 21 | 1483 | 1503 | UGAUUGAGCCGUGCCAAUAUC | GAAAUUGACAGGGCUCAAUCA | Translation |
| ccp-miR171-3-3p | Cc08_g10660 | 3.0 | 11.037 | 1 | 20 | 1618 | 1637 | UGAUUGAGCCGUGCCAAUAU | AUAUUGGUGGGGUUUAAUCA | Translation |
| ccp-miR171-3-3p | Cc08_g12670 | 0.5 | 16.081 | 1 | 21 | 1948 | 1968 | UGAUUGAGCCGUGCCAAUAUC | GAUAUUGGCGCGGCUCAAUCA | Cleavage |
| ccp-miR171-3-3p | Cc08_g15610 | 2.0 | 18.097 | 1 | 20 | 3527 | 3546 | UGAUUGAGCCGUGCCAAUAU | AUAUUGGCAUGGCUCUAUCA | Cleavage |
| ccp-miR171-4-3p | Cc02_g24390 | 0.5 | 21.288 | 1 | 21 | 1431 | 1451 | UGAUUGAGCCGUGCCAAUAUC | GAUAUUGGCGCGGCUCAAUCA | Cleavage |
| ccp-miR171-4-3p | Cc07_g04680 | 2.5 | 14.942 | 1 | 21 | 364 | 384 | UGAUUGAGCCGUGCCAAUAUC | GAUAUUGCAAUGGCUCAAUCA | Cleavage |
| ccp-miR171-4-3p | Cc07_g18230 | 3.0 | 22.752 | 1 | 21 | 1483 | 1503 | UGAUUGAGCCGUGCCAAUAUC | GAAAUUGACAGGGCUCAAUCA | Translation |
| ccp-miR171-4-3p | Cc08_g10660 | 3.0 | 11.037 | 1 | 20 | 1618 | 1637 | UGAUUGAGCCGUGCCAAUAU | AUAUUGGUGGGGUUUAAUCA | Translation |
| ccp-miR171-4-3p | Cc08_g12670 | 0.5 | 16.081 | 1 | 21 | 1948 | 1968 | UGAUUGAGCCGUGCCAAUAUC | GAUAUUGGCGCGGCUCAAUCA | Cleavage |
| ccp-miR171-4-3p | Cc08_g15610 | 2.0 | 18.097 | 1 | 20 | 3527 | 3546 | UGAUUGAGCCGUGCCAAUAU | AUAUUGGCAUGGCUCUAUCA | Cleavage |
| ccp-miR171-4-5p | Cc06_g18180 | 2.5 | 18.467 | 1 | 20 | 3322 | 3341 | UGUUGGCACGGUUCAAUCAC | GUGAUUGAACUGUGCGAGCA | Cleavage |
| ccp-miR171-5-3p | Cc02_g24390 | 0.0 | 21.288 | 1 | 21 | 1431 | 1451 | UGAUUGAGCCGCGCCAAUAUC | GAUAUUGGCGCGGCUCAAUCA | Cleavage |
| ccp-miR171-5-3p | Cc02_g31340 | 3.0 | 13.646 | 1 | 21 | 1153 | 1173 | UGAUUGAGCCGCGCCAAUAUC | GAUACUGGAGCUGCUCAAUCA | Translation |
| ccp-miR171-5-3p | Cc08_g10660 | 2.5 | 11.037 | 1 | 20 | 1618 | 1637 | UGAUUGAGCCGCGCCAAUAU | AUAUUGGUGGGGUUUAAUCA | Translation |
| ccp-miR171-5-3p | Cc08_g12670 | 0.0 | 16.081 | 1 | 21 | 1948 | 1968 | UGAUUGAGCCGCGCCAAUAUC | GAUAUUGGCGCGGCUCAAUCA | Cleavage |
| ccp-miR171-5-3p | Cc08_g15610 | 3.0 | 18.097 | 1 | 20 | 3527 | 3546 | UGAUUGAGCCGCGCCAAUAU | AUAUUGGCAUGGCUCUAUCA | Cleavage |
| ccp-miR171-5-5p | Cc00_g32070 | 3.0 | 17.971 | 1 | 20 | 1835 | 1854 | AGAUAUUGGUCCGGUUCAAU | AUUGAGCUGGUGCAAUAUCU | Translation |
| ccp-miR171-5-5p | Cc02_g06960 | 2.5 | 16.146 | 1 | 20 | 281 | 300 | AGAUAUUGGUCCGGUUCAAU | AUUCAACUGGACUAGUAUCU | Cleavage |
| ccp-miR171-5-5p | Cc07_g07750 | 3.0 | 16.752 | 1 | 20 | 1206 | 1225 | AGAUAUUGGUCCGGUUCAAU | AUUGAACUCGAGCAAUAUUU | Translation |
| ccp-miR171-6-3p | Cc00_g08660 | 3.0 | 19.941 | 1 | 20 | 3600 | 3619 | UGAUUGAACCGGACCAAUAU | AUAUUGUUUCGGUUCACUCA | Cleavage |
| ccp-miR171-6-3p | Cc02_g24390 | 3.0 | 21.288 | 1 | 21 | 1431 | 1451 | UGAUUGAACCGGACCAAUAUC | GAUAUUGGCGCGGCUCAAUCA | Cleavage |
| ccp-miR171-6-3p | Cc04_g14940 | 3.0 | 17.473 | 1 | 20 | 397 | 416 | UGAUUGAACCGGACCAAUAU | AUAUUGUUCCGAUUCGAUUA | Translation |
| ccp-miR171-6-3p | Cc06_g04580 | 3.0 | 21.843 | 1 | 20 | 1572 | 1591 | UGAUUGAACCGGACCAAUAU | AUGGUGGUUCUGUUCAAUCA | Translation |
| ccp-miR171-6-3p | Cc08_g10660 | 2.5 | 11.037 | 1 | 20 | 1618 | 1637 | UGAUUGAACCGGACCAAUAU | AUAUUGGUGGGGUUUAAUCA | Translation |
| ccp-miR171-6-3p | Cc08_g12670 | 3.0 | 16.081 | 1 | 21 | 1948 | 1968 | UGAUUGAACCGGACCAAUAUC | GAUAUUGGCGCGGCUCAAUCA | Cleavage |
| ccp-miR171-6-5p | Cc01_g06720 | 3.0 | 20.341 | 1 | 20 | 442 | 461 | GAUAUUGGCGCGGCUCAAUC | GAUGGAGCUGCAUCAAUAUC | Translation |
| ccp-miR171-6-5p | Cc01_g07920 | 3.0 | 15.544 | 1 | 20 | 2034 | 2053 | GAUAUUGGCGCGGCUCAAUC | GAUUGAGGUGCGUCAGUGUC | Cleavage |
| ccp-miR171-6-5p | Cc04_g09230 | 3.0 | 14.069 | 1 | 20 | 2370 | 2389 | GAUAUUGGCGCGGCUCAAUC | GAUUUAGCUUUGCCAAUAUC | Translation |
| ccp-miR171-7-5p | Cc01_g14000 | 3.0 | 23.063 | 1 | 21 | 344 | 364 | UAUUGGUGCGGUUCAAUGGCA | UCCCAUUGAACCGCGCCAGUU | Cleavage |
| ccp-miR171-8-3p | Cc02_g24390 | 0.5 | 21.288 | 1 | 21 | 1431 | 1451 | UGAUUGAGCCGUGCCAAUAUC | GAUAUUGGCGCGGCUCAAUCA | Cleavage |
| ccp-miR171-8-3p | Cc07_g04680 | 2.5 | 14.942 | 1 | 21 | 364 | 384 | UGAUUGAGCCGUGCCAAUAUC | GAUAUUGCAAUGGCUCAAUCA | Cleavage |
| ccp-miR171-8-3p | Cc07_g18230 | 3.0 | 22.752 | 1 | 21 | 1483 | 1503 | UGAUUGAGCCGUGCCAAUAUC | GAAAUUGACAGGGCUCAAUCA | Translation |
| ccp-miR171-8-3p | Cc08_g10660 | 3.0 | 11.037 | 1 | 20 | 1618 | 1637 | UGAUUGAGCCGUGCCAAUAU | AUAUUGGUGGGGUUUAAUCA | Translation |
| ccp-miR171-8-3p | Cc08_g12670 | 0.5 | 16.081 | 1 | 21 | 1948 | 1968 | UGAUUGAGCCGUGCCAAUAUC | GAUAUUGGCGCGGCUCAAUCA | Cleavage |
| ccp-miR171-8-3p | Cc08_g15610 | 2.0 | 18.097 | 1 | 20 | 3527 | 3546 | UGAUUGAGCCGUGCCAAUAU | AUAUUGGCAUGGCUCUAUCA | Cleavage |
| ccp-miR171-9-3p | Cc00_g14930 | 3.0 | 22.151 | 1 | 21 | 2722 | 2742 | UGAUUGAGCCGCGUCAAUAUC | GGUAUUGAAGCUGUUCAAUCA | Translation |
| ccp-miR171-9-3p | Cc02_g24390 | 0.5 | 21.288 | 1 | 21 | 1431 | 1451 | UGAUUGAGCCGCGUCAAUAUC | GAUAUUGGCGCGGCUCAAUCA | Cleavage |
| ccp-miR171-9-3p | Cc07_g18230 | 3.0 | 22.752 | 1 | 21 | 1483 | 1503 | UGAUUGAGCCGCGUCAAUAUC | GAAAUUGACAGGGCUCAAUCA | Translation |
| ccp-miR171-9-3p | Cc08_g10660 | 3.0 | 11.037 | 1 | 20 | 1618 | 1637 | UGAUUGAGCCGCGUCAAUAU | AUAUUGGUGGGGUUUAAUCA | Translation |
| ccp-miR171-9-3p | Cc08_g12670 | 0.5 | 16.081 | 1 | 21 | 1948 | 1968 | UGAUUGAGCCGCGUCAAUAUC | GAUAUUGGCGCGGCUCAAUCA | Cleavage |
| ccp-miR171-9-5p | Cc00_g19440 | 3.0 | 15.433 | 1 | 21 | 1875 | 1895 | AGGUAUUGAUGCGCCUCAAUC | GAUUGAGGUGCAUGGAUGUCU | Cleavage |
| ccp-miR171-9-5p | Cc00_g25340 | 3.0 | 22.604 | 1 | 21 | 2580 | 2600 | AGGUAUUGAUGCGCCUCAAUC | GAUUGAGGUGCAUGGAUGUCU | Cleavage |
| ccp-miR171-9-5p | Cc01_g08050 | 3.0 | 17.114 | 1 | 21 | 2148 | 2168 | AGGUAUUGAUGCGCCUCAAUC | GAUUGAGGUGCAUCGAUGUCC | Cleavage |
| ccp-miR171-9-5p | Cc01_g08070 | 3.0 | 16.389 | 1 | 21 | 4905 | 4925 | AGGUAUUGAUGCGCCUCAAUC | GAUUGAGGUGCAUGGAUGUCU | Cleavage |
| ccp-miR171-9-5p | Cc01_g08120 | 3.0 | 17.443 | 1 | 21 | 2301 | 2321 | AGGUAUUGAUGCGCCUCAAUC | GAUUGCGGUGCAUCGAUGUCU | Cleavage |
| ccp-miR171-9-5p | Cc09_g07580 | 2.5 | 12.538 | 1 | 20 | 263 | 282 | AGGUAUUGAUGCGCCUCAAU | AUUGGAGCGCAUCAAUGUCU | Cleavage |
| ccp-miR171b-1-3p | Cc02_g12890 | 3.0 | 14.392 | 1 | 20 | 1460 | 1479 | UAAUUGAACCGCACCAAUAU | AUCUUGGUGGAGUUCAAUUA | Translation |
| ccp-miR171b-1-3p | Cc08_g10660 | 3.0 | 11.037 | 1 | 20 | 1618 | 1637 | UAAUUGAACCGCACCAAUAU | AUAUUGGUGGGGUUUAAUCA | Translation |
| ccp-miR171b-1-3p | Cc09_g09020 | 3.0 | 19.948 | 1 | 20 | 868 | 887 | UAAUUGAACCGCACCAAUAU | AUUUUGGUGUGCUUCAAUUG | Translation |
| ccp-miR171b-2-5p | Cc01_g06720 | 2.5 | 20.341 | 1 | 20 | 442 | 461 | GAUAUUGACGCGGCUCAAUC | GAUGGAGCUGCAUCAAUAUC | Translation |
| ccp-miR171b-2-5p | Cc01_g07920 | 2.5 | 15.544 | 1 | 20 | 2034 | 2053 | GAUAUUGACGCGGCUCAAUC | GAUUGAGGUGCGUCAGUGUC | Cleavage |
| ccp-miR171f-3p | Cc01_g20550 | 3.0 | 19.05 | 1 | 20 | 127 | 146 | AUGAGCCGAACCAAUAUCAC | GUGAUUUUGGUUUGGUUCAG | Cleavage |
| ccp-miR171f-3p | Cc03_g16170 | 2.5 | 16.004 | 1 | 20 | 1934 | 1953 | AUGAGCCGAACCAAUAUCAC | GUGAUAUUGGUUGGGCUCUU | Cleavage |
| ccp-miR171f-3p | Cc06_g09190 | 3.0 | 14.351 | 1 | 20 | 892 | 911 | AUGAGCCGAACCAAUAUCAC | GAGGUAUUGGAUCGGUUCAU | Translation |
| ccp-miR172-1-3p | Cc01_g11840 | 1.5 | 13.751 | 1 | 20 | 1877 | 1896 | AGAAUCUUGAUGAUGCUCCA | UGCAGCAUCAUCAGGAUUCU | Cleavage |
| ccp-miR172-1-3p | Cc06_g04100 | 3.0 | 11.57 | 1 | 20 | 1698 | 1717 | AGAAUCUUGAUGAUGCUCCA | UAGGGCAUCAUCAAGAUUUA | Cleavage |
| ccp-miR172-1-3p | Cc07_g05500 | 3.0 | 13.901 | 1 | 21 | 1073 | 1093 | AGAAUCUUGAUGAUGCUCCAC | GUUGAGCACCAUCAAGGUUUU | Cleavage |
| ccp-miR172-1-3p | Cc07_g06200 | 2.5 | 14.173 | 1 | 20 | 1827 | 1846 | AGAAUCUUGAUGAUGCUCCA | UGCAGCAUCAUCAGGAUUCC | Cleavage |
| ccp-miR172-1-3p | Cc07_g09990 | 3.0 | 16.733 | 1 | 20 | 1530 | 1549 | AGAAUCUUGAUGAUGCUCCA | UCGAUUAUCAUCAAGAUUUU | Cleavage |
| ccp-miR172-1-3p | Cc08_g09580 | 3.0 | 13.375 | 1 | 20 | 3233 | 3252 | AGAAUCUUGAUGAUGCUCCA | UGAAGCAGCAUCAAGAUUCC | Cleavage |
| ccp-miR172-1-3p | Cc08_g14760 | 3.0 | 14.993 | 1 | 20 | 855 | 874 | AGAAUCUUGAUGAUGCUCCA | UGAAGCAUCAUAAGGAUUUU | Translation |
| ccp-miR172-1-3p | Cc09_g02450 | 1.5 | 15.971 | 1 | 20 | 1527 | 1546 | AGAAUCUUGAUGAUGCUCCA | UGCAGCAUCAUCAGGAUUCU | Cleavage |
| ccp-miR172-1-3p | Cc10_g00420 | 3.0 | 21.39 | 1 | 21 | 1951 | 1971 | AGAAUCUUGAUGAUGCUCCAC | GUGGAGUUUGAUCAAGAUUUU | Cleavage |
| ccp-miR172-1-5p | Cc00_g05770 | 3.0 | 13.046 | 1 | 21 | 543 | 563 | GCAGCAUCAUCAAGAUUCCCG | UGGGAAAUUUGAAGAUGUUGC | Translation |
| ccp-miR172-1-5p | Cc00_g09120 | 3.0 | 16.069 | 1 | 20 | 4583 | 4602 | GCAGCAUCAUCAAGAUUCCC | AGGAGUCUUGGAGAUGCUGC | Translation |
| ccp-miR172-1-5p | Cc00_g10740 | 3.0 | 14.123 | 1 | 20 | 418 | 437 | GCAGCAUCAUCAAGAUUCCC | AGGAAACUUGAAGAUGCUGC | Translation |
| ccp-miR172-1-5p | Cc00_g13080 | 3.0 | 15.033 | 1 | 21 | 1590 | 1610 | GCAGCAUCAUCAAGAUUCCCG | UGGGAGUCUAAGUGAUGCUGC | Translation |
| ccp-miR172-1-5p | Cc00_g13960 | 2.5 | 19.049 | 1 | 21 | 801 | 821 | GCAGCAUCAUCAAGAUUCCCG | UGGGAGUUUGGAUGAUGCUGU | Cleavage |
| ccp-miR172-1-5p | Cc00_g35530 | 3.0 | 14.138 | 1 | 20 | 733 | 752 | GCAGCAUCAUCAAGAUUCCC | AGGAAACUUGAAGAUGCUGC | Translation |
| ccp-miR172-1-5p | Cc02_g18720 | 3.0 | 14.371 | 1 | 21 | 1233 | 1254 | GCAGCAUCA-UCAAGAUUCCCG | CAGGAGUCUUGGGUGAUGCUGC | Translation |
| ccp-miR172-1-5p | Cc02_g21190 | 2.5 | 18.06 | 1 | 20 | 4 | 23 | GCAGCAUCAUCAAGAUUCCC | GGGAACAUUGAUGAUGCUGU | Cleavage |
| ccp-miR172-1-5p | Cc02_g35070 | 3.0 | 15.08 | 1 | 21 | 316 | 336 | GCAGCAUCAUCAAGAUUCCCG | UGAGGAUAUUGGUGAUGCUGC | Cleavage |
| ccp-miR172-1-5p | Cc03_g00800 | 2.5 | 18.82 | 1 | 21 | 1347 | 1367 | GCAGCAUCAUCAAGAUUCCCG | UGGGGCUAUUGAUGAUGCUGC | Cleavage |
| ccp-miR172-1-5p | Cc03_g09420 | 3.0 | 22.626 | 1 | 21 | 647 | 667 | GCAGCAUCAUCAAGAUUCCCG | CGGGAUCCUUGUUGAUGCUGC | Translation |
| ccp-miR172-1-5p | Cc04_g04580 | 2.5 | 13.193 | 1 | 20 | 2889 | 2908 | GCAGCAUCAUCAAGAUUCCC | GGGAAGCUUGAAGAUGCUGU | Translation |
| ccp-miR172-1-5p | Cc04_g04890 | 2.0 | 19.249 | 1 | 21 | 1217 | 1237 | GCAGCAUCAUCAAGAUUCCCG | UGGGAAUAUUGUUGAUGCUGC | Translation |
| ccp-miR172-1-5p | Cc04_g07810 | 3.0 | 13.505 | 1 | 20 | 557 | 576 | GCAGCAUCAUCAAGAUUCCC | AGGAAUCUGGAUGAUGUUGU | Cleavage |
| ccp-miR172-1-5p | Cc04_g10640 | 2.0 | 12.82 | 1 | 20 | 682 | 701 | GCAGCAUCAUCAAGAUUCCC | GGAAAUCUUGUUGAUGCUGC | Translation |
| ccp-miR172-1-5p | Cc05_g00360 | 3.0 | 14.686 | 1 | 21 | 1866 | 1886 | GCAGCAUCAUCAAGAUUCCCG | CGAGGACUUUGAUGAUGCUGC | Cleavage |
| ccp-miR172-1-5p | Cc05_g15020 | 3.0 | 21.525 | 1 | 21 | 9167 | 9187 | GCAGCAUCAUCAAGAUUCCCG | UGGGAAUAUUGGUGAUGUUGA | Cleavage |
| ccp-miR172-1-5p | Cc06_g02350 | 3.0 | 17.775 | 1 | 20 | 1042 | 1061 | GCAGCAUCAUCAAGAUUCCC | GGAAAUUUUGGUGAUGCUGA | Cleavage |
| ccp-miR172-1-5p | Cc06_g21860 | 3.0 | 15.556 | 1 | 20 | 1367 | 1386 | GCAGCAUCAUCAAGAUUCCC | CGGAAACUUGAAGAUGCUGC | Translation |
| ccp-miR172-1-5p | Cc06_g21870 | 3.0 | 16.309 | 1 | 20 | 760 | 779 | GCAGCAUCAUCAAGAUUCCC | AGGAAACUUGAAGAUGCUGC | Translation |
| ccp-miR172-1-5p | Cc06_g21980 | 3.0 | 16.307 | 1 | 20 | 790 | 809 | GCAGCAUCAUCAAGAUUCCC | AGGAAACUUGAAGAUGCUGC | Translation |
| ccp-miR172-1-5p | Cc06_g21990 | 3.0 | 12.691 | 1 | 20 | 707 | 726 | GCAGCAUCAUCAAGAUUCCC | AGGAAACUUGAAGAUGCUGC | Translation |
| ccp-miR172-1-5p | Cc06_g22000 | 2.0 | 16.864 | 1 | 20 | 1487 | 1506 | GCAGCAUCAUCAAGAUUCCC | AGGAAUCUUGAGGAUGCUGC | Translation |
| ccp-miR172-1-5p | Cc06_g22030 | 3.0 | 14.123 | 1 | 20 | 429 | 448 | GCAGCAUCAUCAAGAUUCCC | AGGAAACUUGAAGAUGCUGC | Translation |
| ccp-miR172-1-5p | Cc07_g08410 | 2.5 | 16.129 | 1 | 20 | 2563 | 2582 | GCAGCAUCAUCAAGAUUCCC | GGUGAUCUUGGUGAUGUUGC | Cleavage |
| ccp-miR172-1-5p | Cc07_g08780 | 3.0 | 20.961 | 1 | 21 | 1155 | 1175 | GCAGCAUCAUCAAGAUUCCCG | UGAGAUUCUUGAUGGUGUUGC | Cleavage |
| ccp-miR172-1-5p | Cc07_g10580 | 3.0 | 23.829 | 1 | 20 | 991 | 1010 | GCAGCAUCAUCAAGAUUCCC | GGCAAUGUGGAUGAUGCUGC | Cleavage |
| ccp-miR172-1-5p | Cc07_g19820 | 3.0 | 16.571 | 1 | 20 | 2095 | 2114 | GCAGCAUCAUCAAGAUUCCC | AGGAAACUUGAAGAUGCUGC | Translation |
| ccp-miR172-1-5p | Cc07_g19830 | 3.0 | 16.571 | 1 | 20 | 524 | 543 | GCAGCAUCAUCAAGAUUCCC | AGGAAACUUGAAGAUGCUGC | Translation |
| ccp-miR172-1-5p | Cc11_g02140 | 3.0 | 14.448 | 1 | 20 | 781 | 800 | GCAGCAUCAUCAAGAUUCCC | AGGAAACUUGAAGAUGCUGC | Translation |
| ccp-miR172-1-5p | Cc11_g02160 | 2.0 | 16.147 | 1 | 20 | 1180 | 1199 | GCAGCAUCAUCAAGAUUCCC | AGGAAUCUUGAGGAUGCUGC | Translation |
| ccp-miR172-2-3p | Cc01_g11840 | 1.0 | 13.751 | 1 | 20 | 1877 | 1896 | GGAAUCUUGAUGAUGCUGCA | UGCAGCAUCAUCAGGAUUCU | Cleavage |
| ccp-miR172-2-3p | Cc01_g16800 | 3.0 | 15.003 | 1 | 24 | 174 | 197 | GGAAUCUUGAUGAUGCUGCAUCAG | CUUCUUCAGCAUUGUCAAGAUUCA | Cleavage |
| ccp-miR172-2-3p | Cc02_g22390 | 2.5 | 12.737 | 1 | 20 | 352 | 371 | GGAAUCUUGAUGAUGCUGCA | AGCAUCAUCAUCAAGGUUCC | Cleavage |
| ccp-miR172-2-3p | Cc06_g21970 | 1.5 | 15.948 | 1 | 24 | 551 | 574 | GGAAUCUUGAUGAUGCUGCAUCAG | CUUUUGCAGCAUCCUUAAGAUUCC | Translation |
| ccp-miR172-2-3p | Cc07_g06200 | 0.5 | 14.173 | 1 | 24 | 1823 | 1846 | GGAAUCUUGAUGAUGCUGCAUCAG | CUGCUGCAGCAUCAUCAGGAUUCC | Cleavage |
| ccp-miR172-2-3p | Cc08_g09580 | 2.0 | 13.375 | 1 | 23 | 3230 | 3252 | GGAAUCUUGAUGAUGCUGCAUCA | UGAUGAAGCAGCAUCAAGAUUCC | Cleavage |
| ccp-miR172-2-3p | Cc09_g02450 | 1.0 | 15.971 | 1 | 24 | 1523 | 1546 | GGAAUCUUGAUGAUGCUGCAUCAG | CUUCUGCAGCAUCAUCAGGAUUCU | Cleavage |
| ccp-miR172-2-5p | Cc00_g19560 | 3.0 | 19.359 | 1 | 21 | 588 | 608 | GCGGCAUCAUCAAGAUUCACA | UGUGAAUGUUGGUGAUGCAGC | Cleavage |
| ccp-miR172-2-5p | Cc02_g36410 | 3.0 | 19.843 | 1 | 21 | 516 | 536 | GCGGCAUCAUCAAGAUUCACA | UGUGAAUGUUGGUGAUGCAGC | Cleavage |
| ccp-miR172-2-5p | Cc05_g07490 | 3.0 | 16.152 | 1 | 21 | 732 | 752 | GCGGCAUCAUCAAGAUUCACA | UGGUAAUCUUGAUGAUGUCGU | Cleavage |
| ccp-miR172-2-5p | Cc11_g01750 | 3.0 | 19.392 | 1 | 21 | 676 | 696 | GCGGCAUCAUCAAGAUUCACA | UGUGAAUGUUGGUGAUGCAGC | Cleavage |
| ccp-miR172-3-3p | Cc01_g11840 | 2.5 | 13.751 | 1 | 20 | 1877 | 1896 | UGAAUCUUGAUGAUGCCGCA | UGCAGCAUCAUCAGGAUUCU | Cleavage |
| ccp-miR172-3-3p | Cc01_g16800 | 3.0 | 15.003 | 1 | 20 | 178 | 197 | UGAAUCUUGAUGAUGCCGCA | UUCAGCAUUGUCAAGAUUCA | Cleavage |
| ccp-miR172-3-3p | Cc07_g06200 | 2.5 | 14.173 | 1 | 20 | 1827 | 1846 | UGAAUCUUGAUGAUGCCGCA | UGCAGCAUCAUCAGGAUUCC | Cleavage |
| ccp-miR172-3-3p | Cc09_g02450 | 2.5 | 15.971 | 1 | 20 | 1527 | 1546 | UGAAUCUUGAUGAUGCCGCA | UGCAGCAUCAUCAGGAUUCU | Cleavage |
| ccp-miR172-3-5p | Cc00_g00990 | 3.0 | 23.073 | 1 | 20 | 2938 | 2957 | CAGCAUCAUCAAGAUUCCCA | UGGGACUGAUGAUGAUGCUG | Cleavage |
| ccp-miR172-3-5p | Cc00_g04080 | 3.0 | 19.059 | 1 | 20 | 45 | 64 | CAGCAUCAUCAAGAUUCCCA | UGACAAUCUUGAUGAUGCUC | Cleavage |
| ccp-miR172-3-5p | Cc00_g05770 | 3.0 | 13.204 | 1 | 20 | 543 | 562 | CAGCAUCAUCAAGAUUCCCA | UGGGAAAUUUGAAGAUGUUG | Cleavage |
| ccp-miR172-3-5p | Cc00_g13080 | 3.0 | 15.028 | 1 | 20 | 1590 | 1609 | CAGCAUCAUCAAGAUUCCCA | UGGGAGUCUAAGUGAUGCUG | Translation |
| ccp-miR172-3-5p | Cc00_g13960 | 2.0 | 18.586 | 1 | 20 | 801 | 820 | CAGCAUCAUCAAGAUUCCCA | UGGGAGUUUGGAUGAUGCUG | Translation |
| ccp-miR172-3-5p | Cc01_g13270 | 3.0 | 19.863 | 1 | 20 | 1401 | 1420 | CAGCAUCAUCAAGAUUCCCA | UGGCAGUUUAGAUGAUGCUG | Translation |
| ccp-miR172-3-5p | Cc01_g15920 | 3.0 | 20.208 | 1 | 20 | 933 | 952 | CAGCAUCAUCAAGAUUCCCA | UGGGGAUAUUGAUGAAGCUG | Cleavage |
| ccp-miR172-3-5p | Cc01_g16960 | 3.0 | 11.018 | 1 | 20 | 711 | 730 | CAGCAUCAUCAAGAUUCCCA | UAGGAAUUUUGGUGGUGUUG | Cleavage |
| ccp-miR172-3-5p | Cc02_g21020 | 2.5 | 16.122 | 1 | 20 | 165 | 184 | CAGCAUCAUCAAGAUUCCCA | UGGGAAUGAUGAUGAUGUUG | Cleavage |
| ccp-miR172-3-5p | Cc02_g21190 | 3.0 | 18.06 | 1 | 20 | 3 | 22 | CAGCAUCAUCAAGAUUCCCA | GGGGAACAUUGAUGAUGCUG | Cleavage |
| ccp-miR172-3-5p | Cc02_g31330 | 3.0 | 19.57 | 1 | 20 | 1620 | 1639 | CAGCAUCAUCAAGAUUCCCA | UGGGAGGCUUGAUGAGGCUG | Cleavage |
| ccp-miR172-3-5p | Cc02_g34430 | 3.0 | 23.88 | 1 | 20 | 318 | 337 | CAGCAUCAUCAAGAUUCCCA | UGGGAGGCUUGAUGGUGCUC | Cleavage |
| ccp-miR172-3-5p | Cc02_g35070 | 3.0 | 15.091 | 1 | 20 | 316 | 335 | CAGCAUCAUCAAGAUUCCCA | UGAGGAUAUUGGUGAUGCUG | Cleavage |
| ccp-miR172-3-5p | Cc02_g36350 | 3.0 | 22.257 | 1 | 20 | 351 | 370 | CAGCAUCAUCAAGAUUCCCA | UGGUCAGCUUGAUGAUGCUG | Cleavage |
| ccp-miR172-3-5p | Cc03_g00800 | 2.5 | 16.413 | 1 | 20 | 1347 | 1366 | CAGCAUCAUCAAGAUUCCCA | UGGGGCUAUUGAUGAUGCUG | Cleavage |
| ccp-miR172-3-5p | Cc04_g04080 | 3.0 | 17.313 | 1 | 20 | 303 | 322 | CAGCAUCAUCAAGAUUCCCA | UAGAAAUCUUGAUGAUGCUC | Cleavage |
| ccp-miR172-3-5p | Cc04_g04580 | 3.0 | 12.956 | 1 | 20 | 2888 | 2907 | CAGCAUCAUCAAGAUUCCCA | AGGGAAGCUUGAAGAUGCUG | Cleavage |
| ccp-miR172-3-5p | Cc04_g04890 | 2.0 | 19.076 | 1 | 20 | 1217 | 1236 | CAGCAUCAUCAAGAUUCCCA | UGGGAAUAUUGUUGAUGCUG | Translation |
| ccp-miR172-3-5p | Cc04_g10640 | 3.0 | 14.192 | 1 | 20 | 681 | 700 | CAGCAUCAUCAAGAUUCCCA | AGGAAAUCUUGUUGAUGCUG | Translation |
| ccp-miR172-3-5p | Cc05_g02410 | 3.0 | 18.256 | 1 | 20 | 979 | 998 | CAGCAUCAUCAAGAUUCCCA | UGGGAAGUUUGCUGGUGCUG | Translation |
| ccp-miR172-3-5p | Cc05_g02550 | 3.0 | 16.138 | 1 | 20 | 1611 | 1630 | CAGCAUCAUCAAGAUUCCCA | UGGGAAGUUUGCUGGUGCUG | Translation |
| ccp-miR172-3-5p | Cc05_g07490 | 3.0 | 16.082 | 1 | 20 | 732 | 751 | CAGCAUCAUCAAGAUUCCCA | UGGUAAUCUUGAUGAUGUCG | Cleavage |
| ccp-miR172-3-5p | Cc05_g15020 | 2.0 | 22.561 | 1 | 20 | 9167 | 9186 | CAGCAUCAUCAAGAUUCCCA | UGGGAAUAUUGGUGAUGUUG | Cleavage |
| ccp-miR172-3-5p | Cc06_g02350 | 3.0 | 18.163 | 1 | 20 | 1041 | 1060 | CAGCAUCAUCAAGAUUCCCA | AGGAAAUUUUGGUGAUGCUG | Cleavage |
| ccp-miR172-3-5p | Cc06_g07240 | 3.0 | 17.264 | 1 | 20 | 3041 | 3060 | CAGCAUCAUCAAGAUUCCCA | UGGUGGUCUUGAUGAUGCUC | Cleavage |
| ccp-miR172-3-5p | Cc06_g13800 | 3.0 | 24.906 | 1 | 20 | 122 | 141 | CAGCAUCAUCAAGAUUCCCA | UGGGAGUUAUGGUGGUGCUG | Cleavage |
| ccp-miR172-3-5p | Cc06_g16010 | 3.0 | 15.608 | 1 | 20 | 1206 | 1225 | CAGCAUCAUCAAGAUUCCCA | UGAAGAUCUUGAUGGUGCUG | Cleavage |
| ccp-miR172-3-5p | Cc06_g17320 | 3.0 | 16.053 | 1 | 20 | 846 | 865 | CAGCAUCAUCAAGAUUCCCA | UGGGAAGUUUGCUGGUGCUG | Translation |
| ccp-miR172-3-5p | Cc06_g22000 | 3.0 | 16.81 | 1 | 20 | 1486 | 1505 | CAGCAUCAUCAAGAUUCCCA | GAGGAAUCUUGAGGAUGCUG | Cleavage |
| ccp-miR172-3-5p | Cc07_g02680 | 2.5 | 17.913 | 1 | 20 | 1308 | 1327 | CAGCAUCAUCAAGAUUCCCA | UGAUGAUCUUGAUGAUGCUG | Cleavage |
| ccp-miR172-3-5p | Cc07_g08780 | 3.0 | 20.677 | 1 | 20 | 1155 | 1174 | CAGCAUCAUCAAGAUUCCCA | UGAGAUUCUUGAUGGUGUUG | Cleavage |
| ccp-miR172-3-5p | Cc07_g14610 | 3.0 | 15.578 | 1 | 20 | 822 | 841 | CAGCAUCAUCAAGAUUCCCA | UGUGAAACUUGAUGAUGCUC | Cleavage |
| ccp-miR172-3-5p | Cc07_g15350 | 3.0 | 23.204 | 1 | 20 | 1275 | 1294 | CAGCAUCAUCAAGAUUCCCA | UGGCAGGUUUGAUGAUGCUG | Cleavage |
| ccp-miR172-3-5p | Cc08_g05300 | 3.0 | 20.11 | 1 | 20 | 348 | 367 | CAGCAUCAUCAAGAUUCCCA | UGGGUCUCUUGUUGAUGCUG | Translation |
| ccp-miR172-3-5p | Cc09_g04920 | 3.0 | 17.414 | 1 | 20 | 1626 | 1645 | CAGCAUCAUCAAGAUUCCCA | UGGUCAGCUUGAUGAUGCUG | Cleavage |
| ccp-miR172-3-5p | Cc09_g07090 | 3.0 | 21.999 | 1 | 20 | 1035 | 1055 | CAGCAUCAUC-AAGAUUCCCA | UGGGAAACUUUGAUGAUGCUG | Translation |
| ccp-miR172-3-5p | Cc10_g06290 | 3.0 | 21.573 | 1 | 20 | 696 | 715 | CAGCAUCAUCAAGAUUCCCA | UGGUGUUCUUGGUGAUGCUG | Cleavage |
| ccp-miR172-3-5p | Cc10_g12890 | 2.0 | 14.109 | 1 | 20 | 645 | 664 | CAGCAUCAUCAAGAUUCCCA | UGGGAAUCUGGAUGAUGCUA | Translation |
| ccp-miR172-3-5p | Cc11_g02160 | 3.0 | 16.23 | 1 | 20 | 1179 | 1198 | CAGCAUCAUCAAGAUUCCCA | GAGGAAUCUUGAGGAUGCUG | Cleavage |
| ccp-miR172d-1-3p | Cc01_g11840 | 1.0 | 13.751 | 1 | 21 | 1876 | 1896 | GGAAUCUUGAUGAUGCUGCAG | CUGCAGCAUCAUCAGGAUUCU | Cleavage |
| ccp-miR172d-1-3p | Cc01_g16800 | 3.0 | 15.003 | 1 | 24 | 174 | 197 | GGAAUCUUGAUGAUGCUGCAGCAG | CUUCUUCAGCAUUGUCAAGAUUCA | Cleavage |
| ccp-miR172d-1-3p | Cc02_g22390 | 2.5 | 12.737 | 1 | 21 | 351 | 371 | GGAAUCUUGAUGAUGCUGCAG | CAGCAUCAUCAUCAAGGUUCC | Cleavage |
| ccp-miR172d-1-3p | Cc06_g21970 | 1.5 | 15.948 | 1 | 24 | 551 | 574 | GGAAUCUUGAUGAUGCUGCAGCAG | CUUUUGCAGCAUCCUUAAGAUUCC | Translation |
| ccp-miR172d-1-3p | Cc07_g06200 | 0.5 | 14.173 | 1 | 24 | 1823 | 1846 | GGAAUCUUGAUGAUGCUGCAGCAG | CUGCUGCAGCAUCAUCAGGAUUCC | Cleavage |
| ccp-miR172d-1-3p | Cc08_g09580 | 2.0 | 13.375 | 1 | 23 | 3230 | 3252 | GGAAUCUUGAUGAUGCUGCAGCA | UGAUGAAGCAGCAUCAAGAUUCC | Cleavage |
| ccp-miR172d-1-3p | Cc09_g02450 | 1.0 | 15.971 | 1 | 24 | 1523 | 1546 | GGAAUCUUGAUGAUGCUGCAGCAG | CUUCUGCAGCAUCAUCAGGAUUCU | Cleavage |
| ccp-miR172d-1-5p | Cc00_g04080 | 3.0 | 16.446 | 1 | 20 | 48 | 67 | GUGGAGCAUCAUCAAGAUUC | CAAUCUUGAUGAUGCUCUGA | Cleavage |
| ccp-miR172d-1-5p | Cc00_g07300 | 2.5 | 18.446 | 1 | 23 | 146 | 168 | GUGGAGCAUCAUCAAGAUUCACG | UGUGAAUCUAUAUGAUGUUCCAC | Cleavage |
| ccp-miR172d-1-5p | Cc00_g35870 | 2.0 | 15.401 | 1 | 20 | 66 | 85 | GUGGAGCAUCAUCAAGAUUC | GAAACUUGAUGAUGCUCUGC | Cleavage |
| ccp-miR172d-1-5p | Cc01_g17230 | 3.0 | 16.408 | 1 | 21 | 910 | 930 | GUGGAGCAUCAUCAAGAUUCA | UGAUUCUUGAUGAUGUUUUGC | Cleavage |
| ccp-miR172d-1-5p | Cc03_g05480 | 2.0 | 21.694 | 1 | 20 | 582 | 601 | GUGGAGCAUCAUCAAGAUUC | GAAUCUUGAUGAGGUUCCAU | Cleavage |
| ccp-miR172d-1-5p | Cc04_g04080 | 2.0 | 17.043 | 1 | 20 | 306 | 325 | GUGGAGCAUCAUCAAGAUUC | AAAUCUUGAUGAUGCUCCAA | Cleavage |
| ccp-miR172d-1-5p | Cc04_g10350 | 2.0 | 16.887 | 1 | 24 | 605 | 628 | GUGGAGCAUCAUCAAGAUUCACGA | UCCGGAAUCUUCAUGAUGCUCUAU | Cleavage |
| ccp-miR172d-1-5p | Cc05_g08360 | 3.0 | 23.608 | 1 | 20 | 1124 | 1143 | GUGGAGCAUCAUCAAGAUUC | GGAGCUUGAUGAGGCUCUAC | Cleavage |
| ccp-miR172d-1-5p | Cc05_g14730 | 3.0 | 22.111 | 1 | 24 | 136 | 159 | GUGGAGCAUCAUCAAGAUUCACGA | UAGCGCAUCUUGAUGAUGAUCCAU | Cleavage |
| ccp-miR172d-1-5p | Cc06_g07240 | 3.0 | 17.44 | 1 | 20 | 3044 | 3063 | GUGGAGCAUCAUCAAGAUUC | UGGUCUUGAUGAUGCUCCAA | Cleavage |
| ccp-miR172d-1-5p | Cc09_g04450 | 3.0 | 17.455 | 1 | 23 | 1398 | 1420 | GUGGAGCAUCAUCAAGAUUCACG | UGUGAAAACUGAUGAUGCUCCAC | Cleavage |
| ccp-miR172d-1-5p | Cc09_g04890 | 2.0 | 16.559 | 1 | 20 | 1293 | 1312 | GUGGAGCAUCAUCAAGAUUC | GAAACUUGAUGAUGCUCUGC | Cleavage |
| ccp-miR172d-1-5p | Cc09_g05550 | 3.0 | 19.424 | 1 | 20 | 797 | 816 | GUGGAGCAUCAUCAAGAUUC | GGAACUUGAAGAUGCUUCAC | Translation |
| ccp-miR172d-1-5p | Cc10_g07530 | 3.0 | 19.957 | 1 | 20 | 49 | 68 | GUGGAGCAUCAUCAAGAUUC | GAGUCUUCAUGCUGCUCCAU | Translation |
| ccp-miR172d-1-5p | Cc11_g11810 | 2.5 | 13.806 | 1 | 20 | 483 | 502 | GUGGAGCAUCAUCAAGAUUC | GAAUUUUGAUGAAGCUUUAC | Cleavage |
| ccp-miR172d-1-5p | Cc11_g15270 | 3.0 | 17.157 | 1 | 21 | 435 | 455 | GUGGAGCAUCAUCAAGAUUCA | UGUAUCUUGAUGGAGCUCCAU | Cleavage |
| ccp-miR172d-2-5p | Cc00_g01310 | 2.5 | 21.095 | 1 | 20 | 1156 | 1175 | UGCAGCAUCAUCAAGAUUCC | AGGAUCUUGUUGAUGCUGCA | Translation |
| ccp-miR172d-2-5p | Cc00_g05770 | 3.0 | 13.228 | 1 | 22 | 543 | 564 | UGCAGCAUCAUCAAGAUUCCCA | UGGGAAAUUUGAAGAUGUUGCA | Translation |
| ccp-miR172d-2-5p | Cc00_g09120 | 2.0 | 16.069 | 1 | 20 | 4584 | 4603 | UGCAGCAUCAUCAAGAUUCC | GGAGUCUUGGAGAUGCUGCA | Translation |
| ccp-miR172d-2-5p | Cc00_g10740 | 2.0 | 14.412 | 1 | 20 | 419 | 438 | UGCAGCAUCAUCAAGAUUCC | GGAAACUUGAAGAUGCUGCA | Translation |
| ccp-miR172d-2-5p | Cc00_g12340 | 3.0 | 19.466 | 1 | 22 | 540 | 561 | UGCAGCAUCAUCAAGAUUCCCA | UUGUAAUCCUGAUGAUGUUGUA | Cleavage |
| ccp-miR172d-2-5p | Cc00_g13080 | 3.0 | 15.184 | 1 | 22 | 1590 | 1611 | UGCAGCAUCAUCAAGAUUCCCA | UGGGAGUCUAAGUGAUGCUGCA | Cleavage |
| ccp-miR172d-2-5p | Cc00_g22160 | 2.5 | 15.38 | 1 | 20 | 1190 | 1209 | UGCAGCAUCAUCAAGAUUCC | GGAGACUUGAAGAUGCUGCA | Translation |
| ccp-miR172d-2-5p | Cc00_g25040 | 2.5 | 14.546 | 1 | 24 | 1327 | 1350 | UGCAGCAUCAUCAAGAUUCCCACA | UGGAGGAGUCUUAAGGAUGCUGCA | Translation |
| ccp-miR172d-2-5p | Cc00_g29760 | 3.0 | 15.35 | 1 | 24 | 794 | 817 | UGCAGCAUCAUCAAGAUUCCCACA | UGGAGGAAGCUUAAGGAUGCUGCA | Translation |
| ccp-miR172d-2-5p | Cc00_g35530 | 2.0 | 14.428 | 1 | 20 | 734 | 753 | UGCAGCAUCAUCAAGAUUCC | GGAAACUUGAAGAUGCUGCA | Translation |
| ccp-miR172d-2-5p | Cc01_g18680 | 2.5 | 18.713 | 1 | 20 | 2009 | 2028 | UGCAGCAUCAUCAAGAUUCC | AGGAUGUUGAUGAUGCUGCA | Cleavage |
| ccp-miR172d-2-5p | Cc02_g18720 | 3.0 | 15.765 | 1 | 20 | 1235 | 1255 | UGCAGCAUCA-UCAAGAUUCC | GGAGUCUUGGGUGAUGCUGCA | Translation |
| ccp-miR172d-2-5p | Cc02_g24150 | 3.0 | 18.35 | 1 | 20 | 575 | 594 | UGCAGCAUCAUCAAGAUUCC | GGGAUCUUGAUGCAGCUGCA | Cleavage |
| ccp-miR172d-2-5p | Cc04_g04080 | 2.5 | 16.543 | 1 | 24 | 301 | 324 | UGCAGCAUCAUCAAGAUUCCCACA | UCUAGAAAUCUUGAUGAUGCUCCA | Cleavage |
| ccp-miR172d-2-5p | Cc04_g04890 | 2.0 | 19.306 | 1 | 24 | 1215 | 1238 | UGCAGCAUCAUCAAGAUUCCCACA | UCUGGGAAUAUUGUUGAUGCUGCA | Translation |
| ccp-miR172d-2-5p | Cc04_g07810 | 2.0 | 13.494 | 1 | 20 | 558 | 577 | UGCAGCAUCAUCAAGAUUCC | GGAAUCUGGAUGAUGUUGUA | Cleavage |
| ccp-miR172d-2-5p | Cc04_g10350 | 3.0 | 17.065 | 1 | 20 | 608 | 627 | UGCAGCAUCAUCAAGAUUCC | GGAAUCUUCAUGAUGCUCUA | Cleavage |
| ccp-miR172d-2-5p | Cc04_g10640 | 3.0 | 12.799 | 1 | 23 | 680 | 702 | UGCAGCAUCAUCAAGAUUCCCAC | GAGGAAAUCUUGUUGAUGCUGCC | Translation |
| ccp-miR172d-2-5p | Cc04_g15220 | 3.0 | 16.587 | 1 | 20 | 2740 | 2759 | UGCAGCAUCAUCAAGAUUCC | AGAAUCUUAGUGAUGUUGCA | Cleavage |
| ccp-miR172d-2-5p | Cc06_g21860 | 2.0 | 15.237 | 1 | 20 | 1368 | 1387 | UGCAGCAUCAUCAAGAUUCC | GGAAACUUGAAGAUGCUGCA | Translation |
| ccp-miR172d-2-5p | Cc06_g21870 | 2.0 | 16.995 | 1 | 20 | 761 | 780 | UGCAGCAUCAUCAAGAUUCC | GGAAACUUGAAGAUGCUGCA | Translation |
| ccp-miR172d-2-5p | Cc06_g21940 | 2.5 | 17.335 | 1 | 24 | 1090 | 1113 | UGCAGCAUCAUCAAGAUUCCCACA | UGGAGGAGUCUUAAGGAUGCUGCA | Translation |
| ccp-miR172d-2-5p | Cc06_g21980 | 2.0 | 16.993 | 1 | 20 | 791 | 810 | UGCAGCAUCAUCAAGAUUCC | GGAAACUUGAAGAUGCUGCA | Translation |
| ccp-miR172d-2-5p | Cc06_g21990 | 2.0 | 12.698 | 1 | 20 | 708 | 727 | UGCAGCAUCAUCAAGAUUCC | GGAAACUUGAAGAUGCUGCA | Translation |
| ccp-miR172d-2-5p | Cc06_g22000 | 1.0 | 17.065 | 1 | 20 | 1488 | 1507 | UGCAGCAUCAUCAAGAUUCC | GGAAUCUUGAGGAUGCUGCA | Translation |
| ccp-miR172d-2-5p | Cc06_g22030 | 2.0 | 14.412 | 1 | 20 | 430 | 449 | UGCAGCAUCAUCAAGAUUCC | GGAAACUUGAAGAUGCUGCA | Translation |
| ccp-miR172d-2-5p | Cc07_g10580 | 3.0 | 23.885 | 1 | 23 | 989 | 1011 | UGCAGCAUCAUCAAGAUUCCCAC | GAGGCAAUGUGGAUGAUGCUGCA | Cleavage |
| ccp-miR172d-2-5p | Cc07_g19820 | 2.0 | 16.671 | 1 | 20 | 2096 | 2115 | UGCAGCAUCAUCAAGAUUCC | GGAAACUUGAAGAUGCUGCA | Translation |
| ccp-miR172d-2-5p | Cc07_g19830 | 2.0 | 16.671 | 1 | 20 | 525 | 544 | UGCAGCAUCAUCAAGAUUCC | GGAAACUUGAAGAUGCUGCA | Translation |
| ccp-miR172d-2-5p | Cc08_g09000 | 3.0 | 17.233 | 1 | 20 | 1968 | 1987 | UGCAGCAUCAUCAAGAUUCC | AGAAACUUGAUGAUGCUGUG | Cleavage |
| ccp-miR172d-2-5p | Cc11_g02140 | 2.0 | 14.798 | 1 | 20 | 782 | 801 | UGCAGCAUCAUCAAGAUUCC | GGAAACUUGAAGAUGCUGCA | Translation |
| ccp-miR172d-2-5p | Cc11_g02160 | 1.0 | 16.235 | 1 | 20 | 1181 | 1200 | UGCAGCAUCAUCAAGAUUCC | GGAAUCUUGAGGAUGCUGCA | Translation |
| ccp-miR172d-3-3p | Cc01_g04220 | 3.0 | 22.694 | 1 | 23 | 149 | 171 | UGAGAAUCUUGAUGAUGCUGCAU | AGGCGGCGACUUCAAGAUUCUCA | Cleavage |
| ccp-miR172d-3-3p | Cc01_g09090 | 3.0 | 8.115 | 1 | 20 | 325 | 344 | UGAGAAUCUUGAUGAUGCUG | CAUCAUCAUCACCAUUCUCA | Translation |
| ccp-miR172d-3-3p | Cc01_g11840 | 0.5 | 12.33 | 1 | 22 | 1877 | 1898 | UGAGAAUCUUGAUGAUGCUGCA | UGCAGCAUCAUCAGGAUUCUCA | Cleavage |
| ccp-miR172d-3-3p | Cc01_g21590 | 3.0 | 14.354 | 1 | 20 | 1764 | 1783 | UGAGAAUCUUGAUGAUGCUG | CAGGAUCAUUAAGAUUUUUG | Cleavage |
| ccp-miR172d-3-3p | Cc02_g15050 | 2.5 | 19.081 | 1 | 23 | 695 | 717 | UGAGAAUCUUGAUGAUGCUGCAU | AUACAGCAACUUCAAGGUUCUCA | Cleavage |
| ccp-miR172d-3-3p | Cc02_g22090 | 3.0 | 16.766 | 1 | 23 | 134 | 156 | UGAGAAUCUUGAUGAUGCUGCAU | AGGUAGCACUUUCAAGAUUCUCA | Cleavage |
| ccp-miR172d-3-3p | Cc02_g34000 | 2.5 | 19.998 | 1 | 23 | 8 | 30 | UGAGAAUCUUGAUGAUGCUGCAU | AUUCAGCAUCAUCAUGGUUCUCU | Translation |
| ccp-miR172d-3-3p | Cc04_g04580 | 3.0 | 18.454 | 1 | 23 | 5067 | 5089 | UGAGAAUCUUGAUGAUGCUGCAU | GUGCAGCAUUCUUCAGAUUCUCA | Translation |
| ccp-miR172d-3-3p | Cc07_g00550 | 2.5 | 7.217 | 1 | 20 | 38 | 57 | UGAGAAUCUUGAUGAUGCUG | CAUCAUCAUCAAGAUGCUCA | Cleavage |
| ccp-miR172d-3-3p | Cc07_g06200 | 2.0 | 14.317 | 1 | 22 | 1827 | 1848 | UGAGAAUCUUGAUGAUGCUGCA | UGCAGCAUCAUCAGGAUUCCCA | Cleavage |
| ccp-miR172d-3-3p | Cc07_g17000 | 3.0 | 11.305 | 1 | 20 | 200 | 220 | UGAGAAUCUUGAUG-AUGCUG | CAGCAUUCAUCAAGAUUUUUA | Cleavage |
| ccp-miR172d-3-3p | Cc08_g13820 | 3.0 | 24.848 | 1 | 23 | 643 | 665 | UGAGAAUCUUGAUGAUGCUGCAU | GUUCAGCUUUAUCAAGAUGCUCA | Cleavage |
| ccp-miR172d-3-3p | Cc09_g00710 | 3.0 | 16.071 | 1 | 20 | 1067 | 1086 | UGAGAAUCUUGAUGAUGCUG | CAGCAUCGCCAACAUUUUCA | Cleavage |
| ccp-miR172d-3-3p | Cc09_g02450 | 1.5 | 13.418 | 1 | 22 | 1527 | 1548 | UGAGAAUCUUGAUGAUGCUGCA | UGCAGCAUCAUCAGGAUUCUCC | Cleavage |
| ccp-miR172d-3-3p | Cc09_g02500 | 3.0 | 15.244 | 1 | 20 | 158 | 177 | UGAGAAUCUUGAUGAUGCUG | UGGCACCUUCAAGAUUCUCA | Cleavage |
| ccp-miR172d-3-3p | Cc09_g09350 | 2.5 | 9.955 | 1 | 23 | 198 | 220 | UGAGAAUCUUGAUGAUGCUGCAU | AUCAAACAUCAUCAGGAUUCUCA | Cleavage |
| ccp-miR172d-3-3p | Cc10_g13500 | 3.0 | 17.152 | 1 | 20 | 1017 | 1036 | UGAGAAUCUUGAUGAUGCUG | CAGUGUUCUCAAGAUUCUUA | Cleavage |
| ccp-miR172d-3-3p | Cc10_g14800 | 2.5 | 18.314 | 1 | 20 | 2038 | 2057 | UGAGAAUCUUGAUGAUGCUG | CAGCAUCAGCAGAAUUCUCA | Cleavage |
| ccp-miR172d-3-3p | Cc10_g16030 | 3.0 | 17.152 | 1 | 20 | 1738 | 1757 | UGAGAAUCUUGAUGAUGCUG | CAGUGUUCUCAAGAUUCUUA | Cleavage |
| ccp-miR172d-3-3p | Cc11_g13310 | 3.0 | 9.325 | 1 | 20 | 427 | 446 | UGAGAAUCUUGAUGAUGCUG | CAAGAUCAUCAUGAUUCUCA | Translation |
| ccp-miR172d-3-5p | Cc00_g00430 | 3.0 | 16.833 | 1 | 24 | 470 | 493 | GUGUAGCAUCAUCAAGAUUCACAU | AUUUGAGUCUUAAGGAUGCUGCAC | Translation |
| ccp-miR172d-3-5p | Cc00_g01310 | 2.5 | 19.791 | 1 | 20 | 1157 | 1176 | GUGUAGCAUCAUCAAGAUUC | GGAUCUUGUUGAUGCUGCAU | Cleavage |
| ccp-miR172d-3-5p | Cc00_g18670 | 3.0 | 18.892 | 1 | 24 | 975 | 998 | GUGUAGCAUCAUCAAGAUUCACAU | AUUUGAGUCUUAAGGAUGCUGCAC | Translation |
| ccp-miR172d-3-5p | Cc01_g18680 | 3.0 | 18.902 | 1 | 20 | 2010 | 2029 | GUGUAGCAUCAUCAAGAUUC | GGAUGUUGAUGAUGCUGCAA | Cleavage |
| ccp-miR172d-3-5p | Cc01_g21720 | 3.0 | 19.384 | 1 | 21 | 1094 | 1114 | GUGUAGCAUCAUCAAGAUUCA | UGUAUCUUGAUAAUGCUAUGC | Translation |
| ccp-miR172d-3-5p | Cc02_g21560 | 2.5 | 15.257 | 1 | 22 | 1388 | 1409 | GUGUAGCAUCAUCAAGAUUCAC | GAGAAUCAUGAUGAUACUACAC | Cleavage |
| ccp-miR172d-3-5p | Cc02_g35800 | 2.5 | 18.229 | 1 | 21 | 1365 | 1384 | GUGUAGCAUCAUCAAGAUUCA | UGAA-CUUGAUGAUGCUAUAC | Cleavage |
| ccp-miR172d-3-5p | Cc04_g04890 | 3.0 | 19.688 | 1 | 23 | 1217 | 1239 | GUGUAGCAUCAUCAAGAUUCACA | UGGGAAUAUUGUUGAUGCUGCAU | Cleavage |
| ccp-miR172d-3-5p | Cc04_g15220 | 3.0 | 17.452 | 1 | 24 | 2736 | 2760 | GUGUAGCAUCAUCAAGAUUC-ACAU | AUGUAGAAUCUUAGUGAUGUUGCAU | Cleavage |
| ccp-miR172d-3-5p | Cc06_g18090 | 3.0 | 14.496 | 1 | 20 | 843 | 862 | GUGUAGCAUCAUCAAGAUUC | GGAUUUUGGUGAUGCAACAC | Cleavage |
| ccp-miR172d-3-5p | Cc06_g22000 | 2.5 | 16.66 | 1 | 20 | 1489 | 1508 | GUGUAGCAUCAUCAAGAUUC | GAAUCUUGAGGAUGCUGCAA | Translation |
| ccp-miR172d-3-5p | Cc07_g13260 | 3.0 | 15.838 | 1 | 24 | 350 | 373 | GUGUAGCAUCAUCAAGAUUCACAU | AUUUGAAGCCUGAUGAUGCUACGU | Cleavage |
| ccp-miR172d-3-5p | Cc08_g01550 | 3.0 | 16.829 | 1 | 22 | 1034 | 1055 | GUGUAGCAUCAUCAAGAUUCAC | GUGAAUCUGGAUGAUGUGACAC | Cleavage |
| ccp-miR172d-3-5p | Cc11_g02160 | 2.5 | 16.324 | 1 | 20 | 1182 | 1201 | GUGUAGCAUCAUCAAGAUUC | GAAUCUUGAGGAUGCUGCAA | Translation |
| ccp-miR174e-3p | Cc00_g35320 | 3.0 | 19.297 | 1 | 21 | 918 | 938 | UUUAUGGAACAGAGGGAGUAG | CUACUACUUCCGUUCCAUGAA | Translation |
| ccp-miR174e-3p | Cc01_g13460 | 3.0 | 13.79 | 1 | 20 | 2458 | 2477 | UUUAUGGAACAGAGGGAGUA | UAUUUCCUGCGUUCCAUAAA | Translation |
| ccp-miR174e-3p | Cc04_g11540 | 3.0 | 18.809 | 1 | 20 | 2003 | 2022 | UUUAUGGAACAGAGGGAGUA | CACUUUCUCUGUUCCAUGAG | Cleavage |
| ccp-miR174e-3p | Cc08_g03550 | 3.0 | 16.23 | 1 | 20 | 2107 | 2126 | UUUAUGGAACAGAGGGAGUA | UACUUCUUUUUUUCCAUAAG | Translation |
| ccp-miR174e-3p | Cc09_g10710 | 3.0 | 19.297 | 1 | 21 | 1254 | 1274 | UUUAUGGAACAGAGGGAGUAG | CUACUACUUCCGUUCCAUGAA | Translation |
| ccp-miR174e-3p | Cc10_g02590 | 3.0 | 9.743 | 1 | 21 | 2469 | 2489 | UUUAUGGAACAGAGGGAGUAG | UUGGUCUCUCUGUUUCAUGAA | Cleavage |
| ccp-miR174e-3p | Cc10_g16090 | 3.0 | 15.058 | 1 | 21 | 49 | 69 | UUUAUGGAACAGAGGGAGUAG | UUGCUUCCUUGGUUCCGUAAA | Translation |
| ccp-miR1863b-1-3p | Cc00_g02300 | 1.5 | 15.116 | 1 | 20 | 1183 | 1202 | AGCUCUGAUACCAUGUUAAC | GUUGACAUGGGAUCAGAGCU | Translation |
| ccp-miR1863b-1-3p | Cc02_g35500 | 3.0 | 14.82 | 1 | 20 | 253 | 271 | AGCUCUGAUACCAUGUUAAC | UUUAACAUG-UAUCAGAGCU | Translation |
| ccp-miR1863b-1-3p | Cc02_g35510 | 3.0 | 14.82 | 1 | 20 | 253 | 271 | AGCUCUGAUACCAUGUUAAC | UUUAACAUG-UAUCAGAGCU | Translation |
| ccp-miR1863b-1-3p | Cc07_g08250 | 3.0 | 20.845 | 1 | 21 | 958 | 978 | AGCUCUGAUACCAUGUUAACU | GGUUGGCCUGGUACCAGAGCU | Cleavage |
| ccp-miR1863b-2-5p | Cc02_g13570 | 3.0 | 12.925 | 1 | 24 | 694 | 717 | AGCUCUGAUACCAACUUAUCCGUU | AAGGGACAAGUUGGUGUUGGAGUU | Cleavage |
| ccp-miR1863b-2-5p | Cc02_g39830 | 3.0 | 13.052 | 1 | 24 | 846 | 869 | AGCUCUGAUACCAACUUAUCCGUU | GGUGGAUGAGUUGGAAUCACAGCU | Translation |
| ccp-miR1863b-2-5p | Cc04_g03210 | 3.0 | 14.721 | 1 | 24 | 2273 | 2296 | AGCUCUGAUACCAACUUAUCCGUU | GGUAGAUAAUUUGUUAUUAGAGUU | Translation |
| ccp-miR1878-3p | Cc00_g01590 | 3.0 | 12.445 | 1 | 20 | 1660 | 1679 | AUUUGUAGUGUUCAAAUAGA | UCAAUUUGAAUAAUACAAGU | Cleavage |
| ccp-miR1878-3p | Cc00_g13900 | 1.5 | 18.936 | 1 | 24 | 1318 | 1341 | AUUUGUAGUGUUCAAAUAGAGCUU | GGGAUCUGUUUGAACAUUACAGAU | Cleavage |
| ccp-miR1878-3p | Cc00_g20530 | 3.0 | 14.484 | 1 | 24 | 16 | 39 | AUUUGUAGUGUUCAAAUAGAGCUU | AAUCUCUAUUUGAACAAUAUGGGU | Cleavage |
| ccp-miR1878-3p | Cc03_g01500 | 3.0 | 18.79 | 1 | 22 | 1693 | 1714 | AUUUGUAGUGUUCAAAUAGAGC | GUUCUAUUUGGGCACCACAGAU | Cleavage |
| ccp-miR1878-3p | Cc04_g14930 | 3.0 | 13.904 | 1 | 20 | 610 | 629 | AUUUGUAGUGUUCAAAUAGA | UUUGAUUGAUCACUACAAAU | Translation |
| ccp-miR1878-3p | Cc05_g05230 | 3.0 | 16.536 | 1 | 21 | 1009 | 1029 | AUUUGUAGUGUUCAAAUAGAG | CUUUCUCUGAACGCUACAAAU | Cleavage |
| ccp-miR1878-3p | Cc06_g15730 | 2.5 | 18.156 | 1 | 21 | 1368 | 1388 | AUUUGUAGUGUUCAAAUAGAG | UUGUAUUUGAAGACUACAAGU | Translation |
| ccp-miR1878-3p | Cc11_g02780 | 3.0 | 22.028 | 1 | 21 | 59 | 79 | AUUUGUAGUGUUCAAAUAGAG | UUCUAUUCAGACACUGCAAAU | Cleavage |
| ccp-miR1885a-3p | Cc00_g15780 | 3.0 | 13.7 | 1 | 20 | 309 | 328 | CAUCAAUGAAAAGUAUUAUU | AAUUCUUCUUUUCAUUGAUG | Cleavage |
| ccp-miR1885a-3p | Cc01_g08910 | 3.0 | 17.419 | 1 | 22 | 826 | 847 | CAUCAAUGAAAAGUAUUAUUGU | AAAAUAAUAUUUUGCAUUUAUG | Translation |
| ccp-miR1885a-3p | Cc02_g00660 | 3.0 | 15.805 | 1 | 20 | 1644 | 1663 | CAUCAAUGAAAAGUAUUAUU | AAUAAUUUUCUUUAUUGAUG | Translation |
| ccp-miR1885a-3p | Cc02_g25320 | 2.0 | 14.094 | 1 | 22 | 31 | 52 | CAUCAAUGAAAAGUAUUAUUGU | AGGAUAAUACUAUUUAUUGAUG | Translation |
| ccp-miR1885a-3p | Cc02_g37300 | 3.0 | 15.655 | 1 | 20 | 1860 | 1879 | CAUCAAUGAAAAGUAUUAUU | CAUGCUACUUUUCGUUGAUG | Cleavage |
| ccp-miR1885a-3p | Cc04_g05910 | 1.0 | 10.527 | 1 | 20 | 1295 | 1314 | CAUCAAUGAAAAGUAUUAUU | AAUCAUACUUUUCAUUGAUG | Cleavage |
| ccp-miR1885a-3p | Cc07_g04080 | 3.0 | 14.346 | 1 | 20 | 1261 | 1280 | CAUCAAUGAAAAGUAUUAUU | GAUUAUCCUUUUUAUUGAUG | Cleavage |
| ccp-miR1885a-3p | Cc08_g00050 | 2.5 | 15.493 | 1 | 20 | 1279 | 1298 | CAUCAAUGAAAAGUAUUAUU | GAUAAUAUUAUUUAUUGAUG | Translation |
| ccp-miR1885a-3p | Cc08_g14650 | 2.5 | 17.316 | 1 | 20 | 347 | 366 | CAUCAAUGAAAAGUAUUAUU | AGUGAAGCUUUUCAUUGAUG | Cleavage |
| ccp-miR1919-5p | Cc04_g03600 | 2.0 | 19.879 | 1 | 21 | 1687 | 1707 | UGUCGCAGGUGACUUUCGCCU | AGGUGAAAGUUACUUGCGGCA | Cleavage |
| ccp-miR1919-5p | Cc11_g01570 | 2.0 | 21.16 | 1 | 21 | 263 | 283 | UGUCGCAGGUGACUUUCGCCU | AUGCGAAAGUCUCCUGCGACA | Translation |
| ccp-miR1919-5p | Cc11_g01580 | 1.5 | 22.798 | 1 | 21 | 575 | 595 | UGUCGCAGGUGACUUUCGCCU | AUGCGAAAGUCAUCUGCGACA | Cleavage |
| ccp-miR1919-5p | Cc11_g01590 | 2.5 | 18.31 | 1 | 20 | 240 | 259 | UGUCGCAGGUGACUUUCGCC | UGCGAAAAUCAUCUGCGACA | Cleavage |
| ccp-miR2111-3p | Cc05_g10460 | 3.0 | 21.43 | 1 | 20 | 358 | 377 | UCCUCAGGAUACAGAUUACC | GGUGAUCUGUUUCUUGAGGU | Translation |
| ccp-miR2111-3p | Cc06_g07830 | 3.0 | 18.608 | 1 | 21 | 2215 | 2235 | UCCUCAGGAUACAGAUUACCU | GGGUUAUUUGGAUCCUGAGGG | Translation |
| ccp-miR2111-3p | Cc10_g07730 | 3.0 | 17.071 | 1 | 20 | 1670 | 1689 | UCCUCAGGAUACAGAUUACC | GGUAAUCUGAGUCCUGAGAA | Translation |
| ccp-miR2111-5p | Cc07_g00750 | 0.5 | 19.674 | 1 | 20 | 1387 | 1406 | UAAUCUGCAUCCUGAGGUUU | GAACCUCAGGAUGCAGAUUA | Cleavage |
| ccp-miR2111-5p | Cc07_g14070 | 3.0 | 13.19 | 1 | 20 | 1965 | 1984 | UAAUCUGCAUCCUGAGGUUU | AAAACUCAGGAUGGAGAUUG | Cleavage |
| ccp-miR2275d-1-5p | Cc00_g23290 | 3.0 | 18.751 | 1 | 20 | 312 | 331 | AGAGUUGGAGAAAAGAUAAC | GUUUUCUUCUCUUCAAUUCU | Cleavage |
| ccp-miR2275d-1-5p | Cc00_g32160 | 2.0 | 8.172 | 1 | 20 | 665 | 684 | AGAGUUGGAGAAAAGAUAAC | UUUAUCUUUUUUCCAACUUU | Cleavage |
| ccp-miR2275d-1-5p | Cc02_g32120 | 2.5 | 16.734 | 1 | 21 | 63 | 83 | AGAGUUGGAGAAAAGAUAACC | GGUUUUUUUUUCUCUAACUUU | Cleavage |
| ccp-miR2275d-1-5p | Cc02_g33850 | 2.5 | 6.26 | 1 | 20 | 565 | 584 | AGAGUUGGAGAAAAGAUAAC | GUUUUUUUUUCUCCAACUCG | Cleavage |
| ccp-miR2275d-1-5p | Cc03_g04230 | 3.0 | 8.784 | 1 | 21 | 258 | 278 | AGAGUUGGAGAAAAGAUAACC | GGCUAUCUUUUUCUCAACUCU | Translation |
| ccp-miR2275d-1-5p | Cc03_g04420 | 3.0 | 9.224 | 1 | 21 | 1288 | 1308 | AGAGUUGGAGAAAAGAUAACC | GGCUAUCUUCUCUACAACUCU | Cleavage |
| ccp-miR2275d-1-5p | Cc05_g16080 | 3.0 | 2.439 | 1 | 20 | 210 | 229 | AGAGUUGGAGAAAAGAUAAC | GCUACCUUUUCUGCAACUCU | Cleavage |
| ccp-miR2275d-1-5p | Cc06_g15820 | 2.5 | 22.159 | 1 | 20 | 45 | 64 | AGAGUUGGAGAAAAGAUAAC | GUUUUCUUUACUCCAGCUCU | Translation |
| ccp-miR2275d-1-5p | Cc07_g05850 | 3.0 | 7.18 | 1 | 21 | 148 | 168 | AGAGUUGGAGAAAAGAUAACC | GGUUCUUGUUUCUUCAACUCU | Cleavage |
| ccp-miR2275d-1-5p | Cc11_g13930 | 3.0 | 6.218 | 1 | 21 | 231 | 251 | AGAGUUGGAGAAAAGAUAACC | GGUUAUCAUUCCUCUAAUUCU | Translation |
| ccp-miR2275d-2-3p | Cc00_g10290 | 3.0 | 17.098 | 1 | 22 | 1932 | 1953 | CUUGUUUUUCUCCUUUAUCUCU | AGUGAUUAAGGGGAAAAACAGG | Cleavage |
| ccp-miR2275d-2-3p | Cc00_g10950 | 3.0 | 6.036 | 1 | 22 | 589 | 610 | CUUGUUUUUCUCCUUUAUCUCU | GGAGGAAGAGGAGGAAGACAAG | Cleavage |
| ccp-miR2275d-2-3p | Cc00_g12250 | 2.5 | 15.766 | 1 | 21 | 1300 | 1320 | CUUGUUUUUCUCCUUUAUCUC | GAGAUGAAGGAGAGAAACAUG | Cleavage |
| ccp-miR2275d-2-3p | Cc00_g16960 | 2.5 | 14.807 | 1 | 22 | 3859 | 3880 | CUUGUUUUUCUCCUUUAUCUCU | AUAGAGAAAGUAGAAGAACAAG | Cleavage |
| ccp-miR2275d-2-3p | Cc00_g20120 | 2.5 | 10.807 | 1 | 20 | 1034 | 1053 | CUUGUUUUUCUCCUUUAUCU | AGAAAAAAGAGAAAAACGAG | Cleavage |
| ccp-miR2275d-2-3p | Cc01_g04330 | 2.0 | 12.405 | 1 | 20 | 11 | 30 | CUUGUUUUUCUCCUUUAUCU | AGAAAAGGGGGAAAAACAAG | Cleavage |
| ccp-miR2275d-2-3p | Cc01_g08150 | 3.0 | 5.455 | 1 | 20 | 452 | 471 | CUUGUUUUUCUCCUUUAUCU | AGAAAAAGGAGAAAAAGAGG | Cleavage |
| ccp-miR2275d-2-3p | Cc01_g20100 | 3.0 | 14.689 | 1 | 20 | 3706 | 3725 | CUUGUUUUUCUCCUUUAUCU | AGGUGAAGAAGAGAAGCAAG | Cleavage |
| ccp-miR2275d-2-3p | Cc02_g02350 | 3.0 | 14.624 | 1 | 20 | 1753 | 1772 | CUUGUUUUUCUCCUUUAUCU | AGAAAAAGGAGGAAAAGAAG | Cleavage |
| ccp-miR2275d-2-3p | Cc02_g04510 | 3.0 | 14.115 | 1 | 22 | 343 | 364 | CUUGUUUUUCUCCUUUAUCUCU | GGAGAUCAAAGUGAAAAACAAG | Translation |
| ccp-miR2275d-2-3p | Cc02_g06660 | 3.0 | 14.119 | 1 | 20 | 553 | 572 | CUUGUUUUUCUCCUUUAUCU | UGAUGAAGGAGAAAGAUGAG | Cleavage |
| ccp-miR2275d-2-3p | Cc02_g17350 | 3.0 | 18.207 | 1 | 21 | 1721 | 1741 | CUUGUUUUUCUCCUUUAUCUC | GAGUUAAAGGAGAAGAACCAG | Cleavage |
| ccp-miR2275d-2-3p | Cc02_g31610 | 3.0 | 17.991 | 1 | 22 | 947 | 968 | CUUGUUUUUCUCCUUUAUCUCU | AUGGAUGAAGGGGAAAAACGAU | Cleavage |
| ccp-miR2275d-2-3p | Cc02_g38000 | 1.5 | 17.494 | 1 | 20 | 89 | 108 | CUUGUUUUUCUCCUUUAUCU | AGAUAAAGGGGGAAGACAAG | Cleavage |
| ccp-miR2275d-2-3p | Cc02_g39800 | 3.0 | 20.77 | 1 | 20 | 213 | 232 | CUUGUUUUUCUCCUUUAUCU | AGGAGAAGGAGAAGAGCAAG | Cleavage |
| ccp-miR2275d-2-3p | Cc03_g01830 | 3.0 | 15.433 | 1 | 20 | 447 | 466 | CUUGUUUUUCUCCUUUAUCU | GGAUGAAGGAGCAGAGCAAG | Translation |
| ccp-miR2275d-2-3p | Cc03_g02480 | 3.0 | 15.373 | 1 | 20 | 1812 | 1831 | CUUGUUUUUCUCCUUUAUCU | GGAUAGAGGUGAAAAAUGAG | Translation |
| ccp-miR2275d-2-3p | Cc03_g03020 | 2.5 | 17.238 | 1 | 22 | 95 | 116 | CUUGUUUUUCUCCUUUAUCUCU | AGGGAUAGGGGAGAAGAGCAAG | Cleavage |
| ccp-miR2275d-2-3p | Cc03_g08250 | 3.0 | 18.975 | 1 | 22 | 1137 | 1158 | CUUGUUUUUCUCCUUUAUCUCU | AGAGAUAGAGGAGAAGAAUCAG | Cleavage |
| ccp-miR2275d-2-3p | Cc03_g08440 | 3.0 | 3.27 | 1 | 22 | 120 | 141 | CUUGUUUUUCUCCUUUAUCUCU | GGAUAAAAAAGAGAAAAACAAG | Cleavage |
| ccp-miR2275d-2-3p | Cc03_g14480 | 2.5 | 19.165 | 1 | 22 | 5142 | 5163 | CUUGUUUUUCUCCUUUAUCUCU | AAAGCUAGAGGAGGAAAACAGG | Cleavage |
| ccp-miR2275d-2-3p | Cc04_g04270 | 2.0 | 11.501 | 1 | 22 | 646 | 667 | CUUGUUUUUCUCCUUUAUCUCU | AAAGAAAGGGGAGAAAAACAAG | Cleavage |
| ccp-miR2275d-2-3p | Cc05_g06380 | 2.0 | 13.245 | 1 | 22 | 160 | 181 | CUUGUUUUUCUCCUUUAUCUCU | ACAGAUAAAGGAAGAAAACGAG | Translation |
| ccp-miR2275d-2-3p | Cc06_g16880 | 2.0 | 14.487 | 1 | 20 | 621 | 640 | CUUGUUUUUCUCCUUUAUCU | AGAAAAAGGGGAAAAAUAAG | Cleavage |
| ccp-miR2275d-2-3p | Cc07_g05180 | 3.0 | 19.775 | 1 | 21 | 100 | 120 | CUUGUUUUUCUCCUUUAUCUC | GAGAUAUAGGAGAAAAGCAUG | Cleavage |
| ccp-miR2275d-2-3p | Cc07_g11120 | 3.0 | 18.997 | 1 | 20 | 302 | 321 | CUUGUUUUUCUCCUUUAUCU | AGUUGAAGGAGAAAGGCGAG | Cleavage |
| ccp-miR2275d-2-3p | Cc07_g14870 | 2.5 | 19.279 | 1 | 20 | 1540 | 1559 | CUUGUUUUUCUCCUUUAUCU | GGACAAGGGAGAAAGACAAG | Cleavage |
| ccp-miR2275d-2-3p | Cc08_g03820 | 3.0 | 13.538 | 1 | 22 | 702 | 723 | CUUGUUUUUCUCCUUUAUCUCU | GGAGGGAAAGGAGGAAAAUGAG | Cleavage |
| ccp-miR2275d-2-3p | Cc09_g03320 | 3.0 | 19.457 | 1 | 20 | 426 | 445 | CUUGUUUUUCUCCUUUAUCU | GGAUAAAGCAGAAAAAGAAG | Cleavage |
| ccp-miR2275d-2-3p | Cc09_g08570 | 3.0 | 8.912 | 1 | 20 | 719 | 738 | CUUGUUUUUCUCCUUUAUCU | AGAUAAAGAAGAAAAGGAAG | Cleavage |
| ccp-miR2275d-2-3p | Cc09_g08590 | 3.0 | 14.029 | 1 | 20 | 1208 | 1227 | CUUGUUUUUCUCCUUUAUCU | AUAUGAGGGAGGAAAGCAAG | Cleavage |
| ccp-miR2275d-2-3p | Cc09_g08870 | 2.5 | 8.564 | 1 | 20 | 77 | 96 | CUUGUUUUUCUCCUUUAUCU | AGAUUGAGGAGAAAAACAAA | Cleavage |
| ccp-miR2275d-2-3p | Cc09_g10350 | 3.0 | 4.965 | 1 | 22 | 1212 | 1233 | CUUGUUUUUCUCCUUUAUCUCU | AAAGAGAAAGGAGAAGAACCAG | Cleavage |
| ccp-miR2275d-2-3p | Cc09_g10350 | 3.0 | 4.547 | 1 | 22 | 1241 | 1262 | CUUGUUUUUCUCCUUUAUCUCU | ACGGAAAGGGGAGAAGAACAAG | Cleavage |
| ccp-miR2275d-2-3p | Cc10_g01950 | 2.5 | 16.199 | 1 | 22 | 579 | 600 | CUUGUUUUUCUCCUUUAUCUCU | AUGGACAGAGGAGAAAAACGAG | Cleavage |
| ccp-miR2275d-2-3p | Cc11_g11690 | 3.0 | 18.041 | 1 | 21 | 2763 | 2783 | CUUGUUUUUCUCCUUUAUCUC | GAGGUGAAGGAAAAGGACAAG | Translation |
| ccp-miR2275d-2-3p | Cc11_g11710 | 2.0 | 8.572 | 1 | 20 | 636 | 655 | CUUGUUUUUCUCCUUUAUCU | AGAUAAAGGAGAAAGAGAAG | Cleavage |
| ccp-miR2275d-2-3p | Cc11_g17250 | 2.5 | 7.506 | 1 | 20 | 716 | 735 | CUUGUUUUUCUCCUUUAUCU | AGAGGAAGGAGACAAACAAG | Cleavage |
| ccp-miR2592-1-5p | Cc02_g11410 | 3.0 | 13.761 | 1 | 22 | 845 | 866 | GAGUAAUUCGAACUUGAUAAGG | UUUUAUCAAGAUUAAAUUAUUC | Translation |
| ccp-miR2592-1-5p | Cc05_g11230 | 3.0 | 19.324 | 1 | 20 | 331 | 350 | GAGUAAUUCGAACUUGAUAA | UUUUCAAGUUGGAAUUAUUU | Translation |
| ccp-miR2592-1-5p | Cc07_g05790 | 3.0 | 21.214 | 1 | 22 | 1451 | 1472 | GAGUAAUUCGAACUUGAUAAGG | UUUUGUGGAGUUCGAGUUAUUC | Cleavage |
| ccp-miR2592-1-5p | Cc10_g05880 | 3.0 | 20.78 | 1 | 22 | 1160 | 1181 | GAGUAAUUCGAACUUGAUAAGG | CCUUACGAAGUUCAAAUUACUC | Translation |
| ccp-miR2592-2-5p | Cc04_g03520 | 2.0 | 18.966 | 1 | 20 | 427 | 446 | GACUAGGACCAAAGUAUUUC | GAAAUAUUUUGGUCUUGGUU | Cleavage |
| ccp-miR2592-2-5p | Cc06_g17030 | 3.0 | 20.679 | 1 | 20 | 2079 | 2098 | GACUAGGACCAAAGUAUUUC | GAAGUAUUUUGAUCUUAGUU | Translation |
| ccp-miR2592s-1-3p | Cc00_g10740 | 2.5 | 14.778 | 1 | 21 | 520 | 540 | AAAUGCUUGAUUAAUGUUGUU | AGGAAGAUUAAUCAAGCAUUU | Cleavage |
| ccp-miR2592s-1-3p | Cc01_g07160 | 3.0 | 13.589 | 1 | 21 | 1139 | 1158 | AAAUGCUUGAUUAAUGUUGUU | AACAAC-UUAGUCAAGUAUUU | Cleavage |
| ccp-miR2592s-1-3p | Cc01_g18910 | 2.5 | 13.939 | 1 | 21 | 209 | 229 | AAAUGCUUGAUUAAUGUUGUU | GAUGAUAUUCAUCAAGCAUUU | Cleavage |
| ccp-miR2592s-1-3p | Cc02_g25940 | 2.5 | 16.967 | 1 | 20 | 354 | 373 | AAAUGCUUGAUUAAUGUUGU | AUGAAAUUGAUCAAGCAUUU | Cleavage |
| ccp-miR2592s-1-3p | Cc04_g03400 | 3.0 | 9.113 | 1 | 21 | 31 | 51 | AAAUGCUUGAUUAAUGUUGUU | GAAACUAUUAGUCAAGCAUUU | Cleavage |
| ccp-miR2592s-1-3p | Cc04_g08960 | 2.5 | 15.099 | 1 | 20 | 811 | 830 | AAAUGCUUGAUUAAUGUUGU | ACAACAUUGAGCAGGUAUUU | Translation |
| ccp-miR2592s-1-3p | Cc06_g21180 | 3.0 | 11.332 | 1 | 20 | 484 | 503 | AAAUGCUUGAUUAAUGUUGU | ACUACAUUCAUUGAGCAUUU | Cleavage |
| ccp-miR2592s-1-3p | Cc06_g21870 | 3.0 | 17.669 | 1 | 21 | 862 | 882 | AAAUGCUUGAUUAAUGUUGUU | AGGAAGGUUAAUCAAGCAUUU | Cleavage |
| ccp-miR2592s-1-3p | Cc06_g21980 | 3.0 | 17.668 | 1 | 21 | 892 | 912 | AAAUGCUUGAUUAAUGUUGUU | AGGAAGGUUAAUCAAGCAUUU | Cleavage |
| ccp-miR2592s-1-3p | Cc06_g22030 | 2.5 | 14.778 | 1 | 21 | 531 | 551 | AAAUGCUUGAUUAAUGUUGUU | AGGAAGAUUAAUCAAGCAUUU | Cleavage |
| ccp-miR2592s-1-3p | Cc09_g02360 | 3.0 | 9.267 | 1 | 19 | 648 | 667 | AAAUGCUUGAUU-AAUGUUG | AAACAUUUAAUCAAGCAUUU | Cleavage |
| ccp-miR2592s-1-3p | Cc09_g06120 | 3.0 | 14.903 | 1 | 21 | 862 | 882 | AAAUGCUUGAUUAAUGUUGUU | GGCAGUAUUAGGCAAGCAUUU | Translation |
| ccp-miR2592s-1-3p | Cc11_g02140 | 3.0 | 17.91 | 1 | 21 | 883 | 903 | AAAUGCUUGAUUAAUGUUGUU | AGGAAGGUUAAUCAAGCAUUU | Cleavage |
| ccp-miR2592s-1-3p | Cc11_g06410 | 2.0 | 11.32 | 1 | 21 | 2104 | 2124 | AAAUGCUUGAUUAAUGUUGUU | AAGAACAUUAAGCAAGCAUUU | Translation |
| ccp-miR2592s-2-3p | Cc00_g10740 | 2.5 | 14.778 | 1 | 21 | 520 | 540 | AAAUGCUUGAUUAAUGUUGUU | AGGAAGAUUAAUCAAGCAUUU | Cleavage |
| ccp-miR2592s-2-3p | Cc01_g07160 | 3.0 | 13.589 | 1 | 21 | 1139 | 1158 | AAAUGCUUGAUUAAUGUUGUU | AACAAC-UUAGUCAAGUAUUU | Cleavage |
| ccp-miR2592s-2-3p | Cc01_g18910 | 2.5 | 13.939 | 1 | 21 | 209 | 229 | AAAUGCUUGAUUAAUGUUGUU | GAUGAUAUUCAUCAAGCAUUU | Cleavage |
| ccp-miR2592s-2-3p | Cc02_g25940 | 2.5 | 16.967 | 1 | 20 | 354 | 373 | AAAUGCUUGAUUAAUGUUGU | AUGAAAUUGAUCAAGCAUUU | Cleavage |
| ccp-miR2592s-2-3p | Cc04_g03400 | 3.0 | 9.113 | 1 | 21 | 31 | 51 | AAAUGCUUGAUUAAUGUUGUU | GAAACUAUUAGUCAAGCAUUU | Cleavage |
| ccp-miR2592s-2-3p | Cc04_g08960 | 2.5 | 15.099 | 1 | 20 | 811 | 830 | AAAUGCUUGAUUAAUGUUGU | ACAACAUUGAGCAGGUAUUU | Translation |
| ccp-miR2592s-2-3p | Cc06_g21180 | 3.0 | 11.332 | 1 | 20 | 484 | 503 | AAAUGCUUGAUUAAUGUUGU | ACUACAUUCAUUGAGCAUUU | Cleavage |
| ccp-miR2592s-2-3p | Cc06_g21870 | 3.0 | 17.669 | 1 | 21 | 862 | 882 | AAAUGCUUGAUUAAUGUUGUU | AGGAAGGUUAAUCAAGCAUUU | Cleavage |
| ccp-miR2592s-2-3p | Cc06_g21980 | 3.0 | 17.668 | 1 | 21 | 892 | 912 | AAAUGCUUGAUUAAUGUUGUU | AGGAAGGUUAAUCAAGCAUUU | Cleavage |
| ccp-miR2592s-2-3p | Cc06_g22030 | 2.5 | 14.778 | 1 | 21 | 531 | 551 | AAAUGCUUGAUUAAUGUUGUU | AGGAAGAUUAAUCAAGCAUUU | Cleavage |
| ccp-miR2592s-2-3p | Cc09_g02360 | 3.0 | 9.267 | 1 | 19 | 648 | 667 | AAAUGCUUGAUU-AAUGUUG | AAACAUUUAAUCAAGCAUUU | Cleavage |
| ccp-miR2592s-2-3p | Cc09_g06120 | 3.0 | 14.903 | 1 | 21 | 862 | 882 | AAAUGCUUGAUUAAUGUUGUU | GGCAGUAUUAGGCAAGCAUUU | Translation |
| ccp-miR2592s-2-3p | Cc11_g02140 | 3.0 | 17.91 | 1 | 21 | 883 | 903 | AAAUGCUUGAUUAAUGUUGUU | AGGAAGGUUAAUCAAGCAUUU | Cleavage |
| ccp-miR2592s-2-3p | Cc11_g06410 | 2.0 | 11.32 | 1 | 21 | 2104 | 2124 | AAAUGCUUGAUUAAUGUUGUU | AAGAACAUUAAGCAAGCAUUU | Translation |
| ccp-miR2612-5p | Cc07_g03680 | 3.0 | 19.351 | 1 | 20 | 1097 | 1116 | UGAUAGUGUCAACUAUUACC | GCUAAUAGUUGACCCUAUUA | Cleavage |
| ccp-miR2642-3p | Cc01_g07700 | 3.0 | 19.463 | 1 | 20 | 499 | 518 | AUGAGUUUCUUCAAAUCAUU | GCAGAUUUGAAGAAACUCAU | Cleavage |
| ccp-miR2642-3p | Cc02_g04050 | 3.0 | 19.73 | 1 | 21 | 1019 | 1039 | AUGAGUUUCUUCAAAUCAUUU | GGAUGAGUUGAAGAAAUUUGU | Cleavage |
| ccp-miR2642-3p | Cc02_g04480 | 3.0 | 19.124 | 1 | 21 | 1396 | 1416 | AUGAGUUUCUUCAAAUCAUUU | GGAUGAUUUGAGAAAGCUCGU | Translation |
| ccp-miR2642-3p | Cc02_g06450 | 2.5 | 14.546 | 1 | 20 | 3071 | 3090 | AUGAGUUUCUUCAAAUCAUU | AAGGAUUUGAAGAAGCUCAG | Cleavage |
| ccp-miR2642-3p | Cc03_g11620 | 3.0 | 17.007 | 1 | 21 | 1831 | 1851 | AUGAGUUUCUUCAAAUCAUUU | GAAAAGUUUGAAGAAGCUCAU | Cleavage |
| ccp-miR2642-3p | Cc03_g12710 | 3.0 | 16.246 | 1 | 21 | 108 | 128 | AUGAGUUUCUUCAAAUCAUUU | GAAUGAUUUGGAGAAACUGAA | Cleavage |
| ccp-miR2642-3p | Cc03_g13660 | 3.0 | 15.32 | 1 | 21 | 1424 | 1444 | AUGAGUUUCUUCAAAUCAUUU | AUAUGAUUUCAAGGAGCUCAU | Cleavage |
| ccp-miR2642-3p | Cc03_g16180 | 3.0 | 16.347 | 1 | 21 | 980 | 1000 | AUGAGUUUCUUCAAAUCAUUU | GAUUAAUUUGAAUAAACUCAU | Translation |
| ccp-miR2642-3p | Cc04_g03120 | 1.5 | 14.135 | 1 | 20 | 1347 | 1366 | AUGAGUUUCUUCAAAUCAUU | GAUGAUUUGAAGAAACUCAC | Cleavage |
| ccp-miR2642-3p | Cc05_g03370 | 3.0 | 20.596 | 1 | 21 | 52 | 72 | AUGAGUUUCUUCAAAUCAUUU | AAGGGAUAUGAAGAAAUUCAU | Cleavage |
| ccp-miR2642-3p | Cc06_g09760 | 3.0 | 15.614 | 1 | 21 | 139 | 159 | AUGAGUUUCUUCAAAUCAUUU | AAAAGAAUUGGGGAAACUCAU | Cleavage |
| ccp-miR2642-3p | Cc06_g19350 | 2.0 | 13.284 | 1 | 21 | 2086 | 2106 | AUGAGUUUCUUCAAAUCAUUU | AAAUGAUUUGAAUAAAUUUAU | Translation |
| ccp-miR2642-3p | Cc07_g11340 | 3.0 | 12.885 | 1 | 21 | 1341 | 1361 | AUGAGUUUCUUCAAAUCAUUU | GAAUGAUGUGAAAGAACUUAU | Translation |
| ccp-miR2642-3p | Cc08_g04850 | 3.0 | 12.885 | 1 | 21 | 1341 | 1361 | AUGAGUUUCUUCAAAUCAUUU | GAAUGAUGUGAAAGAACUUAU | Translation |
| ccp-miR2642-3p | Cc10_g09100 | 3.0 | 13.552 | 1 | 20 | 1864 | 1883 | AUGAGUUUCUUCAAAUCAUU | AAUGAUUUGACGGGAUUUAU | Translation |
| ccp-miR2657-3p | Cc00_g17320 | 3.0 | 17.255 | 1 | 20 | 1095 | 1114 | UGUUAUUUCAACGGUUUUGU | ACAAAACUGCUGAAAUAGCU | Translation |
| ccp-miR2657-3p | Cc00_g28770 | 3.0 | 14.778 | 1 | 21 | 232 | 252 | UGUUAUUUCAACGGUUUUGUU | AGGAAAAUUGUUGAAAUAGCA | Cleavage |
| ccp-miR2657-3p | Cc02_g12530 | 2.0 | 17.077 | 1 | 22 | 1822 | 1843 | UGUUAUUUCAACGGUUUUGUUA | UAACAAAGCUGGUGAAAUAACA | Translation |
| ccp-miR2657-3p | Cc07_g05420 | 3.0 | 3.498 | 1 | 22 | 18 | 39 | UGUUAUUUCAACGGUUUUGUUA | UAACAGAAACAUUGAAAUGACA | Cleavage |
| ccp-miR2669a-5p | Cc00_g25360 | 2.5 | 12.393 | 1 | 20 | 842 | 861 | AAAGUUCAGUCUUCAAAAUG | CAUUUUGAUGGAUGAACUUU | Translation |
| ccp-miR2669a-5p | Cc04_g02540 | 3.0 | 15.538 | 1 | 21 | 1340 | 1360 | AAAGUUCAGUCUUCAAAAUGU | ACACUUUGAAGAAAGAACUUU | Translation |
| ccp-miR2669a-5p | Cc07_g07350 | 2.5 | 15.906 | 1 | 20 | 256 | 275 | AAAGUUCAGUCUUCAAAAUG | UAUUUUGAAGAAUGGAUUUU | Translation |
| ccp-miR2669a-5p | Cc07_g19530 | 3.0 | 8.679 | 1 | 21 | 1709 | 1729 | AAAGUUCAGUCUUCAAAAUGU | ACAUUUUAAAAACUGAACUUG | Translation |
| ccp-miR2669a-5p | Cc09_g02640 | 2.0 | 19.589 | 1 | 21 | 1969 | 1989 | AAAGUUCAGUCUUCAAAAUGU | AUAUUUUGGAGACCGAACUUU | Cleavage |
| ccp-miR2669a-5p | Cc10_g03700 | 3.0 | 21.023 | 1 | 21 | 787 | 808 | AAAGUUCAGUCUUCAA-AAUGU | AUAUUCUUGAAGAAUGAACUUU | Translation |
| ccp-miR2669a-5p | Cc10_g11710 | 2.5 | 11.01 | 1 | 20 | 243 | 262 | AAAGUUCAGUCUUCAAAAUG | CAUUUUGAUGGAUGAACUUU | Translation |
| ccp-miR2669a-5p | Cc11_g16560 | 2.5 | 22.97 | 1 | 21 | 686 | 706 | AAAGUUCAGUCUUCAAAAUGU | ACAUUGUGGAGACUGGGCUUU | Cleavage |
| ccp-miR2673a-1-5p | Cc00_g00230 | 2.5 | 5.573 | 1 | 22 | 286 | 307 | CCUCUUCCUCUUCCUCUUUCAU | GUGAAAGAAGUAGAGGGAGAGG | Cleavage |
| ccp-miR2673a-1-5p | Cc00_g01020 | 3.0 | 11.023 | 1 | 22 | 472 | 493 | CCUCUUCCUCUUCCUCUUUCAU | AUGGAGGAGGAGGAGGAGGAGA | Cleavage |
| ccp-miR2673a-1-5p | Cc00_g01970 | 2.0 | 19.63 | 1 | 20 | 145 | 164 | CCUCUUCCUCUUCCUCUUUC | GAAAGAGGAGGAGGAGGAGC | Cleavage |
| ccp-miR2673a-1-5p | Cc00_g02330 | 3.0 | 15.133 | 1 | 22 | 62 | 83 | CCUCUUCCUCUUCCUCUUUCAU | GUGGAGCAGGAGGAGGAGGAGG | Cleavage |
| ccp-miR2673a-1-5p | Cc00_g04130 | 2.0 | 14.034 | 1 | 20 | 2104 | 2123 | CCUCUUCCUCUUCCUCUUUC | GGAAGGGGAAGGGGAAGGGG | Cleavage |
| ccp-miR2673a-1-5p | Cc00_g05920 | 2.0 | 2.444 | 1 | 20 | 1573 | 1592 | CCUCUUCCUCUUCCUCUUUC | GGAGGAGGAGGAGGAGGAGG | Cleavage |
| ccp-miR2673a-1-5p | Cc00_g07840 | 2.5 | 19.502 | 1 | 21 | 824 | 844 | CCUCUUCCUCUUCCUCUUUCA | UGGAGGAGGAAGAAGAGGAGG | Cleavage |
| ccp-miR2673a-1-5p | Cc00_g08730 | 2.5 | 20.847 | 1 | 20 | 1000 | 1019 | CCUCUUCCUCUUCCUCUUUC | AGAAGAGGAGGAGGAGGAGG | Cleavage |
| ccp-miR2673a-1-5p | Cc00_g10130 | 1.5 | 21.129 | 1 | 22 | 1372 | 1393 | CCUCUUCCUCUUCCUCUUUCAU | AUGGAAGAGGGAGAGGGAGAGG | Cleavage |
| ccp-miR2673a-1-5p | Cc00_g10620 | 2.0 | 8.435 | 1 | 22 | 7 | 28 | CCUCUUCCUCUUCCUCUUUCAU | GUGGAGGAGGAGGAGGAGGAGG | Cleavage |
| ccp-miR2673a-1-5p | Cc00_g31120 | 2.0 | 23.663 | 1 | 22 | 1201 | 1222 | CCUCUUCCUCUUCCUCUUUCAU | AAGGAGGAGGAGGAGGAGGAGG | Cleavage |
| ccp-miR2673a-1-5p | Cc01_g03320 | 2.5 | 9.399 | 1 | 20 | 206 | 225 | CCUCUUCCUCUUCCUCUUUC | GAGAGGGGGAGAGGAAGAGC | Cleavage |
| ccp-miR2673a-1-5p | Cc01_g03340 | 2.0 | 8.326 | 1 | 20 | 1179 | 1198 | CCUCUUCCUCUUCCUCUUUC | GGAGGAGGAGGAGGAGGAGG | Cleavage |
| ccp-miR2673a-1-5p | Cc01_g06370 | 1.5 | 10.457 | 1 | 21 | 827 | 847 | CCUCUUCCUCUUCCUCUUUCA | UGAAAGAAGAGGAGGAAGAGG | Cleavage |
| ccp-miR2673a-1-5p | Cc01_g06470 | 2.0 | 6.199 | 1 | 20 | 699 | 718 | CCUCUUCCUCUUCCUCUUUC | AGAGGAGGAAGAGGAAGAGG | Cleavage |
| ccp-miR2673a-1-5p | Cc01_g07160 | 1.5 | 16.038 | 1 | 22 | 564 | 585 | CCUCUUCCUCUUCCUCUUUCAU | GUGGGAGAGGAAGGGGAAGAGG | Cleavage |
| ccp-miR2673a-1-5p | Cc01_g07840 | 2.5 | 16.776 | 1 | 20 | 35 | 54 | CCUCUUCCUCUUCCUCUUUC | GGAGGAGGAGGAGGAGGGGG | Cleavage |
| ccp-miR2673a-1-5p | Cc01_g09070 | 3.0 | 1.211 | 1 | 22 | 28 | 49 | CCUCUUCCUCUUCCUCUUUCAU | GUGGAAGAAGAAGAGGAAGUGG | Cleavage |
| ccp-miR2673a-1-5p | Cc01_g10100 | 2.0 | 8.211 | 1 | 20 | 204 | 223 | CCUCUUCCUCUUCCUCUUUC | GAGAGAGGAAGAGGAAGAAG | Cleavage |
| ccp-miR2673a-1-5p | Cc01_g12890 | 2.5 | 6.036 | 1 | 20 | 904 | 923 | CCUCUUCCUCUUCCUCUUUC | AGAGGAGGAGGAGGAAGAGG | Cleavage |
| ccp-miR2673a-1-5p | Cc01_g12890 | 3.0 | 6.764 | 1 | 22 | 977 | 998 | CCUCUUCCUCUUCCUCUUUCAU | AUCGAAGAGGAGGAAGAAGAGG | Cleavage |
| ccp-miR2673a-1-5p | Cc01_g14680 | 2.5 | 15.232 | 1 | 22 | 1129 | 1150 | CCUCUUCCUCUUCCUCUUUCAU | AUGUAGGAGGAGGAGGAGGAGG | Cleavage |
| ccp-miR2673a-1-5p | Cc01_g14710 | 2.0 | 21.473 | 1 | 21 | 1807 | 1827 | CCUCUUCCUCUUCCUCUUUCA | UGGAGGAGGAGGAGGAGGAGG | Cleavage |
| ccp-miR2673a-1-5p | Cc01_g16900 | 2.0 | 18.846 | 1 | 20 | 1940 | 1959 | CCUCUUCCUCUUCCUCUUUC | GGAGGAGGAGGAGGAGGAGG | Cleavage |
| ccp-miR2673a-1-5p | Cc01_g18390 | 1.5 | 21.664 | 1 | 21 | 704 | 724 | CCUCUUCCUCUUCCUCUUUCA | UGAAGGAGGAGGAGGAGGAGG | Cleavage |
| ccp-miR2673a-1-5p | Cc01_g20540 | 2.5 | 14.87 | 1 | 20 | 21 | 40 | CCUCUUCCUCUUCCUCUUUC | GGGGGAGGGAGAGGGAGAGG | Cleavage |
| ccp-miR2673a-1-5p | Cc01_g20930 | 3.0 | 16.365 | 1 | 22 | 158 | 179 | CCUCUUCCUCUUCCUCUUUCAU | GUCAAAGAGGAGGAGGAGGAGA | Cleavage |
| ccp-miR2673a-1-5p | Cc01_g21740 | 2.0 | 10.532 | 1 | 20 | 297 | 316 | CCUCUUCCUCUUCCUCUUUC | GGAGGAGGAGGAGGAGGAGG | Cleavage |
| ccp-miR2673a-1-5p | Cc01_g21960 | 2.0 | 7.005 | 1 | 22 | 169 | 190 | CCUCUUCCUCUUCCUCUUUCAU | AAGGAGGAGGAGGAGGAGGAGG | Cleavage |
| ccp-miR2673a-1-5p | Cc02_g00460 | 2.0 | 17.799 | 1 | 22 | 6 | 27 | CCUCUUCCUCUUCCUCUUUCAU | GUGGAGGAGAAAGAGGAAGAGG | Cleavage |
| ccp-miR2673a-1-5p | Cc02_g00840 | 1.5 | 6.705 | 1 | 22 | 496 | 517 | CCUCUUCCUCUUCCUCUUUCAU | GUGGAAGAGGAGGAGGAGGAGG | Cleavage |
| ccp-miR2673a-1-5p | Cc02_g01240 | 2.5 | 24.127 | 1 | 20 | 19 | 38 | CCUCUUCCUCUUCCUCUUUC | GAGAGAGGGAGAGGGAGAGA | Cleavage |
| ccp-miR2673a-1-5p | Cc02_g02430 | 2.0 | 12.007 | 1 | 20 | 148 | 167 | CCUCUUCCUCUUCCUCUUUC | GGAGGAGGAGGAGGAGGAGG | Cleavage |
| ccp-miR2673a-1-5p | Cc02_g02560 | 2.0 | 7.442 | 1 | 20 | 166 | 185 | CCUCUUCCUCUUCCUCUUUC | GGGAGAGGAGGAGGGAGAGG | Cleavage |
| ccp-miR2673a-1-5p | Cc02_g10870 | 1.0 | 5.786 | 1 | 22 | 583 | 604 | CCUCUUCCUCUUCCUCUUUCAU | GUGGAAGAGGAAGAGGAGGAGG | Cleavage |
| ccp-miR2673a-1-5p | Cc02_g12290 | 2.0 | 10.819 | 1 | 20 | 1284 | 1303 | CCUCUUCCUCUUCCUCUUUC | GGAGGAGGAAGAAGAAGAGG | Cleavage |
| ccp-miR2673a-1-5p | Cc02_g12450 | 3.0 | 19.142 | 1 | 22 | 145 | 166 | CCUCUUCCUCUUCCUCUUUCAU | GUGGAAGAGGAAGAGCAAGAGC | Cleavage |
| ccp-miR2673a-1-5p | Cc02_g12900 | 1.5 | 10.744 | 1 | 20 | 1393 | 1412 | CCUCUUCCUCUUCCUCUUUC | GGAGGAGGAAGAGGAGGAGG | Cleavage |
| ccp-miR2673a-1-5p | Cc02_g12900 | 1.5 | 6.401 | 1 | 20 | 1414 | 1433 | CCUCUUCCUCUUCCUCUUUC | GGAGGAGGAAGAGGAGGAGG | Cleavage |
| ccp-miR2673a-1-5p | Cc02_g17040 | 2.5 | 21.679 | 1 | 22 | 452 | 473 | CCUCUUCCUCUUCCUCUUUCAU | AUGGUAGGGGAAGAGGAGGAGG | Cleavage |
| ccp-miR2673a-1-5p | Cc02_g18790 | 2.0 | 22.268 | 1 | 20 | 1876 | 1895 | CCUCUUCCUCUUCCUCUUUC | GGAGGAGGAGGAGGAGGAGG | Cleavage |
| ccp-miR2673a-1-5p | Cc02_g22790 | 2.0 | 9.983 | 1 | 20 | 1685 | 1704 | CCUCUUCCUCUUCCUCUUUC | GGAGGAGGAGGAGGAGGAGG | Cleavage |
| ccp-miR2673a-1-5p | Cc02_g25100 | 2.0 | 9.826 | 1 | 20 | 1085 | 1104 | CCUCUUCCUCUUCCUCUUUC | GGAGGAGGAGGAGGAGGAGG | Cleavage |
| ccp-miR2673a-1-5p | Cc02_g26140 | 2.0 | 10.266 | 1 | 22 | 116 | 137 | CCUCUUCCUCUUCCUCUUUCAU | AAGGAGGAGGAAAAGGAAGAGG | Translation |
| ccp-miR2673a-1-5p | Cc02_g26220 | 2.5 | 14.311 | 1 | 20 | 776 | 795 | CCUCUUCCUCUUCCUCUUUC | AAAAGAGGAAGGGGAAGAGC | Cleavage |
| ccp-miR2673a-1-5p | Cc02_g27010 | 1.5 | 23.08 | 1 | 20 | 449 | 468 | CCUCUUCCUCUUCCUCUUUC | GAAAGGGGAAGGGGAAGGGG | Cleavage |
| ccp-miR2673a-1-5p | Cc02_g32220 | 3.0 | 11.675 | 1 | 21 | 375 | 395 | CCUCUUCCUCUUCCUCUUUCA | UGGAGGAGGAGGAGGAAAAGG | Cleavage |
| ccp-miR2673a-1-5p | Cc02_g32560 | 2.0 | 6.373 | 1 | 20 | 630 | 649 | CCUCUUCCUCUUCCUCUUUC | AGAAGAGGAAGAGGAGGAGG | Cleavage |
| ccp-miR2673a-1-5p | Cc02_g33650 | 2.0 | 13.329 | 1 | 20 | 995 | 1014 | CCUCUUCCUCUUCCUCUUUC | GGAAGGGGAAGAGGAGGGGG | Cleavage |
| ccp-miR2673a-1-5p | Cc02_g34160 | 3.0 | 16.488 | 1 | 22 | 1303 | 1324 | CCUCUUCCUCUUCCUCUUUCAU | GUGGAAGAGGAAGAGGUAGAGA | Cleavage |
| ccp-miR2673a-1-5p | Cc02_g34200 | 3.0 | 9.467 | 1 | 21 | 441 | 461 | CCUCUUCCUCUUCCUCUUUCA | UGGAGGAGGAGGAGGAGGAGA | Cleavage |
| ccp-miR2673a-1-5p | Cc02_g34300 | 2.0 | 17.404 | 1 | 20 | 474 | 493 | CCUCUUCCUCUUCCUCUUUC | AAAAGAGGAGGAGGAGGAGG | Cleavage |
| ccp-miR2673a-1-5p | Cc02_g36360 | 2.5 | 6.896 | 1 | 20 | 1184 | 1203 | CCUCUUCCUCUUCCUCUUUC | CAAGGAGGAGGAGGAGGAGG | Cleavage |
| ccp-miR2673a-1-5p | Cc02_g37630 | 1.5 | 7.602 | 1 | 20 | 1732 | 1751 | CCUCUUCCUCUUCCUCUUUC | GAAAGAAGAAGAGGAGGAGG | Cleavage |
| ccp-miR2673a-1-5p | Cc02_g39020 | 2.5 | 20.907 | 1 | 20 | 470 | 489 | CCUCUUCCUCUUCCUCUUUC | AAAGGAGGAGGAGGAGGAGG | Cleavage |
| ccp-miR2673a-1-5p | Cc03_g00120 | 2.5 | 7.383 | 1 | 22 | 454 | 475 | CCUCUUCCUCUUCCUCUUUCAU | AGGAAGGAGGAGGAGGAGGAGA | Cleavage |
| ccp-miR2673a-1-5p | Cc03_g00650 | 0.5 | 19.093 | 1 | 20 | 19 | 38 | CCUCUUCCUCUUCCUCUUUC | GGAAGAGGAAGAGGAAGAGG | Cleavage |
| ccp-miR2673a-1-5p | Cc03_g00690 | 2.5 | 17.968 | 1 | 20 | 89 | 108 | CCUCUUCCUCUUCCUCUUUC | AGAAGAGGAGGAGGAGGAGG | Cleavage |
| ccp-miR2673a-1-5p | Cc03_g00710 | 2.0 | 9.699 | 1 | 20 | 163 | 182 | CCUCUUCCUCUUCCUCUUUC | GGAGGAGGAGGAGGAGGAGG | Cleavage |
| ccp-miR2673a-1-5p | Cc03_g00870 | 1.5 | 15.477 | 1 | 20 | 829 | 848 | CCUCUUCCUCUUCCUCUUUC | GGAGGAGGAGGAGGAAGAGG | Cleavage |
| ccp-miR2673a-1-5p | Cc03_g00980 | 2.0 | 14.93 | 1 | 20 | 796 | 815 | CCUCUUCCUCUUCCUCUUUC | AAGAGAGGAGGAGGAAGAGG | Cleavage |
| ccp-miR2673a-1-5p | Cc03_g02360 | 1.5 | 6.555 | 1 | 20 | 554 | 573 | CCUCUUCCUCUUCCUCUUUC | GAAGGAGGAGGAGGAGGAGG | Cleavage |
| ccp-miR2673a-1-5p | Cc03_g05550 | 2.0 | 16.022 | 1 | 20 | 198 | 217 | CCUCUUCCUCUUCCUCUUUC | GGAGGAGGAGGAGGAGGAGG | Cleavage |
| ccp-miR2673a-1-5p | Cc03_g07650 | 2.0 | 11.232 | 1 | 22 | 99 | 120 | CCUCUUCCUCUUCCUCUUUCAU | GUGGAGGAGGAGGAGGAGGAGG | Cleavage |
| ccp-miR2673a-1-5p | Cc03_g15730 | 2.0 | 21.9 | 1 | 22 | 80 | 101 | CCUCUUCCUCUUCCUCUUUCAU | GUGGAGGAGAAAGAGGAAGAGG | Cleavage |
| ccp-miR2673a-1-5p | Cc03_g15980 | 1.5 | 7.992 | 1 | 20 | 666 | 685 | CCUCUUCCUCUUCCUCUUUC | GAAAGAGGAGGAUGAAGAGG | Cleavage |
| ccp-miR2673a-1-5p | Cc04_g00200 | 3.0 | 10.805 | 1 | 22 | 155 | 176 | CCUCUUCCUCUUCCUCUUUCAU | GUGGAAUAGGGGGAGGAGGAGG | Cleavage |
| ccp-miR2673a-1-5p | Cc04_g01970 | 2.0 | 15.214 | 1 | 20 | 224 | 243 | CCUCUUCCUCUUCCUCUUUC | GGAGGAGGAGGAGGAGGAGG | Cleavage |
| ccp-miR2673a-1-5p | Cc04_g01980 | 2.5 | 4.088 | 1 | 20 | 690 | 709 | CCUCUUCCUCUUCCUCUUUC | AGAAGAGGAGGAGGAGGAGG | Cleavage |
| ccp-miR2673a-1-5p | Cc04_g02450 | 2.0 | 5.313 | 1 | 20 | 623 | 642 | CCUCUUCCUCUUCCUCUUUC | GAAGGAGGAUGAGGAGGAGG | Translation |
| ccp-miR2673a-1-5p | Cc04_g03550 | 2.5 | 8.001 | 1 | 20 | 650 | 669 | CCUCUUCCUCUUCCUCUUUC | CGAAGAGGAGGAGGAGGAGG | Cleavage |
| ccp-miR2673a-1-5p | Cc04_g04320 | 2.5 | 15.658 | 1 | 20 | 943 | 962 | CCUCUUCCUCUUCCUCUUUC | UGAAGAGGAGGAGGAGGAGG | Cleavage |
| ccp-miR2673a-1-5p | Cc04_g07130 | 1.5 | 13.777 | 1 | 20 | 813 | 832 | CCUCUUCCUCUUCCUCUUUC | GGAGGAGGAGGAGGAAGAGG | Cleavage |
| ccp-miR2673a-1-5p | Cc04_g09600 | 1.0 | 11.779 | 1 | 20 | 261 | 280 | CCUCUUCCUCUUCCUCUUUC | GAAGGAGGAGGAGGAAGAGG | Cleavage |
| ccp-miR2673a-1-5p | Cc04_g11850 | 2.0 | 24.477 | 1 | 20 | 2000 | 2019 | CCUCUUCCUCUUCCUCUUUC | GGAGGAGGAGGAGGAGGAGG | Cleavage |
| ccp-miR2673a-1-5p | Cc04_g12130 | 1.5 | 2.369 | 1 | 20 | 613 | 632 | CCUCUUCCUCUUCCUCUUUC | GGAGGAGGAGGAGGAAGAGG | Cleavage |
| ccp-miR2673a-1-5p | Cc04_g17260 | 2.0 | 10.071 | 1 | 20 | 1318 | 1337 | CCUCUUCCUCUUCCUCUUUC | GGAGGAGGAGGAGGAGGAGG | Cleavage |
| ccp-miR2673a-1-5p | Cc05_g00710 | 2.0 | 21.9 | 1 | 22 | 80 | 101 | CCUCUUCCUCUUCCUCUUUCAU | GUGGAGGAGAAAGAGGAAGAGG | Cleavage |
| ccp-miR2673a-1-5p | Cc05_g03110 | 0.5 | 12.274 | 1 | 20 | 1573 | 1592 | CCUCUUCCUCUUCCUCUUUC | GGAAGAGGAAGAGGAAGAGG | Cleavage |
| ccp-miR2673a-1-5p | Cc05_g07190 | 1.5 | 3.382 | 1 | 20 | 336 | 355 | CCUCUUCCUCUUCCUCUUUC | GGAGGAGGAGGAGGAAGAGG | Cleavage |
| ccp-miR2673a-1-5p | Cc05_g08020 | 2.0 | 6.483 | 1 | 20 | 570 | 589 | CCUCUUCCUCUUCCUCUUUC | GAGGGAGGAGGAGGAGGAGG | Cleavage |
| ccp-miR2673a-1-5p | Cc05_g10310 | 2.5 | 22.663 | 1 | 20 | 1425 | 1444 | CCUCUUCCUCUUCCUCUUUC | AAAGGAGGAAGAGGAAGAGA | Cleavage |
| ccp-miR2673a-1-5p | Cc05_g11850 | 1.0 | 10.393 | 1 | 20 | 404 | 423 | CCUCUUCCUCUUCCUCUUUC | GGAGGAGGAAGAGGAAGAGG | Cleavage |
| ccp-miR2673a-1-5p | Cc05_g14350 | 3.0 | 23.65 | 1 | 21 | 230 | 250 | CCUCUUCCUCUUCCUCUUUCA | UGGAGGAGGAGGAGGAGGAGA | Cleavage |
| ccp-miR2673a-1-5p | Cc05_g14910 | 2.0 | 22.189 | 1 | 21 | 312 | 332 | CCUCUUCCUCUUCCUCUUUCA | UGGAGGAGGAGGAGGAGGAGG | Cleavage |
| ccp-miR2673a-1-5p | Cc05_g16590 | 2.0 | 12.496 | 1 | 22 | 960 | 981 | CCUCUUCCUCUUCCUCUUUCAU | GUGGAAGGGGAGGAGGAGGAGG | Cleavage |
| ccp-miR2673a-1-5p | Cc06_g00260 | 2.0 | 16.853 | 1 | 20 | 982 | 1001 | CCUCUUCCUCUUCCUCUUUC | GGAGGAGGAGGAGGAGGAGG | Cleavage |
| ccp-miR2673a-1-5p | Cc06_g01660 | 2.5 | 12.987 | 1 | 22 | 688 | 709 | CCUCUUCCUCUUCCUCUUUCAU | GUGGAAGAAGAAGACGAAGAGG | Cleavage |
| ccp-miR2673a-1-5p | Cc06_g01670 | 2.0 | 9.647 | 1 | 20 | 568 | 587 | CCUCUUCCUCUUCCUCUUUC | GGAGGAGGAGGAGGAGGAGG | Cleavage |
| ccp-miR2673a-1-5p | Cc06_g03880 | 2.0 | 20.347 | 1 | 20 | 1396 | 1415 | CCUCUUCCUCUUCCUCUUUC | AAAAGAGGAGGAGGAGGAGG | Cleavage |
| ccp-miR2673a-1-5p | Cc06_g03920 | 2.5 | 18.041 | 1 | 20 | 358 | 377 | CCUCUUCCUCUUCCUCUUUC | GGAGGGGGAGGAGGAGGAGG | Cleavage |
| ccp-miR2673a-1-5p | Cc06_g05090 | 1.5 | 23.526 | 1 | 20 | 463 | 482 | CCUCUUCCUCUUCCUCUUUC | GAAGGAGGAGGAGGAGGAGG | Cleavage |
| ccp-miR2673a-1-5p | Cc06_g06170 | 2.0 | 15.612 | 1 | 21 | 101 | 121 | CCUCUUCCUCUUCCUCUUUCA | UGGAGGAGGAGGAGGAGGAGG | Cleavage |
| ccp-miR2673a-1-5p | Cc06_g07420 | 2.5 | 23.64 | 1 | 20 | 632 | 651 | CCUCUUCCUCUUCCUCUUUC | CGAAGAGGAGGAGGAGGAGG | Cleavage |
| ccp-miR2673a-1-5p | Cc06_g08120 | 3.0 | 9.637 | 1 | 22 | 389 | 410 | CCUCUUCCUCUUCCUCUUUCAU | AUGAGAGAAGAGGGGGAGGAGG | Cleavage |
| ccp-miR2673a-1-5p | Cc06_g11410 | 2.0 | 23.383 | 1 | 20 | 462 | 481 | CCUCUUCCUCUUCCUCUUUC | GGAGGAGGAGGAGGAGGAGG | Cleavage |
| ccp-miR2673a-1-5p | Cc06_g13000 | 2.0 | 19.307 | 1 | 20 | 177 | 196 | CCUCUUCCUCUUCCUCUUUC | GAAGGAGGAAGAGGAAGAAG | Cleavage |
| ccp-miR2673a-1-5p | Cc06_g17310 | 1.0 | 20.339 | 1 | 22 | 2556 | 2577 | CCUCUUCCUCUUCCUCUUUCAU | AUGGAAGAGGAAGAGGGAGAGG | Cleavage |
| ccp-miR2673a-1-5p | Cc06_g17620 | 2.0 | 10.542 | 1 | 22 | 1269 | 1290 | CCUCUUCCUCUUCCUCUUUCAU | GUCAAGGGGGAAGAGGAAGAGG | Cleavage |
| ccp-miR2673a-1-5p | Cc06_g19070 | 2.0 | 14.492 | 1 | 20 | 114 | 133 | CCUCUUCCUCUUCCUCUUUC | GGGAGAGGCAGAGGAAGAGG | Cleavage |
| ccp-miR2673a-1-5p | Cc06_g19080 | 2.0 | 24.423 | 1 | 22 | 2175 | 2196 | CCUCUUCCUCUUCCUCUUUCAU | GUGGAAGAGGCAGAGGAAGGGG | Cleavage |
| ccp-miR2673a-1-5p | Cc06_g20810 | 2.5 | 15.579 | 1 | 20 | 220 | 239 | CCUCUUCCUCUUCCUCUUUC | GGAGGAGGAAGAGGUAGAGG | Cleavage |
| ccp-miR2673a-1-5p | Cc06_g21600 | 3.0 | 9.944 | 1 | 22 | 228 | 249 | CCUCUUCCUCUUCCUCUUUCAU | GUGGUGGAGGAGGAGGAGGAGG | Cleavage |
| ccp-miR2673a-1-5p | Cc06_g21630 | 2.0 | 22.139 | 1 | 22 | 488 | 509 | CCUCUUCCUCUUCCUCUUUCAU | GUGGAGGAGGAGGAGGAGGAGG | Cleavage |
| ccp-miR2673a-1-5p | Cc06_g22270 | 2.0 | 22.966 | 1 | 22 | 1483 | 1504 | CCUCUUCCUCUUCCUCUUUCAU | AUGGAGGAGGAGGAGGAGGAGG | Cleavage |
| ccp-miR2673a-1-5p | Cc07_g01150 | 2.0 | 12.794 | 1 | 20 | 340 | 359 | CCUCUUCCUCUUCCUCUUUC | GGAGGAGGAGGAGGAGGAGG | Cleavage |
| ccp-miR2673a-1-5p | Cc07_g02060 | 2.0 | 9.149 | 1 | 20 | 328 | 347 | CCUCUUCCUCUUCCUCUUUC | GGAGGAGGAGGAGGAGGAGG | Cleavage |
| ccp-miR2673a-1-5p | Cc07_g03350 | 2.5 | 11.136 | 1 | 20 | 215 | 234 | CCUCUUCCUCUUCCUCUUUC | GAGAGAGGGAGAGGGAGAGA | Cleavage |
| ccp-miR2673a-1-5p | Cc07_g03910 | 1.5 | 4.209 | 1 | 22 | 2755 | 2776 | CCUCUUCCUCUUCCUCUUUCAU | AAGAAGGAGGAGGAGGAGGAGG | Cleavage |
| ccp-miR2673a-1-5p | Cc07_g04100 | 2.0 | 21.024 | 1 | 20 | 179 | 198 | CCUCUUCCUCUUCCUCUUUC | GGAGGAGGAGGAGGAGGAGG | Cleavage |
| ccp-miR2673a-1-5p | Cc07_g05730 | 2.5 | 19.968 | 1 | 20 | 540 | 559 | CCUCUUCCUCUUCCUCUUUC | AGAAGAGGAGGAGGAGGAGG | Cleavage |
| ccp-miR2673a-1-5p | Cc07_g06030 | 2.0 | 13.522 | 1 | 20 | 320 | 339 | CCUCUUCCUCUUCCUCUUUC | GGAGGAGGAAGAGGAAGAGU | Cleavage |
| ccp-miR2673a-1-5p | Cc07_g06610 | 1.5 | 23.584 | 1 | 22 | 1865 | 1886 | CCUCUUCCUCUUCCUCUUUCAU | AAGAAGGAGGAGGAGGAGGAGG | Cleavage |
| ccp-miR2673a-1-5p | Cc07_g10280 | 2.0 | 18.648 | 1 | 21 | 639 | 659 | CCUCUUCCUCUUCCUCUUUCA | UGGAGGAGGAGGAGGAGGAGG | Cleavage |
| ccp-miR2673a-1-5p | Cc07_g11070 | 2.0 | 17.404 | 1 | 20 | 555 | 574 | CCUCUUCCUCUUCCUCUUUC | AAAAGAGGAGGAGGAGGAGG | Cleavage |
| ccp-miR2673a-1-5p | Cc07_g14600 | 3.0 | 17.627 | 1 | 22 | 1595 | 1616 | CCUCUUCCUCUUCCUCUUUCAU | AUGGAGGAGGAGGAGGAGGAGU | Cleavage |
| ccp-miR2673a-1-5p | Cc07_g14750 | 1.5 | 18.064 | 1 | 20 | 121 | 140 | CCUCUUCCUCUUCCUCUUUC | UAAAGAGGAGGAGGAAGAGG | Cleavage |
| ccp-miR2673a-1-5p | Cc07_g15770 | 1.5 | 11.62 | 1 | 20 | 2456 | 2475 | CCUCUUCCUCUUCCUCUUUC | GGAAGAGGACGAGGAAGAGG | Translation |
| ccp-miR2673a-1-5p | Cc07_g17350 | 2.0 | 4.245 | 1 | 22 | 864 | 885 | CCUCUUCCUCUUCCUCUUUCAU | GUGGAGGAGGAGGAGGAGGAGG | Cleavage |
| ccp-miR2673a-1-5p | Cc07_g17870 | 2.0 | 15.709 | 1 | 20 | 818 | 837 | CCUCUUCCUCUUCCUCUUUC | GAAACAGGAGGAGGAGGAGG | Cleavage |
| ccp-miR2673a-1-5p | Cc08_g03150 | 1.5 | 24.504 | 1 | 22 | 135 | 156 | CCUCUUCCUCUUCCUCUUUCAU | GUGAGAGAGGGAGAGGGAGAGG | Cleavage |
| ccp-miR2673a-1-5p | Cc08_g03570 | 3.0 | 6.921 | 1 | 22 | 658 | 679 | CCUCUUCCUCUUCCUCUUUCAU | GUGGAGGUGGAGGAGGAGGAGG | Cleavage |
| ccp-miR2673a-1-5p | Cc08_g10720 | 2.5 | 15.984 | 1 | 22 | 276 | 297 | CCUCUUCCUCUUCCUCUUUCAU | AAGAAAGGGGGAGGGGAAGAGU | Cleavage |
| ccp-miR2673a-1-5p | Cc08_g12550 | 2.5 | 9.401 | 1 | 22 | 687 | 708 | CCUCUUCCUCUUCCUCUUUCAU | GUGAAAGAGUCAGAGGAGGAGG | Cleavage |
| ccp-miR2673a-1-5p | Cc08_g16470 | 3.0 | 9.148 | 1 | 22 | 18 | 39 | CCUCUUCCUCUUCCUCUUUCAU | AGGAGGGAGGAAGGGGGAGAGA | Cleavage |
| ccp-miR2673a-1-5p | Cc08_g16940 | 2.0 | 15.707 | 1 | 20 | 837 | 856 | CCUCUUCCUCUUCCUCUUUC | GGAGGAGGAGGAGGAGGAGG | Cleavage |
| ccp-miR2673a-1-5p | Cc09_g00180 | 2.0 | 19.202 | 1 | 20 | 1 | 20 | CCUCUUCCUCUUCCUCUUUC | GGAGGAGGAGGAGGAGGAGG | Cleavage |
| ccp-miR2673a-1-5p | Cc09_g00920 | 1.5 | 8.855 | 1 | 20 | 861 | 880 | CCUCUUCCUCUUCCUCUUUC | GGAAGAUGAAGAGGAAGAGG | Cleavage |
| ccp-miR2673a-1-5p | Cc09_g02810 | 2.0 | 20.971 | 1 | 20 | 417 | 436 | CCUCUUCCUCUUCCUCUUUC | GGAGGAGGAGGAGGAGGAGG | Cleavage |
| ccp-miR2673a-1-5p | Cc09_g03190 | 2.5 | 14.379 | 1 | 20 | 294 | 313 | CCUCUUCCUCUUCCUCUUUC | AAAGGAGGAGGAGGAGGAGG | Cleavage |
| ccp-miR2673a-1-5p | Cc09_g03650 | 2.5 | 18.484 | 1 | 22 | 4 | 25 | CCUCUUCCUCUUCCUCUUUCAU | AUGGAAGAGGGAGAAGAGGAGG | Cleavage |
| ccp-miR2673a-1-5p | Cc09_g04650 | 2.5 | 18.941 | 1 | 21 | 2208 | 2228 | CCUCUUCCUCUUCCUCUUUCA | UUAAGGAGGAGGAGGAGGAGG | Cleavage |
| ccp-miR2673a-1-5p | Cc09_g05310 | 1.5 | 10.783 | 1 | 20 | 593 | 612 | CCUCUUCCUCUUCCUCUUUC | GAAGGAGGAAGACGAAGAGG | Cleavage |
| ccp-miR2673a-1-5p | Cc09_g06510 | 2.0 | 20.687 | 1 | 20 | 365 | 384 | CCUCUUCCUCUUCCUCUUUC | GGAGGAGGAGGAGGAGGAGG | Cleavage |
| ccp-miR2673a-1-5p | Cc09_g08070 | 2.0 | 14.161 | 1 | 22 | 515 | 536 | CCUCUUCCUCUUCCUCUUUCAU | GUGGAGGAGGAGGAGGAGGAGG | Cleavage |
| ccp-miR2673a-1-5p | Cc09_g08670 | 3.0 | 15.119 | 1 | 22 | 211 | 232 | CCUCUUCCUCUUCCUCUUUCAU | GUGAAAGAGGAGGAGGUAGAGU | Cleavage |
| ccp-miR2673a-1-5p | Cc09_g09330 | 2.5 | 18.456 | 1 | 20 | 1038 | 1057 | CCUCUUCCUCUUCCUCUUUC | AAAGGAGGAGGAGGAGGAGG | Cleavage |
| ccp-miR2673a-1-5p | Cc09_g09380 | 1.0 | 14.73 | 1 | 20 | 554 | 573 | CCUCUUCCUCUUCCUCUUUC | AAAAGAGGAAGAGGAAGAGG | Cleavage |
| ccp-miR2673a-1-5p | Cc09_g10610 | 1.5 | 5.632 | 1 | 20 | 186 | 205 | CCUCUUCCUCUUCCUCUUUC | GAAGGAGGAGGAGGAGGAGG | Cleavage |
| ccp-miR2673a-1-5p | Cc09_g10930 | 2.0 | 12.472 | 1 | 20 | 3379 | 3398 | CCUCUUCCUCUUCCUCUUUC | GGAGGAGGAGGAGGAGGAGG | Cleavage |
| ccp-miR2673a-1-5p | Cc10_g02120 | 3.0 | 16.547 | 1 | 22 | 65 | 86 | CCUCUUCCUCUUCCUCUUUCAU | GUGGAGGAGGAGGUGGAGGAGG | Translation |
| ccp-miR2673a-1-5p | Cc10_g03290 | 3.0 | 12.477 | 1 | 22 | 501 | 522 | CCUCUUCCUCUUCCUCUUUCAU | GUGGAGGAGGAGAAGGAGGAGG | Translation |
| ccp-miR2673a-1-5p | Cc10_g04600 | 3.0 | 22.926 | 1 | 22 | 707 | 728 | CCUCUUCCUCUUCCUCUUUCAU | AUGCAAGGGGAAGAGCAAGAGG | Cleavage |
| ccp-miR2673a-1-5p | Cc10_g04730 | 2.5 | 12.832 | 1 | 20 | 1215 | 1234 | CCUCUUCCUCUUCCUCUUUC | UGAAGGGGAGGAGGAAGAGG | Cleavage |
| ccp-miR2673a-1-5p | Cc10_g06040 | 2.0 | 5.943 | 1 | 20 | 1457 | 1476 | CCUCUUCCUCUUCCUCUUUC | GGAGGAGGAGGAGGAGGAGG | Cleavage |
| ccp-miR2673a-1-5p | Cc10_g08960 | 2.0 | 3.872 | 1 | 20 | 78 | 97 | CCUCUUCCUCUUCCUCUUUC | AAAAGAGGAAGAGGAAGAGA | Cleavage |
| ccp-miR2673a-1-5p | Cc10_g09010 | 2.5 | 7.461 | 1 | 20 | 1029 | 1048 | CCUCUUCCUCUUCCUCUUUC | GAAAGAGGAAGAGAAAGAGA | Cleavage |
| ccp-miR2673a-1-5p | Cc10_g09010 | 2.5 | 4.761 | 1 | 20 | 1134 | 1153 | CCUCUUCCUCUUCCUCUUUC | GAAAGAGGAAGAGAAAGAGA | Cleavage |
| ccp-miR2673a-1-5p | Cc10_g09510 | 1.5 | 0.475 | 1 | 22 | 984 | 1005 | CCUCUUCCUCUUCCUCUUUCAU | AAGAGAGAGGGAGAGGGAGAGG | Cleavage |
| ccp-miR2673a-1-5p | Cc10_g10610 | 2.0 | 12.133 | 1 | 20 | 184 | 203 | CCUCUUCCUCUUCCUCUUUC | GGGAGAGGGAGAGGGAGAGG | Cleavage |
| ccp-miR2673a-1-5p | Cc10_g12890 | 2.0 | 17.006 | 1 | 20 | 1539 | 1558 | CCUCUUCCUCUUCCUCUUUC | GGAAGAGGAAGAAGGAGAGG | Cleavage |
| ccp-miR2673a-1-5p | Cc10_g14440 | 2.0 | 4.807 | 1 | 20 | 864 | 883 | CCUCUUCCUCUUCCUCUUUC | GGAGGAGGAGGAGGGAGAGG | Cleavage |
| ccp-miR2673a-1-5p | Cc10_g14970 | 2.0 | 11.907 | 1 | 20 | 846 | 865 | CCUCUUCCUCUUCCUCUUUC | UAAAGAAGAAGAGGAAGAGG | Cleavage |
| ccp-miR2673a-1-5p | Cc10_g15540 | 2.5 | 21.192 | 1 | 21 | 531 | 551 | CCUCUUCCUCUUCCUCUUUCA | UGGAAGAGGAGGAGGAGGAGA | Cleavage |
| ccp-miR2673a-1-5p | Cc11_g00220 | 3.0 | 22.082 | 1 | 21 | 689 | 709 | CCUCUUCCUCUUCCUCUUUCA | UGGAGGAGAAGGAGGAGGAGG | Cleavage |
| ccp-miR2673a-1-5p | Cc11_g00540 | 1.5 | 13.006 | 1 | 20 | 142 | 161 | CCUCUUCCUCUUCCUCUUUC | GAAGGAGGAGGAGGAGGAGG | Cleavage |
| ccp-miR2673a-1-5p | Cc11_g02320 | 2.5 | 20.768 | 1 | 21 | 3426 | 3446 | CCUCUUCCUCUUCCUCUUUCA | UUGAAGAAGAAGAGGAAGAGG | Cleavage |
| ccp-miR2673a-1-5p | Cc11_g05060 | 2.0 | 17.339 | 1 | 22 | 459 | 480 | CCUCUUCCUCUUCCUCUUUCAU | GUGGAGGAGGAGGAGGAGGAGG | Cleavage |
| ccp-miR2673a-1-5p | Cc11_g05230 | 2.0 | 18.949 | 1 | 20 | 596 | 615 | CCUCUUCCUCUUCCUCUUUC | GGAGGAGGAGGAGGAGGAGG | Cleavage |
| ccp-miR2673a-1-5p | Cc11_g10310 | 3.0 | 3.902 | 1 | 21 | 1434 | 1454 | CCUCUUCCUCUUCCUCUUUCA | UGGGAGAGGGAGAAGGAGAGG | Cleavage |
| ccp-miR2673a-1-5p | Cc11_g11510 | 2.0 | 15.729 | 1 | 20 | 223 | 242 | CCUCUUCCUCUUCCUCUUUC | GGAAGAGGAAGAAGAGGAGG | Cleavage |
| ccp-miR2673a-1-5p | Cc11_g11850 | 2.0 | 12.159 | 1 | 20 | 513 | 532 | CCUCUUCCUCUUCCUCUUUC | GGAGGAGGAAGAAGAAGAGG | Cleavage |
| ccp-miR2673a-1-5p | Cc11_g12680 | 2.5 | 6.898 | 1 | 20 | 294 | 313 | CCUCUUCCUCUUCCUCUUUC | GGAGGAGGAGGAGGAGGGGG | Cleavage |
| ccp-miR2673a-1-5p | Cc11_g14870 | 2.5 | 0.422 | 1 | 20 | 104 | 123 | CCUCUUCCUCUUCCUCUUUC | GAGAGAGGGAGAGGGAGAGA | Cleavage |
| ccp-miR2673a-2-3p | Cc00_g01020 | 2.5 | 11.023 | 1 | 21 | 473 | 493 | CCUCUUCCUCUUCCUCUUCCG | UGGAGGAGGAGGAGGAGGAGA | Cleavage |
| ccp-miR2673a-2-3p | Cc00_g02330 | 2.5 | 15.133 | 1 | 22 | 62 | 83 | CCUCUUCCUCUUCCUCUUCCGC | GUGGAGCAGGAGGAGGAGGAGG | Cleavage |
| ccp-miR2673a-2-3p | Cc00_g04130 | 1.5 | 14.034 | 1 | 22 | 2102 | 2123 | CCUCUUCCUCUUCCUCUUCCGC | GAGGAAGGGGAAGGGGAAGGGG | Cleavage |
| ccp-miR2673a-2-3p | Cc00_g05920 | 1.5 | 2.444 | 1 | 22 | 1571 | 1592 | CCUCUUCCUCUUCCUCUUCCGC | GAGGAGGAGGAGGAGGAGGAGG | Cleavage |
| ccp-miR2673a-2-3p | Cc00_g07840 | 2.0 | 19.502 | 1 | 21 | 824 | 844 | CCUCUUCCUCUUCCUCUUCCG | UGGAGGAGGAAGAAGAGGAGG | Cleavage |
| ccp-miR2673a-2-3p | Cc00_g08730 | 2.0 | 20.847 | 1 | 20 | 1000 | 1019 | CCUCUUCCUCUUCCUCUUCC | AGAAGAGGAGGAGGAGGAGG | Cleavage |
| ccp-miR2673a-2-3p | Cc00_g09560 | 2.5 | 14.469 | 1 | 22 | 268 | 289 | CCUCUUCCUCUUCCUCUUCCGC | GAGGAGGAGGAGGAGGAUGAGG | Cleavage |
| ccp-miR2673a-2-3p | Cc00_g10130 | 1.0 | 21.129 | 1 | 21 | 1373 | 1393 | CCUCUUCCUCUUCCUCUUCCG | UGGAAGAGGGAGAGGGAGAGG | Cleavage |
| ccp-miR2673a-2-3p | Cc00_g10620 | 1.5 | 8.435 | 1 | 22 | 7 | 28 | CCUCUUCCUCUUCCUCUUCCGC | GUGGAGGAGGAGGAGGAGGAGG | Cleavage |
| ccp-miR2673a-2-3p | Cc00_g13080 | 3.0 | 14.566 | 1 | 22 | 765 | 786 | CCUCUUCCUCUUCCUCUUCCGC | GCAGAGGAGGAAGAAGAGGAGG | Cleavage |
| ccp-miR2673a-2-3p | Cc00_g26700 | 2.5 | 17.494 | 1 | 20 | 2061 | 2080 | CCUCUUCCUCUUCCUCUUCC | UGAGGAGGAGGAGGAGGAGG | Cleavage |
| ccp-miR2673a-2-3p | Cc00_g31120 | 1.5 | 23.663 | 1 | 20 | 1203 | 1222 | CCUCUUCCUCUUCCUCUUCC | GGAGGAGGAGGAGGAGGAGG | Cleavage |
| ccp-miR2673a-2-3p | Cc00_g32520 | 2.5 | 20.219 | 1 | 20 | 927 | 946 | CCUCUUCCUCUUCCUCUUCC | UGAGGAGGAGGAGGAGGAGG | Cleavage |
| ccp-miR2673a-2-3p | Cc01_g03340 | 1.5 | 8.326 | 1 | 22 | 1177 | 1198 | CCUCUUCCUCUUCCUCUUCCGC | GAGGAGGAGGAGGAGGAGGAGG | Cleavage |
| ccp-miR2673a-2-3p | Cc01_g06370 | 2.5 | 8.702 | 1 | 20 | 834 | 853 | CCUCUUCCUCUUCCUCUUCC | AGAGGAGGAAGAGGAAGAGA | Cleavage |
| ccp-miR2673a-2-3p | Cc01_g06470 | 1.5 | 6.199 | 1 | 22 | 697 | 718 | CCUCUUCCUCUUCCUCUUCCGC | GCAGAGGAGGAAGAGGAAGAGG | Cleavage |
| ccp-miR2673a-2-3p | Cc01_g07160 | 1.0 | 16.038 | 1 | 22 | 564 | 585 | CCUCUUCCUCUUCCUCUUCCGC | GUGGGAGAGGAAGGGGAAGAGG | Cleavage |
| ccp-miR2673a-2-3p | Cc01_g07840 | 2.0 | 16.776 | 1 | 22 | 33 | 54 | CCUCUUCCUCUUCCUCUUCCGC | GCGGAGGAGGAGGAGGAGGGGG | Cleavage |
| ccp-miR2673a-2-3p | Cc01_g09070 | 2.5 | 1.211 | 1 | 22 | 28 | 49 | CCUCUUCCUCUUCCUCUUCCGC | GUGGAAGAAGAAGAGGAAGUGG | Cleavage |
| ccp-miR2673a-2-3p | Cc01_g10100 | 1.0 | 6.792 | 1 | 22 | 208 | 229 | CCUCUUCCUCUUCCUCUUCCGC | GAGGAAGAGGAAGAAGAAGAGG | Cleavage |
| ccp-miR2673a-2-3p | Cc01_g11520 | 3.0 | 8.455 | 1 | 22 | 283 | 304 | CCUCUUCCUCUUCCUCUUCCGC | GUGGAGGAGGAAAAGGAGGAGA | Translation |
| ccp-miR2673a-2-3p | Cc01_g12890 | 2.0 | 6.036 | 1 | 20 | 904 | 923 | CCUCUUCCUCUUCCUCUUCC | AGAGGAGGAGGAGGAAGAGG | Cleavage |
| ccp-miR2673a-2-3p | Cc01_g14710 | 1.5 | 21.473 | 1 | 21 | 1807 | 1827 | CCUCUUCCUCUUCCUCUUCCG | UGGAGGAGGAGGAGGAGGAGG | Cleavage |
| ccp-miR2673a-2-3p | Cc01_g16900 | 2.0 | 17.2 | 1 | 22 | 1932 | 1953 | CCUCUUCCUCUUCCUCUUCCGC | GCGGAUGAGGAGGAGGAGGAGG | Cleavage |
| ccp-miR2673a-2-3p | Cc01_g20540 | 2.0 | 14.87 | 1 | 22 | 19 | 40 | CCUCUUCCUCUUCCUCUUCCGC | GAGGGGGAGGGAGAGGGAGAGG | Cleavage |
| ccp-miR2673a-2-3p | Cc01_g21150 | 2.5 | 22.25 | 1 | 20 | 534 | 553 | CCUCUUCCUCUUCCUCUUCC | CGAGGAGGAGGAGGAGGAGG | Cleavage |
| ccp-miR2673a-2-3p | Cc01_g21740 | 1.5 | 7.918 | 1 | 22 | 298 | 319 | CCUCUUCCUCUUCCUCUUCCGC | GAGGAGGAGGAGGAGGAGGAGG | Cleavage |
| ccp-miR2673a-2-3p | Cc01_g21960 | 1.5 | 6.478 | 1 | 22 | 172 | 193 | CCUCUUCCUCUUCCUCUUCCGC | GAGGAGGAGGAGGAGGAGGAGG | Cleavage |
| ccp-miR2673a-2-3p | Cc02_g00460 | 1.5 | 17.799 | 1 | 22 | 6 | 27 | CCUCUUCCUCUUCCUCUUCCGC | GUGGAGGAGAAAGAGGAAGAGG | Cleavage |
| ccp-miR2673a-2-3p | Cc02_g00840 | 1.0 | 6.705 | 1 | 22 | 496 | 517 | CCUCUUCCUCUUCCUCUUCCGC | GUGGAAGAGGAGGAGGAGGAGG | Cleavage |
| ccp-miR2673a-2-3p | Cc02_g02430 | 1.5 | 12.007 | 1 | 20 | 148 | 167 | CCUCUUCCUCUUCCUCUUCC | GGAGGAGGAGGAGGAGGAGG | Cleavage |
| ccp-miR2673a-2-3p | Cc02_g02560 | 1.5 | 16.486 | 1 | 22 | 254 | 275 | CCUCUUCCUCUUCCUCUUCCGC | GCGGAAGAGGAGGCGGAAGAGG | Translation |
| ccp-miR2673a-2-3p | Cc02_g03970 | 2.0 | 8.674 | 1 | 21 | 61 | 81 | CCUCUUCCUCUUCCUCUUCCG | CGGAAGAAGAAGAAGAAGAGG | Cleavage |
| ccp-miR2673a-2-3p | Cc02_g10870 | 0.5 | 5.786 | 1 | 22 | 583 | 604 | CCUCUUCCUCUUCCUCUUCCGC | GUGGAAGAGGAAGAGGAGGAGG | Cleavage |
| ccp-miR2673a-2-3p | Cc02_g12290 | 1.5 | 10.819 | 1 | 22 | 1282 | 1303 | CCUCUUCCUCUUCCUCUUCCGC | GGGGAGGAGGAAGAAGAAGAGG | Cleavage |
| ccp-miR2673a-2-3p | Cc02_g12450 | 2.5 | 19.142 | 1 | 22 | 145 | 166 | CCUCUUCCUCUUCCUCUUCCGC | GUGGAAGAGGAAGAGCAAGAGC | Cleavage |
| ccp-miR2673a-2-3p | Cc02_g12900 | 1.0 | 10.744 | 1 | 22 | 1391 | 1412 | CCUCUUCCUCUUCCUCUUCCGC | GAGGAGGAGGAAGAGGAGGAGG | Cleavage |
| ccp-miR2673a-2-3p | Cc02_g17040 | 2.0 | 21.679 | 1 | 21 | 453 | 473 | CCUCUUCCUCUUCCUCUUCCG | UGGUAGGGGAAGAGGAGGAGG | Cleavage |
| ccp-miR2673a-2-3p | Cc02_g18790 | 1.5 | 22.268 | 1 | 22 | 1874 | 1895 | CCUCUUCCUCUUCCUCUUCCGC | GCGGAGGAGGAGGAGGAGGAGG | Cleavage |
| ccp-miR2673a-2-3p | Cc02_g21500 | 3.0 | 18.893 | 1 | 22 | 134 | 155 | CCUCUUCCUCUUCCUCUUCCGC | GUGGACGAGGAGGAGGAGGAGA | Cleavage |
| ccp-miR2673a-2-3p | Cc02_g21730 | 2.0 | 11.231 | 1 | 20 | 2043 | 2062 | CCUCUUCCUCUUCCUCUUCC | GGAAGAGGAAGAAGAAGAGU | Cleavage |
| ccp-miR2673a-2-3p | Cc02_g22790 | 1.5 | 9.983 | 1 | 22 | 1683 | 1704 | CCUCUUCCUCUUCCUCUUCCGC | GAGGAGGAGGAGGAGGAGGAGG | Cleavage |
| ccp-miR2673a-2-3p | Cc02_g25100 | 1.5 | 9.826 | 1 | 22 | 1083 | 1104 | CCUCUUCCUCUUCCUCUUCCGC | GAGGAGGAGGAGGAGGAGGAGG | Cleavage |
| ccp-miR2673a-2-3p | Cc02_g26140 | 1.5 | 10.266 | 1 | 20 | 118 | 137 | CCUCUUCCUCUUCCUCUUCC | GGAGGAGGAAAAGGAAGAGG | Translation |
| ccp-miR2673a-2-3p | Cc02_g26500 | 3.0 | 12.44 | 1 | 22 | 358 | 379 | CCUCUUCCUCUUCCUCUUCCGC | GCGGAAGAAGAGGAGGAAGAAG | Cleavage |
| ccp-miR2673a-2-3p | Cc02_g28760 | 3.0 | 19.097 | 1 | 22 | 64 | 85 | CCUCUUCCUCUUCCUCUUCCGC | GCGGAGGAGGAUGAGGAUGAGG | Translation |
| ccp-miR2673a-2-3p | Cc02_g32560 | 1.5 | 6.373 | 1 | 20 | 630 | 649 | CCUCUUCCUCUUCCUCUUCC | AGAAGAGGAAGAGGAGGAGG | Cleavage |
| ccp-miR2673a-2-3p | Cc02_g33650 | 1.5 | 13.329 | 1 | 20 | 995 | 1014 | CCUCUUCCUCUUCCUCUUCC | GGAAGGGGAAGAGGAGGGGG | Cleavage |
| ccp-miR2673a-2-3p | Cc02_g34160 | 2.5 | 16.488 | 1 | 22 | 1303 | 1324 | CCUCUUCCUCUUCCUCUUCCGC | GUGGAAGAGGAAGAGGUAGAGA | Cleavage |
| ccp-miR2673a-2-3p | Cc02_g34200 | 2.5 | 9.467 | 1 | 21 | 441 | 461 | CCUCUUCCUCUUCCUCUUCCG | UGGAGGAGGAGGAGGAGGAGA | Cleavage |
| ccp-miR2673a-2-3p | Cc02_g34300 | 1.5 | 11.252 | 1 | 22 | 478 | 499 | CCUCUUCCUCUUCCUCUUCCGC | GAGGAGGAGGAGGAGGAGGAGG | Cleavage |
| ccp-miR2673a-2-3p | Cc03_g00650 | 0.0 | 19.093 | 1 | 22 | 17 | 38 | CCUCUUCCUCUUCCUCUUCCGC | GAGGAAGAGGAAGAGGAAGAGG | Cleavage |
| ccp-miR2673a-2-3p | Cc03_g00690 | 2.0 | 17.968 | 1 | 20 | 89 | 108 | CCUCUUCCUCUUCCUCUUCC | AGAAGAGGAGGAGGAGGAGG | Cleavage |
| ccp-miR2673a-2-3p | Cc03_g00710 | 1.5 | 9.699 | 1 | 20 | 163 | 182 | CCUCUUCCUCUUCCUCUUCC | GGAGGAGGAGGAGGAGGAGG | Cleavage |
| ccp-miR2673a-2-3p | Cc03_g00860 | 3.0 | 17.077 | 1 | 22 | 276 | 297 | CCUCUUCCUCUUCCUCUUCCGC | GCUGAAGAAGAGGAGGAGGAGG | Cleavage |
| ccp-miR2673a-2-3p | Cc03_g00870 | 1.0 | 15.477 | 1 | 22 | 827 | 848 | CCUCUUCCUCUUCCUCUUCCGC | GAGGAGGAGGAGGAGGAAGAGG | Cleavage |
| ccp-miR2673a-2-3p | Cc03_g00980 | 2.0 | 13.599 | 1 | 22 | 800 | 821 | CCUCUUCCUCUUCCUCUUCCGC | GAGGAGGAGGAAGAGGAAGAAG | Cleavage |
| ccp-miR2673a-2-3p | Cc03_g02360 | 1.5 | 5.499 | 1 | 20 | 557 | 576 | CCUCUUCCUCUUCCUCUUCC | GGAGGAGGAGGAGGAGGAGG | Cleavage |
| ccp-miR2673a-2-3p | Cc03_g04370 | 2.0 | 24.468 | 1 | 22 | 430 | 451 | CCUCUUCCUCUUCCUCUUCCGC | GAGGAAGGAGAAGGGGAAGAGG | Cleavage |
| ccp-miR2673a-2-3p | Cc03_g05550 | 1.5 | 16.022 | 1 | 22 | 196 | 217 | CCUCUUCCUCUUCCUCUUCCGC | GCGGAGGAGGAGGAGGAGGAGG | Cleavage |
| ccp-miR2673a-2-3p | Cc03_g06370 | 2.5 | 20.828 | 1 | 22 | 1561 | 1582 | CCUCUUCCUCUUCCUCUUCCGC | GAGGAAGAGGAAGGGGUAGGGG | Cleavage |
| ccp-miR2673a-2-3p | Cc03_g07650 | 1.5 | 11.232 | 1 | 22 | 99 | 120 | CCUCUUCCUCUUCCUCUUCCGC | GUGGAGGAGGAGGAGGAGGAGG | Cleavage |
| ccp-miR2673a-2-3p | Cc03_g15730 | 1.5 | 21.9 | 1 | 22 | 80 | 101 | CCUCUUCCUCUUCCUCUUCCGC | GUGGAGGAGAAAGAGGAAGAGG | Cleavage |
| ccp-miR2673a-2-3p | Cc04_g00200 | 2.5 | 10.805 | 1 | 22 | 155 | 176 | CCUCUUCCUCUUCCUCUUCCGC | GUGGAAUAGGGGGAGGAGGAGG | Cleavage |
| ccp-miR2673a-2-3p | Cc04_g01970 | 1.5 | 15.214 | 1 | 22 | 222 | 243 | CCUCUUCCUCUUCCUCUUCCGC | GCGGAGGAGGAGGAGGAGGAGG | Cleavage |
| ccp-miR2673a-2-3p | Cc04_g01980 | 2.0 | 4.088 | 1 | 20 | 690 | 709 | CCUCUUCCUCUUCCUCUUCC | AGAAGAGGAGGAGGAGGAGG | Cleavage |
| ccp-miR2673a-2-3p | Cc04_g03550 | 2.0 | 8.001 | 1 | 20 | 650 | 669 | CCUCUUCCUCUUCCUCUUCC | CGAAGAGGAGGAGGAGGAGG | Cleavage |
| ccp-miR2673a-2-3p | Cc04_g04320 | 2.0 | 15.658 | 1 | 22 | 941 | 962 | CCUCUUCCUCUUCCUCUUCCGC | GCUGAAGAGGAGGAGGAGGAGG | Cleavage |
| ccp-miR2673a-2-3p | Cc04_g07130 | 1.0 | 13.777 | 1 | 22 | 811 | 832 | CCUCUUCCUCUUCCUCUUCCGC | GAGGAGGAGGAGGAGGAAGAGG | Cleavage |
| ccp-miR2673a-2-3p | Cc04_g08490 | 3.0 | 13.955 | 1 | 22 | 756 | 777 | CCUCUUCCUCUUCCUCUUCCGC | GCGGGGGUGGAGGAGGAGGAGG | Cleavage |
| ccp-miR2673a-2-3p | Cc04_g09450 | 2.0 | 12.582 | 1 | 22 | 707 | 728 | CCUCUUCCUCUUCCUCUUCCGC | GGGGAAGGGGAUGGGGAAGAGG | Translation |
| ccp-miR2673a-2-3p | Cc04_g09600 | 2.0 | 11.779 | 1 | 22 | 259 | 280 | CCUCUUCCUCUUCCUCUUCCGC | GAGAAGGAGGAGGAGGAAGAGG | Cleavage |
| ccp-miR2673a-2-3p | Cc04_g11850 | 1.5 | 24.477 | 1 | 22 | 1998 | 2019 | CCUCUUCCUCUUCCUCUUCCGC | GCGGAGGAGGAGGAGGAGGAGG | Cleavage |
| ccp-miR2673a-2-3p | Cc04_g12130 | 1.0 | 2.369 | 1 | 22 | 611 | 632 | CCUCUUCCUCUUCCUCUUCCGC | GAGGAGGAGGAGGAGGAAGAGG | Cleavage |
| ccp-miR2673a-2-3p | Cc04_g17260 | 1.5 | 10.071 | 1 | 22 | 1316 | 1337 | CCUCUUCCUCUUCCUCUUCCGC | GAGGAGGAGGAGGAGGAGGAGG | Cleavage |
| ccp-miR2673a-2-3p | Cc05_g00710 | 1.5 | 21.9 | 1 | 22 | 80 | 101 | CCUCUUCCUCUUCCUCUUCCGC | GUGGAGGAGAAAGAGGAAGAGG | Cleavage |
| ccp-miR2673a-2-3p | Cc05_g03110 | 0.0 | 12.274 | 1 | 22 | 1571 | 1592 | CCUCUUCCUCUUCCUCUUCCGC | GAGGAAGAGGAAGAGGAAGAGG | Cleavage |
| ccp-miR2673a-2-3p | Cc05_g07190 | 1.0 | 3.382 | 1 | 22 | 334 | 355 | CCUCUUCCUCUUCCUCUUCCGC | GAGGAGGAGGAGGAGGAAGAGG | Cleavage |
| ccp-miR2673a-2-3p | Cc05_g10900 | 2.5 | 8.922 | 1 | 20 | 514 | 533 | CCUCUUCCUCUUCCUCUUCC | AGAGGAGGGAGAGGAGGAGG | Cleavage |
| ccp-miR2673a-2-3p | Cc05_g11850 | 0.5 | 10.393 | 1 | 22 | 402 | 423 | CCUCUUCCUCUUCCUCUUCCGC | GAGGAGGAGGAAGAGGAAGAGG | Cleavage |
| ccp-miR2673a-2-3p | Cc05_g14350 | 2.5 | 23.65 | 1 | 21 | 230 | 250 | CCUCUUCCUCUUCCUCUUCCG | UGGAGGAGGAGGAGGAGGAGA | Cleavage |
| ccp-miR2673a-2-3p | Cc05_g14910 | 1.5 | 22.189 | 1 | 21 | 312 | 332 | CCUCUUCCUCUUCCUCUUCCG | UGGAGGAGGAGGAGGAGGAGG | Cleavage |
| ccp-miR2673a-2-3p | Cc05_g16590 | 1.5 | 12.496 | 1 | 22 | 960 | 981 | CCUCUUCCUCUUCCUCUUCCGC | GUGGAAGGGGAGGAGGAGGAGG | Cleavage |
| ccp-miR2673a-2-3p | Cc06_g00260 | 1.5 | 16.853 | 1 | 22 | 980 | 1001 | CCUCUUCCUCUUCCUCUUCCGC | GCGGAGGAGGAGGAGGAGGAGG | Cleavage |
| ccp-miR2673a-2-3p | Cc06_g01660 | 2.0 | 12.987 | 1 | 22 | 688 | 709 | CCUCUUCCUCUUCCUCUUCCGC | GUGGAAGAAGAAGACGAAGAGG | Cleavage |
| ccp-miR2673a-2-3p | Cc06_g01670 | 1.5 | 9.647 | 1 | 20 | 568 | 587 | CCUCUUCCUCUUCCUCUUCC | GGAGGAGGAGGAGGAGGAGG | Cleavage |
| ccp-miR2673a-2-3p | Cc06_g03920 | 2.0 | 18.041 | 1 | 21 | 357 | 377 | CCUCUUCCUCUUCCUCUUCCG | CGGAGGGGGAGGAGGAGGAGG | Cleavage |
| ccp-miR2673a-2-3p | Cc06_g06170 | 1.5 | 15.612 | 1 | 21 | 101 | 121 | CCUCUUCCUCUUCCUCUUCCG | UGGAGGAGGAGGAGGAGGAGG | Cleavage |
| ccp-miR2673a-2-3p | Cc06_g07420 | 2.0 | 23.64 | 1 | 20 | 632 | 651 | CCUCUUCCUCUUCCUCUUCC | CGAAGAGGAGGAGGAGGAGG | Cleavage |
| ccp-miR2673a-2-3p | Cc06_g08120 | 2.5 | 11.402 | 1 | 20 | 397 | 416 | CCUCUUCCUCUUCCUCUUCC | AGAGGGGGAGGAGGAAGAGG | Cleavage |
| ccp-miR2673a-2-3p | Cc06_g11410 | 1.5 | 23.383 | 1 | 22 | 460 | 481 | CCUCUUCCUCUUCCUCUUCCGC | GCGGAGGAGGAGGAGGAGGAGG | Cleavage |
| ccp-miR2673a-2-3p | Cc06_g12560 | 2.5 | 20.037 | 1 | 22 | 310 | 331 | CCUCUUCCUCUUCCUCUUCCGC | GAGGAGGAGGAGGAGGAGGAGA | Cleavage |
| ccp-miR2673a-2-3p | Cc06_g13000 | 1.5 | 18.7 | 1 | 22 | 181 | 202 | CCUCUUCCUCUUCCUCUUCCGC | GAGGAAGAGGAAGAAGAGGAGG | Cleavage |
| ccp-miR2673a-2-3p | Cc06_g13260 | 3.0 | 11.412 | 1 | 22 | 742 | 763 | CCUCUUCCUCUUCCUCUUCCGC | GUAGAGGAGGAGGAGGAAGAGA | Cleavage |
| ccp-miR2673a-2-3p | Cc06_g13650 | 3.0 | 4.842 | 1 | 22 | 14 | 35 | CCUCUUCCUCUUCCUCUUCCGC | GCGGCAGAGGAGGAGGAGGAGA | Cleavage |
| ccp-miR2673a-2-3p | Cc06_g17310 | 0.5 | 20.339 | 1 | 21 | 2557 | 2577 | CCUCUUCCUCUUCCUCUUCCG | UGGAAGAGGAAGAGGGAGAGG | Cleavage |
| ccp-miR2673a-2-3p | Cc06_g18300 | 2.5 | 18.311 | 1 | 21 | 67 | 87 | CCUCUUCCUCUUCCUCUUCCG | CGGAAGAGGCAGAGAAAGAGG | Cleavage |
| ccp-miR2673a-2-3p | Cc06_g19070 | 1.5 | 14.492 | 1 | 22 | 112 | 133 | CCUCUUCCUCUUCCUCUUCCGC | GAGGGAGAGGCAGAGGAAGAGG | Cleavage |
| ccp-miR2673a-2-3p | Cc06_g19080 | 1.5 | 24.423 | 1 | 22 | 2175 | 2196 | CCUCUUCCUCUUCCUCUUCCGC | GUGGAAGAGGCAGAGGAAGGGG | Cleavage |
| ccp-miR2673a-2-3p | Cc06_g20810 | 2.0 | 15.579 | 1 | 20 | 220 | 239 | CCUCUUCCUCUUCCUCUUCC | GGAGGAGGAAGAGGUAGAGG | Cleavage |
| ccp-miR2673a-2-3p | Cc06_g21600 | 2.5 | 9.944 | 1 | 22 | 228 | 249 | CCUCUUCCUCUUCCUCUUCCGC | GUGGUGGAGGAGGAGGAGGAGG | Cleavage |
| ccp-miR2673a-2-3p | Cc06_g21630 | 1.5 | 22.139 | 1 | 22 | 488 | 509 | CCUCUUCCUCUUCCUCUUCCGC | GUGGAGGAGGAGGAGGAGGAGG | Cleavage |
| ccp-miR2673a-2-3p | Cc06_g22270 | 1.5 | 22.966 | 1 | 21 | 1484 | 1504 | CCUCUUCCUCUUCCUCUUCCG | UGGAGGAGGAGGAGGAGGAGG | Cleavage |
| ccp-miR2673a-2-3p | Cc06_g23180 | 3.0 | 22.295 | 1 | 22 | 1467 | 1488 | CCUCUUCCUCUUCCUCUUCCGC | GAGGAAGAGGAAGGGGAAGACU | Cleavage |
| ccp-miR2673a-2-3p | Cc07_g01150 | 1.5 | 12.794 | 1 | 22 | 338 | 359 | CCUCUUCCUCUUCCUCUUCCGC | GAGGAGGAGGAGGAGGAGGAGG | Cleavage |
| ccp-miR2673a-2-3p | Cc07_g02060 | 1.5 | 9.149 | 1 | 22 | 326 | 347 | CCUCUUCCUCUUCCUCUUCCGC | GCGGAGGAGGAGGAGGAGGAGG | Cleavage |
| ccp-miR2673a-2-3p | Cc07_g03910 | 1.5 | 5.068 | 1 | 22 | 2761 | 2782 | CCUCUUCCUCUUCCUCUUCCGC | GAGGAGGAGGAGGAGGAGGAGG | Cleavage |
| ccp-miR2673a-2-3p | Cc07_g04100 | 1.5 | 21.024 | 1 | 22 | 177 | 198 | CCUCUUCCUCUUCCUCUUCCGC | GAGGAGGAGGAGGAGGAGGAGG | Cleavage |
| ccp-miR2673a-2-3p | Cc07_g05730 | 2.0 | 19.968 | 1 | 21 | 539 | 559 | CCUCUUCCUCUUCCUCUUCCG | CAGAAGAGGAGGAGGAGGAGG | Cleavage |
| ccp-miR2673a-2-3p | Cc07_g06030 | 1.5 | 13.522 | 1 | 20 | 320 | 339 | CCUCUUCCUCUUCCUCUUCC | GGAGGAGGAAGAGGAAGAGU | Cleavage |
| ccp-miR2673a-2-3p | Cc07_g06610 | 1.5 | 21.933 | 1 | 22 | 1871 | 1892 | CCUCUUCCUCUUCCUCUUCCGC | GAGGAGGAGGAGGAGGAGGAGG | Cleavage |
| ccp-miR2673a-2-3p | Cc07_g06610 | 1.5 | 17.758 | 1 | 22 | 1895 | 1916 | CCUCUUCCUCUUCCUCUUCCGC | GAGGAGGAGGAGGAGGAGGAGG | Cleavage |
| ccp-miR2673a-2-3p | Cc07_g08260 | 2.0 | 14.695 | 1 | 22 | 232 | 253 | CCUCUUCCUCUUCCUCUUCCGC | GAGGAAGAGGAAGAAGAAGAGU | Cleavage |
| ccp-miR2673a-2-3p | Cc07_g10280 | 1.5 | 18.648 | 1 | 21 | 639 | 659 | CCUCUUCCUCUUCCUCUUCCG | UGGAGGAGGAGGAGGAGGAGG | Cleavage |
| ccp-miR2673a-2-3p | Cc07_g11070 | 1.5 | 11.252 | 1 | 22 | 559 | 580 | CCUCUUCCUCUUCCUCUUCCGC | GAGGAGGAGGAGGAGGAGGAGG | Cleavage |
| ccp-miR2673a-2-3p | Cc07_g12520 | 3.0 | 8.861 | 1 | 21 | 929 | 949 | CCUCUUCCUCUUCCUCUUCCG | CUGAGGAGGAAGAGGAGGAGC | Cleavage |
| ccp-miR2673a-2-3p | Cc07_g14600 | 2.5 | 17.627 | 1 | 21 | 1596 | 1616 | CCUCUUCCUCUUCCUCUUCCG | UGGAGGAGGAGGAGGAGGAGU | Cleavage |
| ccp-miR2673a-2-3p | Cc07_g14750 | 2.5 | 18.064 | 1 | 20 | 121 | 140 | CCUCUUCCUCUUCCUCUUCC | UAAAGAGGAGGAGGAAGAGG | Cleavage |
| ccp-miR2673a-2-3p | Cc07_g15770 | 1.0 | 11.62 | 1 | 22 | 2454 | 2475 | CCUCUUCCUCUUCCUCUUCCGC | GAGGAAGAGGACGAGGAAGAGG | Translation |
| ccp-miR2673a-2-3p | Cc07_g17350 | 1.5 | 4.245 | 1 | 22 | 864 | 885 | CCUCUUCCUCUUCCUCUUCCGC | GUGGAGGAGGAGGAGGAGGAGG | Cleavage |
| ccp-miR2673a-2-3p | Cc07_g20150 | 2.5 | 8.342 | 1 | 22 | 249 | 270 | CCUCUUCCUCUUCCUCUUCCGC | GAGGAGGAGGAGGAGGAGGAGA | Cleavage |
| ccp-miR2673a-2-3p | Cc08_g03150 | 2.5 | 24.504 | 1 | 22 | 135 | 156 | CCUCUUCCUCUUCCUCUUCCGC | GUGAGAGAGGGAGAGGGAGAGG | Cleavage |
| ccp-miR2673a-2-3p | Cc08_g03160 | 2.0 | 6.871 | 1 | 20 | 545 | 564 | CCUCUUCCUCUUCCUCUUCC | AGAAGAAGAAGAGGAAGAGG | Cleavage |
| ccp-miR2673a-2-3p | Cc08_g03570 | 2.5 | 6.921 | 1 | 22 | 658 | 679 | CCUCUUCCUCUUCCUCUUCCGC | GUGGAGGUGGAGGAGGAGGAGG | Cleavage |
| ccp-miR2673a-2-3p | Cc08_g16800 | 2.5 | 22.132 | 1 | 20 | 397 | 416 | CCUCUUCCUCUUCCUCUUCC | AGGGGAGGAGGAGGAAGAGG | Cleavage |
| ccp-miR2673a-2-3p | Cc08_g16940 | 1.5 | 15.707 | 1 | 22 | 835 | 856 | CCUCUUCCUCUUCCUCUUCCGC | GAGGAGGAGGAGGAGGAGGAGG | Cleavage |
| ccp-miR2673a-2-3p | Cc09_g00180 | 1.5 | 19.202 | 1 | 22 | 2 | 23 | CCUCUUCCUCUUCCUCUUCCGC | GAGGAGGAGGAGGAGGAGGAGG | Cleavage |
| ccp-miR2673a-2-3p | Cc09_g00920 | 1.0 | 8.855 | 1 | 22 | 859 | 880 | CCUCUUCCUCUUCCUCUUCCGC | GAGGAAGAUGAAGAGGAAGAGG | Cleavage |
| ccp-miR2673a-2-3p | Cc09_g02810 | 1.5 | 20.971 | 1 | 22 | 415 | 436 | CCUCUUCCUCUUCCUCUUCCGC | GCGGAGGAGGAGGAGGAGGAGG | Cleavage |
| ccp-miR2673a-2-3p | Cc09_g03650 | 2.0 | 18.484 | 1 | 21 | 5 | 25 | CCUCUUCCUCUUCCUCUUCCG | UGGAAGAGGGAGAAGAGGAGG | Cleavage |
| ccp-miR2673a-2-3p | Cc09_g03750 | 2.5 | 22.363 | 1 | 22 | 2422 | 2443 | CCUCUUCCUCUUCCUCUUCCGC | GAGGGAGAGGGAGAGGGAGAGA | Cleavage |
| ccp-miR2673a-2-3p | Cc09_g05310 | 1.0 | 10.099 | 1 | 22 | 597 | 618 | CCUCUUCCUCUUCCUCUUCCGC | GAGGAAGACGAAGAGGAAGAGG | Cleavage |
| ccp-miR2673a-2-3p | Cc09_g06510 | 1.5 | 20.687 | 1 | 22 | 363 | 384 | CCUCUUCCUCUUCCUCUUCCGC | GCGGAGGAGGAGGAGGAGGAGG | Cleavage |
| ccp-miR2673a-2-3p | Cc09_g08070 | 1.5 | 14.161 | 1 | 22 | 515 | 536 | CCUCUUCCUCUUCCUCUUCCGC | GUGGAGGAGGAGGAGGAGGAGG | Cleavage |
| ccp-miR2673a-2-3p | Cc09_g09330 | 2.0 | 19.941 | 1 | 20 | 1041 | 1060 | CCUCUUCCUCUUCCUCUUCC | GGAGGAGGAGGAGGAGGGGG | Cleavage |
| ccp-miR2673a-2-3p | Cc09_g09380 | 2.0 | 14.73 | 1 | 20 | 554 | 573 | CCUCUUCCUCUUCCUCUUCC | AAAAGAGGAAGAGGAAGAGG | Cleavage |
| ccp-miR2673a-2-3p | Cc09_g10610 | 2.5 | 4.448 | 1 | 20 | 189 | 208 | CCUCUUCCUCUUCCUCUUCC | GGAGGAGGAGGAGGAGGAGA | Cleavage |
| ccp-miR2673a-2-3p | Cc09_g10930 | 1.5 | 12.472 | 1 | 22 | 3377 | 3398 | CCUCUUCCUCUUCCUCUUCCGC | GGGGAGGAGGAGGAGGAGGAGG | Cleavage |
| ccp-miR2673a-2-3p | Cc10_g00100 | 2.5 | 11.98 | 1 | 22 | 957 | 978 | CCUCUUCCUCUUCCUCUUCCGC | GCAGAGGAGGAGGAGGAGGAGG | Cleavage |
| ccp-miR2673a-2-3p | Cc10_g02120 | 2.5 | 16.547 | 1 | 22 | 65 | 86 | CCUCUUCCUCUUCCUCUUCCGC | GUGGAGGAGGAGGUGGAGGAGG | Translation |
| ccp-miR2673a-2-3p | Cc10_g03290 | 2.5 | 12.477 | 1 | 22 | 501 | 522 | CCUCUUCCUCUUCCUCUUCCGC | GUGGAGGAGGAGAAGGAGGAGG | Translation |
| ccp-miR2673a-2-3p | Cc10_g04730 | 2.0 | 12.832 | 1 | 21 | 1214 | 1234 | CCUCUUCCUCUUCCUCUUCCG | CUGAAGGGGAGGAGGAAGAGG | Cleavage |
| ccp-miR2673a-2-3p | Cc10_g05150 | 3.0 | 11.951 | 1 | 21 | 821 | 841 | CCUCUUCCUCUUCCUCUUCCG | CGGAGGAGGAGGAGGAGGAAG | Cleavage |
| ccp-miR2673a-2-3p | Cc10_g06040 | 1.5 | 5.943 | 1 | 22 | 1455 | 1476 | CCUCUUCCUCUUCCUCUUCCGC | GAGGAGGAGGAGGAGGAGGAGG | Cleavage |
| ccp-miR2673a-2-3p | Cc10_g08500 | 2.0 | 11.743 | 1 | 22 | 208 | 229 | CCUCUUCCUCUUCCUCUUCCGC | GAGGACGAGGAGGAGGAGGAGG | Cleavage |
| ccp-miR2673a-2-3p | Cc10_g09510 | 2.5 | 1.857 | 1 | 22 | 990 | 1011 | CCUCUUCCUCUUCCUCUUCCGC | GAGGGAGAGGGAGAGGGAGAGA | Cleavage |
| ccp-miR2673a-2-3p | Cc10_g10610 | 1.5 | 10.022 | 1 | 22 | 188 | 209 | CCUCUUCCUCUUCCUCUUCCGC | GAGGGAGAGGGAGAGGGAGAGG | Cleavage |
| ccp-miR2673a-2-3p | Cc10_g12890 | 1.5 | 17.006 | 1 | 20 | 1539 | 1558 | CCUCUUCCUCUUCCUCUUCC | GGAAGAGGAAGAAGGAGAGG | Cleavage |
| ccp-miR2673a-2-3p | Cc10_g14440 | 1.5 | 4.807 | 1 | 20 | 864 | 883 | CCUCUUCCUCUUCCUCUUCC | GGAGGAGGAGGAGGGAGAGG | Cleavage |
| ccp-miR2673a-2-3p | Cc10_g15540 | 2.0 | 21.192 | 1 | 21 | 531 | 551 | CCUCUUCCUCUUCCUCUUCCG | UGGAAGAGGAGGAGGAGGAGA | Cleavage |
| ccp-miR2673a-2-3p | Cc11_g00260 | 2.5 | 14.461 | 1 | 20 | 510 | 529 | CCUCUUCCUCUUCCUCUUCC | AGAAGAGGAGGAGGGAGGGG | Cleavage |
| ccp-miR2673a-2-3p | Cc11_g00900 | 2.5 | 15.128 | 1 | 22 | 557 | 578 | CCUCUUCCUCUUCCUCUUCCGC | GAGGAGGAGGAGGAGGAAAAGG | Cleavage |
| ccp-miR2673a-2-3p | Cc11_g02320 | 2.0 | 20.768 | 1 | 20 | 3427 | 3446 | CCUCUUCCUCUUCCUCUUCC | UGAAGAAGAAGAGGAAGAGG | Cleavage |
| ccp-miR2673a-2-3p | Cc11_g03910 | 2.0 | 12.654 | 1 | 22 | 436 | 457 | CCUCUUCCUCUUCCUCUUCCGC | GAGGAGGAGGAGGAAGAAGAGG | Cleavage |
| ccp-miR2673a-2-3p | Cc11_g05060 | 1.5 | 17.339 | 1 | 22 | 459 | 480 | CCUCUUCCUCUUCCUCUUCCGC | GUGGAGGAGGAGGAGGAGGAGG | Cleavage |
| ccp-miR2673a-2-3p | Cc11_g05230 | 1.5 | 18.949 | 1 | 20 | 596 | 615 | CCUCUUCCUCUUCCUCUUCC | GGAGGAGGAGGAGGAGGAGG | Cleavage |
| ccp-miR2673a-2-3p | Cc11_g07690 | 2.5 | 8.878 | 1 | 20 | 231 | 250 | CCUCUUCCUCUUCCUCUUCC | UGAAGAGGAAGAGGAAGAAG | Cleavage |
| ccp-miR2673a-2-3p | Cc11_g11510 | 1.5 | 15.729 | 1 | 20 | 223 | 242 | CCUCUUCCUCUUCCUCUUCC | GGAAGAGGAAGAAGAGGAGG | Cleavage |
| ccp-miR2673a-2-3p | Cc11_g11850 | 1.5 | 12.159 | 1 | 22 | 511 | 532 | CCUCUUCCUCUUCCUCUUCCGC | GAGGAGGAGGAAGAAGAAGAGG | Cleavage |
| ccp-miR2673a-2-3p | Cc11_g12680 | 2.0 | 6.898 | 1 | 22 | 292 | 313 | CCUCUUCCUCUUCCUCUUCCGC | GAGGAGGAGGAGGAGGAGGGGG | Cleavage |
| ccp-miR2673a-2-3p | Cc11_g14860 | 3.0 | 14.903 | 1 | 22 | 363 | 384 | CCUCUUCCUCUUCCUCUUCCGC | GCGGAGGAGGAGGAGGAAGGAG | Cleavage |
| ccp-miR2873b-1-5p | Cc00_g21960 | 3.0 | 9.465 | 1 | 20 | 1818 | 1837 | UUGGACUUGAGAUUUAGAAU | AGUUUCAAUCUCAAGUUCAA | Cleavage |
| ccp-miR2873b-1-5p | Cc00_g24560 | 3.0 | 13.15 | 1 | 20 | 103 | 122 | UUGGACUUGAGAUUUAGAAU | AUCCUAAACUUCAAGUCUAA | Cleavage |
| ccp-miR2873b-1-5p | Cc00_g26490 | 3.0 | 17.308 | 1 | 20 | 1379 | 1398 | UUGGACUUGAGAUUUAGAAU | AAUCUGAAUAUCAAGUUCAA | Translation |
| ccp-miR2873b-1-5p | Cc02_g35160 | 3.0 | 18.144 | 1 | 21 | 384 | 404 | UUGGACUUGAGAUUUAGAAUA | UGUACUAGAUCUCAGGUCUAA | Cleavage |
| ccp-miR2873b-1-5p | Cc04_g07250 | 3.0 | 16.184 | 1 | 20 | 1872 | 1891 | UUGGACUUGAGAUUUAGAAU | GUUCCAAAUAUCAAGUUCAA | Translation |
| ccp-miR2873b-1-5p | Cc06_g14900 | 2.5 | 16.808 | 1 | 20 | 1630 | 1649 | UUGGACUUGAGAUUUAGAAU | AUUUUAAGUUUCAAGUUUAA | Cleavage |
| ccp-miR2873b-1-5p | Cc06_g18040 | 3.0 | 8.51 | 1 | 20 | 593 | 611 | UUGGACUUGAGAUUUAGAAU | AUUCAAAAUC-CAAGUCCAA | Translation |
| ccp-miR2873b-1-5p | Cc07_g11390 | 3.0 | 13.365 | 1 | 20 | 728 | 747 | UUGGACUUGAGAUUUAGAAU | AUCCUAGAUCUCAAGUUCAU | Cleavage |
| ccp-miR2873b-1-5p | Cc08_g16260 | 3.0 | 13.312 | 1 | 20 | 990 | 1009 | UUGGACUUGAGAUUUAGAAU | GUUUUAAUUUUCAAGUUCAA | Cleavage |
| ccp-miR2873b-1-5p | Cc11_g01360 | 3.0 | 16.719 | 1 | 20 | 521 | 540 | UUGGACUUGAGAUUUAGAAU | GUUCUGAAUUUCAAGACCAA | Cleavage |
| ccp-miR2873b-1-5p | Cc11_g09480 | 3.0 | 20.551 | 1 | 21 | 1471 | 1491 | UUGGACUUGAGAUUUAGAAUA | UAUUCUAAAGCUCAAGCUCAA | Cleavage |
| ccp-miR2873b-2-5p | Cc00_g29010 | 3.0 | 11.973 | 1 | 21 | 165 | 185 | UUGGACUUGAGAUUUGGAGGU | AUCUCCAAAUUUCAAGUUCGU | Cleavage |
| ccp-miR2873b-2-5p | Cc02_g20480 | 2.5 | 21.242 | 1 | 20 | 11 | 30 | UUGGACUUGAGAUUUGGAGG | CUUCCAAGUUCCAAGUCCAA | Translation |
| ccp-miR2873b-2-5p | Cc02_g28720 | 3.0 | 20.675 | 1 | 20 | 2686 | 2705 | UUGGACUUGAGAUUUGGAGG | CCUCCAACUCUCAGGUCCGU | Cleavage |
| ccp-miR2873b-2-5p | Cc04_g07250 | 3.0 | 16.184 | 1 | 20 | 1872 | 1891 | UUGGACUUGAGAUUUGGAGG | GUUCCAAAUAUCAAGUUCAA | Translation |
| ccp-miR2873b-2-5p | Cc04_g07330 | 3.0 | 15.558 | 1 | 21 | 765 | 785 | UUGGACUUGAGAUUUGGAGGU | AUCUUCAACUCUCGAGUUCAA | Cleavage |
| ccp-miR2873b-2-5p | Cc04_g07600 | 3.0 | 11.312 | 1 | 21 | 1165 | 1185 | UUGGACUUGAGAUUUGGAGGU | GCUUCCAGAUCUCGAGUCAAA | Cleavage |
| ccp-miR2873b-2-5p | Cc05_g00050 | 3.0 | 16.933 | 1 | 21 | 1211 | 1231 | UUGGACUUGAGAUUUGGAGGU | GCCUCCGAAUCUCAGGUCAAG | Cleavage |
| ccp-miR2873b-2-5p | Cc05_g02420 | 2.5 | 18.238 | 1 | 20 | 292 | 310 | UUGGACUUGAGAUUUGGAGG | CCUCCAA-UCUCAAGUCCAG | Cleavage |
| ccp-miR2873b-2-5p | Cc05_g02520 | 2.0 | 7.066 | 1 | 20 | 135 | 154 | UUGGACUUGAGAUUUGGAGG | CCUUCCAAUUUCAAGUCCAA | Cleavage |
| ccp-miR2873b-2-5p | Cc05_g02520 | 2.0 | 9.61 | 1 | 20 | 195 | 214 | UUGGACUUGAGAUUUGGAGG | CCUUCCAAUUUCAAGUCCAA | Cleavage |
| ccp-miR2873b-2-5p | Cc05_g09270 | 3.0 | 9.792 | 1 | 21 | 165 | 185 | UUGGACUUGAGAUUUGGAGGU | AUCUCCAAAUUUCAAGUUCGU | Cleavage |
| ccp-miR2873b-2-5p | Cc06_g00590 | 2.5 | 18.433 | 1 | 21 | 2256 | 2276 | UUGGACUUGAGAUUUGGAGGU | ACCUUCAGGUUUCAAGUCCAG | Cleavage |
| ccp-miR2873b-2-5p | Cc07_g09990 | 3.0 | 11.608 | 1 | 20 | 966 | 985 | UUGGACUUGAGAUUUGGAGG | CCUCCAAAUUUUAUGUCCAG | Cleavage |
| ccp-miR2873b-2-5p | Cc08_g11720 | 3.0 | 12.33 | 1 | 20 | 555 | 574 | UUGGACUUGAGAUUUGGAGG | CGUCCAAAGCUCAAGUCCAC | Cleavage |
| ccp-miR2873b-2-5p | Cc08_g14510 | 3.0 | 22.486 | 1 | 20 | 1165 | 1184 | UUGGACUUGAGAUUUGGAGG | UCUCCAAAUUUUGAGUCCAC | Cleavage |
| ccp-miR2873b-2-5p | Cc10_g04850 | 2.5 | 8.824 | 1 | 20 | 479 | 497 | UUGGACUUGAGAUUUGGAGG | CCUCCAAAU-UCAGGUCCAA | Translation |
| ccp-miR2923-3p | Cc00_g14500 | 2.5 | 18.397 | 1 | 22 | 862 | 883 | AGACAAAAAUAUAGACACCAAA | UCUGGUGUCUUUAUGUUUGUUU | Cleavage |
| ccp-miR2923-3p | Cc00_g17420 | 3.0 | 9.104 | 1 | 22 | 1026 | 1047 | AGACAAAAAUAUAGACACCAAA | UUUGGUGUCUACCUCUUUGUCU | Translation |
| ccp-miR2923-3p | Cc01_g02140 | 3.0 | 20.546 | 1 | 22 | 1738 | 1759 | AGACAAAAAUAUAGACACCAAA | UCUGUUGGUUGUAUUUUUGUCU | Cleavage |
| ccp-miR2923-3p | Cc02_g26580 | 2.5 | 23.699 | 1 | 20 | 2883 | 2902 | AGACAAAAAUAUAGACACCA | UGGUGUUCAUGUUUUUGUUU | Cleavage |
| ccp-miR2923-3p | Cc04_g04980 | 3.0 | 14.584 | 1 | 20 | 1179 | 1197 | AGACAAAAAUAUAGACACCA | UGGUGU-UAUAUGUUUGUCU | Cleavage |
| ccp-miR2923-3p | Cc04_g06620 | 2.5 | 16.013 | 1 | 22 | 4370 | 4391 | AGACAAAAAUAUAGACACCAAA | UUUGGAUUCUAUAUUUUUGUUU | Cleavage |
| ccp-miR2923-3p | Cc08_g13560 | 3.0 | 21.224 | 1 | 20 | 1481 | 1501 | AGACAAA-AAUAUAGACACCA | UGGUGUCAAUAUUGUUUGUCU | Cleavage |
| ccp-miR319-1-3p | Cc01_g07440 | 2.5 | 21.661 | 1 | 20 | 1327 | 1346 | UUGGACUGAAGGGAGCUCCC | UGGAGCUCCCUUCACUCCAA | Cleavage |
| ccp-miR319-1-3p | Cc01_g13090 | 3.0 | 18.959 | 1 | 20 | 284 | 303 | UUGGACUGAAGGGAGCUCCC | UGGAGCUCCCUUCACUUCAA | Cleavage |
| ccp-miR319-1-3p | Cc05_g08060 | 3.0 | 12.366 | 1 | 21 | 1641 | 1660 | UUGGACUGAAGGGAGCUCCCU | AGGGAAC-CCCUUCAGUCCAA | Cleavage |
| ccp-miR319-1-3p | Cc05_g13920 | 3.0 | 14.143 | 1 | 20 | 1295 | 1314 | UUGGACUGAAGGGAGCUCCC | CAGAGCCCCCUUCAGUCCAA | Cleavage |
| ccp-miR319-1-5p | Cc00_g02180 | 2.5 | 19.299 | 1 | 20 | 795 | 814 | GAGCUUUCUUCAGUCCACUC | GGGUGCACUGAAGAAAGCUG | Cleavage |
| ccp-miR319-1-5p | Cc00_g23400 | 3.0 | 8.2 | 1 | 19 | 13 | 32 | GAGCUUUC-UUCAGUCCACU | AAUGGACUGAAAGAAAGCUC | Translation |
| ccp-miR319-1-5p | Cc01_g14910 | 3.0 | 20.116 | 1 | 20 | 3207 | 3226 | GAGCUUUCUUCAGUCCACUC | GAAUGUGCUGGAGAAAGCUC | Cleavage |
| ccp-miR319-1-5p | Cc04_g07150 | 3.0 | 24.314 | 1 | 20 | 1061 | 1080 | GAGCUUUCUUCAGUCCACUC | CAUUGGACUGAAGGGAGCUC | Cleavage |
| ccp-miR319-1-5p | Cc05_g14310 | 2.5 | 15.95 | 1 | 20 | 1011 | 1030 | GAGCUUUCUUCAGUCCACUC | GAGUGGAUUCAAGAGAGCUU | Translation |
| ccp-miR319-1-5p | Cc06_g08420 | 3.0 | 23.41 | 1 | 19 | 772 | 791 | GAGCUUUCUUC-AGUCCACU | AGUGGAUUUGGAGAAAGCUC | Cleavage |
| ccp-miR319-1-5p | Cc06_g08820 | 2.5 | 21.641 | 1 | 20 | 1464 | 1483 | GAGCUUUCUUCAGUCCACUC | UAAUGGACUGAAGAAAGUUC | Cleavage |
| ccp-miR319-1-5p | Cc08_g01030 | 3.0 | 22.227 | 1 | 20 | 81 | 99 | GAGCUUUCUUCAGUCCACUC | GAGGGGACUGA-GAAAGCUC | Translation |
| ccp-miR319-1-5p | Cc09_g04880 | 2.5 | 19.299 | 1 | 20 | 841 | 860 | GAGCUUUCUUCAGUCCACUC | GGGUGCACUGAAGAAAGCUG | Cleavage |
| ccp-miR319a-1-3p | Cc01_g07440 | 2.5 | 21.661 | 1 | 20 | 1327 | 1346 | UUGGACUGAAGGGAGCUCCC | UGGAGCUCCCUUCACUCCAA | Cleavage |
| ccp-miR319a-1-3p | Cc01_g13090 | 3.0 | 18.959 | 1 | 20 | 284 | 303 | UUGGACUGAAGGGAGCUCCC | UGGAGCUCCCUUCACUUCAA | Cleavage |
| ccp-miR319a-1-3p | Cc05_g08060 | 3.0 | 12.366 | 1 | 20 | 1642 | 1660 | UUGGACUGAAGGGAGCUCCC | GGGAAC-CCCUUCAGUCCAA | Cleavage |
| ccp-miR319a-1-3p | Cc05_g13920 | 3.0 | 14.143 | 1 | 20 | 1295 | 1314 | UUGGACUGAAGGGAGCUCCC | CAGAGCCCCCUUCAGUCCAA | Cleavage |
| ccp-miR319a-2-3p | Cc01_g07440 | 2.5 | 22.12 | 1 | 20 | 1328 | 1347 | CUUGGACUGAAGGGAGCUCC | GGAGCUCCCUUCACUCCAAA | Cleavage |
| ccp-miR319a-2-3p | Cc01_g13090 | 3.0 | 19.31 | 1 | 20 | 285 | 304 | CUUGGACUGAAGGGAGCUCC | GGAGCUCCCUUCACUUCAAA | Cleavage |
| ccp-miR319a-2-3p | Cc02_g11670 | 3.0 | 19.717 | 1 | 20 | 5 | 24 | CUUGGACUGAAGGGAGCUCC | GGAGUUCUUUCCGGUCCAAG | Translation |
| ccp-miR319a-2-3p | Cc05_g13920 | 3.0 | 13.906 | 1 | 20 | 1296 | 1315 | CUUGGACUGAAGGGAGCUCC | AGAGCCCCCUUCAGUCCAAA | Cleavage |
| ccp-miR319a-2-3p | Cc11_g14910 | 3.0 | 23.136 | 1 | 20 | 506 | 525 | CUUGGACUGAAGGGAGCUCC | GGGUCUCCCUCCAGUUCAAG | Translation |
| ccp-miR319a-2-5p | Cc04_g07150 | 3.0 | 24.314 | 1 | 20 | 1061 | 1080 | GAGCUCUCUCCAGUCCAGUC | CAUUGGACUGAAGGGAGCUC | Translation |
| ccp-miR319a-2-5p | Cc10_g09150 | 2.5 | 17.46 | 1 | 20 | 581 | 600 | GAGCUCUCUCCAGUCCAGUC | CCCUGGACUGGAGAGAGUUC | Cleavage |
| ccp-miR319a-2-5p | Cc11_g04630 | 2.5 | 15.025 | 1 | 20 | 987 | 1006 | GAGCUCUCUCCAGUCCAGUC | GAUUGGGUUGGAGAGAGCUG | Cleavage |
| ccp-miR319a-3-3p | Cc01_g07440 | 2.5 | 21.661 | 1 | 20 | 1327 | 1346 | UUGGACUGAAGGGAGCUCCC | UGGAGCUCCCUUCACUCCAA | Cleavage |
| ccp-miR319a-3-3p | Cc01_g13090 | 3.0 | 18.959 | 1 | 20 | 284 | 303 | UUGGACUGAAGGGAGCUCCC | UGGAGCUCCCUUCACUUCAA | Cleavage |
| ccp-miR319a-3-3p | Cc05_g08060 | 3.0 | 12.366 | 1 | 21 | 1641 | 1660 | UUGGACUGAAGGGAGCUCCCU | AGGGAAC-CCCUUCAGUCCAA | Cleavage |
| ccp-miR319a-3-3p | Cc05_g13920 | 3.0 | 14.143 | 1 | 20 | 1295 | 1314 | UUGGACUGAAGGGAGCUCCC | CAGAGCCCCCUUCAGUCCAA | Cleavage |
| ccp-miR319a-3-5p | Cc00_g22610 | 2.5 | 16.931 | 1 | 20 | 1590 | 1609 | AGAGCUUUCUUCAGUCCACU | AGUGGAAUGAGGGAAGUUCU | Cleavage |
| ccp-miR319a-3-5p | Cc00_g35360 | 2.5 | 17.042 | 1 | 20 | 250 | 269 | AGAGCUUUCUUCAGUCCACU | AGUGGAAUGAGGGAAGUUCU | Cleavage |
| ccp-miR319a-3-5p | Cc02_g02420 | 3.0 | 18.714 | 1 | 20 | 97 | 116 | AGAGCUUUCUUCAGUCCACU | AGACGACUGGAGAAGGCUCU | Cleavage |
| ccp-miR319a-3-5p | Cc02_g14010 | 2.5 | 22.575 | 1 | 20 | 316 | 335 | AGAGCUUUCUUCAGUCCACU | AUUGGGGUGAAGAAAGCUCU | Cleavage |
| ccp-miR319a-3-5p | Cc04_g07150 | 3.0 | 24.887 | 1 | 20 | 1062 | 1081 | AGAGCUUUCUUCAGUCCACU | AUUGGACUGAAGGGAGCUCC | Cleavage |
| ccp-miR319a-3-5p | Cc05_g14310 | 2.5 | 15.967 | 1 | 21 | 1011 | 1031 | AGAGCUUUCUUCAGUCCACUC | GAGUGGAUUCAAGAGAGCUUU | Cleavage |
| ccp-miR319a-3-5p | Cc06_g02870 | 3.0 | 16.441 | 1 | 20 | 1657 | 1676 | AGAGCUUUCUUCAGUCCACU | AGUGGACGGAAGGAGGUUUU | Cleavage |
| ccp-miR319a-3-5p | Cc06_g08820 | 2.5 | 22.655 | 1 | 20 | 1465 | 1484 | AGAGCUUUCUUCAGUCCACU | AAUGGACUGAAGAAAGUUCC | Cleavage |
| ccp-miR319a-3-5p | Cc06_g09730 | 2.5 | 21.707 | 1 | 20 | 842 | 861 | AGAGCUUUCUUCAGUCCACU | AGGGGAUUGGGGAAAGCUCU | Cleavage |
| ccp-miR319a-3-5p | Cc08_g01030 | 3.0 | 22.293 | 1 | 21 | 81 | 100 | AGAGCUUUCUUCAGUCCACUC | GAGGGGACUGA-GAAAGCUCU | Translation |
| ccp-miR319a-3-5p | Cc11_g09220 | 3.0 | 20.286 | 1 | 19 | 93 | 112 | AGAGCUUUCUUCAG-UCCAC | CUGGAGCUGAAGAAAGCUCU | Cleavage |
| ccp-miR319a-3-5p | Cc11_g17450 | 3.0 | 22.088 | 1 | 20 | 778 | 797 | AGAGCUUUCUUCAGUCCACU | AGUGGACACAAGAGAGCUUU | Cleavage |
| ccp-miR319c-1-3p | Cc02_g14410 | 2.0 | 10.332 | 1 | 20 | 937 | 956 | UUGGACUGAAGGGUUUCCUU | AGGGAACCCCUUCAGUCCAG | Cleavage |
| ccp-miR319c-1-3p | Cc02_g16220 | 3.0 | 12.795 | 1 | 20 | 211 | 230 | UUGGACUGAAGGGUUUCCUU | AAGAAAGCUCUUAAGUCCAA | Cleavage |
| ccp-miR319c-1-3p | Cc04_g10640 | 3.0 | 16.679 | 1 | 21 | 472 | 492 | UUGGACUGAAGGGUUUCCUUC | GAAGGAACCCUUUCAGUCUAC | Cleavage |
| ccp-miR319c-1-3p | Cc05_g08060 | 1.5 | 12.366 | 1 | 21 | 1640 | 1660 | UUGGACUGAAGGGUUUCCUUC | GAGGGAACCCCUUCAGUCCAA | Cleavage |
| ccp-miR319c-1-3p | Cc05_g11430 | 3.0 | 11.653 | 1 | 21 | 902 | 922 | UUGGACUGAAGGGUUUCCUUC | GAGGGGACCCCUUCAGUCCAU | Cleavage |
| ccp-miR319c-1-3p | Cc06_g03750 | 3.0 | 23.883 | 1 | 20 | 1034 | 1053 | UUGGACUGAAGGGUUUCCUU | AAGGAACUCCUUUAGUUCAG | Cleavage |
| ccp-miR319c-1-3p | Cc06_g10060 | 3.0 | 10.851 | 1 | 21 | 3784 | 3804 | UUGGACUGAAGGGUUUCCUUC | GAGAGAAGACCUUCAGUCCAA | Cleavage |
| ccp-miR319c-1-3p | Cc10_g10080 | 3.0 | 19.129 | 1 | 20 | 262 | 281 | UUGGACUGAAGGGUUUCCUU | AAGGAAAGACUGCAGUCCAA | Translation |
| ccp-miR319c-1-5p | Cc00_g08870 | 2.5 | 24.536 | 1 | 21 | 877 | 897 | AUCCAAUGAUGCAGGAGCCGG | UCUGUUCCUGGAUCAUUGGAU | Translation |
| ccp-miR319c-1-5p | Cc03_g02590 | 3.0 | 21.567 | 1 | 21 | 514 | 534 | AUCCAAUGAUGCAGGAGCCGG | CUGUCUUCUGUGUCAUUGGAU | Cleavage |
| ccp-miR319c-1-5p | Cc04_g00630 | 3.0 | 21.69 | 1 | 20 | 1903 | 1922 | AUCCAAUGAUGCAGGAGCCG | UGGCUUCUGUGUUGUUGGAU | Cleavage |
| ccp-miR319c-1-5p | Cc09_g02360 | 3.0 | 24.77 | 1 | 20 | 999 | 1018 | AUCCAAUGAUGCAGGAGCCG | CGGCUCCAGCAGUGUUGGAU | Translation |
| ccp-miR319c-2-3p | Cc01_g07440 | 2.5 | 22.12 | 1 | 20 | 1328 | 1347 | CUUGGACUGAAGGGAGCUCC | GGAGCUCCCUUCACUCCAAA | Cleavage |
| ccp-miR319c-2-3p | Cc01_g13090 | 3.0 | 19.31 | 1 | 20 | 285 | 304 | CUUGGACUGAAGGGAGCUCC | GGAGCUCCCUUCACUUCAAA | Cleavage |
| ccp-miR319c-2-3p | Cc02_g11670 | 3.0 | 19.717 | 1 | 20 | 5 | 24 | CUUGGACUGAAGGGAGCUCC | GGAGUUCUUUCCGGUCCAAG | Translation |
| ccp-miR319c-2-3p | Cc05_g13920 | 3.0 | 13.906 | 1 | 20 | 1296 | 1315 | CUUGGACUGAAGGGAGCUCC | AGAGCCCCCUUCAGUCCAAA | Cleavage |
| ccp-miR319c-2-3p | Cc11_g14910 | 3.0 | 23.136 | 1 | 20 | 506 | 525 | CUUGGACUGAAGGGAGCUCC | GGGUCUCCCUCCAGUUCAAG | Translation |
| ccp-miR319c-2-5p | Cc00_g22610 | 3.0 | 16.931 | 1 | 20 | 1590 | 1609 | AGAGCUUCCUUCAGCCCACU | AGUGGAAUGAGGGAAGUUCU | Cleavage |
| ccp-miR319c-2-5p | Cc00_g35360 | 3.0 | 17.042 | 1 | 20 | 250 | 269 | AGAGCUUCCUUCAGCCCACU | AGUGGAAUGAGGGAAGUUCU | Cleavage |
| ccp-miR319c-2-5p | Cc02_g14010 | 3.0 | 22.575 | 1 | 20 | 316 | 335 | AGAGCUUCCUUCAGCCCACU | AUUGGGGUGAAGAAAGCUCU | Cleavage |
| ccp-miR319c-2-5p | Cc05_g11560 | 3.0 | 22.595 | 1 | 20 | 1853 | 1873 | AGAGCUUCCUUCAGCC-CACU | GGUGCGGUUGAAGGGAGCUCU | Cleavage |
| ccp-miR3439-5p | Cc00_g04710 | 3.0 | 20.541 | 1 | 21 | 830 | 850 | UUGGGGUUUGGAAAUCAAGUU | GGCUUGAUCUUCAAGCCUCAA | Cleavage |
| ccp-miR3439-5p | Cc00_g09980 | 3.0 | 15.087 | 1 | 21 | 1811 | 1831 | UUGGGGUUUGGAAAUCAAGUU | AACUUCAUUUCCACACCCCAU | Cleavage |
| ccp-miR3439-5p | Cc01_g01330 | 3.0 | 19.853 | 1 | 20 | 535 | 554 | UUGGGGUUUGGAAAUCAAGU | GUUUGGUUGCCAAACCUCAA | Cleavage |
| ccp-miR3439-5p | Cc01_g01350 | 3.0 | 19.141 | 1 | 20 | 304 | 323 | UUGGGGUUUGGAAAUCAAGU | GUUUGGUUGCCAAACCUCAA | Cleavage |
| ccp-miR3439-5p | Cc02_g12010 | 1.5 | 18.28 | 1 | 21 | 1060 | 1080 | UUGGGGUUUGGAAAUCAAGUU | AGCUUGAUUUCCAAACUUCAA | Cleavage |
| ccp-miR3439-5p | Cc02_g12020 | 3.0 | 18.325 | 1 | 21 | 1717 | 1737 | UUGGGGUUUGGAAAUCAAGUU | AAAUUGAUUUCUAAGCUUCAA | Cleavage |
| ccp-miR3439-5p | Cc03_g04330 | 3.0 | 20.457 | 1 | 21 | 388 | 408 | UUGGGGUUUGGAAAUCAAGUU | AGUUUGAUUUCAAGGCCCCAA | Translation |
| ccp-miR3439-5p | Cc03_g08400 | 3.0 | 18.676 | 1 | 20 | 351 | 370 | UUGGGGUUUGGAAAUCAAGU | ACCUGAUUUUCAAGCCCUGA | Cleavage |
| ccp-miR3439-5p | Cc03_g10590 | 3.0 | 5.267 | 1 | 20 | 11 | 30 | UUGGGGUUUGGAAAUCAAGU | ACUUGAGUACUAAACCCUAA | Cleavage |
| ccp-miR3439-5p | Cc03_g10780 | 2.5 | 22.725 | 1 | 20 | 2461 | 2480 | UUGGGGUUUGGAAAUCAAGU | GCUUGUUUUCCAAACCUCAG | Cleavage |
| ccp-miR3439-5p | Cc03_g10810 | 3.0 | 19.803 | 1 | 20 | 2143 | 2162 | UUGGGGUUUGGAAAUCAAGU | AAUUGUUUUCCAAACCUCAG | Cleavage |
| ccp-miR3439-5p | Cc04_g07980 | 3.0 | 15.176 | 1 | 21 | 1214 | 1234 | UUGGGGUUUGGAAAUCAAGUU | AACUUGUUGUCCAAACUCCAG | Cleavage |
| ccp-miR3439-5p | Cc06_g11530 | 2.5 | 12.888 | 1 | 21 | 1826 | 1846 | UUGGGGUUUGGAAAUCAAGUU | AAUUUGAUUUCCAGGCUCUAA | Cleavage |
| ccp-miR3439-5p | Cc10_g02940 | 3.0 | 14.127 | 1 | 20 | 1189 | 1208 | UUGGGGUUUGGAAAUCAAGU | ACUUGUGUUCCAGACCUCAA | Cleavage |
| ccp-miR3439-5p | Cc10_g14550 | 2.5 | 6.966 | 1 | 20 | 381 | 400 | UUGGGGUUUGGAAAUCAAGU | ACUUGAUUUCCAGUUCCCAA | Cleavage |
| ccp-miR3439-5p | Cc11_g13220 | 3.0 | 12.005 | 1 | 21 | 8 | 28 | UUGGGGUUUGGAAAUCAAGUU | AAUUCGAUAUCCAAACUCCAA | Cleavage |
| ccp-miR3627-1-5p | Cc00_g11150 | 2.0 | 13.739 | 1 | 22 | 431 | 452 | UUGUCGCAGGAGAUAUGGCACU | GGUGUCAUCUCUCCUGCGACAG | Cleavage |
| ccp-miR3627-1-5p | Cc04_g14900 | 2.5 | 15.981 | 1 | 20 | 365 | 384 | UUGUCGCAGGAGAUAUGGCA | UGCCAGAUCUCCUGCGAGAA | Cleavage |
| ccp-miR3627-1-5p | Cc10_g01190 | 2.5 | 16.285 | 1 | 20 | 2619 | 2638 | UUGUCGCAGGAGAUAUGGCA | UGUCAUAUUUUCUGUGACAG | Cleavage |
| ccp-miR3627-2-3p | Cc02_g34630 | 2.5 | 22.563 | 1 | 20 | 1127 | 1146 | GGUGCCAUUCCUCCUGCGAC | UUUGCAGGAGAAAUGGCACC | Translation |
| ccp-miR3627-2-3p | Cc05_g11330 | 2.5 | 18.306 | 1 | 20 | 67 | 86 | GGUGCCAUUCCUCCUGCGAC | GUCGCCGCAGGAAUGGUACC | Cleavage |
| ccp-miR3627-2-3p | Cc06_g14560 | 2.5 | 19.363 | 1 | 21 | 1581 | 1601 | GGUGCCAUUCCUCCUGCGACA | UGUCGCAGAAGGAAUGGCAUA | Cleavage |
| ccp-miR3627-2-3p | Cc06_g20740 | 2.5 | 20.197 | 1 | 22 | 249 | 270 | GGUGCCAUUCCUCCUGCGACAC | GGGUCGCAGGAGGAAAGGCAUU | Cleavage |
| ccp-miR3627-2-5p | Cc00_g11150 | 3.0 | 13.565 | 1 | 21 | 431 | 451 | UGUCGCAGGAGCAAUGGCGCU | GGUGUCAUCUCUCCUGCGACA | Cleavage |
| ccp-miR3627-2-5p | Cc01_g05360 | 2.5 | 17.081 | 1 | 21 | 1206 | 1226 | UGUCGCAGGAGCAAUGGCGCU | GGCACCAUUGCUCUUGCGACC | Cleavage |
| ccp-miR390-3p | Cc01_g13840 | 2.0 | 19.93 | 1 | 20 | 1566 | 1584 | CGCUAUCCAUCCUGAGUUUU | AAAAC-CAGGAUGGAUAGCG | Cleavage |
| ccp-miR390-3p | Cc02_g18360 | 3.0 | 15.151 | 1 | 20 | 4647 | 4666 | CGCUAUCCAUCCUGAGUUUU | AAAAUUCAGGAUUGAUAGUC | Cleavage |
| ccp-miR390-3p | Cc03_g00490 | 2.5 | 14.349 | 1 | 20 | 732 | 751 | CGCUAUCCAUCCUGAGUUUU | AAAGCUCAGGUAGGAUAGCG | Translation |
| ccp-miR390-3p | Cc03_g09790 | 3.0 | 16.903 | 1 | 20 | 382 | 401 | CGCUAUCCAUCCUGAGUUUU | AAAAUUGAGGAUGGAAAGCG | Cleavage |
| ccp-miR390-5p | Cc00_g28220 | 3.0 | 17.256 | 1 | 20 | 473 | 492 | AAGCUCAGGAGGGAUAGCGC | GUCCUGUCCCUCCUGGGUUU | Cleavage |
| ccp-miR390-5p | Cc02_g15910 | 2.0 | 17.915 | 1 | 21 | 725 | 745 | AAGCUCAGGAGGGAUAGCGCC | GGAGCUGUCCCUUCUGAGCUU | Cleavage |
| ccp-miR390-5p | Cc11_g07430 | 3.0 | 18.161 | 1 | 21 | 922 | 942 | AAGCUCAGGAGGGAUAGCGCC | GGCACAAUUCCUUCUGAGCUU | Cleavage |
| ccp-miR390a-1-3p | Cc03_g00490 | 2.0 | 14.349 | 1 | 20 | 732 | 751 | CGCUAUCCCUCCUGAGCUUU | AAAGCUCAGGUAGGAUAGCG | Translation |
| ccp-miR390a-1-5p | Cc02_g38340 | 3.0 | 17.385 | 1 | 21 | 175 | 195 | AAACUCAGGAUGGAUAGCGCC | GGCACUUUCCAUUCUGGGUUU | Cleavage |
| ccp-miR390a-2-3p | Cc03_g04010 | 3.0 | 14.925 | 1 | 20 | 812 | 831 | CGCUAUCCCUCCUGAGCUUC | GAAGUUCAGUAGGGAUAGGG | Translation |
| ccp-miR390a-2-3p | Cc10_g03860 | 3.0 | 24.237 | 1 | 20 | 98 | 116 | CGCUAUCCCUCCUGAGCUUC | GAGGCUCAG-AGGGAUGGCG | Translation |
| ccp-miR390a-2-5p | Cc02_g15910 | 3.0 | 17.915 | 1 | 21 | 725 | 745 | AAGCUCAGGAUGGAUAGCGCU | GGAGCUGUCCCUUCUGAGCUU | Translation |
| ccp-miR390a-2-5p | Cc02_g21660 | 3.0 | 13.123 | 1 | 21 | 327 | 347 | AAGCUCAGGAUGGAUAGCGCU | AGAGUUAUCAAUUCUGAGCUU | Cleavage |
| ccp-miR393a-3p | Cc02_g21180 | 2.0 | 20.335 | 1 | 20 | 1373 | 1392 | AUCAUGCUAUCCUUUUGGAU | AUCCAAAGGGACAGUAUGAU | Translation |
| ccp-miR393a-3p | Cc05_g10710 | 3.0 | 18.425 | 1 | 21 | 568 | 588 | AUCAUGCUAUCCUUUUGGAUA | UAUCCAAGGGCAGAGCAUGAU | Translation |
| ccp-miR393a-3p | Cc07_g11260 | 2.0 | 15.905 | 1 | 20 | 3358 | 3377 | AUCAUGCUAUCCUUUUGGAU | UUCCAAAGGGAUAGCGUGAU | Cleavage |
| ccp-miR393a-3p | Cc10_g09700 | 2.5 | 15.13 | 1 | 20 | 3939 | 3958 | AUCAUGCUAUCCUUUUGGAU | AUCAAAAAGGUUGGCAUGAU | Translation |
| ccp-miR393a-5p | Cc00_g10200 | 3.0 | 21.354 | 1 | 21 | 1122 | 1141 | CAAAGGGAUCGCAUUGAUCCU | AGGAUCAGUG-GAUCUCUUUG | Translation |
| ccp-miR393a-5p | Cc01_g20300 | 2.5 | 20.976 | 1 | 21 | 1825 | 1845 | CAAAGGGAUCGCAUUGAUCCU | GGAGACAAUGCGAUCCCUUUG | Cleavage |
| ccp-miR393a-5p | Cc02_g02180 | 3.0 | 18.791 | 1 | 21 | 915 | 935 | CAAAGGGAUCGCAUUGAUCCU | AGGAUCAAGUUGAUUCCUUUG | Cleavage |
| ccp-miR393a-5p | Cc03_g14240 | 3.0 | 21.15 | 1 | 20 | 85 | 104 | CAAAGGGAUCGCAUUGAUCC | GGAUGGAUGCGGUUCUUUUG | Cleavage |
| ccp-miR393a-5p | Cc07_g01170 | 2.0 | 18.926 | 1 | 20 | 1818 | 1837 | CAAAGGGAUCGCAUUGAUCC | GAAACAAUGCGAUCCCUUUG | Cleavage |
| ccp-miR393a-5p | Cc08_g03640 | 3.0 | 21.15 | 1 | 20 | 85 | 104 | CAAAGGGAUCGCAUUGAUCC | GGAUGGAUGCGGUUCUUUUG | Cleavage |
| ccp-miR393a-5p | Cc08_g03650 | 3.0 | 21.96 | 1 | 20 | 73 | 92 | CAAAGGGAUCGCAUUGAUCC | GGAUGGAUGCGGUUCUUUUG | Cleavage |
| ccp-miR393a-5p | Cc08_g10680 | 3.0 | 19.281 | 1 | 20 | 430 | 449 | CAAAGGGAUCGCAUUGAUCC | GGAUUGAUGGGGUUCCUUUG | Translation |
| ccp-miR393b-3p | Cc01_g00350 | 3.0 | 23.761 | 1 | 20 | 1231 | 1250 | UCAAUGCGAUCCCUUUGGAU | AUCACAAAGGAUCGCAUUGA | Cleavage |
| ccp-miR393b-3p | Cc02_g07080 | 0.0 | 16.619 | 1 | 21 | 310 | 330 | UCAAUGCGAUCCCUUUGGAUG | CAUCCAAAGGGAUCGCAUUGA | Cleavage |
| ccp-miR393b-3p | Cc02_g31040 | 3.0 | 16.15 | 1 | 21 | 1132 | 1152 | UCAAUGCGAUCCCUUUGGAUG | UAUACCAAUGGAUCGCAUUGA | Cleavage |
| ccp-miR393b-3p | Cc02_g38230 | 3.0 | 9.63 | 1 | 20 | 1680 | 1699 | UCAAUGCGAUCCCUUUGGAU | AUUGAAAGGGAACGCAUUGG | Translation |
| ccp-miR393b-3p | Cc07_g09400 | 3.0 | 21.791 | 1 | 20 | 203 | 222 | UCAAUGCGAUCCCUUUGGAU | AGCCAAGGCGAUUGCAUUGA | Cleavage |
| ccp-miR393b-3p | Cc10_g16100 | 3.0 | 22.448 | 1 | 20 | 839 | 858 | UCAAUGCGAUCCCUUUGGAU | UUUCACAGGGAUUGCAUUGA | Cleavage |
| ccp-miR393b-3p | Cc11_g13330 | 3.0 | 18.996 | 1 | 20 | 1259 | 1279 | UCAAUGCGA-UCCCUUUGGAU | AUCCAAAGGUACUCGCAUUGA | Translation |
| ccp-miR394-3p | Cc03_g04140 | 3.0 | 18.054 | 1 | 20 | 5333 | 5352 | AGGUGGGCAUACUGCCAACU | AGAUGGCAGGAUGCCCGUCU | Translation |
| ccp-miR394-3p | Cc08_g04140 | 3.0 | 16.264 | 1 | 20 | 1545 | 1564 | AGGUGGGCAUACUGCCAACU | AUUUGGCAGUAUCCUCACUU | Cleavage |
| ccp-miR394-5p | Cc04_g00510 | 3.0 | 18.58 | 1 | 20 | 2561 | 2580 | UUGGCAUUCUGUCCACCUCC | AGAGGUGGACGGAAUGCUAU | Cleavage |
| ccp-miR394-5p | Cc07_g17350 | 2.5 | 17.058 | 1 | 22 | 2148 | 2169 | UUGGCAUUCUGUCCACCUCCAU | AAGGAGUUGAACAGAAUGCCGA | Cleavage |
| ccp-miR394-5p | Cc11_g17010 | 1.0 | 14.886 | 1 | 22 | 1231 | 1252 | UUGGCAUUCUGUCCACCUCCAU | AAGGAGGUUGACAGAAUGCCAA | Cleavage |
| ccp-miR394a-5p | Cc06_g08230 | 3.0 | 19.627 | 1 | 22 | 1425 | 1446 | UUGGCAUUCUGUCGACCUUCAU | GUGAAGGCCGUCAGAAUGUCGA | Cleavage |
| ccp-miR394a-5p | Cc11_g17010 | 1.0 | 14.886 | 1 | 22 | 1231 | 1252 | UUGGCAUUCUGUCGACCUUCAU | AAGGAGGUUGACAGAAUGCCAA | Cleavage |
| ccp-miR395a-1-3p | Cc00_g25390 | 2.5 | 16.964 | 1 | 22 | 433 | 454 | CUGAAGUGUUUGGGGGAACUCC | GUAGUUCCCCCACAAACUUCGG | Translation |
| ccp-miR395a-1-3p | Cc00_g31260 | 3.0 | 18.087 | 1 | 22 | 376 | 397 | CUGAAGUGUUUGGGGGAACUCC | GUAGUUCCUCCACAAACUUCGG | Translation |
| ccp-miR395a-1-3p | Cc02_g05220 | 3.0 | 18.512 | 1 | 20 | 1386 | 1405 | CUGAAGUGUUUGGGGGAACU | AGUAUCCACAAAUACUUCAG | Cleavage |
| ccp-miR395a-1-3p | Cc02_g08440 | 2.5 | 18.718 | 1 | 22 | 436 | 457 | CUGAAGUGUUUGGGGGAACUCC | GUAGUUCCCCCACAAACUUCGG | Translation |
| ccp-miR395a-1-3p | Cc02_g09970 | 3.0 | 14.724 | 1 | 22 | 1287 | 1308 | CUGAAGUGUUUGGGGGAACUCC | GAAGUUCACCCAAGCAGUUCAG | Cleavage |
| ccp-miR395a-1-3p | Cc02_g31660 | 3.0 | 14.54 | 1 | 20 | 1005 | 1024 | CUGAAGUGUUUGGGGGAACU | AGUUUUCUUAAAGACUUCAG | Cleavage |
| ccp-miR395a-1-3p | Cc03_g04380 | 3.0 | 17.672 | 1 | 21 | 679 | 699 | CUGAAGUGUUUGGGGGAACUC | GAGUUCCUCCAAACUCUUCAU | Cleavage |
| ccp-miR395a-1-3p | Cc05_g07700 | 3.0 | 12.874 | 1 | 21 | 958 | 978 | CUGAAGUGUUUGGGGGAACUC | GAAUUUCCUCGAGCACUUCAG | Cleavage |
| ccp-miR395a-1-3p | Cc06_g20520 | 3.0 | 11.723 | 1 | 20 | 1669 | 1688 | CUGAAGUGUUUGGGGGAACU | AGUUCCCCCAAAUACCUCAA | Cleavage |
| ccp-miR395a-1-3p | Cc07_g14190 | 3.0 | 23.12 | 1 | 22 | 1228 | 1249 | CUGAAGUGUUUGGGGGAACUCC | GCAGUUGCUCCAAUUACUUCAG | Translation |
| ccp-miR395a-1-3p | Cc07_g15540 | 2.5 | 18.152 | 1 | 20 | 104 | 123 | CUGAAGUGUUUGGGGGAACU | AGUUCCCCCAAACUCUUCAA | Cleavage |
| ccp-miR395a-1-3p | Cc08_g01790 | 3.0 | 18.934 | 1 | 21 | 1236 | 1256 | CUGAAGUGUUUGGGGGAACUC | GAGUUCUCUCAAUUAUUUCAG | Translation |
| ccp-miR395a-1-3p | Cc10_g10360 | 2.5 | 10.599 | 1 | 21 | 1942 | 1962 | CUGAAGUGUUUGGGGGAACUC | GAAUUCUUCCAAACAUUUCAG | Cleavage |
| ccp-miR395a-2-3p | Cc00_g25390 | 2.5 | 16.964 | 1 | 22 | 433 | 454 | CUGAAGUGUUUGGGGGAACUCC | GUAGUUCCCCCACAAACUUCGG | Translation |
| ccp-miR395a-2-3p | Cc00_g31260 | 3.0 | 18.087 | 1 | 22 | 376 | 397 | CUGAAGUGUUUGGGGGAACUCC | GUAGUUCCUCCACAAACUUCGG | Translation |
| ccp-miR395a-2-3p | Cc02_g05220 | 3.0 | 18.512 | 1 | 20 | 1386 | 1405 | CUGAAGUGUUUGGGGGAACU | AGUAUCCACAAAUACUUCAG | Cleavage |
| ccp-miR395a-2-3p | Cc02_g08440 | 2.5 | 18.718 | 1 | 22 | 436 | 457 | CUGAAGUGUUUGGGGGAACUCC | GUAGUUCCCCCACAAACUUCGG | Translation |
| ccp-miR395a-2-3p | Cc02_g09970 | 3.0 | 14.724 | 1 | 22 | 1287 | 1308 | CUGAAGUGUUUGGGGGAACUCC | GAAGUUCACCCAAGCAGUUCAG | Cleavage |
| ccp-miR395a-2-3p | Cc02_g31660 | 3.0 | 14.54 | 1 | 20 | 1005 | 1024 | CUGAAGUGUUUGGGGGAACU | AGUUUUCUUAAAGACUUCAG | Cleavage |
| ccp-miR395a-2-3p | Cc03_g04380 | 3.0 | 17.672 | 1 | 21 | 679 | 699 | CUGAAGUGUUUGGGGGAACUC | GAGUUCCUCCAAACUCUUCAU | Cleavage |
| ccp-miR395a-2-3p | Cc05_g07700 | 3.0 | 12.874 | 1 | 21 | 958 | 978 | CUGAAGUGUUUGGGGGAACUC | GAAUUUCCUCGAGCACUUCAG | Cleavage |
| ccp-miR395a-2-3p | Cc06_g20520 | 3.0 | 11.723 | 1 | 20 | 1669 | 1688 | CUGAAGUGUUUGGGGGAACU | AGUUCCCCCAAAUACCUCAA | Cleavage |
| ccp-miR395a-2-3p | Cc07_g14190 | 3.0 | 23.12 | 1 | 22 | 1228 | 1249 | CUGAAGUGUUUGGGGGAACUCC | GCAGUUGCUCCAAUUACUUCAG | Translation |
| ccp-miR395a-2-3p | Cc07_g15540 | 2.5 | 18.152 | 1 | 20 | 104 | 123 | CUGAAGUGUUUGGGGGAACU | AGUUCCCCCAAACUCUUCAA | Cleavage |
| ccp-miR395a-2-3p | Cc08_g01790 | 3.0 | 18.934 | 1 | 21 | 1236 | 1256 | CUGAAGUGUUUGGGGGAACUC | GAGUUCUCUCAAUUAUUUCAG | Translation |
| ccp-miR395a-2-3p | Cc10_g10360 | 2.5 | 10.599 | 1 | 21 | 1942 | 1962 | CUGAAGUGUUUGGGGGAACUC | GAAUUCUUCCAAACAUUUCAG | Cleavage |
| ccp-miR395a-3-3p | Cc00_g25390 | 2.5 | 16.964 | 1 | 22 | 433 | 454 | CUGAAGUGUUUGGGGGAACUCC | GUAGUUCCCCCACAAACUUCGG | Translation |
| ccp-miR395a-3-3p | Cc00_g31260 | 3.0 | 18.087 | 1 | 22 | 376 | 397 | CUGAAGUGUUUGGGGGAACUCC | GUAGUUCCUCCACAAACUUCGG | Translation |
| ccp-miR395a-3-3p | Cc02_g05220 | 3.0 | 18.512 | 1 | 20 | 1386 | 1405 | CUGAAGUGUUUGGGGGAACU | AGUAUCCACAAAUACUUCAG | Cleavage |
| ccp-miR395a-3-3p | Cc02_g08440 | 2.5 | 18.718 | 1 | 22 | 436 | 457 | CUGAAGUGUUUGGGGGAACUCC | GUAGUUCCCCCACAAACUUCGG | Translation |
| ccp-miR395a-3-3p | Cc02_g09970 | 3.0 | 14.724 | 1 | 22 | 1287 | 1308 | CUGAAGUGUUUGGGGGAACUCC | GAAGUUCACCCAAGCAGUUCAG | Cleavage |
| ccp-miR395a-3-3p | Cc02_g31660 | 3.0 | 14.54 | 1 | 20 | 1005 | 1024 | CUGAAGUGUUUGGGGGAACU | AGUUUUCUUAAAGACUUCAG | Cleavage |
| ccp-miR395a-3-3p | Cc03_g04380 | 3.0 | 17.672 | 1 | 21 | 679 | 699 | CUGAAGUGUUUGGGGGAACUC | GAGUUCCUCCAAACUCUUCAU | Cleavage |
| ccp-miR395a-3-3p | Cc05_g07700 | 3.0 | 12.874 | 1 | 21 | 958 | 978 | CUGAAGUGUUUGGGGGAACUC | GAAUUUCCUCGAGCACUUCAG | Cleavage |
| ccp-miR395a-3-3p | Cc06_g20520 | 3.0 | 11.723 | 1 | 20 | 1669 | 1688 | CUGAAGUGUUUGGGGGAACU | AGUUCCCCCAAAUACCUCAA | Cleavage |
| ccp-miR395a-3-3p | Cc07_g14190 | 3.0 | 23.12 | 1 | 22 | 1228 | 1249 | CUGAAGUGUUUGGGGGAACUCC | GCAGUUGCUCCAAUUACUUCAG | Translation |
| ccp-miR395a-3-3p | Cc07_g15540 | 2.5 | 18.152 | 1 | 20 | 104 | 123 | CUGAAGUGUUUGGGGGAACU | AGUUCCCCCAAACUCUUCAA | Cleavage |
| ccp-miR395a-3-3p | Cc08_g01790 | 3.0 | 18.934 | 1 | 21 | 1236 | 1256 | CUGAAGUGUUUGGGGGAACUC | GAGUUCUCUCAAUUAUUUCAG | Translation |
| ccp-miR395a-3-3p | Cc10_g10360 | 2.5 | 10.599 | 1 | 21 | 1942 | 1962 | CUGAAGUGUUUGGGGGAACUC | GAAUUCUUCCAAACAUUUCAG | Cleavage |
| ccp-miR395b-1-3p | Cc02_g08010 | 3.0 | 15.446 | 1 | 20 | 1490 | 1509 | GUGAAGUGUUUGGGGGAACU | AUUUCCCUCCAACACUUCGC | Translation |
| ccp-miR395b-1-3p | Cc03_g04380 | 2.5 | 17.672 | 1 | 21 | 679 | 699 | GUGAAGUGUUUGGGGGAACUC | GAGUUCCUCCAAACUCUUCAU | Cleavage |
| ccp-miR395b-1-3p | Cc04_g07660 | 3.0 | 13.668 | 1 | 20 | 730 | 749 | GUGAAGUGUUUGGGGGAACU | AGCUCUCCCAAUCAUUUCAC | Translation |
| ccp-miR395b-1-3p | Cc06_g09280 | 3.0 | 3.016 | 1 | 20 | 183 | 202 | GUGAAGUGUUUGGGGGAACU | AAUUUCUCCAAAAACUUCAC | Cleavage |
| ccp-miR395b-1-3p | Cc06_g20520 | 3.0 | 11.723 | 1 | 20 | 1669 | 1688 | GUGAAGUGUUUGGGGGAACU | AGUUCCCCCAAAUACCUCAA | Cleavage |
| ccp-miR395b-1-3p | Cc07_g01480 | 3.0 | 7.463 | 1 | 20 | 979 | 998 | GUGAAGUGUUUGGGGGAACU | CGUUCCCUCCAACACUUUAC | Translation |
| ccp-miR395b-1-3p | Cc07_g15540 | 2.5 | 18.152 | 1 | 20 | 104 | 123 | GUGAAGUGUUUGGGGGAACU | AGUUCCCCCAAACUCUUCAA | Cleavage |
| ccp-miR395b-2-3p | Cc02_g08010 | 2.5 | 15.446 | 1 | 20 | 1490 | 1509 | GUGAAGUGUUCGAGGGAACU | AUUUCCCUCCAACACUUCGC | Translation |
| ccp-miR395b-2-3p | Cc02_g16670 | 3.0 | 17.137 | 1 | 20 | 698 | 717 | GUGAAGUGUUCGAGGGAACU | GGUUCUUUCGGACAUUUUAC | Cleavage |
| ccp-miR395b-2-3p | Cc04_g16610 | 3.0 | 11.032 | 1 | 20 | 808 | 827 | GUGAAGUGUUCGAGGGAACU | AGUGCCCUUGAAUACUUCGU | Cleavage |
| ccp-miR395b-2-3p | Cc05_g07700 | 3.0 | 12.874 | 1 | 21 | 958 | 978 | GUGAAGUGUUCGAGGGAACUC | GAAUUUCCUCGAGCACUUCAG | Cleavage |
| ccp-miR395b-2-3p | Cc07_g01480 | 2.5 | 7.463 | 1 | 20 | 979 | 998 | GUGAAGUGUUCGAGGGAACU | CGUUCCCUCCAACACUUUAC | Translation |
| ccp-miR395h-5p | Cc01_g11480 | 3.0 | 11.378 | 1 | 21 | 1678 | 1698 | AUGAAGUGUUUUGGAGUACUU | AAGUACUCCAAGGCACGUUAU | Cleavage |
| ccp-miR395h-5p | Cc01_g11510 | 3.0 | 11.908 | 1 | 21 | 829 | 849 | AUGAAGUGUUUUGGAGUACUU | AAGUACUCCAAGGCACGUUAU | Cleavage |
| ccp-miR395h-5p | Cc02_g30790 | 3.0 | 13.847 | 1 | 20 | 1741 | 1760 | AUGAAGUGUUUUGGAGUACU | AGUAUUCCAGAAUGCUUCAG | Cleavage |
| ccp-miR395h-5p | Cc07_g15110 | 3.0 | 14.459 | 1 | 21 | 583 | 603 | AUGAAGUGUUUUGGAGUACUU | GAUUGCUCCAAAACGCUUUGU | Cleavage |
| ccp-miR395h-5p | Cc07_g19270 | 2.5 | 14.546 | 1 | 21 | 520 | 541 | AUGAAGU-GUUUUGGAGUACUU | ACGUACUCCAAAACCACUUUAU | Cleavage |
| ccp-miR395t-1-3p | Cc00_g07040 | 3.0 | 17.866 | 1 | 20 | 537 | 556 | GUGAAGUGUUUUGGGAAGCU | AGCUUCUUGAGACAUUUUAC | Cleavage |
| ccp-miR395t-1-3p | Cc01_g18570 | 2.0 | 10.741 | 1 | 20 | 2164 | 2183 | GUGAAGUGUUUUGGGAAGCU | GGCAUCCCAAAACAUUUCAC | Cleavage |
| ccp-miR395t-1-3p | Cc02_g00070 | 3.0 | 20.099 | 1 | 20 | 777 | 796 | GUGAAGUGUUUUGGGAAGCU | AGCUUCUUGAGAUACUUCAU | Cleavage |
| ccp-miR395t-1-3p | Cc03_g03340 | 3.0 | 13.45 | 1 | 20 | 170 | 189 | GUGAAGUGUUUUGGGAAGCU | CGCUUUCCAGGGCACUUCAC | Cleavage |
| ccp-miR395t-1-3p | Cc06_g06510 | 2.5 | 12.531 | 1 | 20 | 371 | 390 | GUGAAGUGUUUUGGGAAGCU | AGGUUCACAAAACAUUUCAC | Cleavage |
| ccp-miR395t-1-3p | Cc06_g09450 | 3.0 | 15.119 | 1 | 20 | 1523 | 1542 | GUGAAGUGUUUUGGGAAGCU | AGCUUUCCCAAAUGUUUCAC | Cleavage |
| ccp-miR395t-1-3p | Cc10_g03890 | 3.0 | 20.604 | 1 | 20 | 1247 | 1266 | GUGAAGUGUUUUGGGAAGCU | UGCUGCCCAGAACACUUCAU | Cleavage |
| ccp-miR395t-1-3p | Cc11_g02760 | 2.5 | 6.808 | 1 | 20 | 63 | 82 | GUGAAGUGUUUUGGGAAGCU | UGCUUCUCAAAAUGCUUCAC | Cleavage |
| ccp-miR395t-2-3p | Cc03_g05030 | 3.0 | 14.317 | 1 | 20 | 1042 | 1061 | GUGAAGUGUUUGGGGAAACU | AGUUUCCUCAGAAAUUUCAU | Cleavage |
| ccp-miR395t-2-3p | Cc06_g09280 | 2.5 | 3.016 | 1 | 20 | 183 | 202 | GUGAAGUGUUUGGGGAAACU | AAUUUCUCCAAAAACUUCAC | Cleavage |
| ccp-miR395t-2-3p | Cc06_g09450 | 3.0 | 15.119 | 1 | 20 | 1523 | 1542 | GUGAAGUGUUUGGGGAAACU | AGCUUUCCCAAAUGUUUCAC | Cleavage |
| ccp-miR395x-5p | Cc00_g07040 | 3.0 | 17.866 | 1 | 20 | 537 | 556 | GUGAAGUGUUUGGAGAAGCU | AGCUUCUUGAGACAUUUUAC | Cleavage |
| ccp-miR395x-5p | Cc01_g10450 | 3.0 | 16.376 | 1 | 21 | 1316 | 1336 | GUGAAGUGUUUGGAGAAGCUA | UAGCUGCUCCUAAUACUUCAU | Translation |
| ccp-miR395x-5p | Cc02_g00070 | 3.0 | 20.099 | 1 | 20 | 777 | 796 | GUGAAGUGUUUGGAGAAGCU | AGCUUCUUGAGAUACUUCAU | Cleavage |
| ccp-miR395x-5p | Cc02_g14970 | 3.0 | 8.389 | 1 | 20 | 81 | 100 | GUGAAGUGUUUGGAGAAGCU | AGCUGCUCCAAUCGUUUCAC | Translation |
| ccp-miR395x-5p | Cc02_g31850 | 2.0 | 6.278 | 1 | 20 | 237 | 256 | GUGAAGUGUUUGGAGAAGCU | AGCUUCUUCAAGAACUUCAC | Cleavage |
| ccp-miR395x-5p | Cc06_g09280 | 2.5 | 3.016 | 1 | 20 | 183 | 202 | GUGAAGUGUUUGGAGAAGCU | AAUUUCUCCAAAAACUUCAC | Cleavage |
| ccp-miR395x-5p | Cc06_g09450 | 3.0 | 15.119 | 1 | 20 | 1523 | 1542 | GUGAAGUGUUUGGAGAAGCU | AGCUUUCCCAAAUGUUUCAC | Cleavage |
| ccp-miR395x-5p | Cc07_g02770 | 3.0 | 17.156 | 1 | 20 | 646 | 665 | GUGAAGUGUUUGGAGAAGCU | AACUUCACCAAAUAUUUCAC | Cleavage |
| ccp-miR395x-5p | Cc07_g11500 | 2.0 | 17.693 | 1 | 21 | 982 | 1002 | GUGAAGUGUUUGGAGAAGCUA | UGGCUGCUCCAAACAUUUCAC | Cleavage |
| ccp-miR395x-5p | Cc08_g12840 | 2.5 | 1.261 | 1 | 20 | 176 | 195 | GUGAAGUGUUUGGAGAAGCU | AGCUUCUCUUAAAACUUCAC | Translation |
| ccp-miR395x-5p | Cc11_g02760 | 3.0 | 6.808 | 1 | 20 | 63 | 82 | GUGAAGUGUUUGGAGAAGCU | UGCUUCUCAAAAUGCUUCAC | Cleavage |
| ccp-miR396-1-3p | Cc02_g18640 | 3.0 | 21.145 | 1 | 20 | 1334 | 1353 | GUUCAAGAAAGCUGUGGAAA | UUGCCAUGGCUUUUUUGGAC | Cleavage |
| ccp-miR396-1-3p | Cc02_g19710 | 2.5 | 14.421 | 1 | 21 | 161 | 181 | GUUCAAGAAAGCUGUGGAAAA | UUUUCCAUAGCUUUCUUGGUC | Cleavage |
| ccp-miR396-1-3p | Cc02_g31140 | 3.0 | 11.697 | 1 | 20 | 155 | 173 | GUUCAAGAAAGCUGUGGAAA | UUUCCGUA-CUUUCUUGAAC | Cleavage |
| ccp-miR396-1-3p | Cc03_g09150 | 3.0 | 10.598 | 1 | 20 | 133 | 152 | GUUCAAGAAAGCUGUGGAAA | CCUUCACAGCUUUCUUGAGC | Cleavage |
| ccp-miR396-1-3p | Cc03_g12030 | 3.0 | 11.242 | 1 | 20 | 1182 | 1201 | GUUCAAGAAAGCUGUGGAAA | UUUUCAUAGCUCCCUUGAAC | Translation |
| ccp-miR396-1-3p | Cc06_g08100 | 0.0 | 10.294 | 1 | 21 | 303 | 323 | GUUCAAGAAAGCUGUGGAAAA | UUUUCCACAGCUUUCUUGAAC | Cleavage |
| ccp-miR396-1-3p | Cc07_g19350 | 3.0 | 9.503 | 1 | 21 | 188 | 208 | GUUCAAGAAAGCUGUGGAAAA | UUUUCUGUAGCUUCCUUGAAU | Cleavage |
| ccp-miR396-1-3p | Cc10_g11850 | 3.0 | 12.132 | 1 | 20 | 328 | 347 | GUUCAAGAAAGCUGUGGAAA | UUUUUAUGACUUUCUUGAAC | Cleavage |
| ccp-miR396-1-5p | Cc01_g13810 | 3.0 | 17.886 | 1 | 21 | 949 | 969 | UCCCACAGCUUCCUUGAACUU | AAGUUCAAGGAUGUUGUGAGA | Translation |
| ccp-miR396-1-5p | Cc01_g18610 | 3.0 | 21.987 | 1 | 20 | 811 | 830 | UCCCACAGCUUCCUUGAACU | AGCUCAAGGAAGCUGUGCGG | Cleavage |
| ccp-miR396-1-5p | Cc02_g21130 | 3.0 | 14.115 | 1 | 21 | 877 | 897 | UCCCACAGCUUCCUUGAACUU | AAUUUCAUGGAAGAUGUGGGA | Cleavage |
| ccp-miR396-1-5p | Cc04_g00510 | 2.5 | 17.828 | 1 | 20 | 277 | 296 | UCCCACAGCUUCCUUGAACU | AGUUCAAGGAAACUGUGGAA | Translation |
| ccp-miR396-1-5p | Cc05_g15560 | 3.0 | 24.239 | 1 | 20 | 1534 | 1553 | UCCCACAGCUUCCUUGAACU | ACUUUAACGAAGUUGUGGGA | Cleavage |
| ccp-miR396-1-5p | Cc06_g08100 | 0.0 | 21.546 | 1 | 21 | 403 | 423 | UCCCACAGCUUCCUUGAACUU | AAGUUCAAGGAAGCUGUGGGA | Cleavage |
| ccp-miR396-1-5p | Cc08_g14860 | 2.5 | 19.661 | 1 | 21 | 1118 | 1138 | UCCCACAGCUUCCUUGAACUU | AAGUUCGUGGAGGUUGUGGGA | Cleavage |
| ccp-miR396-1-5p | Cc10_g07110 | 2.5 | 16.753 | 1 | 21 | 954 | 974 | UCCCACAGCUUCCUUGAACUU | GAGGUUAAGGAAUCUGUGGGA | Translation |
| ccp-miR396a-3p | Cc01_g03960 | 2.0 | 14.177 | 1 | 20 | 638 | 657 | GUUCAAUAAAGCUGUGGGAU | AUCCUACAGUUUUGUUGAAU | Cleavage |
| ccp-miR396a-3p | Cc06_g08100 | 3.0 | 10.294 | 1 | 20 | 304 | 323 | GUUCAAUAAAGCUGUGGGAU | UUUCCACAGCUUUCUUGAAC | Cleavage |
| ccp-miR396a-3p | Cc09_g05830 | 3.0 | 19.119 | 1 | 20 | 3252 | 3271 | GUUCAAUAAAGCUGUGGGAU | AUCUCGUGGUUUUGUUGAAC | Cleavage |
| ccp-miR396a-3p | Cc11_g05300 | 2.5 | 20.623 | 1 | 20 | 652 | 671 | GUUCAAUAAAGCUGUGGGAU | UUCCCACAGCCUUAUUGGAC | Translation |
| ccp-miR396a-5p | Cc00_g15760 | 3.0 | 19.582 | 1 | 21 | 141 | 161 | UUCCACAGCUUUCUUGAACUG | CAGUUGAAGAAAGUUCUGGAA | Cleavage |
| ccp-miR396a-5p | Cc02_g00930 | 2.0 | 16.902 | 1 | 21 | 825 | 846 | UUCCACA-GCUUUCUUGAACUG | CCGUUCAAGAAAGCCUGUGGAA | Cleavage |
| ccp-miR396a-5p | Cc02_g13260 | 3.0 | 21.19 | 1 | 20 | 1196 | 1215 | UUCCACAGCUUUCUUGAACU | AGAUGGAGAAGGCUGUGGAA | Cleavage |
| ccp-miR396a-5p | Cc02_g36500 | 2.0 | 18.051 | 1 | 19 | 1027 | 1046 | UUCCACA-GCUUUCUUGAAC | GUUCAAGAAAGCAUGUGGAA | Cleavage |
| ccp-miR396a-5p | Cc03_g04410 | 3.0 | 20.285 | 1 | 21 | 1596 | 1617 | UUCCACAGCUUUCUUGA-ACUG | CGGUGUGAAGAAAGCUGUGGAA | Cleavage |
| ccp-miR396a-5p | Cc03_g04610 | 2.5 | 14.7 | 1 | 20 | 263 | 282 | UUCCACAGCUUUCUUGAACU | AGUUUAAGAAGGCUGAGGAA | Cleavage |
| ccp-miR396a-5p | Cc04_g00510 | 1.5 | 17.828 | 1 | 21 | 276 | 296 | UUCCACAGCUUUCUUGAACUG | UAGUUCAAGGAAACUGUGGAA | Translation |
| ccp-miR396a-5p | Cc05_g03020 | 3.0 | 22.758 | 1 | 21 | 1983 | 2004 | UUCCACAGCUUUCUUGA-ACUG | CAGUGUGAAGAAAGCUGUGGAA | Cleavage |
| ccp-miR396a-5p | Cc05_g16590 | 3.0 | 20.196 | 1 | 20 | 1386 | 1405 | UUCCACAGCUUUCUUGAACU | AGUGGGAGGAAGCUGUGGAA | Cleavage |
| ccp-miR396a-5p | Cc06_g05390 | 2.0 | 17.417 | 1 | 21 | 783 | 804 | UUCCACA-GCUUUCUUGAACUG | CCGUUCAAGAAAGCCUGUGGAA | Cleavage |
| ccp-miR396a-5p | Cc06_g08100 | 1.0 | 21.546 | 1 | 20 | 404 | 423 | UUCCACAGCUUUCUUGAACU | AGUUCAAGGAAGCUGUGGGA | Cleavage |
| ccp-miR396a-5p | Cc06_g12040 | 2.0 | 12.48 | 1 | 21 | 665 | 686 | UUCCACA-GCUUUCUUGAACUG | CCGUUCAAGAAAGCCUGUGGAA | Cleavage |
| ccp-miR396a-5p | Cc07_g07350 | 2.0 | 24.972 | 1 | 19 | 1021 | 1040 | UUCCACA-GCUUUCUUGAAC | GUUCAAGAAAGCCUGUGGAA | Cleavage |
| ccp-miR396a-5p | Cc07_g09590 | 3.0 | 16.001 | 1 | 20 | 764 | 783 | UUCCACAGCUUUCUUGAACU | AGAUCAAGAGAUCUGUGGGA | Translation |
| ccp-miR396a-5p | Cc07_g20540 | 3.0 | 16.474 | 1 | 20 | 2081 | 2100 | UUCCACAGCUUUCUUGAACU | AGUUUAAUAAAGCUGUUGAA | Cleavage |
| ccp-miR396a-5p | Cc08_g05740 | 3.0 | 8.329 | 1 | 20 | 1727 | 1746 | UUCCACAGCUUUCUUGAACU | AGUUUGAGAAAGUUGUGAAA | Cleavage |
| ccp-miR396a-5p | Cc09_g08870 | 3.0 | 20.803 | 1 | 20 | 131 | 150 | UUCCACAGCUUUCUUGAACU | UGUUCAAGAAGGCUGCGGAA | Cleavage |
| ccp-miR396a-5p | Cc09_g09000 | 2.5 | 15.835 | 1 | 20 | 320 | 339 | UUCCACAGCUUUCUUGAACU | UGUUCAAGAAAGCUGAGGAA | Cleavage |
| ccp-miR396a-5p | Cc09_g10660 | 3.0 | 16.068 | 1 | 20 | 1109 | 1128 | UUCCACAGCUUUCUUGAACU | AGUACAAGAAGGAUGUGGAG | Cleavage |
| ccp-miR396a-5p | Cc11_g03060 | 3.0 | 24.133 | 1 | 21 | 657 | 678 | UUCCACAGCUUUCUUGA-ACUG | CGGUGUGAAGAAAGCUGUGGAA | Cleavage |
| ccp-miR396a-5p | Cc11_g10740 | 3.0 | 19.787 | 1 | 20 | 221 | 240 | UUCCACAGCUUUCUUGAACU | AGUUCAAUGAGGUUGUGGAG | Cleavage |
| ccp-miR396a-5p | Cc11_g11830 | 3.0 | 19.658 | 1 | 21 | 1289 | 1309 | UUCCACAGCUUUCUUGAACUG | CAGUUCAAUAAUGCUGUGGAC | Translation |
| ccp-miR396b-1-3p | Cc03_g01960 | 3.0 | 12.372 | 1 | 21 | 132 | 152 | GCUCAAGAAAGCUGCGGGAAA | UUUCUUGCAAUUUUUUUGAGC | Cleavage |
| ccp-miR396b-1-3p | Cc05_g09860 | 3.0 | 12.24 | 1 | 20 | 758 | 777 | GCUCAAGAAAGCUGCGGGAA | UUCUAUCAGUUUUCUUGAGC | Cleavage |
| ccp-miR396b-1-3p | Cc06_g08100 | 3.0 | 10.294 | 1 | 21 | 303 | 323 | GCUCAAGAAAGCUGCGGGAAA | UUUUCCACAGCUUUCUUGAAC | Cleavage |
| ccp-miR396b-1-5p | Cc00_g15760 | 3.0 | 19.582 | 1 | 20 | 142 | 161 | UUCCACAGCUUUCUUGAACU | AGUUGAAGAAAGUUCUGGAA | Cleavage |
| ccp-miR396b-1-5p | Cc02_g00930 | 2.0 | 16.902 | 1 | 19 | 827 | 846 | UUCCACA-GCUUUCUUGAAC | GUUCAAGAAAGCCUGUGGAA | Cleavage |
| ccp-miR396b-1-5p | Cc02_g13260 | 3.0 | 21.19 | 1 | 21 | 1195 | 1215 | UUCCACAGCUUUCUUGAACUU | GAGAUGGAGAAGGCUGUGGAA | Cleavage |
| ccp-miR396b-1-5p | Cc02_g36500 | 2.0 | 18.051 | 1 | 19 | 1027 | 1046 | UUCCACA-GCUUUCUUGAAC | GUUCAAGAAAGCAUGUGGAA | Cleavage |
| ccp-miR396b-1-5p | Cc03_g04610 | 2.5 | 14.7 | 1 | 21 | 262 | 282 | UUCCACAGCUUUCUUGAACUU | GAGUUUAAGAAGGCUGAGGAA | Cleavage |
| ccp-miR396b-1-5p | Cc04_g00510 | 1.5 | 17.828 | 1 | 20 | 277 | 296 | UUCCACAGCUUUCUUGAACU | AGUUCAAGGAAACUGUGGAA | Translation |
| ccp-miR396b-1-5p | Cc05_g16590 | 3.0 | 20.196 | 1 | 21 | 1385 | 1405 | UUCCACAGCUUUCUUGAACUU | GAGUGGGAGGAAGCUGUGGAA | Cleavage |
| ccp-miR396b-1-5p | Cc06_g05390 | 2.0 | 17.417 | 1 | 19 | 785 | 804 | UUCCACA-GCUUUCUUGAAC | GUUCAAGAAAGCCUGUGGAA | Cleavage |
| ccp-miR396b-1-5p | Cc06_g08100 | 1.0 | 21.546 | 1 | 21 | 403 | 423 | UUCCACAGCUUUCUUGAACUU | AAGUUCAAGGAAGCUGUGGGA | Cleavage |
| ccp-miR396b-1-5p | Cc06_g12040 | 2.0 | 12.48 | 1 | 19 | 667 | 686 | UUCCACA-GCUUUCUUGAAC | GUUCAAGAAAGCCUGUGGAA | Cleavage |
| ccp-miR396b-1-5p | Cc07_g07350 | 2.0 | 24.972 | 1 | 19 | 1021 | 1040 | UUCCACA-GCUUUCUUGAAC | GUUCAAGAAAGCCUGUGGAA | Cleavage |
| ccp-miR396b-1-5p | Cc07_g09590 | 3.0 | 16.001 | 1 | 21 | 763 | 783 | UUCCACAGCUUUCUUGAACUU | GAGAUCAAGAGAUCUGUGGGA | Translation |
| ccp-miR396b-1-5p | Cc07_g20540 | 3.0 | 16.474 | 1 | 21 | 2080 | 2100 | UUCCACAGCUUUCUUGAACUU | AAGUUUAAUAAAGCUGUUGAA | Cleavage |
| ccp-miR396b-1-5p | Cc08_g05740 | 3.0 | 8.329 | 1 | 21 | 1726 | 1746 | UUCCACAGCUUUCUUGAACUU | AAGUUUGAGAAAGUUGUGAAA | Cleavage |
| ccp-miR396b-1-5p | Cc09_g08870 | 3.0 | 20.803 | 1 | 20 | 131 | 150 | UUCCACAGCUUUCUUGAACU | UGUUCAAGAAGGCUGCGGAA | Cleavage |
| ccp-miR396b-1-5p | Cc09_g09000 | 2.5 | 15.835 | 1 | 20 | 320 | 339 | UUCCACAGCUUUCUUGAACU | UGUUCAAGAAAGCUGAGGAA | Cleavage |
| ccp-miR396b-1-5p | Cc09_g10660 | 3.0 | 16.068 | 1 | 21 | 1108 | 1128 | UUCCACAGCUUUCUUGAACUU | AAGUACAAGAAGGAUGUGGAG | Cleavage |
| ccp-miR396b-1-5p | Cc11_g10740 | 3.0 | 19.787 | 1 | 21 | 220 | 240 | UUCCACAGCUUUCUUGAACUU | GAGUUCAAUGAGGUUGUGGAG | Cleavage |
| ccp-miR396b-1-5p | Cc11_g11830 | 3.0 | 19.658 | 1 | 20 | 1290 | 1309 | UUCCACAGCUUUCUUGAACU | AGUUCAAUAAUGCUGUGGAC | Translation |
| ccp-miR396b-2-5p | Cc00_g15760 | 3.0 | 19.582 | 1 | 20 | 142 | 161 | UUCCACAGCUUUCUUGAACU | AGUUGAAGAAAGUUCUGGAA | Cleavage |
| ccp-miR396b-2-5p | Cc02_g00930 | 2.0 | 16.902 | 1 | 19 | 827 | 846 | UUCCACA-GCUUUCUUGAAC | GUUCAAGAAAGCCUGUGGAA | Cleavage |
| ccp-miR396b-2-5p | Cc02_g13260 | 3.0 | 21.19 | 1 | 21 | 1195 | 1215 | UUCCACAGCUUUCUUGAACUU | GAGAUGGAGAAGGCUGUGGAA | Cleavage |
| ccp-miR396b-2-5p | Cc02_g36500 | 2.0 | 18.051 | 1 | 19 | 1027 | 1046 | UUCCACA-GCUUUCUUGAAC | GUUCAAGAAAGCAUGUGGAA | Cleavage |
| ccp-miR396b-2-5p | Cc03_g04610 | 2.5 | 14.7 | 1 | 21 | 262 | 282 | UUCCACAGCUUUCUUGAACUU | GAGUUUAAGAAGGCUGAGGAA | Cleavage |
| ccp-miR396b-2-5p | Cc04_g00510 | 1.5 | 17.828 | 1 | 20 | 277 | 296 | UUCCACAGCUUUCUUGAACU | AGUUCAAGGAAACUGUGGAA | Translation |
| ccp-miR396b-2-5p | Cc05_g16590 | 3.0 | 20.196 | 1 | 21 | 1385 | 1405 | UUCCACAGCUUUCUUGAACUU | GAGUGGGAGGAAGCUGUGGAA | Cleavage |
| ccp-miR396b-2-5p | Cc06_g05390 | 2.0 | 17.417 | 1 | 19 | 785 | 804 | UUCCACA-GCUUUCUUGAAC | GUUCAAGAAAGCCUGUGGAA | Cleavage |
| ccp-miR396b-2-5p | Cc06_g08100 | 1.0 | 21.546 | 1 | 21 | 403 | 423 | UUCCACAGCUUUCUUGAACUU | AAGUUCAAGGAAGCUGUGGGA | Cleavage |
| ccp-miR396b-2-5p | Cc06_g12040 | 2.0 | 12.48 | 1 | 19 | 667 | 686 | UUCCACA-GCUUUCUUGAAC | GUUCAAGAAAGCCUGUGGAA | Cleavage |
| ccp-miR396b-2-5p | Cc07_g07350 | 2.0 | 24.972 | 1 | 19 | 1021 | 1040 | UUCCACA-GCUUUCUUGAAC | GUUCAAGAAAGCCUGUGGAA | Cleavage |
| ccp-miR396b-2-5p | Cc07_g09590 | 3.0 | 16.001 | 1 | 21 | 763 | 783 | UUCCACAGCUUUCUUGAACUU | GAGAUCAAGAGAUCUGUGGGA | Translation |
| ccp-miR396b-2-5p | Cc07_g20540 | 3.0 | 16.474 | 1 | 21 | 2080 | 2100 | UUCCACAGCUUUCUUGAACUU | AAGUUUAAUAAAGCUGUUGAA | Cleavage |
| ccp-miR396b-2-5p | Cc08_g05740 | 3.0 | 8.329 | 1 | 21 | 1726 | 1746 | UUCCACAGCUUUCUUGAACUU | AAGUUUGAGAAAGUUGUGAAA | Cleavage |
| ccp-miR396b-2-5p | Cc09_g08870 | 3.0 | 20.803 | 1 | 20 | 131 | 150 | UUCCACAGCUUUCUUGAACU | UGUUCAAGAAGGCUGCGGAA | Cleavage |
| ccp-miR396b-2-5p | Cc09_g09000 | 2.5 | 15.835 | 1 | 20 | 320 | 339 | UUCCACAGCUUUCUUGAACU | UGUUCAAGAAAGCUGAGGAA | Cleavage |
| ccp-miR396b-2-5p | Cc09_g10660 | 3.0 | 16.068 | 1 | 21 | 1108 | 1128 | UUCCACAGCUUUCUUGAACUU | AAGUACAAGAAGGAUGUGGAG | Cleavage |
| ccp-miR396b-2-5p | Cc11_g10740 | 3.0 | 19.787 | 1 | 21 | 220 | 240 | UUCCACAGCUUUCUUGAACUU | GAGUUCAAUGAGGUUGUGGAG | Cleavage |
| ccp-miR396b-2-5p | Cc11_g11830 | 3.0 | 19.658 | 1 | 20 | 1290 | 1309 | UUCCACAGCUUUCUUGAACU | AGUUCAAUAAUGCUGUGGAC | Translation |
| ccp-miR396c-3p | Cc02_g00930 | 0.0 | 16.902 | 1 | 21 | 826 | 846 | UUCCACAGGCUUUCUUGAACG | CGUUCAAGAAAGCCUGUGGAA | Cleavage |
| ccp-miR396c-3p | Cc02_g36500 | 1.0 | 18.051 | 1 | 21 | 1026 | 1046 | UUCCACAGGCUUUCUUGAACG | CGUUCAAGAAAGCAUGUGGAA | Cleavage |
| ccp-miR396c-3p | Cc03_g07070 | 3.0 | 22.542 | 1 | 20 | 727 | 746 | UUCCACAGGCUUUCUUGAAC | GUUCAAGAAUGCUUGUGGGU | Translation |
| ccp-miR396c-3p | Cc05_g09230 | 3.0 | 15.748 | 1 | 20 | 564 | 583 | UUCCACAGGCUUUCUUGAAC | AUGCAAGGAAGUCUGUGGAA | Cleavage |
| ccp-miR396c-3p | Cc06_g05390 | 0.0 | 17.417 | 1 | 21 | 784 | 804 | UUCCACAGGCUUUCUUGAACG | CGUUCAAGAAAGCCUGUGGAA | Cleavage |
| ccp-miR396c-3p | Cc06_g06780 | 2.5 | 22.589 | 1 | 20 | 1276 | 1295 | UUCCACAGGCUUUCUUGAAC | GUUGAGGAAGGCCUGUGGAG | Cleavage |
| ccp-miR396c-3p | Cc06_g08100 | 3.0 | 21.546 | 1 | 20 | 405 | 423 | UUCCACAGGCUUUCUUGAAC | GUUCAAGGAAGC-UGUGGGA | Cleavage |
| ccp-miR396c-3p | Cc06_g12040 | 0.0 | 12.48 | 1 | 21 | 666 | 686 | UUCCACAGGCUUUCUUGAACG | CGUUCAAGAAAGCCUGUGGAA | Cleavage |
| ccp-miR396c-3p | Cc07_g07350 | 0.0 | 24.972 | 1 | 22 | 1019 | 1040 | UUCCACAGGCUUUCUUGAACGA | UCGUUCAAGAAAGCCUGUGGAA | Cleavage |
| ccp-miR396c-3p | Cc08_g01550 | 1.5 | 17.535 | 1 | 20 | 11694 | 11713 | UUCCACAGGCUUUCUUGAAC | UUUUAAGAAAGCCUGUGGAA | Cleavage |
| ccp-miR396c-3p | Cc11_g03230 | 3.0 | 17.854 | 1 | 21 | 1782 | 1802 | UUCCACAGGCUUUCUUGAACG | UGGUAAAGAAAGGCUGUGGAA | Translation |
| ccp-miR396c-3p | Cc11_g14710 | 2.5 | 17.491 | 1 | 21 | 142 | 162 | UUCCACAGGCUUUCUUGAACG | UGUUCAAGAAAGCCUGUGGUG | Cleavage |
| ccp-miR396f-5p | Cc00_g05620 | 2.5 | 20.368 | 1 | 25 | 396 | 420 | AGCUUUCUUGAGCUUCUUGAUUAGC | GGUCGUUAAGAAGCUAAGGAAGGCU | Translation |
| ccp-miR396f-5p | Cc00_g05660 | 3.0 | 23.396 | 1 | 25 | 396 | 420 | AGCUUUCUUGAGCUUCUUGAUUAGC | GGUGGUUAAGAAGCUAAGGAGGGCU | Translation |
| ccp-miR396f-5p | Cc00_g05670 | 2.5 | 20.248 | 1 | 25 | 609 | 633 | AGCUUUCUUGAGCUUCUUGAUUAGC | GGUCGUUAAGAAGCUAAGGAAGGCU | Translation |
| ccp-miR396f-5p | Cc00_g05690 | 2.5 | 17.48 | 1 | 25 | 396 | 420 | AGCUUUCUUGAGCUUCUUGAUUAGC | GGUGGUCAAGAAGCUAAGGAGGGCU | Translation |
| ccp-miR396f-5p | Cc00_g10620 | 2.5 | 16.534 | 1 | 25 | 261 | 285 | AGCUUUCUUGAGCUUCUUGAUUAGC | GCAAUUCAAGAACUUUAGGAAAGCU | Cleavage |
| ccp-miR396f-5p | Cc00_g12070 | 3.0 | 22.48 | 1 | 20 | 1632 | 1651 | AGCUUUCUUGAGCUUCUUGA | UCGAGAGGUUCAAGAAAGAU | Cleavage |
| ccp-miR396f-5p | Cc00_g26370 | 2.5 | 19.255 | 1 | 25 | 396 | 420 | AGCUUUCUUGAGCUUCUUGAUUAGC | GGUGGUCAAGAAGCUAAGGAGGGCU | Translation |
| ccp-miR396f-5p | Cc00_g35910 | 3.0 | 17.27 | 1 | 25 | 396 | 420 | AGCUUUCUUGAGCUUCUUGAUUAGC | GGUAGUCAAGAAGUUAAGGAGGGCU | Translation |
| ccp-miR396f-5p | Cc02_g03320 | 3.0 | 13.81 | 1 | 25 | 1233 | 1257 | AGCUUUCUUGAGCUUCUUGAUUAGC | GCAAUGCAAAAAGCUCAAGAAGGUU | Cleavage |
| ccp-miR396f-5p | Cc02_g04000 | 2.0 | 18.728 | 1 | 21 | 226 | 246 | AGCUUUCUUGAGCUUCUUGAU | GUCAAGAACCUCGAGAAAGUU | Cleavage |
| ccp-miR396f-5p | Cc02_g04020 | 2.0 | 19.258 | 1 | 21 | 380 | 400 | AGCUUUCUUGAGCUUCUUGAU | GUCAAGAACCUCGAGAAAGUU | Cleavage |
| ccp-miR396f-5p | Cc02_g04410 | 3.0 | 22.875 | 1 | 24 | 514 | 537 | AGCUUUCUUGAGCUUCUUGAUUAG | UUGGGCAAGAAGAUCAAGAAAGCG | Cleavage |
| ccp-miR396f-5p | Cc02_g12540 | 3.0 | 16.387 | 1 | 20 | 585 | 604 | AGCUUUCUUGAGCUUCUUGA | UCAAGAAGUUCAAGAAAGGC | Cleavage |
| ccp-miR396f-5p | Cc02_g12650 | 2.0 | 18.695 | 1 | 25 | 411 | 436 | AGCUUUCUUGAGCUUCUUGAU-UAGC | GCUGUGUCAAAGAGUUCAAGAAAGCU | Cleavage |
| ccp-miR396f-5p | Cc02_g14870 | 2.5 | 18.425 | 1 | 22 | 252 | 273 | AGCUUUCUUGAGCUUCUUGAUU | GAUCAAGAAGCUCAAGGGAGUG | Cleavage |
| ccp-miR396f-5p | Cc02_g16440 | 2.5 | 19.732 | 1 | 25 | 2145 | 2169 | AGCUUUCUUGAGCUUCUUGAUUAGC | GGUGGUCAAGAAGCUAAGGAGGGCU | Translation |
| ccp-miR396f-5p | Cc02_g28000 | 2.0 | 16.286 | 1 | 22 | 195 | 216 | AGCUUUCUUGAGCUUCUUGAUU | GAUCAAGAAUCUCAAGGAAGUU | Cleavage |
| ccp-miR396f-5p | Cc02_g29320 | 2.5 | 20.484 | 1 | 25 | 396 | 420 | AGCUUUCUUGAGCUUCUUGAUUAGC | GGUGGUCAAGAAGCUAAGGAGGGCU | Translation |
| ccp-miR396f-5p | Cc02_g29340 | 2.5 | 17.48 | 1 | 25 | 291 | 315 | AGCUUUCUUGAGCUUCUUGAUUAGC | GGUGGUCAAGAAGCUAAGGAGGGCU | Translation |
| ccp-miR396f-5p | Cc02_g31000 | 3.0 | 14.081 | 1 | 23 | 4175 | 4197 | AGCUUUCUUGAGCUUCUUGAUUA | UGGUUAAGGAGCUCCAGAAAGCA | Translation |
| ccp-miR396f-5p | Cc02_g33640 | 3.0 | 17.196 | 1 | 22 | 603 | 624 | AGCUUUCUUGAGCUUCUUGAUU | GAUUAAGAAGAUCAUGAAGGCU | Cleavage |
| ccp-miR396f-5p | Cc02_g39270 | 3.0 | 16.333 | 1 | 24 | 967 | 990 | AGCUUUCUUGAGCUUCUUGAUUAG | CCAGUAAAGAAGCUGAAGGAAGUU | Translation |
| ccp-miR396f-5p | Cc03_g12420 | 2.5 | 14.905 | 1 | 25 | 408 | 432 | AGCUUUCUUGAGCUUCUUGAUUAGC | GCAAUUCAAGAACUUUAGGAAAGCU | Cleavage |
| ccp-miR396f-5p | Cc04_g09150 | 2.5 | 21.919 | 1 | 22 | 538 | 559 | AGCUUUCUUGAGCUUCUUGAUU | AGCCAAGAAGCCUAAGAAAGCU | Translation |
| ccp-miR396f-5p | Cc04_g14730 | 3.0 | 19.907 | 1 | 24 | 118 | 142 | AGCUU-UCUUGAGCUUCUUGAUUAG | CUCCUCAAGAAGCUCAAGACGAGCU | Cleavage |
| ccp-miR396f-5p | Cc05_g04930 | 3.0 | 17.643 | 1 | 25 | 298 | 322 | AGCUUUCUUGAGCUUCUUGAUUAGC | GCUACUGAAGAAACUCAAGGAGGCU | Cleavage |
| ccp-miR396f-5p | Cc05_g06900 | 2.5 | 16.495 | 1 | 20 | 279 | 298 | AGCUUUCUUGAGCUUCUUGA | CUGAGAAGCUCAAGAGAGCU | Cleavage |
| ccp-miR396f-5p | Cc05_g07410 | 3.0 | 16.002 | 1 | 25 | 390 | 414 | AGCUUUCUUGAGCUUCUUGAUUAGC | GAUGGCUAAGAAGAUCAAGAAAGUU | Cleavage |
| ccp-miR396f-5p | Cc06_g00810 | 3.0 | 17.979 | 1 | 23 | 2555 | 2577 | AGCUUUCUUGAGCUUCUUGAUUA | UCAUGAAGAUGCUUGAGAAAGCU | Cleavage |
| ccp-miR396f-5p | Cc06_g16020 | 2.5 | 20.278 | 1 | 20 | 4703 | 4722 | AGCUUUCUUGAGCUUCUUGA | CCAAGAAGUUUAAGAAGGCU | Cleavage |
| ccp-miR396f-5p | Cc06_g21040 | 2.5 | 9.976 | 1 | 25 | 256 | 280 | AGCUUUCUUGAGCUUCUUGAUUAGC | GUUGAUCAAGAAACUCAAGAAAGUA | Cleavage |
| ccp-miR396f-5p | Cc06_g21600 | 3.0 | 16.352 | 1 | 22 | 821 | 842 | AGCUUUCUUGAGCUUCUUGAUU | GGUCAAGAAGCUCCAGAGGGCA | Translation |
| ccp-miR396f-5p | Cc07_g05430 | 2.5 | 18.346 | 1 | 22 | 4621 | 4642 | AGCUUUCUUGAGCUUCUUGAUU | AAACAAGAAGCUGAAGGAAGCU | Translation |
| ccp-miR396f-5p | Cc07_g15340 | 3.0 | 13.446 | 1 | 23 | 838 | 860 | AGCUUUCUUGAGCUUCUUGAUUA | UAGUCAAGGAGCUGAAGAAAACU | Translation |
| ccp-miR396f-5p | Cc07_g17780 | 2.0 | 18.38 | 1 | 20 | 815 | 834 | AGCUUUCUUGAGCUUCUUGA | UUAGGGAGUUCAAGAAAGCU | Cleavage |
| ccp-miR396f-5p | Cc08_g02710 | 1.0 | 19.008 | 1 | 20 | 1181 | 1200 | AGCUUUCUUGAGCUUCUUGA | UCAGGAAGUUCAAGAAAGCU | Cleavage |
| ccp-miR396f-5p | Cc08_g07060 | 3.0 | 16.736 | 1 | 24 | 1398 | 1421 | AGCUUUCUUGAGCUUCUUGAUUAG | UUAUUCAAGAUGUUUAAGGGAGCU | Cleavage |
| ccp-miR396f-5p | Cc09_g03400 | 2.5 | 16.305 | 1 | 24 | 2194 | 2217 | AGCUUUCUUGAGCUUCUUGAUUAG | CUCGUGAAGAAGCUCAAGAAAGUC | Cleavage |
| ccp-miR396f-5p | Cc09_g03900 | 3.0 | 17.453 | 1 | 25 | 760 | 784 | AGCUUUCUUGAGCUUCUUGAUUAGC | GAUAAAUGAGAAGCUAAAGAAAGCU | Translation |
| ccp-miR396f-5p | Cc10_g00050 | 3.0 | 19.166 | 1 | 23 | 2101 | 2123 | AGCUUUCUUGAGCUUCUUGAUUA | UGACCGAGAAGCUCAAAAAAGCU | Cleavage |
| ccp-miR396f-5p | Cc11_g03450 | 2.5 | 19.263 | 1 | 22 | 1041 | 1062 | AGCUUUCUUGAGCUUCUUGAUU | AAUCAAGAUCCUCAAGGAAGCU | Cleavage |
| ccp-miR396f-5p | Cc11_g14500 | 2.0 | 17.061 | 1 | 25 | 564 | 588 | AGCUUUCUUGAGCUUCUUGAUUAGC | GCUUGUCAAGAAGCCCAAGAGAGUU | Translation |
| ccp-miR396f-5p | Cc11_g14620 | 3.0 | 17.578 | 1 | 20 | 830 | 849 | AGCUUUCUUGAGCUUCUUGA | UCGAGAGGUUCAAGAAAGAU | Cleavage |
| ccp-miR396f-5p | Cc11_g17520 | 2.5 | 18.866 | 1 | 21 | 267 | 287 | AGCUUUCUUGAGCUUCUUGAU | AUAAACAAGCUCAAGAAGGCU | Cleavage |
| ccp-miR396g-1-3p | Cc02_g18640 | 3.0 | 21.145 | 1 | 20 | 1334 | 1353 | GUUCAAGAAAGCUGUGGAAA | UUGCCAUGGCUUUUUUGGAC | Cleavage |
| ccp-miR396g-1-3p | Cc02_g19710 | 2.5 | 14.421 | 1 | 21 | 161 | 181 | GUUCAAGAAAGCUGUGGAAAA | UUUUCCAUAGCUUUCUUGGUC | Cleavage |
| ccp-miR396g-1-3p | Cc02_g31140 | 3.0 | 11.697 | 1 | 20 | 155 | 173 | GUUCAAGAAAGCUGUGGAAA | UUUCCGUA-CUUUCUUGAAC | Cleavage |
| ccp-miR396g-1-3p | Cc03_g09150 | 3.0 | 10.598 | 1 | 20 | 133 | 152 | GUUCAAGAAAGCUGUGGAAA | CCUUCACAGCUUUCUUGAGC | Cleavage |
| ccp-miR396g-1-3p | Cc03_g12030 | 3.0 | 11.242 | 1 | 20 | 1182 | 1201 | GUUCAAGAAAGCUGUGGAAA | UUUUCAUAGCUCCCUUGAAC | Translation |
| ccp-miR396g-1-3p | Cc06_g08100 | 0.0 | 10.294 | 1 | 21 | 303 | 323 | GUUCAAGAAAGCUGUGGAAAA | UUUUCCACAGCUUUCUUGAAC | Cleavage |
| ccp-miR396g-1-3p | Cc07_g19350 | 3.0 | 9.503 | 1 | 21 | 188 | 208 | GUUCAAGAAAGCUGUGGAAAA | UUUUCUGUAGCUUCCUUGAAU | Cleavage |
| ccp-miR396g-1-3p | Cc10_g11850 | 3.0 | 12.132 | 1 | 20 | 328 | 347 | GUUCAAGAAAGCUGUGGAAA | UUUUUAUGACUUUCUUGAAC | Cleavage |
| ccp-miR397a-1-3p | Cc01_g18220 | 3.0 | 17.221 | 1 | 20 | 687 | 706 | UCUGCGCUGCACCCAAUCAU | AAGAUUGGGUGCUGUGUAGA | Cleavage |
| ccp-miR397a-1-3p | Cc11_g03200 | 3.0 | 21.759 | 1 | 21 | 1744 | 1764 | UCUGCGCUGCACCCAAUCAUG | UAUGGCUGGGAGCAGCGUAGA | Translation |
| ccp-miR397a-1-5p | Cc01_g13110 | 3.0 | 13.878 | 1 | 21 | 675 | 695 | UCAUUGAGUGCAGCGUUGAUG | CAUCAAUGCCGCACUCAAUCA | Cleavage |
| ccp-miR397a-1-5p | Cc01_g13190 | 1.0 | 14.614 | 1 | 20 | 877 | 896 | UCAUUGAGUGCAGCGUUGAU | AUCAAUGCUGCGCUCAAUGA | Cleavage |
| ccp-miR397a-1-5p | Cc01_g13860 | 2.5 | 10.619 | 1 | 21 | 642 | 662 | UCAUUGAGUGCAGCGUUGAUG | CAUCAAUGCAGCGCUUAAUGA | Cleavage |
| ccp-miR397a-1-5p | Cc01_g13870 | 1.0 | 15.862 | 1 | 21 | 1269 | 1289 | UCAUUGAGUGCAGCGUUGAUG | CAUCAAUGCUGCACUUAAUGA | Cleavage |
| ccp-miR397a-1-5p | Cc01_g14850 | 1.5 | 17.398 | 1 | 21 | 4064 | 4084 | UCAUUGAGUGCAGCGUUGAUG | CAUCAACGCUGCACUCAAUCA | Cleavage |
| ccp-miR397a-1-5p | Cc01_g21550 | 2.5 | 17.51 | 1 | 21 | 877 | 897 | UCAUUGAGUGCAGCGUUGAUG | UGUCAAUGCUGGACUUAAUGA | Translation |
| ccp-miR397a-1-5p | Cc02_g17410 | 3.0 | 10.286 | 1 | 21 | 771 | 791 | UCAUUGAGUGCAGCGUUGAUG | CAUCAAUGCGGCACUCAACGA | Cleavage |
| ccp-miR397a-1-5p | Cc04_g06530 | 2.5 | 13.959 | 1 | 21 | 675 | 695 | UCAUUGAGUGCAGCGUUGAUG | CAUCAAUGCCGCCCUCAAUGA | Translation |
| ccp-miR397a-1-5p | Cc05_g00010 | 3.0 | 11.904 | 1 | 20 | 2290 | 2309 | UCAUUGAGUGCAGCGUUGAU | AUUUACGUUGCAUUCGAUGA | Cleavage |
| ccp-miR397a-1-5p | Cc06_g01620 | 0.5 | 19.759 | 1 | 21 | 740 | 760 | UCAUUGAGUGCAGCGUUGAUG | UAUCAAUGCUGCACUCAAUGA | Cleavage |
| ccp-miR397a-1-5p | Cc06_g01880 | 2.0 | 12.287 | 1 | 21 | 663 | 683 | UCAUUGAGUGCAGCGUUGAUG | CAUCAAUGCUGCACUCAAUAA | Cleavage |
| ccp-miR397a-1-5p | Cc06_g02430 | 2.0 | 17.151 | 1 | 20 | 793 | 812 | UCAUUGAGUGCAGCGUUGAU | AUCAACGCUGCGCUAAAUGA | Cleavage |
| ccp-miR397a-1-5p | Cc06_g02440 | 1.5 | 16.015 | 1 | 20 | 836 | 855 | UCAUUGAGUGCAGCGUUGAU | AUCAACGCUGCACUCAACGA | Cleavage |
| ccp-miR397a-1-5p | Cc06_g03130 | 3.0 | 20.445 | 1 | 21 | 3141 | 3161 | UCAUUGAGUGCAGCGUUGAUG | UGUCAAUGCUCAACUCAAUGA | Translation |
| ccp-miR397a-1-5p | Cc07_g14670 | 1.5 | 15.492 | 1 | 20 | 700 | 719 | UCAUUGAGUGCAGCGUUGAU | AUCAACGCUGCACUAAAUGA | Cleavage |
| ccp-miR397a-1-5p | Cc07_g14990 | 3.0 | 10.748 | 1 | 20 | 304 | 323 | UCAUUGAGUGCAGCGUUGAU | AUCUAUGCUGCACUCAAUCA | Cleavage |
| ccp-miR397a-1-5p | Cc08_g00150 | 3.0 | 16.551 | 1 | 21 | 387 | 407 | UCAUUGAGUGCAGCGUUGAUG | CAUCCAUGCUUCACUCGAUGA | Translation |
| ccp-miR397a-3-3p | Cc04_g04500 | 3.0 | 20.956 | 1 | 20 | 1315 | 1334 | CAUCAACGCUGCACUCAAUG | GAUUUAGUGCAGAGUUGAUG | Cleavage |
| ccp-miR397a-3-3p | Cc08_g10610 | 3.0 | 18.518 | 1 | 21 | 1645 | 1665 | CAUCAACGCUGCACUCAAUGA | UUUUUGGGUGCAGGGUUGAUG | Cleavage |
| ccp-miR397a-3-3p | Cc11_g12940 | 3.0 | 23.663 | 1 | 21 | 666 | 686 | CAUCAACGCUGCACUCAAUGA | UCAUCGAGUGCAACGUUGAUC | Translation |
| ccp-miR397a-3-5p | Cc00_g19320 | 2.5 | 16.516 | 1 | 20 | 500 | 519 | AUUGGGUGCAGCGCAGAUGA | UCAUCUGUGUUGCACUCAAG | Cleavage |
| ccp-miR397a-3-5p | Cc01_g14850 | 2.5 | 17.391 | 1 | 20 | 4063 | 4082 | AUUGGGUGCAGCGCAGAUGA | UCAUCAACGCUGCACUCAAU | Cleavage |
| ccp-miR397a-3-5p | Cc02_g08850 | 3.0 | 19.192 | 1 | 21 | 2597 | 2617 | AUUGGGUGCAGCGCAGAUGAA | UUUCUCUGUGAUGCACCCAAU | Translation |
| ccp-miR397a-3-5p | Cc02_g32190 | 3.0 | 11.897 | 1 | 21 | 2226 | 2246 | AUUGGGUGCAGCGCAGAUGAA | UUCAACUGCUUUGCAUCCAAU | Cleavage |
| ccp-miR397a-3-5p | Cc02_g37680 | 2.5 | 15.005 | 1 | 21 | 747 | 767 | AUUGGGUGCAGCGCAGAUGAA | UGCAUCUGCGCCGCGCCCAAU | Translation |
| ccp-miR397a-3-5p | Cc03_g03580 | 3.0 | 17.218 | 1 | 21 | 1699 | 1719 | AUUGGGUGCAGCGCAGAUGAA | UUUAAGUGCGCUGCAUCCAAU | Cleavage |
| ccp-miR397a-3-5p | Cc06_g01880 | 3.0 | 12.232 | 1 | 20 | 662 | 681 | AUUGGGUGCAGCGCAGAUGA | UCAUCAAUGCUGCACUCAAU | Cleavage |
| ccp-miR397a-3-5p | Cc06_g12900 | 3.0 | 21.165 | 1 | 21 | 1851 | 1871 | AUUGGGUGCAGCGCAGAUGAA | UUCGUCCGUGUUGCACUCAAU | Cleavage |
| ccp-miR397a-3-5p | Cc10_g07860 | 3.0 | 21.188 | 1 | 20 | 2617 | 2636 | AUUGGGUGCAGCGCAGAUGA | UUGUCUGCUCUGCACUUAAU | Cleavage |
| ccp-miR397a-4-3p | Cc02_g08850 | 3.0 | 16.444 | 1 | 20 | 2601 | 2620 | AUGAUUGGGUGCAGCGCAGA | UCUGUGAUGCACCCAAUCUU | Cleavage |
| ccp-miR397a-4-3p | Cc06_g12900 | 2.5 | 22.633 | 1 | 22 | 1853 | 1874 | AUGAUUGGGUGCAGCGCAGAUG | CGUCCGUGUUGCACUCAAUCAU | Cleavage |
| ccp-miR397a-4-3p | Cc07_g14990 | 3.0 | 10.994 | 1 | 21 | 304 | 324 | AUGAUUGGGUGCAGCGCAGAU | AUCUAUGCUGCACUCAAUCAC | Cleavage |
| ccp-miR397a-4-3p | Cc10_g11940 | 2.5 | 21.22 | 1 | 20 | 281 | 300 | AUGAUUGGGUGCAGCGCAGA | UCUGAGCUGCUUCCAAUCAU | Translation |
| ccp-miR397a-4-5p | Cc00_g19320 | 2.5 | 16.516 | 1 | 20 | 500 | 519 | AUUGGGUGCAGCGCAGAUGA | UCAUCUGUGUUGCACUCAAG | Cleavage |
| ccp-miR397a-4-5p | Cc01_g14850 | 2.5 | 17.391 | 1 | 20 | 4063 | 4082 | AUUGGGUGCAGCGCAGAUGA | UCAUCAACGCUGCACUCAAU | Cleavage |
| ccp-miR397a-4-5p | Cc02_g08850 | 3.0 | 19.192 | 1 | 21 | 2597 | 2617 | AUUGGGUGCAGCGCAGAUGAA | UUUCUCUGUGAUGCACCCAAU | Translation |
| ccp-miR397a-4-5p | Cc02_g32190 | 3.0 | 11.897 | 1 | 21 | 2226 | 2246 | AUUGGGUGCAGCGCAGAUGAA | UUCAACUGCUUUGCAUCCAAU | Cleavage |
| ccp-miR397a-4-5p | Cc02_g37680 | 2.5 | 15.005 | 1 | 21 | 747 | 767 | AUUGGGUGCAGCGCAGAUGAA | UGCAUCUGCGCCGCGCCCAAU | Translation |
| ccp-miR397a-4-5p | Cc03_g03580 | 3.0 | 17.218 | 1 | 21 | 1699 | 1719 | AUUGGGUGCAGCGCAGAUGAA | UUUAAGUGCGCUGCAUCCAAU | Cleavage |
| ccp-miR397a-4-5p | Cc06_g01880 | 3.0 | 12.232 | 1 | 20 | 662 | 681 | AUUGGGUGCAGCGCAGAUGA | UCAUCAAUGCUGCACUCAAU | Cleavage |
| ccp-miR397a-4-5p | Cc06_g12900 | 3.0 | 21.165 | 1 | 21 | 1851 | 1871 | AUUGGGUGCAGCGCAGAUGAA | UUCGUCCGUGUUGCACUCAAU | Cleavage |
| ccp-miR397a-4-5p | Cc10_g07860 | 3.0 | 21.188 | 1 | 20 | 2617 | 2636 | AUUGGGUGCAGCGCAGAUGA | UUGUCUGCUCUGCACUUAAU | Cleavage |
| ccp-miR398-3p | Cc08_g11810 | 2.0 | 17.896 | 1 | 20 | 1225 | 1244 | UGUGUUCUCAUGUUGCCCCU | AGGGGGAACAUGAGAGUACA | Cleavage |
| ccp-miR398-5p | Cc05_g04080 | 3.0 | 17.953 | 1 | 20 | 1758 | 1777 | GGGGCGACCUGAGAACACAU | AUGUGUUCACGGGUCGUUUC | Cleavage |
| ccp-miR398a-3p | Cc03_g02460 | 3.0 | 17.492 | 1 | 20 | 196 | 215 | UGUGUUCUCAGGUCACCCCU | AGGGGUGUUCUGAGAUCACA | Cleavage |
| ccp-miR398a-3p | Cc06_g02470 | 2.5 | 21.185 | 1 | 20 | 2103 | 2122 | UGUGUUCUCAGGUCACCCCU | AGGGGUGAACUGAGAACAUU | Cleavage |
| ccp-miR398a-3p | Cc06_g08270 | 2.5 | 20.113 | 1 | 20 | 257 | 277 | UGUGUUCUCAGGUC-ACCCCU | AGGGGUCGACUUGAGAACACA | Cleavage |
| ccp-miR398b-3p | Cc06_g02470 | 3.0 | 21.185 | 1 | 21 | 2102 | 2122 | UGUGUUCUCAGGUCGCCCCUG | CAGGGGUGAACUGAGAACAUU | Cleavage |
| ccp-miR398b-3p | Cc06_g08270 | 2.5 | 20.113 | 1 | 21 | 256 | 277 | UGUGUUCUCAGGUCG-CCCCUG | CAGGGGUCGACUUGAGAACACA | Cleavage |
| ccp-miR398b-5p | Cc02_g30260 | 3.0 | 18.878 | 1 | 21 | 5637 | 5657 | GGGGCAACAUGAGAACACAUA | UAGGUGUUUUUAUGUUGUCUC | Cleavage |
| ccp-miR398b-5p | Cc04_g04040 | 3.0 | 15.092 | 1 | 20 | 656 | 676 | GGGGCAAC-AUGAGAACACAU | GUGUGUUUUCAUAGUUGCCUC | Translation |
| ccp-miR398b-5p | Cc07_g14490 | 3.0 | 21.129 | 1 | 20 | 375 | 394 | GGGGCAACAUGAGAACACAU | AUGUGUUCUUUUCUUGUCCC | Translation |
| ccp-miR398b-5p | Cc07_g20820 | 3.0 | 17.839 | 1 | 20 | 1541 | 1560 | GGGGCAACAUGAGAACACAU | AUGUGUUCUGGUUUUGCCUC | Translation |
| ccp-miR399-1-3p | Cc02_g13390 | 2.0 | 20.786 | 1 | 21 | 277 | 297 | UGCCAAAGGAGAAUUGCUCUG | UGGGGCAAAUCUCCUUUGGCA | Cleavage |
| ccp-miR399-1-3p | Cc03_g11870 | 3.0 | 13.099 | 1 | 20 | 337 | 356 | UGCCAAAGGAGAAUUGCUCU | GGAGAAAUUUUUUUUUGGCA | Cleavage |
| ccp-miR399-1-3p | Cc07_g08090 | 3.0 | 14.508 | 1 | 20 | 913 | 932 | UGCCAAAGGAGAAUUGCUCU | GGAGCAUUUCUCCUGUGGCA | Cleavage |
| ccp-miR399-1-3p | Cc07_g20990 | 3.0 | 22.194 | 1 | 20 | 2295 | 2313 | UGCCAAAGGAGAAUUGCUCU | GGAGUAAUUCUC-UUUGGCA | Cleavage |
| ccp-miR399-1-5p | Cc03_g03570 | 3.0 | 19.402 | 1 | 20 | 2698 | 2717 | CAUAGCGAUUCUCCUUUGUC | GAUGAGGGAGAAUUGUUGUG | Cleavage |
| ccp-miR399-1-5p | Cc07_g15860 | 3.0 | 16.455 | 1 | 20 | 659 | 678 | CAUAGCGAUUCUCCUUUGUC | GACAAACGAGGAUGGCUAUG | Cleavage |
| ccp-miR399-1-5p | Cc08_g11860 | 1.5 | 22.377 | 1 | 20 | 1160 | 1179 | CAUAGCGAUUCUCCUUUGUC | GACCAAGGAGAAUCGCUGUG | Cleavage |
| ccp-miR399-1-5p | Cc10_g12630 | 3.0 | 24.138 | 1 | 20 | 1828 | 1847 | CAUAGCGAUUCUCCUUUGUC | GAUGAAGGAGAACCGUUGUG | Cleavage |
| ccp-miR399-2-3p | Cc00_g22210 | 3.0 | 17.534 | 1 | 21 | 841 | 861 | UGCCAAAGGAGAAUUGCCCUG | UGGGUUAAUUCUCUUUUGGUA | Cleavage |
| ccp-miR399-2-3p | Cc02_g13390 | 1.5 | 20.786 | 1 | 21 | 277 | 297 | UGCCAAAGGAGAAUUGCCCUG | UGGGGCAAAUCUCCUUUGGCA | Cleavage |
| ccp-miR399-2-3p | Cc07_g19050 | 3.0 | 22.862 | 1 | 21 | 76 | 96 | UGCCAAAGGAGAAUUGCCCUG | UAGGGCCUUUUUCCUUUGGUA | Cleavage |
| ccp-miR399-2-5p | Cc00_g24960 | 3.0 | 19.099 | 1 | 20 | 1751 | 1770 | GUGUGAUUCUCCUUUGGCAU | AUGCUAAAAGAGAAGCACAC | Cleavage |
| ccp-miR399-2-5p | Cc02_g23410 | 3.0 | 17.857 | 1 | 20 | 902 | 921 | GUGUGAUUCUCCUUUGGCAU | AUGGAGAAGGAGAAUCAUAC | Cleavage |
| ccp-miR399-2-5p | Cc05_g10850 | 3.0 | 17.494 | 1 | 21 | 1547 | 1567 | GUGUGAUUCUCCUUUGGCAUG | UAUGCAAAAUGGGAAUUACAC | Cleavage |
| ccp-miR399-2-5p | Cc06_g04570 | 2.5 | 22.0 | 1 | 19 | 638 | 657 | GUGUGAUUCUCC-UUUGGCA | UGCCAAAUGGAGAAUCAUAC | Cleavage |
| ccp-miR399-2-5p | Cc09_g02930 | 3.0 | 23.31 | 1 | 20 | 2720 | 2739 | GUGUGAUUCUCCUUUGGCAU | AUGCCAAAGAAGGAUUGCAU | Translation |
| ccp-miR399-3-3p | Cc00_g22210 | 3.0 | 17.534 | 1 | 21 | 841 | 861 | UGCCAAAGGAGAAUUGCCCUG | UGGGUUAAUUCUCUUUUGGUA | Cleavage |
| ccp-miR399-3-3p | Cc02_g13390 | 1.5 | 20.786 | 1 | 21 | 277 | 297 | UGCCAAAGGAGAAUUGCCCUG | UGGGGCAAAUCUCCUUUGGCA | Cleavage |
| ccp-miR399-3-3p | Cc07_g19050 | 3.0 | 22.862 | 1 | 21 | 76 | 96 | UGCCAAAGGAGAAUUGCCCUG | UAGGGCCUUUUUCCUUUGGUA | Cleavage |
| ccp-miR399-3-5p | Cc02_g16340 | 3.0 | 24.654 | 1 | 20 | 1404 | 1423 | CAGGGCCGUUCUCCUUUGGC | GCCAAAUGGGAUUGGCCCUG | Translation |
| ccp-miR399-3-5p | Cc03_g06250 | 3.0 | 23.949 | 1 | 21 | 1675 | 1695 | CAGGGCCGUUCUCCUUUGGCA | UGCCAGAGGAGGAUGGUCCUU | Cleavage |
| ccp-miR399-3-5p | Cc08_g02940 | 3.0 | 23.191 | 1 | 21 | 581 | 601 | CAGGGCCGUUCUCCUUUGGCA | UCCUAAGGGAGAAUGGCCUUG | Cleavage |
| ccp-miR399-4-3p | Cc02_g13390 | 0.0 | 20.786 | 1 | 21 | 277 | 297 | UGCCAAAGGAGAUUUGCCCCG | UGGGGCAAAUCUCCUUUGGCA | Cleavage |
| ccp-miR399-4-3p | Cc02_g13390 | 1.0 | 21.105 | 1 | 20 | 223 | 242 | UGCCAAAGGAGAUUUGCCCC | UGGGCAAAUCUCCUUUGGCA | Cleavage |
| ccp-miR399-4-3p | Cc02_g22540 | 2.5 | 16.122 | 1 | 20 | 1233 | 1252 | UGCCAAAGGAGAUUUGCCCC | CUGGCAAAUCUCUUUUGGCA | Cleavage |
| ccp-miR399-4-3p | Cc05_g07830 | 2.5 | 13.634 | 1 | 20 | 588 | 607 | UGCCAAAGGAGAUUUGCCCC | GGGGAAAAUUUCCUUUGGCU | Cleavage |
| ccp-miR399-4-3p | Cc11_g08330 | 2.5 | 22.161 | 1 | 20 | 1781 | 1800 | UGCCAAAGGAGAUUUGCCCC | GGGACAAGUUUCUUUUGGCA | Cleavage |
| ccp-miR399-4-5p | Cc00_g14400 | 2.5 | 12.11 | 1 | 20 | 523 | 542 | GGCAAAUACUCCUUUGGCAG | UUGCAAAAGGAGGAUUUGCC | Cleavage |
| ccp-miR399-4-5p | Cc02_g02560 | 3.0 | 18.56 | 1 | 21 | 886 | 906 | GGCAAAUACUCCUUUGGCAGG | CCUGCCGAGGCUGUAUUUGCC | Translation |
| ccp-miR399-4-5p | Cc07_g20880 | 3.0 | 15.441 | 1 | 21 | 619 | 639 | GGCAAAUACUCCUUUGGCAGG | UUUGCCAAUAGAGUGUUUGCC | Cleavage |
| ccp-miR399-5-3p | Cc02_g30570 | 3.0 | 19.02 | 1 | 20 | 2232 | 2251 | UGCCAAAGGAGAAUCACACU | AGUGUGAUUAUACUUUGGUG | Translation |
| ccp-miR399-5-3p | Cc07_g10950 | 3.0 | 20.953 | 1 | 20 | 2955 | 2975 | UGCCAAA-GGAGAAUCACACU | AGUGUGCUUCUCCGUUUGGCA | Cleavage |
| ccp-miR399-5-3p | Cc10_g06710 | 2.0 | 16.625 | 1 | 20 | 889 | 908 | UGCCAAAGGAGAAUCACACU | AGUCUGAUUCUCGUUUGGCA | Cleavage |
| ccp-miR399-5-5p | Cc01_g13230 | 3.0 | 15.895 | 1 | 20 | 3621 | 3640 | CAGGGCAAUUCUCCUUUGGC | GUGAAAGGAGAAUUGCUCUU | Cleavage |
| ccp-miR399-5-5p | Cc02_g35800 | 2.5 | 13.657 | 1 | 21 | 362 | 382 | CAGGGCAAUUCUCCUUUGGCA | UGUUAAAGGAGAAAUGUCCUG | Cleavage |
| ccp-miR399-5-5p | Cc04_g02120 | 3.0 | 20.144 | 1 | 21 | 713 | 733 | CAGGGCAAUUCUCCUUUGGCA | UGCCAAGGAAGAAUUUCCCUG | Cleavage |
| ccp-miR399-5-5p | Cc05_g00500 | 3.0 | 24.978 | 1 | 20 | 101 | 120 | CAGGGCAAUUCUCCUUUGGC | GCCAGAGGGCAAUUGUCUUG | Translation |
| ccp-miR399-5-5p | Cc09_g07230 | 3.0 | 20.615 | 1 | 21 | 402 | 422 | CAGGGCAAUUCUCCUUUGGCA | UGCAAAUGGAGAGUUGUCCUG | Cleavage |
| ccp-miR399-6-3p | Cc01_g03630 | 3.0 | 21.363 | 1 | 20 | 2957 | 2976 | UGCCAAAGGAGAUUUGUCCC | GGCACAAAUCCUUUUUGGCA | Translation |
| ccp-miR399-6-3p | Cc02_g13390 | 0.5 | 20.786 | 1 | 21 | 277 | 297 | UGCCAAAGGAGAUUUGUCCCG | UGGGGCAAAUCUCCUUUGGCA | Cleavage |
| ccp-miR399-6-3p | Cc02_g22540 | 3.0 | 16.122 | 1 | 20 | 1233 | 1252 | UGCCAAAGGAGAUUUGUCCC | CUGGCAAAUCUCUUUUGGCA | Cleavage |
| ccp-miR399-6-3p | Cc02_g31340 | 3.0 | 18.676 | 1 | 21 | 3385 | 3405 | UGCCAAAGGAGAUUUGUCCCG | UGGAACACAUUUUCUUUGGCA | Cleavage |
| ccp-miR399-6-3p | Cc05_g07830 | 3.0 | 13.634 | 1 | 20 | 588 | 607 | UGCCAAAGGAGAUUUGUCCC | GGGGAAAAUUUCCUUUGGCU | Cleavage |
| ccp-miR399-6-3p | Cc11_g08330 | 1.5 | 22.161 | 1 | 20 | 1781 | 1800 | UGCCAAAGGAGAUUUGUCCC | GGGACAAGUUUCUUUUGGCA | Cleavage |
| ccp-miR399-6-5p | Cc02_g28660 | 2.5 | 15.622 | 1 | 21 | 3013 | 3033 | GGGCAACUAGUCCUCUGGCAG | UUGCCAGUGGACAAGUUGCUC | Translation |
| ccp-miR399a-3p | Cc02_g32810 | 3.0 | 16.976 | 1 | 21 | 680 | 700 | UGCCAAAGGAGAACGGCCCUG | CAUGGCUUUUCUUCUUUGGCA | Cleavage |
| ccp-miR399a-3p | Cc07_g19050 | 2.0 | 22.862 | 1 | 21 | 76 | 96 | UGCCAAAGGAGAACGGCCCUG | UAGGGCCUUUUUCCUUUGGUA | Cleavage |
| ccp-miR399a-3p | Cc08_g14930 | 3.0 | 19.839 | 1 | 21 | 1442 | 1463 | UGCCAAAGGAG-AACGGCCCUG | CAGGGCUGUUACUCUUUUGGCA | Cleavage |
| ccp-miR399f-3p | Cc01_g13140 | 3.0 | 19.636 | 1 | 20 | 1438 | 1457 | UGCCAAAGGAGAUUUGCCCG | CUGGUAAAACUCUUUUGGCA | Cleavage |
| ccp-miR399f-3p | Cc02_g13390 | 0.5 | 21.105 | 1 | 21 | 222 | 242 | UGCCAAAGGAGAUUUGCCCGG | UUGGGCAAAUCUCCUUUGGCA | Cleavage |
| ccp-miR399f-3p | Cc02_g13390 | 1.0 | 20.786 | 1 | 20 | 278 | 297 | UGCCAAAGGAGAUUUGCCCG | GGGGCAAAUCUCCUUUGGCA | Cleavage |
| ccp-miR399f-3p | Cc02_g22540 | 1.5 | 16.122 | 1 | 21 | 1232 | 1252 | UGCCAAAGGAGAUUUGCCCGG | UCUGGCAAAUCUCUUUUGGCA | Cleavage |
| ccp-miR399f-3p | Cc11_g04700 | 2.5 | 16.319 | 1 | 21 | 911 | 931 | UGCCAAAGGAGAUUUGCCCGG | UCUGGCGAAUUUCUUUUGGCA | Cleavage |
| ccp-miR399f-5p | Cc07_g10900 | 3.0 | 23.426 | 1 | 21 | 2319 | 2339 | GGGCAACUACUCCUUUGGCAG | CUGCAGAAGGGGUAGUUGUUC | Cleavage |
| ccp-miR399j-3p | Cc02_g13390 | 1.5 | 20.786 | 1 | 21 | 277 | 297 | UGCCAAAGGAGAGUUGCCCUA | UGGGGCAAAUCUCCUUUGGCA | Cleavage |
| ccp-miR399j-3p | Cc07_g10820 | 3.0 | 11.97 | 1 | 21 | 1343 | 1363 | UGCCAAAGGAGAGUUGCCCUA | UAGGGUAACUCUCUUUCGGUA | Cleavage |
| ccp-miR403-3p | Cc01_g07560 | 3.0 | 15.517 | 1 | 20 | 261 | 280 | UUAGAUUCACGCACAAACUC | UAAUCUGUGCGUGAAUCUAA | Cleavage |
| ccp-miR403-3p | Cc02_g33600 | 3.0 | 18.332 | 1 | 21 | 2414 | 2434 | UUAGAUUCACGCACAAACUCG | CAAAUUUGUGGGUGAAUCUAA | Translation |
| ccp-miR403-3p | Cc09_g06770 | 1.0 | 19.728 | 1 | 20 | 3699 | 3718 | UUAGAUUCACGCACAAACUC | GAGUUUGUACGUGAAUCUAA | Cleavage |
| ccp-miR403-3p | Cc10_g05710 | 3.0 | 19.321 | 1 | 20 | 1131 | 1150 | UUAGAUUCACGCACAAACUC | AAGUUUGCGGGUGAAUCUAA | Translation |
| ccp-miR403-5p | Cc00_g17350 | 3.0 | 9.512 | 1 | 20 | 1121 | 1140 | GUUUGUGCGUGAAUCUAACG | CUUUAGAUUUAUCCACAAAC | Cleavage |
| ccp-miR403a-3p | Cc02_g33600 | 3.0 | 18.332 | 1 | 21 | 2414 | 2434 | UUAGAUUCACGCACAAACCUG | CAAAUUUGUGGGUGAAUCUAA | Translation |
| ccp-miR403a-3p | Cc08_g07250 | 3.0 | 19.971 | 1 | 20 | 171 | 190 | UUAGAUUCACGCACAAACCU | UGUUUUGUGUGUGAAUCUGA | Cleavage |
| ccp-miR403a-3p | Cc09_g06770 | 3.0 | 19.728 | 1 | 20 | 3699 | 3718 | UUAGAUUCACGCACAAACCU | GAGUUUGUACGUGAAUCUAA | Cleavage |
| ccp-miR403a-3p | Cc10_g05710 | 3.0 | 19.321 | 1 | 20 | 1131 | 1150 | UUAGAUUCACGCACAAACCU | AAGUUUGCGGGUGAAUCUAA | Translation |
| ccp-miR403a-5p | Cc00_g17350 | 3.0 | 9.512 | 1 | 20 | 1121 | 1140 | GUUUGUGCGUGAAUCUAACG | CUUUAGAUUUAUCCACAAAC | Cleavage |
| ccp-miR408-3p | Cc07_g05370 | 3.0 | 15.609 | 1 | 20 | 357 | 376 | UGCUCUGCCUCGUCCCCGUC | GACGAAGAUGAGGCAGAGUA | Cleavage |
| ccp-miR408-3p | Cc09_g07900 | 3.0 | 11.828 | 1 | 21 | 210 | 230 | UGCUCUGCCUCGUCCCCGUCU | GGAUGGGGAUGAGGGGGAGCA | Cleavage |
| ccp-miR408-5p | Cc09_g05370 | 3.0 | 17.764 | 1 | 21 | 1246 | 1266 | CAGGGAAGAGGCAGUGCACGG | UCGUGAAUUGCUUCUUUCUUG | Cleavage |
| ccp-miR408b-3p | Cc00_g02380 | 3.0 | 16.017 | 1 | 20 | 1166 | 1185 | UGCUUUUCCCUUUUCCCUCU | AGAGGGAGAUGGAGAAAGCA | Translation |
| ccp-miR408b-3p | Cc00_g04690 | 3.0 | 11.483 | 1 | 20 | 14 | 33 | UGCUUUUCCCUUUUCCCUCU | AGAUGGAGAAGGGGAGAGUA | Cleavage |
| ccp-miR408b-3p | Cc00_g08750 | 2.5 | 11.58 | 1 | 20 | 54 | 73 | UGCUUUUCCCUUUUCCCUCU | AGAAGGAAAAGGGAAAAGGA | Cleavage |
| ccp-miR408b-3p | Cc00_g18010 | 3.0 | 11.488 | 1 | 20 | 14 | 33 | UGCUUUUCCCUUUUCCCUCU | AGAUGGAGAAGGGGAGAGUA | Cleavage |
| ccp-miR408b-3p | Cc00_g27900 | 3.0 | 12.726 | 1 | 20 | 1268 | 1287 | UGCUUUUCCCUUUUCCCUCU | AGAUGGAGGAGGGGGAAGCA | Cleavage |
| ccp-miR408b-3p | Cc00_g29160 | 2.5 | 6.075 | 1 | 21 | 53 | 73 | UGCUUUUCCCUUUUCCCUCUC | GGGAGGAAAAAGGGAAAAGCU | Cleavage |
| ccp-miR408b-3p | Cc00_g32890 | 3.0 | 11.483 | 1 | 20 | 14 | 33 | UGCUUUUCCCUUUUCCCUCU | AGAUGGAGAAGGGGAGAGUA | Cleavage |
| ccp-miR408b-3p | Cc01_g05700 | 2.5 | 13.71 | 1 | 21 | 976 | 996 | UGCUUUUCCCUUUUCCCUCUC | GAGGGGGAAGAGGGAAAGGUG | Cleavage |
| ccp-miR408b-3p | Cc01_g07960 | 3.0 | 18.93 | 1 | 20 | 1048 | 1067 | UGCUUUUCCCUUUUCCCUCU | AGAUGGAAGAGGGUAAAGCA | Cleavage |
| ccp-miR408b-3p | Cc01_g11900 | 3.0 | 14.558 | 1 | 20 | 284 | 303 | UGCUUUUCCCUUUUCCCUCU | AGAUAGGAGAGGGAAAAGCA | Cleavage |
| ccp-miR408b-3p | Cc01_g15500 | 3.0 | 16.464 | 1 | 20 | 10 | 29 | UGCUUUUCCCUUUUCCCUCU | AGUGGGGAAUGGGAGAAGCA | Translation |
| ccp-miR408b-3p | Cc01_g16500 | 2.5 | 7.755 | 1 | 20 | 1935 | 1955 | UGCUUUUCCCUUU-UCCCUCU | GGAGGGAGAAAGGGGAAAGCA | Cleavage |
| ccp-miR408b-3p | Cc01_g16520 | 3.0 | 9.01 | 1 | 20 | 1363 | 1382 | UGCUUUUCCCUUUUCCCUCU | AAAGGGAGAAGGAAAAGGCA | Cleavage |
| ccp-miR408b-3p | Cc02_g18660 | 3.0 | 16.436 | 1 | 20 | 256 | 275 | UGCUUUUCCCUUUUCCCUCU | AGGGGGAAGAAGGAGGAGCA | Translation |
| ccp-miR408b-3p | Cc02_g22520 | 3.0 | 17.078 | 1 | 20 | 762 | 781 | UGCUUUUCCCUUUUCCCUCU | AGAGGGCAAAAGGGAAGGCA | Translation |
| ccp-miR408b-3p | Cc02_g26450 | 3.0 | 10.172 | 1 | 20 | 14 | 33 | UGCUUUUCCCUUUUCCCUCU | AGAUGGAGAAGGGGAGAGUA | Cleavage |
| ccp-miR408b-3p | Cc02_g27010 | 3.0 | 23.423 | 1 | 20 | 444 | 463 | UGCUUUUCCCUUUUCCCUCU | AGAGGGAAAGGGGAAGGGGA | Cleavage |
| ccp-miR408b-3p | Cc02_g34720 | 3.0 | 10.172 | 1 | 20 | 14 | 33 | UGCUUUUCCCUUUUCCCUCU | AGAUGGAGAAGGGGAGAGUA | Cleavage |
| ccp-miR408b-3p | Cc02_g36730 | 2.5 | 9.602 | 1 | 20 | 1034 | 1053 | UGCUUUUCCCUUUUCCCUCU | AGAUGGAGAAGGGAAGAGUA | Cleavage |
| ccp-miR408b-3p | Cc03_g03640 | 3.0 | 10.207 | 1 | 20 | 14 | 33 | UGCUUUUCCCUUUUCCCUCU | AGAUGGAGAAGGGGAGAGUA | Cleavage |
| ccp-miR408b-3p | Cc03_g13460 | 3.0 | 11.483 | 1 | 20 | 14 | 33 | UGCUUUUCCCUUUUCCCUCU | AGAUGGAGAAGGGGAGAGUA | Cleavage |
| ccp-miR408b-3p | Cc04_g11380 | 3.0 | 17.779 | 1 | 21 | 1257 | 1277 | UGCUUUUCCCUUUUCCCUCUC | GAGAGGGAAAAUGUGGAAGCA | Translation |
| ccp-miR408b-3p | Cc04_g16220 | 3.0 | 20.807 | 1 | 21 | 1711 | 1731 | UGCUUUUCCCUUUUCCCUCUC | GAGAGGGAGGUGGAAAAAGCA | Translation |
| ccp-miR408b-3p | Cc06_g00920 | 2.5 | 7.335 | 1 | 20 | 1607 | 1626 | UGCUUUUCCCUUUUCCCUCU | GGAGGGAGAAGAGAGAAGCA | Translation |
| ccp-miR408b-3p | Cc06_g18110 | 3.0 | 14.932 | 1 | 21 | 3475 | 3495 | UGCUUUUCCCUUUUCCCUCUC | GGGAGGGAAAAAGUAAGAGCA | Translation |
| ccp-miR408b-3p | Cc07_g14050 | 3.0 | 14.655 | 1 | 20 | 978 | 997 | UGCUUUUCCCUUUUCCCUCU | AGGGGGAGAAGGGAAAGGGA | Cleavage |
| ccp-miR408b-3p | Cc07_g17240 | 3.0 | 14.181 | 1 | 21 | 970 | 990 | UGCUUUUCCCUUUUCCCUCUC | GGGGUGGAAAAGGGGGAAGCA | Cleavage |
| ccp-miR408b-3p | Cc07_g21430 | 3.0 | 16.754 | 1 | 20 | 2053 | 2073 | UGCUUUUC-CCUUUUCCCUCU | AGAGGGAAAAAGUGAAAAGCA | Translation |
| ccp-miR408b-3p | Cc08_g05340 | 2.5 | 14.682 | 1 | 21 | 1092 | 1113 | UGCUUUUCCCUUUUCC-CUCUC | GGGAGAGGAAAAGGGAAGAGCA | Cleavage |
| ccp-miR408b-3p | Cc08_g12310 | 2.5 | 16.733 | 1 | 20 | 5935 | 5954 | UGCUUUUCCCUUUUCCCUCU | UGAGGGCAAAGGGAAGAGCA | Cleavage |
| ccp-miR408b-3p | Cc09_g06820 | 2.5 | 16.837 | 1 | 21 | 1210 | 1230 | UGCUUUUCCCUUUUCCCUCUC | GAGAGGGAAAAAGGAAAAGUU | Translation |
| ccp-miR408b-3p | Cc09_g10320 | 2.5 | 18.152 | 1 | 20 | 2951 | 2970 | UGCUUUUCCCUUUUCCCUCU | GGAGGUAAAAGGGGGAAGCA | Cleavage |
| ccp-miR408b-3p | Cc11_g08230 | 2.0 | 11.604 | 1 | 20 | 2174 | 2192 | UGCUUUUCCCUUUUCCCUCU | AGAGGGAAAAGG-AAAAGCA | Cleavage |
| ccp-miR408b-3p | Cc11_g09530 | 2.0 | 15.085 | 1 | 20 | 721 | 740 | UGCUUUUCCCUUUUCCCUCU | GGAGGGAAAAAGGAAGAGCA | Translation |
| ccp-miR408b-5p | Cc00_g08790 | 3.0 | 12.769 | 1 | 20 | 1303 | 1322 | ACAGGGAAGAGGAACAGCAU | CUGCUGUUACUUUUCCUUGU | Cleavage |
| ccp-miR408b-5p | Cc01_g10640 | 2.5 | 20.443 | 1 | 20 | 1141 | 1160 | ACAGGGAAGAGGAACAGCAU | AUGAUGAUCCUCUUCUCUGU | Cleavage |
| ccp-miR408b-5p | Cc02_g28520 | 3.0 | 15.804 | 1 | 20 | 2296 | 2315 | ACAGGGAAGAGGAACAGCAU | AUGCAUUUUCUUUUCCCUGU | Cleavage |
| ccp-miR408b-5p | Cc02_g39230 | 3.0 | 15.256 | 1 | 20 | 1143 | 1162 | ACAGGGAAGAGGAACAGCAU | AUGCUCUAUCUCUUCCUUGU | Cleavage |
| ccp-miR408b-5p | Cc02_g39240 | 3.0 | 12.317 | 1 | 20 | 2216 | 2235 | ACAGGGAAGAGGAACAGCAU | AUUUUGCUCCUUUUCCCUGU | Cleavage |
| ccp-miR408b-5p | Cc02_g39670 | 3.0 | 16.143 | 1 | 20 | 1956 | 1975 | ACAGGGAAGAGGAACAGCAU | AAGCUGUUCUUCAUCCUUGU | Cleavage |
| ccp-miR408b-5p | Cc07_g11800 | 3.0 | 7.881 | 1 | 21 | 864 | 884 | ACAGGGAAGAGGAACAGCAUA | UAAGCUGUUCUUUUUUCUUGU | Cleavage |
| ccp-miR408b-5p | Cc08_g05640 | 2.5 | 16.735 | 1 | 21 | 1434 | 1454 | ACAGGGAAGAGGAACAGCAUA | UAUGUUAUUCUUCUUCUCUGU | Cleavage |
| ccp-miR4245-5p | Cc00_g01260 | 3.0 | 24.429 | 1 | 21 | 1767 | 1787 | ACAAAGUUUGAUCCUGACAAG | UUUGUCGGGUUCAUAUUUUGU | Cleavage |
| ccp-miR4245-5p | Cc00_g01900 | 3.0 | 18.148 | 1 | 20 | 901 | 920 | ACAAAGUUUGAUCCUGACAA | UUGCCGGGGUCACACUUUGU | Cleavage |
| ccp-miR4245-5p | Cc02_g05980 | 3.0 | 22.149 | 1 | 21 | 2906 | 2926 | ACAAAGUUUGAUCCUGACAAG | CUUGGCAAGACCAAACUUUGU | Translation |
| ccp-miR4245-5p | Cc06_g17380 | 2.5 | 13.368 | 1 | 20 | 1468 | 1487 | ACAAAGUUUGAUCCUGACAA | UUGUCAGAAUCAAGCUUUGG | Cleavage |
| ccp-miR4245-5p | Cc08_g10560 | 3.0 | 15.784 | 1 | 21 | 685 | 705 | ACAAAGUUUGAUCCUGACAAG | UUUUUCUGGAUCCAACUUUGU | Translation |
| ccp-miR4245-5p | Cc10_g00200 | 3.0 | 14.739 | 1 | 20 | 2157 | 2176 | ACAAAGUUUGAUCCUGACAA | UGGUUAGGAUCAAUCUUUGU | Cleavage |
| ccp-miR4246-5p | Cc00_g00640 | 3.0 | 12.477 | 1 | 20 | 818 | 837 | AAAUCCAAUUUUGAUUGUUU | AAACUGUCAAAAUUGUAUUU | Cleavage |
| ccp-miR4246-5p | Cc02_g10120 | 3.0 | 8.69 | 1 | 22 | 1092 | 1113 | AAAUCCAAUUUUGAUUGUUUAA | UUAAACCAUUAAAAUUGGAUAU | Cleavage |
| ccp-miR4246-5p | Cc02_g10290 | 3.0 | 10.328 | 1 | 21 | 1640 | 1660 | AAAUCCAAUUUUGAUUGUUUA | UUAACAAUUAAAAUUGAAUUU | Cleavage |
| ccp-miR4246-5p | Cc02_g17970 | 3.0 | 15.594 | 1 | 22 | 783 | 804 | AAAUCCAAUUUUGAUUGUUUAA | UGGAACAAAUAAGGUUGGAUUU | Cleavage |
| ccp-miR4246-5p | Cc02_g36450 | 3.0 | 19.542 | 1 | 22 | 549 | 570 | AAAUCCAAUUUUGAUUGUUUAA | UUCAAUAUUCAAAGUUGGAUUU | Cleavage |
| ccp-miR4246-5p | Cc03_g03950 | 2.5 | 15.775 | 1 | 20 | 1873 | 1892 | AAAUCCAAUUUUGAUUGUUU | AAACAGUCAGAAUUGGAUGU | Cleavage |
| ccp-miR4246-5p | Cc03_g10770 | 2.5 | 12.298 | 1 | 20 | 904 | 923 | AAAUCCAAUUUUGAUUGUUU | AAACAAUCAAAACUUGAUUU | Cleavage |
| ccp-miR4246-5p | Cc04_g06790 | 3.0 | 19.189 | 1 | 20 | 238 | 257 | AAAUCCAAUUUUGAUUGUUU | AAGCCGUCGAAAUUGGGUUU | Cleavage |
| ccp-miR4246-5p | Cc04_g15750 | 2.5 | 18.572 | 1 | 20 | 4110 | 4129 | AAAUCCAAUUUUGAUUGUUU | AAAGAAUUGGAAUUGGAUUU | Cleavage |
| ccp-miR4246-5p | Cc07_g19780 | 3.0 | 23.239 | 1 | 22 | 1776 | 1797 | AAAUCCAAUUUUGAUUGUUUAA | UCGAUCUAUCAAGAUUGGAUUU | Cleavage |
| ccp-miR4246-5p | Cc08_g00080 | 3.0 | 13.163 | 1 | 20 | 175 | 194 | AAAUCCAAUUUUGAUUGUUU | AAACAAACAUGGUUGGAUUU | Translation |
| ccp-miR4246-5p | Cc08_g12290 | 3.0 | 17.394 | 1 | 20 | 576 | 595 | AAAUCCAAUUUUGAUUGUUU | AUAUAAUCAAAAUUGGAUGU | Cleavage |
| ccp-miR426-1-5p | Cc01_g18480 | 3.0 | 18.144 | 1 | 21 | 1511 | 1531 | UUUUGGAAAUUUGUAAUUACU | AGUGGUUGCAAAUAUUCAAAA | Cleavage |
| ccp-miR426-1-5p | Cc02_g13000 | 3.0 | 12.384 | 1 | 20 | 857 | 876 | UUUUGGAAAUUUGUAAUUAC | GAAAUUAAAAAUUUUCGAAA | Cleavage |
| ccp-miR426-1-5p | Cc04_g05870 | 2.0 | 19.826 | 1 | 21 | 1614 | 1634 | UUUUGGAAAUUUGUAAUUACU | AGUAAUUACAGAUCUCCAAGA | Cleavage |
| ccp-miR426-1-5p | Cc06_g01040 | 2.0 | 12.224 | 1 | 21 | 517 | 537 | UUUUGGAAAUUUGUAAUUACU | GGUAAUUAAAAAUUUUCAGAA | Cleavage |
| ccp-miR426-1-5p | Cc07_g10020 | 2.5 | 4.823 | 1 | 20 | 113 | 132 | UUUUGGAAAUUUGUAAUUAC | GCAAUUAUAAAUUUUCAAAG | Cleavage |
| ccp-miR426-2-5p | Cc01_g01180 | 3.0 | 18.108 | 1 | 21 | 3223 | 3243 | UUUUGGAAAUUUCUCCAUACA | UGGGUGGAGAAGUUUCCAAAC | Cleavage |
| ccp-miR426-2-5p | Cc01_g21590 | 3.0 | 23.396 | 1 | 21 | 1851 | 1871 | UUUUGGAAAUUUCUCCAUACA | UGUAUUGGGAGUUUUCCAAAA | Translation |
| ccp-miR426-2-5p | Cc02_g26510 | 3.0 | 13.979 | 1 | 20 | 1180 | 1199 | UUUUGGAAAUUUCUCCAUAC | AAAUGGAGAAAUAUCCAAAA | Cleavage |
| ccp-miR426-2-5p | Cc04_g12940 | 3.0 | 14.878 | 1 | 21 | 212 | 232 | UUUUGGAAAUUUCUCCAUACA | UGGAUGGAGAAAUUGCCAGAA | Cleavage |
| ccp-miR426-2-5p | Cc05_g07610 | 3.0 | 14.771 | 1 | 20 | 535 | 554 | UUUUGGAAAUUUCUCCAUAC | GGAUGGAAAUAUUUCCAAAA | Translation |
| ccp-miR426-2-5p | Cc05_g09450 | 3.0 | 15.403 | 1 | 21 | 1004 | 1024 | UUUUGGAAAUUUCUCCAUACA | UGUUUGGAGAAGUUUUCAAGG | Cleavage |
| ccp-miR426-2-5p | Cc05_g10580 | 3.0 | 14.35 | 1 | 21 | 1307 | 1327 | UUUUGGAAAUUUCUCCAUACA | UUUAUUGAGAAAUUUCCAAAU | Cleavage |
| ccp-miR426-2-5p | Cc09_g02530 | 3.0 | 15.165 | 1 | 20 | 564 | 583 | UUUUGGAAAUUUCUCCAUAC | GUAUGGGAAAGUUUCUAAGA | Cleavage |
| ccp-miR426-2-5p | Cc10_g00740 | 3.0 | 21.171 | 1 | 21 | 464 | 483 | UUUUGGAAAUUUCUCCAUACA | UGUA-GGAGAAAUUUUUAAAA | Cleavage |
| ccp-miR426-2-5p | Cc10_g04140 | 2.5 | 18.761 | 1 | 20 | 347 | 366 | UUUUGGAAAUUUCUCCAUAC | GUAUGGAGAAGUCUUCAAGA | Cleavage |
| ccp-miR426-2-5p | Cc10_g07970 | 3.0 | 19.596 | 1 | 20 | 865 | 884 | UUUUGGAAAUUUCUCCAUAC | GUUUGGAGAAGUUUCCAAGC | Cleavage |
| ccp-miR426-2-5p | Cc10_g07980 | 3.0 | 18.373 | 1 | 20 | 721 | 740 | UUUUGGAAAUUUCUCCAUAC | GUUUGGAGAAGUUUCCAAGC | Cleavage |
| ccp-miR426-2-5p | Cc10_g11030 | 3.0 | 18.405 | 1 | 20 | 928 | 947 | UUUUGGAAAUUUCUCCAUAC | GAAUGGAGAAGUUUUCAAGG | Cleavage |
| ccp-miR477i-1-5p | Cc01_g19160 | 2.5 | 15.051 | 1 | 20 | 720 | 739 | ACUCUCCCUCAAGGGCUUCC | AGAGGCUUUUGAGGGAGAGU | Cleavage |
| ccp-miR477i-1-5p | Cc02_g26280 | 2.0 | 17.298 | 1 | 21 | 577 | 597 | ACUCUCCCUCAAGGGCUUCCC | GGGAAGCCAUUGAGGGAGAGA | Cleavage |
| ccp-miR477i-1-5p | Cc07_g02740 | 3.0 | 24.339 | 1 | 20 | 1955 | 1974 | ACUCUCCCUCAAGGGCUUCC | GGAAGCUGUUGAUGGGGAGU | Cleavage |
| ccp-miR477i-2-3p | Cc04_g07550 | 3.0 | 16.8 | 1 | 21 | 2604 | 2624 | GAGGCGCUUGGGGAGAGUGGA | UCCUCCCUUCUCAAGCGCCUC | Cleavage |
| ccp-miR477i-2-3p | Cc06_g12040 | 3.0 | 7.912 | 1 | 21 | 170 | 190 | GAGGCGCUUGGGGAGAGUGGA | UCUUCCCUUCCCAAGCGCCUC | Cleavage |
| ccp-miR477i-2-3p | Cc08_g09950 | 3.0 | 12.457 | 1 | 20 | 117 | 136 | GAGGCGCUUGGGGAGAGUGG | CAAUUCUCCUCAUGCGCCUC | Cleavage |
| ccp-miR477i-2-5p | Cc01_g19160 | 2.5 | 15.051 | 1 | 20 | 720 | 739 | ACUCUCCCUCAAGGGCUUCC | AGAGGCUUUUGAGGGAGAGU | Cleavage |
| ccp-miR477i-2-5p | Cc02_g26280 | 2.0 | 17.298 | 1 | 20 | 578 | 597 | ACUCUCCCUCAAGGGCUUCC | GGAAGCCAUUGAGGGAGAGA | Cleavage |
| ccp-miR477i-2-5p | Cc07_g02740 | 3.0 | 24.339 | 1 | 20 | 1955 | 1974 | ACUCUCCCUCAAGGGCUUCC | GGAAGCUGUUGAUGGGGAGU | Cleavage |
| ccp-miR482a-1-3p | Cc00_g09350 | 3.0 | 15.875 | 1 | 20 | 2035 | 2054 | GGAAUUGGUGGAUUGUCAAG | UUUGAUAAUUCACCAGUUUU | Cleavage |
| ccp-miR482a-1-3p | Cc00_g18200 | 2.5 | 18.572 | 1 | 20 | 774 | 793 | GGAAUUGGUGGAUUGUCAAG | CUUGAUAAACAACCAAUUCC | Translation |
| ccp-miR482a-1-3p | Cc00_g18490 | 3.0 | 15.026 | 1 | 20 | 412 | 431 | GGAAUUGGUGGAUUGUCAAG | CUUGAAAAUCCAGCAAUUUU | Cleavage |
| ccp-miR482a-1-3p | Cc01_g04540 | 3.0 | 12.697 | 1 | 20 | 988 | 1007 | GGAAUUGGUGGAUUGUCAAG | UUUGAUAGUCCACCAAUGCC | Cleavage |
| ccp-miR482a-1-3p | Cc01_g04560 | 3.0 | 14.061 | 1 | 20 | 1198 | 1217 | GGAAUUGGUGGAUUGUCAAG | UUUGAUAGUCCACCAAUGCC | Cleavage |
| ccp-miR482a-1-3p | Cc05_g03610 | 3.0 | 15.142 | 1 | 20 | 277 | 296 | GGAAUUGGUGGAUUGUCAAG | UUUGAUAAUUCACCAGUUUU | Cleavage |
| ccp-miR482a-1-3p | Cc07_g00490 | 3.0 | 9.992 | 1 | 20 | 367 | 386 | GGAAUUGGUGGAUUGUCAAG | AUUCAUAAUCCAUCAAUUCC | Cleavage |
| ccp-miR482a-1-3p | Cc08_g05880 | 3.0 | 14.249 | 1 | 20 | 1984 | 2003 | GGAAUUGGUGGAUUGUCAAG | UUUGAUAAUCCAUCAAUGCC | Cleavage |
| ccp-miR482a-1-3p | Cc08_g09120 | 3.0 | 9.678 | 1 | 20 | 79 | 98 | GGAAUUGGUGGAUUGUCAAG | CUUGACAAAUUAUUAAUUCC | Cleavage |
| ccp-miR482a-1-3p | Cc09_g02230 | 2.5 | 14.418 | 1 | 20 | 154 | 173 | GGAAUUGGUGGAUUGUCAAG | CUCUACAAUCCACCAGUUCC | Cleavage |
| ccp-miR482a-2-3p | Cc00_g09140 | 3.0 | 23.596 | 1 | 20 | 535 | 554 | UUUCCUAGCCCGCCCAUUCC | GGCAUGGGUGGUUUAGGAAA | Translation |
| ccp-miR482a-2-3p | Cc00_g19390 | 3.0 | 19.189 | 1 | 20 | 619 | 638 | UUUCCUAGCCCGCCCAUUCC | GGCAUGGGUGGUUUAGGAAA | Translation |
| ccp-miR482a-2-3p | Cc00_g19640 | 3.0 | 19.691 | 1 | 20 | 588 | 607 | UUUCCUAGCCCGCCCAUUCC | GGCAUGGGUGGUUUAGGAAA | Translation |
| ccp-miR482a-2-3p | Cc00_g19650 | 3.0 | 24.945 | 1 | 20 | 427 | 446 | UUUCCUAGCCCGCCCAUUCC | GGCAUGGGUGGUUUAGGAAA | Translation |
| ccp-miR482a-2-3p | Cc00_g23630 | 3.0 | 24.534 | 1 | 20 | 565 | 584 | UUUCCUAGCCCGCCCAUUCC | GGCAUGGGUGGUUUAGGAAA | Translation |
| ccp-miR482a-2-3p | Cc00_g25160 | 3.0 | 23.521 | 1 | 20 | 604 | 623 | UUUCCUAGCCCGCCCAUUCC | GGCAUGGGUGGUUUAGGAAA | Translation |
| ccp-miR482a-2-3p | Cc00_g27620 | 3.0 | 19.54 | 1 | 20 | 575 | 594 | UUUCCUAGCCCGCCCAUUCC | GGCAUGGGUGGUUUAGGAAA | Translation |
| ccp-miR482a-2-3p | Cc00_g30510 | 3.0 | 19.103 | 1 | 20 | 475 | 494 | UUUCCUAGCCCGCCCAUUCC | GGCAUGGGUGGUUUAGGAAA | Translation |
| ccp-miR482a-2-3p | Cc00_g35040 | 1.5 | 19.194 | 1 | 22 | 620 | 641 | UUUCCUAGCCCGCCCAUUCCUA | UAGGAAUGGGCGGGAUAGGGAA | Cleavage |
| ccp-miR482a-2-3p | Cc01_g00420 | 3.0 | 23.866 | 1 | 20 | 1675 | 1694 | UUUCCUAGCCCGCCCAUUCC | GGCAUGGGUGGUUUAGGAAA | Translation |
| ccp-miR482a-2-3p | Cc01_g00440 | 3.0 | 20.387 | 1 | 20 | 553 | 572 | UUUCCUAGCCCGCCCAUUCC | GGCAUGGGUGGUUUAGGAAA | Translation |
| ccp-miR482a-2-3p | Cc01_g02020 | 3.0 | 19.071 | 1 | 20 | 611 | 630 | UUUCCUAGCCCGCCCAUUCC | GGCAUGGGUGGUUUAGGAAA | Translation |
| ccp-miR482a-2-3p | Cc01_g02870 | 3.0 | 18.385 | 1 | 20 | 535 | 554 | UUUCCUAGCCCGCCCAUUCC | GGCAUGGGUGGUUUAGGAAA | Translation |
| ccp-miR482a-2-3p | Cc01_g05280 | 3.0 | 22.371 | 1 | 20 | 586 | 605 | UUUCCUAGCCCGCCCAUUCC | GGCAUGGGUGGUUUAGGAAA | Translation |
| ccp-miR482a-2-3p | Cc03_g06210 | 3.0 | 20.895 | 1 | 20 | 586 | 605 | UUUCCUAGCCCGCCCAUUCC | GGGAUGGGUGGUCUGGGGAA | Translation |
| ccp-miR482a-2-3p | Cc03_g14560 | 3.0 | 21.876 | 1 | 22 | 548 | 569 | UUUCCUAGCCCGCCCAUUCCUA | UAGGAAUGGGCGGGAUCGGGAA | Cleavage |
| ccp-miR482a-2-3p | Cc03_g14580 | 3.0 | 24.584 | 1 | 22 | 629 | 650 | UUUCCUAGCCCGCCCAUUCCUA | UGGGAAUGGGUGGAUUGGGGAA | Translation |
| ccp-miR482a-2-3p | Cc04_g12940 | 3.0 | 24.478 | 1 | 21 | 45 | 65 | UUUCCUAGCCCGCCCAUUCCU | AGGCAUGGGAGGGUUGGGAAA | Cleavage |
| ccp-miR482a-2-3p | Cc06_g11650 | 3.0 | 14.656 | 1 | 20 | 520 | 539 | UUUCCUAGCCCGCCCAUUCC | GGAAUUGGUGGGAUAGGGAA | Cleavage |
| ccp-miR482a-2-3p | Cc06_g16160 | 2.5 | 24.349 | 1 | 20 | 595 | 614 | UUUCCUAGCCCGCCCAUUCC | GGGAUGGGGGGGUUGGGAAA | Cleavage |
| ccp-miR482a-2-3p | Cc06_g16170 | 3.0 | 22.903 | 1 | 20 | 529 | 548 | UUUCCUAGCCCGCCCAUUCC | GGGAUGGGUGGCUUGGGAAA | Translation |
| ccp-miR482a-2-3p | Cc06_g17030 | 3.0 | 18.791 | 1 | 22 | 887 | 908 | UUUCCUAGCCCGCCCAUUCCUA | UUGGAGUAGGUGGGAUAGGAAA | Cleavage |
| ccp-miR482a-2-3p | Cc06_g20740 | 3.0 | 19.74 | 1 | 20 | 580 | 599 | UUUCCUAGCCCGCCCAUUCC | GGGAUGGGGGGCUUAGGAAA | Translation |
| ccp-miR482a-2-3p | Cc08_g13260 | 3.0 | 17.953 | 1 | 20 | 924 | 943 | UUUCCUAGCCCGCCCAUUCC | GGAAAGGGAAGGCUAGGAAA | Translation |
| ccp-miR482a-2-3p | Cc11_g05380 | 3.0 | 16.864 | 1 | 22 | 422 | 443 | UUUCCUAGCCCGCCCAUUCCUA | UGGGAAUGGGAGGGCUCGGGAA | Cleavage |
| ccp-miR482a-2-3p | Cc11_g10450 | 3.0 | 16.533 | 1 | 20 | 893 | 912 | UUUCCUAGCCCGCCCAUUCC | GGAAUGCUGGGGCUAGGAAA | Cleavage |
| ccp-miR482a-2-5p | Cc00_g19450 | 3.0 | 23.165 | 1 | 22 | 1637 | 1658 | GAAGUUUUGGGAAUGGGCUGCU | GGGAAUUCAUUCCCAAAACUUC | Cleavage |
| ccp-miR482a-2-5p | Cc01_g19540 | 3.0 | 18.172 | 1 | 21 | 1912 | 1932 | GAAGUUUUGGGAAUGGGCUGC | GCAGCCUGUUCUCACAACUUC | Cleavage |
| ccp-miR482a-2-5p | Cc02_g36190 | 3.0 | 9.908 | 1 | 22 | 1987 | 2008 | GAAGUUUUGGGAAUGGGCUGCU | AUCAGCCACUGCCCAAAACUUC | Cleavage |
| ccp-miR482a-2-5p | Cc04_g05520 | 3.0 | 12.394 | 1 | 22 | 391 | 412 | GAAGUUUUGGGAAUGGGCUGCU | AGCACCCCAUUGCUAAAGCUUC | Translation |
| ccp-miR482a-2-5p | Cc06_g13670 | 3.0 | 13.936 | 1 | 22 | 693 | 714 | GAAGUUUUGGGAAUGGGCUGCU | AGUGGGCCAUUUUCAAAACUUC | Cleavage |
| ccp-miR482a-2-5p | Cc07_g04930 | 2.5 | 17.826 | 1 | 22 | 657 | 678 | GAAGUUUUGGGAAUGGGCUGCU | GGCAGCCCAUGUUCAGAACUUC | Cleavage |
| ccp-miR482a-2-5p | Cc08_g00190 | 2.0 | 15.752 | 1 | 20 | 2173 | 2192 | GAAGUUUUGGGAAUGGGCUG | CAGUGCAUUCCCAAAACUUU | Cleavage |
| ccp-miR482a-2-5p | Cc08_g00380 | 3.0 | 20.06 | 1 | 20 | 907 | 926 | GAAGUUUUGGGAAUGGGCUG | CAGCGCGUCCUCAAAACUUC | Cleavage |
| ccp-miR482a-2-5p | Cc08_g01080 | 3.0 | 16.628 | 1 | 20 | 294 | 313 | GAAGUUUUGGGAAUGGGCUG | CAAUCCAUUCACAAAGCUUC | Translation |
| ccp-miR482a-2-5p | Cc08_g01730 | 2.5 | 4.464 | 1 | 20 | 513 | 532 | GAAGUUUUGGGAAUGGGCUG | CUGCCCAUUACCAAAAUUUC | Translation |
| ccp-miR482a-2-5p | Cc09_g05470 | 3.0 | 13.832 | 1 | 22 | 496 | 517 | GAAGUUUUGGGAAUGGGCUGCU | AGUAGUUCAUUUCCCAAACUUC | Cleavage |
| ccp-miR482a-3-3p | Cc00_g12170 | 3.0 | 21.027 | 1 | 22 | 1498 | 1519 | UCUUCCCUAGUCCUCCCAUCCC | GGGGAGGGGGGAUUGGGGAAGA | Cleavage |
| ccp-miR482a-3-3p | Cc01_g16600 | 3.0 | 14.621 | 1 | 20 | 431 | 450 | UCUUCCCUAGUCCUCCCAUC | GAUAGGAAGAUUGGGGAAGA | Cleavage |
| ccp-miR482a-3-3p | Cc02_g32490 | 3.0 | 17.689 | 1 | 22 | 640 | 661 | UCUUCCCUAGUCCUCCCAUCCC | GGCAUGGGAGGCAUAGGGAAGA | Translation |
| ccp-miR482a-3-3p | Cc02_g33930 | 3.0 | 24.226 | 1 | 22 | 1264 | 1285 | UCUUCCCUAGUCCUCCCAUCCC | GAGAUGGGAGGGUUAGGAAAGG | Cleavage |
| ccp-miR482a-3-3p | Cc03_g04520 | 3.0 | 20.635 | 1 | 22 | 496 | 517 | UCUUCCCUAGUCCUCCCAUCCC | GGGAUGGGCGGUCUCGGGAAGA | Translation |
| ccp-miR482a-3-3p | Cc03_g04730 | 3.0 | 20.635 | 1 | 22 | 595 | 616 | UCUUCCCUAGUCCUCCCAUCCC | GGGAUGGGCGGUCUCGGGAAGA | Translation |
| ccp-miR482a-3-3p | Cc03_g06210 | 2.5 | 21.024 | 1 | 22 | 586 | 607 | UCUUCCCUAGUCCUCCCAUCCC | GGGAUGGGUGGUCUGGGGAAGA | Translation |
| ccp-miR482a-3-3p | Cc07_g09980 | 2.5 | 18.184 | 1 | 22 | 562 | 583 | UCUUCCCUAGUCCUCCCAUCCC | GGAAUGGGAGGAGUGGGGAAGA | Translation |
| ccp-miR482a-3-3p | Cc07_g17270 | 3.0 | 19.005 | 1 | 22 | 1753 | 1774 | UCUUCCCUAGUCCUCCCAUCCC | GGGAUGGGGGGACUCGGUAAGA | Cleavage |
| ccp-miR482a-3-3p | Cc10_g05860 | 3.0 | 18.864 | 1 | 20 | 1210 | 1229 | UCUUCCCUAGUCCUCCCAUC | GAUGGGUGGACUAUGGAGGA | Cleavage |
| ccp-miR482a-3-3p | Cc11_g04950 | 2.5 | 18.394 | 1 | 22 | 322 | 343 | UCUUCCCUAGUCCUCCCAUCCC | GGAAUGGGGGGACUCGGGAAGA | Cleavage |
| ccp-miR482a-3-3p | Cc11_g05380 | 2.5 | 16.731 | 1 | 22 | 424 | 445 | UCUUCCCUAGUCCUCCCAUCCC | GGAAUGGGAGGGCUCGGGAAGA | Cleavage |
| ccp-miR482a-3-3p | Cc11_g11060 | 3.0 | 10.657 | 1 | 20 | 528 | 547 | UCUUCCCUAGUCCUCCCAUC | CACGGGAGGAUUGGGGAAGA | Cleavage |
| ccp-miR482a-3-5p | Cc00_g04680 | 3.0 | 16.23 | 1 | 22 | 1405 | 1426 | GAUGGGUGACCGGGGAAGAUUU | GAACUUUCCCUGGUCACCCAUG | Cleavage |
| ccp-miR482a-3-5p | Cc01_g21840 | 3.0 | 18.186 | 1 | 21 | 2119 | 2139 | GAUGGGUGACCGGGGAAGAUU | AUUCUUCCCUGGUCAUUCAUU | Cleavage |
| ccp-miR482a-3-5p | Cc02_g17590 | 3.0 | 13.27 | 1 | 20 | 166 | 185 | GAUGGGUGACCGGGGAAGAU | UUCCUCCCCGGUCAUCUAUC | Cleavage |
| ccp-miR482a-3-5p | Cc02_g24000 | 2.5 | 20.056 | 1 | 22 | 46 | 67 | GAUGGGUGACCGGGGAAGAUUU | AGCUCUUCCCGGGUCAUCCAUC | Cleavage |
| ccp-miR482a-4-3p | Cc00_g12170 | 3.0 | 21.027 | 1 | 22 | 1498 | 1519 | UCUUCCCUAGUCCUCCCAUCCC | GGGGAGGGGGGAUUGGGGAAGA | Cleavage |
| ccp-miR482a-4-3p | Cc01_g16600 | 3.0 | 14.621 | 1 | 20 | 431 | 450 | UCUUCCCUAGUCCUCCCAUC | GAUAGGAAGAUUGGGGAAGA | Cleavage |
| ccp-miR482a-4-3p | Cc02_g32490 | 3.0 | 17.689 | 1 | 22 | 640 | 661 | UCUUCCCUAGUCCUCCCAUCCC | GGCAUGGGAGGCAUAGGGAAGA | Translation |
| ccp-miR482a-4-3p | Cc02_g33930 | 3.0 | 24.226 | 1 | 22 | 1264 | 1285 | UCUUCCCUAGUCCUCCCAUCCC | GAGAUGGGAGGGUUAGGAAAGG | Cleavage |
| ccp-miR482a-4-3p | Cc03_g04520 | 3.0 | 20.635 | 1 | 22 | 496 | 517 | UCUUCCCUAGUCCUCCCAUCCC | GGGAUGGGCGGUCUCGGGAAGA | Translation |
| ccp-miR482a-4-3p | Cc03_g04730 | 3.0 | 20.635 | 1 | 22 | 595 | 616 | UCUUCCCUAGUCCUCCCAUCCC | GGGAUGGGCGGUCUCGGGAAGA | Translation |
| ccp-miR482a-4-3p | Cc03_g06210 | 2.5 | 21.024 | 1 | 22 | 586 | 607 | UCUUCCCUAGUCCUCCCAUCCC | GGGAUGGGUGGUCUGGGGAAGA | Translation |
| ccp-miR482a-4-3p | Cc07_g09980 | 2.5 | 18.184 | 1 | 22 | 562 | 583 | UCUUCCCUAGUCCUCCCAUCCC | GGAAUGGGAGGAGUGGGGAAGA | Translation |
| ccp-miR482a-4-3p | Cc07_g17270 | 3.0 | 19.005 | 1 | 22 | 1753 | 1774 | UCUUCCCUAGUCCUCCCAUCCC | GGGAUGGGGGGACUCGGUAAGA | Cleavage |
| ccp-miR482a-4-3p | Cc10_g05860 | 3.0 | 18.864 | 1 | 20 | 1210 | 1229 | UCUUCCCUAGUCCUCCCAUC | GAUGGGUGGACUAUGGAGGA | Cleavage |
| ccp-miR482a-4-3p | Cc11_g04950 | 2.5 | 18.394 | 1 | 22 | 322 | 343 | UCUUCCCUAGUCCUCCCAUCCC | GGAAUGGGGGGACUCGGGAAGA | Cleavage |
| ccp-miR482a-4-3p | Cc11_g05380 | 2.5 | 16.731 | 1 | 22 | 424 | 445 | UCUUCCCUAGUCCUCCCAUCCC | GGAAUGGGAGGGCUCGGGAAGA | Cleavage |
| ccp-miR482a-4-3p | Cc11_g11060 | 3.0 | 10.657 | 1 | 20 | 528 | 547 | UCUUCCCUAGUCCUCCCAUC | CACGGGAGGAUUGGGGAAGA | Cleavage |
| ccp-miR482a-4-5p | Cc02_g24000 | 3.0 | 20.056 | 1 | 22 | 46 | 67 | GAUGGGUGACUCGGGCAGAUUU | AGCUCUUCCCGGGUCAUCCAUC | Cleavage |
| ccp-miR482a-4-5p | Cc10_g05950 | 2.5 | 16.428 | 1 | 22 | 44 | 65 | GAUGGGUGACUCGGGCAGAUUU | GGAUCUUCUCGAGUCAUCCAUU | Cleavage |
| ccp-miR482a-5-3p | Cc00_g12170 | 3.0 | 21.027 | 1 | 22 | 1498 | 1519 | UCUUCCCUAGUCCUCCCAUCCC | GGGGAGGGGGGAUUGGGGAAGA | Cleavage |
| ccp-miR482a-5-3p | Cc01_g16600 | 3.0 | 14.621 | 1 | 20 | 431 | 450 | UCUUCCCUAGUCCUCCCAUC | GAUAGGAAGAUUGGGGAAGA | Cleavage |
| ccp-miR482a-5-3p | Cc02_g32490 | 3.0 | 17.689 | 1 | 22 | 640 | 661 | UCUUCCCUAGUCCUCCCAUCCC | GGCAUGGGAGGCAUAGGGAAGA | Translation |
| ccp-miR482a-5-3p | Cc02_g33930 | 3.0 | 24.226 | 1 | 22 | 1264 | 1285 | UCUUCCCUAGUCCUCCCAUCCC | GAGAUGGGAGGGUUAGGAAAGG | Cleavage |
| ccp-miR482a-5-3p | Cc03_g04520 | 3.0 | 20.635 | 1 | 22 | 496 | 517 | UCUUCCCUAGUCCUCCCAUCCC | GGGAUGGGCGGUCUCGGGAAGA | Translation |
| ccp-miR482a-5-3p | Cc03_g04730 | 3.0 | 20.635 | 1 | 22 | 595 | 616 | UCUUCCCUAGUCCUCCCAUCCC | GGGAUGGGCGGUCUCGGGAAGA | Translation |
| ccp-miR482a-5-3p | Cc03_g06210 | 2.5 | 21.024 | 1 | 22 | 586 | 607 | UCUUCCCUAGUCCUCCCAUCCC | GGGAUGGGUGGUCUGGGGAAGA | Translation |
| ccp-miR482a-5-3p | Cc07_g09980 | 2.5 | 18.184 | 1 | 22 | 562 | 583 | UCUUCCCUAGUCCUCCCAUCCC | GGAAUGGGAGGAGUGGGGAAGA | Translation |
| ccp-miR482a-5-3p | Cc07_g17270 | 3.0 | 19.005 | 1 | 22 | 1753 | 1774 | UCUUCCCUAGUCCUCCCAUCCC | GGGAUGGGGGGACUCGGUAAGA | Cleavage |
| ccp-miR482a-5-3p | Cc10_g05860 | 3.0 | 18.864 | 1 | 20 | 1210 | 1229 | UCUUCCCUAGUCCUCCCAUC | GAUGGGUGGACUAUGGAGGA | Cleavage |
| ccp-miR482a-5-3p | Cc11_g04950 | 2.5 | 18.394 | 1 | 22 | 322 | 343 | UCUUCCCUAGUCCUCCCAUCCC | GGAAUGGGGGGACUCGGGAAGA | Cleavage |
| ccp-miR482a-5-3p | Cc11_g05380 | 2.5 | 16.731 | 1 | 22 | 424 | 445 | UCUUCCCUAGUCCUCCCAUCCC | GGAAUGGGAGGGCUCGGGAAGA | Cleavage |
| ccp-miR482a-5-3p | Cc11_g11060 | 3.0 | 10.657 | 1 | 20 | 528 | 547 | UCUUCCCUAGUCCUCCCAUC | CACGGGAGGAUUGGGGAAGA | Cleavage |
| ccp-miR482a-5-5p | Cc00_g04680 | 2.5 | 16.23 | 1 | 22 | 1405 | 1426 | GAUGGGUGACUGGGGAAGGUUU | GAACUUUCCCUGGUCACCCAUG | Cleavage |
| ccp-miR482a-5-5p | Cc01_g16220 | 3.0 | 10.355 | 1 | 21 | 238 | 258 | GAUGGGUGACUGGGGAAGGUU | GACCCUCCUCAGUAACUCAUC | Cleavage |
| ccp-miR482a-5-5p | Cc02_g08180 | 2.5 | 19.893 | 1 | 22 | 760 | 781 | GAUGGGUGACUGGGGAAGGUUU | AAGCCUUUCCUAGUCGCCCAUU | Cleavage |
| ccp-miR482a-5-5p | Cc04_g14370 | 3.0 | 24.044 | 1 | 22 | 798 | 819 | GAUGGGUGACUGGGGAAGGUUU | AUACUCUUCCCAGUCAUCUAUC | Cleavage |
| ccp-miR482a-5-5p | Cc08_g08530 | 3.0 | 17.582 | 1 | 20 | 460 | 479 | GAUGGGUGACUGGGGAAGGU | UCCAUUUCCAGUCACCCAUC | Cleavage |
| ccp-miR482a-5-5p | Cc10_g05950 | 3.0 | 16.428 | 1 | 22 | 44 | 65 | GAUGGGUGACUGGGGAAGGUUU | GGAUCUUCUCGAGUCAUCCAUU | Cleavage |
| ccp-miR482a-5-5p | Cc11_g01710 | 3.0 | 20.103 | 1 | 20 | 1070 | 1089 | GAUGGGUGACUGGGGAAGGU | UCCUUCCACAGUCACCCAUG | Cleavage |
| ccp-miR482b-1-3p | Cc00_g05750 | 3.0 | 21.964 | 1 | 19 | 699 | 718 | CUUCCCAAAC-CUCCCAUUU | AGAUGGGGGAGUUUGGGAAG | Translation |
| ccp-miR482b-1-3p | Cc02_g04600 | 3.0 | 22.174 | 1 | 22 | 2391 | 2412 | CUUCCCAAACCUCCCAUUUCCU | ACGAAGUGGGGGGGUUGGGAAU | Translation |
| ccp-miR482b-1-3p | Cc02_g12440 | 3.0 | 21.01 | 1 | 22 | 695 | 716 | CUUCCCAAACCUCCCAUUUCCU | GGGAAAUGGAGGGUUUGGGAUG | Cleavage |
| ccp-miR482b-1-3p | Cc02_g31990 | 2.5 | 24.902 | 1 | 22 | 818 | 839 | CUUCCCAAACCUCCCAUUUCCU | AAGAAAGGGGAGGUGUGGGGAG | Cleavage |
| ccp-miR482b-1-3p | Cc03_g12990 | 2.5 | 20.339 | 1 | 20 | 844 | 863 | CUUCCCAAACCUCCCAUUUC | CAAAGGGGAGGUUUGGGAGG | Cleavage |
| ccp-miR482b-1-3p | Cc05_g13720 | 2.5 | 18.201 | 1 | 22 | 506 | 527 | CUUCCCAAACCUCCCAUUUCCU | AAGGAAUAGGAGGUUUGGGAAC | Cleavage |
| ccp-miR482b-1-3p | Cc06_g04960 | 3.0 | 11.838 | 1 | 22 | 625 | 646 | CUUCCCAAACCUCCCAUUUCCU | AAGAAAGAGGGGGUUUGGGAGG | Cleavage |
| ccp-miR482b-1-3p | Cc06_g05600 | 2.5 | 19.568 | 1 | 20 | 1471 | 1490 | CUUCCCAAACCUCCCAUUUC | GGAGUGGGGGGUAUGGGAAG | Cleavage |
| ccp-miR482b-1-3p | Cc06_g11740 | 3.0 | 17.944 | 1 | 22 | 1622 | 1643 | CUUCCCAAACCUCCCAUUUCCU | AUGAGGUGGGAGGUAUGGGAAC | Cleavage |
| ccp-miR482b-1-3p | Cc08_g15650 | 3.0 | 16.479 | 1 | 20 | 365 | 384 | CUUCCCAAACCUCCCAUUUC | GGAAUGGGCGAUUUGGGGAG | Translation |
| ccp-miR482b-1-3p | Cc11_g14850 | 3.0 | 15.68 | 1 | 20 | 110 | 129 | CUUCCCAAACCUCCCAUUUC | GGAAUGGGGGCGUUGGGAAG | Translation |
| ccp-miR482b-1-5p | Cc01_g09760 | 2.0 | 8.969 | 1 | 21 | 1712 | 1732 | GGAAUGGGAGGACUGGGAAAG | UUUUUCCAGUCCUAUCAUUCC | Cleavage |
| ccp-miR482b-1-5p | Cc02_g15110 | 2.5 | 10.31 | 1 | 21 | 110 | 130 | GGAAUGGGAGGACUGGGAAAG | CUUUCUCAGUGUUUCCAUUCC | Translation |
| ccp-miR482b-1-5p | Cc04_g09000 | 3.0 | 17.574 | 1 | 21 | 679 | 699 | GGAAUGGGAGGACUGGGAAAG | UUUUCUUGGUCCUUUUAUUCC | Cleavage |
| ccp-miR482b-1-5p | Cc10_g05400 | 3.0 | 6.592 | 1 | 21 | 98 | 118 | GGAAUGGGAGGACUGGGAAAG | CUUUUCCAGCCCUGCCAUUUC | Cleavage |
| ccp-miR482b-1-5p | Cc11_g17080 | 3.0 | 5.09 | 1 | 20 | 15 | 34 | GGAAUGGGAGGACUGGGAAA | UAUCCUAUUCCUCCCAUUCU | Cleavage |
| ccp-miR482b-2-5p | Cc01_g09760 | 2.0 | 8.969 | 1 | 21 | 1712 | 1732 | GGAAUGGGAGGACUGGGAAAG | UUUUUCCAGUCCUAUCAUUCC | Cleavage |
| ccp-miR482b-2-5p | Cc02_g15110 | 2.5 | 10.31 | 1 | 21 | 110 | 130 | GGAAUGGGAGGACUGGGAAAG | CUUUCUCAGUGUUUCCAUUCC | Translation |
| ccp-miR482b-2-5p | Cc04_g09000 | 3.0 | 17.574 | 1 | 21 | 679 | 699 | GGAAUGGGAGGACUGGGAAAG | UUUUCUUGGUCCUUUUAUUCC | Cleavage |
| ccp-miR482b-2-5p | Cc10_g05400 | 3.0 | 6.592 | 1 | 21 | 98 | 118 | GGAAUGGGAGGACUGGGAAAG | CUUUUCCAGCCCUGCCAUUUC | Cleavage |
| ccp-miR482b-2-5p | Cc11_g17080 | 3.0 | 5.09 | 1 | 20 | 15 | 34 | GGAAUGGGAGGACUGGGAAA | UAUCCUAUUCCUCCCAUUCU | Cleavage |
| ccp-miR5013-5p | Cc00_g19900 | 3.0 | 14.944 | 1 | 21 | 712 | 732 | UUUGUGACAUAAAGGUGCUUC | GGAGCAUUAUUAUGUCAUAAA | Cleavage |
| ccp-miR5013-5p | Cc05_g05560 | 3.0 | 15.343 | 1 | 21 | 1013 | 1033 | UUUGUGACAUAAAGGUGCUUC | GAAGGAUCUUUAUGUAACAAA | Cleavage |
| ccp-miR5013-5p | Cc11_g14440 | 3.0 | 21.864 | 1 | 20 | 600 | 619 | UUUGUGACAUAAAGGUGCUU | AAGUUUCUUUGUGUCACAAG | Cleavage |
| ccp-miR5014a-5p | Cc00_g32770 | 3.0 | 17.498 | 1 | 20 | 611 | 630 | UUGUACAAAUUUAAUUGUAU | AUGCAGUUGAUUUUGUGCAA | Translation |
| ccp-miR5014a-5p | Cc01_g19980 | 3.0 | 10.067 | 1 | 20 | 2367 | 2386 | UUGUACAAAUUUAAUUGUAU | AUAAAGUUUAAUUUGUAUAA | Cleavage |
| ccp-miR5014a-5p | Cc02_g03120 | 3.0 | 12.904 | 1 | 20 | 1400 | 1419 | UUGUACAAAUUUAAUUGUAU | AUAUAAAUAAACUUGUAUAA | Translation |
| ccp-miR5014a-5p | Cc02_g09340 | 3.0 | 14.867 | 1 | 21 | 1657 | 1677 | UUGUACAAAUUUAAUUGUAUA | UGUACAAGUAAAUGUGUGCAA | Cleavage |
| ccp-miR5014a-5p | Cc02_g30220 | 2.0 | 7.83 | 1 | 20 | 2376 | 2395 | UUGUACAAAUUUAAUUGUAU | AUAGAAUUAAUUUUGUACAA | Translation |
| ccp-miR5014a-5p | Cc05_g07760 | 3.0 | 15.869 | 1 | 21 | 792 | 812 | UUGUACAAAUUUAAUUGUAUA | UGUACAAUUAGAAAUGUACAA | Translation |
| ccp-miR5014a-5p | Cc07_g13360 | 3.0 | 12.013 | 1 | 20 | 1145 | 1164 | UUGUACAAAUUUAAUUGUAU | AUAAGAUUAAAUUUGUGUAG | Cleavage |
| ccp-miR5014a-5p | Cc10_g14360 | 3.0 | 13.133 | 1 | 20 | 186 | 205 | UUGUACAAAUUUAAUUGUAU | AUGGAAUGAAAUUUGUACAG | Cleavage |
| ccp-miR5020a-3p | Cc00_g07020 | 3.0 | 18.361 | 1 | 20 | 1881 | 1900 | UGGAAGAAUGUAAGACUUGC | GCAGUUCUUACCUUUUUCCA | Translation |
| ccp-miR5020a-3p | Cc01_g11710 | 2.5 | 5.692 | 1 | 20 | 130 | 149 | UGGAAGAAUGUAAGACUUGC | GCGAGUCUUACAUAUUUCCA | Cleavage |
| ccp-miR5020a-3p | Cc01_g16270 | 3.0 | 17.559 | 1 | 20 | 779 | 798 | UGGAAGAAUGUAAGACUUGC | GCAACUCAUACCUUCUUCCA | Translation |
| ccp-miR5020a-3p | Cc02_g15030 | 2.5 | 21.792 | 1 | 20 | 609 | 628 | UGGAAGAAUGUAAGACUUGC | GCAUAUCUUGCAUUCUUCCA | Cleavage |
| ccp-miR5020a-3p | Cc06_g08040 | 2.5 | 18.851 | 1 | 20 | 970 | 989 | UGGAAGAAUGUAAGACUUGC | UCAGGUCUUGCAUUUUUCCA | Cleavage |
| ccp-miR5020a-3p | Cc10_g01180 | 3.0 | 19.883 | 1 | 20 | 1624 | 1643 | UGGAAGAAUGUAAGACUUGC | GGAAGUCUUUCGUUCUUUCA | Translation |
| ccp-miR5020a-3p | Cc11_g09660 | 3.0 | 16.164 | 1 | 20 | 2654 | 2673 | UGGAAGAAUGUAAGACUUGC | GCAAGCUUCACAUUCUUUCA | Cleavage |
| ccp-miR5042-1-3p | Cc02_g35510 | 3.0 | 11.219 | 1 | 20 | 3272 | 3291 | UGGGGCUUGGUCGAAGAUAG | CUUUCUUUGAUCAAGCUCUA | Cleavage |
| ccp-miR5042-1-3p | Cc02_g35530 | 3.0 | 14.009 | 1 | 20 | 3095 | 3114 | UGGGGCUUGGUCGAAGAUAG | CUUUCUUUGAUCAAGCUCUA | Cleavage |
| ccp-miR5042-1-3p | Cc05_g01130 | 3.0 | 17.128 | 1 | 20 | 433 | 452 | UGGGGCUUGGUCGAAGAUAG | CUAUUUUUGACCAAGUCUCC | Cleavage |
| ccp-miR5042-1-3p | Cc06_g19800 | 3.0 | 20.337 | 1 | 20 | 1785 | 1804 | UGGGGCUUGGUCGAAGAUAG | CUAGCUUUGAUGAAGCCCCA | Translation |
| ccp-miR5042-1-3p | Cc08_g00020 | 0.5 | 13.934 | 1 | 21 | 1009 | 1029 | UGGGGCUUGGUCGAAGAUAGU | ACUAUCUUCGACCAAGCCCCG | Cleavage |
| ccp-miR5042-2-3p | Cc02_g35510 | 3.0 | 11.219 | 1 | 20 | 3272 | 3291 | UGGGGCUUGGUCGAAGAUAG | CUUUCUUUGAUCAAGCUCUA | Cleavage |
| ccp-miR5042-2-3p | Cc02_g35530 | 3.0 | 14.009 | 1 | 20 | 3095 | 3114 | UGGGGCUUGGUCGAAGAUAG | CUUUCUUUGAUCAAGCUCUA | Cleavage |
| ccp-miR5042-2-3p | Cc05_g01130 | 3.0 | 17.128 | 1 | 22 | 431 | 452 | UGGGGCUUGGUCGAAGAUAGUU | ACCUAUUUUUGACCAAGUCUCC | Cleavage |
| ccp-miR5042-2-3p | Cc06_g19800 | 3.0 | 20.337 | 1 | 20 | 1785 | 1804 | UGGGGCUUGGUCGAAGAUAG | CUAGCUUUGAUGAAGCCCCA | Translation |
| ccp-miR5042-2-3p | Cc08_g00020 | 0.5 | 13.934 | 1 | 22 | 1008 | 1029 | UGGGGCUUGGUCGAAGAUAGUU | AACUAUCUUCGACCAAGCCCCG | Cleavage |
| ccp-miR5167b-5p | Cc00_g06990 | 3.0 | 7.894 | 1 | 20 | 971 | 990 | UCUAGUUAAAGUAAUUCAAC | GUUGAAUUACUUAAACAAGG | Cleavage |
| ccp-miR5167b-5p | Cc08_g07550 | 3.0 | 14.402 | 1 | 20 | 995 | 1014 | UCUAGUUAAAGUAAUUCAAC | GAUGGGUUACUUUAGUUAGA | Cleavage |
| ccp-miR5167b-5p | Cc09_g03570 | 3.0 | 18.609 | 1 | 20 | 381 | 400 | UCUAGUUAAAGUAAUUCAAC | GUUGGAUUACUUUGGCAAGA | Cleavage |
| ccp-miR5167b-5p | Cc10_g00090 | 3.0 | 18.893 | 1 | 20 | 458 | 477 | UCUAGUUAAAGUAAUUCAAC | GUUGAAAUACUUCAAUUGGA | Cleavage |
| ccp-miR5210-5p | Cc00_g19890 | 3.0 | 15.438 | 1 | 22 | 594 | 615 | UAAAUGUGAUGGAAUUAGGGGU | AACUUUAAUUCUGCCACAUUUA | Translation |
| ccp-miR5210-5p | Cc00_g20170 | 3.0 | 13.844 | 1 | 21 | 1164 | 1184 | UAAAUGUGAUGGAAUUAGGGG | CCUCUAAUUCCAUGACAAUUA | Cleavage |
| ccp-miR5210-5p | Cc01_g06600 | 3.0 | 14.472 | 1 | 22 | 1339 | 1360 | UAAAUGUGAUGGAAUUAGGGGU | AUUCCUAAUCCCAUCACUUUUA | Cleavage |
| ccp-miR5210-5p | Cc01_g21830 | 3.0 | 15.445 | 1 | 21 | 849 | 869 | UAAAUGUGAUGGAAUUAGGGG | UCCUUGAUUUUUUCACAUUUA | Translation |
| ccp-miR5210-5p | Cc06_g18610 | 3.0 | 17.122 | 1 | 22 | 967 | 988 | UAAAUGUGAUGGAAUUAGGGGU | GCUCCUGAUUAUAUCACAUUUG | Cleavage |
| ccp-miR5210-5p | Cc08_g07210 | 3.0 | 16.058 | 1 | 20 | 1715 | 1734 | UAAAUGUGAUGGAAUUAGGG | ACCUGAUUCCAUCACAUAUA | Cleavage |
| ccp-miR5210-5p | Cc09_g04780 | 3.0 | 10.21 | 1 | 20 | 376 | 395 | UAAAUGUGAUGGAAUUAGGG | UCCUAUUUCCUUCAUAUUUA | Translation |
| ccp-miR5210-5p | Cc11_g01460 | 3.0 | 8.205 | 1 | 21 | 2690 | 2710 | UAAAUGUGAUGGAAUUAGGGG | CCCUUAAAUCCAUUACAUUUU | Cleavage |
| ccp-miR5272f-3p | Cc00_g05990 | 3.0 | 15.089 | 1 | 23 | 756 | 777 | GAAUUGAUUCUGUUUGGAGACAU | GUCUCUCCAAAC-GAAUCAGUUU | Translation |
| ccp-miR5272f-3p | Cc01_g09750 | 3.0 | 15.881 | 1 | 24 | 145 | 168 | GAAUUGAUUCUGUUUGGAGACAUU | AUUCUUUCUAAUCAGAAUCAAUUA | Cleavage |
| ccp-miR5272f-3p | Cc01_g18580 | 3.0 | 15.564 | 1 | 24 | 294 | 317 | GAAUUGAUUCUGUUUGGAGACAUU | AAGUUUUCCAAAGAAAAUCGAUUC | Translation |
| ccp-miR5272f-3p | Cc02_g23190 | 3.0 | 11.605 | 1 | 20 | 911 | 930 | GAAUUGAUUCUGUUUGGAGA | UCUUCAAAAACAAUUAAUUC | Translation |
| ccp-miR5272f-3p | Cc03_g02190 | 3.0 | 15.026 | 1 | 20 | 512 | 531 | GAAUUGAUUCUGUUUGGAGA | UCUCUAAAUAAGGUCAAUUC | Translation |
| ccp-miR5272f-3p | Cc03_g12040 | 1.5 | 18.042 | 1 | 24 | 136 | 159 | GAAUUGAUUCUGUUUGGAGACAUU | AAUGUCCCCAGACAGAAUCAAUUC | Cleavage |
| ccp-miR5272f-3p | Cc05_g07360 | 1.0 | 11.02 | 1 | 24 | 193 | 216 | GAAUUGAUUCUGUUUGGAGACAUU | AAUGUCCCCAAACAGAAUCAAUUC | Cleavage |
| ccp-miR5272f-3p | Cc05_g07390 | 0.0 | 12.604 | 1 | 24 | 193 | 216 | GAAUUGAUUCUGUUUGGAGACAUU | AAUGUCUCCAAACAGAAUCAAUUC | Cleavage |
| ccp-miR5272f-3p | Cc06_g00030 | 3.0 | 14.125 | 1 | 24 | 1841 | 1864 | GAAUUGAUUCUGUUUGGAGACAUU | AAACUUUGCAAACAUAAUUAAUUC | Translation |
| ccp-miR5272f-3p | Cc07_g10820 | 3.0 | 10.077 | 1 | 23 | 254 | 276 | GAAUUGAUUCUGUUUGGAGACAU | AUCUCACCAGAAAGAAUCAAUUU | Cleavage |
| ccp-miR5272f-3p | Cc11_g15490 | 3.0 | 16.909 | 1 | 24 | 662 | 685 | GAAUUGAUUCUGUUUGGAGACAUU | AGAGCCUCGGAGCAGAAUCAAUUC | Cleavage |
| ccp-miR5272f-3p | Cc11_g15940 | 3.0 | 15.157 | 1 | 21 | 975 | 995 | GAAUUGAUUCUGUUUGGAGAC | GUCUCCGAACCGAUUCAAUUU | Translation |
| ccp-miR530-3p | Cc00_g03930 | 3.0 | 19.126 | 1 | 20 | 3140 | 3159 | AGGUGCAGAUGCUGAUGCAG | CUACAUCAGGAUUUGCAUCU | Translation |
| ccp-miR530-3p | Cc00_g04830 | 3.0 | 21.209 | 1 | 20 | 1395 | 1414 | AGGUGCAGAUGCUGAUGCAG | UUUCAGCAGCAUUUGCACCU | Cleavage |
| ccp-miR530-3p | Cc00_g19640 | 3.0 | 16.076 | 1 | 20 | 2929 | 2948 | AGGUGCAGAUGCUGAUGCAG | CUGGAUUAGCAUCUGCAUCA | Cleavage |
| ccp-miR530-3p | Cc01_g11980 | 3.0 | 18.054 | 1 | 20 | 1647 | 1665 | AGGUGCAGAUGCUGAUGCAG | CUCCAUCA-CAUCUGCACCU | Cleavage |
| ccp-miR530-3p | Cc02_g30020 | 3.0 | 15.967 | 1 | 20 | 3250 | 3269 | AGGUGCAGAUGCUGAUGCAG | CUGCACCUGCACCUGCACCU | Translation |
| ccp-miR530-3p | Cc04_g11520 | 3.0 | 15.217 | 1 | 20 | 1534 | 1553 | AGGUGCAGAUGCUGAUGCAG | CUACAUCUGUAUCUGCAUCU | Cleavage |
| ccp-miR530-3p | Cc06_g07040 | 2.5 | 11.38 | 1 | 20 | 124 | 143 | AGGUGCAGAUGCUGAUGCAG | CUGCGUCUGCGUCUGCAUCU | Cleavage |
| ccp-miR530-3p | Cc06_g16040 | 3.0 | 17.609 | 1 | 20 | 1891 | 1910 | AGGUGCAGAUGCUGAUGCAG | CUGCAUCAUCAUCAGCAUCU | Cleavage |
| ccp-miR530-3p | Cc07_g03520 | 2.5 | 21.496 | 1 | 20 | 2254 | 2273 | AGGUGCAGAUGCUGAUGCAG | CUGUAUCUGCAUUUGUACCU | Cleavage |
| ccp-miR530-3p | Cc07_g08860 | 3.0 | 15.847 | 1 | 20 | 666 | 685 | AGGUGCAGAUGCUGAUGCAG | CUGCACCUGCACCUGCACCU | Translation |
| ccp-miR530-3p | Cc08_g02300 | 2.5 | 8.877 | 1 | 20 | 115 | 134 | AGGUGCAGAUGCUGAUGCAG | CUGCACCAUCAUCUGCAUCU | Cleavage |
| ccp-miR530-3p | Cc08_g12650 | 3.0 | 23.536 | 1 | 20 | 83 | 102 | AGGUGCAGAUGCUGAUGCAG | UUGCAGCAGCAUCUGCAGCU | Cleavage |
| ccp-miR530-3p | Cc08_g14220 | 1.5 | 18.803 | 1 | 20 | 963 | 982 | AGGUGCAGAUGCUGAUGCAG | CUGCAUCAUCAUCUGCACUU | Cleavage |
| ccp-miR530-3p | Cc10_g03680 | 2.5 | 23.858 | 1 | 20 | 2463 | 2482 | AGGUGCAGAUGCUGAUGCAG | CUGCAUUGGCAUCUACACCU | Cleavage |
| ccp-miR530-3p | Cc11_g13710 | 3.0 | 18.261 | 1 | 20 | 1027 | 1046 | AGGUGCAGAUGCUGAUGCAG | CUGCAUCUGCAUCUGCAUGU | Cleavage |
| ccp-miR530-3p | Cc11_g17040 | 1.5 | 19.366 | 1 | 20 | 116 | 135 | AGGUGCAGAUGCUGAUGCAG | CUGCAUCUGCAUCUGCAUCU | Cleavage |
| ccp-miR530-5p | Cc01_g03560 | 2.5 | 11.959 | 1 | 20 | 706 | 725 | UCUGCAUUUGCACCUGCACC | GGUGUAUAUGCAAAUGCAGA | Cleavage |
| ccp-miR530-5p | Cc02_g22820 | 3.0 | 22.123 | 1 | 20 | 1627 | 1646 | UCUGCAUUUGCACCUGCACC | GGUGCAGGGGACAAUGCAGA | Translation |
| ccp-miR530-5p | Cc02_g39750 | 3.0 | 23.797 | 1 | 20 | 1559 | 1578 | UCUGCAUUUGCACCUGCACC | GGUGCAGGUGCAGGUGGAGG | Cleavage |
| ccp-miR530-5p | Cc03_g04860 | 1.5 | 18.263 | 1 | 20 | 427 | 446 | UCUGCAUUUGCACCUGCACC | GGUGCAGGUGUAAAUGCAGC | Cleavage |
| ccp-miR530-5p | Cc03_g05790 | 2.5 | 24.347 | 1 | 20 | 360 | 379 | UCUGCAUUUGCACCUGCACC | GGUGCAGGUGUCGGUGCAGA | Translation |
| ccp-miR530-5p | Cc04_g06060 | 2.5 | 16.852 | 1 | 21 | 374 | 394 | UCUGCAUUUGCACCUGCACCU | AGGUGCAGGUGCAGGUGCAAA | Cleavage |
| ccp-miR530-5p | Cc06_g22890 | 3.0 | 14.695 | 1 | 21 | 1405 | 1425 | UCUGCAUUUGCACCUGCACCU | AGGGGCAGCUGCAUAUGCAGA | Cleavage |
| ccp-miR530-5p | Cc07_g06640 | 3.0 | 18.575 | 1 | 21 | 2310 | 2330 | UCUGCAUUUGCACCUGCACCU | GGCUGCAAUUGCAAAUGCAGA | Cleavage |
| ccp-miR530-5p | Cc07_g16310 | 2.5 | 18.949 | 1 | 21 | 1400 | 1420 | UCUGCAUUUGCACCUGCACCU | AAGUGCAGGAGCAAAUGCAGG | Cleavage |
| ccp-miR530-5p | Cc07_g20900 | 3.0 | 23.184 | 1 | 21 | 783 | 803 | UCUGCAUUUGCACCUGCACCU | AGUUGCUGCUGCAAAUGCAGA | Cleavage |
| ccp-miR5640-5p | Cc07_g05450 | 3.0 | 15.877 | 1 | 21 | 3318 | 3338 | UGAGAGAAGGAAGUAGACUCU | AGAGGUUGUUUUCUUCUCUCA | Cleavage |
| ccp-miR5640-5p | Cc10_g13300 | 3.0 | 16.741 | 1 | 21 | 2490 | 2510 | UGAGAGAAGGAAGUAGACUCU | AGAUUCUGUUUCCUUUUUUCA | Cleavage |
| ccp-miR5648-5p | Cc00_g03550 | 2.5 | 15.083 | 1 | 24 | 638 | 661 | UUCUGAAAUGUUUGGCUUUGCUUU | GAGUAAAAGCGAAACAUUUCAGAG | Cleavage |
| ccp-miR5648-5p | Cc00_g03560 | 3.0 | 15.49 | 1 | 24 | 725 | 748 | UUCUGAAAUGUUUGGCUUUGCUUU | GAGUAAAAGCGAAACAUUUUAGAG | Cleavage |
| ccp-miR5648-5p | Cc00_g11460 | 3.0 | 19.534 | 1 | 24 | 50 | 73 | UUCUGAAAUGUUUGGCUUUGCUUU | GAGUAAAAGCGAAACAUUUUAGAG | Cleavage |
| ccp-miR5648-5p | Cc00_g15830 | 3.0 | 15.242 | 1 | 24 | 3004 | 3027 | UUCUGAAAUGUUUGGCUUUGCUUU | AAUCCAGAGCUAGACAGUUUAGAA | Cleavage |
| ccp-miR5648-5p | Cc01_g19480 | 3.0 | 23.951 | 1 | 20 | 1124 | 1143 | UUCUGAAAUGUUUGGCUUUG | CGAGGCCAAAUAUUUUGGGA | Cleavage |
| ccp-miR5648-5p | Cc02_g28490 | 3.0 | 18.064 | 1 | 21 | 294 | 314 | UUCUGAAAUGUUUGGCUUUGC | GCAAAGGCAACCAGUUCAGAA | Translation |
| ccp-miR5648-5p | Cc02_g36420 | 3.0 | 21.835 | 1 | 20 | 3826 | 3845 | UUCUGAAAUGUUUGGCUUUG | CAAAGUCGAGCACUUCAGGA | Cleavage |
| ccp-miR5648-5p | Cc03_g02770 | 3.0 | 18.621 | 1 | 20 | 3298 | 3317 | UUCUGAAAUGUUUGGCUUUG | CAAGGUUGAAUGUUUCAGAA | Cleavage |
| ccp-miR5648-5p | Cc03_g11570 | 2.5 | 13.944 | 1 | 24 | 524 | 547 | UUCUGAAAUGUUUGGCUUUGCUUU | GAGUAAAAGCGAAACAUUUCAGAG | Cleavage |
| ccp-miR5648-5p | Cc03_g11880 | 3.0 | 21.326 | 1 | 24 | 443 | 466 | UUCUGAAAUGUUUGGCUUUGCUUU | GAGUAAAAGCGAAACAUUUUAGAG | Cleavage |
| ccp-miR5648-5p | Cc03_g11890 | 2.5 | 19.931 | 1 | 24 | 629 | 652 | UUCUGAAAUGUUUGGCUUUGCUUU | GAGUAAAAGCGAAACAUUUCAGAG | Cleavage |
| ccp-miR5648-5p | Cc03_g11920 | 3.0 | 19.706 | 1 | 24 | 557 | 580 | UUCUGAAAUGUUUGGCUUUGCUUU | GAGUAAAAGCGAAACAUUUUAGAG | Cleavage |
| ccp-miR5648-5p | Cc03_g12140 | 3.0 | 17.547 | 1 | 24 | 629 | 652 | UUCUGAAAUGUUUGGCUUUGCUUU | GAGUAAAAGCAAAACAUUUUAGAG | Cleavage |
| ccp-miR5648-5p | Cc04_g07590 | 3.0 | 16.22 | 1 | 20 | 324 | 343 | UUCUGAAAUGUUUGGCUUUG | GAAAGCCAAGCACUUCAGGA | Cleavage |
| ccp-miR5648-5p | Cc04_g16650 | 3.0 | 20.982 | 1 | 24 | 2186 | 2209 | UUCUGAAAUGUUUGGCUUUGCUUU | GAAACAAAGCCAAACAUUCUAGAU | Cleavage |
| ccp-miR5648-5p | Cc05_g12610 | 2.5 | 11.131 | 1 | 20 | 2181 | 2200 | UUCUGAAAUGUUUGGCUUUG | CACAGCCCAACAUUUUAGAA | Cleavage |
| ccp-miR5648-5p | Cc05_g16450 | 1.5 | 17.24 | 1 | 20 | 635 | 654 | UUCUGAAAUGUUUGGCUUUG | CAGAGCAAAACAUUUCAGAA | Cleavage |
| ccp-miR5648-5p | Cc06_g01250 | 3.0 | 15.722 | 1 | 24 | 1585 | 1608 | UUCUGAAAUGUUUGGCUUUGCUUU | GAACCAUGGCCAAGUAUUUCAGAG | Cleavage |
| ccp-miR5648-5p | Cc07_g01030 | 2.5 | 15.745 | 1 | 21 | 518 | 538 | UUCUGAAAUGUUUGGCUUUGC | GCAAAGCUAAACAUUUCACAG | Cleavage |
| ccp-miR5648-5p | Cc08_g17090 | 3.0 | 19.713 | 1 | 20 | 3347 | 3366 | UUCUGAAAUGUUUGGCUUUG | CAGAGUCAAGCAUUUCAGUA | Cleavage |
| ccp-miR5648-5p | Cc09_g05210 | 3.0 | 20.163 | 1 | 24 | 2275 | 2298 | UUCUGAAAUGUUUGGCUUUGCUUU | AAUAUGGAGCCGGAUAUUUCAGAA | Cleavage |
| ccp-miR5653-1-3p | Cc00_g04380 | 2.5 | 3.682 | 1 | 21 | 441 | 461 | UGGGUUGAGUUGGAUUGACUU | AUGUCAAUCCAACCCAAUCCA | Cleavage |
| ccp-miR5653-1-3p | Cc02_g08390 | 2.0 | 12.461 | 1 | 23 | 75 | 97 | UGGGUUGAGUUGGAUUGACUUGG | CUGAGUCAGCCCAACUCAACUCA | Cleavage |
| ccp-miR5653-1-3p | Cc02_g11880 | 2.5 | 21.571 | 1 | 23 | 1991 | 2013 | UGGGUUGAGUUGGAUUGACUUGG | CUGAGUCAGUCCAAUUCAACCUU | Cleavage |
| ccp-miR5653-1-3p | Cc04_g06900 | 3.0 | 5.363 | 1 | 22 | 588 | 609 | UGGGUUGAGUUGGAUUGACUUG | CAAUUCGAUACAACUCAGCCCA | Cleavage |
| ccp-miR5653-1-3p | Cc04_g07960 | 3.0 | 13.439 | 1 | 20 | 833 | 852 | UGGGUUGAGUUGGAUUGACU | AGUCAUGCCAACUCGGCCCA | Cleavage |
| ccp-miR5653-1-3p | Cc04_g11140 | 3.0 | 4.795 | 1 | 23 | 4 | 26 | UGGGUUGAGUUGGAUUGACUUGG | UCAACUCAACUCAACUCAACUCA | Cleavage |
| ccp-miR5653-1-3p | Cc07_g09000 | 3.0 | 9.73 | 1 | 20 | 347 | 366 | UGGGUUGAGUUGGAUUGACU | GGCCGAUUCAACUCAGCCCA | Cleavage |
| ccp-miR5653-1-3p | Cc07_g11780 | 3.0 | 16.336 | 1 | 21 | 328 | 348 | UGGGUUGAGUUGGAUUGACUU | AAGUCAAACCAAUUCAACCUC | Cleavage |
| ccp-miR5653-1-3p | Cc10_g00770 | 2.5 | 17.219 | 1 | 24 | 757 | 780 | UGGGUUGAGUUGGAUUGACUUGGC | GUCGAGUCGAGUCAACUCAACUCA | Cleavage |
| ccp-miR5653-2-3p | Cc01_g01360 | 2.5 | 16.064 | 1 | 21 | 1387 | 1407 | UGGGUUGAGUUGAGUUGAGUU | AACUCGACUCGACUCGACUCG | Cleavage |
| ccp-miR5653-2-3p | Cc01_g15300 | 2.5 | 20.801 | 1 | 20 | 583 | 602 | UGGGUUGAGUUGAGUUGAGU | ACUCGACUCACCACAACCCA | Translation |
| ccp-miR5653-2-3p | Cc02_g34600 | 3.0 | 6.743 | 1 | 24 | 120 | 143 | UGGGUUGAGUUGAGUUGAGUUGAG | CUCAGCUCAACUUGACUCGGUCCA | Cleavage |
| ccp-miR5653-2-3p | Cc03_g00210 | 2.5 | 12.048 | 1 | 21 | 2373 | 2393 | UGGGUUGAGUUGAGUUGAGUU | GAAUCAACUUAUCUCAACCCA | Translation |
| ccp-miR5653-2-3p | Cc03_g02260 | 3.0 | 15.925 | 1 | 24 | 252 | 275 | UGGGUUGAGUUGAGUUGAGUUGAG | CUCUUCUCAACUCAAUUCAACUCC | Cleavage |
| ccp-miR5653-2-3p | Cc04_g01780 | 3.0 | 12.122 | 1 | 24 | 140 | 163 | UGGGUUGAGUUGAGUUGAGUUGAG | CUCGACUCAGCUCAUCACAACUCA | Translation |
| ccp-miR5653-2-3p | Cc04_g06900 | 3.0 | 5.363 | 1 | 22 | 588 | 609 | UGGGUUGAGUUGAGUUGAGUUG | CAAUUCGAUACAACUCAGCCCA | Cleavage |
| ccp-miR5653-2-3p | Cc04_g11140 | 0.5 | 4.795 | 1 | 24 | 3 | 26 | UGGGUUGAGUUGAGUUGAGUUGAG | CUCAACUCAACUCAACUCAACUCA | Cleavage |
| ccp-miR5653-2-3p | Cc06_g18630 | 3.0 | 13.598 | 1 | 21 | 244 | 264 | UGGGUUGAGUUGAGUUGAGUU | GAUUCAAGUCAUCUCAACUCA | Translation |
| ccp-miR5653-2-3p | Cc06_g21080 | 3.0 | 7.515 | 1 | 24 | 1070 | 1093 | UGGGUUGAGUUGAGUUGAGUUGAG | UUUAACCCAACCCAACUCAACCCU | Cleavage |
| ccp-miR5653-2-3p | Cc07_g21030 | 2.5 | 17.135 | 1 | 21 | 935 | 955 | UGGGUUGAGUUGAGUUGAGUU | AACUCGACUCGACUCGACUCG | Cleavage |
| ccp-miR5653-2-3p | Cc08_g10530 | 3.0 | 9.569 | 1 | 21 | 94 | 114 | UGGGUUGAGUUGAGUUGAGUU | AACUCGACUCAACUCACCCCC | Cleavage |
| ccp-miR5653-2-3p | Cc08_g15000 | 2.0 | 18.332 | 1 | 22 | 1838 | 1859 | UGGGUUGAGUUGAGUUGAGUUG | UGACUCAAAUCAACUUGACCCA | Cleavage |
| ccp-miR5653-2-3p | Cc10_g00770 | 3.0 | 17.226 | 1 | 23 | 763 | 785 | UGGGUUGAGUUGAGUUGAGUUGA | UCGAGUCAACUCAACUCAACUGA | Cleavage |
| ccp-miR5653-2-3p | Cc11_g16160 | 2.5 | 15.5 | 1 | 24 | 51 | 74 | UGGGUUGAGUUGAGUUGAGUUGAG | CUCAGCUCAGCUCAGCUCAGCUCA | Cleavage |
| ccp-miR5658-3p | Cc00_g02810 | 0.5 | 9.082 | 1 | 20 | 128 | 147 | AUGAUGAUGAUGAUGAUGAG | UUCAUCAUCAUCAUCAUCAU | Cleavage |
| ccp-miR5658-3p | Cc00_g07430 | 1.0 | 4.576 | 1 | 20 | 173 | 192 | AUGAUGAUGAUGAUGAUGAG | AUCAUCAUCAUCAUCAUCAU | Cleavage |
| ccp-miR5658-3p | Cc00_g09070 | 0.0 | 5.283 | 1 | 21 | 907 | 927 | AUGAUGAUGAUGAUGAUGAGC | GCUCAUCAUCAUCAUCAUCAU | Cleavage |
| ccp-miR5658-3p | Cc00_g09500 | 2.0 | 10.64 | 1 | 20 | 458 | 477 | AUGAUGAUGAUGAUGAUGAG | CAUAUCAUUAUCAUCAUCAU | Cleavage |
| ccp-miR5658-3p | Cc00_g10960 | 2.5 | 11.118 | 1 | 20 | 2093 | 2112 | AUGAUGAUGAUGAUGAUGAG | AUCAUCAUCAUCAUCAUCCU | Cleavage |
| ccp-miR5658-3p | Cc00_g13860 | 1.5 | 17.519 | 1 | 20 | 324 | 343 | AUGAUGAUGAUGAUGAUGAG | UUCAUCGUCGUCAUCAUCAU | Cleavage |
| ccp-miR5658-3p | Cc00_g13990 | 2.5 | 15.189 | 1 | 21 | 1883 | 1903 | AUGAUGAUGAUGAUGAUGAGC | GUUCAUCAUCAUCAUUGUCAA | Cleavage |
| ccp-miR5658-3p | Cc00_g17830 | 1.0 | 11.281 | 1 | 20 | 198 | 217 | AUGAUGAUGAUGAUGAUGAG | UUCAUCAUCAUCAUCGUCAU | Cleavage |
| ccp-miR5658-3p | Cc00_g18340 | 1.0 | 13.826 | 1 | 20 | 565 | 584 | AUGAUGAUGAUGAUGAUGAG | AUCAUCAUCAUCAUCAUCAU | Cleavage |
| ccp-miR5658-3p | Cc00_g21760 | 1.5 | 4.669 | 1 | 20 | 141 | 160 | AUGAUGAUGAUGAUGAUGAG | UUCUUCAUCAUCAUCAUCAU | Cleavage |
| ccp-miR5658-3p | Cc00_g28240 | 1.0 | 11.608 | 1 | 21 | 335 | 355 | AUGAUGAUGAUGAUGAUGAGC | GAUCAUCAUCAUCAUCAUCAU | Cleavage |
| ccp-miR5658-3p | Cc00_g30460 | 2.0 | 16.734 | 1 | 20 | 368 | 387 | AUGAUGAUGAUGAUGAUGAG | UGCAUCAUCAUCAUCAUCAU | Cleavage |
| ccp-miR5658-3p | Cc01_g01920 | 3.0 | 13.573 | 1 | 20 | 18 | 37 | AUGAUGAUGAUGAUGAUGAG | AACAUCAUCAUCAUCAUCAA | Cleavage |
| ccp-miR5658-3p | Cc01_g05880 | 1.5 | 11.414 | 1 | 20 | 156 | 175 | AUGAUGAUGAUGAUGAUGAG | CUCAUCAUCAUCAUCACCAU | Cleavage |
| ccp-miR5658-3p | Cc01_g06240 | 1.5 | 14.14 | 1 | 20 | 238 | 257 | AUGAUGAUGAUGAUGAUGAG | UUCAUCGUCAUUAUCAUCAU | Cleavage |
| ccp-miR5658-3p | Cc01_g08770 | 3.0 | 16.473 | 1 | 21 | 71 | 91 | AUGAUGAUGAUGAUGAUGAGC | GUUCAUCAUCAUCUUCAUCUU | Cleavage |
| ccp-miR5658-3p | Cc01_g09090 | 1.5 | 10.422 | 1 | 20 | 413 | 432 | AUGAUGAUGAUGAUGAUGAG | CUCAUCAUCAUCAUCAUCUU | Cleavage |
| ccp-miR5658-3p | Cc01_g10300 | 0.5 | 10.908 | 1 | 20 | 1082 | 1101 | AUGAUGAUGAUGAUGAUGAG | UUCAUCAUCAUCAUCAUCAU | Cleavage |
| ccp-miR5658-3p | Cc01_g10490 | 2.5 | 13.313 | 1 | 21 | 820 | 840 | AUGAUGAUGAUGAUGAUGAGC | GGUUAUGAUCAUCAUCAUCAU | Cleavage |
| ccp-miR5658-3p | Cc01_g11530 | 2.5 | 13.914 | 1 | 20 | 165 | 184 | AUGAUGAUGAUGAUGAUGAG | CUCAUCAUCAUCAUCAUGAC | Cleavage |
| ccp-miR5658-3p | Cc01_g11670 | 2.0 | 13.341 | 1 | 21 | 513 | 533 | AUGAUGAUGAUGAUGAUGAGC | GAUCAUCAUCCUCAUCAUCAU | Translation |
| ccp-miR5658-3p | Cc01_g11930 | 1.0 | 9.369 | 1 | 20 | 638 | 657 | AUGAUGAUGAUGAUGAUGAG | GUCAUCAUCAUCAUCAUCAU | Cleavage |
| ccp-miR5658-3p | Cc01_g12280 | 0.0 | 14.455 | 1 | 20 | 438 | 457 | AUGAUGAUGAUGAUGAUGAG | CUCAUCAUCAUCAUCAUCAU | Cleavage |
| ccp-miR5658-3p | Cc01_g12990 | 2.0 | 16.424 | 1 | 20 | 703 | 722 | AUGAUGAUGAUGAUGAUGAG | CUCGUCCUCAUCAUCAUUAU | Cleavage |
| ccp-miR5658-3p | Cc01_g13250 | 2.5 | 21.226 | 1 | 20 | 962 | 981 | AUGAUGAUGAUGAUGAUGAG | CUCAUCAUCAUCAUCACCAC | Cleavage |
| ccp-miR5658-3p | Cc01_g13520 | 2.0 | 10.567 | 1 | 20 | 166 | 185 | AUGAUGAUGAUGAUGAUGAG | UUCAUCAUCAAUAUCAUCAU | Translation |
| ccp-miR5658-3p | Cc01_g13870 | 2.0 | 21.407 | 1 | 20 | 540 | 559 | AUGAUGAUGAUGAUGAUGAG | ACCAUCAUCAUCAUCAUCAU | Cleavage |
| ccp-miR5658-3p | Cc01_g15320 | 1.5 | 19.75 | 1 | 20 | 443 | 462 | AUGAUGAUGAUGAUGAUGAG | CUCCUCAUCAUUAUCAUCAU | Cleavage |
| ccp-miR5658-3p | Cc01_g16720 | 2.0 | 11.895 | 1 | 20 | 1009 | 1028 | AUGAUGAUGAUGAUGAUGAG | UUCCUCAUCGUCAUCAUCAU | Cleavage |
| ccp-miR5658-3p | Cc01_g17710 | 2.0 | 13.025 | 1 | 20 | 282 | 301 | AUGAUGAUGAUGAUGAUGAG | UUCAUCAUCAUCGUCGUCGU | Cleavage |
| ccp-miR5658-3p | Cc01_g18390 | 1.5 | 4.268 | 1 | 20 | 194 | 213 | AUGAUGAUGAUGAUGAUGAG | AUCAUCAUCAUCAUCAUCGU | Cleavage |
| ccp-miR5658-3p | Cc01_g20730 | 1.5 | 15.002 | 1 | 20 | 372 | 391 | AUGAUGAUGAUGAUGAUGAG | AUCAUCAUCAUCAUUAUCAU | Cleavage |
| ccp-miR5658-3p | Cc01_g21160 | 0.5 | 3.229 | 1 | 20 | 63 | 82 | AUGAUGAUGAUGAUGAUGAG | UUCAUCAUCAUCAUCAUCAU | Cleavage |
| ccp-miR5658-3p | Cc01_g21350 | 2.0 | 7.121 | 1 | 20 | 253 | 272 | AUGAUGAUGAUGAUGAUGAG | GUCAUCAUCAUUAUUAUCAU | Cleavage |
| ccp-miR5658-3p | Cc01_g21850 | 2.5 | 11.259 | 1 | 20 | 1737 | 1756 | AUGAUGAUGAUGAUGAUGAG | CUUAUCAUUAUUAUUAUUAU | Cleavage |
| ccp-miR5658-3p | Cc02_g00420 | 2.0 | 17.13 | 1 | 20 | 509 | 528 | AUGAUGAUGAUGAUGAUGAG | CUUGUCAUCAUCAUUAUCGU | Cleavage |
| ccp-miR5658-3p | Cc02_g02270 | 2.0 | 9.567 | 1 | 20 | 27 | 46 | AUGAUGAUGAUGAUGAUGAG | AUCAUCAUCAUCUUCAUCAU | Cleavage |
| ccp-miR5658-3p | Cc02_g02470 | 1.5 | 9.071 | 1 | 20 | 191 | 210 | AUGAUGAUGAUGAUGAUGAG | CUCAUCAUCAUCAUCAUCGG | Cleavage |
| ccp-miR5658-3p | Cc02_g07630 | 3.0 | 12.296 | 1 | 20 | 2061 | 2080 | AUGAUGAUGAUGAUGAUGAG | UGUAUCAUCAUCAUCAUCGU | Cleavage |
| ccp-miR5658-3p | Cc02_g08460 | 2.0 | 10.888 | 1 | 20 | 1869 | 1888 | AUGAUGAUGAUGAUGAUGAG | GUCAUCGUCAUCAUCAUCGU | Cleavage |
| ccp-miR5658-3p | Cc02_g13120 | 1.5 | 19.56 | 1 | 20 | 1136 | 1155 | AUGAUGAUGAUGAUGAUGAG | CUCGUCAUCGUCGUCAUCAU | Cleavage |
| ccp-miR5658-3p | Cc02_g13630 | 2.0 | 12.176 | 1 | 20 | 262 | 281 | AUGAUGAUGAUGAUGAUGAG | UUCAUCAUCAUCAUCGUCAG | Cleavage |
| ccp-miR5658-3p | Cc02_g14920 | 2.5 | 11.458 | 1 | 21 | 3349 | 3369 | AUGAUGAUGAUGAUGAUGAGC | GCUUAACAUCAUGAUCAUCAU | Translation |
| ccp-miR5658-3p | Cc02_g15030 | 2.0 | 17.344 | 1 | 20 | 898 | 917 | AUGAUGAUGAUGAUGAUGAG | UUCAUCAUCAUCAUCCUCAU | Cleavage |
| ccp-miR5658-3p | Cc02_g16900 | 2.5 | 19.689 | 1 | 20 | 87 | 106 | AUGAUGAUGAUGAUGAUGAG | UUCAUCGUCAUUGUCAUCGU | Cleavage |
| ccp-miR5658-3p | Cc02_g17450 | 1.0 | 12.466 | 1 | 21 | 311 | 331 | AUGAUGAUGAUGAUGAUGAGC | GAUCAUCAUCAUCAUCAUCAU | Cleavage |
| ccp-miR5658-3p | Cc02_g18920 | 0.0 | 1.886 | 1 | 20 | 299 | 318 | AUGAUGAUGAUGAUGAUGAG | CUCAUCAUCAUCAUCAUCAU | Cleavage |
| ccp-miR5658-3p | Cc02_g19570 | 1.5 | 6.406 | 1 | 20 | 1 | 20 | AUGAUGAUGAUGAUGAUGAG | AUCAUCAUCAUCGUCAUCAU | Cleavage |
| ccp-miR5658-3p | Cc02_g20240 | 1.0 | 13.563 | 1 | 20 | 240 | 259 | AUGAUGAUGAUGAUGAUGAG | AUCAUCAUCAUCAUCAUCAU | Cleavage |
| ccp-miR5658-3p | Cc02_g20830 | 1.0 | 14.54 | 1 | 20 | 120 | 139 | AUGAUGAUGAUGAUGAUGAG | CUCAUCAUCAUCAUCAUCAG | Cleavage |
| ccp-miR5658-3p | Cc02_g22390 | 1.5 | 12.573 | 1 | 20 | 343 | 362 | AUGAUGAUGAUGAUGAUGAG | CUUAUCAUCAGCAUCAUCAU | Translation |
| ccp-miR5658-3p | Cc02_g22960 | 2.0 | 7.964 | 1 | 20 | 518 | 537 | AUGAUGAUGAUGAUGAUGAG | ACCAUCAUCAUCAUCAUCAU | Cleavage |
| ccp-miR5658-3p | Cc02_g23060 | 3.0 | 15.358 | 1 | 21 | 504 | 524 | AUGAUGAUGAUGAUGAUGAGC | GUUCAUCUUCAUCAUCAUCGA | Cleavage |
| ccp-miR5658-3p | Cc02_g24840 | 1.0 | 11.791 | 1 | 20 | 384 | 403 | AUGAUGAUGAUGAUGAUGAG | AUCAUCAUCAUCAUCAUCAU | Cleavage |
| ccp-miR5658-3p | Cc02_g25580 | 2.0 | 7.407 | 1 | 20 | 163 | 182 | AUGAUGAUGAUGAUGAUGAG | AUCAUCAUCGUCAUCAUCGU | Cleavage |
| ccp-miR5658-3p | Cc02_g26940 | 0.5 | 8.087 | 1 | 20 | 100 | 119 | AUGAUGAUGAUGAUGAUGAG | UUCAUCAUCAUCAUCAUCAU | Cleavage |
| ccp-miR5658-3p | Cc02_g27730 | 1.5 | 15.123 | 1 | 20 | 90 | 109 | AUGAUGAUGAUGAUGAUGAG | UUCAUCAUCAUCAUCAUCAG | Cleavage |
| ccp-miR5658-3p | Cc02_g28490 | 2.0 | 11.53 | 1 | 20 | 242 | 261 | AUGAUGAUGAUGAUGAUGAG | AUCAUCAUCAUCAUCAUCAG | Cleavage |
| ccp-miR5658-3p | Cc02_g31340 | 2.0 | 19.617 | 1 | 20 | 401 | 420 | AUGAUGAUGAUGAUGAUGAG | CUGAUCAUCAUCAUCAUCAG | Cleavage |
| ccp-miR5658-3p | Cc02_g33320 | 3.0 | 14.113 | 1 | 20 | 2896 | 2915 | AUGAUGAUGAUGAUGAUGAG | CAGAUCAUCAUCAUCAUCAU | Cleavage |
| ccp-miR5658-3p | Cc02_g34990 | 0.5 | 15.746 | 1 | 21 | 992 | 1012 | AUGAUGAUGAUGAUGAUGAGC | GUUCAUCAUCAUCAUCAUCAU | Cleavage |
| ccp-miR5658-3p | Cc02_g39490 | 1.5 | 22.348 | 1 | 20 | 726 | 745 | AUGAUGAUGAUGAUGAUGAG | UUCAUCAUCGUUAUCAUCAU | Cleavage |
| ccp-miR5658-3p | Cc03_g01680 | 2.5 | 8.339 | 1 | 20 | 257 | 276 | AUGAUGAUGAUGAUGAUGAG | UGCAUCAUCAUCAUCAUCGU | Cleavage |
| ccp-miR5658-3p | Cc03_g01730 | 2.0 | 9.698 | 1 | 20 | 483 | 502 | AUGAUGAUGAUGAUGAUGAG | GACAUCAUCAUCAUCAUCAU | Cleavage |
| ccp-miR5658-3p | Cc03_g01790 | 2.0 | 20.944 | 1 | 21 | 112 | 132 | AUGAUGAUGAUGAUGAUGAGC | GCUUGUCCUCAUCAUCAUCAU | Cleavage |
| ccp-miR5658-3p | Cc03_g03100 | 2.5 | 6.292 | 1 | 20 | 332 | 351 | AUGAUGAUGAUGAUGAUGAG | GUCAUCGUCAUCGUCAUCGU | Cleavage |
| ccp-miR5658-3p | Cc03_g03940 | 2.5 | 17.696 | 1 | 20 | 273 | 292 | AUGAUGAUGAUGAUGAUGAG | UUUAUCAUCAUCAUCAUCUU | Cleavage |
| ccp-miR5658-3p | Cc03_g05780 | 3.0 | 2.073 | 1 | 20 | 260 | 279 | AUGAUGAUGAUGAUGAUGAG | UACAUCAUCAUCAUCAUCAC | Cleavage |
| ccp-miR5658-3p | Cc03_g06490 | 3.0 | 7.813 | 1 | 20 | 363 | 382 | AUGAUGAUGAUGAUGAUGAG | AUCAUCAUCAUCAUCUUUAU | Cleavage |
| ccp-miR5658-3p | Cc03_g07710 | 3.0 | 13.501 | 1 | 21 | 987 | 1007 | AUGAUGAUGAUGAUGAUGAGC | GGUCGUCGUCAUCGUCGUCAU | Cleavage |
| ccp-miR5658-3p | Cc03_g09680 | 2.5 | 9.586 | 1 | 20 | 221 | 240 | AUGAUGAUGAUGAUGAUGAG | AUCAUCAUCAUUAUCAUCAG | Cleavage |
| ccp-miR5658-3p | Cc03_g10660 | 1.0 | 21.875 | 1 | 20 | 62 | 81 | AUGAUGAUGAUGAUGAUGAG | CUCAUCAUCAUCAUCAUCAA | Cleavage |
| ccp-miR5658-3p | Cc03_g11370 | 2.5 | 9.267 | 1 | 21 | 116 | 136 | AUGAUGAUGAUGAUGAUGAGC | GCCUAUCAUCAUCUUCAUCAU | Cleavage |
| ccp-miR5658-3p | Cc03_g16050 | 0.5 | 17.099 | 1 | 20 | 271 | 290 | AUGAUGAUGAUGAUGAUGAG | CUCAUCGUCAUCAUCAUCAU | Cleavage |
| ccp-miR5658-3p | Cc04_g00130 | 1.0 | 20.83 | 1 | 20 | 105 | 124 | AUGAUGAUGAUGAUGAUGAG | CUCAUCAUCGUCGUCAUCAU | Cleavage |
| ccp-miR5658-3p | Cc04_g00190 | 2.0 | 6.376 | 1 | 20 | 1165 | 1184 | AUGAUGAUGAUGAUGAUGAG | UCCAUCAUCAUCAUCAUCAU | Cleavage |
| ccp-miR5658-3p | Cc04_g00300 | 1.0 | 20.38 | 1 | 21 | 419 | 439 | AUGAUGAUGAUGAUGAUGAGC | GAUCAUCAUCAUCAUCAUCAU | Cleavage |
| ccp-miR5658-3p | Cc04_g00520 | 3.0 | 12.071 | 1 | 20 | 59 | 78 | AUGAUGAUGAUGAUGAUGAG | CUCAUCAUCAUCGCCAUCAC | Cleavage |
| ccp-miR5658-3p | Cc04_g01100 | 1.5 | 15.391 | 1 | 20 | 24 | 43 | AUGAUGAUGAUGAUGAUGAG | GUCGUCAUCAUCAUCAUCAU | Cleavage |
| ccp-miR5658-3p | Cc04_g01950 | 1.0 | 17.802 | 1 | 20 | 128 | 147 | AUGAUGAUGAUGAUGAUGAG | AUCAUCAUCAUCAUCAUCAU | Cleavage |
| ccp-miR5658-3p | Cc04_g02460 | 2.0 | 16.567 | 1 | 20 | 354 | 373 | AUGAUGAUGAUGAUGAUGAG | AUCAUCUUCAUCAUCAUCAU | Cleavage |
| ccp-miR5658-3p | Cc04_g02680 | 2.0 | 12.841 | 1 | 20 | 1201 | 1220 | AUGAUGAUGAUGAUGAUGAG | GCCAUCAUCAUCAUCAUCAU | Cleavage |
| ccp-miR5658-3p | Cc04_g02760 | 2.5 | 18.326 | 1 | 20 | 48 | 67 | AUGAUGAUGAUGAUGAUGAG | UUCGUCGUCGUCAUCAUCGU | Cleavage |
| ccp-miR5658-3p | Cc04_g03480 | 0.5 | 8.491 | 1 | 20 | 99 | 118 | AUGAUGAUGAUGAUGAUGAG | UUCAUCAUCAUCAUCAUCAU | Cleavage |
| ccp-miR5658-3p | Cc04_g04250 | 3.0 | 8.222 | 1 | 20 | 523 | 542 | AUGAUGAUGAUGAUGAUGAG | UCUAUCAUCGUCAUCAUCAU | Cleavage |
| ccp-miR5658-3p | Cc04_g04360 | 0.0 | 8.694 | 1 | 20 | 264 | 283 | AUGAUGAUGAUGAUGAUGAG | CUCAUCAUCAUCAUCAUCAU | Cleavage |
| ccp-miR5658-3p | Cc04_g05970 | 2.5 | 9.18 | 1 | 20 | 93 | 112 | AUGAUGAUGAUGAUGAUGAG | UUCAUCAUCAUUAGCAUCAU | Cleavage |
| ccp-miR5658-3p | Cc04_g08000 | 2.0 | 4.162 | 1 | 20 | 50 | 69 | AUGAUGAUGAUGAUGAUGAG | AUCAUCAUCAUCAUCAUCAC | Cleavage |
| ccp-miR5658-3p | Cc04_g08430 | 2.0 | 12.829 | 1 | 21 | 142 | 162 | AUGAUGAUGAUGAUGAUGAGC | GAUCAUCAUCAUCAUCAUCAA | Cleavage |
| ccp-miR5658-3p | Cc04_g08760 | 1.5 | 15.141 | 1 | 21 | 402 | 422 | AUGAUGAUGAUGAUGAUGAGC | GCUCAUCAUCAUCAUCAUCUU | Cleavage |
| ccp-miR5658-3p | Cc04_g14320 | 1.5 | 14.268 | 1 | 21 | 20 | 40 | AUGAUGAUGAUGAUGAUGAGC | GUUCAUCAACAUCAUCAUCAU | Cleavage |
| ccp-miR5658-3p | Cc04_g15610 | 2.0 | 16.205 | 1 | 20 | 312 | 331 | AUGAUGAUGAUGAUGAUGAG | AGCAUCAUCAUCAUCAUCAU | Cleavage |
| ccp-miR5658-3p | Cc05_g05740 | 3.0 | 8.096 | 1 | 20 | 695 | 714 | AUGAUGAUGAUGAUGAUGAG | GCCAUCAUCAUCGUUAUCAU | Cleavage |
| ccp-miR5658-3p | Cc05_g06380 | 2.0 | 5.669 | 1 | 20 | 38 | 57 | AUGAUGAUGAUGAUGAUGAG | AUCAUCAUCACCAUCAUCAU | Translation |
| ccp-miR5658-3p | Cc05_g11130 | 2.0 | 11.064 | 1 | 21 | 98 | 118 | AUGAUGAUGAUGAUGAUGAGC | GAUCAUCAUCAUCAUCAUCAA | Cleavage |
| ccp-miR5658-3p | Cc05_g11360 | 3.0 | 4.887 | 1 | 20 | 115 | 134 | AUGAUGAUGAUGAUGAUGAG | AUCAUCAUUGUCAUCGUCGU | Cleavage |
| ccp-miR5658-3p | Cc05_g12120 | 3.0 | 9.068 | 1 | 20 | 137 | 156 | AUGAUGAUGAUGAUGAUGAG | AUCGUCGUCGUCAUCGUCAU | Cleavage |
| ccp-miR5658-3p | Cc05_g13040 | 1.5 | 15.587 | 1 | 21 | 565 | 585 | AUGAUGAUGAUGAUGAUGAGC | GAUCAUCAUCGUCAUCAUCAU | Cleavage |
| ccp-miR5658-3p | Cc05_g14830 | 2.0 | 22.214 | 1 | 21 | 472 | 492 | AUGAUGAUGAUGAUGAUGAGC | GCUGAUCAUCUUCAUCAUCAU | Translation |
| ccp-miR5658-3p | Cc05_g16250 | 2.0 | 16.724 | 1 | 21 | 101 | 121 | AUGAUGAUGAUGAUGAUGAGC | GUUCAUCAUCAUCGUCGUCGU | Cleavage |
| ccp-miR5658-3p | Cc06_g00180 | 2.5 | 16.997 | 1 | 21 | 74 | 94 | AUGAUGAUGAUGAUGAUGAGC | GAUCAUCAUCAUCAUCAUCCU | Cleavage |
| ccp-miR5658-3p | Cc06_g00500 | 2.5 | 12.147 | 1 | 20 | 123 | 142 | AUGAUGAUGAUGAUGAUGAG | GACAUCAUCGUCAUCAUCAU | Cleavage |
| ccp-miR5658-3p | Cc06_g00500 | 3.0 | 13.579 | 1 | 20 | 147 | 166 | AUGAUGAUGAUGAUGAUGAG | CUCAUCAUUAUCAUCAUCCA | Cleavage |
| ccp-miR5658-3p | Cc06_g00700 | 2.0 | 8.99 | 1 | 20 | 502 | 521 | AUGAUGAUGAUGAUGAUGAG | UCCAUCAUCAUCAUCAUCAU | Cleavage |
| ccp-miR5658-3p | Cc06_g00860 | 3.0 | 23.891 | 1 | 20 | 612 | 631 | AUGAUGAUGAUGAUGAUGAG | GGCAUCAUCAUCAUCAUCAA | Cleavage |
| ccp-miR5658-3p | Cc06_g00970 | 3.0 | 24.974 | 1 | 20 | 612 | 631 | AUGAUGAUGAUGAUGAUGAG | GGCAUCAUCAUCAUCAUCAA | Cleavage |
| ccp-miR5658-3p | Cc06_g01240 | 2.0 | 23.574 | 1 | 20 | 8 | 27 | AUGAUGAUGAUGAUGAUGAG | GUCAUCAGCAUCAUCAUCAU | Cleavage |
| ccp-miR5658-3p | Cc06_g01920 | 2.0 | 12.85 | 1 | 20 | 115 | 134 | AUGAUGAUGAUGAUGAUGAG | GUCAUCAUCAUCUUCAUCAU | Cleavage |
| ccp-miR5658-3p | Cc06_g03140 | 1.0 | 7.483 | 1 | 20 | 266 | 285 | AUGAUGAUGAUGAUGAUGAG | CUCAUCAUCAUCAUCAUCAA | Cleavage |
| ccp-miR5658-3p | Cc06_g03440 | 1.5 | 16.045 | 1 | 20 | 296 | 315 | AUGAUGAUGAUGAUGAUGAG | AUCAUCAUCAUCAUCAUCGU | Cleavage |
| ccp-miR5658-3p | Cc06_g04760 | 0.5 | 15.138 | 1 | 20 | 585 | 604 | AUGAUGAUGAUGAUGAUGAG | CUCGUCAUCAUCAUCAUCAU | Cleavage |
| ccp-miR5658-3p | Cc06_g06260 | 0.5 | 22.354 | 1 | 20 | 1875 | 1894 | AUGAUGAUGAUGAUGAUGAG | UUCAUCAUCAUCAUCAUCAU | Cleavage |
| ccp-miR5658-3p | Cc06_g06280 | 1.5 | 3.492 | 1 | 20 | 90 | 109 | AUGAUGAUGAUGAUGAUGAG | AUCAUCAUCAUCAUUAUCAU | Cleavage |
| ccp-miR5658-3p | Cc06_g06680 | 1.5 | 19.831 | 1 | 21 | 1187 | 1207 | AUGAUGAUGAUGAUGAUGAGC | GCUCAUCAUCAUCCUCAUCGU | Cleavage |
| ccp-miR5658-3p | Cc06_g07470 | 2.0 | 16.634 | 1 | 20 | 302 | 321 | AUGAUGAUGAUGAUGAUGAG | CUCAGCAUCAUCAUCGUCGU | Cleavage |
| ccp-miR5658-3p | Cc06_g08340 | 1.0 | 6.805 | 1 | 21 | 373 | 393 | AUGAUGAUGAUGAUGAUGAGC | GAUCAUCAUCAUCAUCAUCAU | Cleavage |
| ccp-miR5658-3p | Cc06_g10150 | 2.0 | 4.588 | 1 | 20 | 131 | 150 | AUGAUGAUGAUGAUGAUGAG | CCCAUCAUCAUCAUCAUCAC | Cleavage |
| ccp-miR5658-3p | Cc06_g11520 | 1.0 | 17.782 | 1 | 20 | 1371 | 1390 | AUGAUGAUGAUGAUGAUGAG | AUCAUCAUCAUCAUCAUCAU | Cleavage |
| ccp-miR5658-3p | Cc06_g11580 | 2.0 | 13.797 | 1 | 20 | 2059 | 2078 | AUGAUGAUGAUGAUGAUGAG | CUUAUCGUCAUCUUCAUCAU | Cleavage |
| ccp-miR5658-3p | Cc06_g12460 | 2.0 | 18.303 | 1 | 20 | 260 | 279 | AUGAUGAUGAUGAUGAUGAG | CUCGUCGUCUUCAUCAUCAU | Translation |
| ccp-miR5658-3p | Cc06_g15240 | 1.0 | 16.46 | 1 | 20 | 578 | 597 | AUGAUGAUGAUGAUGAUGAG | CUCAUCAUCAUCGUCGUCAU | Cleavage |
| ccp-miR5658-3p | Cc06_g15920 | 2.5 | 11.63 | 1 | 20 | 411 | 430 | AUGAUGAUGAUGAUGAUGAG | UUCGUCAUCAUCACCAUCAU | Cleavage |
| ccp-miR5658-3p | Cc06_g16230 | 1.5 | 9.234 | 1 | 21 | 630 | 650 | AUGAUGAUGAUGAUGAUGAGC | GCUCAUCAUCAUCAGCAUCAU | Cleavage |
| ccp-miR5658-3p | Cc06_g16370 | 0.5 | 9.272 | 1 | 20 | 244 | 263 | AUGAUGAUGAUGAUGAUGAG | UUCAUCAUCAUCAUCAUCAU | Cleavage |
| ccp-miR5658-3p | Cc06_g17240 | 0.5 | 14.489 | 1 | 21 | 1918 | 1938 | AUGAUGAUGAUGAUGAUGAGC | GUUCAUCAUCAUCAUCAUCAU | Cleavage |
| ccp-miR5658-3p | Cc06_g17660 | 2.0 | 14.696 | 1 | 20 | 744 | 763 | AUGAUGAUGAUGAUGAUGAG | AUCAUCAUCAUCAUCAUCAA | Cleavage |
| ccp-miR5658-3p | Cc06_g19980 | 0.5 | 12.114 | 1 | 21 | 3199 | 3219 | AUGAUGAUGAUGAUGAUGAGC | GCUUAUCAUCAUCAUCAUCAU | Cleavage |
| ccp-miR5658-3p | Cc06_g20160 | 1.5 | 2.896 | 1 | 20 | 189 | 208 | AUGAUGAUGAUGAUGAUGAG | CUCAUCAUCAUCAUCAUUAA | Cleavage |
| ccp-miR5658-3p | Cc06_g21630 | 2.0 | 12.627 | 1 | 20 | 590 | 609 | AUGAUGAUGAUGAUGAUGAG | CUUAUCAUCAUCACCAUCAU | Cleavage |
| ccp-miR5658-3p | Cc06_g21970 | 1.0 | 6.115 | 1 | 21 | 1310 | 1330 | AUGAUGAUGAUGAUGAUGAGC | GAUCAUCAUCAUCAUCAUCAU | Cleavage |
| ccp-miR5658-3p | Cc06_g23200 | 2.5 | 10.045 | 1 | 20 | 1017 | 1036 | AUGAUGAUGAUGAUGAUGAG | CUUAUUAUUAUUAUCAUUAU | Cleavage |
| ccp-miR5658-3p | Cc07_g00110 | 2.0 | 7.015 | 1 | 20 | 297 | 316 | AUGAUGAUGAUGAUGAUGAG | AUCAUCAUCCUCAUCAUCAU | Translation |
| ccp-miR5658-3p | Cc07_g00550 | 3.0 | 10.275 | 1 | 20 | 30 | 49 | AUGAUGAUGAUGAUGAUGAG | GCCAUCAUCAUCAUCAUCAA | Cleavage |
| ccp-miR5658-3p | Cc07_g00950 | 1.0 | 5.523 | 1 | 20 | 476 | 495 | AUGAUGAUGAUGAUGAUGAG | GUCAUCAUCAUCAUCAUCAU | Cleavage |
| ccp-miR5658-3p | Cc07_g01020 | 2.0 | 12.511 | 1 | 20 | 245 | 264 | AUGAUGAUGAUGAUGAUGAG | CGUAUCAUUAUCAUCAUCAU | Cleavage |
| ccp-miR5658-3p | Cc07_g03240 | 2.0 | 10.771 | 1 | 20 | 733 | 752 | AUGAUGAUGAUGAUGAUGAG | AUCAUCAUCGUCGUCAUCAU | Cleavage |
| ccp-miR5658-3p | Cc07_g04300 | 1.0 | 13.648 | 1 | 20 | 816 | 835 | AUGAUGAUGAUGAUGAUGAG | AUCAUCAUCAUCAUCAUCAU | Cleavage |
| ccp-miR5658-3p | Cc07_g06260 | 2.0 | 10.048 | 1 | 20 | 1307 | 1326 | AUGAUGAUGAUGAUGAUGAG | UUCAUCAUCAUCGUCGUCGU | Cleavage |
| ccp-miR5658-3p | Cc07_g06940 | 3.0 | 6.558 | 1 | 21 | 454 | 474 | AUGAUGAUGAUGAUGAUGAGC | GCUCGUCACCAUCAUCCUCAU | Cleavage |
| ccp-miR5658-3p | Cc07_g09610 | 2.0 | 13.445 | 1 | 20 | 665 | 684 | AUGAUGAUGAUGAUGAUGAG | CUCAGCAUCAUCAUCAUCAG | Cleavage |
| ccp-miR5658-3p | Cc07_g11170 | 2.0 | 10.247 | 1 | 21 | 53 | 73 | AUGAUGAUGAUGAUGAUGAGC | GCACAUCAUCACCAUCAUCAU | Translation |
| ccp-miR5658-3p | Cc07_g11460 | 2.0 | 9.985 | 1 | 20 | 130 | 149 | AUGAUGAUGAUGAUGAUGAG | CUCGCCAUCAUCAUCAUCGU | Cleavage |
| ccp-miR5658-3p | Cc07_g13370 | 1.0 | 10.085 | 1 | 20 | 862 | 881 | AUGAUGAUGAUGAUGAUGAG | UUCAUCGUCAUCAUCAUCAU | Cleavage |
| ccp-miR5658-3p | Cc07_g15700 | 2.0 | 9.768 | 1 | 20 | 627 | 646 | AUGAUGAUGAUGAUGAUGAG | UUCAACAUCAUCAUCAUCGU | Cleavage |
| ccp-miR5658-3p | Cc07_g16400 | 3.0 | 18.767 | 1 | 21 | 184 | 204 | AUGAUGAUGAUGAUGAUGAGC | GUUGAUCAUCAUCAACAUCAU | Cleavage |
| ccp-miR5658-3p | Cc07_g19380 | 1.0 | 11.432 | 1 | 20 | 1922 | 1941 | AUGAUGAUGAUGAUGAUGAG | UUCAUCAUCAUCAUCAUCGU | Cleavage |
| ccp-miR5658-3p | Cc07_g21430 | 2.0 | 7.517 | 1 | 20 | 74 | 93 | AUGAUGAUGAUGAUGAUGAG | AUCAUCAUCGUCAUCGUCAU | Cleavage |
| ccp-miR5658-3p | Cc08_g01180 | 1.0 | 7.541 | 1 | 20 | 1853 | 1872 | AUGAUGAUGAUGAUGAUGAG | AUCAUCAUCAUCAUCAUCAU | Cleavage |
| ccp-miR5658-3p | Cc08_g03280 | 3.0 | 15.545 | 1 | 21 | 1332 | 1352 | AUGAUGAUGAUGAUGAUGAGC | GUUCAUUAUCAUCGUGAUCAU | Cleavage |
| ccp-miR5658-3p | Cc08_g03340 | 2.5 | 9.534 | 1 | 20 | 393 | 412 | AUGAUGAUGAUGAUGAUGAG | AUCAUCAUCAUCAUCAUCUU | Cleavage |
| ccp-miR5658-3p | Cc08_g03360 | 1.0 | 9.515 | 1 | 21 | 416 | 436 | AUGAUGAUGAUGAUGAUGAGC | GAUCAUCAUCAUCAUCAUCAU | Cleavage |
| ccp-miR5658-3p | Cc08_g05440 | 1.0 | 6.674 | 1 | 20 | 106 | 125 | AUGAUGAUGAUGAUGAUGAG | AUCAUCAUCAUCAUCAUCAU | Cleavage |
| ccp-miR5658-3p | Cc08_g08340 | 1.5 | 13.112 | 1 | 20 | 74 | 93 | AUGAUGAUGAUGAUGAUGAG | UUCAUCAUCAUCAUCAUCAA | Cleavage |
| ccp-miR5658-3p | Cc08_g09510 | 2.5 | 17.422 | 1 | 20 | 259 | 278 | AUGAUGAUGAUGAUGAUGAG | UUCAUCAUCAUCAUCGUUAG | Cleavage |
| ccp-miR5658-3p | Cc08_g11310 | 0.5 | 5.519 | 1 | 20 | 636 | 655 | AUGAUGAUGAUGAUGAUGAG | CUCAUCAUCGUCAUCAUCAU | Cleavage |
| ccp-miR5658-3p | Cc08_g16120 | 2.5 | 13.816 | 1 | 21 | 134 | 154 | AUGAUGAUGAUGAUGAUGAGC | GUUGAUCAUCAUAAUCAUCAU | Translation |
| ccp-miR5658-3p | Cc08_g16330 | 1.0 | 15.84 | 1 | 20 | 387 | 406 | AUGAUGAUGAUGAUGAUGAG | UUCAUCGUCAUCAUCAUCAU | Cleavage |
| ccp-miR5658-3p | Cc08_g16560 | 2.5 | 12.197 | 1 | 21 | 479 | 499 | AUGAUGAUGAUGAUGAUGAGC | GAUCAUCAUCAUCAUCAUCUU | Cleavage |
| ccp-miR5658-3p | Cc08_g17040 | 2.0 | 23.038 | 1 | 20 | 1268 | 1287 | AUGAUGAUGAUGAUGAUGAG | CUCAACAUCAUCAUCAUCAG | Cleavage |
| ccp-miR5658-3p | Cc09_g01540 | 3.0 | 8.527 | 1 | 20 | 1982 | 2001 | AUGAUGAUGAUGAUGAUGAG | CUCAUCAUCGUUAGCAUUAU | Cleavage |
| ccp-miR5658-3p | Cc09_g01630 | 2.5 | 13.491 | 1 | 20 | 937 | 956 | AUGAUGAUGAUGAUGAUGAG | AAUAUCAUCAUCAUCAUCAU | Cleavage |
| ccp-miR5658-3p | Cc09_g01730 | 0.0 | 24.302 | 1 | 20 | 287 | 306 | AUGAUGAUGAUGAUGAUGAG | CUCAUCAUCAUCAUCAUCAU | Cleavage |
| ccp-miR5658-3p | Cc09_g03080 | 2.5 | 19.796 | 1 | 20 | 181 | 200 | AUGAUGAUGAUGAUGAUGAG | UCCAUCAUCAUCAUCAUUAU | Cleavage |
| ccp-miR5658-3p | Cc09_g04610 | 1.5 | 16.463 | 1 | 21 | 1057 | 1077 | AUGAUGAUGAUGAUGAUGAGC | GGUCAUCAUCAUCAUCGUCAU | Cleavage |
| ccp-miR5658-3p | Cc09_g04620 | 2.0 | 10.926 | 1 | 20 | 270 | 289 | AUGAUGAUGAUGAUGAUGAG | UUCGUCAUCAUCAUCGUUAU | Cleavage |
| ccp-miR5658-3p | Cc09_g08810 | 3.0 | 19.871 | 1 | 20 | 453 | 472 | AUGAUGAUGAUGAUGAUGAG | UUCGUCGUCGUCAUCGUCGU | Cleavage |
| ccp-miR5658-3p | Cc09_g10240 | 2.5 | 9.734 | 1 | 20 | 394 | 413 | AUGAUGAUGAUGAUGAUGAG | GUCGUCGUCGUCAUCAUCAU | Cleavage |
| ccp-miR5658-3p | Cc09_g10350 | 2.5 | 23.173 | 1 | 20 | 442 | 461 | AUGAUGAUGAUGAUGAUGAG | CUCUUCGUCAUCAUCAUCAG | Cleavage |
| ccp-miR5658-3p | Cc09_g10410 | 1.0 | 10.463 | 1 | 21 | 112 | 132 | AUGAUGAUGAUGAUGAUGAGC | GAUCAUCAUCAUCAUCAUCAU | Cleavage |
| ccp-miR5658-3p | Cc10_g00540 | 3.0 | 19.94 | 1 | 21 | 451 | 471 | AUGAUGAUGAUGAUGAUGAGC | GAUCAUCAUCGUCAUGAUCAU | Cleavage |
| ccp-miR5658-3p | Cc10_g00770 | 1.0 | 9.13 | 1 | 20 | 137 | 156 | AUGAUGAUGAUGAUGAUGAG | GUCAUCAUCAUCAUCAUCAU | Cleavage |
| ccp-miR5658-3p | Cc10_g00820 | 1.0 | 15.642 | 1 | 21 | 362 | 382 | AUGAUGAUGAUGAUGAUGAGC | GCUCAUCGUCAUCGUCAUCAU | Cleavage |
| ccp-miR5658-3p | Cc10_g01070 | 2.0 | 6.016 | 1 | 20 | 376 | 395 | AUGAUGAUGAUGAUGAUGAG | CUCGUCAUCACCAUCGUCAU | Translation |
| ccp-miR5658-3p | Cc10_g01330 | 1.0 | 9.401 | 1 | 20 | 439 | 458 | AUGAUGAUGAUGAUGAUGAG | GUCAUCAUCAUCAUCAUCAU | Cleavage |
| ccp-miR5658-3p | Cc10_g02200 | 3.0 | 13.416 | 1 | 20 | 509 | 528 | AUGAUGAUGAUGAUGAUGAG | AACAUCAUCAUCAUCAUCAA | Cleavage |
| ccp-miR5658-3p | Cc10_g07250 | 2.5 | 13.686 | 1 | 21 | 811 | 831 | AUGAUGAUGAUGAUGAUGAGC | GCUUUUCAGCAUCAUCAUCAU | Cleavage |
| ccp-miR5658-3p | Cc10_g08190 | 1.0 | 3.938 | 1 | 20 | 170 | 189 | AUGAUGAUGAUGAUGAUGAG | UUCAUCAUCAUCAUCAUUAU | Cleavage |
| ccp-miR5658-3p | Cc10_g10260 | 3.0 | 5.821 | 1 | 21 | 97 | 117 | AUGAUGAUGAUGAUGAUGAGC | GUUCAUCAUCAUCGUCUUCGU | Cleavage |
| ccp-miR5658-3p | Cc10_g10640 | 2.5 | 21.411 | 1 | 20 | 1437 | 1456 | AUGAUGAUGAUGAUGAUGAG | CUCGUCGUCGUCGUCGUCAU | Cleavage |
| ccp-miR5658-3p | Cc10_g10960 | 0.5 | 15.52 | 1 | 20 | 193 | 212 | AUGAUGAUGAUGAUGAUGAG | UUCAUCAUCAUCAUCAUCAU | Cleavage |
| ccp-miR5658-3p | Cc10_g11830 | 2.5 | 6.661 | 1 | 20 | 1496 | 1515 | AUGAUGAUGAUGAUGAUGAG | UUUAUCAUCAUUAUUAUUAU | Cleavage |
| ccp-miR5658-3p | Cc10_g13590 | 3.0 | 16.999 | 1 | 20 | 137 | 156 | AUGAUGAUGAUGAUGAUGAG | AUCAUCGUCGUCGUCAUCGU | Cleavage |
| ccp-miR5658-3p | Cc10_g14440 | 2.5 | 13.667 | 1 | 20 | 376 | 395 | AUGAUGAUGAUGAUGAUGAG | AUCAUCAUCAUUAUCAUCAA | Cleavage |
| ccp-miR5658-3p | Cc10_g14770 | 0.0 | 5.497 | 1 | 20 | 1622 | 1641 | AUGAUGAUGAUGAUGAUGAG | CUCAUCAUCAUCAUCAUCAU | Cleavage |
| ccp-miR5658-3p | Cc10_g16340 | 1.5 | 8.303 | 1 | 21 | 77 | 97 | AUGAUGAUGAUGAUGAUGAGC | GUUCCUCAUCAUCAUCAUCAU | Cleavage |
| ccp-miR5658-3p | Cc11_g10890 | 1.5 | 16.596 | 1 | 20 | 389 | 408 | AUGAUGAUGAUGAUGAUGAG | UUCAUCAUCCUCAUCAUCAU | Translation |
| ccp-miR5658-3p | Cc11_g12850 | 3.0 | 11.172 | 1 | 21 | 98 | 118 | AUGAUGAUGAUGAUGAUGAGC | GUUCAUCAUCAUUAGCAUUAU | Cleavage |
| ccp-miR5658-3p | Cc11_g14290 | 1.0 | 6.16 | 1 | 20 | 1061 | 1080 | AUGAUGAUGAUGAUGAUGAG | AUCAUCAUCAUCAUCAUCAU | Cleavage |
| ccp-miR5658-3p | Cc11_g15010 | 1.0 | 10.935 | 1 | 20 | 1576 | 1595 | AUGAUGAUGAUGAUGAUGAG | CUCAUCAUCAUCAUCAUUGU | Cleavage |
| ccp-miR5658-3p | Cc11_g17410 | 1.0 | 1.054 | 1 | 20 | 77 | 96 | AUGAUGAUGAUGAUGAUGAG | CUCCUCAUCAUCAUCAUCAU | Cleavage |
| ccp-miR5741a-1-3p | Cc00_g01710 | 3.0 | 16.649 | 1 | 20 | 1335 | 1354 | UAGGGACUAAAUUGAUGAAA | UUUCCUCGGUUUAGUUCUUA | Cleavage |
| ccp-miR5741a-1-3p | Cc00_g03400 | 3.0 | 18.507 | 1 | 20 | 467 | 486 | UAGGGACUAAAUUGAUGAAA | UUUAAUCUAUUUAGUUCCUG | Cleavage |
| ccp-miR5741a-1-3p | Cc00_g23180 | 2.5 | 16.237 | 1 | 20 | 1013 | 1032 | UAGGGACUAAAUUGAUGAAA | UUUCAUUAAAUUAGUUUCUA | Translation |
| ccp-miR5741a-1-3p | Cc01_g06760 | 3.0 | 15.748 | 1 | 20 | 1776 | 1795 | UAGGGACUAAAUUGAUGAAA | UUUCAUUAGUUUAGUCAUUA | Cleavage |
| ccp-miR5741a-1-3p | Cc02_g06250 | 3.0 | 11.865 | 1 | 21 | 5539 | 5559 | UAGGGACUAAAUUGAUGAAAA | UUUUUAUCAUUUUAGUCCCGA | Cleavage |
| ccp-miR5741a-1-3p | Cc02_g19340 | 3.0 | 4.149 | 1 | 21 | 4737 | 4757 | UAGGGACUAAAUUGAUGAAAA | UUUUAGUCAAUUUAAUCCCUA | Cleavage |
| ccp-miR5741a-1-3p | Cc03_g08400 | 3.0 | 17.353 | 1 | 20 | 1348 | 1367 | UAGGGACUAAAUUGAUGAAA | UUUCCUCAAUUUAGUCCAUG | Cleavage |
| ccp-miR5741a-1-3p | Cc03_g13080 | 1.5 | 12.023 | 1 | 20 | 377 | 396 | UAGGGACUAAAUUGAUGAAA | UUUUUUCAAUUUAGUCCCUA | Cleavage |
| ccp-miR5741a-1-3p | Cc05_g11490 | 3.0 | 6.852 | 1 | 20 | 218 | 237 | UAGGGACUAAAUUGAUGAAA | UGUCAUCAAAUCAGUCCCUA | Translation |
| ccp-miR5741a-1-3p | Cc08_g02590 | 3.0 | 15.943 | 1 | 20 | 501 | 520 | UAGGGACUAAAUUGAUGAAA | UUUCAUCAUUUGGGUUCCUA | Translation |
| ccp-miR5741a-1-3p | Cc09_g08390 | 1.5 | 7.052 | 1 | 21 | 751 | 771 | UAGGGACUAAAUUGAUGAAAA | UUUUUAUCAAUUUAGUCUUUA | Cleavage |
| ccp-miR5741a-2-3p | Cc02_g19340 | 3.0 | 17.225 | 1 | 20 | 4679 | 4698 | UAGGGACUAAACUGAUAGUU | AAAUAUCAAUUUCGUCCCUA | Cleavage |
| ccp-miR5741a-2-3p | Cc05_g05860 | 3.0 | 19.758 | 1 | 21 | 97 | 117 | UAGGGACUAAACUGAUAGUUG | CAACUCUCAGUUUUGUUUCUA | Cleavage |
| ccp-miR5741a-2-3p | Cc09_g07380 | 3.0 | 18.525 | 1 | 21 | 559 | 578 | UAGGGACUAAACUGAUAGUUG | CAGCUA-CAGUUUAGUCCUUA | Cleavage |
| ccp-miR5741a-2-3p | Cc09_g07390 | 3.0 | 18.031 | 1 | 21 | 1213 | 1232 | UAGGGACUAAACUGAUAGUUG | CAGCUA-CAGUUUAGUCCUUA | Cleavage |
| ccp-miR5741a-2-3p | Cc09_g10840 | 3.0 | 22.077 | 1 | 21 | 673 | 693 | UAGGGACUAAACUGAUAGUUG | UGGCUGUCACUUUAGUUCCUA | Cleavage |
| ccp-miR5741a-3p | Cc02_g19340 | 3.0 | 17.225 | 1 | 20 | 4679 | 4698 | UAGGGACUAAACUGAUAGUU | AAAUAUCAAUUUCGUCCCUA | Cleavage |
| ccp-miR5741a-3p | Cc05_g05860 | 3.0 | 19.758 | 1 | 21 | 97 | 117 | UAGGGACUAAACUGAUAGUUG | CAACUCUCAGUUUUGUUUCUA | Cleavage |
| ccp-miR5741a-3p | Cc09_g07380 | 3.0 | 18.525 | 1 | 21 | 559 | 578 | UAGGGACUAAACUGAUAGUUG | CAGCUA-CAGUUUAGUCCUUA | Cleavage |
| ccp-miR5741a-3p | Cc09_g07390 | 3.0 | 18.031 | 1 | 21 | 1213 | 1232 | UAGGGACUAAACUGAUAGUUG | CAGCUA-CAGUUUAGUCCUUA | Cleavage |
| ccp-miR5741a-3p | Cc09_g10840 | 3.0 | 22.077 | 1 | 21 | 673 | 693 | UAGGGACUAAACUGAUAGUUG | UGGCUGUCACUUUAGUUCCUA | Cleavage |
| ccp-miR5780-3p | Cc03_g10580 | 3.0 | 15.703 | 1 | 20 | 1179 | 1198 | UGUUUUGAGUGUUUGAUAAA | UUUAUCAAACACAUAAAAUU | Cleavage |
| ccp-miR5780-3p | Cc05_g00800 | 0.5 | 16.614 | 1 | 22 | 971 | 992 | UGUUUUGAGUGUUUGAUAAAUG | CAUUUAUUAAACACUCAAAACA | Cleavage |
| ccp-miR5780-3p | Cc06_g09770 | 3.0 | 16.263 | 1 | 21 | 1962 | 1982 | UGUUUUGAGUGUUUGAUAAAU | AUUUGUCAAAGAGUCAAAAUA | Translation |
| ccp-miR5780-3p | Cc06_g16810 | 2.5 | 12.66 | 1 | 22 | 781 | 802 | UGUUUUGAGUGUUUGAUAAAUG | CAUGUAACAAACACUCAAAAUA | Cleavage |
| ccp-miR5780-3p | Cc06_g23580 | 0.0 | 13.649 | 1 | 22 | 1296 | 1317 | UGUUUUGAGUGUUUGAUAAAUG | CUUUUAUCAAACACUCAAAACA | Cleavage |
| ccp-miR5780-3p | Cc07_g05550 | 2.5 | 8.714 | 1 | 20 | 90 | 109 | UGUUUUGAGUGUUUGAUAAA | UUCUUCAAACACUCAAGACA | Cleavage |
| ccp-miR5780-3p | Cc08_g03290 | 3.0 | 8.538 | 1 | 20 | 551 | 570 | UGUUUUGAGUGUUUGAUAAA | UUUAUCAAACAAUCAAAGGA | Translation |
| ccp-miR5780-3p | Cc09_g05800 | 3.0 | 7.733 | 1 | 20 | 56 | 75 | UGUUUUGAGUGUUUGAUAAA | UUCAUCAAACGCUCAAAAUU | Cleavage |
| ccp-miR5780-3p | Cc10_g00050 | 3.0 | 4.682 | 1 | 22 | 447 | 468 | UGUUUUGAGUGUUUGAUAAAUG | CAUUUUACACACACUCAAAACA | Cleavage |
| ccp-miR5780-3p | Cc11_g01920 | 2.5 | 12.273 | 1 | 22 | 1110 | 1131 | UGUUUUGAGUGUUUGAUAAAUG | UAUUUAUCAAACACCCACAACA | Cleavage |
| ccp-miR5780-3p | Cc11_g06270 | 1.0 | 7.202 | 1 | 22 | 69 | 90 | UGUUUUGAGUGUUUGAUAAAUG | UAUUUAUCAAACACCCAAAACA | Cleavage |
| ccp-miR5780-3p | Cc11_g09280 | 3.0 | 16.726 | 1 | 20 | 1190 | 1209 | UGUUUUGAGUGUUUGAUAAA | UUUAUCAAGGACUCAAAUCA | Translation |
| ccp-miR5780d-3-3p | Cc03_g10580 | 3.0 | 15.703 | 1 | 20 | 1179 | 1198 | UGUUUUGAGUGUUUGAUAAA | UUUAUCAAACACAUAAAAUU | Cleavage |
| ccp-miR5780d-3-3p | Cc05_g00800 | 0.5 | 16.614 | 1 | 22 | 971 | 992 | UGUUUUGAGUGUUUGAUAAAUG | CAUUUAUUAAACACUCAAAACA | Cleavage |
| ccp-miR5780d-3-3p | Cc06_g09770 | 3.0 | 16.263 | 1 | 21 | 1962 | 1982 | UGUUUUGAGUGUUUGAUAAAU | AUUUGUCAAAGAGUCAAAAUA | Translation |
| ccp-miR5780d-3-3p | Cc06_g16810 | 2.5 | 12.66 | 1 | 22 | 781 | 802 | UGUUUUGAGUGUUUGAUAAAUG | CAUGUAACAAACACUCAAAAUA | Cleavage |
| ccp-miR5780d-3-3p | Cc06_g23580 | 0.0 | 13.649 | 1 | 22 | 1296 | 1317 | UGUUUUGAGUGUUUGAUAAAUG | CUUUUAUCAAACACUCAAAACA | Cleavage |
| ccp-miR5780d-3-3p | Cc07_g05550 | 2.5 | 8.714 | 1 | 20 | 90 | 109 | UGUUUUGAGUGUUUGAUAAA | UUCUUCAAACACUCAAGACA | Cleavage |
| ccp-miR5780d-3-3p | Cc08_g03290 | 3.0 | 8.538 | 1 | 20 | 551 | 570 | UGUUUUGAGUGUUUGAUAAA | UUUAUCAAACAAUCAAAGGA | Translation |
| ccp-miR5780d-3-3p | Cc09_g05800 | 3.0 | 7.733 | 1 | 20 | 56 | 75 | UGUUUUGAGUGUUUGAUAAA | UUCAUCAAACGCUCAAAAUU | Cleavage |
| ccp-miR5780d-3-3p | Cc10_g00050 | 3.0 | 4.682 | 1 | 22 | 447 | 468 | UGUUUUGAGUGUUUGAUAAAUG | CAUUUUACACACACUCAAAACA | Cleavage |
| ccp-miR5780d-3-3p | Cc11_g01920 | 2.5 | 12.273 | 1 | 22 | 1110 | 1131 | UGUUUUGAGUGUUUGAUAAAUG | UAUUUAUCAAACACCCACAACA | Cleavage |
| ccp-miR5780d-3-3p | Cc11_g06270 | 1.0 | 7.202 | 1 | 22 | 69 | 90 | UGUUUUGAGUGUUUGAUAAAUG | UAUUUAUCAAACACCCAAAACA | Cleavage |
| ccp-miR5780d-3-3p | Cc11_g09280 | 3.0 | 16.726 | 1 | 20 | 1190 | 1209 | UGUUUUGAGUGUUUGAUAAA | UUUAUCAAGGACUCAAAUCA | Translation |
| ccp-miR5780d-4-3p | Cc03_g10580 | 3.0 | 15.703 | 1 | 20 | 1179 | 1198 | UGUUUUGAGUGUUUGAUAAA | UUUAUCAAACACAUAAAAUU | Cleavage |
| ccp-miR5780d-4-3p | Cc05_g00800 | 0.5 | 16.614 | 1 | 22 | 971 | 992 | UGUUUUGAGUGUUUGAUAAAUG | CAUUUAUUAAACACUCAAAACA | Cleavage |
| ccp-miR5780d-4-3p | Cc06_g09770 | 3.0 | 16.263 | 1 | 21 | 1962 | 1982 | UGUUUUGAGUGUUUGAUAAAU | AUUUGUCAAAGAGUCAAAAUA | Translation |
| ccp-miR5780d-4-3p | Cc06_g16810 | 2.5 | 12.66 | 1 | 22 | 781 | 802 | UGUUUUGAGUGUUUGAUAAAUG | CAUGUAACAAACACUCAAAAUA | Cleavage |
| ccp-miR5780d-4-3p | Cc06_g23580 | 0.0 | 13.649 | 1 | 22 | 1296 | 1317 | UGUUUUGAGUGUUUGAUAAAUG | CUUUUAUCAAACACUCAAAACA | Cleavage |
| ccp-miR5780d-4-3p | Cc07_g05550 | 2.5 | 8.714 | 1 | 20 | 90 | 109 | UGUUUUGAGUGUUUGAUAAA | UUCUUCAAACACUCAAGACA | Cleavage |
| ccp-miR5780d-4-3p | Cc08_g03290 | 3.0 | 8.538 | 1 | 20 | 551 | 570 | UGUUUUGAGUGUUUGAUAAA | UUUAUCAAACAAUCAAAGGA | Translation |
| ccp-miR5780d-4-3p | Cc09_g05800 | 3.0 | 7.733 | 1 | 20 | 56 | 75 | UGUUUUGAGUGUUUGAUAAA | UUCAUCAAACGCUCAAAAUU | Cleavage |
| ccp-miR5780d-4-3p | Cc10_g00050 | 3.0 | 4.682 | 1 | 22 | 447 | 468 | UGUUUUGAGUGUUUGAUAAAUG | CAUUUUACACACACUCAAAACA | Cleavage |
| ccp-miR5780d-4-3p | Cc11_g01920 | 2.5 | 12.273 | 1 | 22 | 1110 | 1131 | UGUUUUGAGUGUUUGAUAAAUG | UAUUUAUCAAACACCCACAACA | Cleavage |
| ccp-miR5780d-4-3p | Cc11_g06270 | 1.0 | 7.202 | 1 | 22 | 69 | 90 | UGUUUUGAGUGUUUGAUAAAUG | UAUUUAUCAAACACCCAAAACA | Cleavage |
| ccp-miR5780d-4-3p | Cc11_g09280 | 3.0 | 16.726 | 1 | 20 | 1190 | 1209 | UGUUUUGAGUGUUUGAUAAA | UUUAUCAAGGACUCAAAUCA | Translation |
| ccp-miR6142-5p | Cc00_g21430 | 3.0 | 16.785 | 1 | 23 | 1063 | 1085 | GACGAUUUUUUAAGAUAUGACUA | UUGACAUAUCUGAAAAGAUCGUU | Cleavage |
| ccp-miR6142-5p | Cc00_g25510 | 2.0 | 13.118 | 1 | 20 | 163 | 182 | GACGAUUUUUUAAGAUAUGA | UUAUAUCUUAAAAAAUGGUC | Cleavage |
| ccp-miR6142-5p | Cc01_g02330 | 3.0 | 19.591 | 1 | 23 | 634 | 656 | GACGAUUUUUUAAGAUAUGACUA | UUGACAUAUCUGAAAAGAUCGUU | Cleavage |
| ccp-miR6142-5p | Cc02_g31230 | 3.0 | 12.702 | 1 | 22 | 3513 | 3534 | GACGAUUUUUUAAGAUAUGACU | ACUCCAAUUUUGAAAAAUCGUC | Cleavage |
| ccp-miR6142-5p | Cc04_g08240 | 3.0 | 14.871 | 1 | 22 | 1846 | 1868 | GACGAUU-UUUUAAGAUAUGACU | AGGCAUGUCUUAAAAUAAUUGUC | Cleavage |
| ccp-miR6142-5p | Cc06_g21730 | 3.0 | 16.059 | 1 | 22 | 1589 | 1610 | GACGAUUUUUUAAGAUAUGACU | AUUGAUGUUUUAGAAAGUCGUC | Cleavage |
| ccp-miR6142-5p | Cc08_g02360 | 3.0 | 9.983 | 1 | 20 | 133 | 152 | GACGAUUUUUUAAGAUAUGA | UCAAGUCUUAACAAGUCGUC | Translation |
| ccp-miR6142-5p | Cc09_g02370 | 3.0 | 16.285 | 1 | 20 | 1332 | 1351 | GACGAUUUUUUAAGAUAUGA | UCAUAUCUUAGAAGGUCAUC | Cleavage |
| ccp-miR6142-5p | Cc09_g09140 | 3.0 | 12.495 | 1 | 22 | 3297 | 3318 | GACGAUUUUUUAAGAUAUGACU | ACUCCAAUUUUGAAAAAUCGUC | Cleavage |
| ccp-miR6142-5p | Cc10_g13720 | 2.0 | 8.316 | 1 | 20 | 432 | 451 | GACGAUUUUUUAAGAUAUGA | ACAUAUCUUAAAAAAUUGUU | Cleavage |
| ccp-miR6188-3p | Cc02_g33720 | 3.0 | 8.024 | 1 | 21 | 92 | 112 | GGUGGAUCAAUGAACCCAGCU | GGCUAGGUUCAUUCAUUCACU | Cleavage |
| ccp-miR6188-3p | Cc03_g16310 | 3.0 | 22.221 | 1 | 21 | 1944 | 1964 | GGUGGAUCAAUGAACCCAGCU | GGCUGGUUUUGUUGAUCCACA | Cleavage |
| ccp-miR6188-3p | Cc04_g06210 | 2.5 | 16.833 | 1 | 21 | 1945 | 1965 | GGUGGAUCAAUGAACCCAGCU | GGUUGAGUUCAUUGAUUCACU | Cleavage |
| ccp-miR6188-3p | Cc08_g01700 | 3.0 | 14.739 | 1 | 21 | 3844 | 3864 | GGUGGAUCAAUGAACCCAGCU | GGCUGGAUUUAAUGAUCCAUC | Translation |
| ccp-miR6198-5p | Cc00_g13960 | 3.0 | 15.96 | 1 | 20 | 2477 | 2496 | GCUCUGUCUUGGCUGGACAU | UUGUCAAACCAAGACAGAGC | Cleavage |
| ccp-miR6198-5p | Cc11_g05930 | 3.0 | 14.102 | 1 | 20 | 126 | 145 | GCUCUGUCUUGGCUGGACAU | AUGUACAUACAAGACAGAGC | Cleavage |
| ccp-miR6253-3p | Cc02_g10420 | 3.0 | 3.252 | 1 | 20 | 393 | 412 | GAGGAAAGUGGAGUGUUGGG | CCCACCGCACCACUUUCUUC | Cleavage |
| ccp-miR6253-3p | Cc02_g27630 | 1.5 | 13.539 | 1 | 22 | 404 | 425 | GAGGAAAGUGGAGUGUUGGGUU | AUUCCAACACUUCAUUUUCCUC | Cleavage |
| ccp-miR6253-3p | Cc02_g34700 | 3.0 | 23.715 | 1 | 22 | 446 | 468 | GAGGAAAGUGGA-GUGUUGGGUU | GGCCCAAUACCUUCACUUUCCUC | Cleavage |
| ccp-miR6253-3p | Cc04_g02800 | 3.0 | 9.746 | 1 | 20 | 154 | 173 | GAGGAAAGUGGAGUGUUGGG | CCCAACACUCUACGUUUCUU | Cleavage |
| ccp-miR6253-3p | Cc04_g07000 | 3.0 | 20.547 | 1 | 22 | 108 | 129 | GAGGAAAGUGGAGUGUUGGGUU | ACCCGAAUCCUCCACUUUUCUC | Cleavage |
| ccp-miR6253-3p | Cc05_g11650 | 3.0 | 1.2 | 1 | 20 | 33 | 52 | GAGGAAAGUGGAGUGUUGGG | CCUAACAUUCUCCUUUCUUC | Translation |
| ccp-miR6253-3p | Cc06_g12470 | 2.5 | 10.941 | 1 | 20 | 1711 | 1730 | GAGGAAAGUGGAGUGUUGGG | UCCAACAUUCUGCUUUCUUC | Cleavage |
| ccp-miR6253-3p | Cc07_g05170 | 3.0 | 14.246 | 1 | 22 | 775 | 796 | GAGGAAAGUGGAGUGUUGGGUU | GGUCCAACCCUUCACUUUCUUU | Cleavage |
| ccp-miR6253-3p | Cc07_g15690 | 3.0 | 3.847 | 1 | 22 | 260 | 281 | GAGGAAAGUGGAGUGUUGGGUU | AUUCCGCCGCUCCACUUUCUUC | Cleavage |
| ccp-miR6253-3p | Cc08_g12400 | 3.0 | 1.292 | 1 | 22 | 103 | 124 | GAGGAAAGUGGAGUGUUGGGUU | ACCCCCACAUUCCACUUCCCUC | Cleavage |
| ccp-miR6253-3p | Cc09_g01640 | 3.0 | 13.029 | 1 | 20 | 4509 | 4528 | GAGGAAAGUGGAGUGUUGGG | CCCAAGGUUCUACUUUCUUC | Cleavage |
| ccp-miR6253-3p | Cc09_g09190 | 2.5 | 16.134 | 1 | 21 | 375 | 395 | GAGGAAAGUGGAGUGUUGGGU | ACCCAACUCUUCAAUUUCCUC | Cleavage |
| ccp-miR6253-3p | Cc11_g16600 | 2.5 | 16.089 | 1 | 20 | 1223 | 1242 | GAGGAAAGUGGAGUGUUGGG | CCUGAUACUCCACUUUUUUC | Cleavage |
| ccp-miR6281-3p | Cc00_g01360 | 2.5 | 7.457 | 1 | 21 | 78 | 98 | GUUAGAGAGAGAGAGAGAGAG | CUCUCUCUCUCUCUCUCUACA | Cleavage |
| ccp-miR6281-3p | Cc00_g02290 | 1.5 | 3.624 | 1 | 21 | 13 | 33 | GUUAGAGAGAGAGAGAGAGAG | CUCUCUCUCUCUCUCUCUAUC | Cleavage |
| ccp-miR6281-3p | Cc00_g02320 | 2.5 | 7.228 | 1 | 21 | 1791 | 1811 | GUUAGAGAGAGAGAGAGAGAG | CUCUCUCUCUCUCUCUCUACU | Cleavage |
| ccp-miR6281-3p | Cc00_g03220 | 3.0 | 1.179 | 1 | 21 | 538 | 558 | GUUAGAGAGAGAGAGAGAGAG | CUCUCUCUCUCUCUCUCUGUA | Cleavage |
| ccp-miR6281-3p | Cc00_g03820 | 1.5 | 3.204 | 1 | 21 | 18 | 38 | GUUAGAGAGAGAGAGAGAGAG | CUCUCUCUCUCUCUCUCUCAC | Cleavage |
| ccp-miR6281-3p | Cc00_g04600 | 1.5 | 5.524 | 1 | 21 | 2072 | 2092 | GUUAGAGAGAGAGAGAGAGAG | CUCUCUCUCUCUCUCUCUAUC | Cleavage |
| ccp-miR6281-3p | Cc00_g13530 | 2.5 | 1.473 | 1 | 21 | 579 | 599 | GUUAGAGAGAGAGAGAGAGAG | CUCUCUCUCUCUCUCUCUAUU | Cleavage |
| ccp-miR6281-3p | Cc00_g15010 | 2.0 | 15.832 | 1 | 21 | 337 | 357 | GUUAGAGAGAGAGAGAGAGAG | CUCUCUCUCUCUCUCUCUCGC | Cleavage |
| ccp-miR6281-3p | Cc00_g16470 | 1.5 | 11.097 | 1 | 21 | 4 | 24 | GUUAGAGAGAGAGAGAGAGAG | CUCUCUCUCUCUCUCUCUUAC | Cleavage |
| ccp-miR6281-3p | Cc00_g17030 | 0.5 | 5.377 | 1 | 21 | 27 | 47 | GUUAGAGAGAGAGAGAGAGAG | CUCUCUCUCUCUCUCUCUAAU | Cleavage |
| ccp-miR6281-3p | Cc00_g21210 | 1.5 | 4.775 | 1 | 21 | 97 | 117 | GUUAGAGAGAGAGAGAGAGAG | CUCUCUCUCUUUCUCUCUAAA | Cleavage |
| ccp-miR6281-3p | Cc00_g24380 | 3.0 | 6.354 | 1 | 21 | 51 | 71 | GUUAGAGAGAGAGAGAGAGAG | CUCUCUCUCUCUCUCUCUGUU | Cleavage |
| ccp-miR6281-3p | Cc00_g26380 | 1.5 | 16.948 | 1 | 21 | 1174 | 1194 | GUUAGAGAGAGAGAGAGAGAG | CUCUCUCUCUCUCUUUCUAGU | Cleavage |
| ccp-miR6281-3p | Cc00_g26740 | 2.5 | 7.34 | 1 | 21 | 346 | 366 | GUUAGAGAGAGAGAGAGAGAG | CUCUCUCUCUCUCUCUCUACG | Cleavage |
| ccp-miR6281-3p | Cc00_g26810 | 3.0 | 10.079 | 1 | 21 | 1669 | 1689 | GUUAGAGAGAGAGAGAGAGAG | CUCUCUCUCUCUCUCUCUGUA | Cleavage |
| ccp-miR6281-3p | Cc00_g33820 | 1.0 | 5.745 | 1 | 21 | 27 | 47 | GUUAGAGAGAGAGAGAGAGAG | CUCUCUCUCUCUCUCUCUAAG | Cleavage |
| ccp-miR6281-3p | Cc01_g04520 | 3.0 | 17.357 | 1 | 21 | 2312 | 2332 | GUUAGAGAGAGAGAGAGAGAG | CUCUCUCUCUCUCUCUCUGUU | Cleavage |
| ccp-miR6281-3p | Cc01_g04920 | 3.0 | 12.378 | 1 | 21 | 2121 | 2141 | GUUAGAGAGAGAGAGAGAGAG | CUCUCUCUCUCUCUCUCUGUG | Cleavage |
| ccp-miR6281-3p | Cc01_g05320 | 1.5 | 4.599 | 1 | 21 | 24 | 44 | GUUAGAGAGAGAGAGAGAGAG | CUCUCUCUCUCUCUCUCUAUC | Cleavage |
| ccp-miR6281-3p | Cc01_g05900 | 0.5 | 11.404 | 1 | 21 | 179 | 199 | GUUAGAGAGAGAGAGAGAGAG | CUCUCUCUCUCUCUCUCUAAU | Cleavage |
| ccp-miR6281-3p | Cc01_g07580 | 2.0 | 2.295 | 1 | 21 | 121 | 141 | GUUAGAGAGAGAGAGAGAGAG | CUCUCUCUCUUUCUCUCUCAC | Cleavage |
| ccp-miR6281-3p | Cc01_g08910 | 2.0 | 7.774 | 1 | 21 | 790 | 810 | GUUAGAGAGAGAGAGAGAGAG | CUCUCUCUCUCUCUCUCUCAU | Cleavage |
| ccp-miR6281-3p | Cc01_g10470 | 1.5 | 13.309 | 1 | 21 | 401 | 421 | GUUAGAGAGAGAGAGAGAGAG | CUCUCUCUCUCUCUCUCUGAA | Cleavage |
| ccp-miR6281-3p | Cc01_g11670 | 2.5 | 4.111 | 1 | 21 | 27 | 47 | GUUAGAGAGAGAGAGAGAGAG | CUCUCUCUCUCUCUCUCUAUA | Cleavage |
| ccp-miR6281-3p | Cc01_g13400 | 2.0 | 18.231 | 1 | 21 | 1752 | 1772 | GUUAGAGAGAGAGAGAGAGAG | CUCUCUCUCUCUCUCUCUGGG | Cleavage |
| ccp-miR6281-3p | Cc01_g14650 | 1.5 | 6.511 | 1 | 20 | 58 | 77 | GUUAGAGAGAGAGAGAGAGA | UCUCUCUCUCUCUCUCUGAA | Cleavage |
| ccp-miR6281-3p | Cc01_g14710 | 1.5 | 4.987 | 1 | 21 | 4326 | 4346 | GUUAGAGAGAGAGAGAGAGAG | CUCUCUCUCUCUCUCUCUAUC | Cleavage |
| ccp-miR6281-3p | Cc01_g15130 | 3.0 | 6.174 | 1 | 21 | 27 | 47 | GUUAGAGAGAGAGAGAGAGAG | CUCUCUCUCUCUCUCUCUGUA | Cleavage |
| ccp-miR6281-3p | Cc01_g15330 | 2.0 | 6.434 | 1 | 21 | 90 | 110 | GUUAGAGAGAGAGAGAGAGAG | CUCUCUCUCUCUCUCUCUCAU | Cleavage |
| ccp-miR6281-3p | Cc01_g16660 | 1.0 | 3.384 | 1 | 21 | 41 | 61 | GUUAGAGAGAGAGAGAGAGAG | CUCUCUUUCUCUCUCUCUAGC | Cleavage |
| ccp-miR6281-3p | Cc01_g17240 | 1.5 | 6.467 | 1 | 21 | 153 | 173 | GUUAGAGAGAGAGAGAGAGAG | CUUUCUCUCUCUCUCUUUAGC | Cleavage |
| ccp-miR6281-3p | Cc02_g00500 | 1.5 | 3.619 | 1 | 21 | 736 | 756 | GUUAGAGAGAGAGAGAGAGAG | CUCUCUCUCUCUCUCUCUCAC | Cleavage |
| ccp-miR6281-3p | Cc02_g00700 | 1.5 | 13.499 | 1 | 21 | 52 | 72 | GUUAGAGAGAGAGAGAGAGAG | CUCUCUCUCUCUCUCUCUAGA | Cleavage |
| ccp-miR6281-3p | Cc02_g01620 | 1.5 | 7.862 | 1 | 21 | 193 | 213 | GUUAGAGAGAGAGAGAGAGAG | CUCUCUCUCUCUCUCUCUAUC | Cleavage |
| ccp-miR6281-3p | Cc02_g01740 | 3.0 | 16.133 | 1 | 21 | 176 | 196 | GUUAGAGAGAGAGAGAGAGAG | CUCUCUCUCUCUCUCUCUGUG | Cleavage |
| ccp-miR6281-3p | Cc02_g01740 | 3.0 | 13.22 | 1 | 21 | 381 | 401 | GUUAGAGAGAGAGAGAGAGAG | CUCUCUCUCUCUCUCUCUGUU | Cleavage |
| ccp-miR6281-3p | Cc02_g01990 | 2.0 | 9.952 | 1 | 21 | 2438 | 2458 | GUUAGAGAGAGAGAGAGAGAG | CUCUCUCUCUCUCUCUCAAAU | Cleavage |
| ccp-miR6281-3p | Cc02_g03340 | 2.0 | 10.154 | 1 | 21 | 1043 | 1063 | GUUAGAGAGAGAGAGAGAGAG | CUCUCUCUCUCUCUCUCUGCC | Cleavage |
| ccp-miR6281-3p | Cc02_g04400 | 1.5 | 10.244 | 1 | 21 | 3715 | 3735 | GUUAGAGAGAGAGAGAGAGAG | CUCUCUCUCUCUCUCUCUAGG | Cleavage |
| ccp-miR6281-3p | Cc02_g05060 | 2.5 | 8.502 | 1 | 21 | 6 | 26 | GUUAGAGAGAGAGAGAGAGAG | CUCUCUCUCUCUCUCUCUAUU | Cleavage |
| ccp-miR6281-3p | Cc02_g06490 | 1.5 | 5.448 | 1 | 21 | 73 | 93 | GUUAGAGAGAGAGAGAGAGAG | CUCUCUCUCUCUCUCUCUGAA | Cleavage |
| ccp-miR6281-3p | Cc02_g07890 | 1.5 | 11.583 | 1 | 21 | 1713 | 1733 | GUUAGAGAGAGAGAGAGAGAG | CUCUCUCUCUCUCUCUCUGAG | Cleavage |
| ccp-miR6281-3p | Cc02_g09670 | 2.0 | 12.495 | 1 | 21 | 113 | 133 | GUUAGAGAGAGAGAGAGAGAG | CUCUUUCUUUCUCUCUCUGAU | Cleavage |
| ccp-miR6281-3p | Cc02_g10070 | 1.5 | 4.224 | 1 | 21 | 41 | 61 | GUUAGAGAGAGAGAGAGAGAG | CUCUCUCUCUCUCUCUCUUAC | Cleavage |
| ccp-miR6281-3p | Cc02_g11750 | 1.5 | 14.653 | 1 | 21 | 439 | 459 | GUUAGAGAGAGAGAGAGAGAG | CUCUCUCUCUCUCUCUCUAUC | Cleavage |
| ccp-miR6281-3p | Cc02_g13510 | 2.0 | 3.335 | 1 | 21 | 282 | 302 | GUUAGAGAGAGAGAGAGAGAG | CUCUCUCUCUUUCUCUCUCAC | Cleavage |
| ccp-miR6281-3p | Cc02_g13590 | 2.5 | 0.575 | 1 | 21 | 216 | 236 | GUUAGAGAGAGAGAGAGAGAG | CUCUCUCUCUCUCUCUCUACU | Cleavage |
| ccp-miR6281-3p | Cc02_g17060 | 2.5 | 6.015 | 1 | 21 | 94 | 114 | GUUAGAGAGAGAGAGAGAGAG | CUCUCUCUCUCUCUCUCUACU | Cleavage |
| ccp-miR6281-3p | Cc02_g17280 | 1.0 | 6.733 | 1 | 21 | 105 | 125 | GUUAGAGAGAGAGAGAGAGAG | CUCUCUCUCUCUCUCUCUAAA | Cleavage |
| ccp-miR6281-3p | Cc02_g18450 | 2.0 | 7.955 | 1 | 21 | 166 | 186 | GUUAGAGAGAGAGAGAGAGAG | CUCUCUCUCUCUCUCUCUCAU | Cleavage |
| ccp-miR6281-3p | Cc02_g19340 | 3.0 | 15.844 | 1 | 21 | 2781 | 2801 | GUUAGAGAGAGAGAGAGAGAG | CUCUCUCUCUCUCUCUCUGUG | Cleavage |
| ccp-miR6281-3p | Cc02_g19760 | 1.5 | 5.068 | 1 | 21 | 168 | 188 | GUUAGAGAGAGAGAGAGAGAG | CUCUCUCUCUCUCUCUCUCAC | Cleavage |
| ccp-miR6281-3p | Cc02_g19870 | 1.0 | 5.931 | 1 | 21 | 34 | 54 | GUUAGAGAGAGAGAGAGAGAG | UUCUCUCUCUCUCUCUCUAAA | Cleavage |
| ccp-miR6281-3p | Cc02_g19880 | 2.5 | 8.137 | 1 | 21 | 223 | 243 | GUUAGAGAGAGAGAGAGAGAG | CUCUCUCUCUCUCUCUCUAUG | Cleavage |
| ccp-miR6281-3p | Cc02_g21230 | 3.0 | 15.253 | 1 | 21 | 2863 | 2883 | GUUAGAGAGAGAGAGAGAGAG | CUCUCUCUCUCUCUCUCUGUU | Cleavage |
| ccp-miR6281-3p | Cc02_g21520 | 2.0 | 3.137 | 1 | 21 | 55 | 75 | GUUAGAGAGAGAGAGAGAGAG | CUCUCUCUCUCUCUCUCUCAU | Cleavage |
| ccp-miR6281-3p | Cc02_g22000 | 2.0 | 3.004 | 1 | 21 | 27 | 47 | GUUAGAGAGAGAGAGAGAGAG | CUCUCUCUCUCUCUCUCUGCC | Cleavage |
| ccp-miR6281-3p | Cc02_g22960 | 2.0 | 0.965 | 1 | 21 | 225 | 245 | GUUAGAGAGAGAGAGAGAGAG | CUCUCUCUCUCUCUCUCUGUC | Cleavage |
| ccp-miR6281-3p | Cc02_g22960 | 2.0 | 0.6 | 1 | 21 | 275 | 295 | GUUAGAGAGAGAGAGAGAGAG | CUCUCUCUCUCUCUCUCUGUC | Cleavage |
| ccp-miR6281-3p | Cc02_g23550 | 3.0 | 11.81 | 1 | 21 | 3973 | 3993 | GUUAGAGAGAGAGAGAGAGAG | CUCUCUCUCUCUCUCUCUGUG | Cleavage |
| ccp-miR6281-3p | Cc02_g25660 | 2.0 | 9.793 | 1 | 21 | 32 | 52 | GUUAGAGAGAGAGAGAGAGAG | CUCUCUCUCUCUCUCUCUGGA | Cleavage |
| ccp-miR6281-3p | Cc02_g26940 | 0.5 | 3.504 | 1 | 21 | 139 | 159 | GUUAGAGAGAGAGAGAGAGAG | UUCUCUCUCUCUCUUUCUAAC | Cleavage |
| ccp-miR6281-3p | Cc02_g27090 | 2.0 | 9.153 | 1 | 21 | 148 | 168 | GUUAGAGAGAGAGAGAGAGAG | CUCUCUCUCUCUCUCUCUCGC | Cleavage |
| ccp-miR6281-3p | Cc02_g28070 | 1.5 | 7.524 | 1 | 21 | 987 | 1007 | GUUAGAGAGAGAGAGAGAGAG | CUCUCUCUCUCUCUCUCUGAA | Cleavage |
| ccp-miR6281-3p | Cc02_g30840 | 1.5 | 11.046 | 1 | 21 | 79 | 99 | GUUAGAGAGAGAGAGAGAGAG | CUCUCUCUCUCUCUCUCUAUC | Cleavage |
| ccp-miR6281-3p | Cc02_g34850 | 2.0 | 6.558 | 1 | 21 | 9 | 29 | GUUAGAGAGAGAGAGAGAGAG | CUCUUUCUUUCUCUCUCUAAA | Cleavage |
| ccp-miR6281-3p | Cc02_g35780 | 1.0 | 12.841 | 1 | 21 | 50 | 70 | GUUAGAGAGAGAGAGAGAGAG | CUCUCUCUCUCUCUCUCUAAG | Cleavage |
| ccp-miR6281-3p | Cc02_g36820 | 2.0 | 13.566 | 1 | 21 | 2025 | 2045 | GUUAGAGAGAGAGAGAGAGAG | CUCUCUCUCUCUUUCUUUAGU | Cleavage |
| ccp-miR6281-3p | Cc02_g37380 | 1.5 | 3.067 | 1 | 21 | 43 | 63 | GUUAGAGAGAGAGAGAGAGAG | CUCUCUCUCUCUCUCUCUCAC | Cleavage |
| ccp-miR6281-3p | Cc02_g39180 | 2.0 | 8.026 | 1 | 21 | 276 | 296 | GUUAGAGAGAGAGAGAGAGAG | CUCUCUCUCUCUCUCUCUCGC | Cleavage |
| ccp-miR6281-3p | Cc02_g39180 | 2.0 | 5.695 | 1 | 21 | 516 | 536 | GUUAGAGAGAGAGAGAGAGAG | CUCUCUCUCUCUCUCUCUCGC | Cleavage |
| ccp-miR6281-3p | Cc02_g39470 | 1.5 | 4.382 | 1 | 21 | 577 | 597 | GUUAGAGAGAGAGAGAGAGAG | CUCUCUCUCUCUCUCUCUAUC | Cleavage |
| ccp-miR6281-3p | Cc02_g39510 | 3.0 | 7.569 | 1 | 21 | 1094 | 1114 | GUUAGAGAGAGAGAGAGAGAG | CUCUCUCUCUCUCUCUCUGUG | Cleavage |
| ccp-miR6281-3p | Cc03_g00370 | 2.0 | 15.12 | 1 | 21 | 254 | 274 | GUUAGAGAGAGAGAGAGAGAG | CUCUCUCUCUCUCUCUCUCGC | Cleavage |
| ccp-miR6281-3p | Cc03_g00390 | 2.0 | 10.146 | 1 | 21 | 3 | 23 | GUUAGAGAGAGAGAGAGAGAG | CUCUCUCUCUCUCUCUCUCGC | Cleavage |
| ccp-miR6281-3p | Cc03_g00650 | 2.0 | 15.281 | 1 | 21 | 2424 | 2444 | GUUAGAGAGAGAGAGAGAGAG | CUCUCUCUCUCUCUCUCUCAU | Cleavage |
| ccp-miR6281-3p | Cc03_g01450 | 3.0 | 0.798 | 1 | 21 | 135 | 155 | GUUAGAGAGAGAGAGAGAGAG | CUCUCUCUCUCUCUCUCUGCU | Cleavage |
| ccp-miR6281-3p | Cc03_g01640 | 1.5 | 1.813 | 1 | 21 | 96 | 116 | GUUAGAGAGAGAGAGAGAGAG | CUCUCUCUCUCUCUCUCUAUC | Cleavage |
| ccp-miR6281-3p | Cc03_g01820 | 2.5 | 7.47 | 1 | 21 | 63 | 83 | GUUAGAGAGAGAGAGAGAGAG | CUCUCUCUCUCUCUCUCUACA | Cleavage |
| ccp-miR6281-3p | Cc03_g02100 | 2.0 | 18.443 | 1 | 21 | 1 | 21 | GUUAGAGAGAGAGAGAGAGAG | CUCUCUCUCUCUCUCUCUCGC | Cleavage |
| ccp-miR6281-3p | Cc03_g04000 | 1.0 | 18.731 | 1 | 21 | 227 | 247 | GUUAGAGAGAGAGAGAGAGAG | CUCUCUCUCUCUCUCUCUGAU | Cleavage |
| ccp-miR6281-3p | Cc03_g04100 | 1.0 | 5.907 | 1 | 21 | 34 | 54 | GUUAGAGAGAGAGAGAGAGAG | CUCUCUCUCUCUCUCUUUAAU | Cleavage |
| ccp-miR6281-3p | Cc03_g08040 | 2.5 | 22.493 | 1 | 21 | 766 | 786 | GUUAGAGAGAGAGAGAGAGAG | CUCUCUCUCUCUCUCUCUACA | Cleavage |
| ccp-miR6281-3p | Cc03_g09590 | 1.5 | 1.264 | 1 | 21 | 5 | 25 | GUUAGAGAGAGAGAGAGAGAG | CUCUCUCUCUCUCUCUCUGAA | Cleavage |
| ccp-miR6281-3p | Cc03_g10730 | 2.0 | 6.01 | 1 | 21 | 34 | 54 | GUUAGAGAGAGAGAGAGAGAG | CUCUCUCUCUCUCUCUCUGCC | Cleavage |
| ccp-miR6281-3p | Cc04_g00240 | 2.0 | 0.635 | 1 | 21 | 431 | 451 | GUUAGAGAGAGAGAGAGAGAG | CUCUCUCUCUCUCUCUCUUAU | Cleavage |
| ccp-miR6281-3p | Cc04_g00240 | 2.0 | 3.286 | 1 | 21 | 964 | 984 | GUUAGAGAGAGAGAGAGAGAG | CUCUCUCUCUCUCUCUCUUAU | Cleavage |
| ccp-miR6281-3p | Cc04_g00290 | 2.0 | 15.299 | 1 | 21 | 180 | 200 | GUUAGAGAGAGAGAGAGAGAG | CUCUCUCUCUCUCUCUCUGUC | Cleavage |
| ccp-miR6281-3p | Cc04_g00940 | 2.0 | 2.963 | 1 | 21 | 1138 | 1158 | GUUAGAGAGAGAGAGAGAGAG | CUCUCUCUCUCUCUCUCUCGC | Cleavage |
| ccp-miR6281-3p | Cc04_g02150 | 3.0 | 17.245 | 1 | 21 | 329 | 349 | GUUAGAGAGAGAGAGAGAGAG | CUCUCUCUCUCUCUCUCUGCU | Cleavage |
| ccp-miR6281-3p | Cc04_g05010 | 3.0 | 15.737 | 1 | 21 | 251 | 271 | GUUAGAGAGAGAGAGAGAGAG | CUCUCUCUCUUUCUCUCUAUA | Cleavage |
| ccp-miR6281-3p | Cc04_g06050 | 2.5 | 4.781 | 1 | 21 | 122 | 142 | GUUAGAGAGAGAGAGAGAGAG | UUCUCUCUCUCUCUCUCUACA | Cleavage |
| ccp-miR6281-3p | Cc04_g06920 | 2.0 | 9.397 | 1 | 21 | 212 | 232 | GUUAGAGAGAGAGAGAGAGAG | CUCUCUCUCUCUCUCUCUCGC | Cleavage |
| ccp-miR6281-3p | Cc04_g07130 | 3.0 | 15.247 | 1 | 21 | 1171 | 1191 | GUUAGAGAGAGAGAGAGAGAG | CUCUCUCUCUCUCUCUCUGUU | Cleavage |
| ccp-miR6281-3p | Cc04_g07200 | 2.0 | 9.817 | 1 | 21 | 157 | 177 | GUUAGAGAGAGAGAGAGAGAG | CUCUCUCUCUCUCUCUCUGGA | Cleavage |
| ccp-miR6281-3p | Cc04_g09020 | 3.0 | 10.129 | 1 | 21 | 182 | 202 | GUUAGAGAGAGAGAGAGAGAG | CUCUCUCUCUCUCUUUCUAUA | Cleavage |
| ccp-miR6281-3p | Cc04_g11740 | 1.5 | 9.935 | 1 | 21 | 47 | 67 | GUUAGAGAGAGAGAGAGAGAG | CUCUCUCUCUCUCUCUCUCAC | Cleavage |
| ccp-miR6281-3p | Cc04_g12200 | 1.0 | 8.299 | 1 | 21 | 61 | 81 | GUUAGAGAGAGAGAGAGAGAG | CUCUCUCUCUCUCUCUCUGAU | Cleavage |
| ccp-miR6281-3p | Cc04_g13400 | 2.0 | 16.945 | 1 | 21 | 1043 | 1063 | GUUAGAGAGAGAGAGAGAGAG | CUCUCUCUCUCUCUCUCUCAU | Cleavage |
| ccp-miR6281-3p | Cc04_g16770 | 2.0 | 5.906 | 1 | 21 | 123 | 143 | GUUAGAGAGAGAGAGAGAGAG | CUCUCUCUCUCUCUCUCAAGC | Cleavage |
| ccp-miR6281-3p | Cc05_g02310 | 2.5 | 6.878 | 1 | 21 | 3708 | 3728 | GUUAGAGAGAGAGAGAGAGAG | CUCUCUCUCUCUCUCUCUAUG | Cleavage |
| ccp-miR6281-3p | Cc05_g02360 | 3.0 | 9.4 | 1 | 21 | 4792 | 4812 | GUUAGAGAGAGAGAGAGAGAG | CUCUCUCUCUCUCUCUCUGUG | Cleavage |
| ccp-miR6281-3p | Cc05_g06350 | 2.5 | 6.479 | 1 | 21 | 291 | 311 | GUUAGAGAGAGAGAGAGAGAG | CUCUCUCUCUCUCUCUCUAUU | Cleavage |
| ccp-miR6281-3p | Cc05_g07030 | 1.5 | 1.383 | 1 | 21 | 55 | 75 | GUUAGAGAGAGAGAGAGAGAG | CUCUCUCUCUCUCUCUCUCAC | Cleavage |
| ccp-miR6281-3p | Cc05_g08120 | 1.5 | 6.799 | 1 | 21 | 8 | 28 | GUUAGAGAGAGAGAGAGAGAG | CCCUCUCUCUCUCUUUCUAAC | Cleavage |
| ccp-miR6281-3p | Cc05_g09440 | 2.0 | 7.482 | 1 | 21 | 2689 | 2709 | GUUAGAGAGAGAGAGAGAGAG | CUCUCUCUCUCUCUCUCUGUC | Cleavage |
| ccp-miR6281-3p | Cc05_g09920 | 2.0 | 4.814 | 1 | 21 | 43 | 63 | GUUAGAGAGAGAGAGAGAGAG | CUCUCUCUCUCUCUCUCUCGC | Cleavage |
| ccp-miR6281-3p | Cc05_g12780 | 2.0 | 6.065 | 1 | 21 | 2694 | 2714 | GUUAGAGAGAGAGAGAGAGAG | CUCUCUCUCUCUCUCUCUCGC | Cleavage |
| ccp-miR6281-3p | Cc05_g13290 | 2.0 | 11.243 | 1 | 21 | 56 | 76 | GUUAGAGAGAGAGAGAGAGAG | CUCUCUCUCUCUCUCUCUGUC | Cleavage |
| ccp-miR6281-3p | Cc05_g15890 | 1.5 | 5.526 | 1 | 21 | 455 | 475 | GUUAGAGAGAGAGAGAGAGAG | CUCUCUCUCUCUCUCUCUCAC | Cleavage |
| ccp-miR6281-3p | Cc05_g15980 | 3.0 | 1.276 | 1 | 21 | 248 | 268 | GUUAGAGAGAGAGAGAGAGAG | CUCUCUCUCUCUCUCUCUGUA | Cleavage |
| ccp-miR6281-3p | Cc05_g16490 | 2.0 | 11.148 | 1 | 21 | 582 | 602 | GUUAGAGAGAGAGAGAGAGAG | CUCUCUCUCUCUCUCUCUCGC | Cleavage |
| ccp-miR6281-3p | Cc06_g00250 | 0.5 | 8.079 | 1 | 21 | 203 | 223 | GUUAGAGAGAGAGAGAGAGAG | CUCUCUCUCUCUCUCUCUAGC | Cleavage |
| ccp-miR6281-3p | Cc06_g00410 | 3.0 | 6.827 | 1 | 21 | 232 | 252 | GUUAGAGAGAGAGAGAGAGAG | CUCUCUCUCUCUCUCUCUGCU | Cleavage |
| ccp-miR6281-3p | Cc06_g00410 | 3.0 | 9.173 | 1 | 21 | 292 | 312 | GUUAGAGAGAGAGAGAGAGAG | CUCUCUCUCUCUCUCUCUGCU | Cleavage |
| ccp-miR6281-3p | Cc06_g02880 | 3.0 | 9.686 | 1 | 21 | 31 | 51 | GUUAGAGAGAGAGAGAGAGAG | CUCUCUCUCUCUCUCUCUGUA | Cleavage |
| ccp-miR6281-3p | Cc06_g03130 | 1.5 | 14.194 | 1 | 21 | 394 | 414 | GUUAGAGAGAGAGAGAGAGAG | CUCUAUCUUUCUCUCUCUAAC | Cleavage |
| ccp-miR6281-3p | Cc06_g03510 | 2.0 | 7.515 | 1 | 21 | 1076 | 1096 | GUUAGAGAGAGAGAGAGAGAG | CUCUCUCUCUCUCUCUCUCAU | Cleavage |
| ccp-miR6281-3p | Cc06_g03780 | 0.0 | 9.827 | 1 | 21 | 139 | 159 | GUUAGAGAGAGAGAGAGAGAG | CUCUCUCUCUCUCUCUCUAAC | Cleavage |
| ccp-miR6281-3p | Cc06_g03850 | 1.5 | 14.68 | 1 | 21 | 147 | 167 | GUUAGAGAGAGAGAGAGAGAG | CUCUCUCUCUCUCUCUCUGAG | Cleavage |
| ccp-miR6281-3p | Cc06_g03920 | 2.0 | 12.749 | 1 | 21 | 920 | 940 | GUUAGAGAGAGAGAGAGAGAG | CUCUCUCUCUCUCUUUUUAGU | Cleavage |
| ccp-miR6281-3p | Cc06_g05040 | 1.0 | 10.796 | 1 | 21 | 16 | 36 | GUUAGAGAGAGAGAGAGAGAG | CUCUCUCUCUCUCUCUCUGAU | Cleavage |
| ccp-miR6281-3p | Cc06_g05560 | 2.0 | 16.848 | 1 | 21 | 695 | 715 | GUUAGAGAGAGAGAGAGAGAG | CUCUCUCUCUCUCUCUAUAGC | Cleavage |
| ccp-miR6281-3p | Cc06_g06810 | 1.5 | 20.643 | 1 | 21 | 101 | 121 | GUUAGAGAGAGAGAGAGAGAG | CUCUCUCUCUCUCUCUCUAGA | Cleavage |
| ccp-miR6281-3p | Cc06_g07160 | 2.0 | 6.201 | 1 | 21 | 102 | 122 | GUUAGAGAGAGAGAGAGAGAG | CUCUCUCUCUCUCUCUCUUGC | Cleavage |
| ccp-miR6281-3p | Cc06_g07160 | 2.0 | 15.605 | 1 | 21 | 167 | 187 | GUUAGAGAGAGAGAGAGAGAG | CUCUCUCUCUCUCUCUCUUGC | Cleavage |
| ccp-miR6281-3p | Cc06_g07220 | 2.0 | 1.473 | 1 | 21 | 133 | 153 | GUUAGAGAGAGAGAGAGAGAG | CUCUCUCUCUCUCUCUCUCGC | Cleavage |
| ccp-miR6281-3p | Cc06_g08170 | 3.0 | 13.038 | 1 | 21 | 157 | 177 | GUUAGAGAGAGAGAGAGAGAG | CUUUCUCUCUCUCUCUCUACG | Cleavage |
| ccp-miR6281-3p | Cc06_g08270 | 2.5 | 1.459 | 1 | 21 | 47 | 67 | GUUAGAGAGAGAGAGAGAGAG | CUCUCUCUCUCUCUCUCUAUA | Cleavage |
| ccp-miR6281-3p | Cc06_g08270 | 2.5 | 7.536 | 1 | 21 | 142 | 162 | GUUAGAGAGAGAGAGAGAGAG | CUCUCUCUCUCUCUCUCUAUA | Cleavage |
| ccp-miR6281-3p | Cc06_g08750 | 2.0 | 15.246 | 1 | 21 | 920 | 940 | GUUAGAGAGAGAGAGAGAGAG | CUCUCUCUCUCUCUCUCUCAU | Cleavage |
| ccp-miR6281-3p | Cc06_g13640 | 1.0 | 12.48 | 1 | 20 | 1927 | 1946 | GUUAGAGAGAGAGAGAGAGA | UCUCUCUCUCUCUCUCUAGU | Cleavage |
| ccp-miR6281-3p | Cc06_g14730 | 2.0 | 13.583 | 1 | 21 | 24 | 44 | GUUAGAGAGAGAGAGAGAGAG | CUCUCUCUCUCUCUCUCUCGC | Cleavage |
| ccp-miR6281-3p | Cc06_g15750 | 2.5 | 6.005 | 1 | 21 | 149 | 169 | GUUAGAGAGAGAGAGAGAGAG | CUCUCUCUCUCUCUCUCUACU | Cleavage |
| ccp-miR6281-3p | Cc06_g15870 | 2.0 | 5.977 | 1 | 21 | 19 | 39 | GUUAGAGAGAGAGAGAGAGAG | CUCUCUCUCUCUCUCUCUUAU | Cleavage |
| ccp-miR6281-3p | Cc06_g17420 | 3.0 | 2.408 | 1 | 21 | 1217 | 1237 | GUUAGAGAGAGAGAGAGAGAG | CUCUCUCUCUCUCUCUUUAUA | Cleavage |
| ccp-miR6281-3p | Cc06_g17920 | 1.5 | 17.965 | 1 | 21 | 1468 | 1488 | GUUAGAGAGAGAGAGAGAGAG | CUCUCUCUCUCUCUCUCUAUC | Cleavage |
| ccp-miR6281-3p | Cc06_g19660 | 3.0 | 8.22 | 1 | 21 | 2837 | 2857 | GUUAGAGAGAGAGAGAGAGAG | CUCUCUCUCUCUCUCUCUGUA | Cleavage |
| ccp-miR6281-3p | Cc06_g22710 | 3.0 | 8.359 | 1 | 21 | 2574 | 2594 | GUUAGAGAGAGAGAGAGAGAG | CUCUCUCUCUCUCUCUCUGCU | Cleavage |
| ccp-miR6281-3p | Cc06_g23740 | 3.0 | 18.252 | 1 | 21 | 1566 | 1586 | GUUAGAGAGAGAGAGAGAGAG | CUCUCUCUCUCUCUCUCUGCA | Cleavage |
| ccp-miR6281-3p | Cc07_g00760 | 1.5 | 11.154 | 1 | 21 | 672 | 692 | GUUAGAGAGAGAGAGAGAGAG | CUCUCUCUCUCUCUCUCUGGU | Cleavage |
| ccp-miR6281-3p | Cc07_g00920 | 1.0 | 9.071 | 1 | 21 | 692 | 712 | GUUAGAGAGAGAGAGAGAGAG | CUCUCUCUCUCUCUCUCUGAU | Cleavage |
| ccp-miR6281-3p | Cc07_g00990 | 2.0 | 15.298 | 1 | 21 | 9 | 29 | GUUAGAGAGAGAGAGAGAGAG | CUCUCUCUCUCUCUCUCUUAU | Cleavage |
| ccp-miR6281-3p | Cc07_g01100 | 1.0 | 22.279 | 1 | 21 | 528 | 548 | GUUAGAGAGAGAGAGAGAGAG | UUCUCUCUCUCUCUCUCUGAU | Cleavage |
| ccp-miR6281-3p | Cc07_g01250 | 1.5 | 14.036 | 1 | 21 | 6 | 26 | GUUAGAGAGAGAGAGAGAGAG | CUCUCUCUCUCUCUCUCUAUC | Cleavage |
| ccp-miR6281-3p | Cc07_g01830 | 2.5 | 6.145 | 1 | 21 | 121 | 141 | GUUAGAGAGAGAGAGAGAGAG | CUCUCUCUCUCUCUCUCUACA | Cleavage |
| ccp-miR6281-3p | Cc07_g02040 | 1.5 | 6.417 | 1 | 21 | 14 | 34 | GUUAGAGAGAGAGAGAGAGAG | CUCUCUCUCUCUCUCUCUCAC | Cleavage |
| ccp-miR6281-3p | Cc07_g02170 | 3.0 | 22.494 | 1 | 21 | 65 | 85 | GUUAGAGAGAGAGAGAGAGAG | CUCUCUCUCUCUCUCUCUGCG | Cleavage |
| ccp-miR6281-3p | Cc07_g02590 | 1.5 | 10.917 | 1 | 21 | 15 | 35 | GUUAGAGAGAGAGAGAGAGAG | CUCUCUCUCUCUCUCUCUCAC | Cleavage |
| ccp-miR6281-3p | Cc07_g03870 | 2.0 | 6.297 | 1 | 21 | 1262 | 1282 | GUUAGAGAGAGAGAGAGAGAG | CUCUCUCUCUCUCUCUUUUAC | Cleavage |
| ccp-miR6281-3p | Cc07_g03870 | 2.0 | 4.266 | 1 | 21 | 1334 | 1354 | GUUAGAGAGAGAGAGAGAGAG | CUCUCUCUCUCUCUCUCUCAU | Cleavage |
| ccp-miR6281-3p | Cc07_g03990 | 1.5 | 13.788 | 1 | 21 | 178 | 198 | GUUAGAGAGAGAGAGAGAGAG | CUCUCUCUCUCUCUCUCUUAC | Cleavage |
| ccp-miR6281-3p | Cc07_g04670 | 0.5 | 19.278 | 1 | 21 | 422 | 442 | GUUAGAGAGAGAGAGAGAGAG | CUCUCUCUCUCUCUUUCUAAC | Cleavage |
| ccp-miR6281-3p | Cc07_g06340 | 2.0 | 1.85 | 1 | 21 | 42 | 62 | GUUAGAGAGAGAGAGAGAGAG | CUCUCUCUCUCUCUCUCUGUC | Cleavage |
| ccp-miR6281-3p | Cc07_g06410 | 1.5 | 24.507 | 1 | 21 | 102 | 122 | GUUAGAGAGAGAGAGAGAGAG | CUCUCUCUCUCUCUCUCUAGG | Cleavage |
| ccp-miR6281-3p | Cc07_g09470 | 2.0 | 24.622 | 1 | 21 | 791 | 811 | GUUAGAGAGAGAGAGAGAGAG | CUCUCUCUCUCUCUCUUUGGU | Cleavage |
| ccp-miR6281-3p | Cc07_g10410 | 1.5 | 6.913 | 1 | 21 | 41 | 61 | GUUAGAGAGAGAGAGAGAGAG | CUCUCUCUCUCUCUCUCUAUC | Cleavage |
| ccp-miR6281-3p | Cc07_g11230 | 1.5 | 6.817 | 1 | 21 | 134 | 154 | GUUAGAGAGAGAGAGAGAGAG | CUCUCUCUCUCUCUCUCUAGA | Cleavage |
| ccp-miR6281-3p | Cc07_g11450 | 2.0 | 17.123 | 1 | 21 | 88 | 108 | GUUAGAGAGAGAGAGAGAGAG | CUCUCUCUCUCUCUCUCUGGG | Cleavage |
| ccp-miR6281-3p | Cc07_g12180 | 1.5 | 4.895 | 1 | 21 | 22 | 42 | GUUAGAGAGAGAGAGAGAGAG | CUCUCUCUCUCUCUCUCUCAC | Cleavage |
| ccp-miR6281-3p | Cc07_g12940 | 2.5 | 0.054 | 1 | 21 | 735 | 755 | GUUAGAGAGAGAGAGAGAGAG | CUCUCUCUCUCUCUCUCUACU | Cleavage |
| ccp-miR6281-3p | Cc07_g14850 | 1.5 | 14.97 | 1 | 21 | 26 | 46 | GUUAGAGAGAGAGAGAGAGAG | CUCUCUCUCUCUCUCUCUCAC | Cleavage |
| ccp-miR6281-3p | Cc07_g18220 | 1.5 | 6.632 | 1 | 21 | 144 | 164 | GUUAGAGAGAGAGAGAGAGAG | CUCUCUCUCUCUCUCUCUCAC | Cleavage |
| ccp-miR6281-3p | Cc08_g00200 | 3.0 | 16.856 | 1 | 21 | 1458 | 1478 | GUUAGAGAGAGAGAGAGAGAG | CUCUCUCUCUCUCUCUCUGUA | Cleavage |
| ccp-miR6281-3p | Cc08_g00280 | 2.0 | 21.181 | 1 | 21 | 168 | 188 | GUUAGAGAGAGAGAGAGAGAG | CUCUCUCUCUCUCUCUCUGGG | Cleavage |
| ccp-miR6281-3p | Cc08_g01390 | 1.5 | 0.827 | 1 | 21 | 12 | 32 | GUUAGAGAGAGAGAGAGAGAG | CUCUCUCUCUCUCUCUCAAAC | Cleavage |
| ccp-miR6281-3p | Cc08_g01930 | 1.5 | 6.817 | 1 | 21 | 134 | 154 | GUUAGAGAGAGAGAGAGAGAG | CUCUCUCUCUCUCUCUCUAGA | Cleavage |
| ccp-miR6281-3p | Cc08_g02110 | 1.5 | 3.733 | 1 | 21 | 9 | 29 | GUUAGAGAGAGAGAGAGAGAG | UUCUCUUUCUCUCUCUCUGGC | Cleavage |
| ccp-miR6281-3p | Cc08_g06860 | 1.5 | 6.295 | 1 | 21 | 56 | 76 | GUUAGAGAGAGAGAGAGAGAG | CUCUCUCUCUCUCUCUCUGAG | Cleavage |
| ccp-miR6281-3p | Cc08_g09840 | 1.5 | 7.021 | 1 | 21 | 95 | 115 | GUUAGAGAGAGAGAGAGAGAG | CUCUCUCUCUCUCUCUCUAUC | Cleavage |
| ccp-miR6281-3p | Cc08_g10400 | 2.0 | 7.992 | 1 | 21 | 12 | 32 | GUUAGAGAGAGAGAGAGAGAG | CUCUCUCUUUCUCUCUCUAGG | Cleavage |
| ccp-miR6281-3p | Cc08_g10530 | 2.0 | 18.551 | 1 | 21 | 1509 | 1529 | GUUAGAGAGAGAGAGAGAGAG | CUCUCUCUCUCUCUCUCUGGG | Cleavage |
| ccp-miR6281-3p | Cc08_g12100 | 3.0 | 3.271 | 1 | 21 | 50 | 70 | GUUAGAGAGAGAGAGAGAGAG | CUCUUUCUCUCUCUCUCUAUA | Cleavage |
| ccp-miR6281-3p | Cc08_g12810 | 1.5 | 8.179 | 1 | 21 | 46 | 66 | GUUAGAGAGAGAGAGAGAGAG | CUCUCUCUCUCUCUCUCUACC | Cleavage |
| ccp-miR6281-3p | Cc08_g13390 | 1.5 | 10.441 | 1 | 21 | 45 | 65 | GUUAGAGAGAGAGAGAGAGAG | CCCUCUCUCUCUCUCUCUGAC | Cleavage |
| ccp-miR6281-3p | Cc08_g15820 | 2.0 | 14.04 | 1 | 21 | 25 | 45 | GUUAGAGAGAGAGAGAGAGAG | CUCUCUUUCUCUCUCUCUGAA | Cleavage |
| ccp-miR6281-3p | Cc09_g02270 | 1.0 | 2.667 | 1 | 20 | 95 | 114 | GUUAGAGAGAGAGAGAGAGA | UCUCUCUCUCUCUCUCUGAU | Cleavage |
| ccp-miR6281-3p | Cc09_g02530 | 3.0 | 5.695 | 1 | 21 | 119 | 139 | GUUAGAGAGAGAGAGAGAGAG | CUCUCUCUCUCUCUCUCUGCA | Cleavage |
| ccp-miR6281-3p | Cc09_g03100 | 2.0 | 13.201 | 1 | 21 | 749 | 769 | GUUAGAGAGAGAGAGAGAGAG | CUCUCUCUCUCUCUCUCAAGC | Cleavage |
| ccp-miR6281-3p | Cc09_g03950 | 1.5 | 6.407 | 1 | 21 | 1300 | 1320 | GUUAGAGAGAGAGAGAGAGAG | UUCUCUCUCUCUCUCUCUCAC | Cleavage |
| ccp-miR6281-3p | Cc09_g04080 | 1.5 | 0.728 | 1 | 21 | 185 | 205 | GUUAGAGAGAGAGAGAGAGAG | CUCUCUCUCUCUCUCUCUAUC | Cleavage |
| ccp-miR6281-3p | Cc09_g10580 | 1.5 | 9.283 | 1 | 21 | 648 | 668 | GUUAGAGAGAGAGAGAGAGAG | UUCUCUCUCUCUCUCUCUCAC | Cleavage |
| ccp-miR6281-3p | Cc09_g10870 | 3.0 | 8.639 | 1 | 21 | 1 | 21 | GUUAGAGAGAGAGAGAGAGAG | CUCUCUCUCUCUCUCUCUGCG | Cleavage |
| ccp-miR6281-3p | Cc10_g01500 | 2.5 | 4.631 | 1 | 21 | 36 | 56 | GUUAGAGAGAGAGAGAGAGAG | CUCUCUCUCUCUCUCUCUAUA | Cleavage |
| ccp-miR6281-3p | Cc10_g02130 | 2.5 | 8.134 | 1 | 21 | 95 | 115 | GUUAGAGAGAGAGAGAGAGAG | CUCUCUCUCUCUCUCUCUACU | Cleavage |
| ccp-miR6281-3p | Cc10_g02920 | 2.0 | 18.204 | 1 | 21 | 13 | 33 | GUUAGAGAGAGAGAGAGAGAG | CUCUCUCUCUCUCUCUCUGCC | Cleavage |
| ccp-miR6281-3p | Cc10_g03680 | 1.5 | 1.006 | 1 | 21 | 28 | 48 | GUUAGAGAGAGAGAGAGAGAG | CUCUCUCUCUCUCUCUCUAUC | Cleavage |
| ccp-miR6281-3p | Cc10_g06630 | 2.0 | 15.171 | 1 | 21 | 64 | 84 | GUUAGAGAGAGAGAGAGAGAG | CUCUCUCUCUCUCUCUCUGUC | Cleavage |
| ccp-miR6281-3p | Cc10_g11210 | 2.0 | 13.503 | 1 | 21 | 1696 | 1716 | GUUAGAGAGAGAGAGAGAGAG | CUCUCUCUCUCUCUCUCUGUC | Cleavage |
| ccp-miR6281-3p | Cc10_g11210 | 2.0 | 20.556 | 1 | 21 | 1799 | 1819 | GUUAGAGAGAGAGAGAGAGAG | CUCUCUCUCUCUCUCUCUGUC | Cleavage |
| ccp-miR6281-3p | Cc10_g12010 | 1.5 | 14.444 | 1 | 21 | 546 | 566 | GUUAGAGAGAGAGAGAGAGAG | CUUUCUCUCUCUCUCUCUAAA | Cleavage |
| ccp-miR6281-3p | Cc10_g14440 | 1.5 | 0.705 | 1 | 21 | 120 | 140 | GUUAGAGAGAGAGAGAGAGAG | CUCUCUCUCUCUCUCUCUCAC | Cleavage |
| ccp-miR6281-3p | Cc10_g16080 | 0.0 | 4.268 | 1 | 20 | 2236 | 2255 | GUUAGAGAGAGAGAGAGAGA | UCUCUCUCUCUCUCUCUAAC | Cleavage |
| ccp-miR6281-3p | Cc10_g16160 | 1.5 | 9.08 | 1 | 21 | 2476 | 2496 | GUUAGAGAGAGAGAGAGAGAG | CUCUCUCUCUCUCUCUCUCAC | Cleavage |
| ccp-miR6281-3p | Cc11_g00350 | 1.5 | 4.839 | 1 | 21 | 61 | 81 | GUUAGAGAGAGAGAGAGAGAG | CUCUCUCUCUCUCUCUCUCAC | Cleavage |
| ccp-miR6281-3p | Cc11_g03200 | 1.5 | 2.267 | 1 | 21 | 370 | 390 | GUUAGAGAGAGAGAGAGAGAG | CUCUCUCUCUCUCUCUCUGAA | Cleavage |
| ccp-miR6281-3p | Cc11_g03540 | 1.5 | 9.612 | 1 | 21 | 1412 | 1432 | GUUAGAGAGAGAGAGAGAGAG | CUCUCUCUCUCUCUCUCUGAA | Cleavage |
| ccp-miR6281-3p | Cc11_g06700 | 2.0 | 11.71 | 1 | 21 | 17 | 37 | GUUAGAGAGAGAGAGAGAGAG | CUCUCUCUCUCUCUCUCUUGC | Cleavage |
| ccp-miR6281-3p | Cc11_g11150 | 1.5 | 6.296 | 1 | 21 | 1443 | 1463 | GUUAGAGAGAGAGAGAGAGAG | CUCUCUCUCUCUCUCUCUCAC | Cleavage |
| ccp-miR6281-3p | Cc11_g11940 | 1.0 | 2.386 | 1 | 21 | 1864 | 1884 | GUUAGAGAGAGAGAGAGAGAG | CUCUCUCUCUCUCUCUUUAAU | Cleavage |
| ccp-miR6281-3p | Cc11_g12500 | 0.5 | 10.885 | 1 | 21 | 280 | 300 | GUUAGAGAGAGAGAGAGAGAG | CUCUCUCUCUCUCUCUCUAAU | Cleavage |
| ccp-miR6281-3p | Cc11_g13250 | 1.0 | 10.845 | 1 | 21 | 3106 | 3126 | GUUAGAGAGAGAGAGAGAGAG | CUCUCUCUCUCUCUCUCUGAU | Cleavage |
| ccp-miR6281-3p | Cc11_g13580 | 2.0 | 7.487 | 1 | 21 | 83 | 103 | GUUAGAGAGAGAGAGAGAGAG | CUCUCUCUCUCUCUCUCUCAU | Cleavage |
| ccp-miR6281-3p | Cc11_g13860 | 2.0 | 9.744 | 1 | 21 | 1340 | 1360 | GUUAGAGAGAGAGAGAGAGAG | CUCUCUCUCUCUCUCUCUCAU | Cleavage |
| ccp-miR6281-3p | Cc11_g16060 | 1.0 | 8.109 | 1 | 21 | 46 | 66 | GUUAGAGAGAGAGAGAGAGAG | CUCUCUCUCUCUCUCUCUAAA | Cleavage |
| ccp-miR6281-3p | Cc11_g17000 | 1.0 | 12.543 | 1 | 21 | 76 | 96 | GUUAGAGAGAGAGAGAGAGAG | CUCUCUCUCUCUCUCUCUAAG | Cleavage |
| ccp-miR6281-3p | Cc11_g17460 | 1.5 | 12.735 | 1 | 21 | 2114 | 2134 | GUUAGAGAGAGAGAGAGAGAG | CUCUCUCUCUCUCUCUCUCAC | Cleavage |
| ccp-miR6440b-5p | Cc07_g02550 | 2.5 | 11.98 | 1 | 20 | 545 | 564 | GAGUUUGACCGAAUUCGAGU | ACUCUAAUUUGGUCAAAUUU | Cleavage |
| ccp-miR6440b-5p | Cc11_g06850 | 3.0 | 19.236 | 1 | 20 | 1155 | 1174 | GAGUUUGACCGAAUUCGAGU | GCUUGAAGUUGGUCAAACUU | Cleavage |
| ccp-miR6459a-2-5p | Cc00_g01320 | 3.0 | 14.88 | 1 | 20 | 3325 | 3344 | AGCUCAAGCUCAAAUUCGAU | AUUGGAUAUGAGUUUGAGUU | Cleavage |
| ccp-miR6459a-2-5p | Cc07_g14090 | 3.0 | 13.882 | 1 | 21 | 2022 | 2042 | AGCUCAAGCUCAAAUUCGAUU | AAUGGAAUUUGAAUUUGAGUU | Translation |
| ccp-miR6459a-2-5p | Cc08_g13100 | 3.0 | 15.308 | 1 | 21 | 429 | 449 | AGCUCAAGCUCAAAUUCGAUU | AAUCGAAUCUGAUGUUGAGCU | Translation |
| ccp-miR6459a-3-5p | Cc01_g19050 | 3.0 | 15.444 | 1 | 20 | 344 | 363 | AGCUCAAGCUCAAACUCGAG | UUUGGGUUUGGGUUUGAGUU | Cleavage |
| ccp-miR6459a-3-5p | Cc02_g24900 | 2.5 | 14.2 | 1 | 20 | 395 | 414 | AGCUCAAGCUCAAACUCGAG | UUUGAGGUUGAGUUUGAGCU | Cleavage |
| ccp-miR6459a-3-5p | Cc02_g31050 | 3.0 | 15.128 | 1 | 20 | 1511 | 1530 | AGCUCAAGCUCAAACUCGAG | CUUGAGUUUGGUUUUGAGUU | Translation |
| ccp-miR6459a-3-5p | Cc05_g10580 | 2.5 | 23.861 | 1 | 20 | 1475 | 1494 | AGCUCAAGCUCAAACUCGAG | CUAGAGUUUGAGUUUGAGCG | Cleavage |
| ccp-miR6459a-3-5p | Cc05_g14650 | 3.0 | 24.18 | 1 | 20 | 316 | 335 | AGCUCAAGCUCAAACUCGAG | CUUGGGCUUGGGCUUGGGCU | Cleavage |
| ccp-miR6459a-3-5p | Cc06_g17440 | 2.5 | 14.284 | 1 | 20 | 834 | 853 | AGCUCAAGCUCAAACUCGAG | UUAGAGUCUGAGCUUGAGCU | Cleavage |
| ccp-miR6459a-4-3p | Cc00_g29110 | 3.0 | 16.536 | 1 | 20 | 47 | 66 | UCGAGUUUGAGCUUGAGCUU | AAGCUCAAGCUCACAUUUGG | Cleavage |
| ccp-miR6459a-4-3p | Cc01_g01360 | 3.0 | 17.73 | 1 | 20 | 1326 | 1345 | UCGAGUUUGAGCUUGAGCUU | GAGCCCAAACUCGAACUCGA | Cleavage |
| ccp-miR6459a-4-3p | Cc01_g19150 | 2.5 | 23.488 | 1 | 20 | 50 | 69 | UCGAGUUUGAGCUUGAGCUU | GAGCUCAAGUUCGGACUUGA | Cleavage |
| ccp-miR6459a-4-3p | Cc02_g07110 | 2.0 | 23.098 | 1 | 20 | 1617 | 1636 | UCGAGUUUGAGCUUGAGCUU | GAGCUCGAACUCAAACUCGA | Cleavage |
| ccp-miR6459a-4-3p | Cc02_g25000 | 3.0 | 22.752 | 1 | 20 | 1573 | 1592 | UCGAGUUUGAGCUUGAGCUU | AAGCUGAAGUUUAGACUUGA | Cleavage |
| ccp-miR6459a-4-3p | Cc02_g35780 | 2.5 | 22.269 | 1 | 21 | 1934 | 1954 | UCGAGUUUGAGCUUGAGCUUU | GAAGCUCGUGCUCAAACUCGC | Cleavage |
| ccp-miR6459a-4-3p | Cc02_g36750 | 3.0 | 19.954 | 1 | 20 | 581 | 600 | UCGAGUUUGAGCUUGAGCUU | GGGCUCGAGAUCAAAUUCGA | Translation |
| ccp-miR6459a-4-3p | Cc04_g14410 | 3.0 | 18.562 | 1 | 21 | 1659 | 1679 | UCGAGUUUGAGCUUGAGCUUU | GAAGAUCAGGCGUAAACUCGA | Translation |
| ccp-miR6459a-4-3p | Cc05_g02690 | 2.5 | 22.35 | 1 | 20 | 4410 | 4429 | UCGAGUUUGAGCUUGAGCUU | AAGGUCGAGCUCGAGCUCGA | Cleavage |
| ccp-miR6459a-4-3p | Cc05_g13650 | 3.0 | 15.488 | 1 | 21 | 1985 | 2005 | UCGAGUUUGAGCUUGAGCUUU | AAUGCACAAGCACAAACUCGA | Translation |
| ccp-miR6459a-4-3p | Cc05_g14250 | 3.0 | 15.592 | 1 | 21 | 146 | 166 | UCGAGUUUGAGCUUGAGCUUU | GACGCUCAAGCUCAAGCUCAA | Cleavage |
| ccp-miR6459a-4-3p | Cc06_g14150 | 2.5 | 20.553 | 1 | 20 | 225 | 244 | UCGAGUUUGAGCUUGAGCUU | GAGUUCAAGUUCAAGUUCGA | Cleavage |
| ccp-miR6459a-4-3p | Cc07_g19690 | 3.0 | 9.138 | 1 | 20 | 590 | 609 | UCGAGUUUGAGCUUGAGCUU | UAGCUCAAGCUCAAAUUCAA | Cleavage |
| ccp-miR6459a-4-3p | Cc08_g03940 | 3.0 | 24.59 | 1 | 20 | 2413 | 2432 | UCGAGUUUGAGCUUGAGCUU | UGGCUCGAGUUCGAACUCGA | Cleavage |
| ccp-miR6459a-4-3p | Cc08_g11020 | 3.0 | 9.217 | 1 | 20 | 227 | 246 | UCGAGUUUGAGCUUGAGCUU | GAUCUCAAGCUCAAACCCGA | Cleavage |
| ccp-miR6459a-4-3p | Cc08_g12490 | 2.5 | 22.319 | 1 | 21 | 144 | 164 | UCGAGUUUGAGCUUGAGCUUU | GAUGUUCCAGCUCAAACUCGA | Cleavage |
| ccp-miR6459a-4-3p | Cc09_g00590 | 3.0 | 21.618 | 1 | 20 | 723 | 742 | UCGAGUUUGAGCUUGAGCUU | GAGCUUAAGCUCAUGCUCGA | Cleavage |
| ccp-miR6459a-4-3p | Cc11_g09480 | 0.0 | 20.649 | 1 | 21 | 1477 | 1497 | UCGAGUUUGAGCUUGAGCUUU | AAAGCUCAAGCUCAAACUCGA | Cleavage |
| ccp-miR6459a-4-3p | Cc11_g09510 | 3.0 | 24.735 | 1 | 20 | 433 | 452 | UCGAGUUUGAGCUUGAGCUU | CAGCUAAAGCUCGAGCUCGA | Cleavage |
| ccp-miR6462c-5p | Cc01_g04130 | 3.0 | 11.037 | 1 | 20 | 1416 | 1435 | AAGGGACAAAAAUGGAGGAA | GUCCUCCAUUUUUGUCCUAU | Cleavage |
| ccp-miR6462c-5p | Cc01_g05310 | 3.0 | 13.778 | 1 | 21 | 1187 | 1207 | AAGGGACAAAAAUGGAGGAAG | UUUCCUCCACUUUGGUUUCUU | Cleavage |
| ccp-miR6462c-5p | Cc01_g09780 | 3.0 | 4.684 | 1 | 21 | 163 | 183 | AAGGGACAAAAAUGGAGGAAG | UUUGUUUCAUUUUAGUCCCUU | Cleavage |
| ccp-miR6462c-5p | Cc01_g13250 | 3.0 | 10.574 | 1 | 22 | 1190 | 1211 | AAGGGACAAAAAUGGAGGAAGA | UAUUUAUUCAUUUUUGUUUCUU | Cleavage |
| ccp-miR6462c-5p | Cc01_g14330 | 2.5 | 20.187 | 1 | 20 | 309 | 328 | AAGGGACAAAAAUGGAGGAA | UUCAUCUAUUCUUGUCCCUU | Translation |
| ccp-miR6462c-5p | Cc01_g16680 | 3.0 | 5.03 | 1 | 21 | 111 | 131 | AAGGGACAAAAAUGGAGGAAG | CUUCCUCCUCUUUUGUUCUUU | Cleavage |
| ccp-miR6462c-5p | Cc01_g20830 | 3.0 | 15.774 | 1 | 22 | 1066 | 1087 | AAGGGACAAAAAUGGAGGAAGA | UGUUCUUCCGUUUUUUUUCCUU | Cleavage |
| ccp-miR6462c-5p | Cc02_g05040 | 3.0 | 14.487 | 1 | 21 | 311 | 331 | AAGGGACAAAAAUGGAGGAAG | CUUCCUCCAAAUUUGUCCCUA | Translation |
| ccp-miR6462c-5p | Cc02_g06470 | 3.0 | 8.19 | 1 | 22 | 255 | 276 | AAGGGACAAAAAUGGAGGAAGA | UUUUUUUUUAGUUUUGUCCCUU | Cleavage |
| ccp-miR6462c-5p | Cc02_g12480 | 3.0 | 11.189 | 1 | 22 | 195 | 217 | AAGGGACAAA-AAUGGAGGAAGA | UCUUCCUUUAUUCUUUGUCCCUU | Translation |
| ccp-miR6462c-5p | Cc02_g13730 | 3.0 | 10.285 | 1 | 22 | 153 | 174 | AAGGGACAAAAAUGGAGGAAGA | UCUUUCUUCAUUUUUAUUCCUU | Cleavage |
| ccp-miR6462c-5p | Cc05_g02940 | 3.0 | 2.086 | 1 | 20 | 433 | 452 | AAGGGACAAAAAUGGAGGAA | UUCCUCCCUUUUUUUUCCUU | Cleavage |
| ccp-miR6462c-5p | Cc06_g07160 | 3.0 | 8.392 | 1 | 22 | 278 | 299 | AAGGGACAAAAAUGGAGGAAGA | UUUUUUUUCGUUUUUGUUCUUU | Cleavage |
| ccp-miR6462c-5p | Cc06_g14140 | 3.0 | 10.416 | 1 | 21 | 1533 | 1553 | AAGGGACAAAAAUGGAGGAAG | UUUCCUCUUUUAUUGUCCUUU | Translation |
| ccp-miR6462c-5p | Cc07_g02510 | 3.0 | 5.074 | 1 | 22 | 262 | 283 | AAGGGACAAAAAUGGAGGAAGA | UGUUUUUCCAUUUUUUUUCCUU | Cleavage |
| ccp-miR6462c-5p | Cc07_g15380 | 3.0 | 17.974 | 1 | 22 | 188 | 209 | AAGGGACAAAAAUGGAGGAAGA | UUUUCCUCCAUCUCUGUUUCUU | Translation |
| ccp-miR6462c-5p | Cc08_g14310 | 3.0 | 12.434 | 1 | 21 | 427 | 447 | AAGGGACAAAAAUGGAGGAAG | UUUUUUCUUUUUUUGUCCUUU | Cleavage |
| ccp-miR6462c-5p | Cc08_g15490 | 2.5 | 16.298 | 1 | 21 | 2245 | 2265 | AAGGGACAAAAAUGGAGGAAG | CUUAAUCCAUUUUUGUCUCUU | Cleavage |
| ccp-miR6462c-5p | Cc09_g04810 | 2.5 | 14.516 | 1 | 21 | 20 | 40 | AAGGGACAAAAAUGGAGGAAG | UUGCCUUCGUUUUUGUCUCUU | Cleavage |
| ccp-miR6462c-5p | Cc10_g15230 | 3.0 | 14.678 | 1 | 21 | 342 | 362 | AAGGGACAAAAAUGGAGGAAG | CUUUCUCCAUUUGAGUCCUUU | Translation |
| ccp-miR6476a-3p | Cc01_g15580 | 3.0 | 13.642 | 1 | 20 | 1343 | 1361 | UCAGUGGAGAUGAAACAUUU | AAGUGUU-CAUCUCCAUUGA | Cleavage |
| ccp-miR6476a-3p | Cc02_g10340 | 2.5 | 19.865 | 1 | 20 | 538 | 557 | UCAGUGGAGAUGAAACAUUU | UAAUGUUUCAUCUUUGCUGA | Cleavage |
| ccp-miR6476a-3p | Cc02_g36430 | 3.0 | 14.688 | 1 | 20 | 1052 | 1071 | UCAGUGGAGAUGAAACAUUU | AAAUGCUUCACUUCUACUGA | Translation |
| ccp-miR6476a-3p | Cc05_g05450 | 2.5 | 8.775 | 1 | 20 | 810 | 829 | UCAGUGGAGAUGAAACAUUU | AGCUGUUUCAACUCCACUGA | Translation |
| ccp-miR6476a-3p | Cc06_g22600 | 2.0 | 13.262 | 1 | 20 | 1155 | 1174 | UCAGUGGAGAUGAAACAUUU | AAAUGUUUUUUCUCCAUUGA | Translation |
| ccp-miR6476a-3p | Cc10_g08510 | 3.0 | 13.898 | 1 | 20 | 2035 | 2053 | UCAGUGGAGAUGAAACAUUU | AAAUGGUUC-UCUCCACUGA | Translation |
| ccp-miR6476a-3p | Cc10_g13110 | 2.5 | 18.357 | 1 | 20 | 847 | 866 | UCAGUGGAGAUGAAACAUUU | AAAUGUUUCGUCUCUAUUGC | Cleavage |
| ccp-miR6815-5p | Cc00_g24660 | 3.0 | 15.904 | 1 | 20 | 568 | 587 | UAGUUGGCAUCAGAGGAGUC | GAUUCUUCUGAUGCCAAUGA | Cleavage |
| ccp-miR6815-5p | Cc01_g19890 | 2.0 | 21.426 | 1 | 20 | 612 | 631 | UAGUUGGCAUCAGAGGAGUC | GGCUCCUCUGAGGUCAACUA | Translation |
| ccp-miR6815-5p | Cc08_g01600 | 2.5 | 20.11 | 1 | 22 | 1931 | 1952 | UAGUUGGCAUCAGAGGAGUCAG | UUGAUUCUUCAGAUGCCAAUUA | Cleavage |
| ccp-miR6815-5p | Cc08_g14380 | 3.0 | 20.449 | 1 | 20 | 679 | 698 | UAGUUGGCAUCAGAGGAGUC | GCCUUCUCCGAUGUCAACUA | Cleavage |
| ccp-miR6815-5p | Cc09_g05560 | 3.0 | 11.202 | 1 | 21 | 345 | 365 | UAGUUGGCAUCAGAGGAGUCA | UGACUAUUUUGAUACCAACUA | Cleavage |
| ccp-miR6815-5p | Cc11_g09160 | 3.0 | 21.241 | 1 | 23 | 751 | 773 | UAGUUGGCAUCAGAGGAGUCAGG | UCAGAUUCUUCUGGUGCUGAUUA | Cleavage |
| ccp-miR7122a-3p | Cc03_g04930 | 0.0 | 14.818 | 1 | 21 | 3866 | 3886 | CCGCGUUUCUUUGUAUGAAGA | UCUUCAUACAAAGAAACGCGG | Cleavage |
| ccp-miR7122a-3p | Cc07_g10240 | 3.0 | 17.797 | 1 | 21 | 76 | 96 | CCGCGUUUCUUUGUAUGAAGA | UUUUCGUUCAAAGAAGCGUGG | Cleavage |
| ccp-miR7122a-5p | Cc00_g31710 | 3.0 | 21.819 | 1 | 20 | 591 | 610 | UUAUACAGAGAAACCGCGGU | GGCGCGGUUUCUGUGUAUAA | Cleavage |
| ccp-miR7122a-5p | Cc01_g07870 | 3.0 | 21.819 | 1 | 20 | 591 | 610 | UUAUACAGAGAAACCGCGGU | GGCGCGGUUUCUGUGUAUAA | Cleavage |
| ccp-miR7122a-5p | Cc03_g04930 | 0.0 | 21.679 | 1 | 22 | 3961 | 3982 | UUAUACAGAGAAACCGCGGUUG | CAACCGCGGUUUCUCUGUAUAA | Cleavage |
| ccp-miR7494b-3p | Cc00_g01310 | 3.0 | 11.777 | 1 | 23 | 25 | 47 | AGAGGGAGAGGAAGAAGAGAAAA | UUUUCCCCUUUUCCUCUCCCUUU | Cleavage |
| ccp-miR7494b-3p | Cc00_g05500 | 2.0 | 2.105 | 1 | 23 | 358 | 380 | AGAGGGAGAGGAAGAAGAGAAAA | UUUUUUCUUCUUUCUUUCCUUCU | Cleavage |
| ccp-miR7494b-3p | Cc00_g07710 | 3.0 | 15.501 | 1 | 22 | 525 | 546 | AGAGGGAGAGGAAGAAGAGAAA | UUUUUCUUUUUUCUUUCUCUUU | Cleavage |
| ccp-miR7494b-3p | Cc00_g08160 | 2.5 | 1.016 | 1 | 22 | 99 | 120 | AGAGGGAGAGGAAGAAGAGAAA | UUCUUCUUCUUCUUCUCUCUCU | Cleavage |
| ccp-miR7494b-3p | Cc00_g09190 | 2.5 | 18.732 | 1 | 22 | 1075 | 1096 | AGAGGGAGAGGAAGAAGAGAAA | UUUCUCUUCGUUUUCUUCCUCU | Cleavage |
| ccp-miR7494b-3p | Cc00_g09270 | 3.0 | 8.878 | 1 | 23 | 8 | 30 | AGAGGGAGAGGAAGAAGAGAAAA | UUUUCUUUUUUUCUUUUCUUUCU | Cleavage |
| ccp-miR7494b-3p | Cc00_g10270 | 2.0 | 11.563 | 1 | 22 | 34 | 55 | AGAGGGAGAGGAAGAAGAGAAA | UCUCUCUUCUUCCUUUCUCUCC | Cleavage |
| ccp-miR7494b-3p | Cc00_g17560 | 3.0 | 8.656 | 1 | 23 | 385 | 407 | AGAGGGAGAGGAAGAAGAGAAAA | UUUUUUCUUCUUCCUUUUUCUCG | Cleavage |
| ccp-miR7494b-3p | Cc00_g18380 | 3.0 | 17.276 | 1 | 22 | 2120 | 2141 | AGAGGGAGAGGAAGAAGAGAAA | UUUCUCUUCUUUUUUUCUUUUU | Cleavage |
| ccp-miR7494b-3p | Cc00_g21700 | 3.0 | 8.374 | 1 | 22 | 164 | 185 | AGAGGGAGAGGAAGAAGAGAAA | UUGUUCUUCUUCUUCUUCUUCU | Cleavage |
| ccp-miR7494b-3p | Cc00_g24520 | 2.5 | 20.822 | 1 | 23 | 121 | 143 | AGAGGGAGAGGAAGAAGAGAAAA | UUUUCUUUUCUUCUUUUCUCUUU | Cleavage |
| ccp-miR7494b-3p | Cc00_g27250 | 1.5 | 10.305 | 1 | 22 | 12 | 33 | AGAGGGAGAGGAAGAAGAGAAA | UCUCUCUUCUUCUUCUUCCUUU | Cleavage |
| ccp-miR7494b-3p | Cc00_g28390 | 2.5 | 19.559 | 1 | 22 | 34 | 55 | AGAGGGAGAGGAAGAAGAGAAA | UUUCUCUUCUUUUCCUUCCUCU | Translation |
| ccp-miR7494b-3p | Cc00_g29330 | 3.0 | 1.404 | 1 | 22 | 892 | 913 | AGAGGGAGAGGAAGAAGAGAAA | UUUCUUUUCUUUCUUUUCUUUU | Cleavage |
| ccp-miR7494b-3p | Cc00_g31940 | 2.0 | 3.645 | 1 | 22 | 1441 | 1462 | AGAGGGAGAGGAAGAAGAGAAA | UUUUUCUUCUUCUUCUUCUUCU | Cleavage |
| ccp-miR7494b-3p | Cc00_g33510 | 2.5 | 6.551 | 1 | 22 | 479 | 500 | AGAGGGAGAGGAAGAAGAGAAA | UUCCUCUUCUUCUUCUUCUUCU | Cleavage |
| ccp-miR7494b-3p | Cc00_g36030 | 2.0 | 14.772 | 1 | 23 | 575 | 597 | AGAGGGAGAGGAAGAAGAGAAAA | UUGUUUCUUCUUCUUCUUCUUCU | Cleavage |
| ccp-miR7494b-3p | Cc01_g03840 | 2.5 | 0.124 | 1 | 23 | 15 | 37 | AGAGGGAGAGGAAGAAGAGAAAA | UUCUUUCUUUUUUCUUUCUCUCU | Cleavage |
| ccp-miR7494b-3p | Cc01_g16760 | 1.5 | 14.426 | 1 | 20 | 1584 | 1603 | AGAGGGAGAGGAAGAAGAGA | UCUCUUCUUCUUUUCCCUUU | Cleavage |
| ccp-miR7494b-3p | Cc01_g17940 | 2.0 | 4.087 | 1 | 21 | 24 | 44 | AGAGGGAGAGGAAGAAGAGAA | UUUUCUUCUUCUUCUUCUUCU | Cleavage |
| ccp-miR7494b-3p | Cc02_g00370 | 2.0 | 12.703 | 1 | 22 | 926 | 947 | AGAGGGAGAGGAAGAAGAGAAA | UCUCUCUUUUUUCUCUCUCUUU | Cleavage |
| ccp-miR7494b-3p | Cc02_g00890 | 3.0 | 3.797 | 1 | 23 | 2 | 24 | AGAGGGAGAGGAAGAAGAGAAAA | UUUCUUCUUCUUCUUCUUCUUCU | Cleavage |
| ccp-miR7494b-3p | Cc02_g03270 | 2.5 | 24.155 | 1 | 23 | 620 | 642 | AGAGGGAGAGGAAGAAGAGAAAA | UUCUUUCUUCUUCUUCUUCUUUU | Cleavage |
| ccp-miR7494b-3p | Cc02_g04440 | 3.0 | 3.897 | 1 | 23 | 354 | 376 | AGAGGGAGAGGAAGAAGAGAAAA | UUUCUUCUUCUUCUUCUUCUUCU | Cleavage |
| ccp-miR7494b-3p | Cc02_g10550 | 3.0 | 3.07 | 1 | 23 | 35 | 57 | AGAGGGAGAGGAAGAAGAGAAAA | UUUUCUCGUCUUCUCCUCUCUCU | Translation |
| ccp-miR7494b-3p | Cc02_g11290 | 2.0 | 10.865 | 1 | 21 | 281 | 301 | AGAGGGAGAGGAAGAAGAGAA | UUUUCUUCUUCUUCUUCUUCU | Cleavage |
| ccp-miR7494b-3p | Cc02_g11430 | 2.0 | 4.817 | 1 | 21 | 279 | 299 | AGAGGGAGAGGAAGAAGAGAA | UACUCUUCUUCUUCUUCCUCU | Cleavage |
| ccp-miR7494b-3p | Cc02_g13510 | 2.5 | 2.238 | 1 | 22 | 237 | 258 | AGAGGGAGAGGAAGAAGAGAAA | UUUCUUUUCUUUCUUUCUUUCU | Cleavage |
| ccp-miR7494b-3p | Cc02_g13710 | 3.0 | 2.602 | 1 | 23 | 181 | 203 | AGAGGGAGAGGAAGAAGAGAAAA | UUUCUUCUUCUUCUUCUUCUUCU | Cleavage |
| ccp-miR7494b-3p | Cc02_g14710 | 2.5 | 2.18 | 1 | 23 | 149 | 171 | AGAGGGAGAGGAAGAAGAGAAAA | UUUUCUCUUCUUCUUCUUCAUCU | Cleavage |
| ccp-miR7494b-3p | Cc02_g15030 | 1.5 | 12.927 | 1 | 23 | 482 | 504 | AGAGGGAGAGGAAGAAGAGAAAA | UUCUCUCUUCUUCUUCUUCUUCU | Cleavage |
| ccp-miR7494b-3p | Cc02_g17060 | 2.0 | 6.9 | 1 | 22 | 369 | 390 | AGAGGGAGAGGAAGAAGAGAAA | UUCCUCUUCUUCUUCUCCUUCU | Cleavage |
| ccp-miR7494b-3p | Cc02_g17070 | 3.0 | 7.382 | 1 | 22 | 651 | 672 | AGAGGGAGAGGAAGAAGAGAAA | UUUUUCUUUUUCCUUUUUCUUU | Cleavage |
| ccp-miR7494b-3p | Cc02_g19210 | 2.5 | 14.1 | 1 | 22 | 911 | 932 | AGAGGGAGAGGAAGAAGAGAAA | UUUCGCUUCUUCUUCUUCUUCU | Cleavage |
| ccp-miR7494b-3p | Cc02_g24110 | 1.5 | 4.309 | 1 | 20 | 241 | 260 | AGAGGGAGAGGAAGAAGAGA | UCUCUUCUUCUUCUUCUUCU | Cleavage |
| ccp-miR7494b-3p | Cc02_g24390 | 2.0 | 8.939 | 1 | 23 | 65 | 87 | AGAGGGAGAGGAAGAAGAGAAAA | UUGUUUCUUCUUCUUCUUCUUCU | Cleavage |
| ccp-miR7494b-3p | Cc02_g33330 | 2.0 | 14.538 | 1 | 21 | 509 | 529 | AGAGGGAGAGGAAGAAGAGAA | UCCUCUUCUUCCUCUUCUUCU | Cleavage |
| ccp-miR7494b-3p | Cc02_g35350 | 3.0 | 7.754 | 1 | 23 | 70 | 92 | AGAGGGAGAGGAAGAAGAGAAAA | UUUCUUCUUCUUCUUCUUCUUCU | Cleavage |
| ccp-miR7494b-3p | Cc02_g35880 | 2.5 | 18.006 | 1 | 22 | 33 | 54 | AGAGGGAGAGGAAGAAGAGAAA | UUCCUCUUCUUCCUUUUCCUUU | Cleavage |
| ccp-miR7494b-3p | Cc02_g36820 | 2.0 | 11.06 | 1 | 23 | 2004 | 2026 | AGAGGGAGAGGAAGAAGAGAAAA | UAUUUUUUUUUUCCUCUCUCUCU | Cleavage |
| ccp-miR7494b-3p | Cc02_g37160 | 2.5 | 13.696 | 1 | 23 | 1860 | 1882 | AGAGGGAGAGGAAGAAGAGAAAA | UUUUUUUUUUUUUUUCUCCCUCU | Cleavage |
| ccp-miR7494b-3p | Cc02_g38810 | 1.5 | 2.816 | 1 | 20 | 52 | 71 | AGAGGGAGAGGAAGAAGAGA | UCUCUUCUUCCUCUUCUUUU | Cleavage |
| ccp-miR7494b-3p | Cc02_g39450 | 2.0 | 9.096 | 1 | 23 | 1548 | 1570 | AGAGGGAGAGGAAGAAGAGAAAA | UAUUUUCUUCUUCUUCUUCUUCU | Cleavage |
| ccp-miR7494b-3p | Cc02_g39820 | 3.0 | 16.081 | 1 | 22 | 909 | 930 | AGAGGGAGAGGAAGAAGAGAAA | UUCUUCUUCUUCUUCUUCUUCU | Cleavage |
| ccp-miR7494b-3p | Cc03_g03310 | 2.5 | 12.429 | 1 | 22 | 107 | 128 | AGAGGGAGAGGAAGAAGAGAAA | UUUCUAUUCUUCUUCUUCUUCU | Cleavage |
| ccp-miR7494b-3p | Cc03_g03480 | 2.5 | 3.411 | 1 | 21 | 199 | 219 | AGAGGGAGAGGAAGAAGAGAA | UUCUCUUCUUCUUCCUCCUCU | Cleavage |
| ccp-miR7494b-3p | Cc03_g14840 | 2.5 | 5.165 | 1 | 22 | 294 | 315 | AGAGGGAGAGGAAGAAGAGAAA | UUUGUCUUCUUCUUCUUCUUCU | Cleavage |
| ccp-miR7494b-3p | Cc03_g15700 | 2.5 | 19.743 | 1 | 22 | 22 | 43 | AGAGGGAGAGGAAGAAGAGAAA | UUUCUCUUCAUUUUCUUCCUCU | Cleavage |
| ccp-miR7494b-3p | Cc03_g15830 | 2.5 | 17.354 | 1 | 22 | 34 | 55 | AGAGGGAGAGGAAGAAGAGAAA | UUUCUCUUCUUUUCCUUCCUCU | Translation |
| ccp-miR7494b-3p | Cc04_g01060 | 3.0 | 0.736 | 1 | 23 | 33 | 55 | AGAGGGAGAGGAAGAAGAGAAAA | UUUUCUUUUUUUCCUUUCUUUUU | Cleavage |
| ccp-miR7494b-3p | Cc04_g06050 | 2.0 | 3.885 | 1 | 22 | 116 | 137 | AGAGGGAGAGGAAGAAGAGAAA | UUUCUCUUCUCUCUCUCUCUCU | Cleavage |
| ccp-miR7494b-3p | Cc04_g07010 | 2.5 | 4.831 | 1 | 23 | 2739 | 2761 | AGAGGGAGAGGAAGAAGAGAAAA | UUGUUUUUUUUUCCUCUCUCUUU | Cleavage |
| ccp-miR7494b-3p | Cc04_g07570 | 3.0 | 17.31 | 1 | 23 | 568 | 590 | AGAGGGAGAGGAAGAAGAGAAAA | UUUUUUUUUUUUCUUCUUCUUCU | Cleavage |
| ccp-miR7494b-3p | Cc04_g08880 | 1.5 | 17.458 | 1 | 23 | 19 | 41 | AGAGGGAGAGGAAGAAGAGAAAA | UCUUUUCUUCUUCUUCUCUCUCU | Cleavage |
| ccp-miR7494b-3p | Cc04_g10100 | 3.0 | 1.479 | 1 | 22 | 48 | 69 | AGAGGGAGAGGAAGAAGAGAAA | UUUUUUUUCUUUUUCUCUUUCU | Cleavage |
| ccp-miR7494b-3p | Cc04_g10510 | 1.5 | 16.689 | 1 | 23 | 175 | 197 | AGAGGGAGAGGAAGAAGAGAAAA | UCUUCUCUUCUUCUUCUUCUUCU | Cleavage |
| ccp-miR7494b-3p | Cc04_g13240 | 2.0 | 4.452 | 1 | 23 | 83 | 105 | AGAGGGAGAGGAAGAAGAGAAAA | UUCUCUCUUCUUCUUUUUCUUCU | Cleavage |
| ccp-miR7494b-3p | Cc05_g01520 | 3.0 | 2.589 | 1 | 23 | 894 | 916 | AGAGGGAGAGGAAGAAGAGAAAA | UUUUUUUUUCUUCCGCUUUCUCU | Translation |
| ccp-miR7494b-3p | Cc05_g10210 | 2.5 | 16.731 | 1 | 22 | 2089 | 2110 | AGAGGGAGAGGAAGAAGAGAAA | UUUCUUUUCUUUCUUUCUCUUU | Cleavage |
| ccp-miR7494b-3p | Cc05_g10750 | 3.0 | 14.265 | 1 | 23 | 147 | 169 | AGAGGGAGAGGAAGAAGAGAAAA | UUUCUUCUUCUUCUUCUUCUUCU | Cleavage |
| ccp-miR7494b-3p | Cc05_g11770 | 3.0 | 13.331 | 1 | 22 | 379 | 400 | AGAGGGAGAGGAAGAAGAGAAA | UUUUUCUUUUUCUUUUUCCUUU | Cleavage |
| ccp-miR7494b-3p | Cc05_g14690 | 2.5 | 13.756 | 1 | 21 | 644 | 664 | AGAGGGAGAGGAAGAAGAGAA | UUUUCUUCUUCUUUUCCUUUU | Cleavage |
| ccp-miR7494b-3p | Cc05_g15070 | 2.5 | 5.936 | 1 | 23 | 128 | 150 | AGAGGGAGAGGAAGAAGAGAAAA | UGUUUUUUUCUUUCUCUCUUUCU | Cleavage |
| ccp-miR7494b-3p | Cc05_g15090 | 3.0 | 12.043 | 1 | 23 | 91 | 113 | AGAGGGAGAGGAAGAAGAGAAAA | UUUCUUCUUCUUCUUCUUCUUCU | Cleavage |
| ccp-miR7494b-3p | Cc06_g00380 | 3.0 | 2.286 | 1 | 23 | 302 | 324 | AGAGGGAGAGGAAGAAGAGAAAA | UUUUUUUUUUUUCUUCUCUCUUU | Cleavage |
| ccp-miR7494b-3p | Cc06_g09860 | 2.5 | 4.773 | 1 | 22 | 161 | 182 | AGAGGGAGAGGAAGAAGAGAAA | UUCCUCUUCUUCUUCUUCUUCU | Cleavage |
| ccp-miR7494b-3p | Cc06_g16190 | 3.0 | 4.717 | 1 | 23 | 2255 | 2277 | AGAGGGAGAGGAAGAAGAGAAAA | UUUCUUCUUCUUCUUCUUCUUCU | Cleavage |
| ccp-miR7494b-3p | Cc06_g19160 | 3.0 | 0.38 | 1 | 23 | 69 | 91 | AGAGGGAGAGGAAGAAGAGAAAA | UUUUCUCCUCCUCCUCUUCUUCU | Cleavage |
| ccp-miR7494b-3p | Cc06_g19990 | 2.0 | 1.284 | 1 | 22 | 130 | 151 | AGAGGGAGAGGAAGAAGAGAAA | UUUCUUUUCUUCUUCUUCUUCU | Cleavage |
| ccp-miR7494b-3p | Cc06_g23020 | 2.5 | 2.908 | 1 | 22 | 249 | 270 | AGAGGGAGAGGAAGAAGAGAAA | UUCCUCUUCUUCUUCUUCUUCU | Cleavage |
| ccp-miR7494b-3p | Cc07_g00150 | 2.0 | 1.671 | 1 | 23 | 97 | 119 | AGAGGGAGAGGAAGAAGAGAAAA | UUCUCUCUCCUUUCUUUCCCUCU | Cleavage |
| ccp-miR7494b-3p | Cc07_g02860 | 2.0 | 9.275 | 1 | 22 | 386 | 407 | AGAGGGAGAGGAAGAAGAGAAA | UUUCUCUUUUUCUUCUCUUUCU | Cleavage |
| ccp-miR7494b-3p | Cc07_g03910 | 1.0 | 21.298 | 1 | 22 | 3 | 24 | AGAGGGAGAGGAAGAAGAGAAA | UUUUUCUUCUUCUUCUCCCUCU | Cleavage |
| ccp-miR7494b-3p | Cc07_g05270 | 2.5 | 8.237 | 1 | 23 | 604 | 626 | AGAGGGAGAGGAAGAAGAGAAAA | UUUUCUUUUCUUUCUUUCUUUCU | Cleavage |
| ccp-miR7494b-3p | Cc07_g10170 | 2.5 | 9.452 | 1 | 21 | 97 | 117 | AGAGGGAGAGGAAGAAGAGAA | UUCUUUUCUUUCUUUCUUUCU | Cleavage |
| ccp-miR7494b-3p | Cc07_g10560 | 3.0 | 9.203 | 1 | 23 | 2705 | 2728 | AGAGGGAGAGGAAGAAG-AGAAAA | UUUUCUACUUUUUCCUCUCUCUCU | Cleavage |
| ccp-miR7494b-3p | Cc07_g14620 | 2.0 | 15.477 | 1 | 23 | 517 | 539 | AGAGGGAGAGGAAGAAGAGAAAA | UUGUUUCUUCUUCUUCUUCUUCU | Cleavage |
| ccp-miR7494b-3p | Cc08_g00440 | 1.5 | 12.967 | 1 | 20 | 233 | 252 | AGAGGGAGAGGAAGAAGAGA | UCUCUUCUUCUUCUUCUUCU | Cleavage |
| ccp-miR7494b-3p | Cc08_g00490 | 2.5 | 12.207 | 1 | 22 | 1214 | 1235 | AGAGGGAGAGGAAGAAGAGAAA | UUCCUCUUCUUCUUCUUCUUCU | Cleavage |
| ccp-miR7494b-3p | Cc08_g01680 | 1.0 | 4.073 | 1 | 23 | 57 | 79 | AGAGGGAGAGGAAGAAGAGAAAA | UGUUCUCUUCUUUCUCUCCUUCU | Cleavage |
| ccp-miR7494b-3p | Cc08_g02070 | 3.0 | 2.365 | 1 | 22 | 45 | 66 | AGAGGGAGAGGAAGAAGAGAAA | UUCCUCUUCUUCUUCUCCUUCA | Cleavage |
| ccp-miR7494b-3p | Cc08_g05990 | 2.5 | 9.977 | 1 | 22 | 365 | 386 | AGAGGGAGAGGAAGAAGAGAAA | UUCUUCUUCUUCCUCUUCUUCU | Cleavage |
| ccp-miR7494b-3p | Cc08_g07700 | 1.5 | 8.845 | 1 | 20 | 47 | 66 | AGAGGGAGAGGAAGAAGAGA | UCUUUUCUUCCUCUUCUUCU | Cleavage |
| ccp-miR7494b-3p | Cc08_g11260 | 1.5 | 6.031 | 1 | 22 | 12 | 33 | AGAGGGAGAGGAAGAAGAGAAA | UCUCUCUUCUUCUUCUUCCUUU | Cleavage |
| ccp-miR7494b-3p | Cc08_g11960 | 3.0 | 9.676 | 1 | 22 | 312 | 333 | AGAGGGAGAGGAAGAAGAGAAA | UUUUUCUUUUUCCUUUUUCUUU | Cleavage |
| ccp-miR7494b-3p | Cc08_g13190 | 3.0 | 11.235 | 1 | 23 | 2015 | 2037 | AGAGGGAGAGGAAGAAGAGAAAA | UUUUUUUUUCUUUCUUUCUUUCU | Cleavage |
| ccp-miR7494b-3p | Cc08_g13270 | 1.5 | 4.896 | 1 | 23 | 2008 | 2030 | AGAGGGAGAGGAAGAAGAGAAAA | UUUUCUCUUCUUUCUUUCUCUCU | Cleavage |
| ccp-miR7494b-3p | Cc08_g15690 | 3.0 | 8.343 | 1 | 23 | 1522 | 1544 | AGAGGGAGAGGAAGAAGAGAAAA | UUUUUUUUUGUUCCUCUUUCUCU | Cleavage |
| ccp-miR7494b-3p | Cc09_g00120 | 3.0 | 10.466 | 1 | 22 | 632 | 653 | AGAGGGAGAGGAAGAAGAGAAA | UUCUUCUUCUUCUUCUUCUUCU | Cleavage |
| ccp-miR7494b-3p | Cc09_g00250 | 2.5 | 15.533 | 1 | 21 | 96 | 116 | AGAGGGAGAGGAAGAAGAGAA | UUCUCUUCUUUUUCUUUCUUU | Cleavage |
| ccp-miR7494b-3p | Cc09_g00550 | 3.0 | 11.272 | 1 | 23 | 506 | 528 | AGAGGGAGAGGAAGAAGAGAAAA | UUUUCCCUUUUUCCUUUUCCUUU | Cleavage |
| ccp-miR7494b-3p | Cc09_g03270 | 3.0 | 11.634 | 1 | 23 | 538 | 560 | AGAGGGAGAGGAAGAAGAGAAAA | UUUUCUAUUUUUCCUUUUCCUUU | Cleavage |
| ccp-miR7494b-3p | Cc09_g04020 | 2.5 | 2.144 | 1 | 22 | 48 | 69 | AGAGGGAGAGGAAGAAGAGAAA | UUCUUCUUCUUCUUCUUCCUCU | Cleavage |
| ccp-miR7494b-3p | Cc09_g10000 | 3.0 | 24.416 | 1 | 23 | 646 | 668 | AGAGGGAGAGGAAGAAGAGAAAA | UUUUCUCAUCUUUCUUUUUCUCU | Cleavage |
| ccp-miR7494b-3p | Cc09_g10330 | 2.5 | 2.831 | 1 | 22 | 344 | 365 | AGAGGGAGAGGAAGAAGAGAAA | UUUCUUUUUUUUUUCUCCCUUU | Cleavage |
| ccp-miR7494b-3p | Cc09_g10760 | 2.0 | 8.73 | 1 | 21 | 6 | 26 | AGAGGGAGAGGAAGAAGAGAA | UCUUCUUCUUCCUCUUCCUCU | Cleavage |
| ccp-miR7494b-3p | Cc10_g03260 | 3.0 | 1.26 | 1 | 23 | 459 | 481 | AGAGGGAGAGGAAGAAGAGAAAA | UUUUUUCUUCUUUUUUUCCUUUU | Cleavage |
| ccp-miR7494b-3p | Cc10_g04440 | 3.0 | 3.209 | 1 | 23 | 1930 | 1952 | AGAGGGAGAGGAAGAAGAGAAAA | UUUCUUCUUCUUCUUCUUCUUCU | Cleavage |
| ccp-miR7494b-3p | Cc10_g08820 | 2.5 | 6.984 | 1 | 23 | 883 | 905 | AGAGGGAGAGGAAGAAGAGAAAA | UUCUCUUUUCUUCCUUUCUUUUU | Cleavage |
| ccp-miR7494b-3p | Cc10_g16360 | 2.5 | 17.808 | 1 | 21 | 47 | 67 | AGAGGGAGAGGAAGAAGAGAA | UUCUCUUCUUCUUCUUUCUCC | Cleavage |
| ccp-miR7494b-3p | Cc11_g08890 | 2.0 | 8.405 | 1 | 22 | 878 | 899 | AGAGGGAGAGGAAGAAGAGAAA | UUUGUUUUCUUCCUCUCUCUCU | Cleavage |
| ccp-miR7494b-3p | Cc11_g10410 | 2.5 | 2.931 | 1 | 22 | 108 | 129 | AGAGGGAGAGGAAGAAGAGAAA | UUUCUCUUCUCCUUCUUCUUCU | Cleavage |
| ccp-miR7494b-3p | Cc11_g15330 | 2.0 | 1.273 | 1 | 22 | 11 | 32 | AGAGGGAGAGGAAGAAGAGAAA | UUUCUUUUCUUCUUCUUCUUCU | Cleavage |
| ccp-miR7494b-3p | Cc11_g17000 | 2.5 | 6.859 | 1 | 22 | 298 | 319 | AGAGGGAGAGGAAGAAGAGAAA | UUUUUCUUCUCUCUCUCUCUCU | Cleavage |
| ccp-miR7494b-3p | Cc11_g17070 | 2.5 | 11.134 | 1 | 23 | 463 | 485 | AGAGGGAGAGGAAGAAGAGAAAA | UUUUCUUUUCUUCCUCUUUUUUU | Cleavage |
| ccp-miR7494b-3p | Cc11_g17340 | 3.0 | 7.574 | 1 | 23 | 121 | 143 | AGAGGGAGAGGAAGAAGAGAAAA | UUUCUUCUUCUUCUUCUUCUUCU | Cleavage |
| ccp-miR7502f-3p | Cc00_g06620 | 3.0 | 14.877 | 1 | 21 | 759 | 780 | UUUAGCAG-UAGAGAUGGAUGG | UCAUCCAUUUCUAUCUGUUAAA | Translation |
| ccp-miR7502f-3p | Cc01_g18550 | 2.5 | 9.932 | 1 | 21 | 331 | 351 | UUUAGCAGUAGAGAUGGAUGG | UCCUCCGCCUCUACUGCUAAA | Cleavage |
| ccp-miR7502f-3p | Cc02_g11250 | 3.0 | 11.05 | 1 | 21 | 378 | 398 | UUUAGCAGUAGAGAUGGAUGG | UCCUUUAUUUUUACUGCUAAA | Cleavage |
| ccp-miR7502f-3p | Cc02_g25800 | 3.0 | 16.428 | 1 | 21 | 206 | 226 | UUUAGCAGUAGAGAUGGAUGG | UCUUCCAUUUCUGCUGUUAAG | Cleavage |
| ccp-miR7502f-3p | Cc02_g28150 | 3.0 | 11.307 | 1 | 21 | 51 | 71 | UUUAGCAGUAGAGAUGGAUGG | CCAUCCAUCUCCGUUGCUAGG | Translation |
| ccp-miR7502f-3p | Cc03_g03250 | 3.0 | 9.495 | 1 | 20 | 331 | 350 | UUUAGCAGUAGAGAUGGAUG | CUUUCAUCUUUACUGCUAAU | Cleavage |
| ccp-miR7502f-3p | Cc06_g21000 | 2.5 | 19.209 | 1 | 21 | 2382 | 2402 | UUUAGCAGUAGAGAUGGAUGG | UCAUCCCUCUCUACUGCUGAC | Cleavage |
| ccp-miR7502f-3p | Cc11_g04820 | 2.0 | 14.46 | 1 | 21 | 1881 | 1901 | UUUAGCAGUAGAGAUGGAUGG | UCAUGCAUCUUUGCUGCUAAA | Cleavage |
| ccp-miR7504a-3p | Cc00_g02080 | 2.5 | 14.852 | 1 | 20 | 698 | 717 | UUUGUUUUUGUCCAAAAUUU | AAACUUUGGACACAAACGAA | Cleavage |
| ccp-miR7504a-3p | Cc00_g02110 | 2.5 | 15.445 | 1 | 20 | 362 | 381 | UUUGUUUUUGUCCAAAAUUU | AAACUUUGGAUAAAAAUGAA | Cleavage |
| ccp-miR7504a-3p | Cc00_g02120 | 2.0 | 22.435 | 1 | 20 | 1070 | 1089 | UUUGUUUUUGUCCAAAAUUU | AAACUUUGGACAAAGACGAA | Cleavage |
| ccp-miR7504a-3p | Cc00_g05700 | 0.5 | 20.69 | 1 | 22 | 146 | 167 | UUUGUUUUUGUCCAAAAUUUCA | UCAAAUUUUGGACGAAAACAAA | Cleavage |
| ccp-miR7504a-3p | Cc00_g09490 | 2.5 | 14.965 | 1 | 20 | 866 | 885 | UUUGUUUUUGUCCAAAAUUU | AAACUUUGGAUAAAAAUGAA | Cleavage |
| ccp-miR7504a-3p | Cc00_g18150 | 3.0 | 14.06 | 1 | 22 | 134 | 155 | UUUGUUUUUGUCCAAAAUUUCA | UAGAAUUGUUGACAAGAACAAA | Cleavage |
| ccp-miR7504a-3p | Cc00_g32700 | 3.0 | 19.795 | 1 | 22 | 134 | 155 | UUUGUUUUUGUCCAAAAUUUCA | UAGAAUCUUUGACAAGAACAAA | Cleavage |
| ccp-miR7504a-3p | Cc01_g02350 | 2.5 | 17.293 | 1 | 22 | 434 | 455 | UUUGUUUUUGUCCAAAAUUUCA | UAGAAUUGUUGACAAAAACAAA | Cleavage |
| ccp-miR7504a-3p | Cc01_g08590 | 3.0 | 10.837 | 1 | 22 | 1715 | 1736 | UUUGUUUUUGUCCAAAAUUUCA | UUAAAUUUUCUGCAAAAACAGA | Cleavage |
| ccp-miR7504a-3p | Cc01_g12300 | 2.5 | 6.863 | 1 | 22 | 1766 | 1787 | UUUGUUUUUGUCCAAAAUUUCA | UAAAAUUUUGGAAAAAAAAAAA | Translation |
| ccp-miR7504a-3p | Cc01_g18570 | 2.0 | 20.143 | 1 | 20 | 3022 | 3041 | UUUGUUUUUGUCCAAAAUUU | AAUUUUUGGAUAAGAACAAA | Cleavage |
| ccp-miR7504a-3p | Cc01_g18740 | 0.5 | 9.775 | 1 | 20 | 545 | 564 | UUUGUUUUUGUCCAAAAUUU | AGAUUUUGGACAAAAACAAA | Cleavage |
| ccp-miR7504a-3p | Cc02_g09120 | 1.5 | 14.423 | 1 | 22 | 2796 | 2817 | UUUGUUUUUGUCCAAAAUUUCA | UCAAUUUUUGGACAAAAAUAAA | Cleavage |
| ccp-miR7504a-3p | Cc02_g09130 | 3.0 | 17.956 | 1 | 20 | 223 | 242 | UUUGUUUUUGUCCAAAAUUU | AGUUUUUGGAUGAGAACAAA | Cleavage |
| ccp-miR7504a-3p | Cc02_g19740 | 3.0 | 7.442 | 1 | 21 | 159 | 179 | UUUGUUUUUGUCCAAAAUUUC | GAAAUUUUGGUUCAAAAUAAA | Translation |
| ccp-miR7504a-3p | Cc02_g31290 | 3.0 | 18.786 | 1 | 21 | 747 | 767 | UUUGUUUUUGUCCAAAAUUUC | GAGAUUUUGGAGAAAGGUAAA | Translation |
| ccp-miR7504a-3p | Cc04_g01550 | 2.5 | 19.127 | 1 | 21 | 2063 | 2083 | UUUGUUUUUGUCCAAAAUUUC | GGAAUUCUGGAUAAAAGCAAA | Cleavage |
| ccp-miR7504a-3p | Cc04_g03120 | 3.0 | 14.341 | 1 | 20 | 1237 | 1256 | UUUGUUUUUGUCCAAAAUUU | AAUUUUUGGACAAUAACAAG | Cleavage |
| ccp-miR7504a-3p | Cc04_g06580 | 2.5 | 16.766 | 1 | 20 | 1035 | 1054 | UUUGUUUUUGUCCAAAAUUU | AGAUUUUGGACACAGACAGA | Cleavage |
| ccp-miR7504a-3p | Cc04_g07220 | 3.0 | 11.037 | 1 | 20 | 406 | 425 | UUUGUUUUUGUCCAAAAUUU | UCAUUUGGGACAAAAACAAA | Cleavage |
| ccp-miR7504a-3p | Cc04_g07590 | 3.0 | 15.07 | 1 | 22 | 1762 | 1783 | UUUGUUUUUGUCCAAAAUUUCA | UGAAAUUUUGGAGGGAAACAAU | Translation |
| ccp-miR7504a-3p | Cc05_g00250 | 3.0 | 21.264 | 1 | 20 | 1563 | 1582 | UUUGUUUUUGUCCAAAAUUU | GAAUUUUGGAGAAAAUCAAA | Translation |
| ccp-miR7504a-3p | Cc06_g14330 | 3.0 | 16.926 | 1 | 20 | 822 | 841 | UUUGUUUUUGUCCAAAAUUU | UGAUUUUGGACGAGGACAAA | Cleavage |
| ccp-miR7504a-3p | Cc06_g16020 | 3.0 | 14.606 | 1 | 21 | 303 | 323 | UUUGUUUUUGUCCAAAAUUUC | GAAAUUUUGGUUGAAAAUAGA | Translation |
| ccp-miR7504a-3p | Cc07_g03340 | 3.0 | 19.625 | 1 | 20 | 1043 | 1062 | UUUGUUUUUGUCCAAAAUUU | AGAUUUUGGACAAGAAUACA | Cleavage |
| ccp-miR7504a-3p | Cc07_g04390 | 2.5 | 21.631 | 1 | 21 | 1675 | 1695 | UUUGUUUUUGUCCAAAAUUUC | GGAGUUUUGGACAGAGAUAAA | Cleavage |
| ccp-miR7504a-3p | Cc07_g09000 | 3.0 | 17.652 | 1 | 22 | 864 | 885 | UUUGUUUUUGUCCAAAAUUUCA | UCAGAUUUUGAACAAAGACAGG | Cleavage |
| ccp-miR7504a-3p | Cc07_g18220 | 2.0 | 23.087 | 1 | 20 | 420 | 439 | UUUGUUUUUGUCCAAAAUUU | AAGUUUUGGACGAAGGCAAA | Cleavage |
| ccp-miR7504a-3p | Cc08_g10070 | 2.5 | 12.02 | 1 | 22 | 1367 | 1388 | UUUGUUUUUGUCCAAAAUUUCA | UUAAAUUUUGGAGAAAAAAAAA | Translation |
| ccp-miR7504a-3p | Cc08_g12180 | 3.0 | 11.147 | 1 | 21 | 2569 | 2589 | UUUGUUUUUGUCCAAAAUUUC | GAAAUUUUGGACAAAAAUAUC | Cleavage |
| ccp-miR7504a-3p | Cc08_g14240 | 2.5 | 15.124 | 1 | 20 | 808 | 827 | UUUGUUUUUGUCCAAAAUUU | AAAUUUUGUCCAGAAACAAA | Translation |
| ccp-miR7504a-3p | Cc10_g08320 | 3.0 | 21.367 | 1 | 20 | 1218 | 1237 | UUUGUUUUUGUCCAAAAUUU | CAAUUUUGGACGAAGGCAGA | Cleavage |
| ccp-miR7504a-3p | Cc11_g05970 | 2.5 | 18.084 | 1 | 20 | 1488 | 1507 | UUUGUUUUUGUCCAAAAUUU | AAGUUGUGGACAUAAACAAA | Cleavage |
| ccp-miR7504a-3p | Cc11_g09850 | 3.0 | 14.311 | 1 | 20 | 150 | 169 | UUUGUUUUUGUCCAAAAUUU | GAAUUUUGGGUAUAAAUAAA | Cleavage |
| ccp-miR7504b-5p | Cc01_g10470 | 2.0 | 10.341 | 1 | 20 | 586 | 605 | AGGAGGAAAAAUGCCAUUAG | CUAAUCGUAUUUUUCUUCCU | Cleavage |
| ccp-miR7504b-5p | Cc01_g15750 | 3.0 | 17.647 | 1 | 20 | 4061 | 4080 | AGGAGGAAAAAUGCCAUUAG | CUGAUGGCAAUUUUUUUCUU | Translation |
| ccp-miR7504b-5p | Cc01_g19290 | 3.0 | 13.359 | 1 | 24 | 2312 | 2335 | AGGAGGAAAAAUGCCAUUAGUCAU | GUUGCUAAUGGUUUUUUUCUUUUU | Cleavage |
| ccp-miR7504b-5p | Cc01_g19550 | 2.5 | 12.417 | 1 | 20 | 1045 | 1064 | AGGAGGAAAAAUGCCAUUAG | CUAGUGGUAUUUUUUUUUCU | Cleavage |
| ccp-miR7504b-5p | Cc02_g05190 | 2.0 | 23.078 | 1 | 24 | 5666 | 5689 | AGGAGGAAAAAUGCCAUUAGUCAU | AAGCUUGAUUGCAUUUUUCCUCCU | Cleavage |
| ccp-miR7504b-5p | Cc02_g13660 | 2.5 | 7.322 | 1 | 20 | 123 | 142 | AGGAGGAAAAAUGCCAUUAG | AUGAUGCCAUUUUUCCUCCU | Cleavage |
| ccp-miR7504b-5p | Cc02_g21620 | 2.0 | 22.966 | 1 | 24 | 1072 | 1095 | AGGAGGAAAAAUGCCAUUAGUCAU | ACGCCUGAUGGCAUAUUUCCUUCU | Translation |
| ccp-miR7504b-5p | Cc02_g28130 | 3.0 | 17.342 | 1 | 20 | 1454 | 1473 | AGGAGGAAAAAUGCCAUUAG | CUGAUGGCUAUUUUUCUCCU | Translation |
| ccp-miR7504b-5p | Cc02_g28280 | 3.0 | 5.708 | 1 | 23 | 336 | 358 | AGGAGGAAAAAUGCCAUUAGUCA | UGUCUAGUUGCAUUUUUCUUUUU | Cleavage |
| ccp-miR7504b-5p | Cc02_g32570 | 2.5 | 15.433 | 1 | 20 | 5995 | 6014 | AGGAGGAAAAAUGCCAUUAG | CUAAUGGUAAUUUUCUUCUU | Translation |
| ccp-miR7504b-5p | Cc05_g14440 | 3.0 | 21.117 | 1 | 21 | 1316 | 1336 | AGGAGGAAAAAUGCCAUUAGU | AAUGGUGUCAUUUUUCCUCCU | Cleavage |
| ccp-miR7504b-5p | Cc06_g12170 | 3.0 | 12.138 | 1 | 20 | 996 | 1015 | AGGAGGAAAAAUGCCAUUAG | CUCGUGGCAUUUGUCUUCCU | Cleavage |
| ccp-miR7504b-5p | Cc07_g07150 | 2.5 | 15.008 | 1 | 21 | 1937 | 1957 | AGGAGGAAAAAUGCCAUUAGU | ACUAAUUGCAGUUUUCCUCUU | Translation |
| ccp-miR7504b-5p | Cc08_g02960 | 3.0 | 15.537 | 1 | 20 | 1295 | 1314 | AGGAGGAAAAAUGCCAUUAG | CUAUUGGCAUCUUUUCUUCU | Translation |
| ccp-miR7504b-5p | Cc08_g10280 | 3.0 | 11.591 | 1 | 20 | 494 | 513 | AGGAGGAAAAAUGCCAUUAG | UUAGUGGCUUUAUUCCUCCU | Translation |
| ccp-miR7530-5p | Cc00_g01310 | 3.0 | 4.788 | 1 | 22 | 2348 | 2369 | CCUUCCUCUCUUCUCCCUCUUC | GGGGAGGGAGAGAAGGGGAGGG | Translation |
| ccp-miR7530-5p | Cc00_g31360 | 3.0 | 13.113 | 1 | 22 | 541 | 562 | CCUUCCUCUCUUCUCCCUCUUC | GUAGAGGGAGAGGAGGAGAGGG | Cleavage |
| ccp-miR7530-5p | Cc01_g03320 | 3.0 | 14.009 | 1 | 21 | 312 | 332 | CCUUCCUCUCUUCUCCCUCUU | GGGAGAGGGAGGAGGGGAAGG | Cleavage |
| ccp-miR7530-5p | Cc01_g05700 | 3.0 | 13.059 | 1 | 22 | 973 | 994 | CCUUCCUCUCUUCUCCCUCUUC | GGGGAGGGGGAAGAGGGAAAGG | Cleavage |
| ccp-miR7530-5p | Cc01_g07660 | 2.5 | 23.021 | 1 | 22 | 586 | 608 | CCUUCCUCUCUUCUCC-CUCUUC | GGAGAGAGGAGAAGAGAGGGAGG | Cleavage |
| ccp-miR7530-5p | Cc01_g09020 | 3.0 | 17.975 | 1 | 22 | 630 | 651 | CCUUCCUCUCUUCUCCCUCUUC | GGAGAGUGAGAAGAGGGGAAAG | Cleavage |
| ccp-miR7530-5p | Cc01_g10600 | 3.0 | 10.327 | 1 | 21 | 2281 | 2301 | CCUUCCUCUCUUCUCCCUCUU | GAGAGAGAGAAGAGGAGAAGG | Cleavage |
| ccp-miR7530-5p | Cc01_g11520 | 3.0 | 9.318 | 1 | 22 | 291 | 312 | CCUUCCUCUCUUCUCCCUCUUC | GGAAAAGGAGGAGAGAGGGAGG | Cleavage |
| ccp-miR7530-5p | Cc01_g12890 | 3.0 | 1.939 | 1 | 21 | 831 | 851 | CCUUCCUCUCUUCUCCCUCUU | AUGAGGAAGAGGAGGGGAAGG | Cleavage |
| ccp-miR7530-5p | Cc01_g16900 | 2.5 | 9.13 | 1 | 22 | 1758 | 1779 | CCUUCCUCUCUUCUCCCUCUUC | GAGGAGGGAGAAGAGACGGAGG | Cleavage |
| ccp-miR7530-5p | Cc02_g03620 | 3.0 | 19.573 | 1 | 21 | 926 | 946 | CCUUCCUCUCUUCUCCCUCUU | GAGAGGGAGAGGGAGGGAGGG | Cleavage |
| ccp-miR7530-5p | Cc02_g07670 | 3.0 | 12.399 | 1 | 22 | 1188 | 1209 | CCUUCCUCUCUUCUCCCUCUUC | GAAAAGGGAAAAGAGAGGGGGG | Cleavage |
| ccp-miR7530-5p | Cc02_g12050 | 3.0 | 22.093 | 1 | 21 | 1110 | 1130 | CCUUCCUCUCUUCUCCCUCUU | AAAGGGGAGAAGAGAGGAGGA | Cleavage |
| ccp-miR7530-5p | Cc02_g23940 | 3.0 | 14.81 | 1 | 20 | 1412 | 1431 | CCUUCCUCUCUUCUCCCUCU | AGAUGGAGAAGGGAGGAGGA | Cleavage |
| ccp-miR7530-5p | Cc02_g30350 | 3.0 | 14.561 | 1 | 21 | 791 | 811 | CCUUCCUCUCUUCUCCCUCUU | GGGAUGUGGAAGAGAGGAAGG | Cleavage |
| ccp-miR7530-5p | Cc02_g32550 | 2.5 | 19.073 | 1 | 20 | 387 | 406 | CCUUCCUCUCUUCUCCCUCU | AGAGGGAGAAGACAUGAAGG | Cleavage |
| ccp-miR7530-5p | Cc02_g39260 | 3.0 | 17.451 | 1 | 20 | 811 | 830 | CCUUCCUCUCUUCUCCCUCU | GGAGGGGGAGGAGAGAAAGG | Cleavage |
| ccp-miR7530-5p | Cc02_g39830 | 3.0 | 19.677 | 1 | 21 | 648 | 669 | CCUUCCUCUCUUC-UCCCUCUU | AGGAGGGAAGAAGAGAGGAAGA | Cleavage |
| ccp-miR7530-5p | Cc03_g04130 | 3.0 | 3.443 | 1 | 22 | 341 | 362 | CCUUCCUCUCUUCUCCCUCUUC | GGGGAAGAAGGAGAGAGGAAGG | Cleavage |
| ccp-miR7530-5p | Cc04_g00350 | 3.0 | 13.96 | 1 | 21 | 1996 | 2016 | CCUUCCUCUCUUCUCCCUCUU | AAGAGGAAGAAGGGAAGAAGG | Cleavage |
| ccp-miR7530-5p | Cc04_g06030 | 3.0 | 13.486 | 1 | 21 | 99 | 119 | CCUUCCUCUCUUCUCCCUCUU | AAGGGGGAGGGGAGAGGGAGA | Cleavage |
| ccp-miR7530-5p | Cc04_g09510 | 3.0 | 4.123 | 1 | 22 | 937 | 959 | CCUUCCUC-UCUUCUCCCUCUUC | GAAGAAGGAGAAGAAGAGGAAGG | Translation |
| ccp-miR7530-5p | Cc05_g06890 | 2.5 | 11.285 | 1 | 22 | 890 | 911 | CCUUCCUCUCUUCUCCCUCUUC | GAAGAGGGAAAAGGGAGGAAGA | Cleavage |
| ccp-miR7530-5p | Cc05_g14880 | 2.5 | 17.733 | 1 | 21 | 84 | 104 | CCUUCCUCUCUUCUCCCUCUU | GAGAGAGAGAGGAGGGGAGGG | Cleavage |
| ccp-miR7530-5p | Cc05_g16070 | 3.0 | 18.672 | 1 | 22 | 2221 | 2242 | CCUUCCUCUCUUCUCCCUCUUC | GGACAGGGAGAAAGGAGGAGGG | Translation |
| ccp-miR7530-5p | Cc06_g10970 | 3.0 | 19.152 | 1 | 21 | 16 | 36 | CCUUCCUCUCUUCUCCCUCUU | AGGAGGGAGAGGAAAGGAAGC | Cleavage |
| ccp-miR7530-5p | Cc06_g17930 | 3.0 | 16.431 | 1 | 22 | 3449 | 3470 | CCUUCCUCUCUUCUCCCUCUUC | GGAGGGGGGGGGGGGGGGAAGG | Cleavage |
| ccp-miR7530-5p | Cc06_g21350 | 3.0 | 14.417 | 1 | 21 | 2322 | 2342 | CCUUCCUCUCUUCUCCCUCUU | GAGAGGGAGAGGAGAGCAAGA | Cleavage |
| ccp-miR7530-5p | Cc07_g00310 | 2.5 | 18.144 | 1 | 22 | 123 | 144 | CCUUCCUCUCUUCUCCCUCUUC | GAAGAGGGGAAAGAGAGGAAGA | Cleavage |
| ccp-miR7530-5p | Cc07_g02320 | 3.0 | 1.91 | 1 | 21 | 1118 | 1138 | CCUUCCUCUCUUCUCCCUCUU | GAGAGAGAGAAGAGGGAAAGG | Cleavage |
| ccp-miR7530-5p | Cc07_g05650 | 3.0 | 22.547 | 1 | 21 | 163 | 183 | CCUUCCUCUCUUCUCCCUCUU | AAAAGGGGGAAGAUAGGGAGG | Cleavage |
| ccp-miR7530-5p | Cc07_g09850 | 3.0 | 20.444 | 1 | 21 | 1478 | 1498 | CCUUCCUCUCUUCUCCCUCUU | AAGAGGUGGAAGAGAGCAAGG | Cleavage |
| ccp-miR7530-5p | Cc07_g10710 | 2.5 | 16.567 | 1 | 22 | 1626 | 1647 | CCUUCCUCUCUUCUCCCUCUUC | GAAGAGGGAGAAGAUGGGAAGA | Cleavage |
| ccp-miR7530-5p | Cc07_g14360 | 2.0 | 17.969 | 1 | 22 | 180 | 201 | CCUUCCUCUCUUCUCCCUCUUC | GCGGAGGCAGAGGAGAGGAAGG | Cleavage |
| ccp-miR7530-5p | Cc08_g03320 | 3.0 | 5.376 | 1 | 21 | 349 | 369 | CCUUCCUCUCUUCUCCCUCUU | AAGAGGGAGCAGAGAGAAGGG | Cleavage |
| ccp-miR7530-5p | Cc08_g03340 | 2.5 | 3.547 | 1 | 22 | 42 | 63 | CCUUCCUCUCUUCUCCCUCUUC | GAAGCGGGAGAAGAGAGAAAGG | Cleavage |
| ccp-miR7530-5p | Cc08_g08730 | 3.0 | 10.685 | 1 | 22 | 2770 | 2791 | CCUUCCUCUCUUCUCCCUCUUC | GUAGAGGGAGAGGAGGAGAGGG | Cleavage |
| ccp-miR7530-5p | Cc08_g10640 | 3.0 | 13.92 | 1 | 22 | 1238 | 1259 | CCUUCCUCUCUUCUCCCUCUUC | GUAGAGGGAGCGGAGAGUAAGG | Cleavage |
| ccp-miR7530-5p | Cc08_g16470 | 2.5 | 8.609 | 1 | 22 | 10 | 31 | CCUUCCUCUCUUCUCCCUCUUC | GAGUAGGGAGGAGGGAGGAAGG | Cleavage |
| ccp-miR7530-5p | Cc09_g02130 | 3.0 | 6.646 | 1 | 21 | 352 | 372 | CCUUCCUCUCUUCUCCCUCUU | GAGAGGGGGGGGGGGGGGAGG | Cleavage |
| ccp-miR7530-5p | Cc09_g08610 | 3.0 | 11.815 | 1 | 21 | 1525 | 1545 | CCUUCCUCUCUUCUCCCUCUU | AAGAGGCAGAAAAGAGGAAGU | Translation |
| ccp-miR7530-5p | Cc10_g00110 | 3.0 | 21.683 | 1 | 21 | 61 | 81 | CCUUCCUCUCUUCUCCCUCUU | GGGGAGGAGAGGAGAGGAGGG | Cleavage |
| ccp-miR7530-5p | Cc11_g04490 | 2.5 | 13.525 | 1 | 21 | 100 | 120 | CCUUCCUCUCUUCUCCCUCUU | AAGGGGGAGGGGAGAGGAAGA | Cleavage |
| ccp-miR7530-5p | Cc11_g17110 | 3.0 | 4.651 | 1 | 21 | 258 | 278 | CCUUCCUCUCUUCUCCCUCUU | GAGAGGUAGGAGAGAGGAUGG | Cleavage |
| ccp-miR7696c-3p | Cc00_g32620 | 2.5 | 15.479 | 1 | 20 | 638 | 657 | UUUUGAAUUAUGCGAAUUUG | CAAAUUCGUUGAAUUCAAAA | Translation |
| ccp-miR7696c-3p | Cc00_g35640 | 3.0 | 10.951 | 1 | 20 | 792 | 811 | UUUUGAAUUAUGCGAAUUUG | UAAAUUCACGGAAUUCAAAA | Translation |
| ccp-miR7696c-3p | Cc00_g35680 | 3.0 | 11.018 | 1 | 20 | 371 | 390 | UUUUGAAUUAUGCGAAUUUG | UAAAUUCACGGAAUUCAAAA | Translation |
| ccp-miR7696c-3p | Cc06_g17480 | 3.0 | 9.624 | 1 | 20 | 1710 | 1729 | UUUUGAAUUAUGCGAAUUUG | UAAAUUUGUGUAAUUCAAAU | Cleavage |
| ccp-miR7696c-3p | Cc07_g09480 | 3.0 | 8.975 | 1 | 21 | 959 | 979 | UUUUGAAUUAUGCGAAUUUGG | UCAAAGUUCCAUAAUUUAAAA | Cleavage |
| ccp-miR7696c-3p | Cc09_g01940 | 3.0 | 17.267 | 1 | 20 | 1019 | 1038 | UUUUGAAUUAUGCGAAUUUG | CUGAUUUGUAUAAUUCAGAA | Cleavage |
| ccp-miR7696c-3p | Cc10_g02300 | 3.0 | 16.323 | 1 | 21 | 1232 | 1252 | UUUUGAAUUAUGCGAAUUUGG | UUAUAUUCACAUAAUUCGAAA | Cleavage |
| ccp-miR7696c-3p | Cc10_g03820 | 3.0 | 12.656 | 1 | 20 | 3358 | 3377 | UUUUGAAUUAUGCGAAUUUG | UAGAUACGCAUCAUUCAAAA | Translation |
| ccp-miR7696c-3p | Cc10_g11630 | 3.0 | 21.416 | 1 | 21 | 1266 | 1286 | UUUUGAAUUAUGCGAAUUUGG | UCAAAUUCAGAUAGUUCAGAA | Cleavage |
| ccp-miR7696c-3p | Cc11_g01380 | 3.0 | 9.005 | 1 | 20 | 1149 | 1168 | UUUUGAAUUAUGCGAAUUUG | UAAAUUCGCAAAAUUUAAAC | Translation |
| ccp-miR7696c-3p | Cc11_g06670 | 3.0 | 8.68 | 1 | 20 | 140 | 159 | UUUUGAAUUAUGCGAAUUUG | UAAAUUAGUAUAAUUCAAGG | Cleavage |
| ccp-miR7743-5p | Cc00_g01950 | 3.0 | 17.159 | 1 | 24 | 864 | 887 | UUUGAACUUUUGUAAUGGUUCUCA | UGUCAACCAUUGUCAGAGUUCAGA | Translation |
| ccp-miR7743-5p | Cc00_g11530 | 3.0 | 17.747 | 1 | 21 | 3057 | 3077 | UUUGAACUUUUGUAAUGGUUC | GGGCCAUUAAAGAAGUUCAGA | Cleavage |
| ccp-miR7743-5p | Cc00_g16160 | 2.5 | 16.28 | 1 | 20 | 534 | 553 | UUUGAACUUUUGUAAUGGUU | GAUCUUUAUAAAAGUUCAAA | Cleavage |
| ccp-miR7743-5p | Cc00_g27110 | 3.0 | 13.722 | 1 | 24 | 1221 | 1244 | UUUGAACUUUUGUAAUGGUUCUCA | UCAAAGCCAUUUAAGAAGUUCAAA | Cleavage |
| ccp-miR7743-5p | Cc01_g02870 | 3.0 | 14.391 | 1 | 21 | 1588 | 1608 | UUUGAACUUUUGUAAUGGUUC | GAAAUAUUAUCAAAGUUCAAA | Translation |
| ccp-miR7743-5p | Cc02_g21380 | 3.0 | 19.868 | 1 | 22 | 1645 | 1666 | UUUGAACUUUUGUAAUGGUUCU | GGAACCAUGUCAAAGGUUCAGA | Cleavage |
| ccp-miR7743-5p | Cc02_g31130 | 3.0 | 15.131 | 1 | 22 | 340 | 361 | UUUGAACUUUUGUAAUGGUUCU | ACAACCAUUGGAGCAGUUCAAA | Translation |
| ccp-miR7743-5p | Cc02_g38780 | 2.5 | 11.654 | 1 | 23 | 1718 | 1740 | UUUGAACUUUUGUAAUGGUUCUC | GGGAACCAUUAAAAAAGUUGAAA | Cleavage |
| ccp-miR7743-5p | Cc03_g07770 | 3.0 | 16.94 | 1 | 24 | 575 | 598 | UUUGAACUUUUGUAAUGGUUCUCA | UGGAAACUGUUGGAGAAGUUCAAA | Cleavage |
| ccp-miR7743-5p | Cc05_g08070 | 2.0 | 14.67 | 1 | 21 | 2011 | 2031 | UUUGAACUUUUGUAAUGGUUC | GAAGCACUACAAAAGUUCAAA | Cleavage |
| ccp-miR7743-5p | Cc05_g13300 | 3.0 | 16.775 | 1 | 22 | 506 | 527 | UUUGAACUUUUGUAAUGGUUCU | AGAACCCUUAAAGAAGUUCAAG | Cleavage |
| ccp-miR7743-5p | Cc06_g10270 | 3.0 | 20.719 | 1 | 24 | 433 | 456 | UUUGAACUUUUGUAAUGGUUCUCA | UUAGAGCUAUUGCAACAGUUCAAG | Translation |
| ccp-miR7743-5p | Cc06_g10560 | 3.0 | 19.417 | 1 | 23 | 510 | 532 | UUUGAACUUUUGUAAUGGUUCUC | GUGGGCCGUUACAAAAAUUCAAA | Cleavage |
| ccp-miR7743-5p | Cc06_g22140 | 3.0 | 12.204 | 1 | 24 | 121 | 144 | UUUGAACUUUUGUAAUGGUUCUCA | UCAAAGCCAUUUAAGAAGUUCAAA | Cleavage |
| ccp-miR7743-5p | Cc10_g11360 | 3.0 | 13.095 | 1 | 24 | 52 | 75 | UUUGAACUUUUGUAAUGGUUCUCA | UCAAAGCCAUUUAAGAAGUUCAAA | Cleavage |
| ccp-miR7822-3p | Cc00_g17070 | 3.0 | 15.626 | 1 | 21 | 1007 | 1028 | UUUGAAAUUGAAU-AAGUGGUG | CACCACUUGACUCAAUUUCAAA | Cleavage |
| ccp-miR7822-3p | Cc01_g01860 | 3.0 | 10.002 | 1 | 20 | 139 | 158 | UUUGAAAUUGAAUAAGUGGU | AUCAAUUAUUCAAUUUUAGG | Cleavage |
| ccp-miR7822-3p | Cc02_g05370 | 2.5 | 8.035 | 1 | 21 | 46 | 66 | UUUGAAAUUGAAUAAGUGGUG | CGCCUCUUCUUCAAUUUCAAA | Cleavage |
| ccp-miR7822-3p | Cc03_g15890 | 3.0 | 16.194 | 1 | 20 | 186 | 205 | UUUGAAAUUGAAUAAGUGGU | ACUGCUAAUUCAAUUUCAGG | Cleavage |
| ccp-miR7822-3p | Cc05_g10990 | 2.5 | 7.481 | 1 | 20 | 30 | 50 | UUUGAAAUUGAAU-AAGUGGU | AUCACUUCAUUCAAUUUCAAA | Cleavage |
| ccp-miR7822-3p | Cc07_g02890 | 3.0 | 22.002 | 1 | 20 | 1937 | 1956 | UUUGAAAUUGAAUAAGUGGU | GCCACUCCUUCAAUUUUAAA | Cleavage |
| ccp-miR7822-3p | Cc08_g04010 | 2.5 | 20.521 | 1 | 21 | 1747 | 1767 | UUUGAAAUUGAAUAAGUGGUG | UACCGUUUGUUCAGUUUCAGA | Cleavage |
| ccp-miR7822-3p | Cc08_g05090 | 3.0 | 15.438 | 1 | 21 | 1235 | 1255 | UUUGAAAUUGAAUAAGUGGUG | CAUCAUUUCUUGAAUUUCAAA | Translation |
| ccp-miR7828-3p | Cc00_g20750 | 3.0 | 21.243 | 1 | 21 | 1717 | 1737 | GAUGACAUGGGCAUCAAAAUG | UAUCUUGAAGCUUAUGUCAUC | Cleavage |
| ccp-miR7828-3p | Cc02_g11230 | 2.5 | 20.818 | 1 | 21 | 1770 | 1790 | GAUGACAUGGGCAUCAAAAUG | CAUAUUGAAGCUCAUGUCAUC | Cleavage |
| ccp-miR7828-3p | Cc03_g15950 | 3.0 | 14.685 | 1 | 20 | 1016 | 1035 | GAUGACAUGGGCAUCAAAAU | AUGUUGAAGCUCAUGUUAUC | Cleavage |
| ccp-miR7828-3p | Cc05_g06510 | 3.0 | 19.928 | 1 | 21 | 247 | 267 | GAUGACAUGGGCAUCAAAAUG | CAAUUCCAUGCCCAUGUCAUC | Cleavage |
| ccp-miR7828-3p | Cc06_g03980 | 3.0 | 21.243 | 1 | 21 | 2396 | 2416 | GAUGACAUGGGCAUCAAAAUG | UAUCUUGAAGCUUAUGUCAUC | Cleavage |
| ccp-miR7982a-1-5p | Cc00_g28630 | 2.0 | 16.457 | 1 | 24 | 1160 | 1182 | AAGUUGGAUGCUUAUGAUAUAUAU | AUAUAUAUCAUAAGCA-CCAACUU | Cleavage |
| ccp-miR7982a-1-5p | Cc01_g06550 | 3.0 | 11.223 | 1 | 24 | 137 | 160 | AAGUUGGAUGCUUAUGAUAUAUAU | AUUUGUAUUACAAGCAACCAACUU | Cleavage |
| ccp-miR7982a-1-5p | Cc02_g25030 | 3.0 | 20.312 | 1 | 20 | 467 | 486 | AAGUUGGAUGCUUAUGAUAU | AUAUCAUUAGUAUCAAACUU | Cleavage |
| ccp-miR7982a-1-5p | Cc07_g01450 | 3.0 | 12.176 | 1 | 21 | 3042 | 3062 | AAGUUGGAUGCUUAUGAUAUA | UAAAUCAUAACCACCCAACUU | Translation |
| ccp-miR7982a-1-5p | Cc08_g17160 | 3.0 | 10.771 | 1 | 22 | 3286 | 3307 | AAGUUGGAUGCUUAUGAUAUAU | AAAUAUCAUCACCAUCUAAUUU | Translation |
| ccp-miR7982a-1-5p | Cc10_g11170 | 2.5 | 9.46 | 1 | 22 | 1055 | 1076 | AAGUUGGAUGCUUAUGAUAUAU | AAGUUUUAUAAGCAUCCAGCUU | Cleavage |
| ccp-miR7982a-2-5p | Cc00_g28630 | 2.0 | 16.457 | 1 | 24 | 1160 | 1182 | AAGUUGGAUGCUUAUGAUAUAUAU | AUAUAUAUCAUAAGCA-CCAACUU | Cleavage |
| ccp-miR7982a-2-5p | Cc01_g06550 | 3.0 | 11.223 | 1 | 24 | 137 | 160 | AAGUUGGAUGCUUAUGAUAUAUAU | AUUUGUAUUACAAGCAACCAACUU | Cleavage |
| ccp-miR7982a-2-5p | Cc02_g25030 | 3.0 | 20.312 | 1 | 20 | 467 | 486 | AAGUUGGAUGCUUAUGAUAU | AUAUCAUUAGUAUCAAACUU | Cleavage |
| ccp-miR7982a-2-5p | Cc07_g01450 | 3.0 | 12.176 | 1 | 21 | 3042 | 3062 | AAGUUGGAUGCUUAUGAUAUA | UAAAUCAUAACCACCCAACUU | Translation |
| ccp-miR7982a-2-5p | Cc08_g17160 | 3.0 | 10.771 | 1 | 22 | 3286 | 3307 | AAGUUGGAUGCUUAUGAUAUAU | AAAUAUCAUCACCAUCUAAUUU | Translation |
| ccp-miR7982a-2-5p | Cc10_g11170 | 2.5 | 9.46 | 1 | 22 | 1055 | 1076 | AAGUUGGAUGCUUAUGAUAUAU | AAGUUUUAUAAGCAUCCAGCUU | Cleavage |
| ccp-miR7997c-3p | Cc02_g05340 | 3.0 | 20.857 | 1 | 22 | 2770 | 2791 | AUAUUGCUCUGAUUCCCCAAAA | UUUCGUGGAAUGAGAGCAAUAU | Translation |
| ccp-miR7997c-3p | Cc05_g10360 | 3.0 | 13.166 | 1 | 20 | 2373 | 2392 | AUAUUGCUCUGAUUCCCCAA | UUGGGGAAAUGGAGUGAUAU | Cleavage |
| ccp-miR8001b-5p | Cc02_g30580 | 3.0 | 16.504 | 1 | 21 | 1332 | 1352 | GGAUUUUCAAACUACUCCCUA | UGGGGAUAGGUUUGAAAAUCC | Cleavage |
| ccp-miR8001b-5p | Cc02_g36370 | 3.0 | 13.137 | 1 | 21 | 1663 | 1683 | GGAUUUUCAAACUACUCCCUA | UUGGGAGUUGUUUGAAAAUCA | Cleavage |
| ccp-miR8001b-5p | Cc06_g14440 | 3.0 | 14.721 | 1 | 22 | 174 | 194 | GGAUUUUCAAACUACUCCCUAC | GCAGGGAG-AGUUGGAAAAUCC | Translation |
| ccp-miR8001b-5p | Cc07_g17380 | 3.0 | 19.306 | 1 | 21 | 572 | 592 | GGAUUUUCAAACUACUCCCUA | UAGGGAGUUGUUUGAGAAGCC | Cleavage |
| ccp-miR8001b-5p | Cc08_g03300 | 3.0 | 16.829 | 1 | 21 | 690 | 710 | GGAUUUUCAAACUACUCCCUA | UAAGGAGGAGUUUGAGGAUCC | Cleavage |
| ccp-miR8001b-5p | Cc09_g10770 | 3.0 | 15.901 | 1 | 23 | 1484 | 1506 | GGAUUUUCAAACUACUCCCUACA | UGGAGGAAGGAGUAUGAAAAUCC | Translation |
| ccp-miR8001b-5p | Cc09_g10940 | 3.0 | 13.214 | 1 | 20 | 1580 | 1598 | GGAUUUUCAAACUACUCCCU | AGGGAG-AAUUUGAAAAUCC | Cleavage |
| ccp-miR8044-5p | Cc00_g26830 | 3.0 | 14.654 | 1 | 21 | 85 | 105 | UUUCAAAUAUCUUUGGAGAUU | AAUCUCCAGAGCUUUUUGAAG | Translation |
| ccp-miR8044-5p | Cc02_g32330 | 3.0 | 13.154 | 1 | 21 | 1457 | 1477 | UUUCAAAUAUCUUUGGAGAUU | AGUUGUCAAAGAUAUUUGAAG | Cleavage |
| ccp-miR8044-5p | Cc02_g34250 | 3.0 | 18.808 | 1 | 19 | 299 | 318 | UUUCAAAUAUCU-UUGGAGA | UCUCCAAGAGGUAUUUGAAG | Cleavage |
| ccp-miR8044-5p | Cc04_g05550 | 3.0 | 12.996 | 1 | 20 | 631 | 650 | UUUCAAAUAUCUUUGGAGAU | AUCUACAAAGACAUUUGAAC | Translation |
| ccp-miR8044-5p | Cc05_g11400 | 3.0 | 13.154 | 1 | 21 | 2086 | 2106 | UUUCAAAUAUCUUUGGAGAUU | AGUUGUCAAAGAUAUUUGAAG | Cleavage |
| ccp-miR8044-5p | Cc05_g13760 | 3.0 | 10.842 | 1 | 20 | 2043 | 2062 | UUUCAAAUAUCUUUGGAGAU | CACUCCAAAUAUAUUUGAAA | Translation |
| ccp-miR8044-5p | Cc06_g04620 | 3.0 | 17.142 | 1 | 21 | 4202 | 4222 | UUUCAAAUAUCUUUGGAGAUU | AAUCUCUAGAAAGAUUUGAAA | Translation |
| ccp-miR8044-5p | Cc06_g12780 | 3.0 | 18.795 | 1 | 20 | 2106 | 2125 | UUUCAAAUAUCUUUGGAGAU | AUCUCCACGGAUAUUGGAAA | Cleavage |
| ccp-miR8044-5p | Cc06_g17060 | 3.0 | 12.003 | 1 | 20 | 2722 | 2741 | UUUCAAAUAUCUUUGGAGAU | UUUUCUGAAGAUGUUUGAAA | Cleavage |
| ccp-miR8044-5p | Cc06_g23550 | 3.0 | 12.929 | 1 | 21 | 1593 | 1613 | UUUCAAAUAUCUUUGGAGAUU | AAUCUCAGAAGAUAUUGGAAA | Cleavage |
| ccp-miR8044-5p | Cc07_g01200 | 3.0 | 22.887 | 1 | 20 | 1343 | 1362 | UUUCAAAUAUCUUUGGAGAU | AUUUCCAAAGCUGUUUGGAG | Translation |
| ccp-miR821b-5p | Cc01_g04330 | 3.0 | 21.409 | 1 | 21 | 1164 | 1184 | AAGUUAUGAACAUAAAAAAUG | UGUUUUUGAUGUCCAUAAUUU | Translation |
| ccp-miR821b-5p | Cc02_g13480 | 3.0 | 13.009 | 1 | 21 | 1134 | 1154 | AAGUUAUGAACAUAAAAAAUG | UAUUUUUUAUGUUCAGAAUUA | Cleavage |
| ccp-miR821b-5p | Cc03_g04880 | 3.0 | 16.397 | 1 | 21 | 1541 | 1561 | AAGUUAUGAACAUAAAAAAUG | UAUGUUUUAUGUUUAUAAGUU | Cleavage |
| ccp-miR821b-5p | Cc06_g04800 | 3.0 | 20.927 | 1 | 20 | 97 | 116 | AAGUUAUGAACAUAAAAAAU | AUUUGUUGUGUUGAUAAUUU | Cleavage |
| ccp-miR821b-5p | Cc06_g12680 | 3.0 | 9.315 | 1 | 20 | 423 | 442 | AAGUUAUGAACAUAAAAAAU | UUUUUUUUUGUUUAUAAUUU | Cleavage |
| ccp-miR821b-5p | Cc06_g13690 | 3.0 | 17.369 | 1 | 21 | 274 | 294 | AAGUUAUGAACAUAAAAAAUG | CACUUUUUAUGUGCAUGGCUU | Translation |
| ccp-miR821b-5p | Cc07_g15770 | 3.0 | 3.521 | 1 | 20 | 220 | 239 | AAGUUAUGAACAUAAAAAAU | AUUUUUUAUAUCUAUAGCUU | Translation |
| ccp-miR821b-5p | Cc07_g19800 | 3.0 | 12.176 | 1 | 21 | 2776 | 2796 | AAGUUAUGAACAUAAAAAAUG | UGUUUUCAAUGUUUAUAACUU | Cleavage |
| ccp-miR821b-5p | Cc07_g20270 | 3.0 | 12.646 | 1 | 20 | 1290 | 1309 | AAGUUAUGAACAUAAAAAAU | AUUUUUGAUGAUGAUAACUU | Translation |
| ccp-miR821b-5p | Cc10_g11500 | 2.0 | 11.075 | 1 | 21 | 1246 | 1266 | AAGUUAUGAACAUAAAAAAUG | UAUUUUUUAUUUUUAUAAUUU | Translation |
| ccp-miR827-5p | Cc02_g00840 | 3.0 | 21.305 | 1 | 20 | 436 | 455 | UUUUGUUGGUGAUCAUCUAA | UUAGCUGAUCACCAGCAAGU | Cleavage |
| ccp-miR827-5p | Cc02_g04070 | 2.0 | 22.109 | 1 | 20 | 919 | 938 | UUUUGUUGGUGAUCAUCUAA | UUAGAUGAUCGCCGGCAAGA | Cleavage |
| ccp-miR827-5p | Cc02_g07670 | 3.0 | 11.703 | 1 | 22 | 1020 | 1041 | UUUUGUUGGUGAUCAUCUAAGG | UUUUAUAUGAACAACAACAAAA | Translation |
| ccp-miR827-5p | Cc02_g35920 | 3.0 | 16.913 | 1 | 22 | 1999 | 2020 | UUUUGUUGGUGAUCAUCUAAGG | CAUUGGAAAAUCACCAGCAAAA | Cleavage |
| ccp-miR827-5p | Cc04_g05710 | 3.0 | 22.08 | 1 | 20 | 2063 | 2082 | UUUUGUUGGUGAUCAUCUAA | UUAGAUCAUCACGAGCAAGA | Cleavage |
| ccp-miR827-5p | Cc04_g10970 | 2.5 | 12.356 | 1 | 20 | 1267 | 1286 | UUUUGUUGGUGAUCAUCUAA | UUAGAGGAUCUCCAACAAGA | Translation |
| ccp-miR827-5p | Cc04_g16840 | 2.5 | 18.561 | 1 | 21 | 677 | 697 | UUUUGUUGGUGAUCAUCUAAG | UUCAGGUGAUGACCAACAAAA | Translation |
| ccp-miR827-5p | Cc06_g03470 | 3.0 | 12.358 | 1 | 20 | 790 | 809 | UUUUGUUGGUGAUCAUCUAA | UUGGGUGCUCACUAACAAGA | Cleavage |
| ccp-miR827-5p | Cc06_g22440 | 3.0 | 16.22 | 1 | 20 | 71 | 90 | UUUUGUUGGUGAUCAUCUAA | UGAGAUCAUCAACAACAAAA | Translation |
| ccp-miR827-5p | Cc07_g19870 | 3.0 | 11.132 | 1 | 21 | 383 | 402 | UUUUGUUGGUGAUCAUCUAAG | UUUAGAUGAU-GCCAACAAGA | Translation |
| ccp-miR827-5p | Cc09_g04400 | 2.5 | 20.323 | 1 | 22 | 4620 | 4641 | UUUUGUUGGUGAUCAUCUAAGG | CAUUAGAUAAUCAUCAGCAGAA | Cleavage |
| ccp-miR827-5p | Cc10_g01300 | 3.0 | 8.04 | 1 | 20 | 352 | 371 | UUUUGUUGGUGAUCAUCUAA | UCAGAUGAAGACCAACAAAA | Translation |
| ccp-miR827-5p | Cc10_g07860 | 3.0 | 15.531 | 1 | 21 | 1603 | 1623 | UUUUGUUGGUGAUCAUCUAAG | CUCAGAUGAUUACUGACAAAG | Cleavage |
| ccp-miR828a-3p | Cc02_g09670 | 3.0 | 7.385 | 1 | 22 | 705 | 726 | AGAUACUCAUUUGAACAAGAUG | UAUCUUUUCAAAAUGAGUAUCU | Cleavage |
| ccp-miR828a-3p | Cc02_g39620 | 0.0 | 18.88 | 1 | 22 | 1279 | 1300 | AGAUACUCAUUUGAACAAGAUG | CAUCUUGUUCAAAUGAGUAUCU | Cleavage |
| ccp-miR828a-3p | Cc05_g15650 | 2.5 | 14.602 | 1 | 21 | 1042 | 1062 | AGAUACUCAUUUGAACAAGAU | AUUUUGUUCAAAUGAUUAUUU | Cleavage |
| ccp-miR828a-3p | Cc07_g21350 | 3.0 | 16.102 | 1 | 22 | 696 | 717 | AGAUACUCAUUUGAACAAGAUG | CUUCUUGUUCGAAAGGGUGUUU | Translation |
| ccp-miR828a-5p | Cc00_g21090 | 3.0 | 12.298 | 1 | 22 | 1115 | 1135 | UCUUGCUCAAAUGAGUAUUCCA | UGGAAUA-UCAUUUGGGUAAGA | Cleavage |
| ccp-miR828a-5p | Cc02_g17830 | 3.0 | 13.672 | 1 | 20 | 1420 | 1439 | UCUUGCUCAAAUGAGUAUUC | AGAUGCUCAUUUGAGCAAGU | Cleavage |
| ccp-miR828a-5p | Cc02_g39620 | 0.0 | 18.204 | 1 | 22 | 1389 | 1410 | UCUUGCUCAAAUGAGUAUUCCA | UGGAAUACUCAUUUGAGCAAGA | Cleavage |
| ccp-miR828a-5p | Cc04_g01360 | 1.0 | 16.041 | 1 | 22 | 431 | 452 | UCUUGCUCAAAUGAGUAUUCCA | UGGAAUUCUCAUUUGAGCAAGA | Cleavage |
| ccp-miR828a-5p | Cc04_g16640 | 3.0 | 11.893 | 1 | 22 | 541 | 562 | UCUUGCUCAAAUGAGUAUUCCA | UGGAAUACUCAUUUGAAAAAGA | Cleavage |
| ccp-miR828a-5p | Cc05_g09530 | 2.5 | 19.318 | 1 | 20 | 560 | 579 | UCUUGCUCAAAUGAGUAUUC | GGAAACUCAUUGGAGCAAGA | Translation |
| ccp-miR828a-5p | Cc05_g09780 | 3.0 | 14.591 | 1 | 22 | 701 | 722 | UCUUGCUCAAAUGAGUAUUCCA | UAGGAAACUCAUUGGGGCAAGA | Translation |
| ccp-miR828a-5p | Cc08_g12180 | 1.0 | 9.574 | 1 | 22 | 2963 | 2984 | UCUUGCUCAAAUGAGUAUUCCA | UGGAAUUCUCAUUUGAGCAAGA | Cleavage |
| ccp-miR837-5p | Cc00_g07000 | 3.0 | 15.663 | 1 | 20 | 197 | 216 | CAUUGUUUCUUGUUUUUUUA | UAAGAAAAUGGCAAACAAUG | Translation |
| ccp-miR837-5p | Cc00_g16360 | 2.5 | 12.146 | 1 | 20 | 852 | 871 | CAUUGUUUCUUGUUUUUUUA | UCAGAGAGCAAGAAACAAUG | Cleavage |
| ccp-miR837-5p | Cc00_g29470 | 3.0 | 9.504 | 1 | 20 | 1388 | 1407 | CAUUGUUUCUUGUUUUUUUA | GAAGGGAGCAAGAAACAAUG | Cleavage |
| ccp-miR837-5p | Cc01_g05810 | 3.0 | 11.952 | 1 | 20 | 492 | 511 | CAUUGUUUCUUGUUUUUUUA | UAGAGUAAUAGGAAACAAUG | Cleavage |
| ccp-miR837-5p | Cc01_g11590 | 3.0 | 20.919 | 1 | 21 | 360 | 380 | CAUUGUUUCUUGUUUUUUUAA | UUAAGAAAAUAAGAGACGAUC | Cleavage |
| ccp-miR837-5p | Cc01_g16380 | 2.5 | 18.521 | 1 | 20 | 1238 | 1257 | CAUUGUUUCUUGUUUUUUUA | UAUAGAAACAAGGAGCAAUG | Cleavage |
| ccp-miR837-5p | Cc01_g19050 | 3.0 | 11.296 | 1 | 21 | 3197 | 3217 | CAUUGUUUCUUGUUUUUUUAA | UUGAGAAGGCCAGAAACAAUG | Translation |
| ccp-miR837-5p | Cc01_g20730 | 2.5 | 13.601 | 1 | 21 | 295 | 315 | CAUUGUUUCUUGUUUUUUUAA | UUAAACAGACAAAAAACAAUG | Translation |
| ccp-miR837-5p | Cc02_g05710 | 2.5 | 14.294 | 1 | 20 | 1810 | 1829 | CAUUGUUUCUUGUUUUUUUA | UAAAGAUACAAGAAACAAUU | Cleavage |
| ccp-miR837-5p | Cc02_g15120 | 3.0 | 16.44 | 1 | 20 | 111 | 130 | CAUUGUUUCUUGUUUUUUUA | UGAAGAAGCAAGCAGCAAUG | Cleavage |
| ccp-miR837-5p | Cc02_g30570 | 3.0 | 14.92 | 1 | 20 | 2282 | 2301 | CAUUGUUUCUUGUUUUUUUA | AAGAAAAAGAAGAAACAGUG | Cleavage |
| ccp-miR837-5p | Cc02_g30920 | 3.0 | 10.919 | 1 | 21 | 70 | 90 | CAUUGUUUCUUGUUUUUUUAA | UUAGAAAAACAAGAAAAAAUU | Cleavage |
| ccp-miR837-5p | Cc02_g33420 | 3.0 | 18.678 | 1 | 21 | 1856 | 1876 | CAUUGUUUCUUGUUUUUUUAA | UUAGGAAAGCCAGAAGCAAUG | Translation |
| ccp-miR837-5p | Cc02_g34130 | 3.0 | 13.352 | 1 | 20 | 591 | 610 | CAUUGUUUCUUGUUUUUUUA | UGAAAAAACAAGUAAAAAUG | Cleavage |
| ccp-miR837-5p | Cc03_g02070 | 3.0 | 7.89 | 1 | 21 | 509 | 529 | CAUUGUUUCUUGUUUUUUUAA | UAAAAAAAAGAUGAAACAAUG | Translation |
| ccp-miR837-5p | Cc03_g03360 | 2.5 | 15.731 | 1 | 20 | 2157 | 2176 | CAUUGUUUCUUGUUUUUUUA | AAAGAAAACGAGAAAUAAUG | Cleavage |
| ccp-miR837-5p | Cc03_g07880 | 3.0 | 17.973 | 1 | 21 | 321 | 341 | CAUUGUUUCUUGUUUUUUUAA | UAAAAGCAACAAGAAAUAAUG | Cleavage |
| ccp-miR837-5p | Cc03_g15860 | 2.5 | 11.807 | 1 | 20 | 334 | 353 | CAUUGUUUCUUGUUUUUUUA | UGAAAAAUCAAGAAACAAUU | Cleavage |
| ccp-miR837-5p | Cc04_g08140 | 3.0 | 9.307 | 1 | 20 | 37 | 56 | CAUUGUUUCUUGUUUUUUUA | AAAAGAAACAAGAAAUGGUG | Cleavage |
| ccp-miR837-5p | Cc04_g10280 | 2.5 | 24.238 | 1 | 20 | 308 | 327 | CAUUGUUUCUUGUUUUUUUA | UGAAAAAGCAGGAGGCAAUG | Cleavage |
| ccp-miR837-5p | Cc05_g02930 | 3.0 | 24.126 | 1 | 20 | 1503 | 1522 | CAUUGUUUCUUGUUUUUUUA | UAAGAAAAUGGGAAAUGAUG | Cleavage |
| ccp-miR837-5p | Cc05_g05360 | 3.0 | 20.826 | 1 | 20 | 1155 | 1174 | CAUUGUUUCUUGUUUUUUUA | UCAAAGGAUGAGAAACAAUG | Cleavage |
| ccp-miR837-5p | Cc05_g13540 | 3.0 | 13.523 | 1 | 21 | 1143 | 1163 | CAUUGUUUCUUGUUUUUUUAA | UUACAAAGAGAAGGAACAAUG | Cleavage |
| ccp-miR837-5p | Cc06_g00980 | 3.0 | 13.197 | 1 | 21 | 2632 | 2652 | CAUUGUUUCUUGUUUUUUUAA | UUGAAAAAGCAAGAUAUAAUG | Cleavage |
| ccp-miR837-5p | Cc06_g03560 | 3.0 | 16.4 | 1 | 20 | 2820 | 2839 | CAUUGUUUCUUGUUUUUUUA | UAAAAGAAGAAGAAAGAAUG | Cleavage |
| ccp-miR837-5p | Cc06_g12580 | 3.0 | 10.778 | 1 | 20 | 2529 | 2548 | CAUUGUUUCUUGUUUUUUUA | UAAAAGAACAAGAAAUAGAG | Cleavage |
| ccp-miR837-5p | Cc06_g14440 | 2.5 | 15.238 | 1 | 21 | 842 | 862 | CAUUGUUUCUUGUUUUUUUAA | UUAAGAAAAAGAGAGACAAUG | Cleavage |
| ccp-miR837-5p | Cc07_g10300 | 3.0 | 20.826 | 1 | 20 | 1218 | 1237 | CAUUGUUUCUUGUUUUUUUA | UCAAAGGAUGAGAAACAAUG | Cleavage |
| ccp-miR837-5p | Cc08_g01270 | 3.0 | 9.809 | 1 | 20 | 1546 | 1565 | CAUUGUUUCUUGUUUUUUUA | CAAAAAUACAAGAAAUAGUG | Cleavage |
| ccp-miR837-5p | Cc08_g03320 | 3.0 | 6.733 | 1 | 20 | 398 | 417 | CAUUGUUUCUUGUUUUUUUA | AAAAAGAGAAAGAAACAAUG | Cleavage |
| ccp-miR837-5p | Cc08_g03340 | 2.5 | 8.3 | 1 | 20 | 92 | 111 | CAUUGUUUCUUGUUUUUUUA | AAAAAGAAAAAGAAACAAUG | Cleavage |
| ccp-miR837-5p | Cc09_g00040 | 3.0 | 5.192 | 1 | 20 | 697 | 716 | CAUUGUUUCUUGUUUUUUUA | UAAAAGAACAAAGAGCAGUG | Translation |
| ccp-miR837-5p | Cc10_g07240 | 2.5 | 12.859 | 1 | 20 | 123 | 142 | CAUUGUUUCUUGUUUUUUUA | CAAGAAAACAAGGGACAAUG | Cleavage |
| ccp-miR837-5p | Cc11_g02000 | 3.0 | 11.296 | 1 | 20 | 971 | 990 | CAUUGUUUCUUGUUUUUUUA | GAAGGGAGCAAGAAACAAUG | Cleavage |
| ccp-miR837-5p | Cc11_g02020 | 3.0 | 9.504 | 1 | 20 | 632 | 651 | CAUUGUUUCUUGUUUUUUUA | GAAGGGAGCAAGAAACAAUG | Cleavage |
| ccp-miR845-3p | Cc02_g22200 | 2.5 | 19.751 | 1 | 21 | 1484 | 1504 | UGCUCUGAUACCACUUGUUGG | CCAACAAGUAGUACCAGGGCA | Cleavage |
| ccp-miR845-3p | Cc04_g15100 | 3.0 | 24.321 | 1 | 20 | 2346 | 2365 | UGCUCUGAUACCACUUGUUG | CUACAAGUGGCAUCAGAGCU | Translation |
| ccp-miR845-3p | Cc09_g00880 | 3.0 | 20.92 | 1 | 20 | 1637 | 1656 | UGCUCUGAUACCACUUGUUG | CAACGAGUGGAAUCCGAGCA | Translation |
| ccp-miR845b-2-5p | Cc00_g00870 | 1.0 | 23.962 | 1 | 20 | 436 | 455 | UCAAUUGGUAUCAGAGCUUG | CAAGCUCUGAUACCAAUUGU | Cleavage |
| ccp-miR845b-2-5p | Cc00_g07160 | 3.0 | 10.296 | 1 | 21 | 368 | 388 | UCAAUUGGUAUCAGAGCUUGG | CUAAGCUCUGAUACCAACUGU | Cleavage |
| ccp-miR845b-2-5p | Cc00_g24560 | 3.0 | 14.844 | 1 | 21 | 146 | 166 | UCAAUUGGUAUCAGAGCUUGG | CUAAGCUCUGAUACCAACUGU | Cleavage |
| ccp-miR845b-2-5p | Cc02_g01450 | 3.0 | 10.22 | 1 | 20 | 1544 | 1563 | UCAAUUGGUAUCAGAGCUUG | CAAGUCUUGACACCAAUUGA | Translation |
| ccp-miR845b-2-5p | Cc03_g01900 | 2.5 | 14.722 | 1 | 20 | 2946 | 2965 | UCAAUUGGUAUCAGAGCUUG | CAAGUUUUGGUGUCAAUUGA | Cleavage |
| ccp-miR845b-2-5p | Cc03_g07910 | 3.0 | 13.478 | 1 | 21 | 887 | 907 | UCAAUUGGUAUCAGAGCUUGG | UCAAGCCUUGAUCCCAAUUGG | Translation |
| ccp-miR845b-2-5p | Cc06_g00480 | 3.0 | 17.642 | 1 | 21 | 2903 | 2923 | UCAAUUGGUAUCAGAGCUUGG | UCGAGUUAUCAUACCAAUUGA | Cleavage |
| ccp-miR845b-2-5p | Cc07_g18890 | 3.0 | 18.57 | 1 | 20 | 1361 | 1379 | UCAAUUGGUAUCAGAGCUUG | CAAGCUUUGA-ACCAGUUGA | Translation |
| ccp-miR845b-2-5p | Cc08_g02450 | 3.0 | 23.208 | 1 | 20 | 2193 | 2212 | UCAAUUGGUAUCAGAGCUUG | CAGGCCCUGAUACCAAUUCA | Cleavage |
| ccp-miR845b-2-5p | Cc09_g03520 | 3.0 | 17.86 | 1 | 21 | 953 | 973 | UCAAUUGGUAUCAGAGCUUGG | UUGGGUUUUGAUACCAGUUGA | Cleavage |
| ccp-miR856-3p | Cc00_g02450 | 3.0 | 13.908 | 1 | 20 | 74 | 93 | UGAUGUUAUCUGUGGUACAA | UUGUCCCACAGAUAAGGUCA | Cleavage |
| ccp-miR856-3p | Cc00_g03670 | 3.0 | 12.471 | 1 | 20 | 395 | 414 | UGAUGUUAUCUGUGGUACAA | UUGUCCCACAGAUAAGGUCA | Cleavage |
| ccp-miR856-3p | Cc00_g12060 | 3.0 | 14.721 | 1 | 20 | 266 | 285 | UGAUGUUAUCUGUGGUACAA | UUGUCCCACAGAUAAGGUCA | Cleavage |
| ccp-miR856-3p | Cc01_g06520 | 3.0 | 15.217 | 1 | 20 | 1991 | 2010 | UGAUGUUAUCUGUGGUACAA | AUGUUCCACAGGUAAUAUCA | Cleavage |
| ccp-miR856-3p | Cc01_g16530 | 2.5 | 17.951 | 1 | 20 | 487 | 506 | UGAUGUUAUCUGUGGUACAA | AUGUGCCAGAGAUAACAUCA | Cleavage |
| ccp-miR856-3p | Cc02_g06020 | 3.0 | 15.448 | 1 | 20 | 1348 | 1367 | UGAUGUUAUCUGUGGUACAA | UUGUCCCACAGAUAAGGUCA | Cleavage |
| ccp-miR856-3p | Cc06_g02210 | 3.0 | 18.722 | 1 | 20 | 873 | 892 | UGAUGUUAUCUGUGGUACAA | UUUUGCUGCAGAUAAUAUCA | Cleavage |
| ccp-miR856-3p | Cc06_g09360 | 3.0 | 11.241 | 1 | 20 | 1062 | 1081 | UGAUGUUAUCUGUGGUACAA | UUUUACCACGAAUAAUAUCA | Translation |
| ccp-miR856-3p | Cc11_g01460 | 3.0 | 14.781 | 1 | 21 | 661 | 681 | UGAUGUUAUCUGUGGUACAAA | UUUGUACCACAAAAAACAUCU | Translation |
| ccp-miR8578-5p | Cc00_g00860 | 3.0 | 15.402 | 1 | 20 | 1088 | 1107 | UUUAUGAGAGAUCUUUCCAA | UUGGAAAUCUCUCUCAUAAU | Cleavage |
| ccp-miR8578-5p | Cc00_g12350 | 3.0 | 21.56 | 1 | 21 | 447 | 467 | UUUAUGAGAGAUCUUUCCAAC | GUUGGGAAAAAUUCUCAUAAA | Translation |
| ccp-miR8578-5p | Cc01_g11030 | 2.5 | 21.109 | 1 | 20 | 909 | 928 | UUUAUGAGAGAUCUUUCCAA | UUGGAAAGAUUUUUCACAAA | Cleavage |
| ccp-miR8578-5p | Cc07_g17040 | 3.0 | 17.869 | 1 | 21 | 2844 | 2864 | UUUAUGAGAGAUCUUUCCAAC | GUUAGAAACAUUUUUCAUAAA | Cleavage |
| ccp-miR8578-5p | Cc07_g20920 | 3.0 | 24.725 | 1 | 21 | 447 | 467 | UUUAUGAGAGAUCUUUCCAAC | GUUGGGAAAAAUUCUCAUAAA | Translation |
| ccp-miR8578-5p | Cc11_g09790 | 3.0 | 14.978 | 1 | 20 | 1734 | 1753 | UUUAUGAGAGAUCUUUCCAA | UUGGAGGGGUAUCUCAUAAG | Translation |
| ccp-miR8691-5p | Cc00_g11210 | 3.0 | 3.777 | 1 | 24 | 34 | 57 | AGAUGAUGAGAAGGGUAGGUCAAU | AUGGUCCUACUUUUCCCAUCAUCU | Translation |
| ccp-miR8691-5p | Cc02_g26650 | 3.0 | 18.314 | 1 | 20 | 727 | 746 | AGAUGAUGAGAAGGGUAGGU | ACCUGCUUUGCUCAUCAUUU | Translation |
| ccp-miR8691-5p | Cc02_g35470 | 3.0 | 15.671 | 1 | 23 | 293 | 315 | AGAUGAUGAGAAGGGUAGGUCAA | UUUAUCUAUUCUUCUGAUCAUUU | Cleavage |
| ccp-miR8691-5p | Cc03_g08600 | 3.0 | 7.749 | 1 | 24 | 397 | 420 | AGAUGAUGAGAAGGGUAGGUCAAU | AUGGUCCUACUUUUCCCAUCAUCU | Translation |
| ccp-miR8691-5p | Cc03_g08970 | 3.0 | 9.842 | 1 | 20 | 430 | 449 | AGAUGAUGAGAAGGGUAGGU | ACCUACUUUCCUCAUCGUUU | Translation |
| ccp-miR8691-5p | Cc04_g15220 | 3.0 | 7.184 | 1 | 24 | 1 | 24 | AGAUGAUGAGAAGGGUAGGUCAAU | AUGGUCCUACUUUUCCCAUCAUCU | Translation |
| ccp-miR8691-5p | Cc05_g03670 | 3.0 | 9.117 | 1 | 20 | 525 | 544 | AGAUGAUGAGAAGGGUAGGU | UUCUGUCCUUCUCAUUAUCU | Cleavage |
| ccp-miR8691-5p | Cc05_g10830 | 2.5 | 15.481 | 1 | 20 | 52 | 71 | AGAUGAUGAGAAGGGUAGGU | ACCUACCUUUUGUAUCAUCU | Translation |
| ccp-miR8691-5p | Cc06_g12660 | 3.0 | 14.017 | 1 | 20 | 4867 | 4887 | AGAUGAUG-AGAAGGGUAGGU | ACCUAUCUUUCUUCAUCAUCU | Translation |
| ccp-miR8691-5p | Cc07_g05170 | 2.5 | 8.311 | 1 | 20 | 64 | 83 | AGAUGAUGAGAAGGGUAGGU | CUCUCCCCUUCUCAUCAUCU | Cleavage |
| ccp-miR8691-5p | Cc08_g08030 | 3.0 | 15.434 | 1 | 20 | 2237 | 2256 | AGAUGAUGAGAAGGGUAGGU | ACUUACUCUUCACAUUAUUU | Translation |
| ccp-miR8691-5p | Cc10_g09570 | 3.0 | 7.36 | 1 | 20 | 245 | 264 | AGAUGAUGAGAAGGGUAGGU | ACCAGCCAUUUUCAUCAUCU | Cleavage |
| ccp-miR8691-5p | Cc11_g14320 | 3.0 | 13.966 | 1 | 20 | 839 | 858 | AGAUGAUGAGAAGGGUAGGU | ACUUAUCCUUCUGGUUAUCU | Cleavage |
| ccp-miR8709a-3p | Cc00_g13980 | 3.0 | 16.641 | 1 | 23 | 675 | 697 | AUAUUAAAACUAAACAUGAACAA | UGGUUCAUGUUGAAUUUUGGUAU | Translation |
| ccp-miR8709a-3p | Cc00_g21760 | 3.0 | 12.909 | 1 | 20 | 2128 | 2147 | AUAUUAAAACUAAACAUGAA | UACAUUUUUGGUUUUAAUGU | Cleavage |
| ccp-miR8709a-3p | Cc02_g33070 | 3.0 | 10.811 | 1 | 23 | 36 | 58 | AUAUUAAAACUAAACAUGAACAA | UUUUUUAUGUUCACUUUUGAUAU | Translation |
| ccp-miR8709a-3p | Cc06_g10430 | 3.0 | 13.174 | 1 | 23 | 3723 | 3745 | AUAUUAAAACUAAACAUGAACAA | UUUUUUAAGUUUAGUUUUAGUAC | Cleavage |
| ccp-miR8709a-3p | Cc07_g19300 | 2.5 | 11.919 | 1 | 24 | 576 | 599 | AUAUUAAAACUAAACAUGAACAAA | UUCAUUCAUGUUUAUUUUUCAUAU | Translation |
| ccp-miR8709a-3p | Cc09_g08950 | 2.0 | 13.371 | 1 | 24 | 564 | 587 | AUAUUAAAACUAAACAUGAACAAA | UAUUUUCAUGUUUAGUAUUAGUGU | Cleavage |
| ccp-miR8746-1-5p | Cc02_g19900 | 3.0 | 18.991 | 1 | 21 | 1293 | 1313 | UCCAUAUUUCACAAUCUCUCC | GGAGGGAUGGUAGAAUAUGGA | Translation |
| ccp-miR8746-1-5p | Cc06_g08870 | 3.0 | 19.993 | 1 | 20 | 593 | 612 | UCCAUAUUUCACAAUCUCUC | GACAGAUUGUGAAUUGUGGA | Cleavage |
| ccp-miR8746-1-5p | Cc06_g14130 | 3.0 | 13.806 | 1 | 20 | 822 | 841 | UCCAUAUUUCACAAUCUCUC | GAGGGAUUGUGAUCUAUGGA | Cleavage |
| ccp-miR8746-1-5p | Cc06_g17040 | 3.0 | 11.5 | 1 | 20 | 780 | 799 | UCCAUAUUUCACAAUCUCUC | GAGAGAUAAUGAAAUAUGGC | Cleavage |
| ccp-miR8746-2-5p | Cc02_g19900 | 3.0 | 18.991 | 1 | 21 | 1293 | 1313 | UCCAUAUUUCACAAUCUCUCC | GGAGGGAUGGUAGAAUAUGGA | Translation |
| ccp-miR8746-2-5p | Cc06_g08870 | 3.0 | 19.993 | 1 | 20 | 593 | 612 | UCCAUAUUUCACAAUCUCUC | GACAGAUUGUGAAUUGUGGA | Cleavage |
| ccp-miR8746-2-5p | Cc06_g14130 | 3.0 | 13.806 | 1 | 20 | 822 | 841 | UCCAUAUUUCACAAUCUCUC | GAGGGAUUGUGAUCUAUGGA | Cleavage |
[truncated: 9,885 more chars]
