# Supplementary material for: A genome-wide analysis of the RNA-guided silencing pathway in coffee reveals insights into its regulatory mechanisms
Source: PLoS One. 2017 Apr 27;12(4):e0176333. doi: 10.1371/journal.pone.0176333 (PMC5407642; doi:10.1371/journal.pone.0176333)
Supplement: S5 Table — miRNA in the respective tissue, target description, and number of associated GO terms. (DOCX) [file pone.0176333.s008.docx]

| **miRNA/tissue** | **Target Description** | **Number of GO** |
| --- | --- | --- |
| miR156 stem | SPL12 Squamosa promoter-binding 12 | 14 |
|  | SPL6 Squamosa promoter-binding 6 | 16 |
|  | TGA1W Teosinte glume architecture 1 | 11 |
|  | CSPLF CASP 1F3 | 1 |
|  | 2ABB Serine threonine phosphatase 2A 55 kDa regulatory subunit B beta isoform | 32 |
|  | FBL11 POZ domain-containing FBL11 | 23 |
|  | AP4E AP-4 complex subunit epsilon | 26 |
|  | PYL9 Abscisic acid receptor PYL9 | 11 |
| miR156 leaves | SPL12 Squamosa promoter-binding 12 | 16 |
|  | TGA1W Teosinte glume architecture 1 | 11 |
|  | SPL6 Squamosa promoter-binding 6 | 20 |
|  | CSPLF CASP 1F3 Short= 1F3 | 1 |
|  | 2ABB Serine threonine phosphatase 2A 55 kDa regulatory subunit B beta isoform | 18 |
|  | IAA13 Auxin-responsive IAA13 | 11 |
|  | 2ABB Serine threonine phosphatase 2A 55 kDa regulatory subunit B beta isoform | 32 |
|  | PYL9 Abscisic acid receptor PYL9 | 11 |
|  | AP4E AP-4 complex subunit epsilon | 17 |
|  | PRSP1 | 3 |
|  | IAA13 Auxin-responsive IAA13 | 11 |
| mir172 stem | AP2 Floral homeotic APETALA 2 | 33 |
|  | PTRB Protease 2 | 6 |
|  | IRE1B Serine threonine- kinase endoribonuclease IRE1b | 37 |
|  | KP1 Kinesin KP1 | 57 |
|  | NLP5 inception 5 | 8 |
|  | NLP6 Nodule inception 6 | 8 |
|  | PP181 Pentatricopeptide repeat-containing At2g33680 | 12 |
|  | CER7 Exosome complex component RRP45B | 26 |
|  | NUP96 Nuclear pore complex NUP96 | 23 |
|  | PP287 Pentatricopeptide repeat-containing At3g59040 | 5 |
|  | UPL1E3 ubiquitin-ligase 1 | 57 |
|  | ACLB1ATP-citrate synthase beta chain 1 | 20 |
|  | AAE17 Probable acyl-activating enzyme peroxisomal | 7 |
|  | MPI2 Mannose-6-phosphate isomerase 2 | 19 |
|  | NUA Nuclear-pore anchor | 43 |
|  | RPAP3 RNA polymerase II-associated 3 | 26 |
|  | NLP8 Nodule inception 8 | 8 |
|  | TSS TPR-domain suppressor of STIMPY | 6 |
|  | NLP1 Nodule inception 1 | 7 |
|  | SRRM2 Serine arginine repetitive matrix 2 | 8 |
|  | FRAY2 Serine threonine- kinase fray2 | 43 |
|  | NLP7 Nodule inception 7 | 8 |
|  | CHR25 CHROMATIN REMODELING 25 | 28 |
|  | GMD2 GDP-mannose 4,6 dehydratase 2 | 20 |
|  | CYPH1 Ferruginol synthase | 7 |
|  | C76B6 Geraniol 8-hydroxylase | 17 |
|  | PP332 Pentatricopeptide repeat-containing At4g21190 | 2 |
|  | BH143 Transcription factor bHLH143 | 1 |
|  | ASIL2 Trihelix transcription factor ASIL2 | 2 |
|  | HERC2 E3 ubiquitin- ligase HERC2 | 15 |
|  | PP435 pentatricopeptide repeat-containing chloroplastic | 12 |
|  | RP8HA Disease resistance RPH8A | 34 |
|  | SMAX1 SUPPRESSOR OF MAX2 1 | 15 |
|  | Y4967 Uncharacterized oxidoreductase At4g09670 | 3 |
|  | LMD2A LMBR1 domain-containing 2 homolog A | 5 |
|  | NSE4A Non-structural maintenance of chromosomes element 4 homolog A | 1 |
|  | COPE2 Coatomer subunit epsilon-2 | 13 |
|  | PUR8 Adenylosuccinate lyase | 7 |
|  | SRP72 Signal recognition particle subunit SRP72 | 9 |
|  | PP165 Pentatricopeptide repeat-containing At2g20540 | 11 |
|  | RPC6 DNA-directed RNA polymerase III subunit RPC6 | 4 |
|  | THE1 Receptor kinase THESEUS 1 | 24 |
|  | NFYA1 Nuclear transcription factor Y subunit A-1 | 26 |
|  | ELP3 Elongator complex 3 | 45 |
|  | SF3A1 Probable splicing factor 3A subunit 1 | 6 |
|  | SDI1 SULFUR DEFICIENCY-INDUCED 1 | 4 |
|  | FOLM Folate synthesis bifunctional mitochondrial | 13 |
|  | PI3K1 Phosphatidylinositol 3- root isoform | 36 |
|  | CAPPC Phosphoenolpyruvate carboxylase 2 | 13 |
|  | NLP2 Nodule inception 2 | 5 |
|  | PP356 Pentatricopeptide repeat-containing At4g38150 | 7 |
|  | IRKI IRK-interacting | 4 |
|  | SCR SCARECROW | 26 |
|  | PLDB1 Phospholipase D beta 1 | 32 |
|  | PP178 Pentatricopeptide repeat-containing chloroplastic | 8 |
|  | SUN2 =SUN domain-containing 2 | 17 |
|  | B561J Cytochrome b561 and DOMON domain-containing At5g47530 | 5 |
|  | ATB13 Homeobox-leucine zipper ATHB-13 | 19 |
|  | PPR26 pentatricopeptide repeat-containing At1g09680 | 2 |
|  | PP221 Pentatricopeptide repeat-containing At3g09060 | 2 |
|  | POT7 Potassium transporter 7 | 10 |
|  | ABC1 ABC transporter mitochondrial | 14 |
| mir172 leaves | KP1 Kinesin KP1 | 46 |
|  | PP287 Pentatricopeptide repeat-containing At3g59040 | 5 |
|  | ACLB1 ATP-citrate synthase beta chain 1 | 20 |
|  | NUA Nuclear-pore anchor | 41 |
|  | RPAP3 RNA polymerase II-associated 3 | 25 |
|  | NLP5 Nodule inception 5 | 8 |
|  | SRRM2 Serine arginine repetitive matrix 2 | 8 |
|  | GMD1 GDP-mannose 4,6 dehydratase | 20 |
|  | BH143 Transcription factor bHLH143 | 7 |
|  | PP332 Pentatricopeptide repeat-containing At4g21190 | 2 |
|  | NSE4A Non-structural maintenance of chromosomes element 4 homolog A | 1 |
|  | PUR8 Adenylosuccinate lyase | 7 |
|  | COPE2 Coatomer subunit epsilon-2 ame | 13 |
|  | SRP72 Signal recognition particle subunit SRP72 | 4 |
|  | THE1 Receptor kinase THESEUS 1 | 24 |
|  | SF3A1 Probable splicing factor 3A subunit 1 | 6 |
|  | ELP3 Elongator complex 3 Short | 45 |
|  | SDI1 SULFUR DEFICIENCY-INDUCED 1 | 4 |
|  | PP356 Pentatricopeptide repeat-containing At4g38150 | 7 |
|  | NLP2 Nodule inception 2 | 6 |
|  | IRKI IRK-interacting | 4 |
|  | TSS TPR-domain suppressor of STIMPY | 6 |
|  | PP178 Pentatricopeptide repeat-containing chloroplastic | 7 |
|  | B561P Cytochrome b561 and DOMON domain-containing At5g35735 | 5 |
|  | ATB13 Homeobox-leucine zipper ATHB-13 | 19 |
|  | POT7 Potassium transporter 7 | 11 |
|  | ABC1 ABC transporter mitochondrial | 14 |
|  | IRE1A Serine threonine- kinase endoribonuclease threonine- kinase endoribonuclease | 34 |
|  | PTRB Protease 2 | 6 |
|  | UPL1 E3 ubiquitin-ligase UPL1 | 8 |
|  | CER7 Exosome complex component | 26 |
|  | NUP96 Nuclear pore complex NUP96 | 23 |
|  | AAE17 Probable acyl-activating enzyme peroxisomal | 6 |
|  | NLP7 Nodule inception 7 | 8 |
|  | GMD2 GDP-mannose 4,6 dehydratase | 20 |
|  | C76B6 Geraniol 8-hydroxylase | 17 |
|  | SMXL2SMAX1-LIKE 2 | 4 |
|  | ASIL2 Trihelix transcription factor ASIL2 | 2 |
|  | HERC2 E3 ubiquitin- ligase | 15 |
|  | PP435 pentatricopeptide repeat-containing chloroplastic | 12 |
|  | Y4967 Uncharacterized oxidoreductase At4g09670 | 3 |
|  | LMD2A LMBR1 domain-containing 2 homolog A | 5 |
|  | RPC6 DNA-directed RNA polymerase III subunit RPC6 | 4 |
|  | NFYA1 Nuclear transcription factor Y subunit A-1 | 26 |
|  | FOLM Folate synthesis bifunctional mitochondrial | 11 |
|  | PI3K1 Phosphatidylinositol 3- root isoform | 36 |
|  | CAPPC Phosphoenolpyruvate carboxylase 2 (PEPCase 2) | 13 |
| miR390 stem | AKR2 Probable aldo-keto reductase 2 | 14 |
|  | RKF1 Probable LRR receptor-like serine threonine- kinase R Receptor-like kinase in flowers 1 | 19 |
|  | RHA1B E3 ubiquitin- ligase RHA1B | 16 |
|  | CDC73 PLANT HOMOLOGOUS TO PARAFIBROMIN | 30 |
| miR390 leaves | RHA1B E3 ubiquitin- ligase RHA1B | 16 |
|  | CDC73 PLANT HOMOLOGOUS TO PARAFIBROMIN | 30 |
|  | AKR2 aldo-keto reductase 2 | 14 |
